# Cobalt/Photoredox Catalyzed Desymmetrization of Oxo and Azabicycles via Asymmetric Reductive Coupling With Alkynes

**DOI:** 10.1002/advs.202523407

**Published:** 2026-06-26

**Authors:** Subhankar Pradhan, Arko Saha, Bholanath Maity, Sayan Dutta, Luigi Cavallo, Basker Sundararaju

**Affiliations:** ^1^ Department of Chemistry Indian Institute of Technology Kanpur Kanpur Uttar Pradesh India; ^2^ Physical Science and Engineering Division King Abdullah University of Science and Technology (KAUST) Thuwal Saudi Arabia

**Keywords:** cobalt, desymmetrization, hydrovinylation, photocatalysis, reductive coupling

## Abstract

Bicyclo[2.2.1]heptane frameworks represent a privileged structural motif prevalent in numerous natural products and bioactive molecules. We report a one‐step, highly enantioselective and diastereoselective reductive protocol for the synthesis of oxa‐ and aza‐bicyclo[2.2.1]heptanes, enabled by [cobalt]/[photoredox] (Co/PC) catalysis. This methodology exploits heterobicyclic strained olefins as *π*‐coupling partners to directly access enantiomerically enriched bicyclo[2.2.1]heptanes from a wide range of alkynes, including terminal alkynes, propargylic alcohols, and internal alkynes. Mechanistic investigations, supported by DFT calculations, reveal that the oxidative coupling between the alkyne and alkene constitutes both the enantio‐ and rate‐determining step. DIPEA serves as an efficient electron‐donor, while the in situ‐generated secondary ammonium salt acts as a competent proton source.

## Graphical abstract

A one‐step, asymmetric reductive coupling of alkynes and oxa‐ and aza‐bicyclic olefins using a cobalt/photoredox catalytic system through desymmetrization.

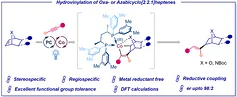

## 1 Introduction

**SCHEME 1:**
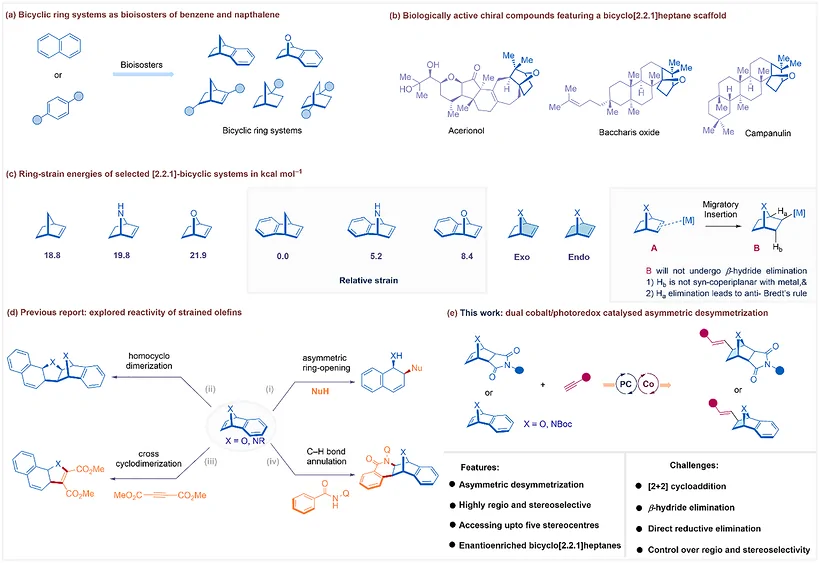
Overview of synthetic approaches to access enantiopure bicyclo[2.2.1]heptanes.

The catalytic enantioselective construction of chiral molecules remains a central challenge in organic chemistry, with broad implications for pharmaceutical development. More than half of approved small‐molecule drugs contain carbon‐centered stereogenic units [1], underscoring the need for efficient catalytic methods to construct these architectures with high fidelity. Beyond stereocontrol, increasing attention has turned to molecular topology, as rigid, saturated bicyclic scaffolds offer distinct advantages over planar aromatic rings—enhancing solubility, metabolic stability, and overall drug‐like properties [2, 3, 4]. Consequently, bridged bicyclic scaffolds have gained prominence as bioisosters for a variety of aromatic systems [4]. For instance, bicyclic[2.2.1]heptanes serve as bioisosters for 1,4‐disubstituted benzene systems, while 1,4‐ epoxynaphthalenes act as bioisosters for naphthalene systems (Scheme 1a) [5]. The synthesis of these scaffolds with high stereochemical and regiochemical precision is essential for their successful incorporation into drug candidates. Among these, oxabicyclo[2.2.1]heptane cores are especially noteworthy, as they appear in a range of biologically active natural products and alkaloids (Scheme 1b) [6, 7]. Representative compounds include Acerionol, a topical antiseptic; Baccharis oxide, used in the treatment of inflammatory skin conditions; and Campanulin, which exhibits anticancer activity. Despite their growing significance, general and modular catalytic approaches for the enantioselective synthesis of functionalized bicyclo[2.2.1]heptanes with high regioselectivity remain scarce. Developing such methods would provide valuable access to structurally diverse and medicinally relevant scaffolds, opening new avenues in drug discovery and molecular design.

Bicyclic olefins are inherently strained molecular systems, and the incorporation of olefinic units (ranging from 0 to 2) within the bicyclic framework significantly increases the overall ring strain energy. Among these, heterobicyclic olefins—particularly those bearing a heteroatom at the bridgehead position—exhibit even greater strain compared to their all‐carbon analogues (Scheme 1c). The presence of a heteroatom at the bridgehead further destabilizes the system due to deviations from ideal bond angles and increased electronic repulsion. These molecules display a distinct diastereofacial preference during coordination to transition metals. Specifically, the sterically less hindered exo face is generally favored over the endo face (Scheme 1c). This preference is further enhanced by the electronic influence of the heteroatom, which facilitates olefin coordination to the metal center. Upon coordination, subsequent migratory insertion may occur, which not only initiates the transformation but also relieves ring strain by releasing non‐optimal bond angles enforced by the rigid bicyclic scaffold (Scheme 1c, right). The resulting metal–alkyl intermediates upon migratory insertion are resistant to *β*‐hydride elimination due to two main factors: (1) the lack of a *syn*‐coplanar *β*‐hydrogen (*H_b_*), and (2) the geometric constraints at the bridgehead position (*H_a_*) (Scheme 1c), which prohibit *β*‐elimination due to Bredt's rule. Consequently, these in situ‐generated alkyl–metal species have been effectively utilized in a range of catalytic transformations, enabling the formation of highly substituted ring systems bearing multiple stereocenters in a single step. The inherent strain and stereoelectronic features of these intermediates render them particularly reactive toward ring‐opening and addition reactions in the presence of suitable nucleophilic or electrophilic partners. The seminal work of the Lautens group on the Pd‐catalyzed reaction of oxabicyclic alkenes with dialkylzinc reagents [8, 9, 10] laid the foundation for subsequent developments in desymmetrization using various hard carbon nucleophiles, such as organometallic reagents and terminal alkynes, leading to either ring‐opening [11, 12, 13, 14, 15, 16, 17, 18, 19, 20, 21, 22, 23, 24, 25, 26, 27, 28, 29, 30, 31, 32, 33, 34, 35], or addition products (Scheme 1di) [17, 23, 24, 32, 35, 36, 37, 38, 39, 40, 41] Building on these early advances, low‐valent nickel [28, 29, 30] and cobalt [32, 33, 34, 35] catalysis was later explored for asymmetric ring‐opening transformations. For instance, Zhao and co‐workers reported a cobalt‐catalyzed reaction between allyltrifluoroborate and an oxabicyclic alkene, in which a chiral diphosphine‐bound cobalt catalyst facilitated enantioselective ring‐opening, while a ligand‐free catalyst, in the presence of a proton source, resulted in racemic hydroallylation [32]. In 2014, the Cheng group disclosed a nickel‐catalyzed reductive coupling of heterobicyclic olefins with activated alkenes, employing a stoichiometric zinc reductant [28]. Analogously, in 2019, the Yoshikai group described cobalt‐catalyzed enantioselective and chemo‐divergent transformations that delivered either an alkylative ring‐opening product or a hydroalkylation product [34]. In this system, the counterion associated with the cobalt catalyst played a decisive role in governing the chemoselectivity of cyclopropanol addition to oxabicyclic alkenes. Another valuable class of transformations involving strained alkenes includes [2+2] cycloadditions, which can yield homo‐ or heterodimerized products bearing multiple stereocenters (Scheme 1dii,iii) [41, 42, 43, 44]. In 2007, Hayashi and the Tam group independently reported the asymmetric homodimerization of bicyclic alkenes using cationic rhodium complexes [41]. In parallel, strained olefins have also been successfully leveraged in asymmetric C–H activation chemistry recently (Scheme 1div) [45, 46, 47, 48, 49]. Despite these advances, a stereo‐ and enantioselective reductive cross‐coupling between heterobicyclic alkenes and alkynes remains unexplored, particularly for the exclusive synthesis of asymmetric hydrovinylated heterobicyclic alkanes. Recently, photoredox/cobalt dual catalysis has emerged as a powerful strategy for constructing complex molecular architectures under mild reaction conditions, while eliminating the need for toxic stoichiometric reducing agents [50, 51]. In this context, we previously reported dual cobalt/photoredox catalytic systems for the reductive coupling of allenyl carbonates with aldehydes [52], as well as allenyl and allyl carbonates with alkynes [53]. Over the last few years, the Xiao group has made significant contributions to the development of asymmetric dual cobalt/photoredox strategies [54, 55, 56, 57], demonstrating their applicability in the reductive coupling of diverse *π*‐components.

Building on our continued interest in low‐valent cobalt/photoredox catalysis, we now report a novel catalytic protocol for the chemo‐, regio‐, and enantioselective reductive coupling of alkynes and bicyclo[2.2.1]heptenes leading to the hydrovinylated product with high enantioinduction [58]. This transformation is compatible with a wide variety of disubstituted alkenyl groups and enables the synthesis of an extensive range of enantioenriched oxa‐ and aza‐substituted bicyclo[2.2.1]heptanes, which represent molecular frameworks that are typically difficult to access through conventional synthetic methods.

## 2 Results and Discussion

We commenced the investigation with 1,4‐dihydro‐1,4‐epoxynaphthalene (**1a**) and phenylacetylene (**2a**) as model substrates, and the results obtained under various catalytic conditions are summarized in Table 1. Using 10 mol% Co(OAc)_2_·4H_2_O, 10 mol% (*R*)‐BINAP (L1), 2 mol% 4CzIPN as photocatalyst, 2 equivalents of DIPEA as electron donor, and 2 equivalents of water in acetonitrile (0.1 m) under an argon atmosphere, the desired hydrovinylated product **3aa** was obtained in 83% isolated yield with enantiomeric ratio (er) of 60:40 and an *E*/*Z* ratio of 83:17 (entry 1). Notably, no side products such as cross‐cyclodimerization or ring‐opening derivatives were detected. Changing the chiral ligand to (*S*)‐BINAP (**L2**) affected the enantioselectivity but had minimal impact on diastereoselectivity or *E*/*Z* ratio (entry 2). Removing water from the reaction mixture notably improved the *E*/*Z* ratio to 98:2, while maintaining similar enantioselectivity (entry 3). Screening a series of other bidentate ligands (**L3**–**L7**) failed to enhance enantioselectivity but favored the formation of the *E*‐isomer, thereby improving diastereoselectivity (entry 4). Significant improvement in enantioselectivity was achieved using alkyl bisphosphine ligands (**L8**–**L9**), with the er reaching 89:11 (entries 5–6). Further optimization by increasing the alkyne (**2a**) concentration to three equivalents and employing a sterically bulkier ligand such as **L9** afforded the product **3aa** in 89% isolated yield with an improved er of 93:7 (entry 7). Solvent screening revealed that, besides acetonitrile, only ethanol supported the reaction, though with diminished efficiency and selectivity (entry 8). Complete optimization conditions are provided in Tables S1–S6. Furthermore, control experiments highlighted the essential roles of the chiral ligand and cobalt catalyst (entry 9), photocatalyst (entry 10), *N*,*N*‐diisopropylethylamine (DIPEA, entry 11), and light (entry 12), as the reaction was entirely suppressed in the absence of any of these components.

**TABLE 1:** Reaction optimization^a^.

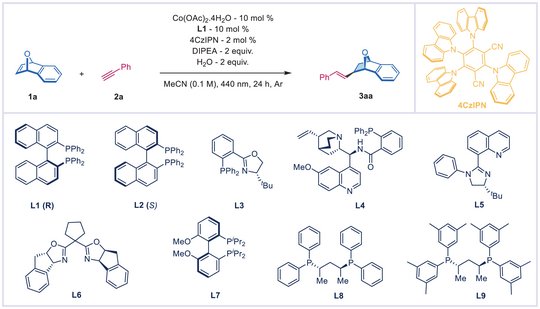				

Entry	Deviations	*E:Z* ratio^b^	Yield (%)^c^	*e.r*.^d^
1	None	83:17	83	60:40
2	**L2** as ligand	88:12	81	40:60
3	Absence of H_2_O	98:2	78	53.5:46.5
4^e^	**L3**–**L7** as ligands	95:5 ‐ >99:1	69–92	50:50–54:46
5^e^	**L8** as ligand	>99:1	63	85:15
6^e^	**L9** as ligand	>99:1	67	89:11
7^e^, ^f^	**L9** as ligand	>99:1	89	93:7
8^e^, ^g^	EtOH as solvent	>99:1	41	80:20
9^e^, ^g^	Absence of [Co] and ligand	—	—	—
10^e^, ^g^	Absence of 4CzIPN	—	—	—
11^e^, ^g^	Absence of DIPEA	—	—	—
12^e^, ^g^	in dark	—	—	—

^a^ All the reactions were carried out in 0.1 mmol of **1a**, 0.2 mmol of **2b**, 0.01 mmol of [Co], 0.01 mmol of ligand, 0.002 mmol of 4CzIPN, 0.2 mmol of DIPEA in 1 mL MeCN (0.1 m) under argon atmosphere upon irradiation at 440 nm for 24 h.

^b^ *E:Z* ratio was determined after analysing the ^1^H NMR of the crude reaction mixture.

^c^ Isolated yield.

^d^ *e.r*. ratio was determined by HPLC on chiral stationary phase.

^e^ Performed in the absence of H_2_O.

^f^ performed with 0.3 mmol of **2a**.

^g^ with **L9** as ligand.

**SCHEME 2:**
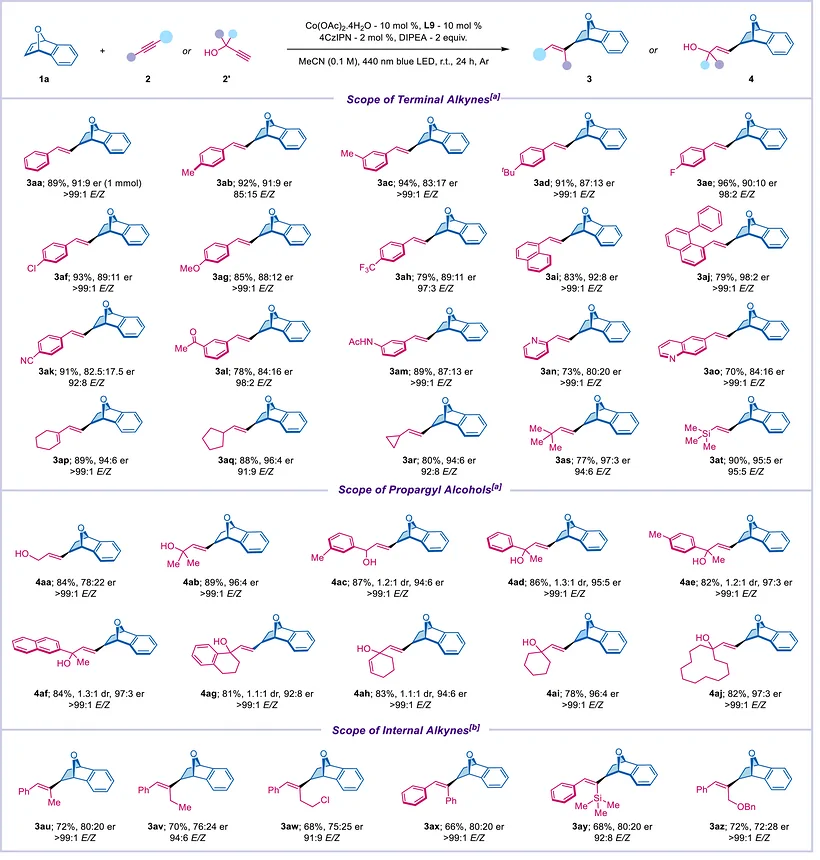
Scope of alkynes. (a) All reactions were carried out using **1a**/**2** or **2’**/Co(OAc)_2_.4H_2_O/**L9**/4CzIPN/DIPEA in 0.1/0.3/0.01/0.01/0.002/0.2 mmol in a closed borosilicate glass vial in MeCN (0.1 m) at room temperature under argon atmosphere. (b) Reaction was carried out for 48 h. (c) e.r for both major and minor isomers is the same. Diastereoselectivities (*dr*) were determined by analyzing the ^1^H NMR of the crude reaction mixture. Enantioselectivities (*er*) were determined by HPLC analysis.

With optimized conditions in hand, we next investigated the scope of alkynes in the desymmetrization reaction, as shown in Scheme 2. To assess the practicality of the method, the reaction was first conducted on a 1.0 mmol, yielding the desymmetrized product **3aa** in 89% isolated yield with an enantiomeric ratio (er) of 91:9. Under optimized conditions, a variety of phenylacetylene derivatives bearing either electron‐donating groups (─Me, ─*^t^* Bu, and ─OMe) or electron‐withdrawing groups (─F, ─Cl, and ─CF_3_) at different positions on the aromatic ring successfully furnished the corresponding products (**3ab**–**3ah**) in good yields, with enantioselectivities reaching up to 91:9 er. The influence of peri‐substitution was also examined under standard conditions, revealing that increased steric bulk near the reaction site enhanced enantioselectivity, delivering products **3ai** and **3aj** with up to 98:2 er. Importantly, the reductive desymmetrization protocol demonstrated excellent functional group tolerance, including reducible functional groups such as nitrile (**2k**), ketone (**2l**), and acetamide (**2m**), affording the corresponding products (**3ak**–**3am**) in good yields and moderate enantiopurity. Alkynes bearing potentially coordinating heterocycles, such as pyridine (**2n**) and quinoline (**2o**), were also well tolerated under the optimized conditions, delivering the corresponding products (**3an**–**3ao**) with moderate er. Notably, the reaction of an enyne substrate demonstrated the strategy's high chemoselectivity for alkyne over alkene coupling, affording dienylated product **3ap** in 86% isolated yield with a 94:6 enantiomeric ratio. Additionally, coupling with trimethylsilylacetylene proceeded smoothly, furnishing product **3at** with excellent enantioselectivity (95:5 er).

The desymmetrization strategy was successfully extended to a broad range of propargyl alcohols. Substitution at the propargylic position proved crucial for achieving high enantioselectivity, likely due to increased steric hindrance adjacent to the *π*‐alkyne system. This effect is evident from the contrasting enantioselectivities observed with prop‐2‐yn‐1‐ol (**2a’**) and 2‐methylbut‐3‐yn‐2‐ol (**2b’**). Propargylic alcohols derived from aromatic aldehydes and ketones underwent smooth reductive coupling to furnish chiral allylic alcohols (**4ac**–**4af**) in good yields and with enantiomeric ratios (er) of up to 97:3 with moderate dr. Substrates derived from α‐tetralone and cyclohexanone also proved compatible, delivering enantioenriched oxabicyclo[2.2.1]heptanes (**4ag**–**4ah**) in good yields. Further, the methodology can be extended to cyclic propargyl alcohols, which underwent reductive coupling to give the corresponding chiral allylic alcohols (**4ai**–**4aj**) with excellent enantioselectivity (er up to 97:3). We next undertook the challenge of employing internal alkynes in the desymmetrization reaction, as their reactivity is traditionally lower than that of terminal alkynes under reductive Co/photoredox conditions. This study aimed to realize both efficient reactivity and high levels of enantio‐ or diastereoselectivity with internal alkynes under the optimized reaction conditions. Initial screening revealed that the reaction proceeded sluggishly with internal alkynes, affording the hydrovinylated product **3au** in only 35% yield after 24 h. Extending the reaction time to 48 h improved the outcome, delivering **3au** in 72% yield with an enantiomeric ratio of 80:20. This trend of moderate reactivity and enantioselectivity was generally observed for both symmetrical and unsymmetrical internal alkynes, providing regioselective products **3av**–**3az** in 66%–72% yield and 72:28–80:20 er.

**SCHEME 3:**
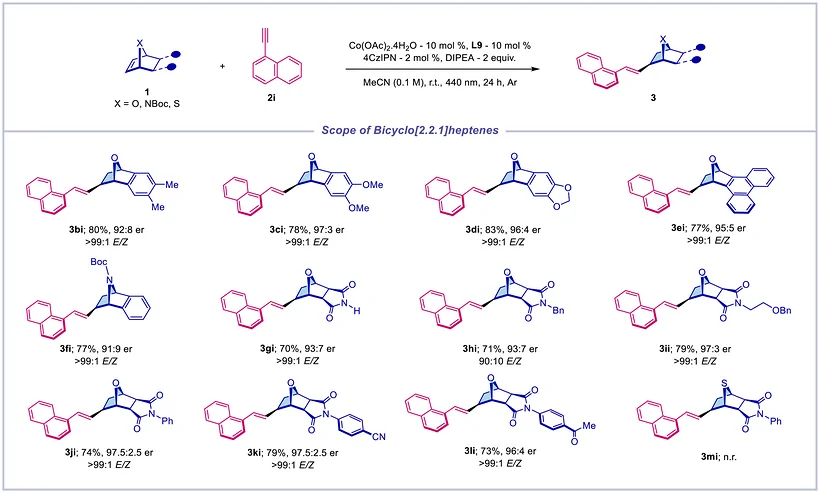
Scope of bicyclo[2.2.1]heptenes. (a) All reactions were carried out using **1**/**2i**/Co(OAc)_2_.4H_2_O/**L9**/4CzIPN/DIPEA in 0.1/0.3/0.01/0.01/0.002/0.2 mmol in a closed borosilicate glass vial in MeCN (0.1 m) at room temperature under argon atmosphere. Enantioselectivities (*er*) were determined by HPLC analysis. **n.r**. = no reaction.

Subsequently, we evaluated a series of bicyclo[2.2.1]heptenes with alkyne **3i** under the established reductive desymmetrization conditions, as shown in Scheme 3. Symmetrical oxabicyclo[2.2.1]heptenes bearing electron‐donating groups (–Me, –OMe, and –OCH_2_O–) delivered the corresponding hydrovinylated products (**3bi**–**3di**) in good yields and with er of up to 94:6. A polyaromatic oxabicyclo[2.2.1]heptene substrate was also adapted, furnishing product **3ei** in 77% yield and 95:5 er. The strategy was further extended by replacing the oxa‐bridge with an aza‐bridge in the strained olefin, resulting in product **3fi** in good yield with 91:9 er, demonstrating the applicability of the protocol to azabicyclo olefins. To prove the role of the aromatic backbone in the bridged bicyclic system, it was substituted with a succinimide moiety. This substrate yielded the hydrovinylated product **3gi** in good yield and with high enantioselectivity (93:7 er), suggesting that the aromatic ring may not be mandatory during the transformation. Encouraged by this result, we explored a range of succinimide‐based substrates, which afforded the corresponding desymmetrized products (**3hi**–**3li**) with excellent enantiomeric ratios, up to 97.5:2.5. Notably, replacing the oxo‐bridge with a thio‐bridge or using substrates lacking a heteroatom at the bridgehead position failed to deliver the desired hydrovinylated products. These observations indicate that a weakly coordinating heteroatom may play a dual role: anchiomerically assisting the enantio‐determining face and stabilizing key cobalt intermediates, thereby likely influencing both reactivity and enantioselectivity.

**SCHEME 4:**
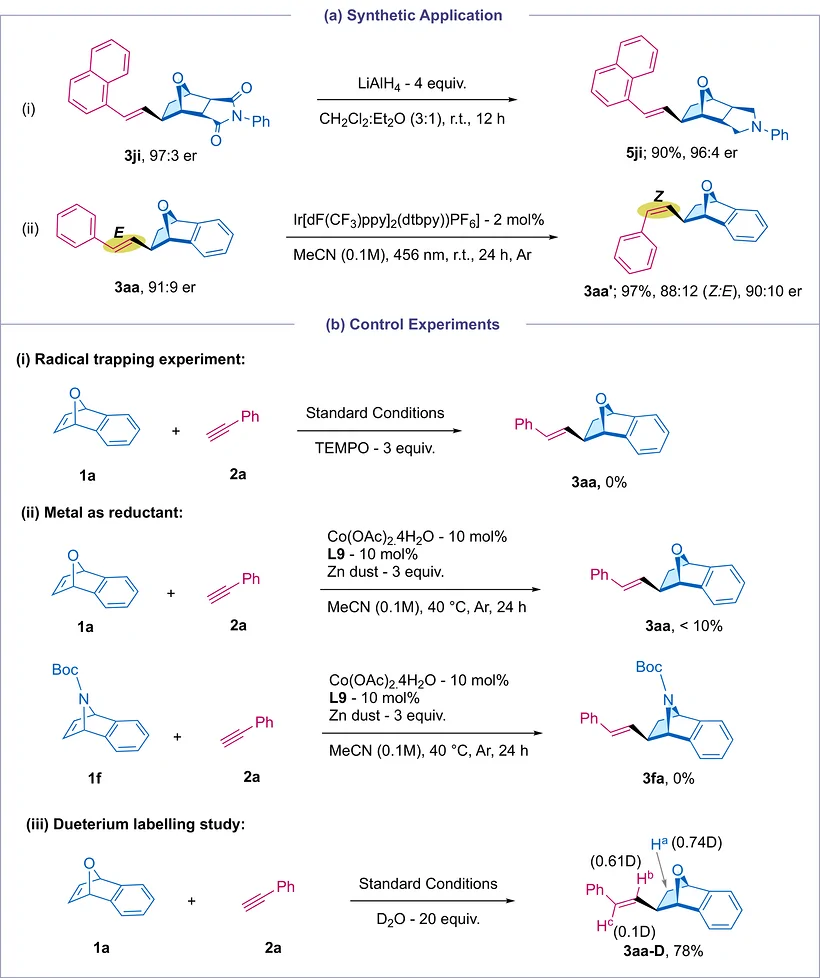
Diversification and control experiments.

To demonstrate the synthetic utility of the developed protocol, late‐stage modifications were carried out as shown in Scheme 4. The succinimide‐derived enantiopure oxa‐bicyclic compound **3ji** underwent smooth amide reduction upon treatment with LiAlH_4_, affording the corresponding *N*‐phenylpyrrolidine‐fused oxa‐bicyclic compound **5ji** in good yield without any loss in enantiopurity (Scheme 4ai). In a separate transformation, the hydrovinylated product **3aa** was subjected to 456 nm light irradiation in the presence of an iridium‐based photosensitizer, enabling *E*‐to‐*Z* alkene isomerization via an energy transfer (EnT) process. The resulting *Z*‐isomer was obtained in good yield with complete retention of the enantiomeric ratio (Scheme 4aii).

To gain deeper insight into the reaction mechanism, we conducted a radical scavenger experiment by adding TEMPO. The addition of TEMPO completely inhibited the catalytic reaction, which is likely attributable to the sequestration of the in situ generated low‐valent cobalt(I) species by the radical scavenger. Additionally, a deuterium labeling experiment was conducted in the presence of 20 equivalents of D_2_O, affording the hydrovinylated product **3aa‐D** in 78% isolated yield with 74% D, 61% D, and 10% D incorporation at the “H^a^,” “H^b^,” and “H^c^” positions, respectively. No deuterium incorporation was observed when the reaction was carried out in CD_3_CN (see the Supporting Information), suggesting that the radical cation of DIPEA exchanged its proton with external D_2_O and subsequently functioned as a proton donor during the catalytic cycle. Subsequently, the reductive coupling reaction was evaluated using a metal‐based reductant (Zn dust); however, this approach did not yield the desired hydrovinylated product, highlighting the broader applicability and effectiveness of the photoredox methodology compared to conventional reductive strategies.

## 3 Computational Studies

To define the complete reaction mechanism and clarify the origin of regio‐, diastereo‐, and enantioselectivity, we carried out density functional theory (DFT) calculations (see Computational Methodology in the Supporting Information). For the calculations, we used substrates **1a** and **2a**, along with Co(OAc)_2_, and 4CzIPN as catalysts. For the ligand, we selected **L8** over the top‐performing **L9** to reduce the computational cost, as its performance is very similar to that of **L9**. We started investigating the mechanistic pathway leading to the formation of the active catalyst **^3^A** (details provided in Figures S13 and S14). The resulting energy profile aligns well with our previously reported finding [53].

**FIGURE 1:**
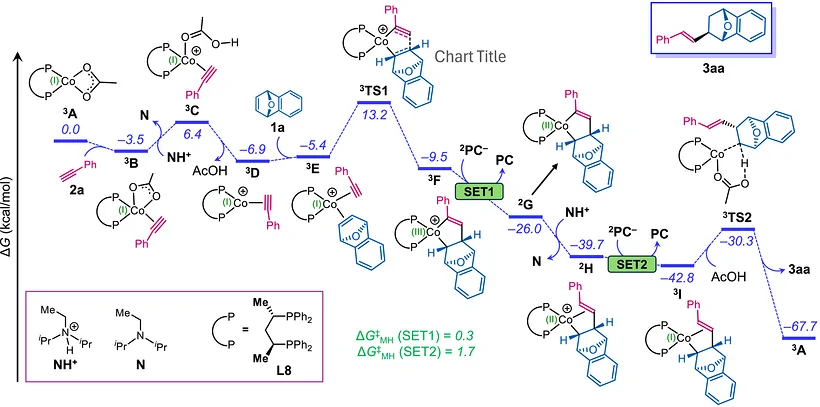
Computed free energy profile for the generation of product **3aa** at the M06(SMD, acetonitrile)/def2‐TZVPP//PBE0‐D3BJ/def2‐TZVP(Co)/def2‐SVP(non‐metals) level of theory. Superscripts 3 and 2 indicate triplet and doublet spin states, respectively, while all other species are in the singlet state unless otherwise specified. **PC** = 4CzIPN.

The cobalt catalytic cycle, illustrated in Figure 1, starts with the coordination of the alkyne **2a** to the active catalyst **^3^A**, an exergonic step releasing 3.5 kcal/mol. The resulting intermediate **^3^B** undergoes barrierless protonation at the carboxylate by **NH^+^**, an endergonic process requiring 9.9 kcal/mol. This is followed by the exergonic liberation of AcOH by 13.3 kcal/mol, which provides the driving force for this overall protonation step, resulting in **^3^D**. The subsequent coordination of the strained olefin **1a** to **^3^D** leads to the formation of adduct **^3^E**. In the next step, the alkyne‐olefin oxidative coupling via migratory insertion requires overcoming a free energy barrier of 20.1 kcal/mol via transition state **^3^TS1**, yielding intermediate **^3^F**. A subsequent exergonic single‐electron reduction of **^3^F** by **^2^PC^–^** (SET1) occurs, producing **^2^G**. This reduction involves a negligible energy barrier of 0.3 kcal/mol (Table S7), estimated using Marcus–Hush theory [59, 60, 61]. The barrierless protonation of **^2^G** gives intermediate **^2^H**, an exergonic step by 13.7 kcal/mol. Another single‐electron reduction (SET2) step, involving **^2^H**, generates **^3^I** with an estimated energy barrier of 1.7 kcal/mol. Finally, protodemetalation assisted by an incoming AcOH molecule releases the desired product, **3aa**, and regenerates the active catalyst **^3^A** through transition state **^3^TS2** with a free energy barrier of 12.5 kcal/mol. Overall, the computed energy profile indicates that the oxidative alkyne‐olefin coupling step via transition state **^3^TS1** is the rate‐limiting step. Two alternative pathways were also investigated and excluded: the first corresponding to protonation at the metal center of **^3^A**, leading to a Co(III)–H species, the second to protonation at the coordinated alkyne in **^3^B**, yielding a Co(III)–vinyl complex (Figure S15). Both routes are thermodynamically unfavorable and involve high free energy barriers; therefore, they were discarded.

**FIGURE 2:**
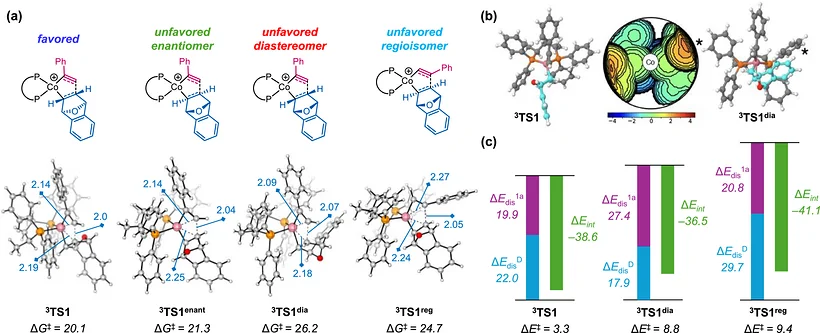
(a) DFT‐optimized geometries of selectivity‐controlling transition states. (b) Steric map of the Co‐complex, the Ph group marked by a ^*^ is pointing upward, clashing with **1a** (cyan) in the unfavored **^3^TS1^dia^**. (c) Results of distortion‐interaction analysis at the M06(SMD, acetonitrile)/def2‐TZVPP level of theory.

As discussed above, **^3^TS1** is identified as the selectivity controlling transition state. Accordingly, we computed the corresponding transition states, **^3^TS1^enant^**, **^3^TS1^reg^**, and **^3^TS1^dia^**, which lead to the enantiomer, regioisomer, and diastereomer that are either unobserved or minor products under experimental conditions. The calculated barriers for those transition states are 1.2, 4.6, and 6.1 kcal/mol higher than that of the favored product (Figure 2a). The relative stability of **^3^TS1** over **^3^TS1^dia^** is mainly due to steric hindrance, as evidenced by analysis of the steric map of the transition state [62, 63], where **1a** clashes with one of the phenyl groups of the ligand in **^3^TS1^dia^** (Figure 2b). In addition, distortion‐interaction analysis [64, 65] was performed on **^3^TS1**, **^3^TS1^reg^**, and **^3^TS1^dia^**, considering **D** and **1a** as the interacting fragments (Figure 2c). The results indicate that although the interaction energy (in absolute value) between the fragments in **^3^TS1^reg^** is significantly higher than that in **^3^TS1**, the distortion energy of the cobalt fragment **D** largely destabilizes the former transition state. On the other hand, the substantial destabilization of **^3^TS1^dia^** relative to ^**3**^ **TS1** is mainly attributed to the higher distortion energy of the strained olefin fragment. In general, the regioselective and enantioselective outcome of the reaction is influenced by the distortion energies of the fragments, consistent with the conclusion that ligand‐substrate steric repulsion governs product selectivity. To further confirm the absolute configuration of the product obtained, we performed circular dichroism both theoretically and experimentally for compound **3ag** in ethanol (for details, Figures S11 and S12). The results obtained further validate the stereochemistry of the major enantiomer obtained in the catalytic process, with an absolute stereochemistry of (1*S*, 2*R*, and 4*R*).

**SCHEME 5:**
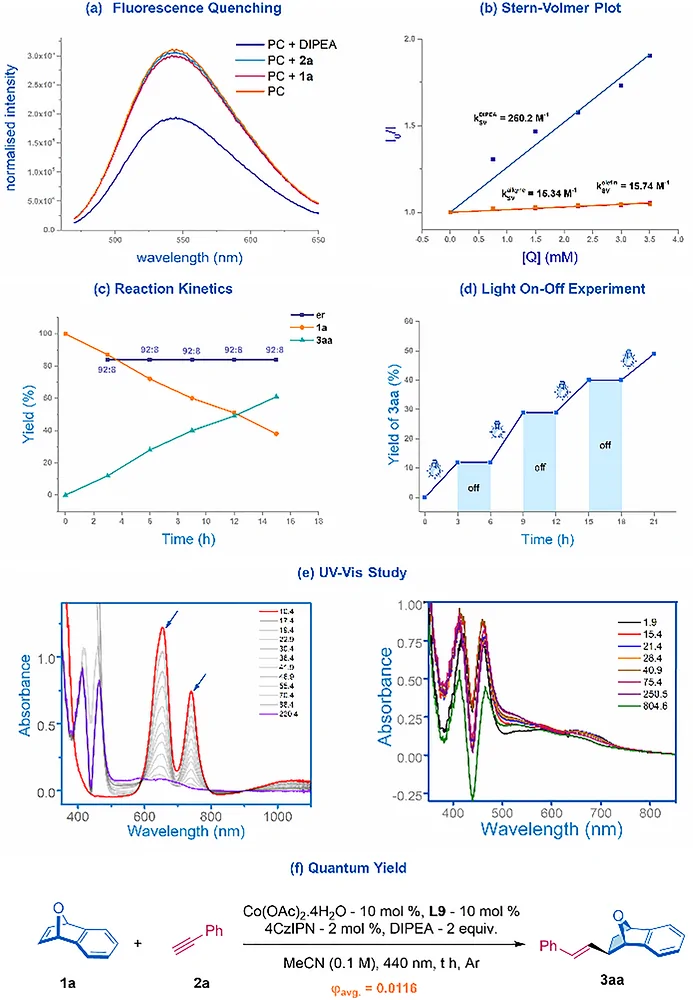
Mechanistic studies.

Additional control experiments were carried out as shown in Scheme 5. Fluorescence quenching studies demonstrated that DIPEA serves as an efficient electron donor, capable of reducing PC^*^ to PC^.−^, as evidenced by the Stern–Volmer plot (Scheme 5a,b). The reaction kinetics were monitored through ^1^H NMR, which revealed that the enantiomeric ratio of the product **3aa** remained unaltered throughout the reaction time (Scheme 5c). Additionally, the light on–off experiment demonstrated that the reaction is not merely photo‐ initiated; rather, continuous irradiation is required throughout the catalytic process (Scheme 5d). The UV–vis kinetic experiments were performed under irradiation at 440 nm, and the results showed that the peaks of parent [Co(II)(**L9**)_2_] at 620 and 710 nm decayed in the presence of the photoredox condition, and new peaks appeared at 410 and 495 nm, which could be of active low‐valent Co(I) intermediate (Scheme 5e). Addition of strained olefin (**1a**) and alkyne (**2a**) to this mixture further helped in the decay of 410 and 495 nm bands, while no strong peak appeared over time. The quantum yield of the desymmetrization reaction was determined to be 0.0116, indicating that the coupling reactions proceed via a photocatalytic pathway rather than a photo‐initiated radical chain mechanism (Scheme 5f).

## 4 Conclusion

This study presents a dual‐catalyzed protocol consisting of a cobalt‐phosphine/photoredox system that enables the efficient and highly selective synthesis of oxo‐ and aza‐bicyclo[2.2.1]heptane frameworks, which are prominent motifs in many biologically active compounds. Utilizing a diverse range of terminal alkynes and strained heterobicyclic olefins, the method proceeds in a single step to afford *E*‐selective alkenylation of bicyclic olefins with excellent enantioselectivity and diastereoselectivity. Internal alkynes afforded only moderate yields and enantioselectivities, while propargylic alcohols bearing a prochiral center showed modest diastereoselectivity. Mechanistic investigations highlight an oxidative coupling between alkyne and alkene as the key enantio‐ and rate‐determining process. The resulting chiral scaffolds are readily diversified through stereocontrolled downstream modifications, illustrating the synthetic utility and broad potential of this transformation for accessing privileged bicyclic architectures of relevance to natural product synthesis and medicinal chemistry.

## Conflicts of Interest

The authors declare no conflicts of interest.

## Supporting information




**Supporting File 1**: advs76210‐sup‐0001‐SuppMat.pdf.

**Supporting File 2**: advs76210‐sup‐0002.cif.

## Data Availability

The data that support the findings of this study are available in the supplementary material of this article.
