# Supplementary material for: Cobalt/Photoredox Catalyzed Desymmetrization of Oxo and Azabicycles via Asymmetric Reductive Coupling With Alkynes
Source: Adv Sci (Weinh). 2026 Jun 26:e23407. Online ahead of print. doi: 10.1002/advs.202523407 (PMC13336453; doi:10.1002/advs.202523407)
Supplement: Supplementary file 1 — Supporting File 1: advs76210‐sup‐0001‐SuppMat.pdf. [file ADVS-9999-e23407-s002.pdf]

Supporting Information

Cobalt/Photoredox Catalyzed Desymmetrization of Oxo and Azabicycles via Asymmetric Reductive Coupling with Alkynes

Subhankar Pradhan,[a] Arko Saha,[a] Bholanath Maity,[b] Sayan Dutta,[b] Luigi Cavallo,*,[b] and Basker Sundararaju*,[a]

[a] Department of Chemistry, Indian Institute of Technology Kanpur, Kanpur-208016, India.

[b]Physical Sciences and Engineering Division, King Abdullah University of Science and Technology (KAUST), Thuwal, 23955-6900, Saudi Arabia.

Email: basker@iitk.ac.in and luigi.cavallo@kaust.edu.sa

Table of Contents **Page No.**

1.	General Information	S2
2.	Synthesis of Starting Materials	S3 – S7
3.	Optimization Studies	S7 – S11
4.	General Procedure C: Catalytic Reaction with Terminal Alkynes	S12
5.	General Procedure D: Catalytic Reaction with Terminal Alkynes	S12
6.	Analytical Data of Compounds	S13 – S44
7.	Synthetic Application	S44 – S45
8.	Control Experiments	S45 – S48
9.	Kinetics Analysis	S48 – S49
10.	Determination of Quantum Yield	S50 – S51
11.	Fluorescence Quenching Study	S51 – S52
12.	UV-Vis Study	S52 – S53
13.	Crystallographic Data	S53 – S54
14.	Circular Dichroism	S54 – S55
15.	Computational Methodology	S55 – S106
16.	References	S106 – S108
15.	NMR Spectra	S109 – S222

1. General Information: Unless otherwise mentioned, all reactions were carried out using 4 mL Borosilicate glass vials under argon atmosphere. Toluene and THF were dried and deoxygenated by distillation over the drying agent (Na-Benzophenone) under nitrogen atmosphere. Other solvents like DMF, dioxane, acetonitrile, acetone, DME were purchased commercially and used as such. ^{1}H, ^{13}C and ^{19}F NMR were recorded on JEOL spectrometers (400 and 500 MHz) using $CDCl_3$ as solvent. Chemical shifts (δ) are given in ppm unit relative to TMS, coupling constants (J) in Hz. The solvent signals were used as references and the chemical shifts were converted to the TMS scale ($CDCl_3$: δ_C = 77 ppm; residual $CHCl_3$ in $CDCl_3$: δ_H = 7.26 ppm). All the reactions were monitored by analytical thin layer chromatography (TLC) using commercial aluminium sheets pre-coated with silica gel. Column Chromatography was conducted using silica gel (Merck, 200-400 mesh) as stationary phase. The fluorescence emission spectra were recorded on a HORIBA Scientific's Fluoromax (steady state and lifetime bench top spectrofluorometer). The ESI-MS spectra were recorded on Agilent 6546LC/Q-TOF mass spectrometer. Deuterated solvents were purchased commercially. UV-Vis kinetics and absorption spectroscopic studies were performed on Agilent 8453 diode-array spectrophotometer to carry out kinetics experiments spectrophotometrically in 1 cm quartz cells (λ= 190–1100 nm range). High Performance Liquid Chromatography (HPLC) analyses were performed on Shimadzu instrument; Model 2010C HT using a chiral stationary phase column (Diacel Co. CHIRALPAK). The chiral HPLC methods were calibrated with the corresponding racemic mixtures.

Photocatalyst (4CzIPN) was prepared according to the reported literature procedure.[1] All other chemicals were purchased and used as such from commercial sources. Commercial Kessil PR160-440nm (max 45 W) blue LED bulbs and PR160 Rig w/Fan kit were purchased from Kessil and utilized for the reaction. Strained olefins (**1a-l**) used for this chemistry were prepared by following known procedure from literature. Alkynes (**2a-h**, **2p-x**, **2a-b**) were purchased from commercial sources and used as such. Other alkynes (**2i-m**, **2o**, **2'c-j**) were synthesized following known literature procedures.

Photocatalytic reactions were performed under Kessil PR160-440nm (max 45 W) blue LED bulbs. In the reaction set up two bulbs were placed (facing towards each other) and on top of it one fan was kept maintaining room temperature during the reaction. A vial stand was used to perform at max 6 reactions at one go. During reactions, the distance between the vials and the light source was 5 cm and the intensity of the bulbs was set to 100 (maximum). The temperature rises to the maximum of 35 °C due to irradiation with two LED bulbs.

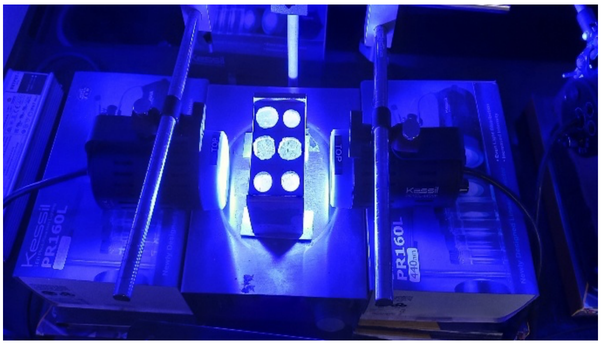

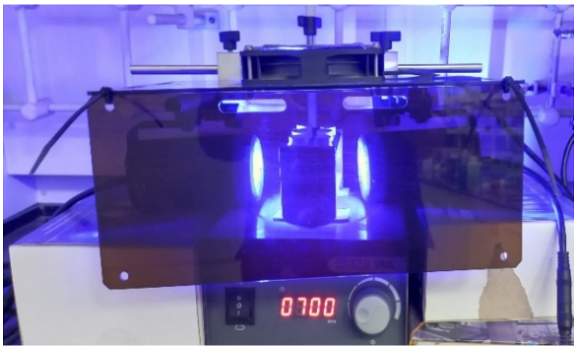

Figure-S1: Pictorial representation of photo reaction set up.

2. Synthesis of Starting Materials

2.1. Synthesis of Alkynes

Alkynes such as **2a-2h**, **2n, 2p-2x**, **2'a**, and **2'b** were purchased from commercial sources and used as such for the catalytic reactions. Other alkynes (**2i-j**,[2] **2k**,[3] **2l-2m**,[4] **2o**,[5] **2y**,[6] **2z**,[7] **2'c-2'j**[8] were prepared according to the previously reported literature procedure and used as such for the catalytic reactions.

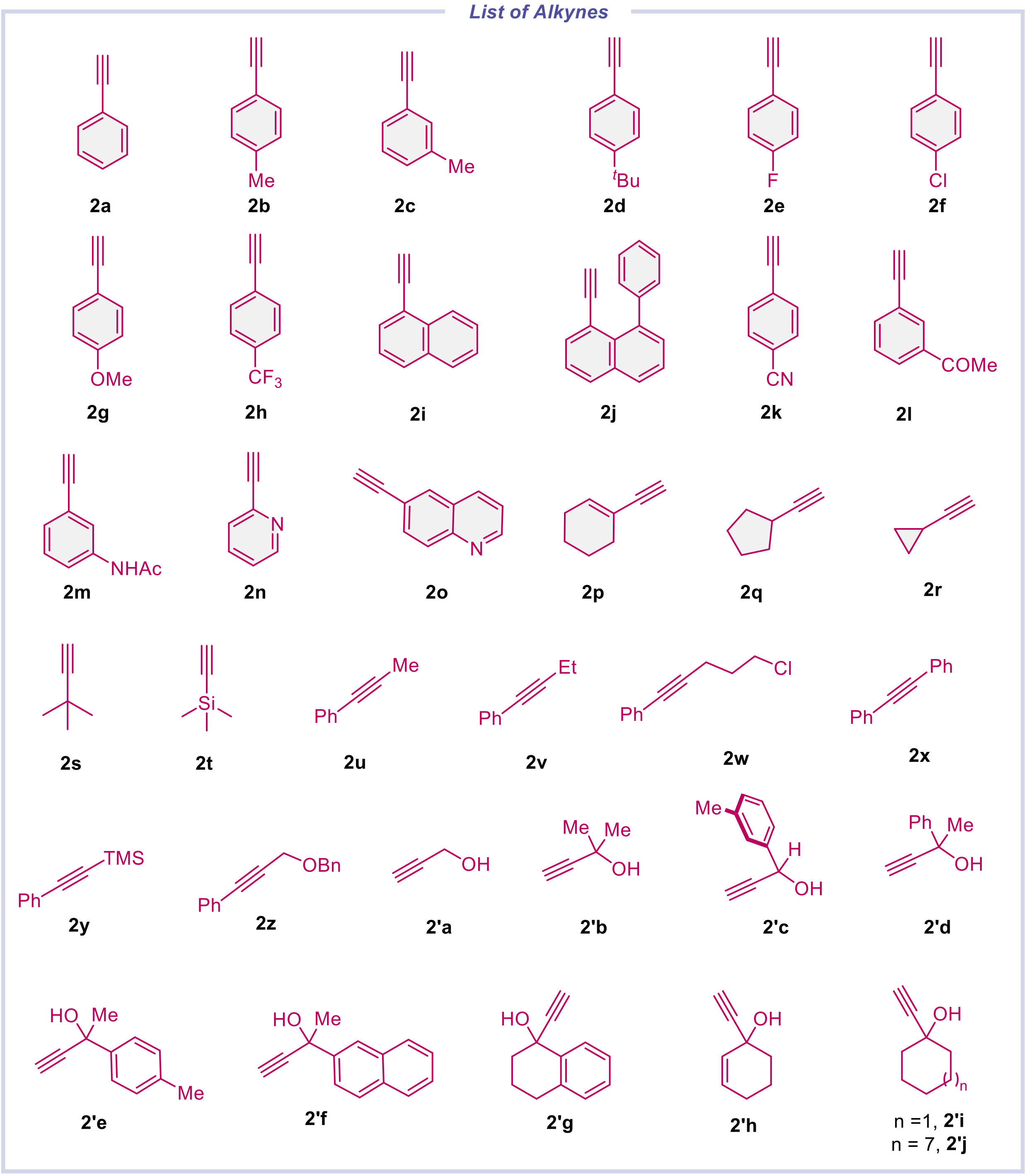

2.2. Synthesis of Oxa/Azabicyclo[2.2.1]heptenes

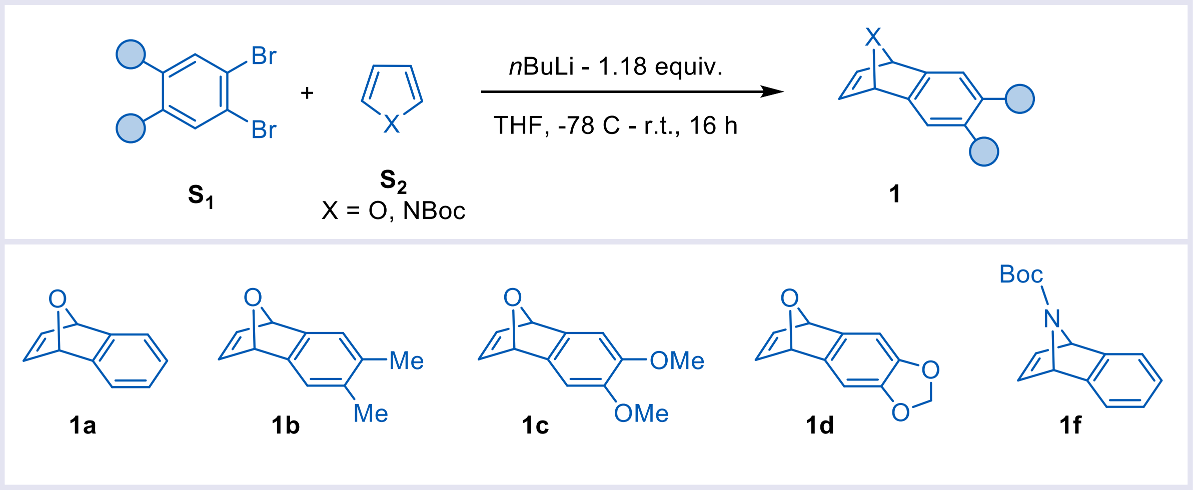

An oven-dried 100 mL round bottom flask equipped with magnetic stirrer, was charged with dibromo benzene derivative (**S1**, 5 mmol, 1 equiv.) and 33 mL dry THF was added under argon atmosphere at room temperature and the reaction mixture was cooled to -78 °C. At this temperature, **S2** (25 mmol, 5 equiv.) was added. To this mixture, *n*-BuLi (1.6 M in THF, 1.18 equiv.) was added dropwise and the reaction mixture was allowed to stir at this temperature for 2 hours and then warmed to room temperature. After 16 hours, the reaction mixture was quenched by saturated solution of NH_4Cl. The reaction mixture was extracted three times with DCM/H_2O; combined organic layer was dried over anhydrous Na_2SO_4, concentrated, and purified by column chromatography (ethyl acetate/hexane = 1:20) on silica gel to afford the desired oxa/azabicyclo[2.2.1]heptenes (**1**), which was used as such for the catalytic reactions.

2.2.1. Synthesis of (4*S*,7*S*)-3a,4,7,7a-tetrahydro-4,7-epoxyisobenzofuran-1,3-dione

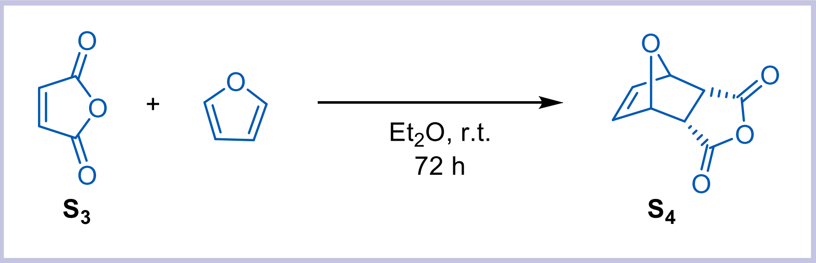

An oven-dried 100 mL two neck round bottom flask equipped with magnetic stirrer, was charged with maleic anhydride (**S_3**, 60 mmol, 1 equiv.), furan (300 mmol, 5 equiv.), and 60 mL dry diethyl ether under argon atmosphere. The reaction mixture was allowed to stir at room temperature for 72 hours and the formation of white precipitate was observed over the reaction time. The resulting precipitate was filtered and was with diethyl ether, then dried in vacuum **S_4** as white solid (8.2 g, 82%). The spectral data of the synthesized compound **S_4** is in accordance with the literature report.[9]

2.2.2 Synthesis of *N*-substituted tetrahydro-1*H*-4,7-epoxyisoindole-1,3(2*H*)-dione: General Procedure A

An oven-dried 250 mL round bottom flask equipped with magnetic stirrer, was charged with *N*-substituted maleimide (**S5**, 25.85 mmol, 1 equiv.), furan (41.0 mmol, 1.6 equiv.) added 90 mL toluene under argon atmosphere. The reaction mixture was stirred at 110 °C for 48 hours. The

reaction mixture was extracted 3 times with ethyl acetate, concentrated, and purified by column chromatography on silica gel (ethyl acetate/hexane = 1:1) to afford desired exo-product. The synthesized exo-products were used as such for the catalytic reactions.

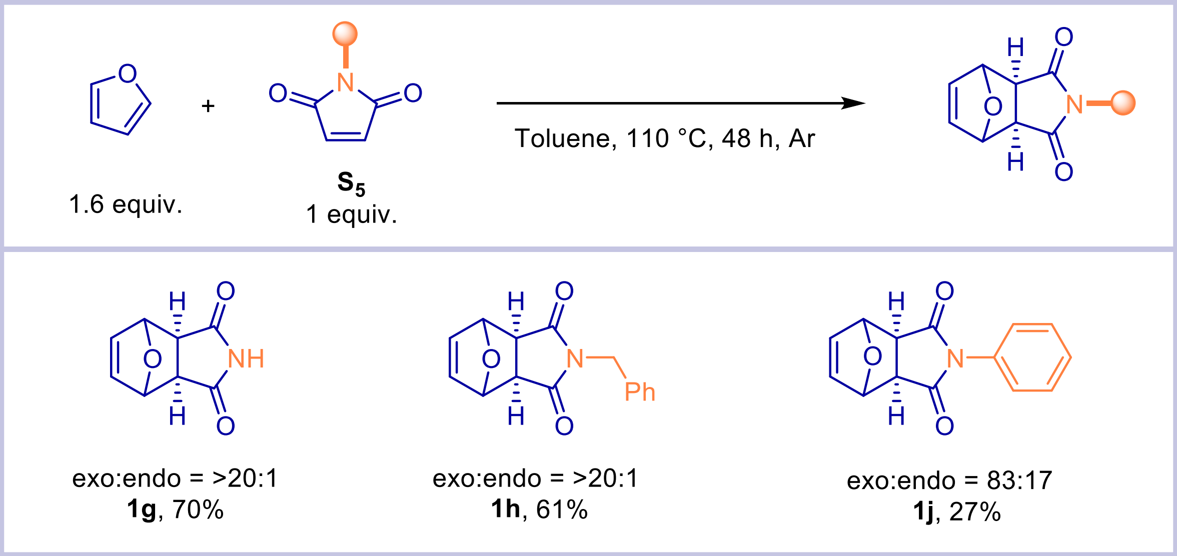

2.2.3 Synthesis of *N*-substituted tetrahydro-1*H*-4,7-epoxyisoindole-1,3(2*H*)-dione: General Procedure B

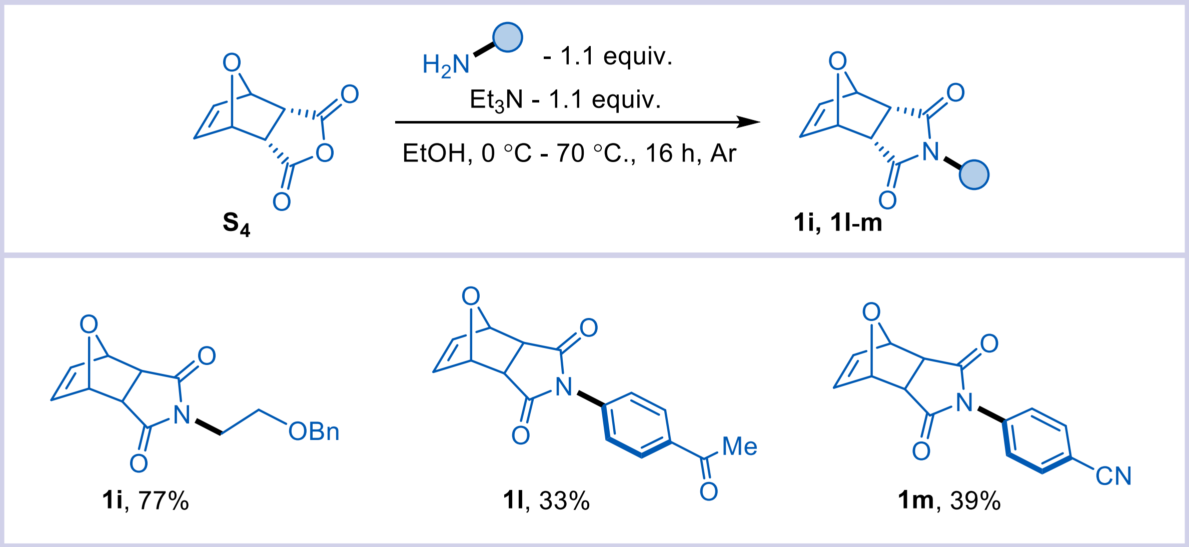

An oven-dried 100 mL two neck round bottom flask equipped with magnetic stirrer, was charged with S_4 (12.0 mmol, 1 equiv.), EtOH (21.5 mL) under argon atmosphere and the reaction mixture was allowed to stir at 0 °C for 5 minutes. To this mixture, a solution of primary amine (13.2 mmol, 1.1 equiv.), triethyl amine (13.2 mmol, 1.1 equiv.) in EtOH (4.3 mL) was added dropwise at the same temperature. The overall reaction mixture was stirred at this temperature for 30 minutes and then transferred to a preheated oil bath for refluxing at 70 °C for 16 hours. After the reaction, ethanol was evaporated, extracted with ethyl acetate, concentrated under vacuum and purified by column chromatography on silica gel (ethyl acetate/hexane = 1:1) to afford the desired product. The products were used as such for the catalytic reactions.

2.2.4 Synthesis of 2-phenyl-3*a*,4,7,7*a*-tetrahydro-1*H*-4,7-epithioisoindole-1,3(2*H*)-dione

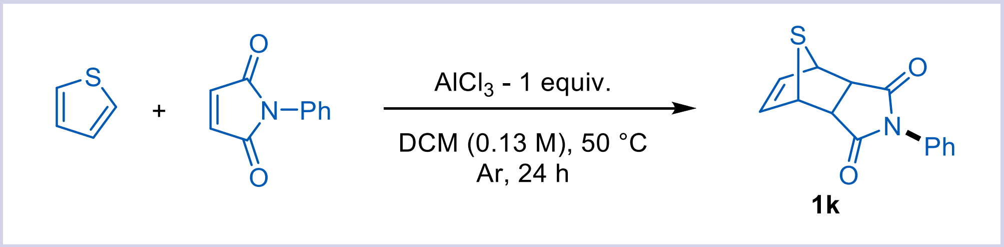

An oven-dried 250 mL two neck round bottom flask equipped with magnetic stirrer, was charged with 1-phenyl-1*H*-pyrrole-2,5-dione (10.4 mmol, 1 equiv.) in 80 mL dry DCM under argon atmosphere. To this solution, $AlCl_3$ (52 mmol, 5 equiv.) was added portion-wise, and the reaction mixture was allowed to stir at room temperature for 30 minutes. To this reaction mixture, thiophene (10.4 mmol, 1 equiv.) was added dropwise and the reaction mixture was shifted to a preheated oil bath to reflux at 50 °C for 24 hours. The reaction mixture was cooled to room temperature and distilled water was added at 0 °C to quench the excess $AlCl_3$. The reaction mixture was then extracted with DCM, concentrated under vacuum, and purified by column chromatography on silica gel (ethyl acetate/hexane = 2:3) to afford the desired product in 80% isolated yield. The synthesised compound was used as such for catalytic reaction.

2-benzyl-3a,4,7,7a-tetrahydro-1*H*-4,7-epoxyisoindole-1,3(2H)-dione (1h): compound **1h** was prepared following general procedure A and the desired product was isolated through column chromatography (ethyl acetate/hexane) in 61% yield as white solid.

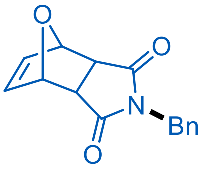

^{1}H NMR ($CDCl_3$, 400 MHz): δ 7.33 – 7.25 (m, 5H), 6.51 (s, 2H), 5.28 (s, 2H), 4.64 (q, J = 7.2 Hz, 2H), 2.85 (d, J = 1.3 Hz, 2H),.

$^{13}C\{^1H\}$ NMR (100 MHz, $CDCl_3$): δ 175.88, 136.53, 135.40, 128.60, 128.10, 127.75, 80.89, 47.48, 42.42.

HRMS (ESI): [$M+H^+$] calculated for $C_{15}H_{14}NO_3^+$ is 256.0968; found 256.0971.

2-(2-(benzyloxy)ethyl)-3a,4,7,7a-tetrahydro-1H-4,7-epoxyisoindole-1,3(2H)-dione (1i): compound **1i** was prepared following general procedure B and the desired product was isolated through column chromatography (ethyl acetate/hexane) in 72% yield as white solid.

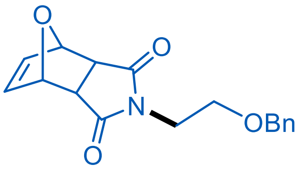

^{1}H NMR ($CDCl_3$, 400 MHz): δ 7.33 – 7.23 (m, 5H), 6.49 (s, 2H), 5.25 (s, 2H), 4.49 (s, 2H), 3.71 (t, J = 5.8 Hz, 2H), 3.61 (t, J = 5.8 Hz, 2H), 2.82 (s, 2H).

$^{13}C\{^1H\}$ NMR (100 MHz, $CDCl_3$): δ 176.10, 137.85, 136.50, 128.32, 127.63, 80.84, 72.65, 66.13, 47.42, 38.33.

HRMS (ESI): [$M+Na^+$] calculated for $C_{17}H_{17}NNaO_4^+$ is 322.1050; found 322.1050.

2-phenyl-3a,4,7,7a-tetrahydro-1H-4,7-epoxyisoindole-1,3(2H)-dione (1j): compound **1j** was prepared following general procedure A and the desired product was isolated through column chromatography (ethyl acetate/hexane) in 44% yield as white solid.

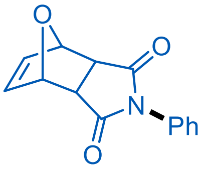

^{1}**H NMR** ($CDCl_3$, 400 MHz): δ 7.47 (dd, J = 8.4, 4.8 Hz, 2H), 7.42 – 7.37 (m, 1H), 7.28 (dd, J = 7.7, 2.3 Hz, 2H), 6.57 (s, 2H), 5.40 (s, 2H), 3.02 (s, 2H).

^{13}**C{^{1}H} NMR** (100 MHz, $CDCl_3$): δ 175.37, 136.66, 131.61, 129.13, 128.77, 126.52, 81.37, 47.49.

HRMS (ESI): [M+H$^+$] calculated for $C_{14}H_{12}NO_3^+$ is 242.0812; found 242.0810.

2-phenyl-3a,4,7,7a-tetrahydro-1H-4,7-epithioisoindole-1,3(2H)-dione (1k): compound **1k** was prepared following general procedure B and the desired product was isolated through column chromatography (ethyl acetate/hexane) in 63% yield as white solid.

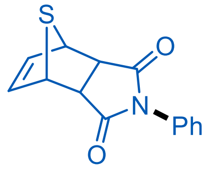

^{1}**H NMR** ($CDCl_3$, 400 MHz): δ 7.47 (tt, J = 8.5, 2.1 Hz, 2H), 7.41 (dt, J = 7.9, 2.1 Hz, 1H), 7.27 (d, J = 2.4 Hz, 1H), 7.25 (s, 1H), 6.64 (t, J = 2.1 Hz, 2H), 4.57 (t, J = 2.4 Hz, 2H), 3.32 (s, 2H).

^{13}**C{^{1}H} NMR** (100 MHz, $CDCl_3$): δ 175.18, 139.92, 131.84, 129.19, 128.88, 126.54, 53.88, 50.11.

HRMS (ESI): [M+H$^+$] calculated for $C_{14}H_{12}NO_2S^+$ is 258.0583; found 258.0575.

2-(4-acetylphenyl)-3a,4,7,7a-tetrahydro-1H-4,7-epoxyisoindole-1,3(2H)-dione (1l): compound **1l** was prepared following general procedure B and the desired product was isolated through column chromatography (ethyl acetate/hexane) in 39% yield as white solid.

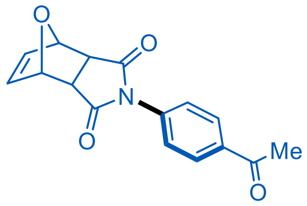

^{1}**H NMR** ($CDCl_3$, 400 MHz): δ 8.05 (d, J = 8.6 Hz, 2H), 7.44 (d, J = 8.5 Hz, 2H), 6.59 (s, 2H), 5.41 (s, 2H), 3.04 (s, 2H), 2.62 (s, 3H).

^{13}**C{^{1}H} NMR** (100 MHz, $CDCl_3$): δ 197.00, 174.88, 136.74, 135.66, 129.11, 126.50, 81.50, 47.63, 26.69.

HRMS (ESI): [M+H$^+$] calculated for $C_{16}H_{14}NO_4^+$ is 284.0917; found 284.0910.

4-(1,3-dioxo-1,3,3a,4,7,7a-hexahydro-2H-4,7-epoxyisoindol-2-yl)benzonitrile (1m): compound **1m** was prepared following general procedure B and the desired product was isolated through column chromatography (ethyl acetate/hexane) in 51% yield as white solid.

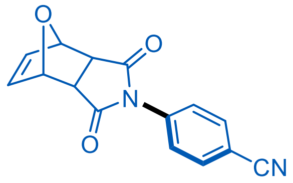

^{1}**H NMR** ($CDCl_3$, 400 MHz): δ 7.75 (d, J = 8.5 Hz, 2H), 7.49 (d, J = 8.5 Hz, 2H), 6.59 (s, 2H), 5.40 (s, 2H), 3.04 (s, 2H).

^{13}**C{^{1}H} NMR** (100 MHz, $CDCl_3$): δ 174.45, 136.74, 135.54, 132.89, 126.98, 117.99, 112.27, 81.53, 47.60.

HRMS (ESI): [M+H$^+$] calculated for $C_{15}H_{11}N_2O_2$+ is 267.0764; found 267.0762.

3. Optimization Studies

3.1. Table S1. Screening of Ligand

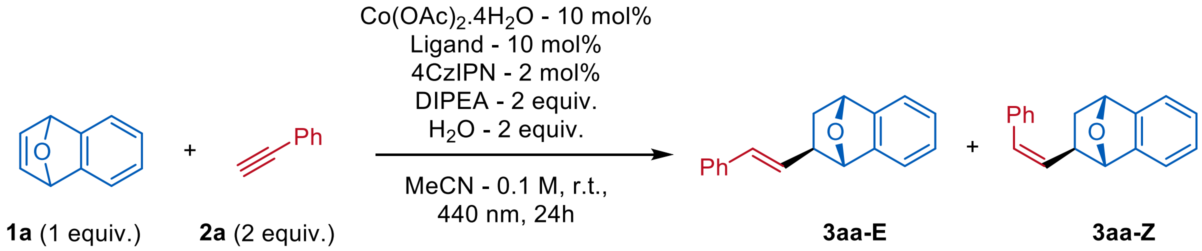

entry	ligand	yield of 3aa (%)	*E:Z* ratio	er of 3aa-E (%)	er of 3aa-Z (%)
1.[a]	**L1**	83	83:17	60:40	64:36
2.[b]	**L2**	72	98:2	40:60	39:61
3.[a]	**L3**	78	71:29	46.5:53.5	47.5:52.5
4.[b]	**L3**	77	98:2	46.5:53.5	48:52
5.[b]	**L4**	70	95:5	51.5:48.5	51.5:48.5
6.[b]	**L5**	92	98:2	51:49	54:46
7.[b]	**L6**	79	>99:1	50:50	-
8.[b]	**L7**	69	>99:1	50:50	-
9.[b,c]	**L8**	63	>99:1	85:15	-
10.[b,c]	**L9**	65	>99:1	89:11	-
11.[b]	**L10**	28	94:6	50:50	50:50
12.[b]	**L11**	trace	-	-	-
13.[b]	**L12**	37	>99:1	50:50	-
14.[b]	**L13**	81	>99:1	50:50	-
15.[b]	**L14**	52	98:2	51.5:48.5	52:48

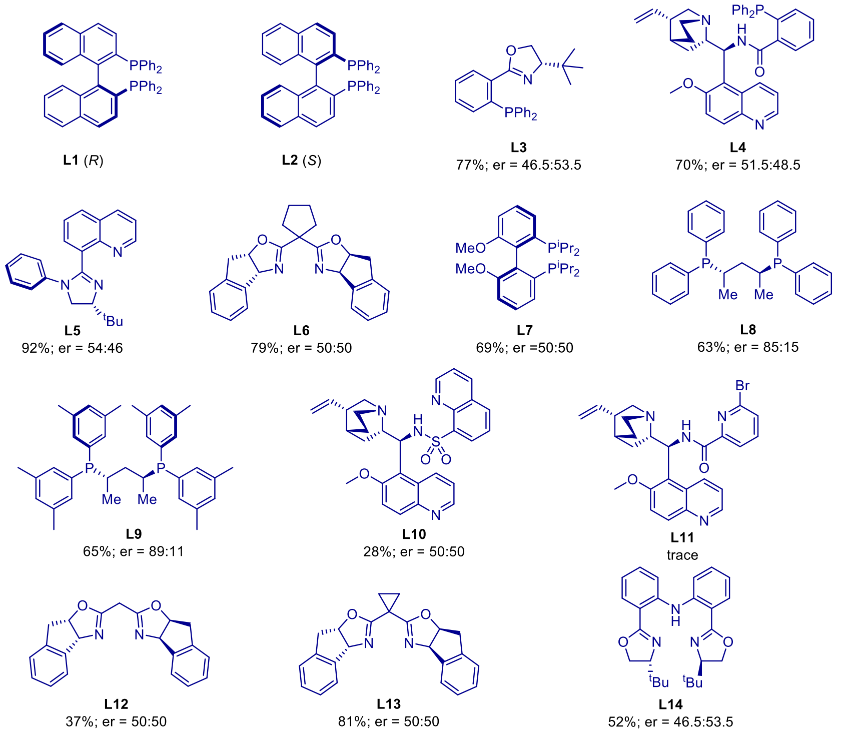

Note: All the reactions were performed considering **1a** (1 equiv.) as limiting reagent, **2a** (2 equiv.), $Co(OAc)_2.4H_2O$ (10 mol%), ligand (10 mol%), 4CzIPN (2 mol%), MeCN (0.1M) as solvent under open air. (a) reactions were performed in presence of H_2O, (b) reactions were performed in absence of H_2O. (c) reactions were performed under argon.

3.2. Table S2. Screening of Photocatalyst

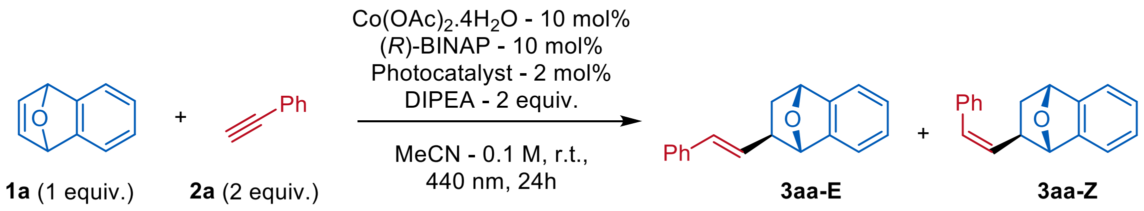

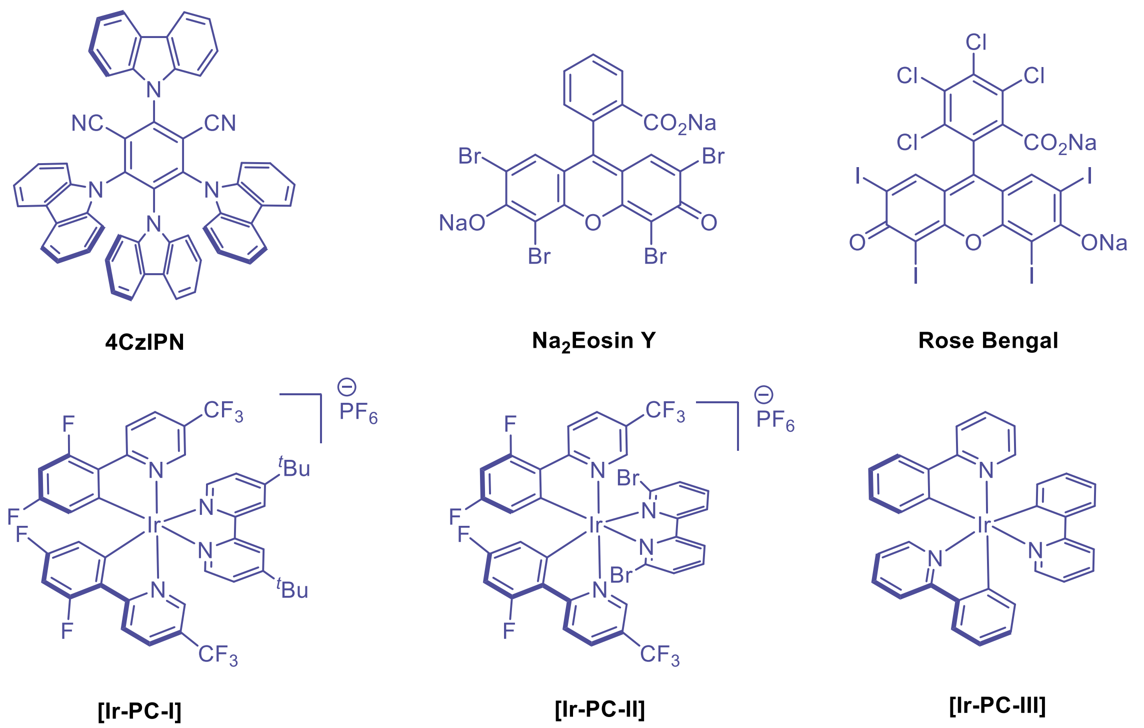

Entry	Photocatalyst	*E/Z* (ratio)	Yield of **3aa** (%)	*er* ratio
1.	4CzIPN	49:1	72	60:40
2.	Na_2EosinY	-	n.o.	-
3.	Rose Bengal	-	n.o.	-
4.	[Ir-PC-I]	1:1.3	62	59:41
5.	[Ir-PC-II]	4.9:1	39	55:45
6.	[Ir-PC-III]	-	n.o.	-

Note: All the reactions were performed considering **1a** (1 equiv.) as limiting reagent, **2a** (2 equiv.), $Co(OAc)_2.4H_2O$ (10 mol%), (*R*)-BINAP (10 mol%), photocatalyst (2 mol%), MeCN (0.1M) as solvent under argon.

3.3. Table S3. Screening of [Co]-salt

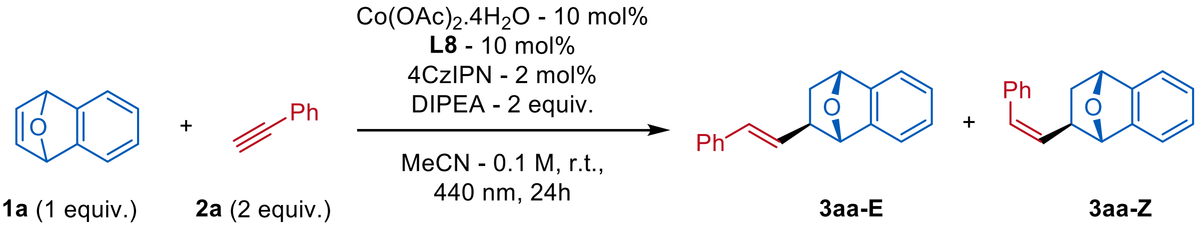

entry	[Co]-salt	yield of 3aa (%)	*E:Z* ratio	er of 3aa (%)
1.	$Co(OAc)_2.4H_2O$	63	>99:1	85:15
2.	$CoCl_2$	29	>99:1	83:17
3.	$CoBr_2$	23	>99:1	76:24
4.	$Co(acac)_2$	54	>99:1	83:17
5.	$Co(ClO_4)_2.6H_2O$	48	>99:1	80:20

Note: All the reactions were performed considering **1a** (1 equiv.) as limiting reagent, **2a** (2 equiv.), $Co(OAc)_2.4H_2O$ (10 mol%), **L8** (10 mol%), 4CzIPN (2 mol%), MeCN (0.1M) as solvent under argon atmosphere.

3.4. Table S4. Screening of Solvent

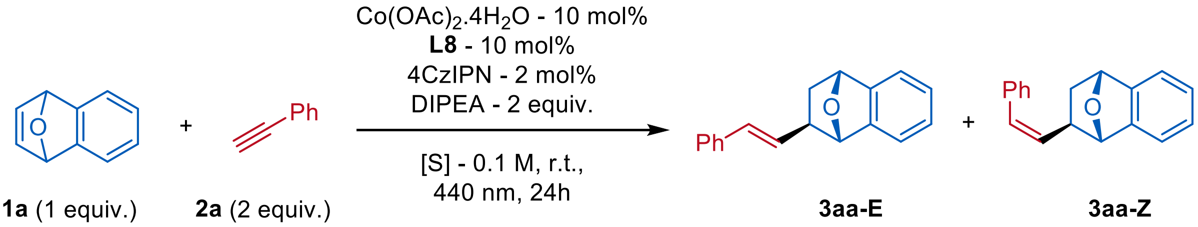

entry	solvent [S]	yield of 3aa (%)	*E:Z* ratio	er of 3aa (%)
1.	MeCN	63	>99:1	85:15
2.	Toluene	trace	-	-
3.	THF	trace	-	-
4.	1,4-Dioxane	trace	-	-

5.	EtOH	39	>99:1	79:21

Note: All the reactions were performed considering **1a** (1 equiv.) as limiting reagent, **2a** (2 equiv.), $Co(OAc)_2.4H_2O$ (10 mol%), **L8** (10 mol%), 4CzIPN (2 mol%) in desired solvent (0.1M) under argon atmosphere.

3.5. Table S5. Screening of Base and Substrate Concentration

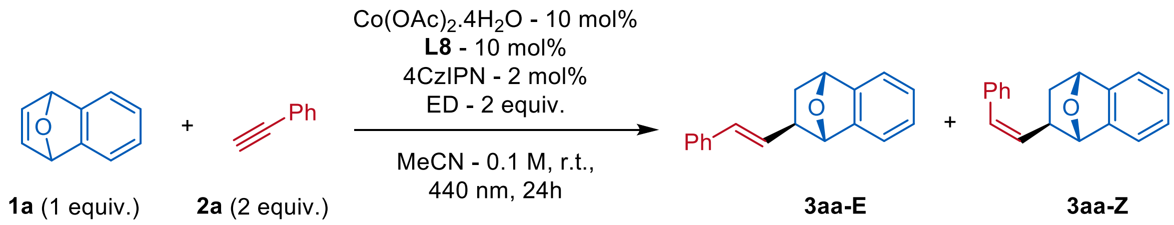

entry	base (equiv.)	yield of 3aa (%)	*E:Z* ratio	er of 3aa (%)
1.	DIPEA(2)	63	>99:1	85:15
2.	DIPEA(3)	72	>99:1	70:30
3.	DIPEA(1)	48	>99:1	83:17
4.	TEA(2)	57	75:25	65:35
5.[a]	DIPEA(2)	85	>99:1	90:10
**6.[a,b]**	**DIPEA(2)**	**89**	**>99:1**	**93:7**
7.[b,c]	DIPEA(2)	88	>99:1	93:7
8.	DABCO(2)	34	>99:1	77:23

Note: [a] 3 equiv. of **2a**; [b] with **L9** as ligand; [c] 4 equiv. of **2a** was used.

3.6. Table S6. Control Experiments

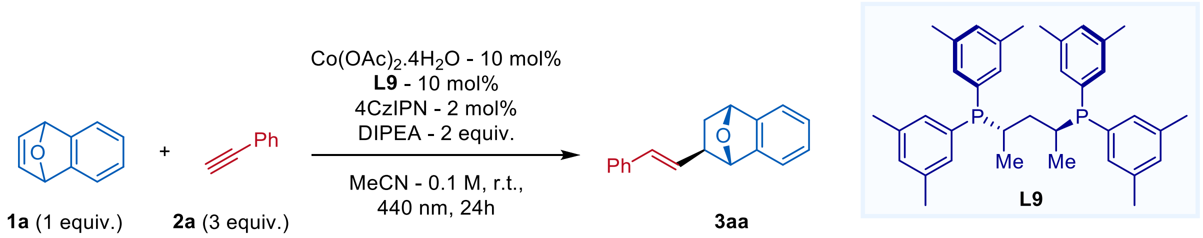

entry	deviations	yield of 3aa (%)	er of 3aa
1.	no change	89	93:7
2.	without 4CzIPN	-	-
3.	without $Co(OAc)_2.4H_2O$ and L12	-	-
4.	without DIPEA	trace	-
5.	in absence of light	-	-

Note: All the reactions were performed considering **1a** (1 equiv.) as limiting reagent, **2a** (2 equiv.), $Co(OAc)_2.4H_2O$ (10 mol%), **L9** (10 mol%), 4CzIPN (2 mol%) in desired solvent (0.1M) under argon atmosphere.

4. General Procedure C: Catalytic Reaction with Terminal Alkynes

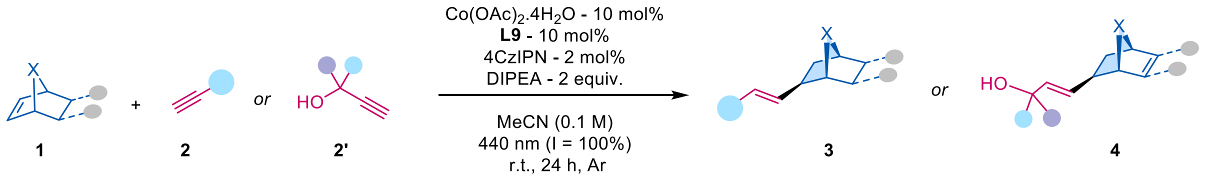

In an oven-dried 4 mL glass vial equipped with a magnetic stir bar, 4CzIPN (0.002 mmol, 2 mol%), ligand **L9** (0.01 mmol, 10 mol%), and $Co(OAc)_2{\cdot}4H_2O$ (0.01 mmol, 10 mol%) were added, and the vial was transferred into a glovebox. Strained olefin **1** (0.1 mmol, 1 equiv.), alkyne **2** or **2′** (0.3 mmol, 3 equiv.), DIPEA (0.2 mmol, 2 equiv.), and MeCN (1 mL, 0.1 M) were subsequently introduced under an argon atmosphere. The vial was sealed with a Teflon-lined cap and irradiated with a Kessil blue LED (λ = 440 nm) at room temperature for 24 h. After completion, volatiles were removed under reduced pressure, and the crude mixture was purified by column chromatography (ethyl acetate/hexane) to afford the desired product (**3** or **4**). The enantiomeric ratio of the isolated products was determined by HPLC analysis in comparison with their racemic counterparts.

5. General Procedure D: Catalytic Reaction with Internal Alkynes

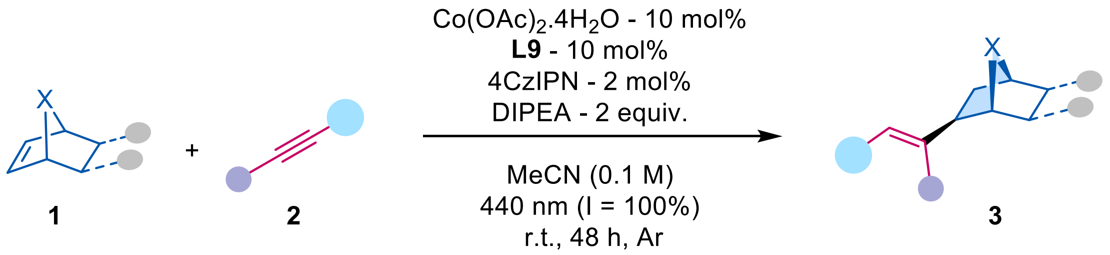

In an oven-dried 4 mL glass vial equipped with a magnetic stir bar, 4CzIPN (0.002 mmol, 2 mol%), ligand **L9** (0.01 mmol, 10 mol%), and $Co(OAc)_2{\cdot}4H_2O$ (0.01 mmol, 10 mol%) were added, and the vial was transferred into a glovebox. Strained olefin **1** (0.1 mmol, 1 equiv.), internal alkyne **2** (0.3 mmol, 3 equiv.), DIPEA (0.2 mmol, 2 equiv.), and MeCN (1 mL, 0.1 M) were subsequently introduced under an argon atmosphere. The vial was sealed with a Teflon-lined cap and irradiated with a Kessil blue LED (λ = 440 nm) at room temperature for 48 h. After completion, volatiles were removed under reduced pressure, and the crude mixture was purified by column chromatography (ethyl acetate/hexane) to afford the desired product (**3**). The enantiomeric ratio of the isolated products was determined by HPLC analysis in comparison with their racemic counterparts.

6. Analytical Data of Compounds

(1*S*,2*R*,4*R*)-2-((*E*)-styryl)-1,2,3,4-tetrahydro-1,4-epoxynaphthalene (3aa): compound **3aa** was prepared following general procedure C and the desired product was isolated through column chromatography (ethyl acetate/hexane = 1:19) in 89% yield with an er of 91:9 as sticky liquid. $[\alpha]_D^{20}$ = +197.34 (c = 0.130, $CHCl_3$).

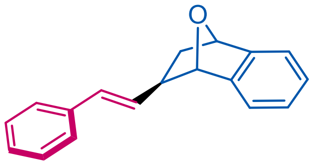

^{1}H NMR ($CDCl_3$, 400 MHz): δ 7.42 – 7.38 (m, 2H), 7.33 (td, *J* = 7.9, 3.9 Hz, 3H), 7.29 – 7.23 (m, 2H), 7.22 – 7.18 (m, 2H), 6.47 (d, *J* = 15.8 Hz, 1H), 6.36 (dd, *J* = 15.8, 9.1 Hz, 1H), 5.49 (d, *J* = 5.0 Hz, 1H), 5.20 (s, 1H), 2.56 (td, *J* = 8.5, 3.7 Hz, 1H), 1.93 (dt, *J* = 11.8, 4.3 Hz, 1H), 1.83 (dd, *J* = 11.8, 8.1 Hz, 1H).

^{13}C{^{1}H} NMR (100 MHz, $CDCl_3$): δ 146.03, 145.17, 137.30, 133.37, 129.88, 128.54, 127.16, 126.71, 126.62, 119.02, 118.91, 84.28, 79.46, 44.12, 35.56.

HRMS (ESI): [M+H$^+$] calculated for $C_{18}H_{17}O^+$ is 249.1274; found 249.1271.

HPLC Condition: The enantiomeric excess was determined by Diacel Chiralpak IA, Hexane/IPA = 98/2, flow rate = 1.00 mL/min, λ = 254 nm, t(minor) = 6.897 min, t(major) = 6.083 min.

Chromatogram of (rac)-3aa

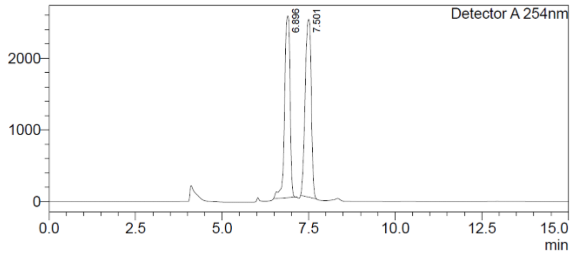

Peak#	Ret. Time	Area	Height	Area%
1	6.896	27880005	2539177	47.969
2	7.501	30241332	2482386	52.031
Total		58121337	5021563	100.000

Chromatogram of (1*S*,2*R*,4*R*)-3aa

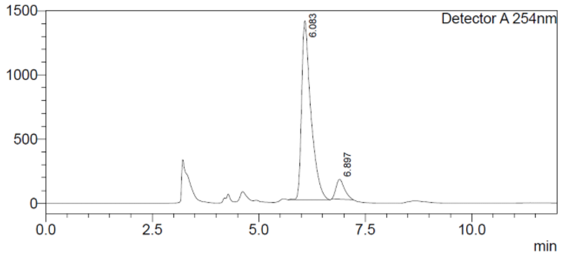

Peak#	Ret. Time	Area	Height	Area%
1	6.083	21191334	1393155	90.770
2	6.897	2154807	156691	9.230
Total		23346141	1549846	100.000

(1*S*,2*R*,4*R*)-2-((*E*)-4-methylstyryl)-1,2,3,4-tetrahydro-1,4-epoxynaphthalene (3ab): compound **3ab** was prepared following general procedure C and the desired product was isolated through column chromatography (ethyl acetate/hexane = 1:19) in 92% (24.1 mg) yield with an er of 91:9 as sticky liquid. $[\alpha]_D^{20}$ = +187.37 (c = 0.166, $CHCl_3$).

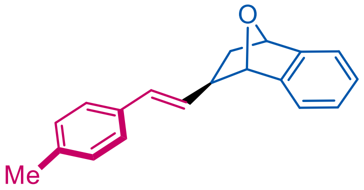

^{1}H NMR ($CDCl_3$, 400 MHz): δ 7.33 – 7.29 (m, 3H), 7.28 – 7.25 (m, 1H), 7.20 (dd, *J* = 5.3, 3.1 Hz, 2H), 7.16 – 7.13 (m, 2H), 6.44 (d, *J* = 14.2 Hz, 1H), 6.31 (dd, *J* = 15.8, 9.2 Hz, 1H), 5.49 (d, *J* = 4.9 Hz, 1H), 5.19 (s, 1H), 2.55 (td, *J* = 8.7, 3.9 Hz, 1H), 2.36 (s, 3H), 1.93 (dt, *J* = 11.9, 4.4 Hz, 1H), 1.83 (dd, *J* = 11.8, 8.1 Hz, 1H).

^{13}C{^{1}H} NMR (100 MHz, $CDCl_3$): δ 146.01, 145.19, 136.92, 134.48, 132.30, 129.69, 129.23, 126.68, 126.60, 125.97, 119.03, 118.90, 84.32, 79.44, 44.11, 35.54, 21.15.

HRMS (ESI): [M+H$^+$] calculated for $C_{19}H_{19}O^+$ is 263.1430; found 263.1431.

HPLC Condition: The enantiomeric excess was determined by Diacel Chiralpak IA, Hexane/IPA = 99/1, flow rate = 1.00 mL/min, λ = 254 nm, t(minor) = 11.877 min, t(major) = 9.676 min.

Chromatogram of (rac)-3ab **Chromatogram of (1*S*,2*R*,4*R*)-3ab**

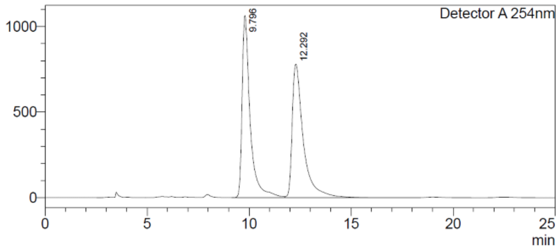

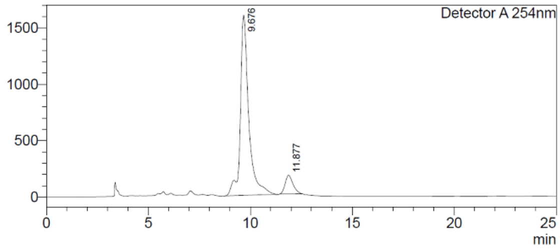

Peak#	Ret. Time	Area	Height	Area%
1	9.796	29261032	1064768	49.750
2	12.292	29555596	780896	50.250
Total		58816628	1845663	100.000

Peak#	Ret. Time	Area	Height	Area%
1	9.676	45866004	1599092	91.104
2	11.877	4478830	165866	8.896
Total		50344834	1764958	100.000

(1*S*,2*R*,4*R*)-2-((*E*)-3-methylstyryl)-1,2,3,4-tetrahydro-1,4-epoxynaphthalene (3ac): compound **3ac** was prepared following general procedure C and the desired product was isolated through column chromatography (ethyl acetate/hexane = 1:19) in 94% (24.6 mg) yield with an er of 83:17 as white solid. $[\alpha]_D^{20}$ = +116.13 (c = 0.155, $CHCl_3$).

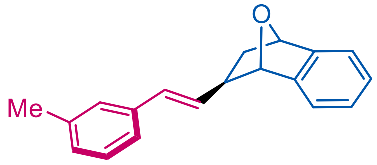

^{1}H NMR ($CDCl_3$, 400 MHz): δ 7.32 – 7.29 (m, 1H), 7.28 – 7.16 (m, 6H), 7.06 (d, *J* = 6.8 Hz, 1H), 6.44 (d, *J* = 15.9 Hz, 1H), 6.35 (dd, *J* = 15.9, 9.1 Hz, 1H), 5.49 (d, *J* = 5.3 Hz, 1H), 5.19 (s, 1H), 2.54 (dd, *J* = 8.3, 3.8 Hz, 1H), 2.36 (s, 3H), 1.93 (dt, *J* = 12.1, 4.5 Hz, 1H), 1.82 (dd, *J* = 12.1, 7.6 Hz, 1H).

^{13}C{^{1}H} NMR (100 MHz, $CDCl_3$): δ 146.01, 145.14, 138.03, 137.20, 133.16, 129.88, 128.42, 127.93, 126.74, 126.67, 126.58, 123.30, 119.01, 118.88, 84.27, 79.43, 44.08, 35.54, 21.36.

HRMS (ESI): [M+H$^+$] calculated for $C_{19}H_{19}O^+$ is 263.1430; found 263.1431.

HPLC Condition: The enantiomeric excess was determined by Diacel Chiralpak IA, Hexane/IPA = 99/1, flow rate = 1.00 mL/min, λ = 254 nm, t(minor) = 13.786 min, t(major) = 9.648 min.

Chromatogram of (rac)-3ac **Chromatogram of (1*S*,2*R*,4*R*)-3ac**

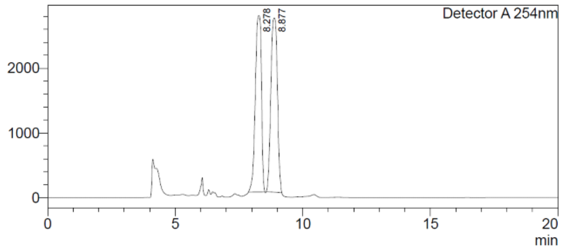

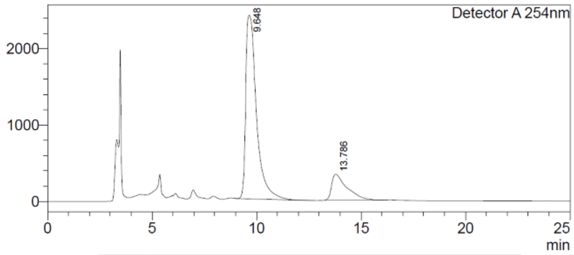

Peak#	Ret. Time	Area	Height	Area%
1	8.278	45284566	2724984	48.706
2	8.877	47690034	2692217	51.294
Total		92974601	5417201	100.000

Peak#	Ret. Time	Area	Height	Area%
1	9.648	85646487	2405603	83.374
2	13.786	17079397	339430	16.626
Total		102725884	2745033	100.000

(1*S*,2*R*,4*R*)-2-((*E*)-4-(*tert*-butyl)styryl)-1,2,3,4-tetrahydro-1,4-epoxynaphthalene (3ad): compound **3ad** was prepared following general procedure C and the desired product was isolated through column chromatography (ethyl acetate/hexane = 1:19) in 91% (27.7 mg) yield with an er of 87:13 as sticky liquid. $[\alpha]_D^{20}$ = +137.16 (c = 0.133, $CHCl_3$).

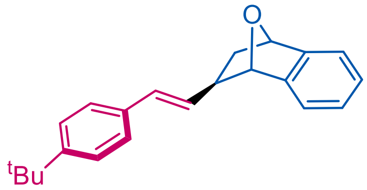

^{1}H NMR ($CDCl_3$, 400 MHz): δ 7.40 – 7.35 (m, 4H), 7.34 – 7.31 (m, 1H), 7.30 – 7.25 (m, 1H), 7.21 (dq, J = 5.4, 2.5 Hz, 2H), 6.51 – 6.44 (m, 1H), 6.40 – 6.30 (m, 1H), 5.50 (t, *J* = 4.1 Hz, 1H), 5.20 (d, *J* = 3.2 Hz, 1H), 2.61 – 2.52 (m, 1H), 1.98 – 1.90 (m, 1H), 1.88 – 1.80 (m, 1H), 1.37 (s, 9H).

$^{13}C\{^1H\}$ NMR (100 MHz, $CDCl_3$): δ 150.19, 146.01, 145.19, 134.48, 132.56, 129.56, 126.65, 125.77, 125.44, 119.01, 118.87, 84.33, 79.42, 44.10, 35.58, 34.49, 31.27.

HRMS (ESI): [$M+H^+$] calculated for $C_{22}H_{25}O^+$ is 305.1900; found 305.1900.

HPLC Condition: The enantiomeric excess was determined by Diacel Chiralpak IB, Hexane/IPA = 99/1, flow rate = 1.00 mL/min, λ = 254 nm, t(minor) = 7.463 min, t(major) = 8.592 min.

Chromatogram of (rac)-3ad

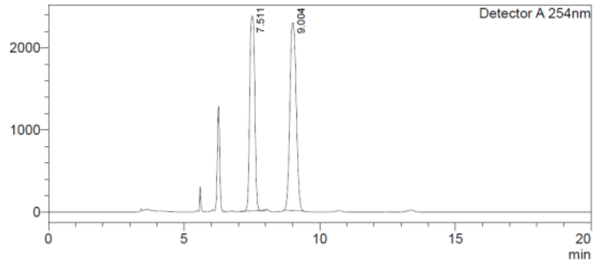

Detector A 254nm

Peak#	Ret. Time	Area	Height	Area%
1	7.511	30608201	2369380	45.206
2	9.004	37100738	2284884	54.794
Total		67708939	4654264	100.000

Chromatogram of (1*S*,2*R*,4*R*)-3ad

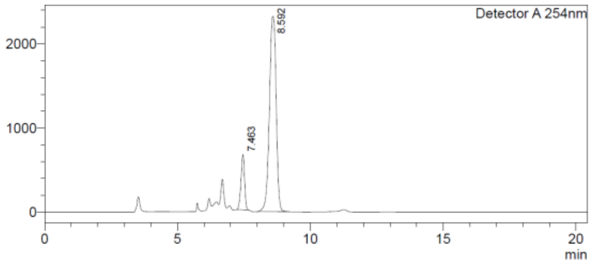

Detector A 254nm

Peak#	Ret. Time	Area	Height	Area%
1	7.463	6162691	657626	13.065
2	8.592	41006753	2322854	86.935
Total		47169444	2980481	100.000

(1*S*,2*R*,4*R*)-2-((*E*)-4-fluorostyryl)-1,2,3,4-tetrahydro-1,4-epoxynaphthalene (3ae):

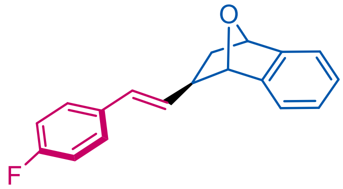

compound **3ae**was prepared following general procedure C and the desired product was isolated through column chromatography (ethyl acetate/hexane = 1:19) in 96% (25.5 mg) yield with an *er* of 91:9 as sticky liquid. $[\alpha]_D^{20}$ = +209.09 (c = 0.11, $CHCl_3$).

^1H NMR ($CDCl_3$, 400 MHz): δ 7.35 (dd, *J* = 8.8, 5.2 Hz, 2H), 7.32 – 7.26 (m, 2H), 7.20 (dd, *J* = 4.9, 3.1 Hz, 2H), 7.01 (t, *J* = 8.5 Hz, 2H), 6.43 (d, *J* = 15.9 Hz, 1H), 6.27 (dd, *J* = 15.9, 9.2 Hz, 1H), 5.49 (d, *J* = 4.9 Hz, 1H), 5.19 (s, 1H), 2.54 (td, *J* = 8.2, 3.7 Hz, 1H), 1.92 (dt, *J* = 12.2, 4.6 Hz, 1H), 1.82 (d, *J* = 19.5 Hz, 1H).

$^{13}C\{^1H\}$ NMR (100 MHz, $CDCl_3$): δ 162.03 (d, *J* = 246.3 Hz), 145.97, 145.06, 133.42 (d, *J* = 3.4 Hz), 133.07 (d, *J* = 2.4 Hz), 128.66, 127.50 (d, *J* = 8.0 Hz), 126.71, 126.61, 119.00, 118.89, 115.38 (d, *J* = 21.6 Hz), 84.21, 79.41, 44.01, 35.51.

$^{19}F\{^1H\}$ NMR (373MHz, $CDCl_3$): δ -114.96.

HRMS (ESI): [$M+H^+$] calculated for $C_{18}H_{16}FO^+$ is 267.1180; found 267.1184.

HPLC Condition: The enantiomeric excess was determined by Diacel Chiralpak IB, Hexane/IPA = 99/1, flow rate = 1.00 mL/min, λ = 254 nm, t(minor) = 9.188 min, t(major) = 9.611 min.

Chromatogram of (rac)-3ae

Chromatogram of (1*S*,2*R*,4*R*)-3ae

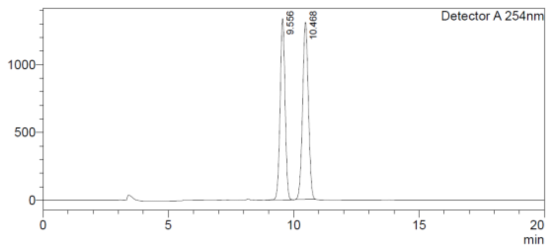

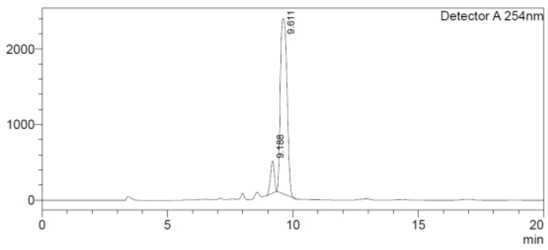

Detector A 254nm

Peak#	Ret. Time	Area	Height	Area%
1	9.556	18427546	1337280	46.860
2	10.468	20896818	1306167	53.140
Total		39324364	2643448	100.000

Detector A 254nm

Peak#	Ret. Time	Area	Height	Area%
1	9.188	4538443	423662	9.599
2	9.611	42741710	2319453	90.401
Total		47280154	2743115	100.000

(1*S*,2*R*,4*R*)-2-((*E*)-4-chlorostyryl)-1,2,3,4-tetrahydro-1,4-epoxynaphthalene (3af):

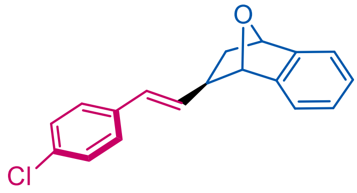

compound **3af** was prepared following general procedure C and the desired product was isolated through column chromatography (ethyl acetate/hexane = 1:19) in 93% (26.2 mg) yield with an *er* of 89:11 as sticky liquid. $[\alpha]_D{}^{20}$ = +183.66 (c = 0.160, $CHCl_3$).

^{1}H NMR ($CDCl_3$, 400 MHz): δ 7.29 (d, J = 3.7 Hz, 4H), 7.26 (d, J = 5.5 Hz, 2H), 7.19 (dd, J = 5.5, 2.4 Hz, 2H), 6.41 (d, J = 15.9 Hz, 1H), 6.32 (dd, J = 15.9, 9.2 Hz, 1H), 5.49 (d, J = 4.9 Hz, 1H), 5.18 (s, 1H), 2.54 (td, J = 8.2, 4.0 Hz, 1H), 1.91 (dt, J = 8.5, 4.6 Hz, 1H), 1.83 (d, J = 7.9 Hz, 1H).

^{13}C{^{1}H} NMR (100 MHz, $CDCl_3$): δ 145.91, 144.95, 135.75, 134.03, 132.67, 128.64, 127.25, 126.98, 126.75, 126.64, 119.01, 118.91, 84.12, 79.42, 44.03, 35.47.

HRMS (ESI): [M+H$^+$] calculated for $C_{18}H_{16}ClO^+$ is 283.0884; found 283.0900.

HPLC Condition: The enantiomeric excess was determined by Diacel Chiralpak IA, Hexane/IPA = 99/1, flow rate = 1.00 mL/min, λ = 254 nm, t(minor) = 13.696 min, t(major) = 10.538 min.

Chromatogram of (rac)-3af

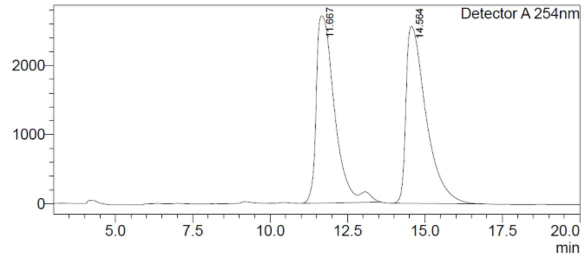

Peak#	Ret. Time	Area	Height	Area%
1	11.667	112284706	2709730	49.219
2	14.564	115848944	2562814	50.781
Total		228133651	5272545	100.000

Chromatogram of (1*S*,2*R*,4*R*)-3af

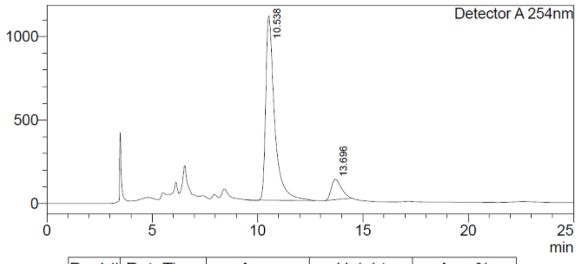

Peak#	Ret. Time	Area	Height	Area%
1	10.538	32229686	1105060	88.721
2	13.696	4097302	122046	11.279
Total		36326989	1227106	100.000

(1*S*,2*R*,4*R*)-2-((*E*)-4-methoxystyryl)-1,2,3,4-tetrahydro-1,4-epoxynaphthalene (3ag):

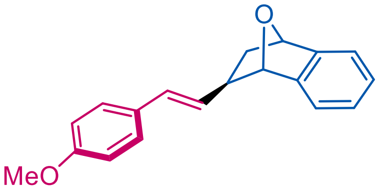

compound **3ag** was prepared following general procedure C and the desired product was isolated through column chromatography (ethyl acetate/hexane = 1:10) in 85% (23.6 mg) yield with an er of 88:12 as white solid. $[\alpha]_D{}^{20}$ = +133.88 (c = 0.117, $CHCl_3$).

^{1}H NMR ($CDCl_3$, 400 MHz): δ 7.32 (d, J = 8.6 Hz, 2H), 7.30 (d, J = 3.6 Hz, 1H), 7.26 (d, J = 8.6 Hz, 1H), 7.21 – 7.15 (m, 2H), 6.86 (d, J = 8.6 Hz, 2H), 6.40 (d, J = 15.8 Hz, 1H), 6.21 (dd, J = 15.8, 9.4 Hz,

1H), 5.48 (d, J = 4.7 Hz, 1H), 5.17 (s, 1H), 3.82 (s, 3H), 2.52 (td, J = 8.6, 3.8 Hz, 1H), 1.91 (dt, J = 11.9, 4.4 Hz, 1H), 1.81 (dd, J = 11.8, 8.1 Hz, 1H).

^{13}C{^{1}H} NMR (100 MHz, $CDCl_3$): δ 158.87, 146.01, 145.21, 131.15, 130.07, 129.21, 127.19, 126.66, 126.58, 119.01, 118.89, 113.94, 84.38, 79.44, 55.26, 44.08, 35.58.

HRMS (ESI): [M+H^{+}] calculated for $C_{19}H_{19}O2^{+}$ is 279.1380; found 279.1383.

HPLC Condition: The enantiomeric excess was determined by Diacel Chiralpak IB, Hexane/IPA = 99/1, flow rate = 1.00 mL/min, λ = 254 nm, t(minor) = 9.611 min, t(major) 10.716.

Chromatogram of (rac)-3ag

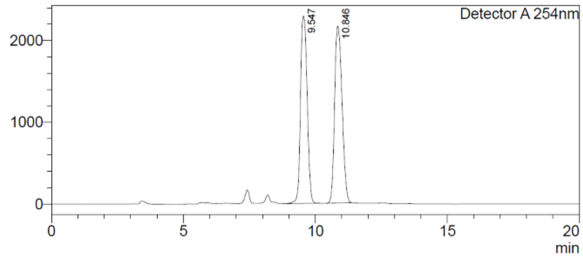

Peak#	Ret. Time	Area	Height	Area%
1	9.547	40167482	2294107	48.620
2	10.846	42448404	2165706	51.380
Total		82615885	4459813	100.000

Chromatogram of (1*S*,2*R*,4*R*)-3ag

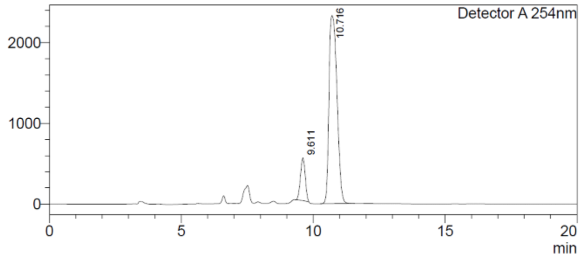

Peak#	Ret. Time	Area	Height	Area%
1	9.611	6654575	531228	11.937
2	10.716	49091497	2332866	88.063
Total		55746072	2864094	100.000

(1*S*,2*R*,4*R*)-2-((*E*)-4-(trifluoromethyl)styryl)-1,2,3,4-tetrahydro-1,4-epoxynaphthalene

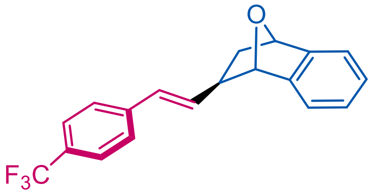

(3ah): compound **3ah** was prepared following general procedure C and the desired product was isolated through column chromatography (ethyl acetate/hexane = 1:19) in 79% (24.9 mg) yield with an er of 89:11 as sticky liquid. $[\alpha]_D^{20}$ = +97.34 (c = 0.190, $CHCl_3$). $[\alpha]_D^{20}$ = +193.01 (c = 0.124, $CHCl_3$).

^{1}H NMR ($CDCl_3$, 400 MHz): δ 7.55 (d, J = 8.2 Hz, 2H), 7.46 (d, J = 8.2 Hz, 2H), 7.31 – 7.26 (m, 2H), 7.18 (dd, J = 5.2, 3.0 Hz, 2H), 6.45 (m, 2H), 5.49 (d, J = 4.7 Hz, 1H), 5.19 (s, 1H), 2.56 (td, J = 7.7, 3.6 Hz, 1H), 1.94 – 1.89 (m, 1H), 1.82 (dd, J = 11.8, 8.1 Hz, 1H).

^{13}C{^{1}H} NMR (100 MHz, $CDCl_3$): δ 145.96, 144.91, 140.77, 136.19, 128.95 (q, J = 32.3 Hz), 128.66, 126.84, 126.71, 126.21, 125.49 (q, J = 3.8 Hz), 124.23 (q, J = 272.2 Hz), 119.07, 118.96, 84.06, 79.46, 44.12, 35.51.

^{19}F{^{1}H} NMR (373MHz, $CDCl_3$): δ -62.30.

HRMS (ESI): [M+H^{+}] calculated for $C_{19}H_{16}F_3O^{+}$ is 317.1148; found 317.1140.

HPLC Condition: The enantiomeric excess was determined by Diacel Chiralpak IA, Hexane/IPA = 99/1, flow rate = 1.00 mL/min, λ = 254 nm, t(minor) = 9.253 min, t(major) = 7.903 min.

Chromatogram of (rac)-3ah

Chromatogram of (1*S*,2*R*,4*R*)-3ah

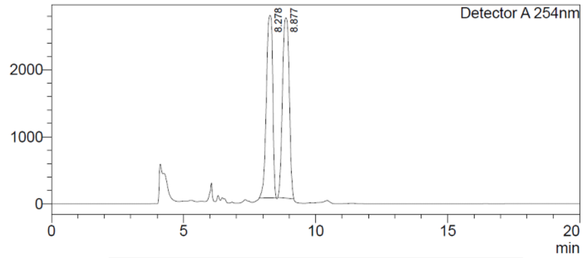

Peak#	Ret. Time	Area	Height	Area%
1	8.278	45284566	2724984	48.706
2	8.877	47690034	2692217	51.294
Total		92974601	5417201	100.000

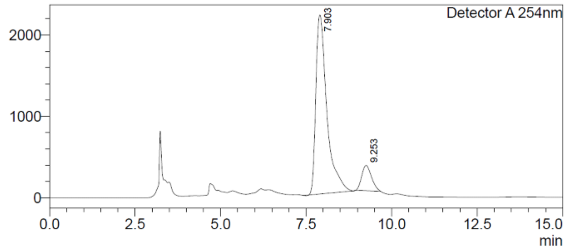

Peak#	Ret. Time	Area	Height	Area%
1	7.903	49279501	2199581	88.848
2	9.253	6185492	311742	11.152
Total		55464994	2511322	100.000

(1*S*,2*R*,4*R*)-2-((*E*)-2-(naphthalen-1-yl)vinyl)-1,2,3,4-tetrahydro-1,4-epoxynaphthalene

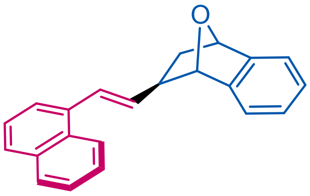

(3ai): compound **3ai** was prepared following general procedure C and the desired product was isolated through column chromatography (ethyl acetate/hexane = 1:19) in 83% (24.7 mg) yield with an er of 93:7 as white solid. $[\alpha]_D^{20}$ = +267.34 (c = 0.115, $CHCl_3$).

^{1}H NMR ($CDCl_3$, 400 MHz): δ 8.18 (d, J = 7.5 Hz, 1H), 7.91 – 7.87 (m, 1H), 7.81 (d, J = 8.2 Hz, 1H), 7.69 (d, J = 6.1 Hz, 1H), 7.58 – 7.51 (m, 2H), 7.49 (d, J = 7.0 Hz, 1H), 7.40 – 7.36 (m, 1H), 7.31 (d, J = 3.4 Hz, 1H), 7.28 – 7.22 (m, 3H), 6.44 (dd, J = 15.5, 9.5 Hz, 1H), 5.55 (d, J = 4.9 Hz, 1H), 5.31 (s, 1H), 2.76 – 2.69 (m, 1H), 2.07 – 2.01 (m, 1H), 1.92 (dd, J = 11.7, 8.1 Hz, 1H).

^{13}C{^{1}H} NMR (100 MHz, $CDCl_3$): δ 146.02, 145.16, 136.51, 134.87, 133.61, 131.02, 128.49, 127.56, 126.90, 126.70, 126.61, 125.84, 125.63, 123.70, 123.64, 119.05, 118.92, 84.28, 79.45, 44.42, 35.64.

HRMS (ESI): [M+H$^+$] calculated for $C_{22}H_{19}O^+$ is 299.1430; found 299.1431.

HPLC Condition: The enantiomeric excess was determined by Diacel Chiralpak IA, Hexane/IPA = 99/1, flow rate = 1.00 mL/min, λ = 254 nm, t(minor) = 11.877 min, t(major) = 9.676 min.

Chromatogram of (rac)-3ai

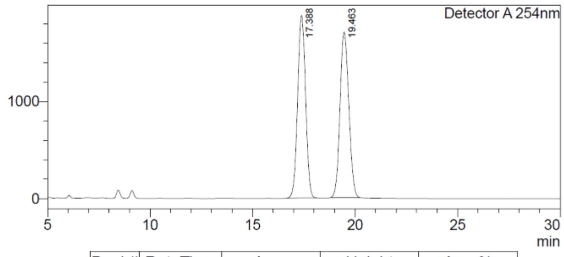

Peak#	Ret. Time	Area	Height	Area%
1	17.388	50601281	1879294	49.956
2	19.463	50691081	1704103	50.044
Total		101292363	3583397	100.000

Chromatogram of (1*S*,2*R*,4*R*)-3ai

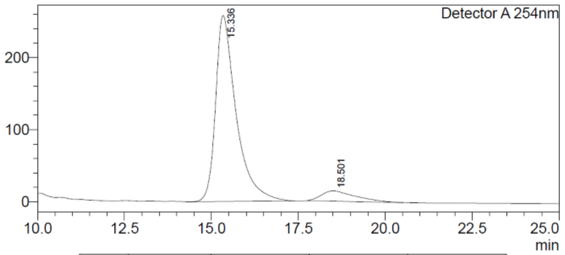

Peak#	Ret. Time	Area	Height	Area%
1	15.336	10631365	258329	91.594
2	18.501	975703	14385	8.406
Total		11607068	272714	100.000

(1*S*,2*R*,4*R*)-2-((*E*)-2-(8-phenylnaphthalen-1-yl)vinyl)-1,2,3,4-tetrahydro-1,4

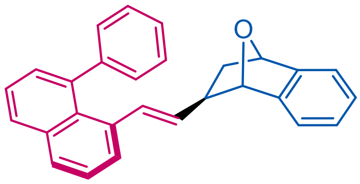

epoxynaphthalene (3aj): compound **3aj**was prepared following general procedure C and the desired product was isolated through column chromatography (ethyl acetate/hexane = 1:19) in 79% (29.5 mg) yield with an er of 98:2 as white solid. $[\alpha]_D^{20}$ = +247.47 (c = 0.210, $CHCl_3$).

¹H NMR ($CDCl_3$, 400 MHz): δ 8.18 (d, *J* = 7.5 Hz, 1H), 7.91 – 7.87 (m, 1H), 7.81 (d, *J* = 8.2 Hz, 1H), 7.69 (d, *J* = 6.1 Hz, 1H), 7.58 – 7.51 (m, 2H), 7.49 (d, *J* = 7.0 Hz, 1H), 7.40 – 7.36 (m, 1H), 7.31 (d, *J* = 3.4 Hz, 1H), 7.28 – 7.22 (m, 3H), 6.44 (dd, *J* = 15.5, 9.5 Hz, 1H), 5.55 (d, *J* = 4.9 Hz, 1H), 5.31 (s, 1H), 2.76 – 2.69 (m, 1H), 2.07 – 2.01 (m, 1H), 1.92 (dd, *J* = 11.7, 8.1 Hz, 1H).

¹³C{¹H} NMR (100 MHz, $CDCl_3$): δ 146.02, 145.16, 136.67, 136.51, 135.46, 135.21, 134.87, 133.61, 131.02, 128.49, 127.56, 126.90, 126.70, 126.61, 125.84, 125.63, 123.70, 123.64, 119.05, 118.92, 84.28, 79.45, 44.42, 35.64.

HRMS (ESI): [M+H⁺] calculated for $C_{28}H_{23}O^+$ is 375.1743; found 375.1745.

HPLC Condition: The enantiomeric excess was determined by Diacel Chiralpak IA, Hexane/IPA = 98/2, flow rate = 0.80 mL/min, λ = 254 nm, t(minor) = 18.335 min, t(major) = 22.465 min.

Chromatogram of (rac)-3aj

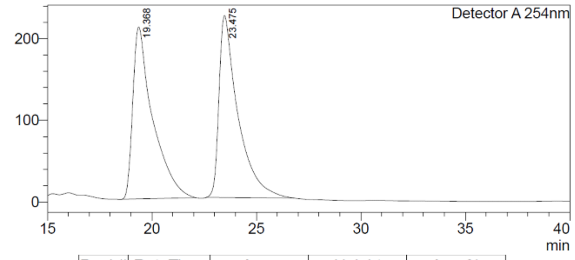

Peak#	Ret. Time	Area	Height	Area%
1	19.368	13981960	210828	49.801
2	23.475	14093784	223135	50.199
Total		28075744	433963	100.000

Chromatogram of (1*S*,2*R*,4*R*)-3aj

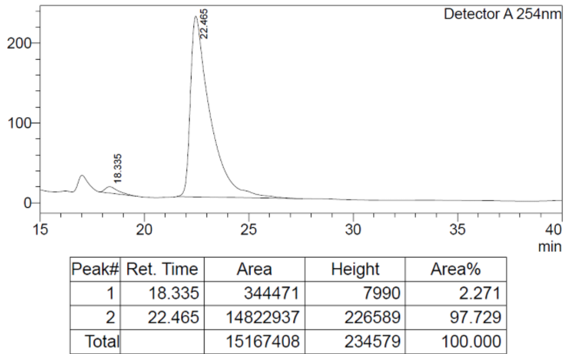

Peak#	Ret. Time	Area	Height	Area%
1	18.335	344471	7990	2.271
2	22.465	14822937	226589	97.729
Total		15167408	234579	100.000

4-((*E*)-2-((1*S*,2*R*,4*R*)-1,2,3,4-tetrahydro-1,4-epoxynaphthalen-2-yl)vinyl)benzonitrile

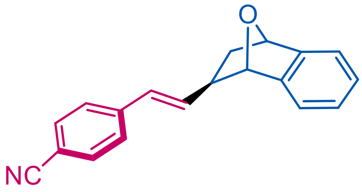

(3ak): compound **3ak** was prepared following general procedure C and the desired product was isolated through column chromatography (ethyl acetate/hexane = 5:95) in 91% (24.8 mg) yield with an er of 82.5:17.5 as white solid. $[\alpha]_D^{20}$ = +123.53 (c = 0.113, $CHCl_3$).

¹H NMR ($CDCl_3$, 400 MHz): δ 7.59 (d, *J* = 8.5 Hz, 2H), 7.45 (d, *J* = 8.5 Hz, 2H), 7.29 (dd, *J* = 8.2, 5.2 Hz, 2H), 7.19 (dd, *J* = 5.5, 3.1 Hz, 2H), 6.47 (d, *J* = 6.7 Hz, 2H), 5.50 (d, *J* = 4.9 Hz, 1H), 5.19 (s, 1H), 2.60 – 2.54 (m, 1H), 1.96 – 1.89 (m, 1H), 1.83 (dd, J = 11.6, 7.9 Hz, 1H).

¹³C{¹H} NMR (100 MHz, $CDCl_3$): δ 145.90, 144.76, 141.79, 137.63, 132.42, 128.47, 126.92, 126.76, 126.55, 119.09, 118.99, 110.35, 83.97, 79.46, 44.19, 35.51.

HRMS (ESI): [M+H⁺] calculated for $C_{19}H_{16}NO^+$ is 274.1226; found 274.1220.

HPLC Condition: The enantiomeric excess was determined by Diacel Chiralpak IA, Hexane/IPA = 98/2, flow rate = 1.00 mL/min, λ = 210 nm, t(minor) = 15.558 min, t(major) = 12.198 min.

Chromatogram of (rac)-3ak **Chromatogram of (1*S*,2*R*,4*R*)-3ak**

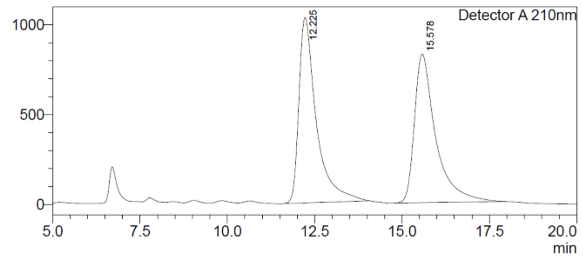

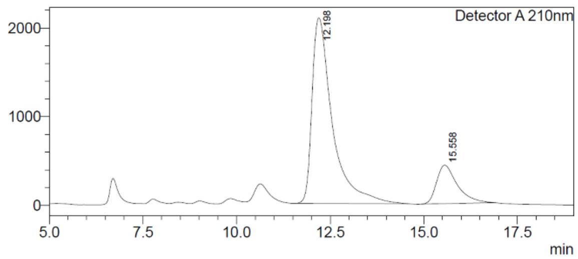

Peak#	Ret. Time	Area	Height	Area%
1	12.225	35891302	1032362	50.618
2	15.578	35014906	826741	49.382
Total		70906208	1859103	100.000

Peak#	Ret. Time	Area	Height	Area%
1	12.198	79053839	2093225	82.494
2	15.558	16775913	434799	17.506
Total		95829752	2528024	100.000

1-(3-((*E*)-2-((1*S*,2*R*,4*R*)-1,2,3,4-tetrahydro-1,4-epoxynaphthalen-2-yl)vinyl)phenyl)ethan-1-one (3al): compound **3al** was prepared following general procedure C and the desired product was isolated through column chromatography (ethyl acetate/hexane = 10:90) in 78% (22.6 mg) yield with an er of 84:16 as white solid. $[\alpha]_D^{20}$ = +285.35 (c = 0.197, $CHCl_3$).

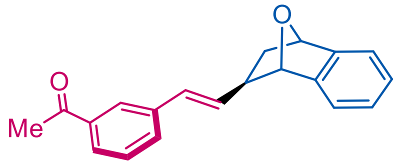

^{1}H NMR ($CDCl_3$, 400 MHz): δ 7.96 (t, J = 1.8 Hz, 1H), 7.81 (dt, J = 7.8, 1.6 Hz, 1H), 7.58 (dt, J = 7.8, 1.6 Hz, 1H), 7.41 (t, J = 7.6 Hz, 1H), 7.32 – 7.29 (m, 1H), 7.28 – 7.24 (m, 1H), 7.19 (dd, J = 5.5, 3.2 Hz, 2H), 6.50 (d, J = 15.6 Hz, 1H), 6.43 (dd, J = 16.0, 8.7 Hz, 1H), 5.50 (d, J = 5.0 Hz, 1H), 5.20 (s, 1H), 2.62 (s, 3H), 2.57 (td, J = 8.5, 4.1 Hz, 1H), 1.98 – 1.89 (m, 1H), 1.83 (dd, J = 11.9, 7.8 Hz, 1H).

^{13}C{^{1}H} NMR (100 MHz, $CDCl_3$): δ 198.14, 145.95, 144.96, 137.76, 137.41, 134.86, 130.53, 128.96, 128.78, 127.00, 126.78, 126.67, 125.85, 119.06, 118.92, 84.14, 79.45, 44.07, 35.50, 26.69.

HRMS (ESI): [M+H$^+$] calculated for $C_{20}H_{19}O_2^+$ is 291.1380; found 291.1381.

HPLC Condition: The enantiomeric excess was determined by Diacel Chiralpak IC, Hexane/IPA = 90/10, flow rate = 1.00 mL/min, λ = 220 nm, t(minor) = 29.049 min, t(major) = 30.285 min.

Chromatogram of (rac)-3al | **Chromatogram of (1*S*,2*R*,4*R*)-3al**

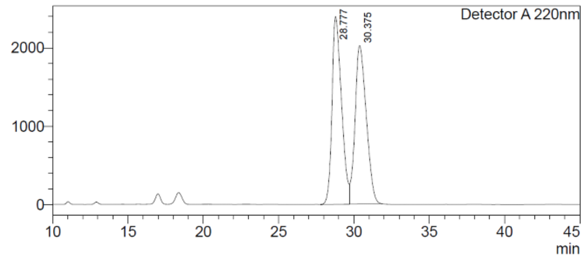

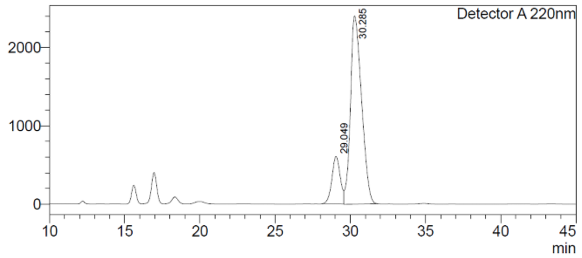

Peak#	Ret. Time	Area	Height	Area%
1	28.777	105124818	2396883	50.147
2	30.375	104509603	2019364	49.853
Total		209634421	4416247	100.000

Peak#	Ret. Time	Area	Height	Area%
1	29.049	23254394	606468	15.817
2	30.285	123765728	2403015	84.183
Total		147020122	3009483	100.000

***N*-(3-((*E*)-2-((1*R*,2*R*,4*R*)-1,2,3,4-tetrahydro-1,4-epoxynaphthalen-2-yl)vinyl)phenyl)acetamide (3am):** compound **3am** was prepared following general procedure C and the desired product was isolated through column chromatography (ethyl acetate/hexane = 20:80) in 89% (27.1 mg) yield with an er of 87:13 as sticky solid. $[\alpha]_D^{20}$ = +247.06 (c = 0.113, $CHCl_3$).

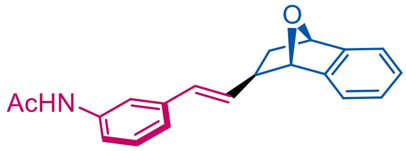

^{1}H NMR ($CDCl_3$, 400 MHz): δ 7.66 (s, 1H), 7.52 (s, 1H), 7.37 (d, J = 7.9 Hz, 1H), 7.30 – 7.27 (m, 1H), 7.27 – 7.23 (m, 2H), 7.18 (dd, J = 5.5, 3.1 Hz, 2H), 7.12 (d, J = 7.3 Hz, 1H), 6.39 (d, J = 15.9 Hz,

1H), 6.31 (dd, J = 15.9, 8.5 Hz, 1H), 5.47 (d, J = 4.9 Hz, 1H), 5.15 (s, 1H), 2.51 (td, J = 8.5, 3.7 Hz, 1H), 2.15 (s, 3H), 1.89 (dt, J = 11.6, 4.3 Hz, 1H), 1.79 (dd, J = 12.2, 7.9 Hz, 1H).

$^{13}C\{^1H\}$ NMR (100 MHz, $CDCl_3$): δ 168.56, 145.93, 145.02, 138.17, 133.91, 129.51, 129.09, 126.72, 126.63, 122.03, 119.03, 118.89, 118.71, 117.51, 84.17, 79.43, 43.98, 35.47, 24.51.

HRMS (ESI): [M+H$^+$] calculated for $C_{20}H_{20}NO^+$ is 306.1489; found 306.1493.

HPLC Condition: The enantiomeric excess was determined by Diacel Chiralpak IB, Hexane/IPA = 95/5, flow rate = 1.00 mL/min, λ = 254 nm, t(minor) = 78.226 min, t(major) = 85.920 min.

Chromatogram of (rac)-3am

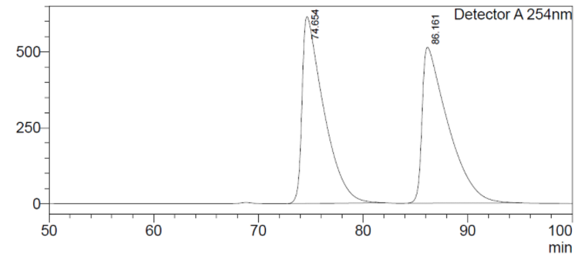

Peak#	Ret. Time	Area	Height	Area%
1	74.654	88530682	615726	50.035
2	86.161	88406770	513711	49.965
Total		176937452	1129437	100.000

Chromatogram of (1*R*,2*R*,4*R*)-3am

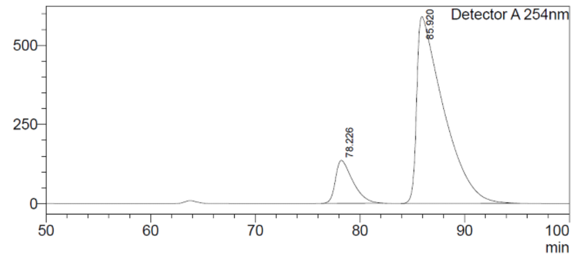

Peak#	Ret. Time	Area	Height	Area%
1	78.226	15624488	135687	12.922
2	85.920	105292934	590130	87.078
Total		120917421	725817	100.000

2-((*E*)-2-((1*S*,2*R*,4*R*)-1,2,3,4-tetrahydro-1,4-epoxynaphthalen-2-yl)vinyl)pyridine (3an):

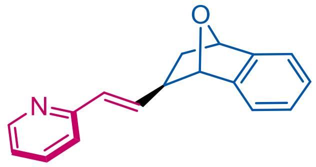

compound **3an** was prepared following general procedure C and the desired product was isolated through column chromatography (ethyl acetate/hexane = 25:75) in 73% (18.2 mg) yield with an er of 80:20 as sticky solid. $[\alpha]_D^{20}$ = +247.62 (c = 0.190, $CHCl_3$).

^1H NMR ($CDCl_3$, 400 MHz): δ 8.54 (dd, J = 5.0, 1.7 Hz, 1H), 7.64 (td, J = 7.8, 2.0 Hz, 1H), 7.35 (d, J = 7.9 Hz, 1H), 7.31 – 7.28 (m, 1H), 7.27 – 7.24 (m, 1H), 7.18 (dd, J = 5.5, 3.1 Hz, 2H), 7.13 (dd, J = 7.6, 4.6 Hz, 1H), 6.81 (dd, J = 15.8, 9.3 Hz, 1H), 6.58 (d, J = 16.0 Hz, 1H), 5.49 (d, J = 4.9 Hz, 1H), 5.22 (s, 1H), 2.60 (td, J = 8.6, 3.7 Hz, 1H), 1.97 (dt, J = 12.0, 4.4 Hz, 1H), 1.82 (dd, J = 11.9, 8.2 Hz, 1H).

$^{13}C\{^1H\}$ NMR (100 MHz, $CDCl_3$): δ 155.64, 149.24, 145.99, 145.00, 138.26, 136.61, 129.92, 126.78, 126.67, 121.86, 120.91, 119.09, 118.93, 83.96, 79.45, 43.87, 35.37.

HRMS (ESI): [M+H$^+$] calculated for $C_{17}H_{16}NO^+$ is 250.1226; found 250.1228.

HPLC Condition: The enantiomeric excess was determined by Diacel Chiralpak IC, Hexane/IPA = 80/20, flow rate = 1.00 mL/min, λ = 254 nm, t(minor) = 20.353 min, t(major) = 18.223 min.

Chromatogram of (rac)-3an

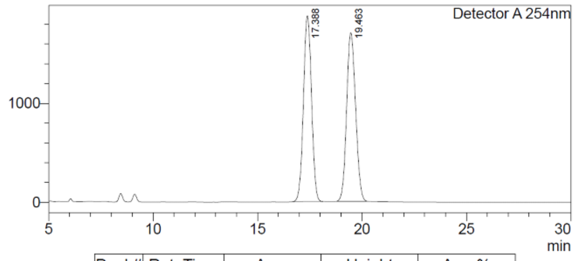

Peak#	Ret. Time	Area	Height	Area%
1	17.388	50601281	1879294	49.956
2	19.463	50691081	1704103	50.044
Total		101292363	3583397	100.000

Chromatogram of (1*S*,2*R*,4*R*)-3an

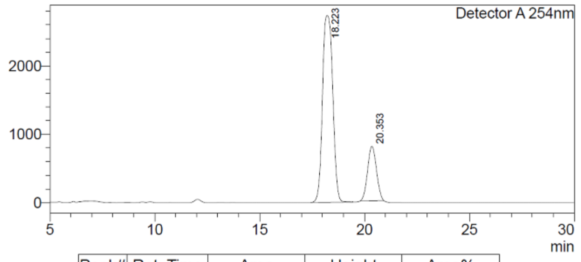

Peak#	Ret. Time	Area	Height	Area%
1	18.223	92492878	2730315	79.886
2	20.353	23288143	795084	20.114
Total		115781020	3525400	100.000

6-((*E*)-2-((1*S*,2*R*,4*R*)-1,2,3,4-tetrahydro-1,4-epoxynaphthalen-2-yl)vinyl)quinoline (3ao): compound **3ao** was prepared following general procedure C and the desired product was isolated through column chromatography (ethyl acetate/hexane = 30:70) in 70% (20.9 mg) yield with an er of 84:16 as sticky solid. $[\alpha]_D^{20}$ = +263.04 (c = 0.211, $CHCl_3$).

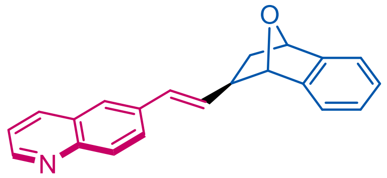

^{1}H NMR ($CDCl_3$, 400 MHz): δ 8.86 (d, *J* = 5.5 Hz, 1H), 8.15 – 8.05 (m, 2H), 7.86 (dd, *J* = 8.7, 2.3 Hz, 1H), 7.70 (d, *J* = 2.3 Hz, 1H), 7.40 (dd, *J* = 8.5, 4.4 Hz, 1H), 7.34 – 7.26 (m, 2H), 7.20 (dd, *J* = 5.3, 3.0 Hz, 2H), 6.63 (d, *J* = 16.0 Hz, 1H), 6.52 (dd, *J* = 16.0, 9.2 Hz, 1H), 5.51 (d, *J* = 4.6 Hz, 1H), 5.23 (s, 1H), 2.61 (td, *J* = 8.2, 3.7 Hz, 1H), 2.00 – 1.94 (m, 1H), 1.86 (dd, *J* = 11.9, 8.2 Hz, 1H).

^{13}C{^{1}H} NMR (100 MHz, $CDCl_3$): δ 149.74, 147.59, 145.98, 144.99, 136.12, 135.62, 135.25, 129.40, 129.19, 128.52, 127.48, 126.82, 126.70, 125.04, 121.42, 119.09, 118.97, 84.19, 79.49, 44.22, 35.59.

HRMS (ESI): [M+H$^+$] calculated for $C_{21}H_{18}NO^+$ is 300.1383; found 300.1381.

HPLC Condition: The enantiomeric excess was determined by Diacel Chiralpak IC, Hexane/IPA = 80/20, flow rate = 1.00 mL/min, λ = 254 nm, t(minor) = 32.723 min, t(major) = 28.001 min.

Chromatogram of (rac)-3ao

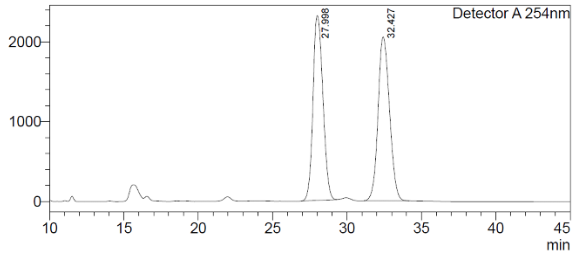

Peak#	Ret. Time	Area	Height	Area%
1	27.998	107309169	2314822	49.304
2	32.427	110337374	2051299	50.696
Total		217646543	4366121	100.000

Chromatogram of (1*S*,2*R*,4*R*)-3ao

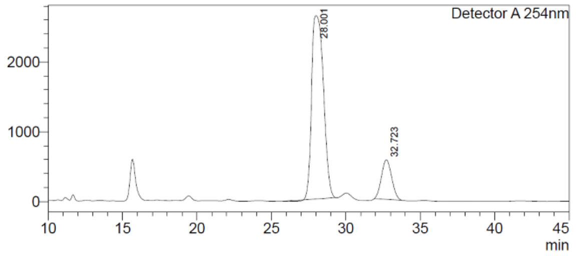

Peak#	Ret. Time	Area	Height	Area%
1	28.001	145007929	2626004	84.269
2	32.723	27070373	566952	15.731
Total		172078301	3192956	100.000

(1*S*,2*R*,4*R*)-2-((*E*)-2-(cyclohex-1-en-1-yl)vinyl)-1,2,3,4-tetrahydro-1,4-epoxynaphthalene (3ap): compound **3ap** was prepared following general procedure C and the desired product was isolated through column chromatography (ethyl acetate/hexane = 1:19) in 89% (22.4 mg) yield with an er of 94:6 as colourless liquid. $[\alpha]_D^{20}$ = +127.13 (c = 0.110, $CHCl_3$).

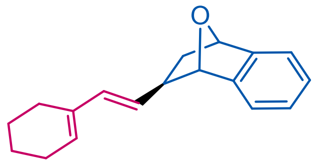

^{1}H NMR ($CDCl_3$, 400 MHz): δ 7.26 – 7.20 (m, 2H), 7.15 (dd, *J* = 5.8, 2.7 Hz, 2H), 6.09 (d, *J* = 15.3 Hz, 1H), 5.71 – 5.63 (m, 2H), 5.42 (d, *J* = 4.3 Hz, 1H), 5.08 (s, 1H), 2.39 (td, *J* = 8.5, 4.0 Hz, 1H), 2.20 – 2.08 (m, 4H), 1.85 – 1.74 (m, 2H), 1.71 – 1.65 (m, 2H), 1.64 – 1.57 (m, 2H).

^{13}C{^{1}H} NMR (100 MHz, $CDCl_3$): δ 145.97, 145.32, 135.42, 133.55, 129.21, 128.31, 118.90, 118.81, 84.56, 79.38, 43.92, 35.74, 25.78, 24.59, 22.55, 22.48.

HRMS (ESI): [M-H]$^+$ calculated for $C_{18}H_{19}O^+$ is 251.1430; found 251.1427.

HPLC Condition: The enantiomeric excess was determined by Diacel Chiralpak IA, Hexane/IPA = 99.5/0.5, flow rate = 0.80 mL/min, λ = 254 nm, t(minor) = 12.759 min, t(major) = 11.362 min.

Chromatogram of (rac)- 3ap **Chromatogram of (1*S*,2*R*,4*R*)-3ap**

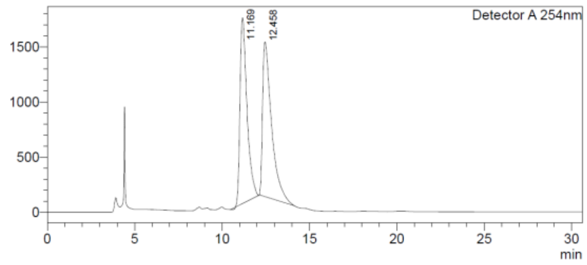

Detector A 254nm

Peak#	Ret. Time	Area	Height	Area%
1	11.169	47073251	1685309	48.373
2	12.458	50239430	1403779	51.627
Total		97312681	3089088	100.000

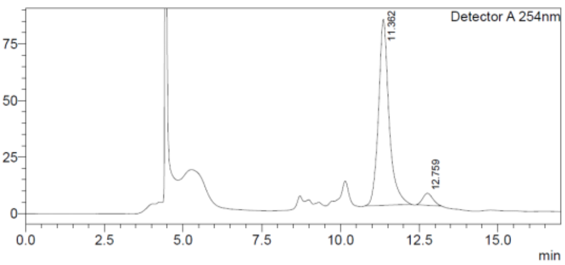

Detector A 254nm

Peak#	Ret. Time	Area	Height	Area%
1	11.362	1917011	82261	94.207
2	12.759	117890	5295	5.793
Total		2034901	87556	100.000

(1*S*,2*R*,4*R*)-2-((*E*)-2-cyclopentylvinyl)-1,2,3,4-tetrahydro-1,4-epoxynaphthalene (3aq):

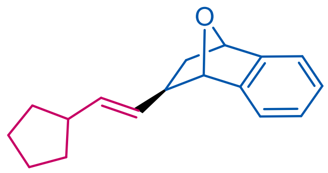

compound **3aq** was prepared following general procedure C and the desired product was isolated through column chromatography (ethyl acetate/hexane = 1:19) in 88% (21.1 mg) yield with an er of 97:3 as colourless liquid. $[\alpha]_D^{20}$ = +163.18 (c = 0.137, $CHCl_3$).

^{1}H NMR ($CDCl_3$, 400 MHz): δ 7.28 – 7.25 (m, 1H), 7.24 – 7.21 (m, 1H), 7.15 (dd, J = 5.4, 3.0 Hz, 2H), 5.59 – 5.44 (m, 2H), 5.41 (d, J = 4.8 Hz, 1H), 5.07 (s, 1H), 2.43 (q, J = 7.9 Hz, 1H), 2.32 (td, J = 7.9, 3.6 Hz, 1H), 1.84 – 1.72 (m, 4H), 1.70 – 1.54 (m, 4H), 1.38 – 1.22 (m, 2H).

^{13}C{^{1}H} NMR (100 MHz, $CDCl_3$): δ 146.04, 145.46, 135.30, 131.22, 126.46, 126.42, 118.90, 118.79, 84.56, 79.35, 43.39, 43.17, 35.53, 33.12, 25.07.

HRMS (ESI): $[M+H]^+$ calculated for $C_{17}H_{21}O^+$ is 241.1587; found 241.1587.

HPLC Condition: The enantiomeric excess was determined by Diacel Chiralpak IG, Hexane/IPA = 99/1, flow rate = 0.80 mL/min, λ = 210 nm, t(minor) = 14.665 min, t(major) = 16.316 min.

Chromatogram of (rac)- 3aq

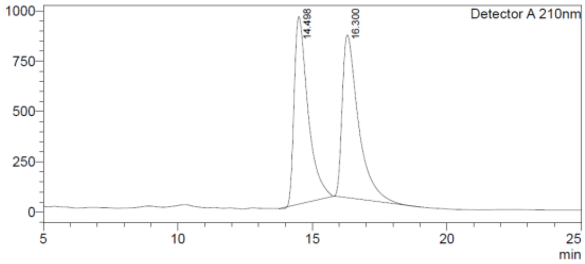

Detector A 210nm

Peak#	Ret. Time	Area	Height	Area%
1	14.498	33303060	930919	49.549
2	16.300	33909243	807998	50.451
Total		67212303	1738917	100.000

Chromatogram of (1*S*,2*R*,4*R*)-3aq

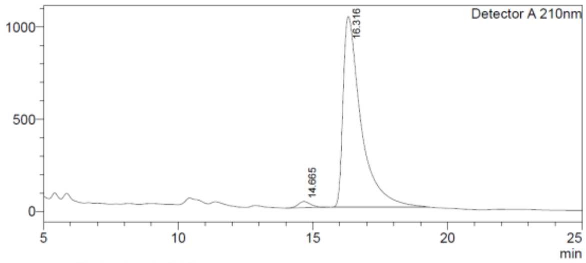

Detector A 210nm

Peak#	Ret. Time	Area	Height	Area%
1	14.665	1105327	33383	2.247
2	16.316	48083485	1035313	97.753
Total		49188811	1068696	100.000

(1*S*,2*R*,4*R*)-2-((*E*)-2-cyclopropylvinyl)-1,2,3,4-tetrahydro-1,4-epoxynaphthalene (3ar):

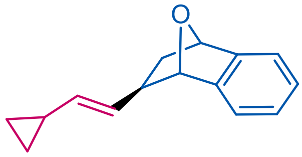

compound **3ar** was prepared following general procedure C and the desired product was isolated through column chromatography (ethyl acetate/hexane = 1:19) in 80% (17.0 mg) yield with an er of 93:7 as colourless liquid. $[\alpha]_D^{20}$ = +101.21 (c = 0.110, $CHCl_3$).

^{1}H NMR ($CDCl_3$, 400 MHz): δ 7.25 – 7.20 (m, 2H), 7.15 (dd, J = 5.4, 3.0 Hz, 2H), 5.65 (dd, J = 15.2, 9.2 Hz, 1H), 5.41 (d, J = 4.8 Hz, 1H), 5.09 – 5.01 (m, 2H), 2.31 (td, J = 8.6, 3.8 Hz, 1H), 1.79 (dt, J = 11.9, 4.3 Hz, 1H), 1.71 (dd, J = 11.8, 8.1 Hz, 1H), 1.46 – 1.35 (m, 1H), 0.70 (d, J = 8.2 Hz, 2H), 0.44 – 0.32 (m, 2H).

$^{13}C\{^1H\}$ **NMR** (100 MHz, $CDCl_3$): δ 146.01, 145.41, 134.13, 130.87, 126.50, 126.44, 118.89, 118.81, 84.47, 79.35, 43.38, 35.48, 13.50, 6.52.

HRMS (ESI): $[M+H]^+$ calculated for $C_{15}H_{17}O^+$ is 213.1274; found 213.1273.

HPLC Condition: The enantiomeric excess was determined by Diacel Chiralpak IG, Hexane/IPA = 99/1, flow rate = 0.80 mL/min, λ = 210 nm, t(minor) = 8.393 min, t(major) = 9.675 min.

Chromatogram of (rac)- 3ar

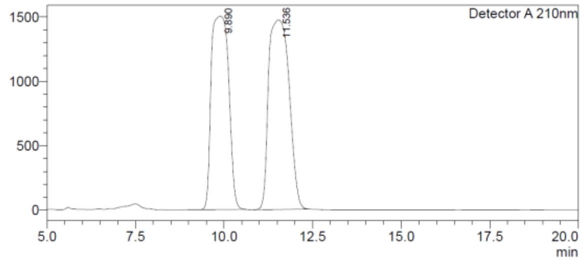

Detector A 210nm

Peak#	Ret. Time	Area	Height	Area%
1	9.890	51595632	1501139	46.444
2	11.536	59495416	1471707	53.556
Total		111091048	2972846	100.000

Chromatogram of (1*S*,2*R*,4*R*)-3ar

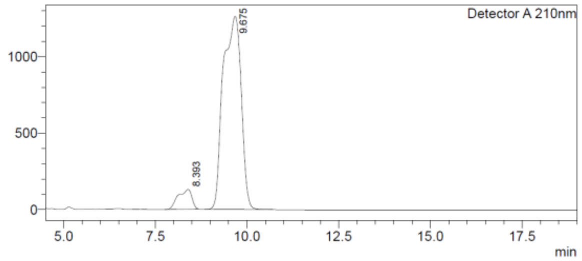

Detector A 210nm

Peak#	Ret. Time	Area	Height	Area%
1	8.393	3360206	129350	7.198
2	9.675	43321218	1263966	92.802
Total		46681424	1393315	100.000

(1*S*,2*R*,4*R*)-2-((*E*)-3,3-dimethylbut-1-en-1-yl)-1,2,3,4-tetrahydro-1,4-epoxynaphthalene

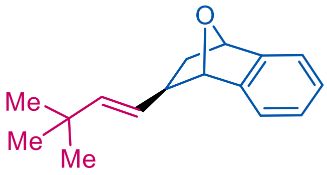

(3as): compound **3as** was prepared following general procedure C and the desired product was isolated through column chromatography (ethyl acetate/hexane = 1:19) in 77% (17.6 mg) yield with an er of 94:6 as colourless liquid. $[\alpha]_D^{20}$ = +133.87 (c = 0.107, $CHCl_3$).

^{1}**H NMR** ($CDCl_3$, 400 MHz): δ 7.30 – 7.25 (m, 1H), 7.23 (dd, *J* = 5.5, 3.1 Hz, 1H), 7.16 (dd, *J* = 5.5, 2.4 Hz, 2H), 5.56 – 5.45 (m, 2H), 5.42 (d, *J* = 4.3 Hz, 1H), 5.05 (s, 1H), 2.31 (q, *J* = 4.1 Hz, 1H), 1.82 – 1.69 (m, 2H), 1.04 (s, 9H).

$^{13}C\{^1H\}$ **NMR** (100 MHz, $CDCl_3$): δ 146.05, 145.46, 141.80, 128.14, 127.90, 126.46, 118.93, 118.80, 84.66, 79.36, 43.57, 35.68, 32.83, 29.74.

HRMS (ESI): $[M+H]^+$ calculated for $C_{15}H_{17}O^+$ is 229.1587; found 229.1583.

HPLC Condition: The enantiomeric excess was determined by Diacel Chiralpak IG, Hexane/IPA = 99/1, flow rate = 0.80 mL/min, λ = 254 nm, t(minor) = 13.934 min, t(major) = 16.185 min.

Chromatogram of (rac)- 3as

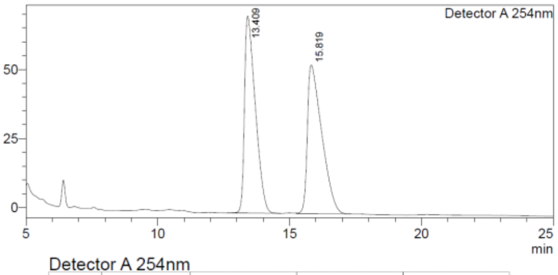

Detector A 254nm

Peak#	Ret. Time	Area	Height	Area%
1	13.409	2043417	71396	50.008
2	15.819	2042791	53859	49.992
Total		4086207	125255	100.000

Chromatogram of (1*S*,2*R*,4*R*)-3as

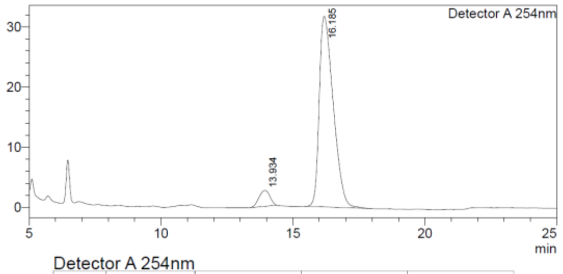

Detector A 254nm

Peak#	Ret. Time	Area	Height	Area%
1	13.934	75809	2653	6.082
2	16.185	1170585	31726	93.918
Total		1246394	34380	100.000

trimethyl((*E*)-2-((1*S*,2*R*,4*R*)-1,2,3,4-tetrahydro-1,4-epoxynaphthalen-2-yl)vinyl)silane

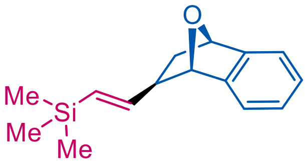

(3at): compound **3at** was prepared following general procedure C and the desired product was isolated through column chromatography (ethyl acetate/hexane = 1:19) in 90% (22 mg) yield with an er of 97:3 as colourless liquid. $[\alpha]_D^{20}$ = +104.54 (c = 0.115, $CHCl_3$).

^{1}H NMR ($CDCl_3$, 400 MHz): δ 7.25 (ddd, *J* = 11.8, 5.9, 3.2 Hz, 2H), 7.16 (dd, *J* = 5.4, 3.2 Hz, 2H), 6.14 (dd, *J* = 18.3, 8.4 Hz, 1H), 5.73 (d, *J* = 18.6 Hz, 1H), 5.44 (d, *J* = 4.5 Hz, 1H), 5.14 (s, 1H), 2.38 (td, *J* = 8.2, 3.6 Hz, 1H), 1.85 (dt, *J* = 11.8, 4.3 Hz, 1H), 1.74 (dd, *J* = 11.8, 8.2 Hz, 1H), 0.10 (s, 9H).

^{13}C{^{1}H} NMR (100 MHz, $CDCl_3$): δ 149.29, 146.04, 145.32, 130.18, 126.57, 126.52, 118.93, 118.85, 84.07, 79.36, 47.14, 35.07, -1.18.

HRMS (ESI): [M+H$^+$] calculated for $C_{15}H_{21}OSi^+$ is 245.1536; found 245.1527.

HPLC Condition: The enantiomeric excess was determined by Diacel Chiralpak IG, Hexane/IPA = 99/1, flow rate = 0.80 mL/min, λ = 210 nm, t(minor) = 12.107 min, t(major) = 13.503 min.

Chromatogram of (rac)- 3at

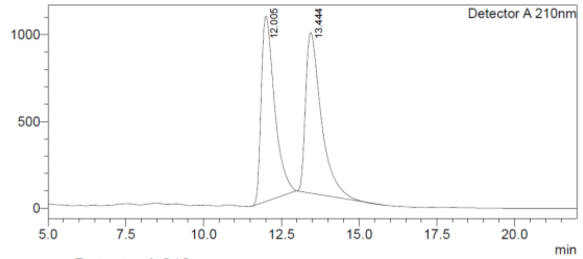

Detector A 210nm

Peak#	Ret. Time	Area	Height	Area%
1	12.005	32035237	1068394	49.684
2	13.444	32442500	926428	50.316
Total		64477736	1994823	100.000

Chromatogram of (1*S*,2*R*,4*R*)-3at

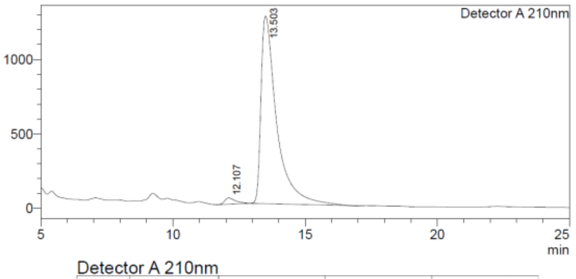

Detector A 210nm

Peak#	Ret. Time	Area	Height	Area%
1	12.107	1189031	41632	2.225
2	13.503	52248257	1259730	97.775
Total		53437287	1301362	100.000

(*E*)-3-((1*S*,2*R*,4*R*)-1,2,3,4-tetrahydro-1,4-epoxynaphthalen-2-yl)prop-2-en-1-ol (4aa):

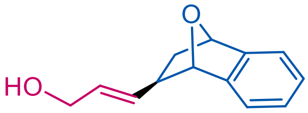

compound **4aa** was prepared following general procedure C and the desired product was isolated through column chromatography (ethyl acetate/hexane = 20:80) in 84% (17.0 mg) yield with an er of 78:22 as sticky liquid. $[\alpha]_D^{20}$ = +297.57 (c = 0.212, $CHCl_3$).

^{1}H NMR ($CDCl_3$, 400 MHz): δ 7.23 (ddd, *J* = 11.0, 5.5, 3.1 Hz, 2H), 7.14 (dd, *J* = 5.5, 2.4 Hz, 2H), 5.81 (dd, *J* = 15.6, 8.8 Hz, 1H), 5.70 (dt, *J* = 15.3, 5.8 Hz, 1H), 5.41 (d, *J* = 4.9 Hz, 1H), 5.08 (s, 1H), 4.12 (d, *J* = 5.7 Hz, 2H), 2.38 (td, *J* = 8.5, 4.0 Hz, 1H), 1.80 (dt, *J* = 11.6, 4.3 Hz, 1H), 1.72 (dd, *J* = 11.6, 7.9 Hz, 1H).

^{13}C{^{1}H} NMR (100 MHz, $CDCl_3$): δ 145.86, 145.03, 135.55, 129.18, 126.67, 126.58, 118.96, 118.86, 84.02, 79.36, 63.43, 43.06, 35.21.

HRMS (ESI): [M+Na$^+$] calculated for $C_{13}H_{14}NaO_2^+$ is 225.0886; found 225.0883.

HPLC Condition: The enantiomeric excess was determined by Diacel Chiralpak IA, Hexane/IPA = 95/5, flow rate = 1.00 mL/min, λ = 210 nm, t(minor) = 14.599 min, t(major) = 18.024 min.

Chromatogram of (rac)-4aa

Chromatogram of (1*S*,2*R*,4*R*)-4aa

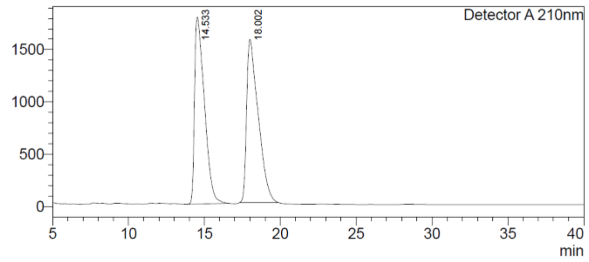

Peak#	Ret. Time	Area	Height	Area%
1	14.533	80720571	1788997	49.282
2	18.002	83073463	1562670	50.718
Total		163794035	3351668	100.000

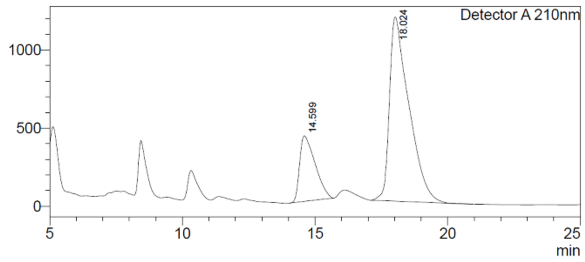

Peak#	Ret. Time	Area	Height	Area%
1	14.599	16982376	419742	21.992
2	18.024	60239017	1178500	78.008
Total		77221394	1598242	100.000

(*E*)-2-methyl-4-((1*S*,2*R*,4*R*)-1,2,3,4-tetrahydro-1,4-epoxynaphthalen-2-yl)but-3-en-2-ol

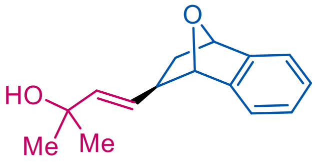

(4ab): compound **4ab** was prepared following general procedure C and the desired product was isolated through column chromatography (ethyl acetate/hexane = 20:80) in 89% (20.5 mg) yield with an *er* of 96:4 as sticky liquid. $[\alpha]_D{}^{20}$ = +147.98 (c = 0.171, $CHCl_3$).

^{1}H NMR ($CDCl_3$, 400 MHz): δ 7.26 – 7.23 (m, 1H), 7.21 (dd, *J* = 5.4, 3.2 Hz, 1H), 7.14 (dd, *J* = 5.4, 3.2 Hz, 2H), 5.74 (dd, *J* = 15.6, 8.4 Hz, 1H), 5.66 (d, *J* = 15.9 Hz, 1H), 5.40 (d, *J* = 5.0 Hz, 1H), 5.06 (s, 1H), 2.33 (td, *J* = 8.2, 3.6 Hz, 1H), 1.83 (brs, 1H), 1.81 – 1.75 (m, 1H), 1.71 (dd, *J* = 11.8, 8.2 Hz, 1H), 1.33 (s, 6H).

^{13}C{^{1}H} NMR (100 MHz, $CDCl_3$): δ 145.93, 145.14, 138.14, 130.11, 126.61, 126.51, 118.95, 118.82, 84.23, 79.35, 70.54, 43.09, 35.43, 29.70.

HRMS (ESI): [M-OH$^+$] calculated for $C_{15}H_{17}O^+$ is 213.1274; found 213.1277.

HPLC Condition: The enantiomeric excess was determined by Diacel Chiralpak IA, Hexane/IPA = 95/5, flow rate = 1.00 mL/min, λ = 220 nm, t(minor) = 15.277 min, t(major) = 17.416 min.

Chromatogram of (rac)-4ab

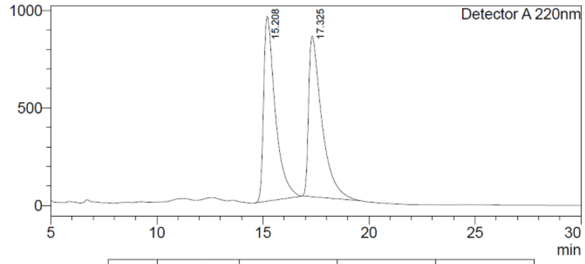

Peak#	Ret. Time	Area	Height	Area%
1	15.208	36245729	948152	50.332
2	17.325	35767122	824930	49.668
Total		72012851	1773082	100.000

Chromatogram of (1*S*,2*R*,4*R*)-4ab

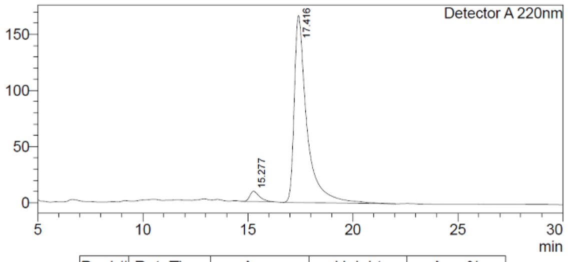

Peak#	Ret. Time	Area	Height	Area%
1	15.277	289066	9098	3.956
2	17.416	7018747	166729	96.044
Total		7307813	175828	100.000

(*E*)-3-((1*S*,2*R*,4*R*)-1,2,3,4-tetrahydro-1,4-epoxynaphthalen-2-yl)-1-(*m*-tolyl)prop-2-en-1-ol

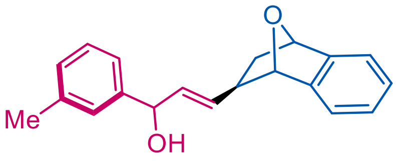

(4ac): compound **4ac** was prepared following general procedure C and the desired product was isolated through column chromatography (ethyl acetate/hexane = 25:75) in 87% (25.4 mg) yield with a dr of 1.2:1 and er of 94:6 as colourless liquid. $[\alpha]_D{}^{20}$ = +141.22 (c = 0.113, $CHCl_3$).

^{1}H NMR ($CDCl_3$, 400 MHz): δ 7.30 – 7.26 (m, 1H), 7.24 – 7.18 (m, 3H), 7.17 – 7.09 (m, 3H), 5.89 (ddd, *J* = 15.1, 9.1, 3.3 Hz, 1H), 5.75 (ddd, *J* = 15.7, 7.0, 4.2 Hz, 1H), 5.42 (t, *J* = 4.5 Hz, 1H), 5.18 (d, *J*

= 9.7 Hz, 1H), 5.10 (s, 1H), 2.38 (d, *J* = 4.8 Hz, 3H), 2.12 (s, 1H), 1.89 – 1.75 (m, 2H), 1.75 – 1.68 (m, 1H).

^{13}C{^{1}H} NMR (100 MHz, $CDCl_3$): δ 145.88, 145.03, 143.08, 138.24, 134.95, 134.74, 132.68, 132.54, 128.47, 128.39, 126.80, 126.75, 126.67, 126.58, 123.19, 123.15, 119.00, 118.86, 84.02, 79.36, 75.18, 75.00, 43.13, 43.08, 35.33, 35.27, 21.45.

HRMS (ESI): [M+H$^+$] calculated for $C_{20}H_{21}O_2^+$ is 292.1486; found 292.1483.

HPLC Condition: The enantiomeric excess was determined by Diacel Chiralpak IC, Hexane/IPA = 96/4, flow rate = 1.00 mL/min, λ = 254 nm, **major diastereomer**: t(minor) = 12.251 min, t(major) = 13.043 min; **minor diastereomer:** t(minor) = 35.116 min, t(major) = 19.001 min.

Chromatogram of (rac)-4ac **Chromatogram of (1*S*,2*R*,4*R*)-4ac**

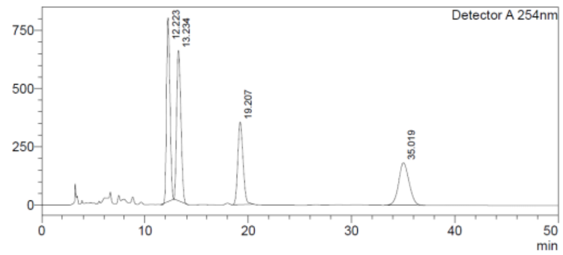

Detector A 254nm

Peak#	Ret. Time	Area	Height	Area%
1	12.223	18237626	788666	29.424
2	13.234	18444509	645117	29.758
3	19.207	12525391	354427	20.208
4	35.019	12774203	182158	20.610
Total		61981730	1970368	100.000

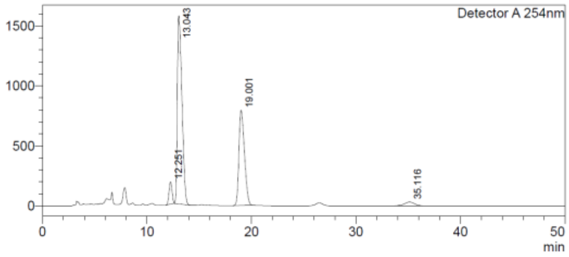

Detector A 254nm

Peak#	Ret. Time	Area	Height	Area%
1	12.251	3704213	185020	4.453
2	13.043	48114100	1568851	57.842
3	19.001	29288426	794017	35.210
4	35.116	2075188	30708	2.495
Total		83181928	2578596	100.000

Note: *The diastereomers are not separable through flash column chromatography.*

(*E*)-2-phenyl-4-((1*S*,2*R*,4*R*)-1,2,3,4-tetrahydro-1,4-epoxynaphthalen-2-yl)but-3-en-2-ol

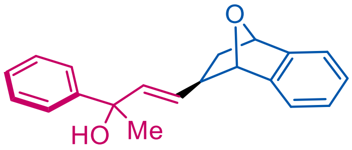

(4ad): compound **4ad** was prepared following general procedure C and the desired product was isolated through column chromatography (ethyl acetate/hexane = 25:75) in 86% (25.1 mg) yield with a dr of 1.3:1 and er of 95:5 as colourless liquid. $[\alpha]_D^{20}$ = +161.22 (c = 0.110, $CHCl_3$).

^{1}H NMR ($CDCl_3$, 400 MHz): δ 7.49 (dd, *J* = 7.9, 4.3 Hz, 2H), 7.36 (td, *J* = 7.9, 2.7 Hz, 2H), 7.29 – 7.21 (m, 3H), 7.16 (dd, *J* = 5.5, 3.1 Hz, 2H), 5.84 (d, *J* = 8.5 Hz, 2H), 5.42 (d, *J* = 4.9 Hz, 1H), 5.10 (s, 1H), 2.43 – 2.35 (m, 1H), 2.08 (s, 1H), 1.82 (dt, *J* = 7.9, 4.3 Hz, 1H), 1.74 (t, *J* = 11.6 Hz, 1H), 1.68 (s, 3H).

^{13}C{^{1}H} NMR (100 MHz, $CDCl_3$): δ 146.95, 145.92, 145.06, 137.20, 131.61, 131.54, 128.20, 126.87, 126.66, 126.56, 125.09, 119.01, 118.86, 84.17, 84.07, 79.36, 74.29, 43.17, 35.45, 35.39, 29.73.

HRMS (ESI): [M+H$^+$] calculated for $C_{20}H_{21}O_2^+$ is 293.1536; found 293.1533.

HPLC Condition: The enantiomeric excess was determined by Diacel Chiralpak IC, Hexane/IPA = 98/2, flow rate = 1.00 mL/min, λ = 254 nm, **major diastereomer**: t(minor) = 27.336 min, t(major) = 22.792 min; **minor diastereomer:** t(minor) = 68.660 min, t(major) = 41.931 min.

Chromatogram of (rac)-4ad **Chromatogram of (1*S*,2*R*,4*R*)-4ad**

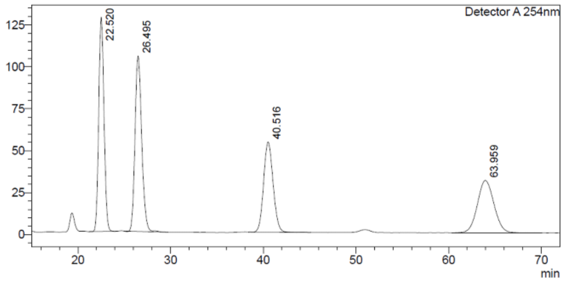

Peak#	Ret. Time	Area	Height	Area%
1	22.520	5052810	127712	28.041
2	26.495	5255882	104601	29.168
3	40.516	3834660	53781	21.281
4	63.959	3875999	31174	21.510
Total		18019350	317268	100.000

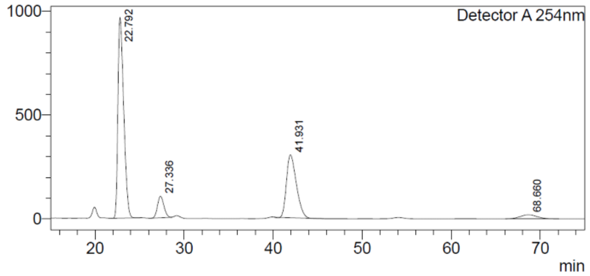

Peak#	Ret. Time	Area	Height	Area%
1	22.792	43718164	965871	58.350
2	27.336	5175427	103420	6.908
3	41.931	23385673	302439	31.212
4	68.660	2645106	19610	3.530
Total		74924370	1391339	100.000

Note: *The diastereomers are not separable through flash column chromatography.*

(*E*)-4-((1*S*,2*R*,4*R*)-1,2,3,4-tetrahydro-1,4-epoxynaphthalen-2-yl)-2-(*p*-tolyl)but-3-en-2-ol)

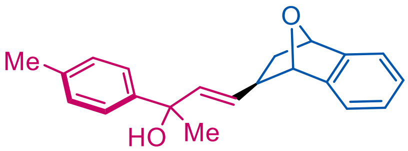

(4ae): compound **4ae** was prepared following general procedure C and the desired product was isolated through column chromatography (ethyl acetate/hexane = 25:75) in 82% (25.1 mg) yield with a dr of 1.2:1 and er of 97:3 as colourless liquid. $[\alpha]_D^{20}$ = +197.24 (c = 0.154, $CHCl_3$).

^{1}H NMR ($CDCl_3$, 400 MHz): δ 7.39 (dd, J = 7.9, 4.2 Hz, 2H), 7.28 – 7.23 (m, 2H), 7.18 (td, J = 6.3, 3.0 Hz, 4H), 5.83 (d, J = 9.1 Hz, 2H), 5.43 (d, J = 4.8 Hz, 1H), 5.11 (s, 1H), 2.40 (dt, J = 7.3, 3.6 Hz, 1H), 2.37 (s, 3H), 2.20 (s, 1H), 1.86 – 1.79 (m, 1H), 1.77 – 1.72 (m, 1H), 1.68 (s, 3H).

^{13}C{^{1}H} NMR (100 MHz, $CDCl_3$): δ 146.03, 145.21, 144.21, 144.17, 137.52, 136.55, 131.41, 131.34, 128.99, 126.75, 126.68, 125.18, 119.12, 118.97, 84.29, 84.19, 79.47, 74.26, 43.28, 35.54, 35.49, 29.82, 29.76, 21.08.

HRMS (ESI): [M+H$^+$] calculated for $C_{21}H_{23}O_2^+$ is 307.1693; found 307.1693.

HPLC Condition: The enantiomeric excess was determined by Diacel Chiralpak IA, Hexane/IPA = 96/4, flow rate = 1.00 mL/min, λ = 220 nm, **major diastereomer**: t(minor) = 24.346 min, t(major) = 38.735 min; **minor diastereomer:** t(minor) = 27.444 min, t(major) = 31.680 min.

Chromatogram of (rac)-4ae

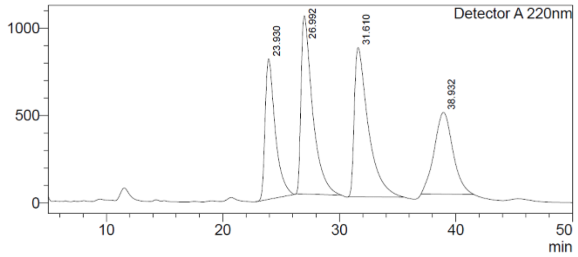

Peak#	Ret. Time	Area	Height	Area%
1	23.930	48480077	803386	20.029
2	26.992	70217423	1019675	29.010
3	31.610	71185546	853396	29.410
4	38.932	52162146	467805	21.551
Total		242045192	3144262	100.000

Chromatogram of (1*S*,2*R*,4*R*)-4ae

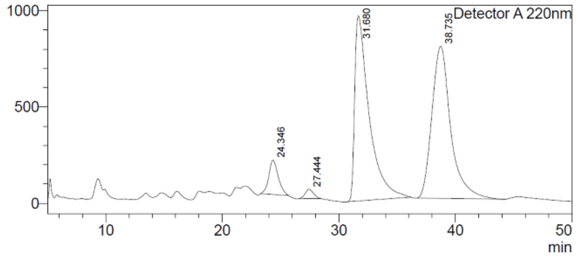

Peak#	Ret. Time	Area	Height	Area%
1	24.346	10042852	178247	5.462
2	27.444	2414609	48226	1.313
3	31.680	82924936	961709	45.096
4	38.735	88501096	789615	48.129
Total		183883493	1977797	100.000

Note: *The diastereomers are not separable through flash column chromatography.*

(*E*)-2-(naphthalen-2-yl)-4-((1*S*,2*R*,4*R*)-1,2,3,4-tetrahydro-1,4-epoxynaphthalen-2-yl)but-3-en -2-ol (4af): compound **4af** was prepared following general procedure C and the desired product was isolated through column chromatography (ethyl acetate/hexane = 25:75) in 84% (28.7 mg) yield with a dr of 1.3:1 and er of 97:3 as sticky liquid. $[\alpha]_D^{20}$ = +258.06 (c = 0.310, $CHCl_3$).

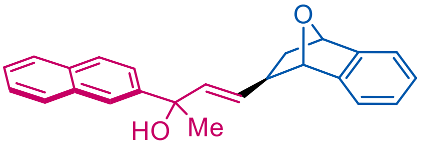

^{1}H NMR ($CDCl_3$, 400 MHz): δ 7.98 (d, J = 7.3 Hz, 1H), 7.86 (dd, J = 10.4, 7.3 Hz, 3H), 7.61 – 7.56 (m, 1H), 7.53 – 7.43 (m, 2H), 7.24 (dd, J = 7.6, 4.6 Hz, 2H), 7.16 (dd, J = 5.5, 3.1 Hz, 2H), 5.94 (d, J = 15.9 Hz, 1H), 5.87 (dd, J = 15.6, 8.2 Hz, 1H), 5.42 (d, J = 4.9 Hz, 1H), 5.12 (d, J = 3.7 Hz, 1H), 2.41 (td, J = 8.2, 4.0 Hz, 1H), 2.32 (s, 1H), 1.88 – 1.81 (m, 1H), 1.78 (s, 3H), 1.76 – 1.71 (m, 1H).

^{13}C{^{1}H} NMR (100 MHz, $CDCl_3$): δ 145.88, 145.03, 144.22, 137.13, 133.11, 132.34, 131.98, 131.92, 128.13, 127.86, 127.46, 126.66, 126.57, 126.05, 125.80, 124.13, 123.17, 119.00, 118.84, 84.16, 84.06, 79.35, 74.47, 43.17, 35.45, 35.38, 29.64.

HRMS (ESI): [M+H$^+$] calculated for $C_{24}H_{23}O_2^+$ is 343.1693; found 343.1691.

HPLC Condition: The enantiomeric excess was determined by Diacel Chiralpak IB, Hexane/IPA = 95/5, flow rate = 1.00 mL/min, λ = 254 nm, **major diastereomer**: t(minor) = 14.097 min, t(major) = 16.906 min; **minor diastereomer:** t(minor) = 12.257 min, t(major) = 24.238 min.

Chromatogram of (rac)-4af

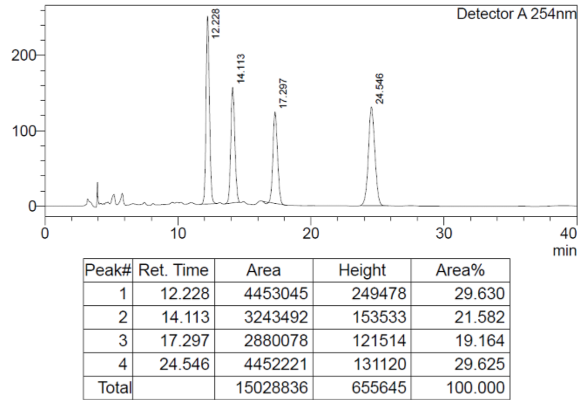

Peak#	Ret. Time	Area	Height	Area%
1	12.228	4453045	249478	29.630
2	14.113	3243492	153533	21.582
3	17.297	2880078	121514	19.164
4	24.546	4452221	131120	29.625
Total		15028836	655645	100.000

Chromatogram of (1*S*,2*R*,4*R*)-4af

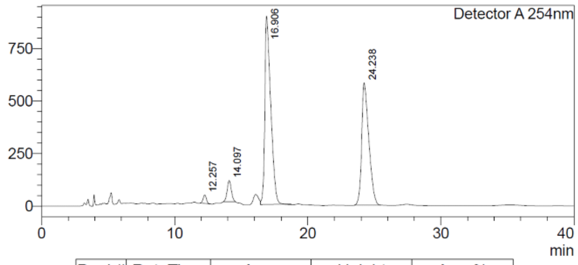

Peak#	Ret. Time	Area	Height	Area%
1	12.257	698497	41475	1.339
2	14.097	2237286	101682	4.289
3	16.906	27500122	895863	52.716
4	24.238	21730658	582678	41.656
Total		52166563	1621698	100.000

Note: *The diastereomers are not separable through flash column chromatography.*

1-((*E*)-2-((1*S*,2*R*,4*R*)-1,2,3,4-tetrahydro-1,4-epoxynaphthalen-2-yl)vinyl)-1,2,3,4-tetrahydronaphthalen-1-ol (4ag): compound **4ag** was prepared following general procedure C and the desired product was isolated through column chromatography (ethyl acetate/hexane = 20:80) in 81% (25.7 mg) yield with a dr of 1.1:1 and er of 92:8 as colourless liquid. $[\alpha]_D^{20}$ = +164.71 (c = 0.113, $CHCl_3$).

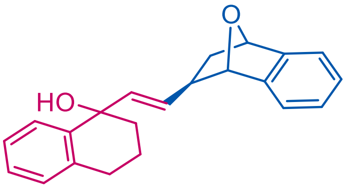

^{1}H NMR ($CDCl_3$, 400 MHz): δ 7.46 (d, J = 7.3 Hz, 1H), 7.24 – 7.19 (m, 3H), 7.15 (dd, J = 5.4, 3.0 Hz, 3H), 7.11 (dd, J = 6.6, 3.0 Hz, 2H), 5.77 – 5.70 (m, 1H), 5.40 (d, J = 4.8 Hz, 1H), 5.08 (s, 1H), 2.89 – 2.69 (m, 4H), 2.44 – 2.36 (m, 1H), 1.96 (q, J = 7.3 Hz, 4H), 1.87 – 1.76 (m, 4H).

^{13}C{^{1}H} NMR (100 MHz, $CDCl_3$): δ 145.98, 145.24, 140.38, 137.26, 137.17, 136.67, 135.62, 134.39, 132.58, 128.99, 128.16, 127.87, 127.59, 127.44, 126.90, 126.65, 126.59, 126.23, 125.84, 123.79, 119.00, 118.87, 84.34, 84.08, 79.43, 79.33, 72.99, 44.09, 43.20, 43.13, 38.14, 35.56, 35.37, 29.68, 28.23, 23.19, 19.55.

HRMS (ESI): [M+H$^+$] calculated for $C_{22}H_{23}O_2^+$ is 319.1963; found 319.1963.

HPLC Condition: The enantiomeric excess was determined by Diacel Chiralpak IC, Hexane/IPA = 96/4, flow rate = 1.00 mL/min, λ = 254 nm, **major diastereomer**: t(minor) = 40.040 min, t(major) = 24.628 min; **minor diastereomer:** t(minor) = 120.718 min, t(major) = 82.813 min.

Chromatogram of (rac)-4ag | **Chromatogram of (1*S*,2*R*,4*R*)-4ag**

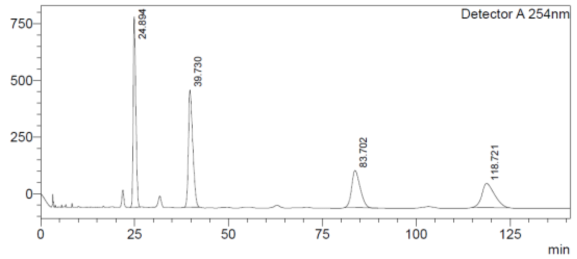

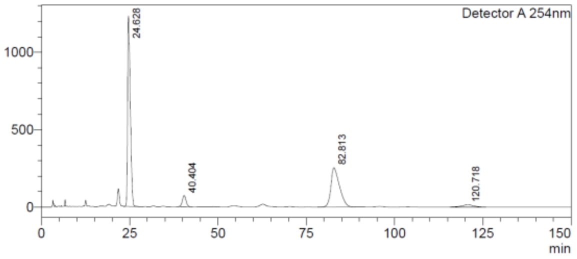

Detector A 254nm

Peak#	Ret. Time	Area	Height	Area%
1	24.894	40639464	840407	30.611
2	39.730	41496324	517272	31.257
3	83.702	25608447	163743	19.289
4	118.721	25014722	106797	18.842
Total		132758957	1628219	100.000

Detector A 254nm

Peak#	Ret. Time	Area	Height	Area%
1	24.628	65287641	1229940	56.308
2	40.404	5257643	72421	4.535
3	82.813	42306548	252768	36.488
4	120.718	3094879	13620	2.669
Total		115946711	1568749	100.000

Note: *The diastereomers are not separable through flash column chromatography.*

1-((*E*)-2-((1*S*,2*R*,4*R*)-1,2,3,4-tetrahydro-1,4-epoxynaphthalen-2-yl)vinyl)cyclohex-2-en-1-ol

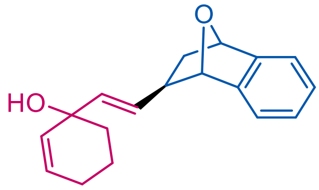

(4ah): compound **4ah** was prepared following general procedure C and the desired product was isolated through column chromatography (ethyl acetate/hexane = 20:80) in 83% (22.2 mg) yield with a dr of 1.1:1 and er of 94:6 as sticky liquid. $[\alpha]_D^{20}$ = +165.08 (c = 0.125, $CHCl_3$).

^{1}H NMR ($CDCl_3$, 400 MHz): δ 7.27 – 7.18 (m, 2H), 7.14 (dd, *J* = 5.4, 3.0 Hz, 2H), 5.84 (dt, *J* = 10.3, 3.6 Hz, 1H), 5.78 – 5.67 (m, 1H), 5.65 – 5.54 (m, 2H), 5.40 (d, *J* = 5.4 Hz, 1H), 5.07 (d, *J* = 10.3 Hz, 1H), 2.36 (td, *J* = 8.5, 4.2 Hz, 1H), 2.04 – 1.95 (m, 2H), 1.90 (s, 1H), 1.83 – 1.67 (m, 4H), 1.62 (dt, *J* = 11.5, 5.7 Hz, 1H).

^{13}C{^{1}H} NMR (100 MHz, $CDCl_3$): δ 146.05, 145.27, 136.41, 136.34, 132.69, 131.57, 130.09, 126.72, 126.65, 119.09, 118.96, 84.42, 84.24, 79.48, 79.44, 70.65, 43.37, 43.33, 36.74, 36.71, 35.62, 35.57, 35.51, 25.13, 19.28.

HRMS (ESI): [M+H$^+$] calculated for $C_{18}H_{21}O_2^+$ is 269.1536; found 269.1531.

HPLC Condition: The enantiomeric excess was determined by Diacel Chiralpak IC, Hexane/IPA = 98/2, flow rate = 1.00 mL/min, λ = 254 nm, **major diastereomer**: t(minor) = 96.205 min, t(major) = 103.937 min; **minor diastereomer:** t(minor) = 111.122 min, t(major) = 125.947 min.

Chromatogram of (rac)-4ah | **Chromatogram of (1*S*,2*R*,4*R*)-4ah**

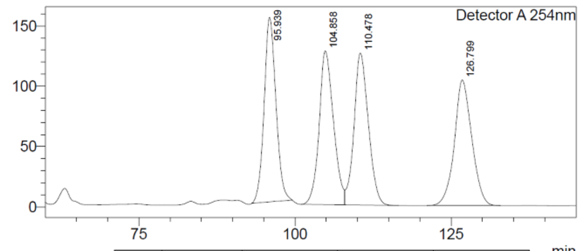

Peak#	Ret. Time	Area	Height	Area%
1	95.939	20115814	152947	24.876
2	104.858	20380539	127417	25.203
3	110.478	20127859	125918	24.890
4	126.799	20241467	104099	25.031
Total		80865679	510380	100.000

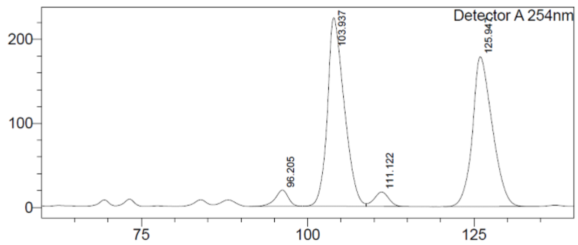

Peak#	Ret. Time	Area	Height	Area%
1	96.205	2612827	19218	3.190
2	103.937	39623789	224232	48.380
3	111.122	2594929	17206	3.168
4	125.947	37069231	178183	45.261
Total		81900776	438840	100.000

***Note:** The diastereomers are not separable through flash column chromatography.*

1-((*E*)-2-((1*S*,2*R*,4*R*)-1,2,3,4-tetrahydro-1,4-epoxynaphthalen-2-yl)vinyl)cyclohexan-1-ol

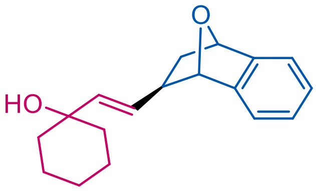

(4ai): compound **4ai** was prepared following general procedure C and the desired product was isolated through column chromatography (ethyl acetate/hexane = 30:70) in 78% (21.1 mg) yield with an er of 96:4 as colourless liquid. $[\alpha]_D^{20}$ = +189.06 (c = 0.117, $CHCl_3$).

^{1}H NMR ($CDCl_3$, 400 MHz): δ 7.27 (t, *J* = 4.2 Hz, 1H), 7.22 (dd, *J* = 5.4, 3.2 Hz, 1H), 7.15 (dd, *J* = 5.1, 3.0 Hz, 2H), 5.78 (dd, *J* = 15.8, 9.1 Hz, 1H), 5.64 (d, *J* = 15.5 Hz, 1H), 5.41 (d, *J* = 4.7 Hz, 1H), 5.07 (s, 1H), 2.35 (td, *J* = 8.5, 3.8 Hz, 1H), 1.79 (dt, *J* = 11.8, 4.1 Hz, 2H), 1.76 – 1.70 (m, 1H), 1.67 – 1.61 (m, 2H), 1.61 – 1.51 (m, 5H), 1.36 – 1.22 (m, 1H).

^{13}C{^{1}H} NMR (100 MHz, $CDCl_3$): δ 145.95, 145.18, 137.97, 130.91, 126.59, 126.51, 118.85, 118.82, 84.29, 79.35, 71.18, 43.27, 38.84, 37.90, 37.84, 25.52, 22.15.

HRMS (ESI): [M+H$^+$] calculated for $C_{18}H_{23}O_2^+$ is 271.1693; found 271.1691.

HPLC Condition: The enantiomeric excess was determined by Diacel Chiralpak IB, Hexane/IPA = 96/4, flow rate = 1.00 mL/min, λ = 254 nm, t(minor) = 8.024 min, t(major) = 11.800 min.

Chromatogram of (rac)-4ai

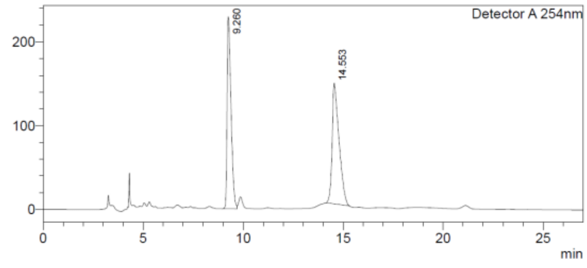

Detector A 254nm

Peak#	Ret. Time	Area	Height	Area%
1	9.260	3097215	229249	47.338
2	14.553	3445619	143985	52.662
Total		6542834	373234	100.000

Chromatogram of (1*S*,2*R*,4*R*)-4ai

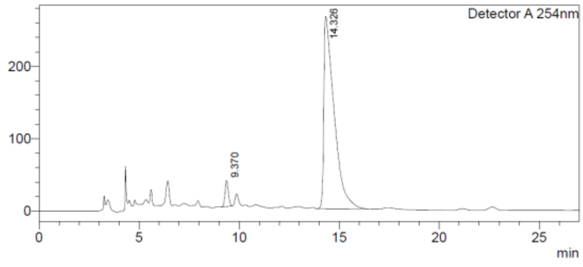

Detector A 254nm

Peak#	Ret. Time	Area	Height	Area%
1	9.370	413242	35978	4.171
2	14.326	9495012	266247	95.829
Total		9908254	302225	100.000

1-((*E*)-2-((1*S*,2*R*,4*R*)-1,2,3,4-tetrahydro-1,4-epoxynaphthalen-2-yl)vinyl)cyclododecan-1-ol

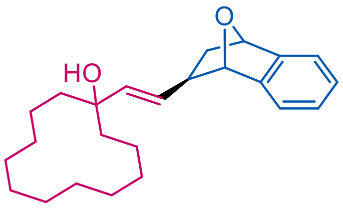

(4aj): compound **4aj** was prepared following general procedure C and the desired product was isolated through column chromatography (ethyl acetate/hexane = 25:75) in 82% (29.0 mg) yield with an er of 97:3 as sticky solid. $[\alpha]_D^{20}$ = +172.04 (c = 0.107, $CHCl_3$).

¹H NMR ($CDCl_3$, 400 MHz): δ 7.26 – 7.19 (m, 1H), 7.15 (dd, J = 5.4, 2.7 Hz, 4H), 5.74 (dd, J = 15.8, 8.7 Hz, 1H), 5.64 (d, J = 15.7 Hz, 1H), 5.41 (d, J = 4.8 Hz, 1H), 5.06 (s, 1H), 2.33 (dd, J = 8.5, 3.8 Hz, 1H), 1.87 (t, J = 7.5 Hz, 2H), 1.78 (d, J = 4.1 Hz, 1H), 1.75 – 1.65 (m, 2H), 1.41 – 1.31 (m, 20H).

¹³C{¹H} NMR (100 MHz, $CDCl_3$): δ 148.06, 145.97, 145.19, 137.50, 130.76, 126.61, 126.52, 118.97, 118.83, 84.40, 79.38, 74.97, 43.31, 35.56, 34.86, 26.42, 26.33, 26.05, 25.95, 22.59, 22.49, 22.21, 22.16, 19.92, 19.73, 19.68.

HRMS (ESI): [M+H⁺] calculated for $C_{24}H_{35}O_2^+$ is 355.2632; found 355.2635.

HPLC Condition: The enantiomeric excess was determined by Diacel Chiralpak IA, Hexane/IPA = 96/4, flow rate = 1.00 mL/min, λ = 220 nm, t(minor) = 30.558 min, t(major) = 27.134 min.

Chromatogram of (rac)-4aj

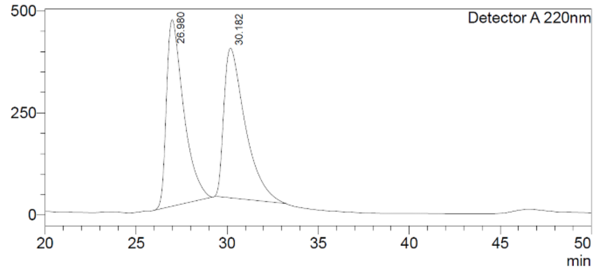

Peak#	Ret. Time	Area	Height	Area%
1	26.980	30136604	456866	50.146
2	30.182	29961511	366471	49.854
Total		60098115	823337	100.000

Chromatogram of (1*S*,2*R*,4*R*)-4aj

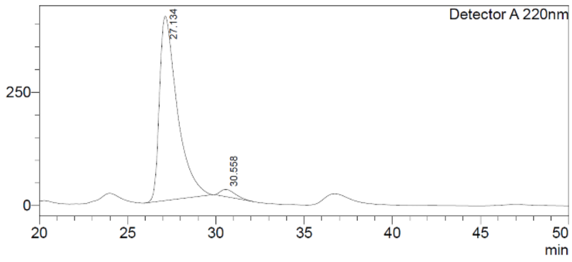

Peak#	Ret. Time	Area	Height	Area%
1	27.134	28634749	407020	96.775
2	30.558	954310	16033	3.225
Total		29589059	423053	100.000

(1*S*,2*R*,4*R*)-2-((*E*)-1-phenylprop-1-en-2-yl)-1,2,3,4-tetrahydro-1,4-epoxynaphthalene (3au):

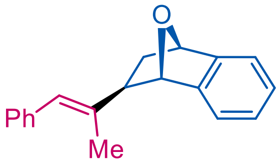

compound **3au** was prepared following general procedure D and the desired product was isolated through column chromatography (ethyl acetate/hexane = 5:95) in 72% (18.8 mg) yield with an er of 80:20 as sticky solid. $[\alpha]_D^{20}$ = +174.47 (c = 0.163, $CHCl_3$).

¹H NMR ($CDCl_3$, 400 MHz): δ 7.32 (t, J = 7.3 Hz, 2H), 7.25 (d, J = 8.5 Hz, 4H), 7.21 – 7.14 (m, 3H), 6.41 (s, 1H), 5.45 (d, J = 4.9 Hz, 1H), 5.30 (s, 1H), 2.48 (dd, J = 8.5, 4.9 Hz, 1H), 2.00 (dt, J = 12.2, 4.9 Hz, 1H), 1.94 (s, 3H), 1.78 (dd, J = 11.6, 8.5 Hz, 1H).

¹³C{¹H} NMR (100 MHz, $CDCl_3$): δ 146.24, 146.01, 140.20, 138.20, 128.95, 128.04, 126.06, 125.55, 119.10, 118.67, 83.06, 79.20, 49.49, 33.88, 15.91.

HRMS (ESI): [M+H⁺] calculated for $C_{19}H_{19}O^+$ is 263.1430; found 263.1431.

HPLC Condition: The enantiomeric excess was determined by Diacel Chiralpak IA, Hexane/IPA = 99/1, flow rate = 1.00 mL/min, λ = 254 nm, t(minor) = 8.124 min, t(major) = 12.336 min.

Chromatogram of (rac)-3au **Chromatogram of (1*S*,2*R*,4*R*)-3au**

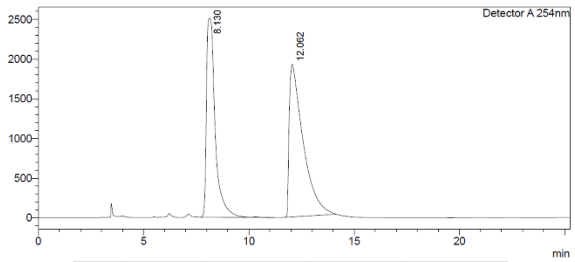

Peak#	Ret. Time	Area	Height	Area%
1	8.130	72175986	2509391	45.004
2	12.062	88199338	1923999	54.996
Total		160375324	4433390	100.000

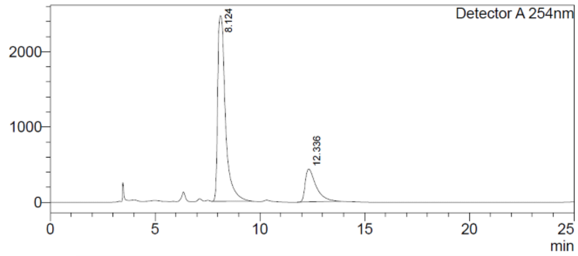

Peak#	Ret. Time	Area	Height	Area%
1	8.124	63227074	2466593	79.932
2	12.336	15874281	438208	20.068
Total		79101355	2904801	100.000

(1*S*,2*R*,4*R*)-2-((*E*)-1-phenylbut-1-en-2-yl)-1,2,3,4-tetrahydro-1,4-epoxynaphthalene (3av): compound **3av** was prepared following general procedure D and the desired product was isolated through column chromatography (ethyl acetate/hexane = 5:95) in 70% (19.3 mg) yield with an er of 76:24 as white solid. $[\alpha]_D^{20}$ = +133.88 (c = 0.203, $CHCl_3$).

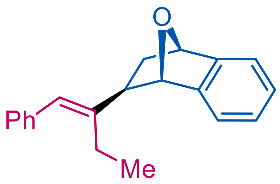

^{1}H NMR ($CDCl_3$, 400 MHz): δ 7.34 (t, *J* = 7.5 Hz, 2H), 7.29 (dd, *J* = 12.7, 6.6 Hz, 4H), 7.24 – 7.18 (m, 3H), 6.59 (s, 1H), 5.50 (d, *J* = 4.9 Hz, 1H), 5.36 (s, 1H), 2.43 (dt, *J* = 7.8, 3.9 Hz, 1H), 2.41 – 2.36 (m, 1H), 2.35 – 2.26 (m, 1H), 2.04 (dt, *J* = 11.6, 4.8 Hz, 1H), 1.89 (dd, *J* = 11.6, 8.6 Hz, 1H).

^{13}C{^{1}H} NMR (100 MHz, $CDCl_3$): δ 146.75, 146.32, 146.13, 138.37, 128.61, 128.07, 126.54, 126.51, 126.04, 124.39, 119.08, 118.68, 83.64, 79.09, 45.75, 36.20, 24.97, 13.42.

HRMS (ESI): [M+H$^+$] calculated for $C_{20}H_{21}O^+$ is 277.1587; found 277.1587.

HPLC Condition: The enantiomeric excess was determined by Diacel Chiralpak IA, Hexane/IPA = 99/1, flow rate = 1.00 mL/min, λ = 220 nm, t(minor) = 17.766 min, t(major) = 13.589 min.

Chromatogram of (rac)-3av

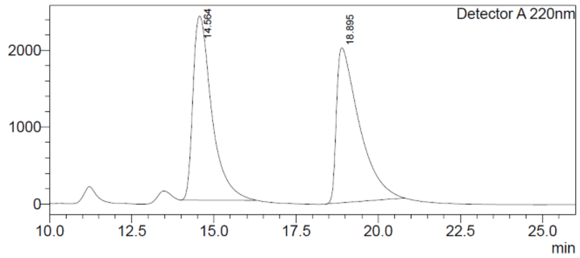

Peak#	Ret. Time	Area	Height	Area%
1	14.564	95555368	2396692	49.454
2	18.895	97665036	2017058	50.546
Total		193220404	4413749	100.000

Chromatogram of (1*S*,2*R*,4*R*)-3av

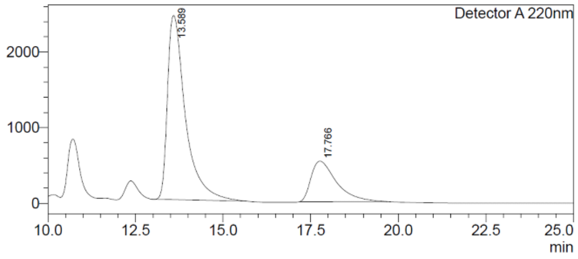

Peak#	Ret. Time	Area	Height	Area%
1	13.589	87012247	2435375	76.234
2	17.766	27125932	535678	23.766
Total		114138179	2971053	100.000

(1*S*,2*R*,4*R*)-2-((*E*)-5-chloro-1-phenylpent-1-en-2-yl)-1,2,3,4-tetrahydro-1,4-epoxynaphthalene (3aw): compound **3aw** was prepared following general procedure D and the desired product was isolated through column chromatography (ethyl acetate/hexane = 5:95) in 68% (22.0 mg) yield with an er of 75:25 as white solid. $[\alpha]_D^{20}$ = +174.19 (c = 0.158, $CHCl_3$).

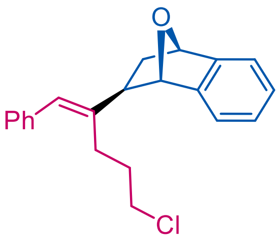

^{1}H NMR ($CDCl_3$, 400 MHz): δ 7.37 – 7.32 (m, 2H), 7.32 – 7.26 (m, 3H), 7.26 – 7.21 (m, 2H), 7.19 (dd, *J* = 5.1, 3.1 Hz, 2H), 6.66 (s, 1H), 5.50 (d, *J* = 4.9 Hz, 1H), 5.35 (s, 1H), 3.49 (t, *J* = 6.7 Hz, 2H), 2.53 – 2.39 (m, 3H), 2.03 (dt, *J* = 11.6, 4.7 Hz, 1H), 1.99 – 1.93 (m, 2H), 1.93 – 1.88 (m, 1H).

$^{13}C\{^1H\}$ **NMR** (100 MHz, $CDCl_3$): δ 146.09, 146.01, 143.66, 137.98, 128.57, 128.19, 126.65, 126.33, 126.03, 119.01, 118.72, 83.58, 79.10, 46.17, 44.87, 36.26, 31.64, 29.54.

HRMS (ESI): $[M+H^+]$ calculated for $C_{20}H_{20}ClO^+$ is 311.1197; found 311.1195.

HPLC Condition: The enantiomeric excess was determined by Diacel Chiralpak IB, Hexane/IPA = 98/2, flow rate = 0.50 mL/min, λ = 220 nm, t(minor) = 17.739min, t(major) = 41.227 min.

Chromatogram of (rac)-3aw

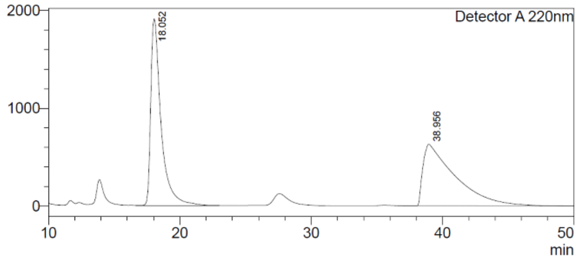

Peak#	Ret. Time	Area	Height	Area%
1	18.052	103551554	1908943	49.164
2	38.956	107072166	629524	50.836
Total		210623720	2538467	100.000

Chromatogram of (1S,2*R*,4*R*)-3aw

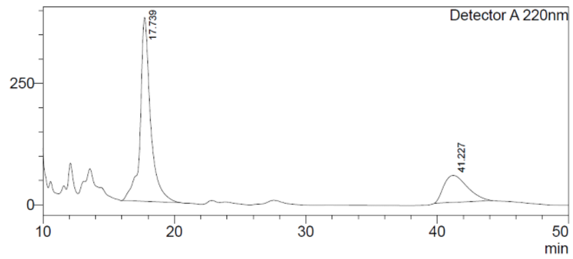

Peak#	Ret. Time	Area	Height	Area%
1	17.739	20773524	379276	74.963
2	41.227	6938084	55671	25.037
Total		27711609	434947	100.000

(1*S*,2*R*,4*R*)-2-((*Z*)-1,2-diphenylvinyl)-1,2,3,4-tetrahydro-1,4-epoxynaphthalene (3ax):

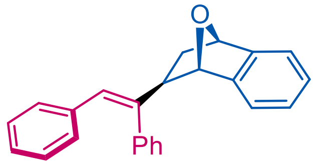

compound **1m** was prepared following general procedure D and the desired product was isolated through column chromatography (ethyl acetate/hexane = 5:95) in 66% (21.4 mg) yield with an er of 80:20 as white solid. $[\alpha]_D^{20}$ = +97.34 (c = 0.190, $CHCl_3$).

^1H **NMR** ($CDCl_3$, 400 MHz): δ 8.18 (d, *J* = 7.5 Hz, 1H), 7.91 – 7.87 (m, 1H), 7.81 (d, *J* = 8.2 Hz, 1H), 7.69 (d, *J* = 6.1 Hz, 1H), 7.58 – 7.51 (m, 2H), 7.49 (d, *J* = 7.0 Hz, 1H), 7.40 – 7.36 (m, 1H), 7.31 (d, *J* = 3.4 Hz, 1H), 7.28 – 7.22 (m, 3H), 6.44 (dd, *J* = 15.5, 9.5 Hz, 1H), 5.55 (d, *J* = 4.9 Hz, 1H), 5.31 (s, 1H), 2.76 – 2.69 (m, 1H), 2.07 – 2.01 (m, 1H), 1.92 (dd, *J* = 11.7, 8.1 Hz, 1H).

$^{13}C\{^1H\}$ **NMR** (100 MHz, $CDCl_3$): δ 146.02, 145.16, 136.51, 134.87, 133.61, 131.02, 128.49, 127.56, 126.90, 126.70, 126.61, 125.84, 125.63, 123.70, 123.64, 119.05, 118.92, 84.28, 79.45, 44.42, 35.64.

HRMS (ESI): $[M+H^+]$ calculated for $C_{24}H_{21}O^+$ is 325.1587; found 325.1589.

HPLC Condition: The enantiomeric excess was determined by Diacel Chiralpak IA, Hexane/IPA = 99/1, flow rate = 1.00 mL/min, λ = 254 nm, t(minor) = 9.872 min, t(major) = 23.157 min.

Chromatogram of (rac)-3ax

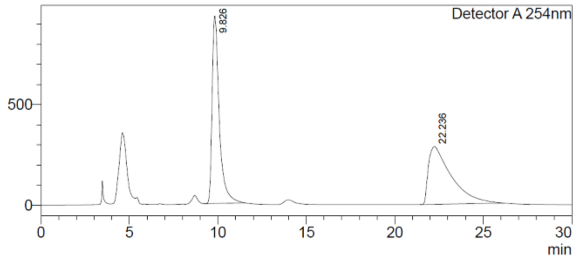

Peak#	Ret. Time	Area	Height	Area%
1	9.826	26167997	932408	50.205
2	22.236	25954653	287514	49.795
Total		52122650	1219923	100.000

Chromatogram of (1*S*,2*R*,4*R*)-3ax

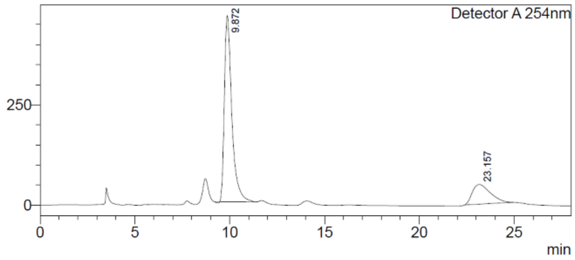

Peak#	Ret. Time	Area	Height	Area%
1	9.872	12710456	464540	79.669
2	23.157	3243629	48951	20.331
Total		15954086	513491	100.000

trimethyl((*Z*)-2-phenyl-1-((1*S*,2*S*,4*R*)-1,2,3,4-tetrahydro-1,4-epoxynaphthalen-2-yl)vinyl)silane (3ay): compound **3ay** was prepared following general procedure D and the desired product was isolated through column chromatography (ethyl acetate/hexane = 5:95) in 68% (21.8 mg) yield with an er of 80:20 as white solid. $[\alpha]_D^{20}$ = +117.40 (c = 0.236, $CHCl_3$).

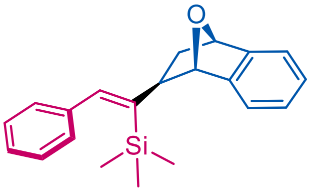

^{1}H NMR ($CDCl_3$, 400 MHz): δ δ 7.32 (t, *J* = 7.3 Hz, 2H), 7.25 (d, *J* = 8.5 Hz, 4H), 7.21 – 7.14 (m, 3H), 6.41 (s, 1H), 5.45 (d, *J* = 4.9 Hz, 1H), 5.30 (s, 1H), 2.48 (dd, *J* = 8.5, 4.9 Hz, 1H), 2.00 (dt, *J* = 12.2, 4.9 Hz, 1H), 1.78 (dd, *J* = 11.6, 8.5 Hz, 1H), 0.10 (s, 9H).

^{13}C{^{1}H} NMR (100 MHz, $CDCl_3$): δ 146.67, 146.48, 146.28, 140.70, 128.60, 127.68, 126.68, 126.50, 119.95, 119.08, 118.70, 111.80, 84.54, 79.33, 44.02, 37.39, 0.33.

HRMS (ESI): [M+H$^+$] calculated for $C_{21}H_{25}OSi^+$ is 321.1669; found 321.1667.

HPLC Condition: The enantiomeric excess was determined by Diacel Chiralpak IA, Hexane/IPA = 99/1, flow rate = 1.00 mL/min, λ = 254 nm, t(minor) = 12.334 min, t(major) = 8.124 min.

Chromatogram of (rac)-3ay

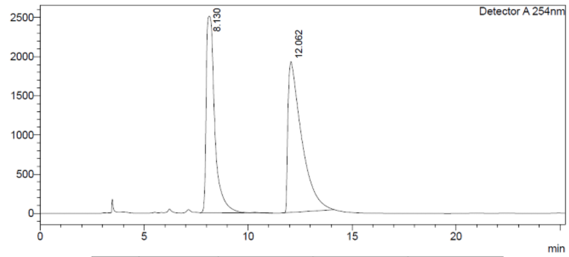

Peak#	Ret. Time	Area	Height	Area%
1	8.130	72175986	2509391	45.004
2	12.062	88199338	1923999	54.996
Total		160375324	4433390	100.000

Chromatogram of (1*S*,2*S*,4*R*)-3ay

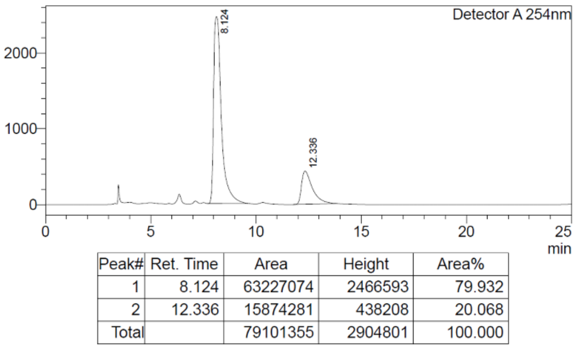

Peak#	Ret. Time	Area	Height	Area%
1	8.124	63227074	2466593	79.932
2	12.336	15874281	438208	20.068
Total		79101355	2904801	100.000

(1*S*,2*R*,4*R*)-2-((*Z*)-3-(benzyloxy)-1-phenylprop-1-en-2-yl)-1,2,3,4-tetrahydro-1,4-epoxynaphthalene (3az): compound **3az** was prepared following general procedure D and the desired product was isolated through column chromatography (ethyl acetate/hexane = 7:93) in 72% (26.5 mg) yield with an er of 72:28 as white solid. $[\alpha]_D^{20}$ = +189.20 (c = 0.134, $CHCl_3$).

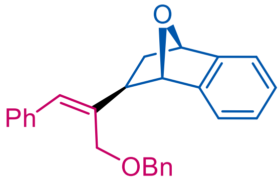

^{1}H NMR ($CDCl_3$, 400 MHz): δ 7.37 – 7.34 (m, 4H), 7.32 (d, *J* = 4.4 Hz, 4H), 7.29 – 7.24 (m, 4H), 7.20 – 7.17 (m, 2H), 6.78 (s, 1H), 5.48 (d, *J* = 4.9 Hz, 1H), 5.44 (s, 1H), 4.58 – 4.49 (m, 2H), 4.26 (d, *J* = 10.1 Hz, 1H), 4.13 (d, *J* = 10.1 Hz, 1H).

^{13}C{^{1}H} NMR (100 MHz, $CDCl_3$): δ 146.13, 140.71, 138.19, 137,09, 130.04, 128.82, 128.32, 128.08, 128.00, 127.64, 126.78, 126.51, 119.00, 118.88, 83.49, 79.09, 72.85, 68.22, 45.29, 35.44.

HRMS (ESI): [M+H$^+$] calculated for $C_{26}H_{24}O_2^+$ is 369.1849; found 369.1849.

HPLC Condition: The enantiomeric excess was determined by Diacel Chiralpak IA, Hexane/IPA = 98/2, flow rate = 1.00 mL/min, λ = 254 nm, t(minor) = 17.065 min, t(major) = 44.365 min.

Chromatogram of (rac)-3az **Chromatogram of (1*S*,2*R*,4*R*)-3az**

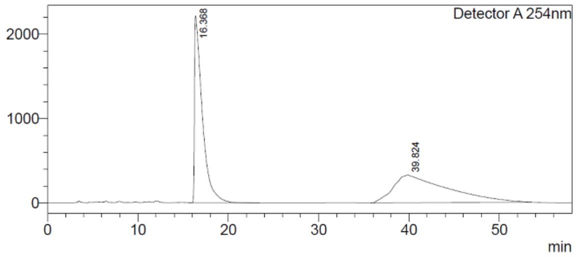

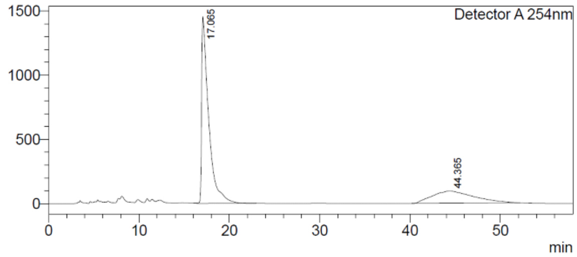

Peak#	Ret. Time	Area	Height	Area%
1	16.368	133522702	2217619	49.390
2	39.824	136820632	329060	50.610
Total		270343334	2546679	100.000

Peak#	Ret. Time	Area	Height	Area%
1	17.065	78627990	1455632	72.034
2	44.365	30526294	94999	27.966
Total		109154284	1550631	100.000

(1*S*,2*R*,4*R*)-6,7-dimethyl-2-((*E*)-2-(naphthalen-1-yl)vinyl)-1,2,3,4-tetrahydro-1,4-epoxynaphthalene (3bi): compound **3bi** was prepared following general procedure D and the desired product was isolated through column chromatography (ethyl acetate/hexane = 5:95) in 80% (26.1 mg) yield with an er of 94.5:5.5 as sticky solid. $[\alpha]_D^{20}$ = +109.40 (c = 0.189, $CHCl_3$).

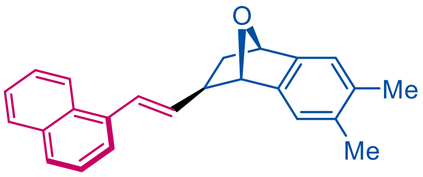

^{1}H NMR ($CDCl_3$, 400 MHz): δ 8.18 (d, J = 7.7 Hz, 1H), 7.91 – 7.86 (m, 1H), 7.80 (d, J = 8.6 Hz, 1H), 7.68 (d, J = 6.3 Hz, 1H), 7.59 – 7.47 (m, 3H), 7.23 (d, J = 15.9 Hz, 1H), 7.16 (s, 1H), 7.09 (s, 1H), 6.43 (dd, J = 15.4, 9.5 Hz, 1H), 5.48 (d, J = 4.7 Hz, 1H), 5.25 (s, 1H), 2.70 (td, J = 8.6, 3.7 Hz, 1H), 2.32 (s, 2H), 2.32 (s, 4H), 2.01 (dt, J = 11.8, 4.4 Hz, 1H), 1.89 (dd, J = 11.8, 8.0 Hz, 1H).

^{13}C{^{1}H} NMR (100 MHz, $CDCl_3$): δ 143.93, 143.08, 136.88, 134.95, 134.71, 134.61, 133.62, 131.04, 128.51, 127.52, 126.70, 125.84, 125.65, 123.74, 123.63, 120.43, 120.33, 84.25, 79.39, 44.91, 36.08, 19.96.

HRMS (ESI): [M+H$^+$] calculated for $C_{24}H_{23}O^+$ is 327.1743; found 327.1741.

HPLC Condition: The enantiomeric excess was determined by Diacel Chiralpak IA, Hexane/IPA = 99/1, flow rate = 0.80 mL/min, λ = 254 nm, t(minor) = 7.826 min, t(major) = 8.234 min.

Chromatogram of (rac)-3bi

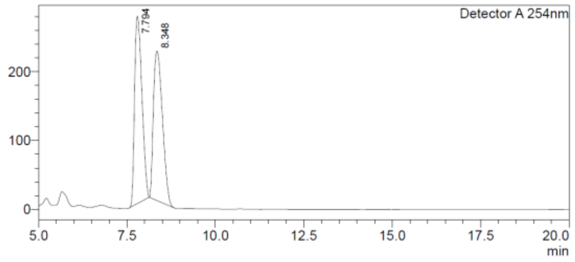

Detector A 254nm

Peak#	Ret. Time	Area	Height	Area%
1	7.794	3926737	271967	50.540
2	8.348	3842888	217217	49.460
Total		7769625	489184	100.000

Chromatogram of (1*R*,2*S*,4*R*)-3bi

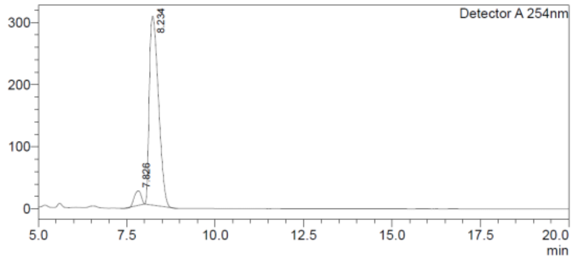

Detector A 254nm

Peak#	Ret. Time	Area	Height	Area%
1	7.826	316024	23464	5.448
2	8.234	5484403	304278	94.552
Total		5800427	327742	100.000

(1*S*,2*R*,4*R*)-6,7-dimethoxy-2-((*E*)-2-(naphthalen-1-yl)vinyl)-1,2,3,4-tetrahydro-1,4-epoxynaphthalene (3ci): compound **3ci** was prepared following general procedure D and the desired product was isolated through column chromatography (ethyl acetate/hexane = 10:90) in 78% (27.9 mg) yield with an er of 94:6 as sticky solid. $[\alpha]_D^{20}$ = +214.39 (c = 0.197, $CHCl_3$).

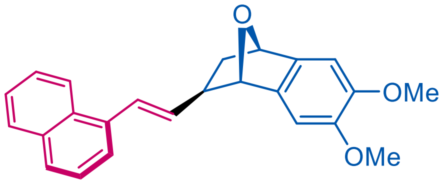

^{1}H NMR ($CDCl_3$, 400 MHz): δ 8.17 – 8.11 (m, 1H), 7.88 – 7.83 (m, 1H), 7.77 (d, J = 8.1 Hz, 1H), 7.64 (d, J = 7.0 Hz, 1H), 7.49 (dp, J = 11.6, 6.5 Hz, 3H), 7.21 (d, J = 15.5 Hz, 1H), 6.97 (s, 1H), 6.90 (s, 1H),

6.37 (dd, J = 15.4, 9.5 Hz, 1H), 5.47 (d, J = 4.8 Hz, 1H), 5.22 (s, 1H), 3.91 (s, 3H), 3.90 (s, 3H), 2.65 (dt, J = 9.1, 4.5 Hz, 1H), 1.96 (dt, J = 8.4, 4.3 Hz, 1H), 1.83 (dd, J = 11.8, 8.1 Hz, 1H).

$^{13}C\{^1H\}$ NMR (100 MHz, $CDCl_3$): δ 147.94, 147.87, 138.31, 137.42, 136.71, 134.88, 133.72, 133.60, 131.01, 128.51, 127.54, 126.81, 125.84, 125.65, 123.69, 123.61, 103.85, 103.78, 84.61, 84.55, 79.73, 56.29, 56.24, 44.95, 36.10.

HRMS (ESI): [M+H$^+$] calculated for $C_{24}H_{23}O_3^+$ is 359.1642; found 359.1642.

HPLC Condition: The enantiomeric excess was determined by Diacel Chiralpak IB, Hexane/IPA = 96/4, flow rate = 1.00 mL/min, λ = 254 nm, t(minor) = 10.239 min, t(major) = 11.829 min.

Chromatogram of (rac)-3ci

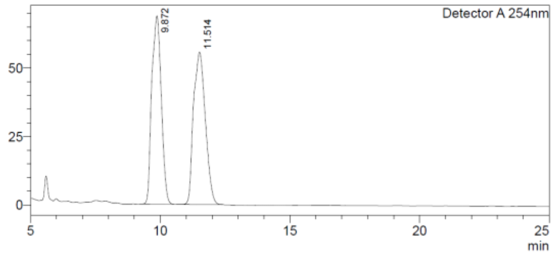

Detector A 254nm

Peak#	Ret. Time	Area	Height	Area%
1	9.872	1753916	68710	49.761
2	11.514	1770735	55712	50.239
Total		3524651	124422	100.000

Chromatogram of (1*S*,2*R*,4*R*)-3ci

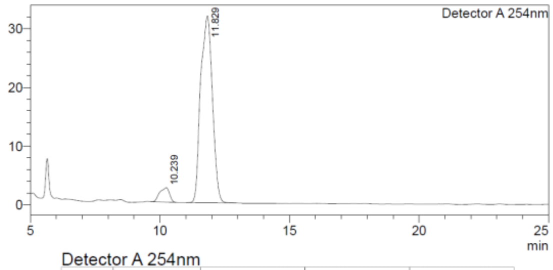

Detector A 254nm

Peak#	Ret. Time	Area	Height	Area%
1	10.239	63922	2444	5.971
2	11.829	1006656	31837	94.029
Total		1070578	34280	100.000

(5*S*,6*R*,8*R*)-6-((*E*)-2-(naphthalen-1-yl)vinyl)-5,6,7,8-tetrahydro-5,8-epoxynaphtho[2,3-*d*][1,3]dioxole (3di): compound **3di** was prepared following general procedure D and the desired product was isolated through column chromatography (ethyl acetate/hexane = 10:90) in 83% (28.4 mg) yield with an er of 96:4 as white solid. $[\alpha]_D^{20}$ = + 209.09 (c = 0.210, $CHCl_3$).

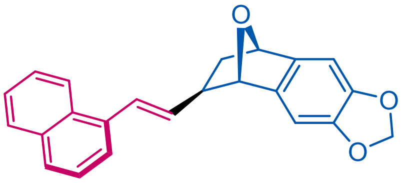

^{1}H NMR ($CDCl_3$, 400 MHz): δ 8.18 – 8.10 (m, 1H), 7.86 (dd, J = 7.7, 1.8 Hz, 1H), 7.78 (d, J = 8.3 Hz, 1H), 7.68 – 7.60 (m, 1H), 7.56 – 7.41 (m, 3H), 7.20 (d, J = 15.5 Hz, 1H), 6.86 (s, 1H), 6.80 (s, 1H), 6.36 (dd, J = 15.7, 9.5 Hz, 1H), 6.00 – 5.96 (m, 1H), 5.43 (d, J = 5.0 Hz, 1H), 5.18 (s, 1H), 2.64 (ddd, J = 9.5, 8.2, 3.6 Hz, 1H), 2.01 – 1.90 (m, 1H), 1.88 – 1.79 (m, 1H).

$^{13}C\{^1H\}$ NMR (100 MHz, $CDCl_3$): δ 146.27, 139.77, 138.83, 136.52, 134.87, 133.61, 131.02, 128.51, 127.57, 126.83, 125.59, 123.68, 101.38, 101.13, 84.30, 79.58, 44.75, 44.65, 35.90.

HRMS (ESI): [M+H$^+$] calculated for $C_{23}H_{19}O_3^+$ is 343.1329; found 343.1331.

HPLC Condition: The enantiomeric excess was determined by Diacel Chiralpak IB, Hexane/IPA = 97/3, flow rate = 1.00 mL/min, λ = 254 nm, t(minor) = 21.111 min, t(major) = 44.935 min.

Chromatogram of (rac)-3di **Chromatogram of (5*S*,6*R*,8*R*)-3di**

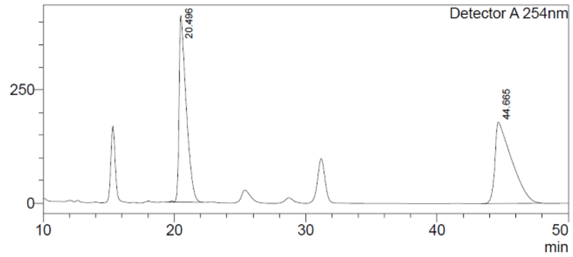

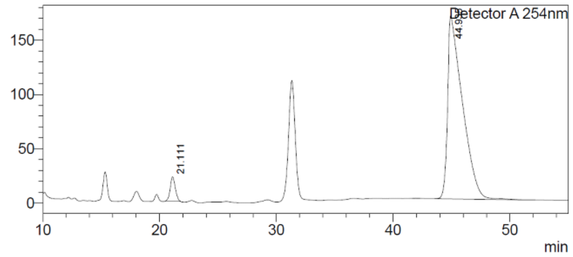

Peak#	Ret. Time	Area	Height	Area%
1	20.496	15603635	412193	49.356
2	44.665	16010811	179751	50.644
Total		31614446	591944	100.000

Peak#	Ret. Time	Area	Height	Area%
1	21.111	675242	22421	4.330
2	44.935	14919515	168748	95.670
Total		15594758	191169	100.000

(1*S*,2*R*,4*R*)-2-((*E*)-2-(naphthalen-1-yl)vinyl)-1,2,3,4-tetrahydro-1,4-epoxytriphenylene

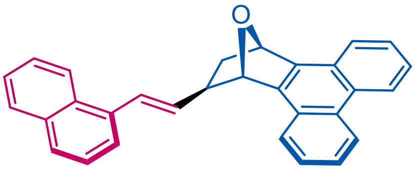

(3ei): compound **3ei** was prepared following general procedure D and the desired product was isolated through column chromatography (ethyl acetate/hexane = 5:95) in 77% (30.6 mg) yield with an er of 94:6 as sticky solid. $[\alpha]_D^{20}$ = + 370.30 (c = 0.274, $CHCl_3$).

^{1}H NMR ($CDCl_3$, 400 MHz): δ 8.79 (d, J = 4.3 Hz, 2H), 8.18 (d, J = 7.8 Hz, 1H), 8.05 (d, J = 4.0 Hz, 1H), 7.97 (dd, J = 6.4, 3.1 Hz, 1H), 7.92 – 7.88 (m, 1H), 7.82 (d, J = 8.1 Hz, 1H), 7.77 (d, J = 7.2 Hz, 1H), 7.71 (td, J = 6.3, 2.9 Hz, 5H), 7.58 – 7.50 (m, 4H), 7.32 – 7.24 (m, 2H), 6.58 (dd, J = 15.3, 9.7 Hz, 1H), 6.15 (d, J = 4.6 Hz, 1H), 5.91 (d, J = 2.8 Hz, 1H), 2.72 (td, J = 8.9, 3.5 Hz, 1H), 2.17 (dt, J = 11.8, 4.1 Hz, 1H), 1.91 (dd, J = 11.7, 8.0 Hz, 1H).

^{13}C{^{1}H} NMR (100 MHz, $CDCl_3$): δ 141.12, 140.07, 136.52, 134.79, 133.62, 131.01, 130.10, 128.51, 127.62, 127.05, 126.97, 126.91, 126.21, 125.86, 125.65, 124.20, 124.15, 123.69, 123.59, 83.95, 78.95, 44.66, 35.76.

HRMS (ESI): [M+H$^+$] calculated for $C_{30}H_{23}O^+$ is 399.1743; found 399.1741.

HPLC Condition: The enantiomeric excess was determined by Diacel Chiralpak IB, Hexane/IPA = 99/1, flow rate = 1.00 mL/min, λ = 254 nm, t(minor) = 16.546 min, t(major) = 17.986 min.

Chromatogram of (rac)-3ei **Chromatogram of (1*S*,2*R*,4*R*)-3ei**

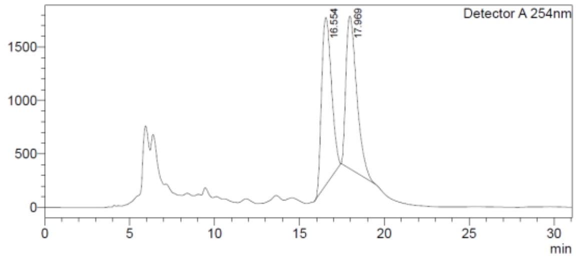

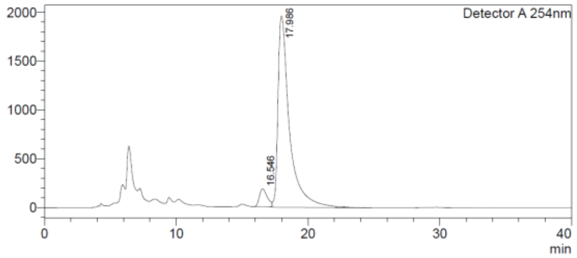

Detector A 254nm

Peak#	Ret. Time	Area	Height	Area%
1	16.554	63119037	1563630	49.937
2	17.969	63279294	1425372	50.063
Total		126398331	2989002	100.000

Detector A 254nm

Peak#	Ret. Time	Area	Height	Area%
1	16.546	7728166	184376	6.105
2	17.986	118865069	1958979	93.895
Total		126593235	2143355	100.000

***tert*-butyl-(1*S*,2*R*,4*R*)-2-((*E*)-2-(naphthalen-1-yl)vinyl)-1,2,3,4-tetrahydro-1,4-epiminonaphthalene-9-carboxylate (3fi):** compound **3fi** was prepared following general

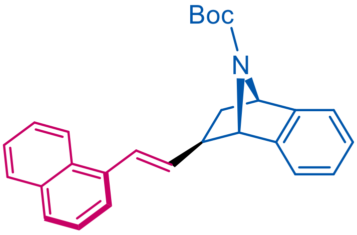

procedure D and the desired product was isolated through column chromatography (ethyl acetate/hexane = 5:95) in 77% (30.6 mg) yield with an er of 91:9 as white solid. $[\alpha]_D^{20}$ = + 87.50 (c = 0.160, $CHCl_3$).

^{1}H NMR ($CDCl_3$, 400 MHz): δ 8.16 – 8.10 (m, 1H), 7.84 (dd, J = 7.1, 2.3 Hz, 1H), 7.76 (d, J = 8.2 Hz, 1H), 7.62 (d, J = 7.2 Hz, 1H), 7.54 –

7.43 (m, 3H), 7.35 (d, J = 3.3 Hz, 1H), 7.28 (s, 1H), 7.21 (s, 1H), 7.19 – 7.16 (m, 3H), 6.37 (dd, J = 15.4, 9.0 Hz, 1H), 5.23 (s, 0H), 5.08 (d, J = 19.7 Hz, 1H), 2.63 (dt, J = 8.7, 4.4 Hz, 1H), 2.05 (dt, J = 11.9, 4.4 Hz, 1H), 1.89 – 1.77 (m, 1H), 1.37 (s, 9H).

^{13}C{^{1}H} NMR (100 MHz, $CDCl_3$): δ 155.31, 145.56, 136.02, 134.95, 133.60, 131.05, 128.49, 127.57, 126.92, 126.55, 126.46, 126.31, 125.87, 125.64, 123.75, 123.68, 119.68, 80.12, 66.61, 61.07, 45.41, 35.70, 28.22.

HRMS (ESI): [M+H$^+$] calculated for $C_{27}H_{28}NO_2^+$ is 398.2115; found 398.2115.

HPLC Condition: The enantiomeric excess was determined by Diacel Chiralpak IA, Hexane/IPA = 98/2, flow rate = 0.8 mL/min, λ = 254 nm, t(minor) = 30.683 min, t(major) = 23.069 min.

Chromatogram of (rac)-3fi

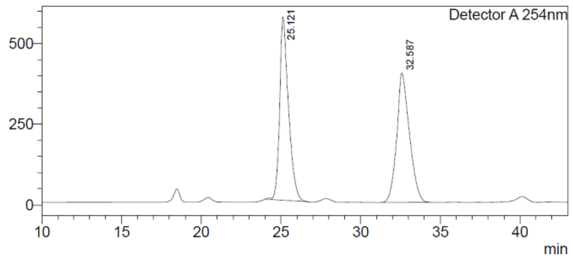

Peak#	Ret. Time	Area	Height	Area%
1	25.121	22019656	568358	49.664
2	32.587	22317177	401384	50.336
Total		44336834	969742	100.000

Chromatogram of (1*S*,2*R*,4*R*)-3fi

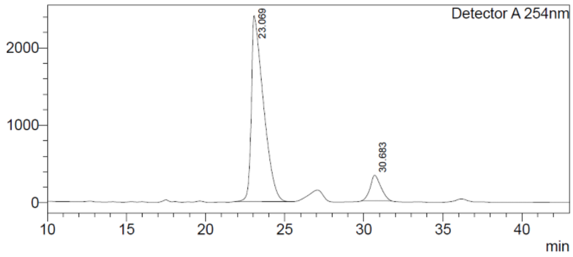

Peak#	Ret. Time	Area	Height	Area%
1	23.069	128460479	2403541	89.221
2	30.683	15519669	331274	10.779
Total		143980148	2734814	100.000

(3a*R*,4*S*,5*R*,7*R*,7a*S*)-5-((*E*)-2-(naphthalen-1-yl)vinyl)hexahydro-1*H*-4,7-epoxyisoindole-1,3(2*H*)-dione (3gi): compound **3gi** was prepared following general procedure D and the desired product was isolated through column chromatography (ethyl acetate/hexane = 40:60) in 70% (22.3 mg) yield with an er of 93:7 as brown solid. $[\alpha]_D^{20}$ = -207.54 (c = 0.180, $CHCl_3$).

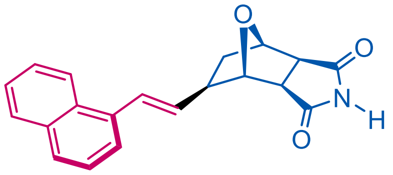

^{1}H NMR ($CDCl_3$, 400 MHz): δ 7.47 (t, J = 7.7 Hz, 2H), 7.39 (t, J = 7.2 Hz, 1H), 7.36 – 7.26 (m, 4H), 7.27 – 7.18 (m, 1H), 6.42 (d, J = 15.8 Hz, 1H), 6.11 (dd, J = 15.8, 9.0 Hz, 1H), 5.06 (d, J = 5.3 Hz, 1H), 4.82 (s, 1H), 3.13 (d, J = 7.2 Hz, 1H), 3.07 (d, J = 7.2 Hz, 1H), 2.74 (td, J = 8.7, 4.3 Hz, 1H), 2.05 (dd, J = 12.8, 8.5 Hz, 1H), 1.80 (dt, J = 12.8, 4.9 Hz, 1H).

^{13}C{^{1}H} NMR (100 MHz, $CDCl_3$): δ 176.26, 176.13, 136.89, 131.90, 131.53, 130.56, 129.31, 128.92, 128.72, 127.65, 126.60, 126.33, 84.42, 79.89, 49.93, 49.73, 45.58, 37.36.

HRMS (ESI): [M+H$^+$] calculated for $C_{20}H_{18}NO_3^+$ is 320.1281; found 320.1281.

HPLC Condition: The enantiomeric excess was determined by Diacel Chiralpak IB, Hexane/IPA = 80/20, flow rate = 1.00 mL/min, λ = 254 nm, t(minor) = 12.725 min, t(major) = 13.398 min.

Chromatogram of (rac)-3gi **Chromatogram of (3a*R*,4*S*,5*R*,7*R*,7a*S*)-3gi**

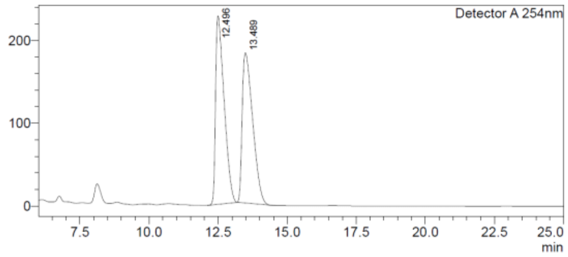

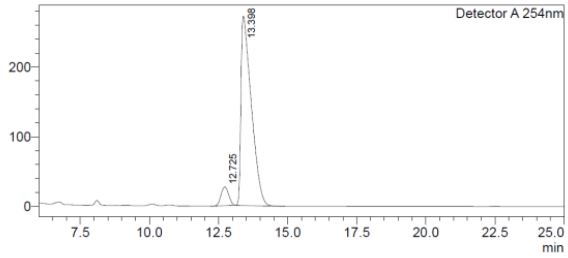

Detector A 254nm

Peak#	Ret. Time	Area	Height	Area%
1	12.496	4893652	227246	50.497
2	13.489	4797291	181115	49.503
Total		9690942	408361	100.000

Detector A 254nm

Peak#	Ret. Time	Area	Height	Area%
1	12.725	519521	26721	6.865
2	13.398	7047693	271603	93.135
Total		7567214	298324	100.000

(3a*R*,4*S*,5*R*,7*R*,7a*S*)-2-benzyl-5-((*E*)-2-(naphthalen-1-yl)vinyl)hexahydro-1*H*-4,7-epoxyisoindole-1,3(2*H*)-dione (3hi): compound **3hi** was prepared following general procedure D and the desired product was isolated through column chromatography (ethyl acetate/hexane = 30:70) in 71% (29 mg) yield with an er of 93:7 as white solid. $[\alpha]_D^{20}$ = -234.58 (c = 0.118, $CHCl_3$).

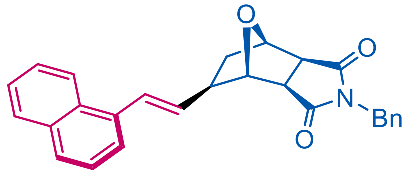

^{1}H NMR ($CDCl_3$, 400 MHz): δ 8.07 (d, *J* = 5.6 Hz, 1H), 7.85 (dd, *J* = 7.6, 2.1 Hz, 1H), 7.77 (d, *J* = 8.2 Hz, 1H), 7.56 – 7.48 (m, 3H), 7.46 – 7.39 (m, 1H), 7.35 – 7.27 (m, 5H), 7.15 (d, *J* = 15.6 Hz, 1H), 6.12 (dd, *J* = 15.6, 9.0 Hz, 1H), 5.00 (d, *J* = 5.3 Hz, 1H), 4.81 (s, 1H), 4.66 (s, 2H), 3.04 (d, *J* = 7.0 Hz, 1H), 2.95 (d, *J* = 7.0 Hz, 1H), 2.83 (td, *J* = 8.8, 4.3 Hz, 1H), 2.06 (dd, *J* = 12.7, 8.6 Hz, 1H), 1.88 – 1.78 (m, 1H).

^{13}C{^{1}H} NMR (100 MHz, $CDCl_3$): δ 176.60, 176.48, 135.36, 134.70, 134.43, 133.53, 130.99, 128.63, 128.52, 128.09, 127.85, 127.80, 127.55, 126.00, 125.74, 125.58, 123.76, 123.61, 83.88, 79.34, 49.77, 49.60, 45.71, 42.56, 37.27.

HRMS (ESI): [M+H$^+$] calculated for $C_{29}H_{28}NO_4^+$ is 454.2013; found 454.2013.

HPLC Condition: The enantiomeric excess was determined by Diacel Chiralpak IA, Hexane/IPA = 85/15, flow rate = 1.00 mL/min, λ = 254 nm, t(minor) = 24.494 min, t(major) = 16.288 min.

Chromatogram of (rac)-3hi

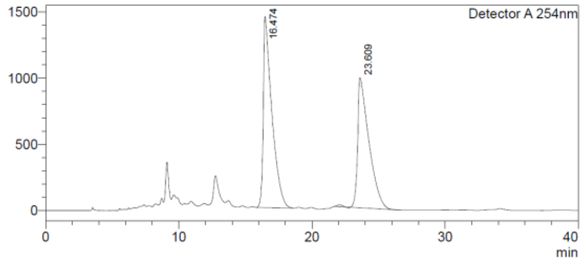

Detector A 254nm

Peak#	Ret. Time	Area	Height	Area%
1	16.474	64596134	1444500	52.656
2	23.609	58078639	984100	47.344
Total		122674773	2428600	100.000

Chromatogram of (3a*R*,4*S*,5*R*,7*R*,7a*S*)-3hi

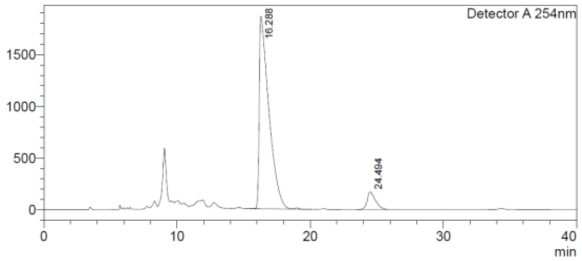

Detector A 254nm

Peak#	Ret. Time	Area	Height	Area%
1	16.288	93813048	1859494	92.708
2	24.494	7378985	169901	7.292
Total		101192034	2029394	100.000

(3*aR*,4*S*,5*R*,7*R*,7*aS*)-2-(2-(benzyloxy)ethyl)-5-((*E*)-2-(naphthalen-1-yl)vinyl)hexahydro-1*H*-4,7-epoxyisoindole-1,3(2*H*)-dione (3ii):

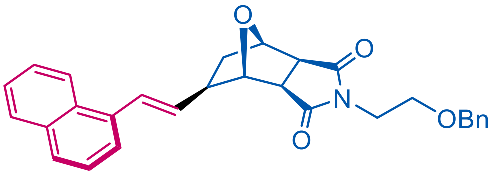

compound **3ii** was prepared following general procedure D and the desired product was isolated through column chromatography (ethyl acetate/hexane = 30:70) in 79% (35.8 mg) yield with an er of 97:3 as white solid. $[\alpha]_D^{20}$ = -219.24 (c = 0.173, $CHCl_3$).

^{1}H NMR ($CDCl_3$, 400 MHz): δ 8.07 (d, J = 7.9 Hz, 1H), 7.87 – 7.83 (m, 1H), 7.77 (d, J = 8.5 Hz, 1H), 7.55 – 7.48 (m, 3H), 7.44 (d, J = 7.3 Hz, 1H), 7.37 – 7.28 (m, 5H), 7.15 (d, J = 15.3 Hz, 1H), 6.11 (dd, J = 15.6, 8.8 Hz, 1H), 4.98 (d, J = 5.5 Hz, 1H), 4.79 (s, 1H), 4.52 (s, 2H), 3.75 (t, J = 5.5 Hz, 2H), 3.64 (t, J = 5.5 Hz, 2H), 3.02 (d, J = 6.7 Hz, 1H), 2.94 (d, J = 7.3 Hz, 1H), 2.83 (td, J = 8.5, 4.0 Hz, 1H), 2.10 – 2.03 (m, 1H), 1.83 (dt, J = 12.8, 4.9 Hz, 1H).

^{13}C{^{1}H} NMR (100 MHz, $CDCl_3$): δ 176.83, 176.72, 137.87, 134.78, 134.49, 133.55, 131.02, 128.53, 128.35, 127.85, 127.68, 127.63, 127.55, 126.00, 125.74, 125.58, 123.77, 123.64, 83.84, 79.27, 72.64, 66.10, 49.72, 49.54, 45.71, 38.50, 37.30.

HRMS (ESI): [M+H$^+$] calculated for $C_{29}H_{28}NO_4^+$ is 454.2013; found 454.2013.

HPLC Condition: The enantiomeric excess was determined by Diacel Chiralpak IA, Hexane/IPA = 85/15, flow rate = 1.00 mL/min, λ = 210 nm, t(minor) = 17.180 min, t(major) = 18.680 min.

Chromatogram of (rac)-3ii

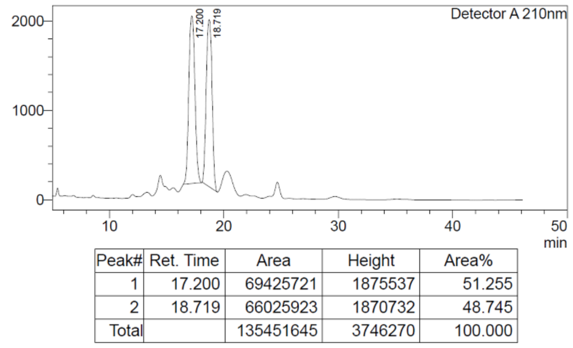

Peak#	Ret. Time	Area	Height	Area%
1	17.200	69425721	1875537	51.255
2	18.719	66025923	1870732	48.745
Total		135451645	3746270	100.000

Chromatogram of (3*aR*,4*S*,5*R*,7*R*,7*aS*)-3ii

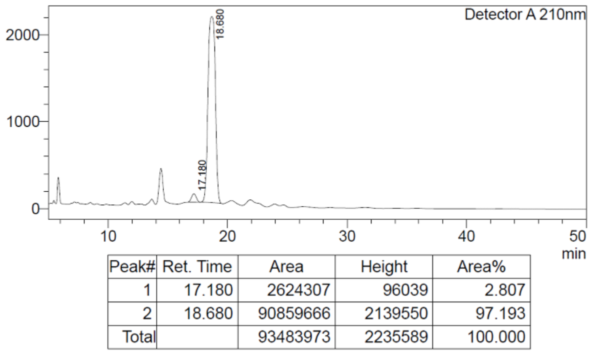

Peak#	Ret. Time	Area	Height	Area%
1	17.180	2624307	96039	2.807
2	18.680	90859666	2139550	97.193
Total		93483973	2235589	100.000

(3*aR*,4*S*,5*R*,7*R*,7*aS*)-5-((*E*)-2-(naphthalen-1-yl)vinyl)-2-phenylhexahydro-1*H*-4,7-epoxyisoindole-1,3(2*H*)-dione (3ji):

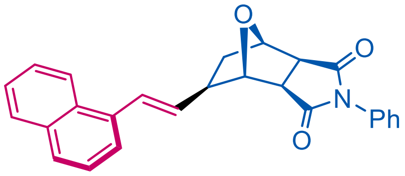

compound **3ji** was prepared following general procedure D and the desired product was isolated through column chromatography (ethyl acetate/hexane = 25:75) in 74% (29.2 mg) yield with an er of 97:3 as white solid. $[\alpha]_D^{20}$ = -239. 40 (c = 0.189, $CHCl_3$).

^{1}H NMR ($CDCl_3$, 400 MHz): δ 8.07 (d, J = 7.9 Hz, 1H), 7.84 (dd, J = 7.4, 1.9 Hz, 1H), 7.76 (d, J = 8.2 Hz, 1H), 7.55 – 7.37 (m, 8H), 7.29 – 7.26 (m, 2H), 7.17 (d, J = 15.6 Hz, 1H), 6.13 (dd, J = 15.6, 9.0 Hz, 1H), 5.09 (d, J = 5.3 Hz, 1H), 4.90 (s, 1H), 3.19 (d, J = 7.2 Hz, 1H), 3.11 (d, J = 6.9 Hz, 1H), 2.88 (td, J = 8.5, 4.3 Hz, 1H), 2.12 (dd, J = 12.8, 8.6 Hz, 1H), 1.88 (dt, J = 12.8, 4.9 Hz, 1H).

^{13}C{^{1}H} NMR (100 MHz, $CDCl_3$): δ 176.28, 176.19, 134.77, 134.59, 133.69, 131.85, 131.16, 129.33, 128.96, 128.68, 128.04, 127.84, 126.61, 126.17, 125.91, 125.74, 123.94, 123.78, 84.48, 79.94, 49.96, 49.79, 45.91, 37.48.

HRMS (ESI): [M+H$^+$] calculated for $C_{26}H_{22}NO_3^+$ is 396.1594; found 396.1593.

HPLC Condition: The enantiomeric excess was determined by Diacel Chiralpak IB, Hexane/IPA = 80/20, flow rate = 1.00 mL/min, λ = 210 nm, t(minor) = 19.155 min, t(major) = 22.135 min.

Chromatogram of (rac)-3ji

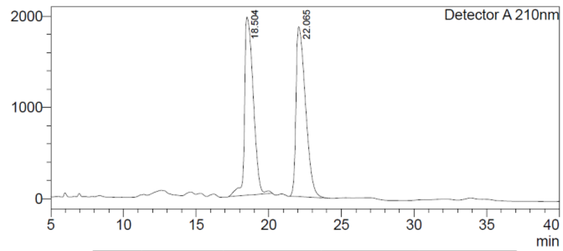

Peak#	Ret. Time	Area	Height	Area%
1	18.504	81940183	1953138	49.032
2	22.065	85176850	1861314	50.968
Total		167117034	3814453	100.000

Chromatogram of (1*R*,2*S*,4*R*)-3ji

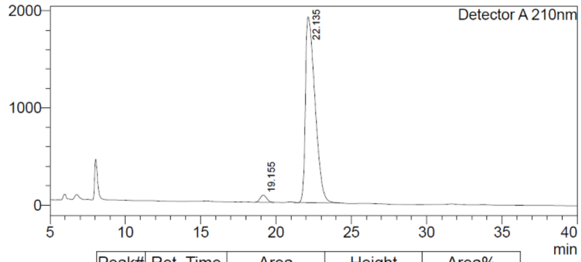

Peak#	Ret. Time	Area	Height	Area%
1	19.155	2188771	72853	2.526
2	22.135	84474654	1909100	97.474
Total		86663425	1981953	100.000

4-((3a*R*,4*S*,5*R*,7*R*,7a*S*)-5-((*E*)-2-(naphthalen-1-yl)vinyl)-1,3-dioxooctahydro-2*H*-4,7-epoxyisoindol-2-yl)benzonitrile (3ki): compound **3ki** was prepared following general procedure D and the desired product was isolated through column chromatography (ethyl acetate/hexane = 30:70) in 79% (33.1 mg) yield with an er of 97:3 as off-white solid. $[\alpha]_D^{20}$ = -327.24 (c = 0.205, $CHCl_3$).

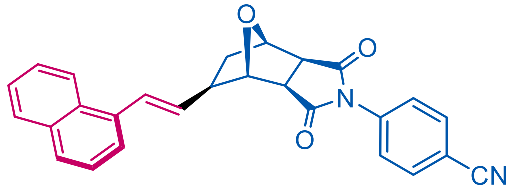

^{1}H NMR ($CDCl_3$, 400 MHz): δ 8.08 (d, *J* = 9.6 Hz, 1H), 7.87 – 7.83 (m, 1H), 7.80 – 7.75 (m, 3H), 7.56 – 7.49 (m, 5H), 7.44 (t, *J* = 7.8 Hz, 1H), 7.19 (d, *J* = 15.6 Hz, 1H), 6.13 (dd, *J* = 15.6, 9.2 Hz, 1H), 5.11 (d, *J* = 5.0 Hz, 1H), 4.91 (s, 1H), 3.24 (d, *J* = 6.9 Hz, 1H), 3.16 (d, *J* = 7.3 Hz, 1H), 2.92 (td, *J* = 8.7, 4.1 Hz, 1H), 2.15 (dd, *J* = 12.8, 8.7 Hz, 1H), 1.91 (dt, *J* = 12.8, 4.8 Hz, 1H).

^{13}C{^{1}H} NMR (100 MHz, $CDCl_3$): δ 175.35, 175.26, 135.61, 134.34, 133.57, 132.93, 131.01, 128.59, 128.01, 127.93, 126.91, 126.07, 125.81, 125.60, 123.81, 123.58, 117.99, 112.34, 84.43, 79.90, 49.85, 49.68, 45.72, 37.33.

HRMS (ESI): [M+H$^+$] calculated for $C_{27}H_{21}N_2O_3^+$ is 421.1547; found 421.1547.

HPLC Condition: The enantiomeric excess was determined by Diacel Chiralpak IB, Hexane/IPA = 80/20, flow rate = 1.00 mL/min, λ = 210 nm, t(minor) = 30.469 min, t(major) = 41.170 min.

Chromatogram of (rac)-3ki

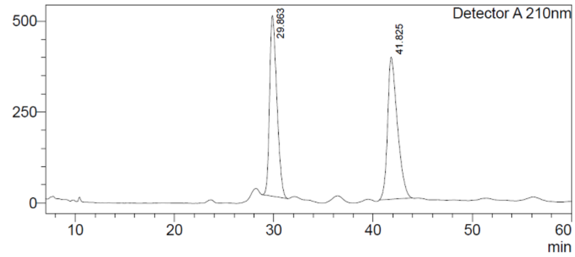

Peak#	Ret. Time	Area	Height	Area%
1	29.863	25316953	496524	48.525
2	41.825	26855760	390209	51.475
Total		52172714	886733	100.000

Chromatogram of (3a*R*,4*S*,5*R*,7*R*,7a*S*)-3ki

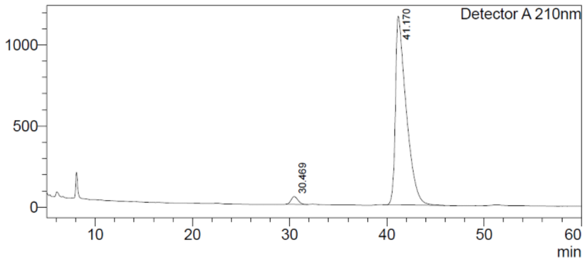

Peak#	Ret. Time	Area	Height	Area%
1	30.469	2324036	47370	2.517
2	41.170	90020914	1163474	97.483
Total		92344949	1210844	100.000

(3a*R*,4*S*,5*R*,7*R*,7a*S*)-2-(4-acetylphenyl)-5-((*E*)-2-(naphthalen-1-yl)vinyl)hexahydro-1*H*-4,7-epoxyisoindole-1,3(2*H*)-dione (3li): compound **3li** was prepared following general procedure D and the desired product was isolated through column chromatography (ethyl acetate/hexane = 30:70) in 73% (31.9 mg) yield with an er of 96:4 as white solid. $[\alpha]_D^{20}$ = -247.17 (c = 0.188, $CHCl_3$).

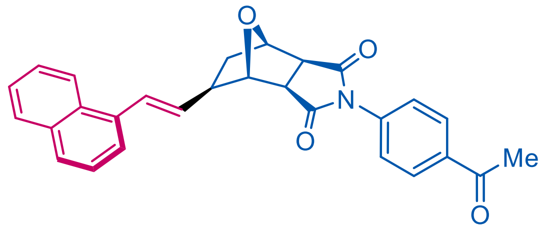

^{1}H NMR ($CDCl_3$, 400 MHz): δ 8.10 – 8.02 (m, 3H), 7.85 (dd, J = 7.7, 2.0 Hz, 1H), 7.78 (d, J = 8.2 Hz, 1H), 7.56 – 7.48 (m, 3H), 7.47 – 7.41 (m, 3H), 7.19 (d, *J* = 15.6 Hz, 1H), 6.14 (dd, *J* = 15.6, 9.0 Hz, 1H), 5.11 (d, *J* = 5.3 Hz, 1H), 4.92 (s, 1H), 3.22 (d, *J* = 7.1 Hz, 1H), 3.14 (d, *J* = 7.2 Hz, 1H), 2.90 (d, *J* = 4.3 Hz,1H), 2.62 (s, 3H), 2.14 (dd, *J* = 12.8, 8.6 Hz, 1H), 1.90 (d, *J* = 12.7 Hz, 1H).

^{13}C{^{1}H} NMR (100 MHz, $CDCl_3$): δ 197.15, 175.81, 175.71, 136.95, 135.88, 134.62, 134.54, 133.70, 131.17, 129.28, 128.71, 128.10, 127.96, 126.57, 126.21, 125.94, 125.75, 123.96, 123.75, 84.56, 80.02, 50.03, 49.84, 45.87, 37.48, 26.82.

HRMS (ESI): [M+H$^+$] calculated for $C_{28}H_{24}NO_4^+$ is 438.1700; found 438.1701.

HPLC Condition: The enantiomeric excess was determined by Diacel Chiralpak IB, Hexane/IPA = 75/25, flow rate = 1.00 mL/min, λ = 254 nm, t(minor) = 33.716 min, t(major) = 26.211 min.

Chromatogram of (rac)-3li

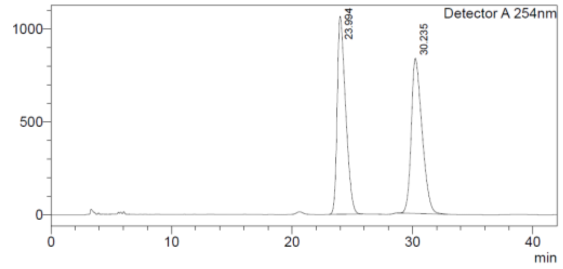

Detector A 254nm

Peak#	Ret. Time	Area	Height	Area%
1	23.994	51630373	1066280	49.806
2	30.235	52031785	835291	50.194
Total		103662158	1901571	100.000

Chromatogram of (3a*R*,4*S*,5*R*,7*R*,7a*S*)-3li

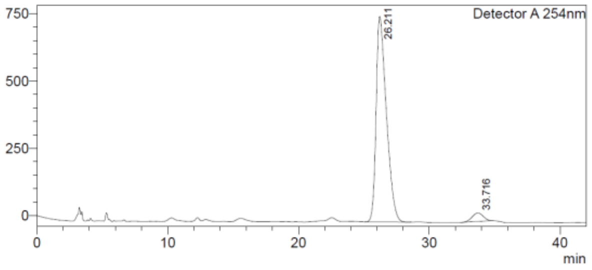

Detector A 254nm

Peak#	Ret. Time	Area	Height	Area%
1	26.211	41601961	763325	95.717
2	33.716	1861574	31394	4.283
Total		43463536	794719	100.000

dibenzyl (1*R*,2*S*,3*R*,4*S*,5*R*)-5-((*E*)-styryl)-7-oxabicyclo[2.2.1]heptane-2,3- dicarboxylate (3na) : compound **3na** was prepared following general procedure D and the desired product was isolated through column chromatography (ethyl acetate/hexane = 15:85) in 62% (29.03 mg) yield with an er of 84:16 as white solid. $[\alpha]_D^{20}$ = -144.28 (c = 0.112, $CHCl_3$).

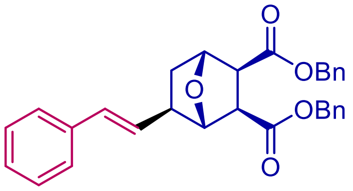

^{1}H NMR ($CDCl_3$, 400 MHz): δ 7.39 – 7.28 (m, 15H), 6.38 (d, *J* = 15.9 Hz, 1H), 6.14 (dd, *J* = 17.2, 9.1 Hz, 1H), 5.14 – 5.00 (m, 3H), 4.96 (d, *J* = 12.2 Hz, 2H), 4.77 (s, 1H), 3.13 (d, *J* = 9.6 Hz, 1H), 3.07 (d, *J* = 9.5 Hz, 1H), 2.64 – 2.57 (m, 1H), 1.91 (dd, *J* = 12.7, 8.4 Hz, 1H), 1.69 (dt, *J* = 12.8, 5.0 Hz, 1H).

^{13}C{^{1}H} NMR (100 MHz, $CDCl_3$): δ 170.66, 170.54, 136.87, 135.51, 135.47, 132.36, 129.67, 128.62, 128.47, 128.38, 128.35, 128.21, 128.11, 127.26, 126.07, 83.31, 78.65, 66.74, 66.68, 51.85, 51.62, 45.93, 37.68.

HRMS (ESI): [M+H$^+$] calculated for $C_{30}H_{29}O_5^+$ is 469.2010; found 469.2008.

HPLC Condition: The enantiomeric excess was determined by Diacel Chiralpak IB, Hexane/IPA = 90/10, flow rate = 1.00 mL/min, λ = 254 nm, t(minor) = 19.488 min, t(major) = 30.671 min.

Chromatogram of (rac)-3na

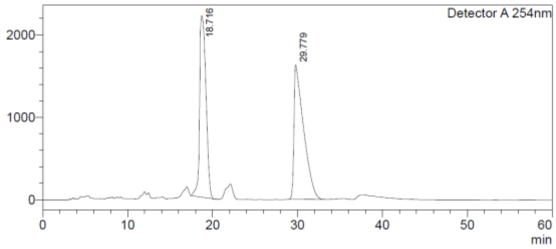

Detector A 254nm

Peak#	Ret. Time	Area	Height	Area%
1	18.716	114895905	2200980	47.291
2	29.779	128057598	1628586	52.709
Total		242953503	3829566	100.000

Chromatogram of (1*R*,2*S*,3*R*,4*S*,5*R*)-3na

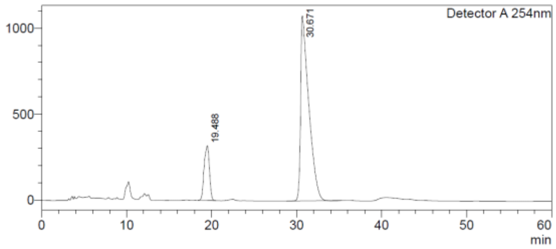

Detector A 254nm

Peak#	Ret. Time	Area	Height	Area%
1	19.488	13779695	319186	16.064
2	30.671	72001451	1076860	83.936
Total		85781145	1396046	100.000

7. Synthetic Utility

(i) Reduction of 3ji:

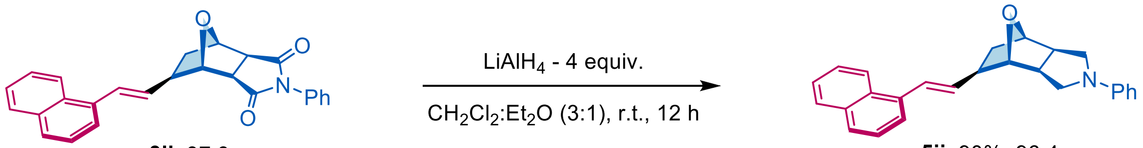

3ji, 97:3 er   **5ji**; 90%, 96:4 er

To a solution of **3ji** (0.05 mmol, 19.8 mg) in DCM:Et$_2$O (3:1), LiAlH$_4$ (0.2 mmol, 15.2 mg) was added at 0 °C under argon atmosphere. The reaction mixture was allowed to stir at room temperature for 12 hours. The reaction was quenched with saturated NH$_4$Cl solution and extracted in DCM, combined organic layer was dried over anhydrous Na$_2$SO$_4$ and concentrated. The crude mixture was purified by flash column chromatography to afford **5ji** in 90% (17.8 mg) yield with an er of 96:4. $[\alpha]_D^{20}$ = -173.41 (c = 0.112, CHCl$_3$).

(3a*S*,4*S*,5*R*,7*R*,7a*R*)-5-((*E*)-2-(naphthalen-1-yl)vinyl)-2-phenyloctahydro-1*H*-4,7-epoxyisoindole (5ji):

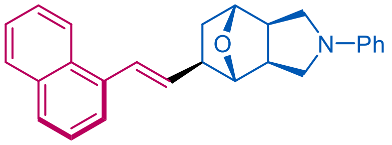

^{1}H NMR (CDCl$_3$, 500 MHz): δ 8.15 – 8.11 (m, 1H), 7.85 (dd, *J* = 7.6, 1.9 Hz, 1H), 7.76 (d, *J* = 8.2 Hz, 1H), 7.58 (dd, *J* = 7.2, 1.1 Hz, 1H), 7.54 – 7.47 (m, 2H), 7.46 – 7.42 (m, 1H), 7.25 – 7.22 (m, 2H), 7.14 (d, *J* = 15.5 Hz, 1H), 6.75 (t, *J* = 7.3 Hz, 1H), 6.67 (d, *J* = 8.0 Hz, 2H), 6.22 (dd, *J* = 15.5, 9.2 Hz, 1H), 4.52 (d, *J* = 5.3 Hz, 1H), 4.31 (s, 1H), 3.74 (td, *J* = 9.2, 5.6 Hz, 2H), 2.97 (ddd, *J* = 9.9, 5.9, 4.4 Hz, 2H), 2.79 – 2.67 (m, 3H), 1.96 (dd, *J* = 12.3, 8.5 Hz, 1H), 1.70 (ddd, *J* = 12.3, 5.1, 3.6 Hz, 1H).

^{13}C{^{1}H} NMR (125 MHz, CDCl$_3$): δ 136.93, 135.04, 133.73, 131.20, 129.17, 128.65, 127.67, 126.24, 125.99, 125.78, 123.85, 123.74, 113.85, 86.02, 81.15, 53.95, 48.12, 47.96, 45.82, 37.44, 29.81.

HRMS (ESI): [M+H$^+$] calculated for $C_{26}H_{26}NO^+$ is 368.2009; found 368.2011.

HPLC Condition: The enantiomeric excess was determined by Diacel Chiralpak IB, Hexane/IPA = 98/2, flow rate = 1.00 mL/min, λ = 254 nm, t(minor) = 32.768 min, t(major) = 29.118 min.

Chromatogram of (rac)-5ji

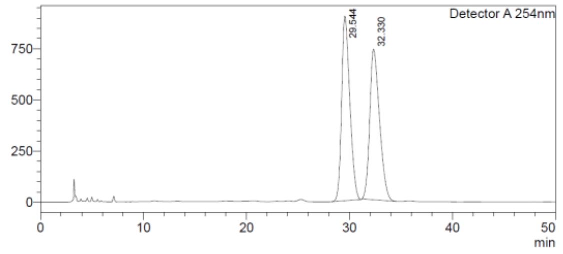

Detector A 254nm

Peak#	Ret. Time	Area	Height	Area%
1	29.544	49555374	900996	50.558
2	32.330	48461019	735426	49.442
Total		98016393	1636422	100.000

Chromatogram of (3*aS*,4*S*,5*R*,7*R*,7*aR*)-5ji

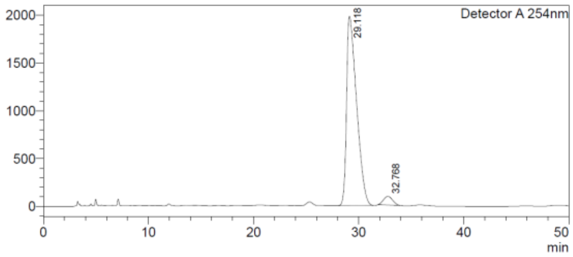

Detector A 254nm

Peak#	Ret. Time	Area	Height	Area%
1	29.118	131371717	1982011	95.957
2	32.768	5534770	90785	4.043
Total		136906487	2072796	100.000

8. Control Experiments

(i) Radical trapping experiment

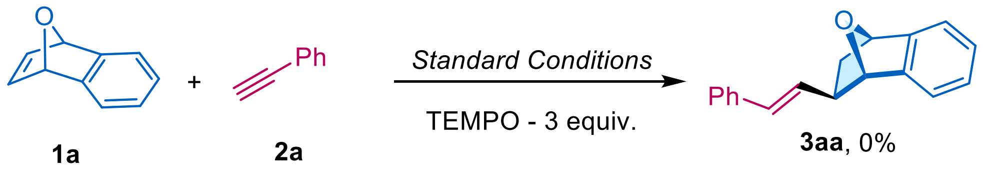

In an oven-dried 4 mL borosilicate glass vial equipped with magnetic stirrer, was charged with 4CzIPN (1.6 mg, 0.002 mmol), **L9** (5.5 mg, 0.01 mmol), $Co(OAc)_2.4H_2O$ (2.5 mg, 0.01 mmol), alkyne (**2a**, 0.3 mmol), strained olefin (**1a**, 0.1 mmol), TEMPO (0.3 mmol) DIPEA (0.035 ml, 0.2 mmol), MeCN (1 mL) under inert atmosphere and the reaction vial was sealed with cap and Teflon. The vial was exposed to Kessil blue LED (wavelength 440 nm) for 24 h at room temperature. After 24 hours, all the volatiles were removed under reduced pressure, and the crude reaction mixture was analysed by ^1H NMR, which indicates no product formation in the presence of radical quencher.

(ii) Reactivities under metal-based reductant

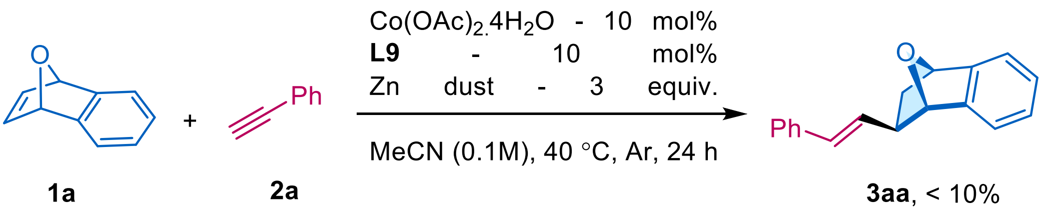

In an oven-dried Schlenk tube equipped with magnetic stirrer, was charged with Zn dust (0.3 mmol), **L9** (5.5 mg, 0.01 mmol), $Co(OAc)_2.4H_2O$ (2.5 mg, 0.01 mmol), alkyne (**2a**, 0.3 mmol), strained olefin (**1a**, 0.1 mmol) MeCN (1 mL) under inert atmosphere and the reaction tube was sealed. The reaction tube was kept at 40 °C for 24 h. After 24 hours, all the volatiles were removed under reduced pressure, and the crude reaction mixture was analysed by ^1H NMR, which indicates trace amount of product formation (<10%) in the presence of Zn dust.

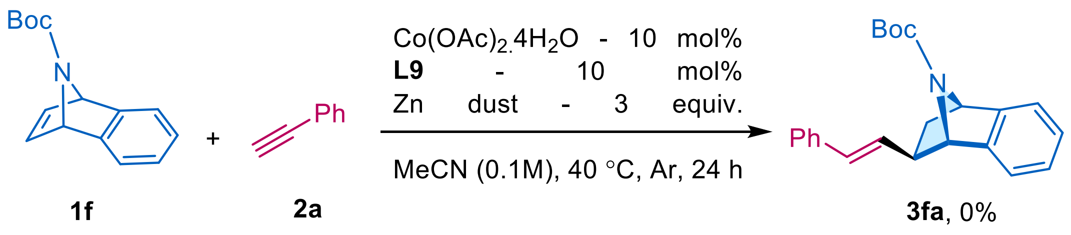

In an oven-dried Schlenk tube equipped with magnetic stirrer, was charged with Zn dust (0.3 mmol), **L9** (5.5 mg, 0.01 mmol), $Co(OAc)_2.4H_2O$ (2.5 mg, 0.01 mmol), alkyne (**2a**, 0.3 mmol), strained olefin (**1f**, 0.1 mmol) MeCN (1 mL) under inert atmosphere and the reaction tube was sealed and kept at 40 °C for 24 h. After 24 hours, all the volatiles were removed under reduced pressure, and the crude reaction mixture was analysed by ^1H NMR, which indicates absolutely no product formation in the presence of Zn dust.

Both the results indicate the high catalytic efficiency of cobalt/photoredox methodology over the traditional reductive coupling methodology.

(iii) D-scrambling Studies

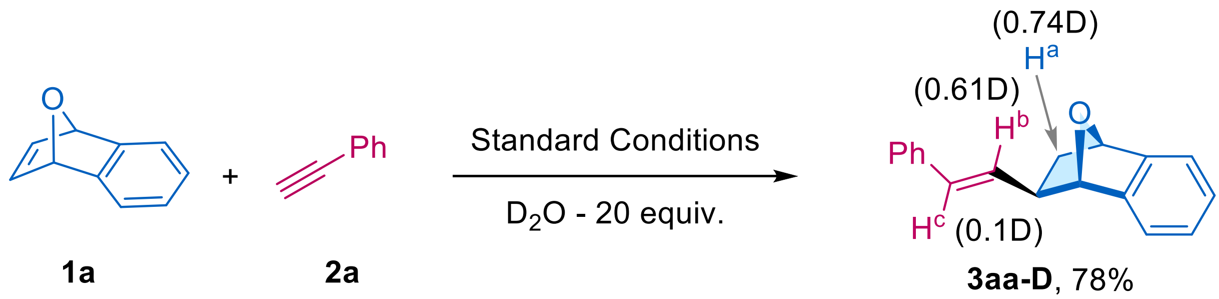

In an oven-dried 4 mL borosilicate glass vial equipped with magnetic stirrer, was charged with 4CzIPN (1.6 mg, 0.002 mmol), **L9** (5.5 mg, 0.01 mmol), $Co(OAc)_2.4H_2O$ (2.5 mg, 0.01 mmol), strained olefin (**1a**, 0.1 mmol), alkyne (**2a**, 0.3 mmol), DIPEA (0.035 ml, 0.2 mmol), D2O (0.036 mL, 2 mmol), MeCN (1 mL, 0.1 M) under inert atmosphere and the reaction vial was sealed with cap and Teflon. The vial was exposed to Kessil blue LED (wavelength 440 nm) for 24 h at room temperature. After 24 hours, all the volatiles were removed under reduced pressure, and the corresponding product (**3aa-D**) was filtered through Celite using DCM as eluent and the filtrate was evaporated under reduced pressure. Crude NMR was submitted to determine the percentage of deuterium incorporated in **3aa-D**.

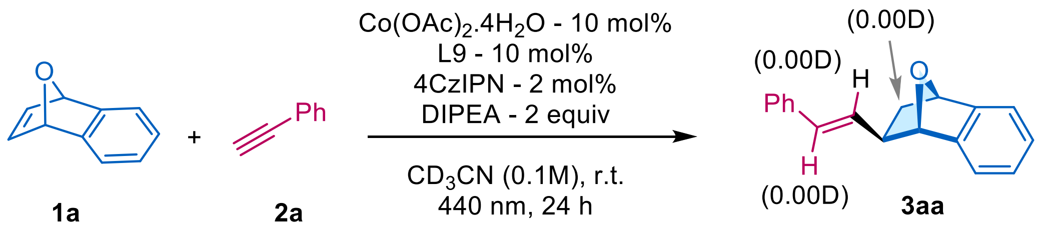

In an oven-dried 4 mL borosilicate glass vial equipped with magnetic stirrer, was charged with 4CzIPN (1.6 mg, 0.002 mmol), **L9** (5.5 mg, 0.01 mmol), $Co(OAc)_2.4H_2O$ (2.5 mg, 0.01 mmol), strained olefin (**1a**, 0.1 mmol), alkyne (**2a**, 0.3 mmol), DIPEA (0.035 ml, 0.2 mmol), CD_3CN (1 mL,

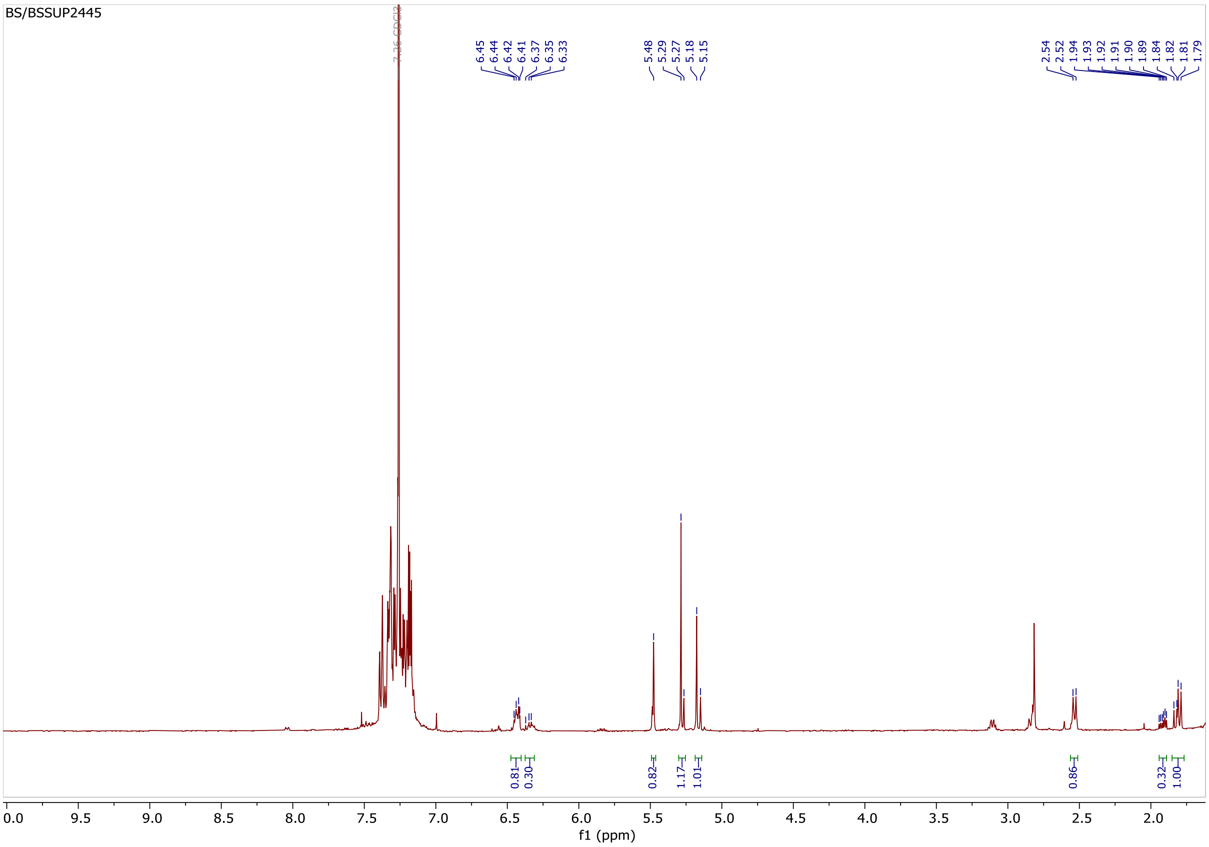

Figure-S2: ^{1}H NMR spectra of crude reaction mixture.

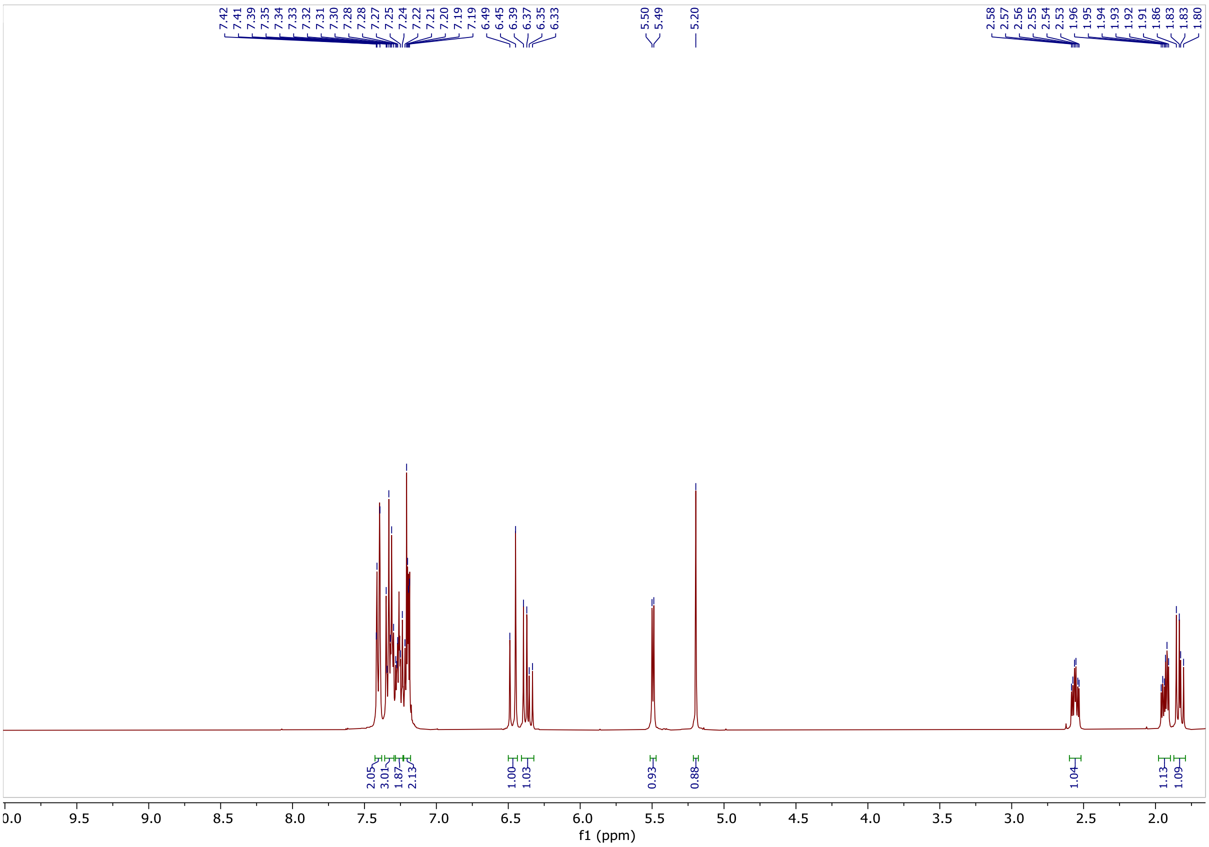

Figure-S3: ^{1}H NMR spectra of **3aa**.

0.1 M) under inert atmosphere and the reaction vial was sealed with cap and Teflon. The vial was exposed to Kessil blue LED (wavelength 440 nm) for 24 h at room temperature. After 24 hours, all the volatiles were removed under reduced pressure, and the corresponding product (**3aa**) was isolated through flash column chromatography using ethyl acetate/hexane as eluent.

9. Kinetics Analysis

9.1 Reaction profile

In an oven-dried 4 mL glass vial equipped with magnetic stirrer, was charged with 4CzIPN (1.6 mg, 0.002 mmol, 2 mol%), **L9** (5.5 mg, 0.01 mmol, 10 mol%), $Co(OAC)_2.4H_2O$ (2.5 mg, 0.01 mmol, 10 mol%), strained olefin (**1a**, 14.4 mg, 0.1 mmol, 1 equiv.) and alkyne (30.6 mg, 0.3 mmol, 3 equiv.), DIPEA (0.035 ml, 0.2 mmol, 2 equiv.), MeCN (1 mL, 0.1M) under inert atmosphere and the reaction vial was sealed with cap and Teflon. The vial was exposed to Kessil blue LED (wavelength 440 nm) for desired reaction time (3h, 6h, 9h, 12h, 15 h) at room temperature. The reaction mixture was passed through a small pad of silica; all the volatiles were removed under reduced pressure and crude NMR was submitted to determine the NMR yield of the desired product (**3aa**) using anisole as an internal standard.

Reaction Time (h)	NMR Yield of 3aa (%)	NMR Yield of 1a ( )	ee of 3aa (%)
0	0	0	-
3	12	87	84
6	28	72	84
9	40	60	84
12	49	51	84
15	61	38	84

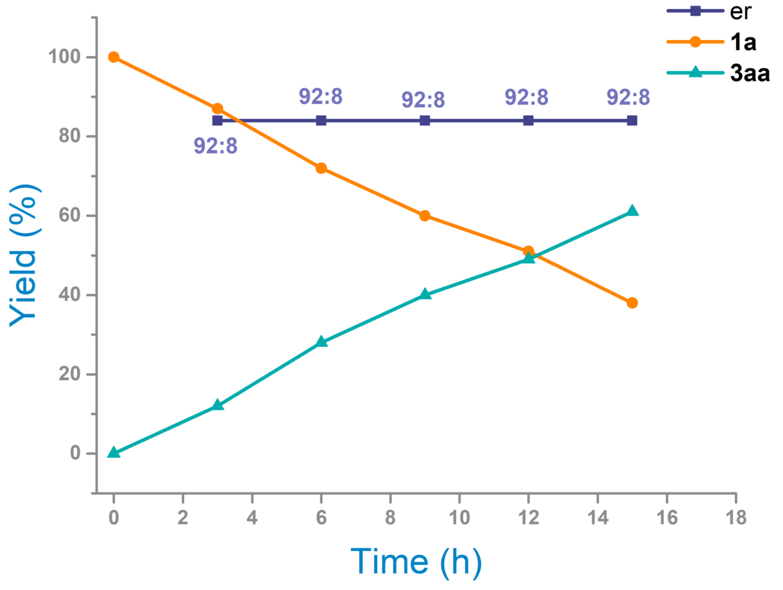

Figure-S4: Reaction kinetics of desymmetrization reaction.

9.2 Light on-off experiment

In an oven-dried 4 mL glass vial equipped with magnetic stirrer, was charged with 4CzIPN (1.6 mg, 0.002 mmol, 2 mol%), **L9** (5.5 mg, 0.01 mmol, 10 mol%), $Co(OAC)_2.4H_2O$ (2.5 mg, 0.01 mmol, 10 mol%), strained olefin (**1a**, 14.4 mg, 0.1 mmol, 1 equiv.) and alkyne (30.6 mg, 0.3 mmol, 3 equiv.), DIPEA (0.035 ml, 0.2 mmol, 2 equiv.), MeCN (1 mL, 0.1M) under inert atmosphere and the reaction vial was sealed with cap and Teflon. For an interval of 3 hours, the reaction mixture was exposed to Kessil blue LED (wavelength 440 nm) and then next 3 hours it was kept under dark condition. This cycle was repeated up to 7 cycles (21 h) and in each interval the reaction mixture was analysed through crude NMR analysis using 1,3,5-trimethoxy benzene as internal standard.

Reaction Time (h)	Time interval (h)	NMR Yield of 3aa (%)
0	0	0
0 - 3 (on)	3	12
3 - 6 (off)	3	12
6 - 9 (on)	3	29
9 - 12 (off)	3	29
12 - 15 (on)	3	40
15 - 18 (off)	3	40
18 - 21 (on)	3	49

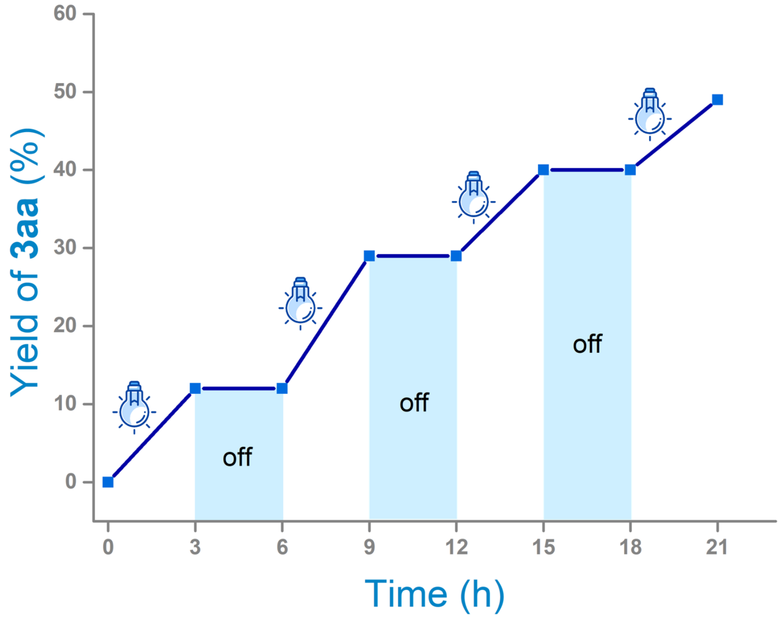

Figure-S5: Plot of on-off experiment.

10. Determination of Quantum Yield

10.A. Determination of light intensity of the Blue LED

The photon flux was determined by using standard ferrioxalate actinometry. A 0.15 M solution of ferrioxalate was made by mixing 0.737 g of potassium ferrioxalate hydrate into 10 mL of 0.05 M H_2SO_4. A buffered solution of phenanthroline was made by mixing 25 mg of phenanthroline and 5.63 g of sodium acetate in 25 mL of 0.5 M H_2SO_4. Both solutions were kept in the dark. To determine the photon flux, 2.0 mL of the ferrioxalate solution was placed in a cuvette and irradiated for 60.0 seconds at λ = 440 nm placing 5 cm away from 40 W Kessil blue LED lamp. After irradiation, 0.35 mL of the phenanthroline solution was added to the cuvette. The solution was then kept for 1 h to permit the Fe^{2+}ions to completely coordinate to phenanthroline. The absorbance of the solution was determined at 510 nm. A non-irradiated sample was also prepared and the absorbance at 510 nm was determined. Conversion was calculated using eq. 1

$$\text{mole of } Fe^{2+} \text{ ion} = \frac{V.\ \Delta A}{\mathcal{E}.\ l} \quad \text{............} \quad (1)$$

Where V is the total volume (0.00310 L) of the solution after addition of the phenanthroline,ΔA is the difference in absorbance at 510 nm between the irradiated and non-irradiated solutions, l is the path length (1.000 cm), and ε is the molar absorptivity at 510 nm (11,100L mol^{-1} cm^{-1}).

$$\text{moles of } Fe^{2+} \text{ ion} = 5.7252 \text{ x } 10^{-7}$$

The photon flux of the light source can be determined following eq. 2

$$\text{Photon flux} = \frac{mole\ of\ \text{Fe2+}}{\varphi.\ t.\ f} \quad \text{...........} \quad (2)$$

Where, ϕ is the quantum yield for the ferrioxalate actinometer (0.999 for a 0.15 M solution at λ = 440 nm), t is the time (60 s), and f is the fraction of light absorbed at λ = 440 nm, $f = 1 - 10^{-A}$.

$$\text{Calculated } f = 1 - 10^{-1.526} = 0.97021$$

$$\text{Photon flux} = \frac{5.7252 \times 10^{-7}\ mol}{0.999 \times 60\ s \times 0.9702} = 9.84493 \text{ x } 10^{-8} \text{ Einstein } s^{-1}$$

10.B Quantum yield calculation

In an oven-dried 4 mL glass vial equipped with magnetic stirrer, was charged with 4CzIPN (0.002 mmol, 2 mol%), **L9** (0.01 mmol, 10 mol%), $Co(OAc)_2.4H_2O$ (0.01 mmol, 10 mol%), alkyne **2a** (0.3 mmol, 3 equiv.) and strained olefin **1a** (0.1 mmol, 1 equiv.), DIPEA (0.2 mmol, 2 equiv.), MeCN (1 mL, 0.1 M) under inert atmosphere and the reaction vial was sealed with cap and Teflon. The vial was exposed to Kessil blue LED (wavelength 440 nm) for 3 h at room temperature. After 3 hours, the reaction mixture was filtered through small pad of celite, and all the volatiles were removed

under reduced pressure. The crude reaction mixture was submitted for ^{1}H NMR analysis using 1,3,5-trimethoxy benzene as internal standard. Moles of product obtained is 1.24 x 10^{-5} .

The quantum yield was calculated as follows:

$$\text{Quantum yield } (\phi) = \frac{moles\ of\ product}{flux \times t \times f}$$

Where, flux is the photon flux determined by ferrioxalate actinometry (9.84493 x 10^{-8} Einstein s^{-1}), t is the time (10800 s), and f (> 0.999) is the fraction of light absorbed by the reaction mixture at 440 nm under the reaction condition mentioned above.

$$\Phi = \frac{1.24 \times 10^{-5}}{9.84493 \times 10^{-8} \times 10800 \times 1} = 0.01160$$

Hence, the quantum yield (Φ) of the reaction is **0.01160** for 3 hours.

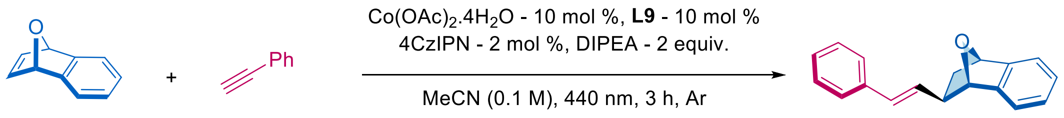

1a **2a** **3aa**

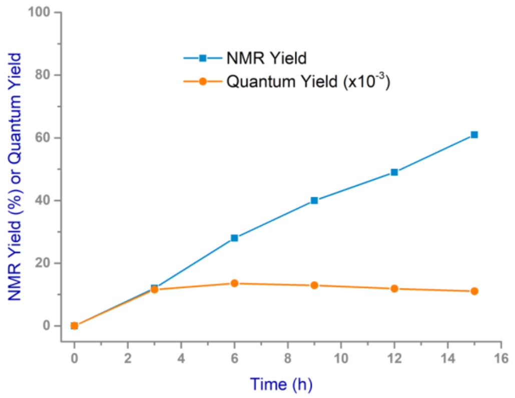

Reaction Time (h)	NMR Yield (%)	Quantum Yield ( )
0	0	0
3	12	0.01160
6	28	0.01357
9	40	0.01292
12	49	0.01187
15	61	0.01108

Figure S6. Correlation plot of reaction kinetics and quantum yield for reductive coupling.

By following the same method, we have calculated the quantum yield at four different time scale for both coupling reactions and the correlation graph of reaction kinetics and quantum yield is plotted.

11. Fluorescence Quenching Study

Fluorescence measurements were carried out using a HORIBA Scientific's Fluoromax (steady state and lifetime bench top spectrofluorometer), using 1 cm path length quartz cuvette equipped with a Teflon® septum. A 0.040 mM solution was prepared by mixing 4CzIPN in CH3CN by an appropriate dilution of 0.003 M stock solution and taken in a fluorescence cuvette (filled up to 2.4 mL). The excitation and emission slit widths were fixed at 2 nm for data collection. Fluorescence emission spectra of 4CzIPN were collected from 470 nm to 650 nm with an excitation wavelength of 450 nm. Λmax(emission) of 4CzIPN was observed at 556 nm. For each fluorescence quenching experiment, 18 μL of 0.1 M solution of the individual reactants was added to 4CzIPN solution (0.040 mM) taken in a fluorescence cuvette, and emission spectra were recorded after each sequential addition. Figure (Figure S7 – S8) shows a decrease in emission

intensity after addition of DIPEA, indicating that it interacts with the excited state of 4CzIPN much faster than strained olefin (1a) or alkyne (2a). Relevant quenching spectra and Stern-Volmer studies of all reactants are shown below.

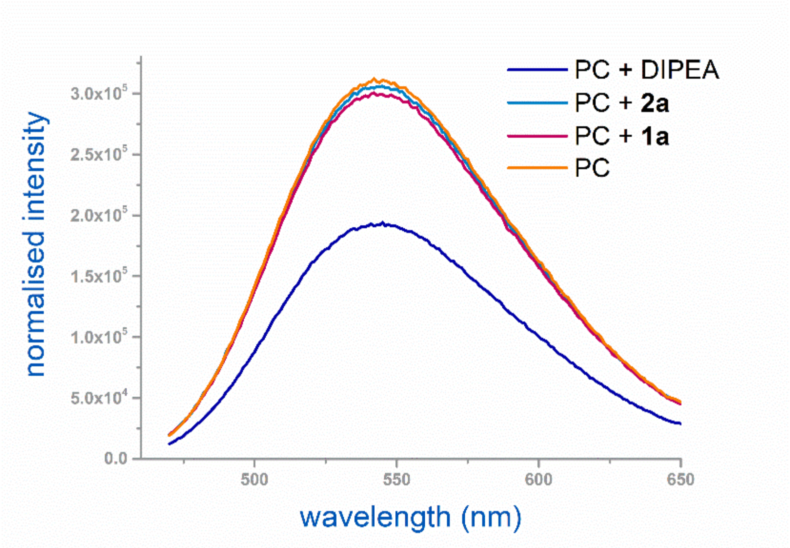

Figure-S7: Combined fluorescence quenching spectra of 0.04 mM solution of 4CzIPN in MeCN with strained olefin (**1a**), alkyne (**2a**), DIPEA as quencher.

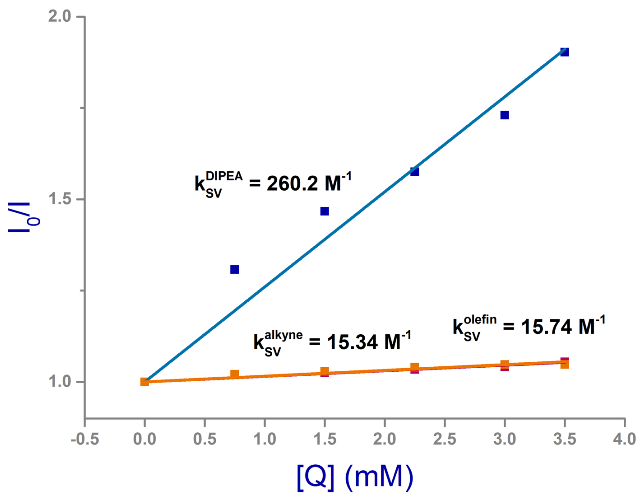

Figure-S8: Combined Stern-Volmer plot of 0.04 mM solution of 4CzIPN in MeCN with strained olefin (**1a**), alkyne (**2a**), DIPEA as quencher.

12. UV-Vis Study

UV-Visible kinetics and absorption spectroscopic studies were performed on Agilent 8453 diode-array spectrophotometer to carry out kinetics experiments spectrophotometrically in 1 cm quartz cells (λ = 190–1100 nm range). To monitor the kinetics of the reaction, we modified the UV-Vis set up by incorporating the Kessil lamp as photon source.

To confirm the formation of low-valent Co(I) active species, we recorded the absorption spectra of reaction mixture aliquot both in the presence and absence of blue light irradiation under inert atmosphere. Initially two bands around 410 nm and 480 nm appeared without light irradiation suggesting the formation of Co(II)**L9** intermediate (purple line). A probable band for low-valent

Co(I) could be seen at 620 nm and 745 nm (red line, Figure-S9) upon irradiation under 440 nm blue LED while the initial bands around 410 and 480 nm decayed. Further this newly appeared bands also decayed upon addition of alkyne (**2a**) and strained olefin (**1a**) but no new prominent band appeared in the UV-Vis spectrum.

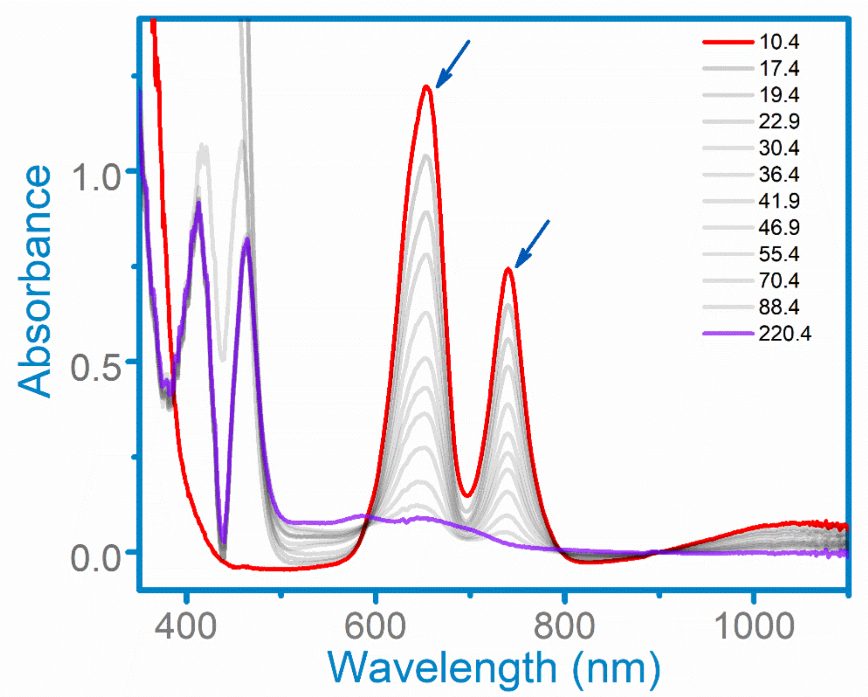

Figure-S9: UV-Vis kinetic spectra of $Co(OAc)_2$**L9** in the presence of 4CzIPN and DIPEA without irradiation (violet line), under irradiation (red line).

13. Crystallographic Summary of 3ag

Crystals suitable for diffraction were obtained from layering of the dichloromethane solution with pentane. Ellipsoids are drawn at the 50% probability level.

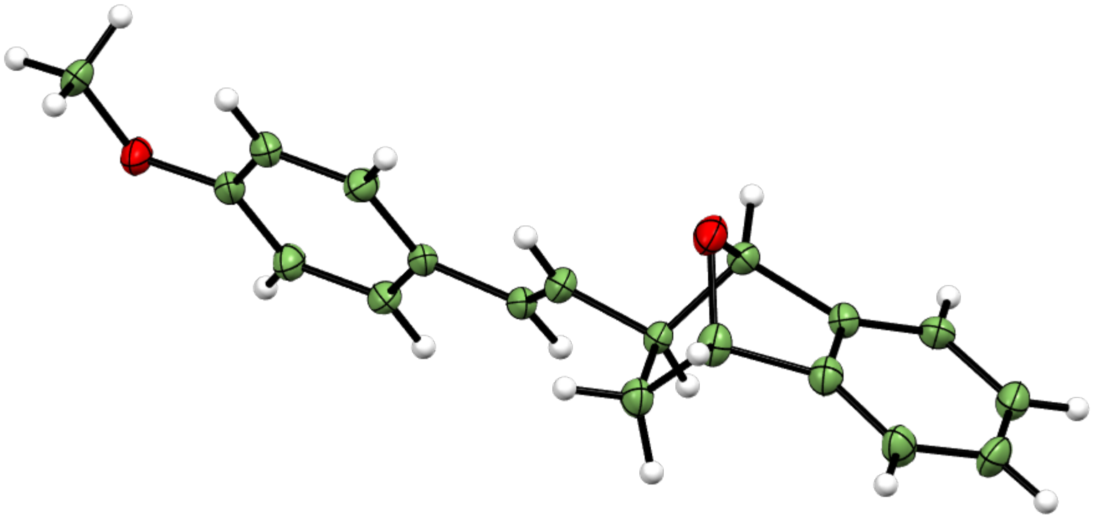

Fig. S10: Structure of **3ag** in solid state (CCDC 2500153). CCD Bruker SMART APEX diffractometer, structures were solved by direct methods and refined (SHELXL-97) by full matrix least squares based on F2.

Crystal data and structure refinement for 3ag:

Identification code	180cta_0_om_a
Empirical formula	C19H18O2
Formula weight	278.33
Temperature/K	296 K
Crystal system	monoclinic
Space group	P1211
a/Å	6.3624 (4)
b/Å	15.6454 (11)
c/Å	7.4038 (5)
α/°	90
β/°	90.759 (2)
γ/°	90
Volume/Å^3	736.93 (9)
Z	2
ρ_{calc}g/cm^3	1.254
μ/mm-1	0.080
F(000)	296.0
Radiation	Mo Kα (λ = 0.71073)
Flack parameter	0.1

14. Circular Dichroism Spectra of 3ag

CD spectra for both enantiomers of **3ag** were recorded in ethanol at 10 μM concentration in a 1×10 mm quartz cell with a Teflon stopper.

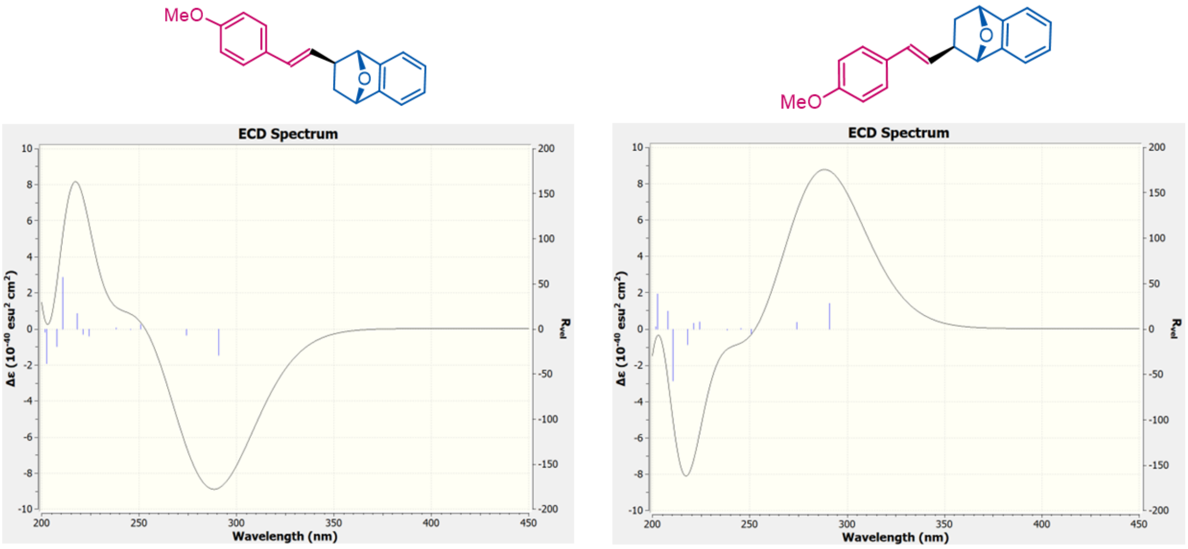

Fig. S11. Computed CD spectra of both enantiomers of the product at the M06(SMD, ethanol)/def2-TZVPP level of theory.

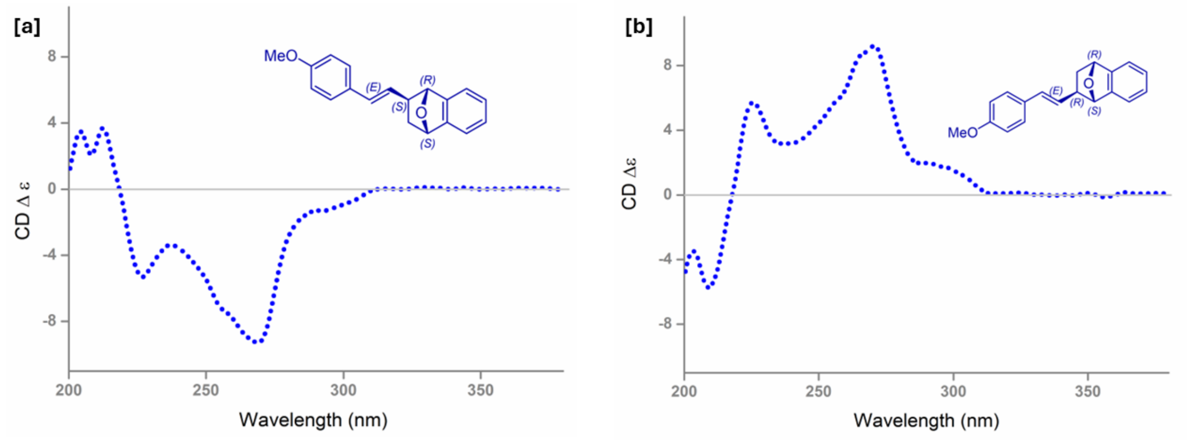

Fig. S12. Experimental CD spectra of both the enantiomers (minor isomer (left), major isomer (right)) in ethanol solvent. Minor isomer obtained through a catalytic reaction with the enantiomeric ligand of **L9**.

15. Computational Methodology

Computations were carried out employing density functional theory (DFT) implemented in the Gaussian 16 software.[10] Geometry optimizations were carried out with the PBE0[11] hybrid density functional including D3 version of Grimme's dispersion with Becke-Johnson damping.[12] For geometrical optimizations, we used Ahlrichs' split valence polarization basis set (def2-SVP)[13] for all the atoms except cobalt which was treated with Ahlrichs' valence triple-ζ polarization basis set (def2-TZVP). No symmetry constraints were imposed during structural optimizations. The frequency analyses were executed on the optimized geometries at the same level to ascertain the nature of stationary points on the potential energy surface either as minima or the transition states characterized by first-order saddle points and also to obtain the thermochemical energy values. The minima were identified by having a full set of real frequencies, whereas the transition states possess only one imaginary frequency. The transition states were searched using the linear synchronous transit (LST) method,[14] and subsequent geometry optimizations were performed by utilizing the default Berny algorithm, implemented in the Gaussian 16. Intrinsic reaction coordinate (IRC) calculations were enforced to ensure that the transition state connects the corresponding real minima.[15] Furthermore, to improve the accuracy of the energies obtained from the PBE0-D3BJ/def2-TZVP(Co)/def2-SVP(non-metals) level of theory, single-point calculations were performed on the optimized geometries with the M06 meta-hybrid density functional[16] employing Ahlrichs' valence triple-ζ basis set with two sets of polarization functions (def2-TZVPP) for all the atoms. Solvation energies in acetonitrile (MeCN) solvent (ε = 35.688) were evaluated by a self-consistent reaction field (SCRF) approach using the SMD continuum solvation model.[17] Unless stated otherwise, ΔG values were used throughout the text. The ΔG values were obtained by adding the ΔE energy terms at the M06(SMD, acetonitrile)/def2-TZVPP(all atoms) level to the respective free energy corrections at the PBE0-D3BJ/def2-TZVP(Co)/def2-SVP(non-metals) level in the gas phase. All the intermediates and transition states were reported in their most stable ground electronic states. As it is known that translational and rotational entropies in solution for association/dissociation processes are overestimated/underestimated, and that the deviation in the free energies is approximately 1.89 kcal/mol from the standard state (1 atm) to 1 M in solution,[18] we have reduced by 1.89 kcal/mol the free energy for addition steps and we have added 1.89 kcal/mol to the free energy of the dissociation steps.

To account the steric at the substrate binding catalytic pocket in terms of buried volume (%V_{bur}), we have used SambVca 2.1 software.[19] To enrich our understanding toward the origin of relative activation barriers of unfavored transition states with respect to the favored one, distortion–interaction analysis was performed.[20] The distortion–interaction model allows for partitioning the activation barrier ($\Delta E^{\ddagger}$) of a transition state into destabilizing distortion energy (ΔE_{dist}) and stabilizing interaction (ΔE_{int}) energy terms.

Estimation of energy barriers

Applying the Marcus–Hush theory of electron transfer,[21] the free energy barrier ($\Delta G^{\ddagger}_{MH}$) of a singlet electron transfer process can be estimated according to the following equation (1):

$$\Delta G^{\ddagger}_{\mathrm{MH}} = \frac{(\Delta G_r + \lambda)^2}{4\lambda} \qquad (1)$$

where ΔG_r is the free energy change of the step, λ is the reorganization energy, which has two components, inner sphere and outer sphere. However, the first one is considered to be neglected, and hence, the total λ will the outer sphere reorganization energy, which can be calculated by the equation (2):

$$\lambda = \lambda_{\mathrm{outer}} = (332\ \mathrm{kcal/mol})\left(\frac{1}{2a_1} + \frac{1}{2a_2} - \frac{1}{\mathrm{R}}\right)\left(\frac{1}{\varepsilon_{\mathrm{opt}}} - \frac{1}{\varepsilon}\right) \qquad (2)$$

where a_1 and a_2 are the radii of donor and acceptor, respectively. R is the sum of a_1 and a_2. ε_{opt} and ε are the optical dielectric constant (ε_{opt} = 1.807) and static dielectric constant (ε = 35.688) of acetonitrile solvent, respectively.

Table S7. Calculated free energy barriers ($\Delta G^{\ddagger}_{MH}$) of single electron transfer (SET) steps and their relevant parameters.

SET	a_1 (Å)	a_2 (Å)	R (Å)	λ	ΔG_r	$\Delta G^{\ddagger}_{MH}$
SET1	7.15	6.74	13.89	12.58	−16.5	0.3
SET2	7.15	7.06	14.21	12.28	−3.1	1.7
SET3	6.66	4.47	11.13	16.93	−13.4	0.2
SET4	4.53	6.58	11.11	16.81	−3.6	2.6
SET5	7.15	6.58	13.73	12.75	9.1	9.3

Active catalyst generation

Under visible-light irradiation, the photoexcited ^{3}**PC** (4CzIPN) undergoes reductive quenching (SET3) in the presence of DIPEA (**N**), generating the radical cation ^{2}**N**$^+$ and ^{2}**PC**$^-$ (Figure S13). This step is exergonic by13.4 kcal/mol with a negligible free energy barrier of 0.2 kcal/mol, estimated using the Marcus-Hush theory (Table S1). The result is consistent with our earlier report on similar complexes.[22] Subsequently, proton transfer from ^{2}**N**$^+$ to **N** affords the protonated amine **NH**$^+$ and α-amino radical species ^{2}**N**$_1$ via transition state ^{2}**N-TS** and an energy barrier of only 7.0 kcal/mol. An exergonic reduction of ^{4}**Co(OAc)**$_2$ to ^{3}**Co(OAc)**$_2^-$ by ^{2}**N**$_1$ then yields the iminium ion **N**$_2^+$ with an estimated energy barrier of 2.6 kcal/mol (SET4). The exergonic release of OAc$^-$ from

intermediate ^{3}**Co(OAc)$_2^-$** produces ^{3}**A**, the active catalyst involves in the Co-catalytic cycle. An alternative pathway, the reduction of Co(II) to Co(I) by ^{2}**PC$^-$** (SET5, Figure S14), was also considered but found to be both thermodynamically and kinetically less favorable compared to SET4.

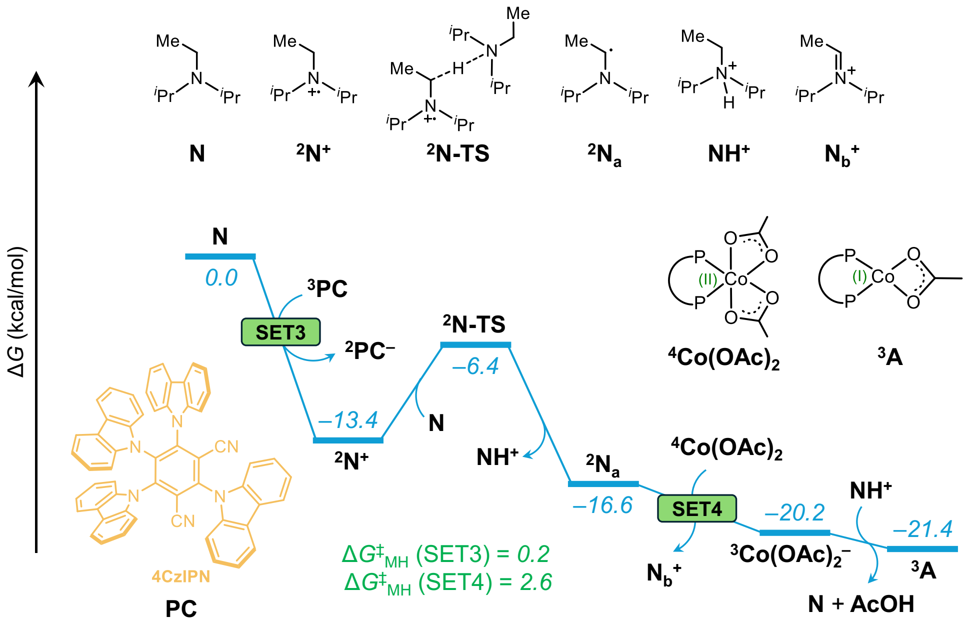

Figure S13. Computed free-energy profile for active catalyst generation was obtained at the M06(SMD, acetonitrile)/def2-TZVPP//PBE0-D3BJ/def2-TZVP(Co)/def2-SVP(non-metals) level of theory. Superscripts 4, 3 and 2 denote quartet, triplet and doublet spin states, respectively, while all other species are assumed to be singlets unless otherwise indicated.

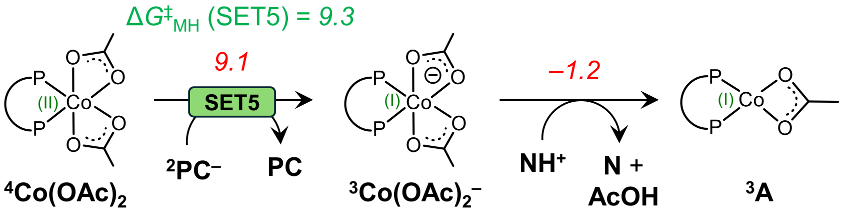

Figure S14. Energetics (free energy in kcal/mol) of Co(II) to Co(I) reduction by reduced state photocatalyst. For other conventions refer Figure S11.

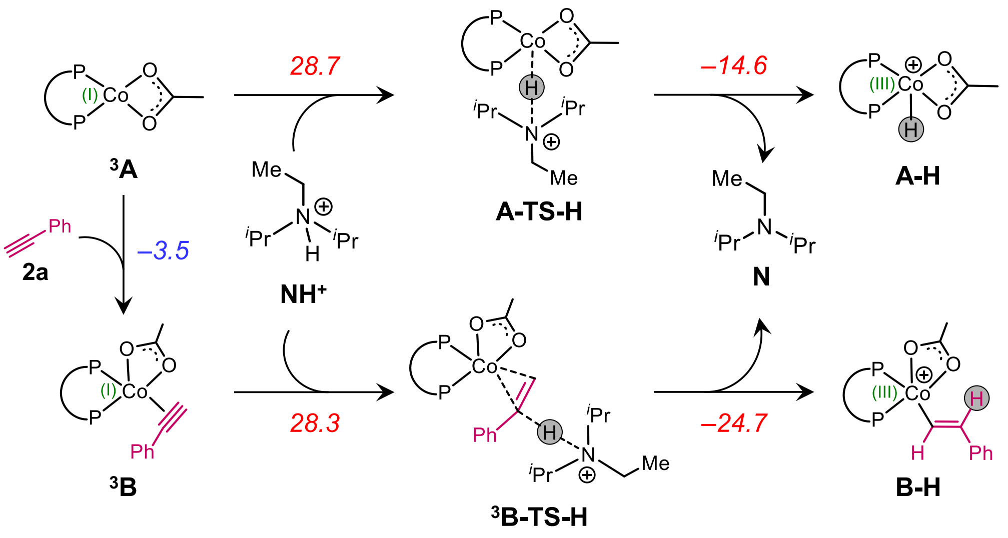

Figure S15. Energetics (free energy in kcal/mol) of alternative routes of protonation pathways leading to Co(III)-H and Co(III)-vinyl complexes. For other conventions refer Figure S11.

Cartesian coordinates (Å) of reactants, product, intermediates and transition states at PBE0-D3BJ/def2-TZVP(Co)/def2-SVP(non-metals) level of theory. *E* represents the absolute electronic energy in Hartree at M06(SMD, acetonitrile)/def2-TZVPP level of theory.

PC

94

E: -2480.87991739

N	5.755442	13.275419	4.776828
N	8.923087	10.717867	10.314401
N	6.918582	12.475829	8.007905
N	8.144361	10.944566	3.570926
N	9.925554	9.054148	4.707004
N	10.170789	8.908434	7.527724
C	8.571693	10.712657	7.750868
C	7.665945	11.624822	7.187157
C	9.884708	7.656966	4.727078
C	9.178611	9.903119	5.526505
C	5.166341	13.409269	9.129158
C	8.763933	10.707537	9.167031
C	8.282359	10.836320	4.955113
C	11.516401	8.711106	7.198488
C	5.548727	12.372033	8.247819
C	7.423610	13.564034	8.718932
C	11.387822	8.311671	3.116802
C	4.640306	11.432036	7.767176
H	4.948824	10.636048	7.086415
C	10.783132	7.162739	3.755530
C	10.839624	9.459942	3.730246
C	8.725841	14.055741	8.766510
H	9.537520	13.576556	8.215365
C	6.361015	14.171736	9.426327

C	7.516971	11.675144	5.792372
C	8.121449	10.333036	1.367931
C	9.314068	9.835129	6.932766
C	6.549597	12.562311	5.227038
C	11.261454	10.743168	3.392647
H	10.869028	11.628502	3.892254
C	3.831157	13.504637	9.530836
H	3.514815	14.297496	10.212321
C	3.317079	11.551341	8.181327
H	2.578682	10.833488	7.817311
C	6.620333	15.301133	10.207591
H	5.813552	15.786863	10.761026
C	9.752945	7.848012	8.346761
C	8.503146	11.726907	1.452264
C	11.960736	7.511164	7.794582
C	7.469081	8.592526	2.966053
H	7.264495	8.262884	3.984581
C	9.082023	6.806545	5.482757
H	8.359699	7.186785	6.204603
C	7.903941	9.885243	2.688823
C	8.492115	7.580481	8.875922
H	7.648755	8.257255	8.729739
C	12.346318	8.460010	2.111851
H	12.777492	7.581087	1.627497
C	8.499464	12.076429	2.820707
C	8.880489	13.344148	3.255453
H	8.891647	13.613126	4.312896
C	9.228811	5.436197	5.288987
H	8.623218	4.750705	5.885778
C	7.308356	7.722701	1.892262
H	6.988985	6.696420	2.085682
C	7.942069	9.446022	0.303764
H	8.108585	9.775544	-0.724114
C	8.956661	15.180018	9.553522
H	9.968402	15.586723	9.616943
C	10.908844	5.783764	3.573120
H	11.600822	5.387465	2.826709
C	2.914372	12.574995	9.052006
H	1.867683	12.639777	9.356282
C	12.369722	9.527926	6.462498
H	12.032581	10.473024	6.037043
C	12.212690	10.867405	2.384343
H	12.542989	11.865967	2.090111
C	10.837447	6.960550	8.523083
C	7.548673	8.139972	0.575223
H	7.413451	7.432642	-0.245638
C	7.918313	15.796808	10.267330
H	8.134733	16.677185	10.875966
C	8.878120	12.676900	0.499823
H	8.888861	12.418320	-0.561310
C	9.248991	13.948385	0.923797

H	9.542286	14.702073	0.190136
C	12.745736	9.740315	1.743128
H	13.488697	9.871144	0.953595
C	13.678327	9.092929	6.278699
H	14.361448	9.705516	5.686464
C	10.137264	4.926521	4.350583
H	10.231120	3.845958	4.223927
C	9.252401	14.273367	2.287418
H	9.546099	15.277148	2.602077
C	13.282538	7.099767	7.607415
H	13.641902	6.172852	8.059663
C	8.335704	6.406651	9.608360
H	7.359366	6.178951	10.041676
C	10.656406	5.790400	9.263516
H	11.485909	5.093903	9.404218
C	9.404283	5.520945	9.805331
H	9.248658	4.611023	10.388555
C	14.130440	7.888913	6.837647
H	15.163711	7.575499	6.674748

³PC

94

E: -2480.78882300

N	5.998602	13.514138	4.736500
N	8.881156	10.645061	10.348330
N	6.949030	12.528569	8.004541
N	8.205560	10.920182	3.559557
N	10.002518	9.127935	4.710012
N	10.213322	8.905895	7.536721
C	8.621135	10.762693	7.792418
C	7.735435	11.716466	7.175874
C	9.925539	7.744564	4.634500
C	9.254363	9.955390	5.562466
C	5.147233	13.410012	9.093630
C	8.760289	10.701860	9.188014
C	8.386856	10.922641	4.950194
C	11.579007	8.778585	7.305215
C	5.559037	12.483157	8.107476
C	7.437117	13.473475	8.901252
C	11.306105	8.480182	2.945855
C	4.650773	11.669874	7.432017
H	4.981541	10.956833	6.675466
C	10.714786	7.299863	3.543752
C	10.854657	9.584635	3.700905
C	8.752093	13.891183	9.105962
H	9.580589	13.446675	8.552472
C	6.347488	14.048512	9.596515
C	7.614569	11.803405	5.801719
C	8.035486	10.094746	1.428646
C	9.359473	9.844702	6.937999
C	6.722817	12.747796	5.224039

C	11.300150	10.879340	3.448460
H	10.991707	11.716053	4.075061
C	3.791576	13.529530	9.397932
H	3.455084	14.237063	10.158951
C	3.303502	11.808257	7.752880
H	2.567366	11.189999	7.234644
C	6.587987	15.050753	10.535683
H	5.760585	15.511125	11.080069
C	9.798146	7.840995	8.338417
C	8.503826	11.464303	1.358109
C	12.048852	7.607402	7.947284
C	7.321716	8.588576	3.229233
H	7.125252	8.401477	4.285292
C	9.160303	6.859653	5.396170
H	8.530856	7.206780	6.213712
C	7.840889	9.807217	2.798553
C	8.518062	7.531409	8.797045
H	7.666206	8.180306	8.586626
C	12.177440	8.688059	1.878425
H	12.535845	7.845406	1.283222
C	8.578993	11.938164	2.692287
C	9.039206	13.222983	2.993700
H	9.097127	13.576430	4.024123
C	9.230562	5.508183	5.078407
H	8.656649	4.794701	5.673283
C	7.055638	7.622150	2.261184
H	6.667879	6.650376	2.573927
C	7.751938	9.117552	0.475506
H	7.896330	9.320970	-0.587907
C	8.966621	14.886540	10.052872
H	9.986004	15.227250	10.246042
C	10.761312	5.942424	3.237894
H	11.359549	5.583731	2.397653
C	2.875214	12.727566	8.720389
H	1.810270	12.813418	8.946110
C	12.433640	9.616721	6.590574
H	12.068375	10.531172	6.123321
C	12.161154	11.067871	2.368893
H	12.503486	12.077971	2.134890
C	10.909062	7.009082	8.607851
C	7.275689	7.877432	0.901919
H	7.057540	7.100151	0.166693
C	7.900735	15.459165	10.762646
H	8.104190	16.240211	11.498209
C	8.890912	12.298067	0.310701
H	8.849691	11.946593	-0.722525
C	9.342293	13.583137	0.604931
H	9.645443	14.248540	-0.206216
C	12.588993	9.988944	1.586804
H	13.264733	10.167076	0.747808
C	13.771501	9.248713	6.497660

H	14.459157	9.884328	5.935201
C	10.024389	5.050606	4.016764
H	10.058485	3.982262	3.792929
C	9.414796	14.036583	1.931995
H	9.768659	15.049469	2.135590
C	13.398394	7.262077	7.844471
H	13.776794	6.361458	8.333397
C	8.366960	6.368203	9.546282
H	7.377184	6.107369	9.927799
C	10.730989	5.847197	9.363208
H	11.579153	5.193171	9.578470
C	9.458552	5.533514	9.829346
H	9.305281	4.630502	10.424008
C	14.251382	8.081705	7.111899
H	15.307603	7.820274	7.018777

^{2}PC$^-$

94

E: -2480.99206414

N	5.712542	13.240792	4.780077
N	8.964954	10.758086	10.316416
N	6.924371	12.470076	8.002872
N	8.187469	10.931632	3.532628
N	9.942901	9.036306	4.689377
N	10.186792	8.857317	7.504284
C	8.597338	10.731153	7.763316
C	7.680660	11.610075	7.172874
C	9.858680	7.650494	4.666665
C	9.186577	9.896284	5.519603
C	5.189307	13.359610	9.184748
C	8.795863	10.748327	9.163115
C	8.295357	10.807162	4.931269
C	11.548931	8.707039	7.282600
C	5.591534	12.299483	8.334695
C	7.397481	13.617438	8.615545
C	11.429347	8.292082	3.122472
C	4.714433	11.284689	7.952536
H	5.042124	10.484884	7.286014
C	10.769312	7.147106	3.706322
C	10.892275	9.437290	3.758794
C	8.672470	14.180097	8.561021
H	9.470462	13.700801	7.991124
C	6.346938	14.207891	9.361183
C	7.488791	11.664104	5.786630
C	8.034661	10.386207	1.315428
C	9.345253	9.818733	6.912003
C	6.514595	12.529867	5.238431
C	11.348938	10.719954	3.458221
H	10.946834	11.595770	3.966259
C	3.874941	13.393350	9.660206
H	3.546786	14.202634	10.317370

C	3.413988	11.343493	8.440522
H	2.702812	10.564511	8.154683
C	6.589148	15.392209	10.063653
H	5.791047	15.862505	10.643602
C	9.753008	7.795727	8.289104
C	8.499532	11.750756	1.418496
C	11.997019	7.516694	7.907345
C	7.365840	8.641034	2.905668
H	7.204409	8.302850	3.929016
C	9.019714	6.803685	5.389948
H	8.306280	7.198180	6.112580
C	7.842059	9.924923	2.641335
C	8.466619	7.515246	8.753309
H	7.635525	8.192028	8.547461
C	12.419556	8.445359	2.148312
H	12.840692	7.568925	1.649546
C	8.572652	12.048525	2.800924
C	9.014373	13.290268	3.261214
H	9.057643	13.510097	4.329351
C	9.128204	5.435931	5.165564
H	8.493068	4.755757	5.738006
C	7.114447	7.807124	1.822664
H	6.757162	6.791434	2.008896
C	7.768685	9.530274	0.242562
H	7.917336	9.872123	-0.784869
C	8.884581	15.357744	9.268754
H	9.874807	15.819619	9.248406
C	10.855152	5.768553	3.493748
H	11.554919	5.368427	2.755854
C	2.994435	12.384290	9.285733
H	1.964137	12.399844	9.648976
C	12.423317	9.537436	6.581949
H	12.071352	10.459658	6.120169
C	12.329864	10.847278	2.481268
H	12.685241	11.846319	2.217950
C	10.844681	6.934512	8.556880
C	7.317012	8.241029	0.502144
H	7.114623	7.558175	-0.326425
C	7.857041	15.961064	10.012958
H	8.059942	16.885510	10.558791
C	8.879384	12.718769	0.484644
H	8.834049	12.499999	-0.585260
C	9.322751	13.957120	0.936175
H	9.621105	14.723710	0.217054
C	12.860140	9.724389	1.826585
H	13.627706	9.859093	1.060830
C	13.752815	9.144345	6.484852
H	14.450513	9.772341	5.925569
C	10.038500	4.918026	4.230766
H	10.101388	3.837662	4.080627
C	9.387039	14.235170	2.311528

H	9.729989	15.217371	2.646122
C	13.340719	7.146011	7.800421
H	13.698306	6.228054	8.273769
C	8.286839	6.351128	9.492289
H	7.290596	6.112486	9.872858
C	10.636841	5.771068	9.302967
H	11.469285	5.094269	9.511747
C	9.357486	5.484243	9.766626
H	9.181208	4.579070	10.352625
C	14.211130	7.958577	7.082327
H	15.261806	7.675989	6.982318

N

28

E: -370.883965796

N	-0.116608	-1.323033	-0.572189
C	-1.326697	-1.124108	0.225960
C	1.048529	-0.686334	0.012274
C	-0.147757	-1.274901	-2.032661
H	-0.939544	-0.980713	1.248396
C	-2.216586	-2.364938	0.313253
C	-2.151102	0.128211	-0.087029
H	1.024576	0.425680	-0.067823
C	2.378930	-1.183521	-0.528117
H	1.022861	-0.900989	1.092767
C	-1.226444	-2.151594	-2.653492
H	0.806970	-1.730108	-2.345568
C	-0.167603	0.127721	-2.657921
H	-2.814979	-2.529713	-0.592588
H	-2.921408	-2.260632	1.153185
H	-1.600619	-3.259038	0.485946
H	-1.521169	1.029773	-0.117667
H	-2.675070	0.044239	-1.051215
H	-2.918531	0.281375	0.687963
H	2.574714	-0.852630	-1.559072
H	2.413825	-2.283965	-0.508375
H	3.200625	-0.800535	0.095530
H	-2.237164	-1.743777	-2.504129
H	-1.196558	-3.169483	-2.240472
H	-1.061910	-2.216505	-3.739695
H	-1.141462	0.624113	-2.543767
H	0.597054	0.783140	-2.213905
H	0.045844	0.060493	-3.736280

^{2}N^{+}

28

E: -370.701042039

N	-0.171897	-1.056891	-0.609010
C	-1.372890	-0.931267	0.227073
C	1.079835	-0.923013	0.115551
C	-0.162208	-1.196001	-2.061858

H	-0.984140	-0.567117	1.190378
C	-1.996224	-2.306689	0.492395
C	-2.371668	0.101326	-0.278920
H	1.104948	0.127281	0.473599
C	2.359965	-1.278888	-0.600936
H	0.961549	-1.523601	1.035929
C	-1.261102	-2.073952	-2.638966
H	0.797028	-1.683447	-2.283847
C	-0.109763	0.201569	-2.706410
H	-2.537988	-2.694782	-0.377949
H	-2.713749	-2.193926	1.317532
H	-1.241617	-3.043338	0.803713
H	-1.906283	1.088056	-0.407632
H	-2.862122	-0.192334	-1.215295
H	-3.156289	0.202208	0.484486
H	2.564703	-0.629797	-1.463344
H	2.375986	-2.329622	-0.925681
H	3.189315	-1.145935	0.107943
H	-2.267012	-1.653103	-2.519297
H	-1.237877	-3.086570	-2.215397
H	-1.072716	-2.164683	-3.718067
H	-1.068146	0.727219	-2.613061
H	0.684275	0.826716	-2.274241
H	0.108804	0.068809	-3.775382

[2]N-TS
56
E: -741.591995224

H	0.277273	-2.828935	-0.165093
N	-0.095987	-1.525913	-0.510904
C	-1.331794	-1.210735	0.279055
C	1.045398	-0.782127	0.074122
C	-0.121485	-1.464250	-2.005140
H	-0.957047	-1.233900	1.314129
C	-2.430704	-2.260683	0.191747
C	-1.911211	0.179424	0.039761
H	0.774849	0.281554	0.166334
C	2.373930	-0.903522	-0.644594
H	1.146729	-1.161258	1.103031
C	-1.204188	-2.315427	-2.646932
H	0.842192	-1.917136	-2.285962
C	-0.147789	-0.051962	-2.588493
H	-3.047003	-2.158252	-0.709088
H	-3.099869	-2.131786	1.054548
H	-2.039871	-3.283114	0.224220
H	-1.157881	0.978218	0.061271
H	-2.454165	0.237052	-0.913095
H	-2.636068	0.394858	0.838014
H	2.391427	-0.377451	-1.607572
H	2.665656	-1.946436	-0.824354
H	3.152143	-0.449010	-0.015112

H	-2.197945	-1.855906	-2.568541
H	-1.253952	-3.327969	-2.228682
H	-0.977791	-2.409645	-3.718454
H	-1.151113	0.390083	-2.556303
H	0.544424	0.640489	-2.092283
H	0.149076	-0.107289	-3.645902
C	0.819553	-4.078956	0.078525
N	1.528301	-4.124635	1.271908
C	-0.277533	-5.091663	-0.173036
H	1.531967	-4.062218	-0.761098
C	2.985885	-3.927163	1.239022
C	0.935213	-4.462722	2.573184
H	-1.095186	-5.052986	0.557135
H	0.132340	-6.113891	-0.160383
H	-0.711583	-4.927481	-1.167797
H	3.205298	-3.585155	0.217312
C	3.717929	-5.251613	1.443050
C	3.478220	-2.855571	2.203578
C	-0.198977	-3.519292	2.950306
H	1.745338	-4.284108	3.292936
C	0.544859	-5.934504	2.728067
H	3.568351	-5.645295	2.459562
H	4.798355	-5.101351	1.303620
H	3.382809	-6.009898	0.720695
H	2.915898	-1.917761	2.098104
H	3.421631	-3.174927	3.254590
H	4.535773	-2.640517	1.993678
H	-1.058285	-3.612584	2.271878
H	0.139246	-2.472815	2.951455
H	-0.552943	-3.756772	3.963588
H	-0.403211	-6.180242	2.234382
H	1.323116	-6.603873	2.337619
H	0.422226	-6.154342	3.798668

NH^+

29

E: -371.357701562

H	0.069217	-2.426011	-0.393874
N	-0.110034	-1.428877	-0.557396
C	-1.369895	-1.150862	0.272826
C	1.068911	-0.710752	0.041907
C	-0.149357	-1.325878	-2.084347
H	-0.951310	-1.016470	1.281587
C	-2.280370	-2.365451	0.328875
C	-2.089699	0.127478	-0.102613
H	0.863446	0.365325	-0.034978
C	2.403200	-1.079885	-0.563594
H	1.055576	-0.967381	1.110241
C	-1.262070	-2.162584	-2.685369
H	0.802470	-1.801485	-2.363980
C	-0.125784	0.096305	-2.613427

H	-2.857841	-2.519212	-0.590268
H	-2.999486	-2.216592	1.146735
H	-1.725762	-3.289259	0.559543
H	-1.423866	1.000436	-0.135479
H	-2.623325	0.044210	-1.058596
H	-2.842739	0.326088	0.673086
H	2.534631	-0.710934	-1.589572
H	2.574994	-2.167929	-0.551802
H	3.194715	-0.623949	0.047891
H	-2.255228	-1.718832	-2.535574
H	-1.268361	-3.194656	-2.305222
H	-1.092462	-2.219931	-3.769745
H	-1.080957	0.618426	-2.486898
H	0.673470	0.710004	-2.176933
H	0.073206	0.040844	-3.693134

$^{2}N_{a}$

27

E: -370.235033514

N	-0.142760	-1.268962	-0.570980
C	-1.349835	-1.092544	0.235409
C	1.053711	-0.901388	0.041326
C	-0.155150	-1.267540	-2.032058
H	-0.959902	-0.878010	1.242252
C	-2.178004	-2.369775	0.378160
C	-2.213429	0.108963	-0.144041
C	2.374493	-1.223531	-0.563737
H	0.994838	-0.852009	1.131284
C	-1.256862	-2.114985	-2.652541
H	0.788648	-1.758302	-2.318784
C	-0.112059	0.136713	-2.645069
H	-2.743737	-2.615207	-0.530508
H	-2.906888	-2.252649	1.195510
H	-1.526329	-3.222293	0.618343
H	-1.610595	1.027457	-0.185686
H	-2.710480	-0.024190	-1.116450
H	-3.004365	0.253065	0.608309
H	2.559506	-0.714289	-1.526503
H	2.519980	-2.309931	-0.749742
H	3.174500	-0.905955	0.120458
H	-2.258697	-1.690981	-2.491292
H	-1.244610	-3.140607	-2.257765
H	-1.099296	-2.166869	-3.740294
H	-1.079285	0.653120	-2.566468
H	0.643720	0.752302	-2.134918
H	0.151029	0.080946	-3.713083

N_{b}^{+}

27

E: -370.140666356

N	-0.117053	-1.167567	-0.593354

C	-1.357017	-1.044446	0.248896
C	1.011846	-1.191100	0.026764
C	-0.159908	-1.221282	-2.090690
H	-0.955129	-0.791397	1.240980
C	-2.092860	-2.369597	0.390589
C	-2.244577	0.111791	-0.177113
C	2.372068	-1.280629	-0.528808
H	0.942963	-1.137485	1.120658
C	-1.292993	-2.063309	-2.649514
H	0.767552	-1.739401	-2.364867
C	-0.104885	0.185440	-2.674648
H	-2.676189	-2.637412	-0.497658
H	-2.797056	-2.276470	1.229865
H	-1.403679	-3.193185	0.626923
H	-1.690932	1.059717	-0.222723
H	-2.743263	-0.061406	-1.139475
H	-3.033202	0.225008	0.580390
H	2.451227	-1.198055	-1.616685
H	2.816727	-2.236563	-0.199338
H	2.987050	-0.496366	-0.057901
H	-2.283907	-1.620738	-2.487865
H	-1.282741	-3.087444	-2.254162
H	-1.142427	-2.130356	-3.736318
H	-1.036613	0.741960	-2.513442
H	0.727850	0.769226	-2.255945
H	0.052542	0.107937	-3.759820

$^{4}Co(OAc)_2$

76

E: -3645.17594788

Co	5.339101	11.612776	24.745016
P	5.261568	12.802214	22.599621
P	7.764290	11.334126	24.417987
C	6.748265	13.238362	20.188593
H	7.700292	13.067767	19.660486
H	6.561047	14.323567	20.197173
H	5.951442	12.759851	19.603988
C	6.833938	12.673498	21.600795
H	6.898457	11.572440	21.525758
C	8.118565	13.160640	22.290041
H	8.953773	12.635600	21.794574
H	8.272469	14.225808	22.044679
C	8.281025	13.025205	23.804966
H	7.521229	13.655616	24.303157
C	9.656154	13.510274	24.242828
H	9.793785	14.561548	23.941103
H	10.469736	12.929444	23.782045
H	9.778957	13.455082	25.332745
C	4.780713	14.553714	22.755249
C	5.696519	15.610095	22.693559
H	6.743851	15.417867	22.461041

C	5.284127	16.920094	22.934195
H	6.011734	17.733281	22.879454
C	3.953070	17.190889	23.243035
H	3.630634	18.217763	23.430649
C	3.035343	16.142379	23.317171
H	1.991691	16.343081	23.570408
C	3.444538	14.833392	23.082622
H	2.725464	14.016126	23.171177
C	4.018935	12.117114	21.444783
C	3.430044	12.887435	20.431480
H	3.680238	13.946759	20.337472
C	2.518992	12.310892	19.551360
H	2.063224	12.919600	18.766617
C	2.188529	10.960281	19.672056
H	1.471687	10.509542	18.981251
C	2.763581	10.193002	20.682283
H	2.494679	9.139786	20.792425
C	3.671823	10.765768	21.573137
H	4.087763	10.166375	22.388464
C	8.450081	10.112449	23.248217
C	7.594869	9.091857	22.808065
H	6.570230	9.049885	23.193454
C	8.048010	8.145785	21.888720
H	7.373873	7.354575	21.552035
C	9.350389	8.211080	21.397853
H	9.701524	7.471655	20.673891
C	10.208258	9.221621	21.833666
H	11.231887	9.273386	21.454833
C	9.763064	10.165448	22.756020
H	10.447063	10.943159	23.101355
C	8.674540	11.090561	25.979192
C	9.686140	10.138505	26.141512
H	9.983459	9.505157	25.303154
C	10.313485	9.987742	27.377946
H	11.100929	9.239299	27.496267
C	9.937800	10.784922	28.456753
H	10.430944	10.665100	29.424474
C	8.920301	11.727199	28.301094
H	8.609677	12.343243	29.148342
C	8.280656	11.876093	27.074207
H	7.462180	12.593624	26.958488
O	5.765043	13.453074	25.750469
C	4.519846	13.610920	25.945930
O	4.807538	9.632687	24.326621
C	4.858203	9.342572	25.569872
O	3.703181	12.763378	25.512623
O	5.158909	10.221835	26.400494
C	4.601838	7.924101	25.992978
H	5.503203	7.327536	25.781539
H	4.389549	7.872971	27.067378
H	3.775080	7.496334	25.410480

C	4.048024	14.841597	26.665464
H	2.973255	14.785775	26.873491
H	4.614156	14.966838	27.598990
H	4.255061	15.717113	26.031503

$^3Co(OAc)_2^-$

76

E: -3645.27732458

Co	5.486961	11.623102	24.609960
P	5.288404	12.746122	22.666419
P	7.711546	11.346724	24.408330
C	6.730095	13.227260	20.183596
H	7.677928	13.067283	19.641082
H	6.533019	14.311303	20.195769
H	5.928841	12.746314	19.606272
C	6.829924	12.657910	21.592645
H	6.902540	11.558642	21.509216
C	8.125901	13.143214	22.266174
H	8.957473	12.607366	21.772869
H	8.285730	14.205700	22.006245
C	8.298132	13.020162	23.781550
H	7.548785	13.663897	24.279414
C	9.681361	13.495747	24.202066
H	9.831131	14.544642	23.891649
H	10.487149	12.903306	23.740278
H	9.811529	13.445410	25.291915
C	4.818106	14.519256	22.762798
C	5.719532	15.581363	22.619090
H	6.754348	15.384330	22.338232
C	5.318096	16.899101	22.840305
H	6.041685	17.710416	22.721770
C	4.005932	17.180328	23.213176
H	3.690716	18.212251	23.389244
C	3.100883	16.128824	23.372972
H	2.073258	16.332878	23.685523
C	3.502213	14.814440	23.158915
H	2.802078	13.995123	23.334835
C	4.023235	12.121712	21.474083
C	3.449206	12.908258	20.464724
H	3.729951	13.960636	20.374481
C	2.515078	12.364267	19.586077
H	2.073058	12.990519	18.805821
C	2.143777	11.023860	19.703707
H	1.409459	10.596151	19.015074
C	2.700640	10.240175	20.712646
H	2.398710	9.195310	20.825321
C	3.629656	10.782795	21.602250
H	4.029203	10.182638	22.428757
C	8.478590	10.115884	23.268749
C	7.631156	9.085672	22.832501
H	6.598283	9.060564	23.206651

C	8.094571	8.124801	21.933113
H	7.422581	7.328086	21.602862
C	9.400592	8.184117	21.448752
H	9.758730	7.435038	20.736954
C	10.250283	9.205501	21.874598
H	11.276345	9.257811	21.499565
C	9.793359	10.160791	22.781652
H	10.472376	10.946469	23.119210
C	8.665684	11.134429	25.963869
C	9.716988	10.229166	26.145837
H	10.044571	9.600740	25.314629
C	10.346581	10.114728	27.386078
H	11.164090	9.399217	27.513966
C	9.935800	10.906255	28.456608
H	10.429871	10.816921	29.428180
C	8.877604	11.801150	28.284487
H	8.534529	12.410847	29.124921
C	8.234199	11.908933	27.054749
H	7.376254	12.579549	26.921837
O	5.750983	13.444473	25.859607
C	4.499999	13.571047	25.992336
O	4.756053	9.505090	24.181396
C	4.816587	9.275862	25.424423
O	3.675045	12.789989	25.478307
O	5.174161	10.129591	26.255870
C	4.494741	7.868062	25.897261
H	5.390175	7.239243	25.763606
H	4.226333	7.865798	26.961967
H	3.686945	7.432770	25.292252
C	4.003050	14.773472	26.776489
H	2.932710	14.681082	27.002846
H	4.582726	14.889249	27.703897
H	4.165706	15.676995	26.167540

^{3}A

69

E: -3416.65893464

Co	5.448310	11.387490	24.345629
P	5.220546	12.718878	22.541887
P	7.703076	11.472658	24.383547
C	6.634154	12.775468	19.989956
H	7.543632	12.432111	19.470248
H	6.533842	13.852650	19.788065
H	5.773899	12.267363	19.532796
C	6.743149	12.466578	21.477105
H	6.851044	11.370681	21.571003
C	8.003788	13.109325	22.078985
H	8.866857	12.632522	21.581559
H	8.036029	14.169985	21.773315
C	8.243584	13.085078	23.597612
H	7.509739	13.768637	24.062107

C	9.627535	13.622986	23.935169
H	9.738432	14.643939	23.534500
H	10.434593	13.015668	23.498096
H	9.791228	13.669013	25.020677
C	5.089641	14.509212	22.897100
C	5.504101	15.538211	22.040477
H	5.929343	15.303987	21.063437
C	5.380757	16.871850	22.427312
H	5.711178	17.664878	21.751988
C	4.835923	17.193371	23.669689
H	4.739758	18.239466	23.970396
C	4.419234	16.177110	24.528801
H	3.996394	16.422538	25.505842
C	4.551628	14.843370	24.149177
H	4.247540	14.039531	24.828480
C	3.809167	12.341451	21.441042
C	3.294434	13.235473	20.494102
H	3.721860	14.236305	20.397529
C	2.225820	12.859505	19.684137
H	1.825937	13.563732	18.950404
C	1.663852	11.588530	19.812644
H	0.824893	11.295955	19.176373
C	2.162676	10.699697	20.763327
H	1.713067	9.710661	20.879696
C	3.226633	11.074536	21.583064
H	3.594592	10.396181	22.361369
C	8.490504	10.111514	23.447613
C	7.729049	8.938776	23.325565
H	6.732589	8.899520	23.780412
C	8.227478	7.844496	22.621831
H	7.625184	6.936995	22.536225
C	9.483546	7.911941	22.020096
H	9.870859	7.056144	21.461842
C	10.243088	9.076189	22.127430
H	11.226618	9.134571	21.654776
C	9.751939	10.169297	22.839520
H	10.360150	11.071940	22.915987
C	8.494855	11.450538	26.032008
C	9.846764	11.154713	26.247453
H	10.491940	10.896211	25.404595
C	10.370405	11.170589	27.537576
H	11.425075	10.934437	27.699066
C	9.548936	11.481485	28.622293
H	9.961993	11.492140	29.634001
C	8.199443	11.761577	28.415995
H	7.548918	11.986008	29.264663
C	7.668797	11.739022	27.126598
H	6.599999	11.914974	26.961487
O	4.243756	11.333035	26.080620
C	3.837304	10.194956	25.720745
O	4.270922	9.663520	24.659748

C	2.798424	9.475325	26.538743
H	2.977908	8.392506	26.515720
H	2.786836	9.849156	27.569855
H	1.810283	9.659492	26.088943

2a

14

E: -308.270283875

C	-1.454780	-0.816186	-0.000001
H	-2.528968	-0.816219	0.000029
C	-0.241450	-0.816144	-0.000035
C	1.187830	-0.816039	-0.000078
C	1.900016	0.376861	-0.211596
C	1.900207	-2.008817	0.211398
C	3.290795	0.372517	-0.210841
H	1.347628	1.303980	-0.375973
C	3.290995	-2.004259	0.210567
H	1.347987	-2.936031	0.375809
C	3.990112	-0.815821	-0.000157
H	3.834493	1.305573	-0.376289
H	3.834829	-2.937239	0.375988
H	5.082617	-0.815727	-0.000188

^{3}B

83

E: -3724.96018608

Co	5.506774	11.170726	24.157646
P	5.352188	12.725838	22.425293
P	7.860885	11.545202	24.336628
C	6.974343	12.901403	19.976479
H	7.991008	12.726652	19.587805
H	6.713467	13.945384	19.741533
H	6.301146	12.226539	19.432138
C	6.961331	12.620042	21.470740
H	7.204892	11.551700	21.589591
C	8.051342	13.437840	22.172657
H	8.983082	13.250441	21.612036
H	7.842116	14.511914	22.027125
C	8.334227	13.227443	23.671458
H	7.609359	13.841753	24.232683
C	9.727462	13.728871	24.031100
H	9.865057	14.752521	23.646206
H	10.520787	13.100903	23.597109
H	9.880621	13.755545	25.118276
C	5.237315	14.491408	22.928834
C	5.168398	15.531668	21.992153
H	5.150869	15.307519	20.923432
C	5.103270	16.857360	22.412474
H	5.052271	17.659810	21.672502
C	5.094311	17.159534	23.774961
H	5.039253	18.200193	24.103777

C	5.144829	16.130694	24.712514
H	5.125645	16.359950	25.780799
C	5.215860	14.801254	24.294465
H	5.241203	13.990030	25.029597
C	3.870210	12.572206	21.354326
C	3.743175	13.008300	20.029247
H	4.582395	13.463139	19.507070
C	2.536776	12.857316	19.348773
H	2.458684	13.189895	18.310716
C	1.434700	12.290843	19.987532
H	0.489776	12.174521	19.450918
C	1.543709	11.884291	21.316020
H	0.684612	11.450434	21.833257
C	2.752736	12.016872	21.996790
H	2.827914	11.681769	23.038137
C	8.918362	10.290093	23.528984
C	8.769548	8.968446	23.979725
H	8.088149	8.757749	24.807606
C	9.468951	7.927675	23.377993
H	9.336894	6.905649	23.740469
C	10.323200	8.185457	22.304202
H	10.864566	7.366958	21.824277
C	10.478942	9.492015	21.847874
H	11.147758	9.704802	21.010417
C	9.784515	10.537754	22.457826
H	9.921811	11.551791	22.084293
C	8.568992	11.592184	26.027778
C	9.898686	11.237153	26.295368
H	10.542057	10.884456	25.485676
C	10.403158	11.327926	27.589881
H	11.440341	11.046932	27.788899
C	9.587135	11.777138	28.628872
H	9.984368	11.847852	29.644549
C	8.262604	12.123335	28.369322
H	7.615445	12.460581	29.182853
C	7.746983	12.026179	27.076623
H	6.694761	12.254769	26.878686
O	4.903249	11.985801	25.866974
C	3.623044	11.836450	25.943046
O	2.957104	11.277856	25.074312
C	2.974145	12.429547	27.172071
H	1.925125	12.118961	27.244942
H	3.527068	12.134811	28.075219
H	3.021350	13.527813	27.104619
C	5.394010	9.516427	22.823806
C	5.284997	9.207627	24.035332
H	5.136322	8.504593	24.845861
C	5.530189	9.391826	21.395946
C	4.417694	9.498775	20.543953
C	6.798468	9.173901	20.825751
C	4.576243	9.418895	19.163904

H	3.428630	9.655163	20.974014
C	6.947825	9.096096	19.445123
H	7.664577	9.064177	21.478213
C	5.840287	9.227039	18.606268
H	3.698989	9.513437	18.519648
H	7.941809	8.927849	19.023171
H	5.960790	9.170070	17.521823

^{3}C

84

E: -3725.41473521

Co	5.674830	11.199919	24.144389
P	5.541950	12.851539	22.478731
P	7.988309	11.537232	24.382258
C	7.037562	14.292944	20.539937
H	8.017341	14.441616	20.059530
H	6.704156	15.270904	20.918836
H	6.328970	13.978139	19.765506
C	7.168367	13.294119	21.688482
H	7.529471	12.321586	21.307505
C	8.214585	13.869115	22.662338
H	9.147817	13.916418	22.076178
H	7.949525	14.924915	22.839203
C	8.563018	13.269257	24.042236
H	7.945700	13.792290	24.793399
C	10.021547	13.546701	24.392997
H	10.246009	14.614431	24.241890
H	10.712897	12.968647	23.761400
H	10.240377	13.303897	25.441478
C	4.932622	14.493075	23.035051
C	4.116261	15.302154	22.233033
H	3.779768	14.940738	21.259237
C	3.728427	16.567646	22.669852
H	3.092351	17.186400	22.032673
C	4.156958	17.048625	23.906465
H	3.856435	18.043701	24.242314
C	4.979773	16.257014	24.707062
H	5.333268	16.632168	25.670845
C	5.361046	14.987116	24.275589
H	5.994039	14.370249	24.916256
C	4.403151	12.400839	21.124306
C	4.828956	11.981168	19.859698
H	5.889650	11.939323	19.612692
C	3.899862	11.591913	18.896488
H	4.249859	11.269794	17.913855
C	2.538293	11.603978	19.185983
H	1.812914	11.304389	18.426407
C	2.107149	11.982078	20.457027
H	1.041844	11.975199	20.699024
C	3.032548	12.368317	21.421827
H	2.690435	12.651753	22.419423

C	8.905549	10.386960	23.306264
C	8.771744	9.014107	23.568037
H	8.188507	8.680053	24.429605
C	9.370184	8.073678	22.736203
H	9.256840	7.009237	22.952504
C	10.101824	8.488227	21.622230
H	10.566081	7.749198	20.965633
C	10.240675	9.847959	21.353553
H	10.820177	10.180070	20.489207
C	9.649805	10.793979	22.192266
H	9.780710	11.853347	21.970007
C	8.655420	11.209239	26.050186
C	9.925993	10.658049	26.261670
H	10.550232	10.379848	25.409364
C	10.394382	10.459875	27.558051
H	11.385105	10.027614	27.715792
C	9.603876	10.813673	28.651707
H	9.974753	10.656465	29.667078
C	8.338211	11.360701	28.447486
H	7.714643	11.629957	29.303156
C	7.860576	11.552010	27.152204
H	6.859933	11.962287	26.992448
O	4.855289	12.296399	25.747137
C	3.735573	12.755592	25.879082
O	2.838498	12.540940	24.931683
C	3.332190	13.589870	27.049473
H	2.346742	13.294296	27.438807
H	4.084005	13.508598	27.841655
H	3.274098	14.640455	26.719875
C	5.240984	9.548825	23.041050
C	5.244476	9.317140	24.284112
H	5.209152	8.625994	25.119982
C	5.184963	9.210096	21.640592
C	3.982589	8.779386	21.060334
C	6.353631	9.244528	20.862274
C	3.958778	8.372166	19.730661
H	3.072106	8.761656	21.661724
C	6.322079	8.822415	19.537554
H	7.289094	9.579309	21.312561
C	5.126686	8.384230	18.968105
H	3.019008	8.036526	19.286625
H	7.241523	8.831845	18.947615
H	5.104299	8.051130	17.927986
H	1.994762	12.966910	25.134862

^{3}D

76

E: -3496.34541269

Co	5.502889	10.542313	23.089566
P	5.351319	12.831061	22.736293
P	5.364680	10.662788	25.416317

C	5.335295	15.159004	24.367158
H	5.904751	15.813706	25.044984
H	4.384084	14.905441	24.859043
H	5.082069	15.740626	23.469495
C	6.151999	13.916277	24.035448
H	7.088747	14.229809	23.545231
C	6.576007	13.138274	25.288360
H	7.388874	12.434893	25.035968
H	7.039759	13.869123	25.971451
C	5.464465	12.404199	26.060545
H	4.492312	12.853054	25.790881
C	5.652459	12.490397	27.568923
H	5.586293	13.539709	27.895932
H	6.640136	12.107542	27.865861
H	4.890563	11.918434	28.118107
C	3.562946	13.227103	22.747810
C	3.026606	14.267650	21.979411
H	3.677313	14.864951	21.336272
C	1.659792	14.534481	22.020448
H	1.250519	15.348268	21.417685
C	0.814485	13.765928	22.821105
H	-0.256440	13.979244	22.847218
C	1.338406	12.719242	23.578275
H	0.685872	12.102109	24.200704
C	2.704452	12.450115	23.536303
H	3.095336	11.614386	24.121806
C	5.913700	13.488471	21.131013
C	6.559543	14.719288	20.966049
H	6.779747	15.357200	21.824327
C	6.927325	15.153256	19.692731
H	7.432678	16.114338	19.574880
C	6.648513	14.368328	18.576036
H	6.937324	14.711974	17.580330
C	6.002574	13.141573	18.732280
H	5.783296	12.522059	17.859851
C	5.642402	12.701374	20.002140
H	5.141588	11.736017	20.121749
C	6.730800	9.767804	26.220335
C	6.637206	9.285813	27.533267
H	5.707705	9.403812	28.094790
C	7.724246	8.642326	28.117926
H	7.644986	8.265453	29.140040
C	8.911219	8.477765	27.402463
H	9.761300	7.971841	27.865665
C	9.009495	8.948316	26.094463
H	9.933344	8.810067	25.528679
C	7.920345	9.585072	25.502963
H	7.991139	9.931320	24.467023
C	3.845157	9.928506	26.103591
C	3.700626	8.535214	26.022451
H	4.514872	7.923728	25.625291

C	2.527261	7.924154	26.450151
H	2.430591	6.838605	26.387099
C	1.477275	8.694933	26.950415
H	0.555550	8.213959	27.285154
C	1.609723	10.079285	27.027588
H	0.794984	10.687522	27.427022
C	2.788453	10.696538	26.608951
H	2.872223	11.782322	26.685385
C	6.040701	9.259473	21.673002
C	4.947337	8.859067	22.140751
H	6.876282	9.268857	20.982563
C	3.746187	8.169715	22.520823
C	3.800095	6.807729	22.864630
C	2.519267	8.853976	22.573608
C	2.639150	6.146411	23.248518
H	4.753949	6.278591	22.821300
C	1.366549	8.182745	22.962903
H	2.475607	9.910027	22.297873
C	1.423767	6.830656	23.300401
H	2.681722	5.085539	23.504963
H	0.415577	8.718043	22.999473
H	0.515072	6.305071	23.602145

1a

19

E: -460.881348825

C	-4.873734	-0.577694	-6.454997
C	-5.218845	-0.201107	-5.006355
C	-4.982554	1.654538	-6.009884
C	-4.724262	0.588933	-7.085640
H	-5.777303	-0.929945	-4.407311
H	-5.322366	2.643233	-6.339950
O	-5.957581	0.998066	-5.208118
C	-3.021853	-0.051627	-3.458636
C	-1.928883	0.788604	-3.176823
C	-1.774451	2.004253	-3.833876
C	-2.706896	2.427929	-4.798778
H	-3.137852	-1.005150	-2.937059
H	-1.194197	0.483396	-2.427822
H	-0.919947	2.641763	-3.594461
H	-2.579967	3.385493	-5.310304
C	-3.778961	1.601875	-5.063133
C	-3.934829	0.374873	-4.400023
H	-4.702724	-1.587247	-6.827630
H	-4.400662	0.780953	-8.108244

^{3}E

95

E: -3957.24972786

Co	4.886610	9.789460	23.535198
P	4.557484	11.535358	21.974817

P	6.095390	11.183697	24.972857
C	6.425715	13.327743	20.699235
H	7.437214	13.761671	20.745835
H	5.718356	14.166367	20.622079
H	6.355233	12.734180	19.778555
C	6.161739	12.501169	21.951375
H	6.904559	11.684702	21.992367
C	6.362854	13.391937	23.188822
H	7.444032	13.605232	23.247868
H	5.885296	14.364357	22.980355
C	5.877149	12.982389	24.587215
H	4.780985	13.093570	24.600320
C	6.492488	13.902165	25.635126
H	6.240471	14.950565	25.411411
H	7.589225	13.813128	25.636854
H	6.138206	13.679860	26.651196
C	3.271934	12.796780	22.282991
C	2.993796	13.834834	21.381998
H	3.505944	13.877321	20.418624
C	2.047419	14.807186	21.698344
H	1.838031	15.610057	20.987887
C	1.369434	14.757854	22.916381
H	0.629305	15.523026	23.161254
C	1.634401	13.727602	23.817263
H	1.100111	13.679010	24.769080
C	2.577008	12.751677	23.499450
H	2.769394	11.935108	24.201322
C	4.318232	10.992346	20.249957
C	5.361566	10.305676	19.608096
H	6.324560	10.170888	20.107446
C	5.188958	9.792043	18.326261
H	6.012972	9.267502	17.837304
C	3.968907	9.950634	17.667819
H	3.834252	9.551331	16.660183
C	2.925918	10.619644	18.302816
H	1.964508	10.741185	17.798874
C	3.094912	11.134621	19.587139
H	2.259847	11.637435	20.077648
C	7.870955	11.040026	25.374462
C	8.789736	11.022621	24.314997
H	8.434011	11.051141	23.282957
C	10.158197	10.968079	24.564777
H	10.862863	10.957634	23.730251
C	10.625718	10.922750	25.878023
H	11.698682	10.872891	26.075926
C	9.719783	10.945395	26.936741
H	10.080988	10.918578	27.967224
C	8.349307	11.009720	26.690084
H	7.650960	11.038541	27.528691
C	5.208140	10.900683	26.543056
C	5.403802	9.666613	27.186067

H	6.138997	8.956073	26.800225
C	4.643014	9.322864	28.299625
H	4.811206	8.362781	28.792719
C	3.664790	10.196477	28.778761
H	3.065983	9.924483	29.650639
C	3.453788	11.414130	28.137010
H	2.688925	12.101788	28.504758
C	4.219326	11.766853	27.025122
H	4.031844	12.725985	26.539200
C	3.216119	9.095677	24.594641
C	2.660326	9.215375	23.497137
C	5.463947	8.184667	22.325987
C	5.309420	6.988117	23.281638
C	7.105310	7.966712	23.941849
C	6.619253	8.817249	22.758688
H	4.300417	6.591928	23.441923
H	7.731881	8.457032	24.694193
O	5.832972	7.578628	24.483944
C	6.457682	4.766014	22.332013
C	7.715482	4.163731	22.163640
C	8.878484	4.799407	22.590337
C	8.829355	6.062708	23.202458
H	5.549578	4.258858	21.997465
H	7.781547	3.178675	21.696421
H	9.842778	4.305279	22.452523
H	9.744841	6.555628	23.538299
C	7.588397	6.644246	23.371835
C	6.416827	6.004284	22.942231
H	5.047172	8.231823	21.319095
H	7.282544	9.427954	22.146814
C	1.820447	9.257892	22.337493
C	2.001484	8.329748	21.297712
C	0.768378	10.186433	22.259104
C	1.142993	8.329243	20.205960
H	2.810711	7.601734	21.360066
C	-0.081072	10.182325	21.157927
H	0.616823	10.901295	23.067847
C	0.101845	9.254921	20.132366
H	1.288309	7.602154	19.404627
H	-0.899621	10.903461	21.106425
H	-0.573163	9.250230	19.273553
H	3.439894	8.841409	25.621829

^{3}TS1

95

E: -3957.22152952

Co	5.127473	9.991173	23.332745
P	5.221317	11.617522	21.671648
P	6.377031	11.249694	24.856117
C	7.208236	13.287071	20.484576
H	8.231233	13.687476	20.565107

H	6.524559	14.144545	20.396102
H	7.145696	12.707428	19.554303
C	6.871483	12.459153	21.719718
H	7.561039	11.597761	21.778583
C	7.082564	13.332918	22.970585
H	8.174163	13.432494	23.091000
H	6.728369	14.347376	22.722622
C	6.482420	13.033819	24.359261
H	5.420466	13.330833	24.337768
C	7.232928	13.878853	25.387963
H	7.151422	14.945546	25.127469
H	8.300938	13.615210	25.394201
H	6.858766	13.750967	26.411450
C	4.001818	12.966832	21.568041
C	3.761645	13.648561	20.365338
H	4.270469	13.338740	19.449570
C	2.860396	14.709120	20.330227
H	2.677332	15.233140	19.389465
C	2.191372	15.100370	21.490926
H	1.482614	15.930961	21.457959
C	2.418511	14.423785	22.687301
H	1.881327	14.708053	23.594470
C	3.316157	13.358837	22.724368
H	3.453803	12.809849	23.658075
C	5.108560	10.742033	20.080586
C	6.232484	10.216187	19.428656
H	7.240574	10.453144	19.775614
C	6.081422	9.382845	18.321478
H	6.966643	8.985413	17.820093
C	4.808717	9.061351	17.853581
H	4.692679	8.410312	16.984422
C	3.684149	9.579789	18.496455
H	2.683680	9.336714	18.131342
C	3.830815	10.410126	19.603995
H	2.944792	10.808910	20.104199
C	8.085840	10.834900	25.335997
C	9.061418	10.772489	24.329403
H	8.781732	10.924107	23.284202
C	10.386863	10.496046	24.649811
H	11.139162	10.451836	23.859084
C	10.751066	10.269647	25.977924
H	11.790068	10.046425	26.229816
C	9.786265	10.328488	26.981060
H	10.067170	10.154446	28.022065
C	8.458040	10.613817	26.665997
H	7.711255	10.664344	27.460796
C	5.389305	11.178185	26.385607
C	5.182798	9.893005	26.917866
H	5.632039	9.018982	26.435165
C	4.379885	9.721001	28.041012
H	4.229196	8.719158	28.448735

C	3.755130	10.821154	28.632053
H	3.120225	10.683364	29.510001
C	3.932019	12.092172	28.090433
H	3.434106	12.954366	28.539566
C	4.743589	12.273477	26.970339
H	4.852351	13.276278	26.556316
C	3.506377	8.717937	22.740566
C	3.185397	9.758244	23.414780
C	5.351394	8.093310	22.271080
C	5.431711	6.895368	23.246550
C	7.202660	8.008847	23.703842
C	6.553397	8.834603	22.577949
H	4.489451	6.407069	23.520388
H	7.870240	8.525357	24.398474
O	6.027244	7.536214	24.371064
C	6.626273	4.791163	22.111774
C	7.897485	4.296234	21.780788
C	9.050059	5.019129	22.081780
C	8.975637	6.264208	22.725417
H	5.727350	4.217558	21.873595
H	7.985019	3.326648	21.285635
H	10.026565	4.606064	21.819128
H	9.882751	6.825584	22.962052
C	7.721077	6.738382	23.059852
C	6.562904	6.012161	22.756023
H	5.086264	7.947294	21.220950
H	7.162479	9.236721	21.766127
C	2.063180	10.550166	23.875587
C	1.052732	10.915656	22.970596
C	1.983440	10.992309	25.204435
C	-0.005192	11.716710	23.385997
H	1.113057	10.575807	21.934706
C	0.915955	11.784561	25.615771
H	2.757591	10.700856	25.913951
C	-0.076211	12.156464	24.708230
H	-0.781598	12.000077	22.671929
H	0.860486	12.113284	26.656104
H	-0.909810	12.783348	25.032708
H	3.041801	7.852840	22.277532

^{3}F

95

E: -3957.25871604

Co	5.021026	9.981616	23.299921
P	4.864978	11.804964	21.780082
P	6.615966	11.241569	24.434744
C	6.930665	12.700028	19.975665
H	8.018083	12.784383	19.822969
H	6.497050	13.692219	19.784666
H	6.535646	12.010629	19.217445
C	6.650798	12.207409	21.389513

H	7.098718	11.200153	21.484380
C	7.304169	13.112118	22.445608
H	8.394234	12.945958	22.401281
H	7.155555	14.164753	22.151758
C	6.829854	12.996432	23.899007
H	5.795597	13.377444	23.928632
C	7.694554	13.816404	24.845266
H	7.699069	14.872180	24.532575
H	8.734575	13.457685	24.843971
H	7.327954	13.772475	25.881228
C	3.987926	13.335331	22.214060
C	4.286512	14.577352	21.633968
H	5.060966	14.661343	20.870537
C	3.584811	15.715857	22.022448
H	3.824251	16.680208	21.568964
C	2.573976	15.624208	22.980240
H	2.023687	16.519424	23.278673
C	2.263571	14.390261	23.548827
H	1.465679	14.307305	24.290061
C	2.969226	13.249882	23.172112
H	2.720182	12.287242	23.623757
C	4.142377	11.231804	20.214530
C	4.535549	9.977379	19.726564
H	5.271321	9.378515	20.270321
C	3.973899	9.475436	18.556744
H	4.283850	8.497817	18.181246
C	3.006839	10.214774	17.873698
H	2.559743	9.816293	16.960291
C	2.609631	11.459366	18.359456
H	1.850817	12.037984	17.828005
C	3.174758	11.970892	19.526619
H	2.848951	12.939758	19.910422
C	8.214897	10.704838	25.094201
C	9.211640	10.365061	24.167937
H	8.997957	10.390128	23.095220
C	10.472139	9.975727	24.612056
H	11.248389	9.717128	23.888313
C	10.737789	9.905111	25.980179
H	11.723386	9.588859	26.328604
C	9.745585	10.233632	26.902860
H	9.953591	10.176792	27.973500
C	8.486196	10.639402	26.465199
H	7.712185	10.899709	27.190963
C	5.394548	11.253405	25.790748
C	5.065907	10.015426	26.379482
H	5.578465	9.094791	26.089951
C	4.064114	9.941564	27.345017
H	3.827216	8.975452	27.795394
C	3.362999	11.087018	27.717433
H	2.575051	11.025864	28.471462
C	3.666958	12.311936	27.122612

H	3.119279	13.212200	27.409820
C	4.675186	12.399271	26.167313
H	4.895231	13.370241	25.721523
C	3.714544	7.627134	22.634465
C	3.484737	8.906875	22.958725
C	5.117745	7.146402	22.627955
C	5.429022	6.132974	23.781796
C	6.791371	7.712698	24.246186
C	6.085583	8.308567	23.008689
H	4.615818	5.444570	24.040497
H	7.261662	8.414420	24.942000
O	5.726613	7.003817	24.868973
C	7.230426	4.347151	22.940081
C	8.607197	4.200266	22.723148
C	9.495129	5.238578	23.008048
C	9.035635	6.459156	23.519025
H	6.541613	3.530983	22.709029
H	8.992975	3.256176	22.331806
H	10.564220	5.092067	22.837778
H	9.732124	7.270456	23.743390
C	7.676214	6.588497	23.753347
C	6.785218	5.546883	23.468766
H	5.377662	6.687654	21.658454
H	6.793412	8.584606	22.209582
C	2.231802	9.651099	23.082617
C	1.490851	10.041109	21.952302
C	1.789290	10.058868	24.355582
C	0.342667	10.814077	22.096771
H	1.827000	9.731201	20.960975
C	0.634328	10.823499	24.493950
H	2.361630	9.760609	25.236669
C	-0.088600	11.209339	23.364321
H	-0.225069	11.107823	21.210831
H	0.296112	11.118668	25.490124
H	-0.993054	11.812549	23.470807
H	2.893371	6.931150	22.410804

^{2}G

95

E: -3957.40695851

Co	5.248237	9.971731	23.006830
P	4.637058	11.844023	21.916732
P	6.653943	11.151279	24.264658
C	6.388323	13.101063	19.947094
H	7.440201	13.252067	19.653710
H	5.920177	14.095630	19.989071
H	5.893959	12.526686	19.153286
C	6.329559	12.396318	21.296375
H	6.821496	11.410863	21.183706
C	7.130437	13.202824	22.328566
H	8.195927	13.094815	22.058793

H	6.898295	14.271606	22.176755
C	6.977838	12.942287	23.834531
H	6.035892	13.416191	24.154962
C	8.141135	13.589308	24.576771
H	8.173441	14.669561	24.360697
H	9.100053	13.153071	24.259132
H	8.081346	13.463567	25.666368
C	3.879030	13.349895	22.637034
C	3.784889	14.562819	21.939180
H	4.114605	14.622728	20.900428
C	3.253886	15.693943	22.555109
H	3.187378	16.632609	21.999753
C	2.805140	15.628140	23.874426
H	2.388592	16.516525	24.355332
C	2.886181	14.426089	24.574606
H	2.537661	14.362115	25.607932
C	3.418328	13.295192	23.958416
H	3.493626	12.360545	24.517296
C	3.714795	11.555135	20.365085
C	4.213089	10.568783	19.500975
H	5.109424	10.005645	19.775412
C	3.555093	10.276427	18.311579
H	3.954842	9.507366	17.646371
C	2.377789	10.948671	17.980323
H	1.854653	10.712341	17.050679
C	1.861335	11.904721	18.850094
H	0.924094	12.413453	18.613147
C	2.526085	12.210068	20.037443
H	2.098014	12.944263	20.721570
C	8.305688	10.556433	24.774021
C	9.215317	10.240627	23.756626
H	8.905812	10.312602	22.709757
C	10.506287	9.822885	24.069706
H	11.207608	9.577375	23.268953
C	10.894585	9.698674	25.404027
H	11.903646	9.360641	25.651073
C	9.987500	9.992813	26.421183
H	10.283963	9.884731	27.467148
C	8.698970	10.424639	26.109431
H	7.990291	10.654322	26.909180
C	5.633712	11.253776	25.789561
C	4.719434	10.207146	26.005061
H	4.705212	9.327147	25.350269
C	3.805107	10.273248	27.055439
H	3.101315	9.450716	27.200022
C	3.787943	11.378573	27.903680
H	3.067935	11.430682	28.723960
C	4.693542	12.419785	27.700589
H	4.685928	13.290689	28.360565
C	5.608063	12.359392	26.651171
H	6.290343	13.196428	26.499767

C	4.025568	7.538373	22.333247
C	3.771080	8.860736	22.400727
C	5.373306	7.053202	22.721681
C	5.344853	6.132768	23.983082
C	6.558898	7.748362	24.671827
C	6.214420	8.253815	23.246602
H	4.481084	5.460904	24.065393
H	6.827784	8.495744	25.425657
O	5.356587	7.077049	25.049375
C	7.277963	4.291954	23.799750
C	8.659488	4.129673	23.979231
C	9.452042	5.189401	24.420691
C	8.889573	6.443527	24.696736
H	6.663491	3.460988	23.442896
H	9.120692	3.159956	23.775306
H	10.525749	5.036787	24.558319
H	9.512768	7.276224	25.031290
C	7.520838	6.589518	24.540857
C	6.723124	5.524577	24.099382
H	5.864979	6.510503	21.893284
H	7.142701	8.388588	22.659366
C	2.412084	9.367442	22.133571
C	1.662090	8.961882	21.016719
C	1.824064	10.301004	23.003465
C	0.393296	9.480476	20.773955
H	2.105497	8.251284	20.315597
C	0.554741	10.821140	22.765834
H	2.380314	10.603259	23.893841
C	-0.167181	10.417284	21.642941
H	-0.159425	9.160901	19.886392
H	0.126936	11.546815	23.462943
H	-1.159634	10.829931	21.445451
H	3.251154	6.802569	22.060711

²H

96

E: -3957.89800798

Co	5.031186	10.025184	23.342713
P	4.867356	11.669250	21.701603
P	6.401513	11.253344	24.554061
C	7.007882	12.802057	20.114173
H	8.097288	12.955158	20.058478
H	6.536967	13.783058	19.957473
H	6.717472	12.145430	19.283682
C	6.642076	12.219909	21.473326
H	7.168380	11.253948	21.586767
C	7.124542	13.160693	22.589141
H	8.226307	13.102193	22.608468
H	6.890403	14.195164	22.285772
C	6.596900	13.010524	24.022400
H	5.554352	13.369553	24.023853

C	7.424026	13.842352	24.993863
H	7.424000	14.898395	24.682103
H	8.468346	13.496529	25.015510
H	7.035087	13.793881	26.020964
C	3.873995	13.173559	21.960519
C	3.878447	14.248839	21.059038
H	4.448148	14.186409	20.129894
C	3.136909	15.395283	21.332443
H	3.146394	16.228239	20.625919
C	2.380628	15.479100	22.502513
H	1.800335	16.380387	22.712963
C	2.357353	14.408750	23.394336
H	1.752160	14.459232	24.302146
C	3.098156	13.260119	23.123668
H	3.062212	12.417876	23.820300
C	4.362335	11.010833	20.080468
C	5.147369	10.006440	19.491710
H	6.079948	9.686842	19.965825
C	4.743897	9.401034	18.305655
H	5.367168	8.627225	17.851825
C	3.544064	9.778851	17.700434
H	3.225895	9.301550	16.771087
C	2.753981	10.765489	18.286024
H	1.813960	11.063938	17.816344
C	3.158602	11.381680	19.470532
H	2.532175	12.153531	19.922855
C	8.056199	10.705998	25.069033
C	8.981594	10.385989	24.065237
H	8.697231	10.454696	23.012457
C	10.267099	9.970126	24.400959
H	10.981290	9.723594	23.612207
C	10.636731	9.861833	25.741776
H	11.642507	9.528044	26.006345
C	9.722642	10.183891	26.743834
H	10.012006	10.106909	27.794179
C	8.437566	10.609837	26.412465
H	7.729990	10.865619	27.204027
C	5.344475	11.278877	26.042840
C	5.158271	10.066196	26.730868
H	5.706613	9.170074	26.431174
C	4.267623	9.989497	27.797333
H	4.141581	9.042906	28.327362
C	3.533824	11.113707	28.179084
H	2.833080	11.052465	29.014690
C	3.698558	12.314081	27.490703
H	3.128143	13.197708	27.785592
C	4.598666	12.400332	26.429707
H	4.709616	13.352683	25.908980
C	4.022196	8.436283	22.334557
C	3.360762	8.781772	23.488391
C	5.281554	7.602762	22.364469

C	5.282936	6.375945	23.326292
C	6.712366	7.658772	24.280672
C	6.293678	8.524265	23.075571
H	4.371306	5.766203	23.328773
H	7.123437	8.179533	25.152832
O	5.490591	6.994061	24.597366
C	7.008262	4.550866	22.414765
C	8.379064	4.261706	22.375462
C	9.309277	5.102944	22.987515
C	8.900478	6.261317	23.661133
H	6.287082	3.890558	21.926987
H	8.724931	3.360769	21.863714
H	10.370807	4.848052	22.946750
H	9.630034	6.921819	24.136443
C	7.542158	6.526489	23.719052
C	6.607483	5.683552	23.102875
H	5.574607	7.313453	21.346233
H	7.141036	8.868807	22.467804
C	2.218489	9.695330	23.584890
C	1.508935	10.152742	22.459904
C	1.851333	10.173576	24.854688
C	0.467809	11.060052	22.605980
H	1.767246	9.786213	21.464510
C	0.809775	11.086345	24.996945
H	2.398555	9.827243	25.734795
C	0.114735	11.530855	23.873301
H	-0.080008	11.402552	21.725328
H	0.537338	11.446040	25.991633
H	-0.707918	12.241205	23.981705
H	3.638396	8.750158	21.360262
H	3.690319	8.315273	24.426752

^{3}I

96

E: -3958.01728759

Co	5.113444	9.956342	23.278683
P	4.946244	11.577649	21.693206
P	6.275244	11.254040	24.692533
C	7.166732	12.814312	20.278764
H	8.250605	13.013334	20.314404
H	6.669057	13.774506	20.076804
H	6.974748	12.145201	19.429126
C	6.706933	12.214846	21.600370
H	7.255134	11.270166	21.764437
C	7.055191	13.176847	22.750509
H	8.154617	13.160618	22.855045
H	6.805258	14.200461	22.420579
C	6.437766	13.023667	24.149963
H	5.384755	13.340339	24.079668
C	7.166894	13.921720	25.140117
H	7.128996	14.970685	24.804162

H	8.225731	13.633312	25.224917
H	6.727493	13.873793	26.146778
C	3.923693	13.092540	21.869557
C	3.947794	14.153875	20.952686
H	4.545021	14.074642	20.041893
C	3.196138	15.304007	21.182741
H	3.224722	16.124619	20.461628
C	2.406035	15.406643	22.328705
H	1.816243	16.309298	22.506558
C	2.361599	14.351194	23.237046
H	1.728791	14.409329	24.125462
C	3.114018	13.200434	23.007055
H	3.066127	12.368439	23.715833
C	4.553528	10.974987	20.013305
C	5.384715	9.994386	19.448761
H	6.284981	9.676103	19.980078
C	5.056597	9.401490	18.233491
H	5.717439	8.643394	17.806239
C	3.881399	9.760892	17.571725
H	3.619909	9.289531	16.621478
C	3.038596	10.715508	18.136322
H	2.110854	10.994778	17.630737
C	3.370633	11.320759	19.348708
H	2.698522	12.062780	19.785288
C	7.975447	10.854945	25.238159
C	8.879865	10.435807	24.252992
H	8.524078	10.287712	23.229709
C	10.207494	10.170710	24.579572
H	10.902271	9.841057	23.803314
C	10.642072	10.307711	25.897817
H	11.680461	10.089039	26.157903
C	9.745787	10.716296	26.884744
H	10.081575	10.821372	27.919250
C	8.419619	10.994516	26.557821
H	7.722613	11.317531	27.334690
C	5.279131	11.297209	26.222625
C	5.206913	10.107300	26.968344
H	5.807615	9.242045	26.672341
C	4.351011	10.008752	28.060005
H	4.309223	9.077457	28.629722
C	3.535323	11.086114	28.414336
H	2.856689	11.004278	29.266467
C	3.583784	12.259932	27.666673
H	2.941292	13.104073	27.928624
C	4.450015	12.368018	26.578492
H	4.461352	13.294615	26.001562
C	3.979289	8.449552	22.381152
C	3.381360	8.809283	23.582171
C	5.160556	7.515883	22.259723
C	5.270123	6.424307	23.354400
C	6.949808	7.671400	23.829350

C	6.356342	8.440177	22.626403
H	4.339368	5.904950	23.616638
H	7.551082	8.243606	24.545046
O	5.774802	7.160173	24.471463
C	6.638683	4.344755	22.386410
C	7.947412	3.913133	22.126596
C	9.040261	4.722908	22.437806
C	8.859444	5.985723	23.017912
H	5.784601	3.711690	22.130956
H	8.114491	2.929691	21.680227
H	10.051587	4.362339	22.232246
H	9.719333	6.618503	23.254503
C	7.565303	6.398485	23.295855
C	6.466756	5.581784	22.983381
H	5.204887	7.067463	21.255366
H	7.087941	8.561558	21.812653
C	2.257866	9.740825	23.708906
C	1.520097	10.208076	22.603077
C	1.899426	10.220225	24.984174
C	0.479633	11.114965	22.768375
H	1.764517	9.851190	21.600311
C	0.860962	11.131787	25.145318
H	2.465198	9.880597	25.855590
C	0.141124	11.585582	24.038519
H	-0.075152	11.462160	21.892941
H	0.613642	11.490581	26.147809
H	-0.677185	12.298544	24.163200
H	3.551112	8.801785	21.438230
H	3.692073	8.302764	24.502819

^{3}TS2

104

E: -4187.08219975

Co	5.907356	9.797221	22.615495
P	6.172553	11.407843	21.016967
P	7.092808	10.912150	24.234458
C	8.155016	13.400015	20.436446
H	9.222401	13.605815	20.254114
H	7.792390	14.129585	21.176869
H	7.604938	13.599784	19.506183
C	7.978387	11.969215	20.930078
H	8.394806	11.276963	20.180826
C	8.788548	11.714037	22.209007
H	8.992797	10.637324	22.291129
H	9.770713	12.194383	22.055967
C	8.208755	12.241350	23.530508
H	7.538053	13.087654	23.305210
C	9.300896	12.712447	24.480408
H	9.829802	13.578244	24.049682
H	10.040747	11.916432	24.652436
H	8.904014	13.010945	25.462024

C	5.281119	13.015202	21.091938
C	4.857695	13.713072	19.955051
H	4.965077	13.259642	18.966925
C	4.310771	14.990217	20.073389
H	3.984145	15.523029	19.176755
C	4.193598	15.591603	21.325085
H	3.773876	16.596366	21.414737
C	4.585289	14.892644	22.465403
H	4.456683	15.333480	23.455903
C	5.100976	13.606037	22.348222
H	5.347588	13.042670	23.248652
C	5.831462	10.898419	19.280802
C	6.669871	11.131691	18.184316
H	7.610511	11.670806	18.309851
C	6.317002	10.681536	16.911097
H	6.985580	10.868848	16.067091
C	5.117099	10.001828	16.714755
H	4.842818	9.648828	15.717785
C	4.272430	9.766289	17.799709
H	3.336183	9.221353	17.658435
C	4.633268	10.203053	19.069771
H	3.992255	9.982118	19.927414
C	8.294240	9.865389	25.156615
C	8.916649	8.815572	24.467019
H	8.651850	8.624364	23.423903
C	9.867628	8.019670	25.104528
H	10.344582	7.204526	24.554611
C	10.200819	8.252996	26.437188
H	10.938887	7.622075	26.938505
C	9.583993	9.294215	27.129817
H	9.839051	9.484140	28.175374
C	8.641037	10.098267	26.493732
H	8.166953	10.911426	27.047251
C	6.192394	11.743935	25.601777
C	5.497647	10.930758	26.513739
H	5.548507	9.844504	26.400394
C	4.756741	11.498437	27.546135
H	4.229209	10.850331	28.250487
C	4.685265	12.886390	27.683979
H	4.101769	13.330065	28.494178
C	5.364658	13.700470	26.781718
H	5.317632	14.787792	26.879287
C	6.115881	13.134225	25.750603
H	6.648632	13.795894	25.065551
C	3.149038	9.717692	22.962642
C	3.118593	10.763869	23.807740
C	3.501867	8.301213	23.296930
C	3.810851	8.021690	24.790491
C	5.608014	7.302960	23.868302
C	4.823363	7.813756	22.624609
H	3.270086	8.634312	25.521943

H	6.700647	7.268715	23.780680
O	5.217284	8.258859	24.866510
C	2.811514	5.652846	25.505133
C	3.087417	4.279419	25.447164
C	4.259281	3.811421	24.852518
C	5.191818	4.699098	24.298781
H	1.886962	6.015381	25.962152
H	2.378779	3.566825	25.876187
H	4.454742	2.736504	24.823653
H	6.103858	4.324940	23.826706
C	4.925887	6.056467	24.379924
C	3.747910	6.525354	24.978222
H	2.647170	7.675702	22.985091
H	4.584358	6.941713	21.993337
C	2.551931	12.087378	23.538391
C	2.041758	12.471510	22.286444
C	2.449456	12.998201	24.600599
C	1.433100	13.707332	22.114564
H	2.127160	11.796247	21.432887
C	1.836235	14.237004	24.429556
H	2.856666	12.720454	25.575915
C	1.318826	14.595047	23.187051
H	1.049650	13.987091	21.130680
H	1.762196	14.925498	25.275318
H	0.836266	15.565433	23.048087
H	2.793469	9.857467	21.935766
H	3.466498	10.623237	24.834640
O	7.730016	8.896440	21.537914
C	7.387513	8.215712	20.556649
O	6.195994	7.788841	20.392000
H	5.589515	8.078406	21.348254
C	8.364576	7.894352	19.461811
H	8.119494	8.529231	18.595742
H	9.392260	8.098099	19.784600
H	8.252759	6.849423	19.142945

3aa
35
E: -770.456403612

C	4.150760	9.644545	22.448042
C	3.774058	10.056422	21.228898
C	4.482587	8.235642	22.803956
C	3.645846	7.754429	24.038465
C	5.624710	7.568720	24.812903
C	5.919744	8.084394	23.382786
H	2.617021	8.132027	24.075317
H	6.395137	7.774463	25.565935
O	4.416300	8.254827	25.121524
C	3.097711	5.146726	23.839285
C	3.664977	3.885417	24.065542
C	4.956383	3.762246	24.578234

C	5.722391	4.896271	24.880666
H	2.088755	5.237323	23.428992
H	3.086799	2.985527	23.842307
H	5.374016	2.767282	24.750276
H	6.737407	4.792609	25.272701
C	5.146776	6.139561	24.679745
C	3.849531	6.262974	24.165578
H	4.314895	7.577250	21.939065
C	3.427058	11.421357	20.821378
C	3.435614	12.512474	21.707350
C	3.067165	11.662516	19.486349
C	3.098399	13.788236	21.272013
H	3.709764	12.359484	22.753376
C	2.728927	12.940382	19.048284
H	3.054501	10.826332	18.781691
C	2.742992	14.010512	19.939872
H	3.111887	14.621087	21.979402
H	2.452605	13.100497	18.003263
H	2.478296	15.014822	19.601060
H	4.227620	10.357173	23.278182
H	3.715206	9.306380	20.430577
H	6.426073	9.059394	23.427327
H	6.546623	7.396536	22.799686

^{3}TS1^{enant}

95

E: -3957.22152952

Co	5.127473	9.991173	23.332745
P	5.221317	11.617522	21.671648
P	6.377031	11.249694	24.856117
C	7.208236	13.287071	20.484576
H	8.231233	13.687476	20.565107
H	6.524559	14.144545	20.396102
H	7.145696	12.707428	19.554303
C	6.871483	12.459153	21.719718
H	7.561039	11.597761	21.778583
C	7.082564	13.332918	22.970585
H	8.174163	13.432494	23.091000
H	6.728369	14.347376	22.722622
C	6.482420	13.033819	24.359261
H	5.420466	13.330833	24.337768
C	7.232928	13.878853	25.387963
H	7.151422	14.945546	25.127469
H	8.300938	13.615210	25.394201
H	6.858766	13.750967	26.411450
C	4.001818	12.966832	21.568041
C	3.761645	13.648561	20.365338
H	4.270469	13.338740	19.449570
C	2.860396	14.709120	20.330227
H	2.677332	15.233140	19.389465
C	2.191372	15.100370	21.490926

H	1.482614	15.930961	21.457959
C	2.418511	14.423785	22.687301
H	1.881327	14.708053	23.594470
C	3.316157	13.358837	22.724368
H	3.453803	12.809849	23.658075
C	5.108560	10.742033	20.080586
C	6.232484	10.216187	19.428656
H	7.240574	10.453144	19.775614
C	6.081422	9.382845	18.321478
H	6.966643	8.985413	17.820093
C	4.808717	9.061351	17.853581
H	4.692679	8.410312	16.984422
C	3.684149	9.579789	18.496455
H	2.683680	9.336714	18.131342
C	3.830815	10.410126	19.603995
H	2.944792	10.808910	20.104199
C	8.085840	10.834900	25.335997
C	9.061418	10.772489	24.329403
H	8.781732	10.924107	23.284202
C	10.386863	10.496046	24.649811
H	11.139162	10.451836	23.859084
C	10.751066	10.269647	25.977924
H	11.790068	10.046425	26.229816
C	9.786265	10.328488	26.981060
H	10.067170	10.154446	28.022065
C	8.458040	10.613817	26.665997
H	7.711255	10.664344	27.460796
C	5.389305	11.178185	26.385607
C	5.182798	9.893005	26.917866
H	5.632039	9.018982	26.435165
C	4.379885	9.721001	28.041012
H	4.229196	8.719158	28.448735
C	3.755130	10.821154	28.632053
H	3.120225	10.683364	29.510001
C	3.932019	12.092172	28.090433
H	3.434106	12.954366	28.539566
C	4.743589	12.273477	26.970339
H	4.852351	13.276278	26.556316
C	3.506377	8.717937	22.740566
C	3.185397	9.758244	23.414780
C	5.351394	8.093310	22.271080
C	5.431711	6.895368	23.246550
C	7.202660	8.008847	23.703842
C	6.553397	8.834603	22.577949
H	4.489451	6.407069	23.520388
H	7.870240	8.525357	24.398474
O	6.027244	7.536214	24.371064
C	6.626273	4.791163	22.111774
C	7.897485	4.296234	21.780788
C	9.050059	5.019129	22.081780
C	8.975637	6.264208	22.725417

H	5.727350	4.217558	21.873595
H	7.985019	3.326648	21.285635
H	10.026565	4.606064	21.819128
H	9.882751	6.825584	22.962052
C	7.721077	6.738382	23.059852
C	6.562904	6.012161	22.756023
H	5.086264	7.947294	21.220950
H	7.162479	9.236721	21.766127
C	2.063180	10.550166	23.875587
C	1.052732	10.915656	22.970596
C	1.983440	10.992309	25.204435
C	-0.005192	11.716710	23.385997
H	1.113057	10.575807	21.934706
C	0.915955	11.784561	25.615771
H	2.757591	10.700856	25.913951
C	-0.076211	12.156464	24.708230
H	-0.781598	12.000077	22.671929
H	0.860486	12.113284	26.656104
H	-0.909810	12.783348	25.032708
H	3.041801	7.852840	22.277532

TS1^{reg}

95

E: -3957.21166660

Co	4.762745	10.039665	23.349529
P	4.940308	11.601622	21.672827
P	6.240946	11.128128	24.783274
C	7.085347	12.933477	20.321273
H	8.165330	13.150079	20.325291
H	6.558678	13.897715	20.266341
H	6.858656	12.369963	19.406899
C	6.701750	12.182456	21.591566
H	7.246737	11.221432	21.622835
C	7.134211	13.025014	22.809702
H	8.229853	12.922520	22.884248
H	6.962143	14.082779	22.549641
C	6.544179	12.867068	24.222855
H	5.526533	13.291046	24.208920
C	7.410907	13.651040	25.205429
H	7.461863	14.708524	24.902953
H	8.437011	13.254095	25.216564
H	7.030907	13.611568	26.234842
C	3.923958	13.115470	21.712827
C	3.769827	13.920606	20.574397
H	4.214235	13.615923	19.624185
C	3.029888	15.098155	20.643335
H	2.913889	15.717888	19.751400
C	2.434399	15.483410	21.844764
H	1.852888	16.406718	21.894535
C	2.571161	14.682941	22.977269
H	2.093267	14.973178	23.915632

C	3.308187	13.502053	22.909992
H	3.396137	12.866590	23.794424
C	4.536811	10.821130	20.079121
C	5.486687	10.118882	19.323674
H	6.542839	10.135376	19.602044
C	5.098193	9.394025	18.198574
H	5.850061	8.857598	17.615592
C	3.758578	9.358015	17.815040
H	3.457331	8.793623	16.929886
C	2.806458	10.050189	18.563274
H	1.754855	10.029589	18.267945
C	3.189204	10.772227	19.691103
H	2.433291	11.302803	20.275325
C	7.891437	10.500571	25.220812
C	8.792928	10.233706	24.179305
H	8.478033	10.350548	23.138888
C	10.086606	9.805167	24.461797
H	10.783049	9.602271	23.645169
C	10.488559	9.628653	25.786194
H	11.501399	9.285991	26.008833
C	9.595531	9.888285	26.823904
H	9.907753	9.752484	27.861685
C	8.301574	10.326972	26.546867
H	7.610773	10.535935	27.366285
C	5.273093	11.217215	26.321543
C	4.981449	9.990572	26.942303
H	5.377411	9.058649	26.527338
C	4.163659	9.954440	28.066812
H	3.950983	8.997683	28.548930
C	3.604853	11.131595	28.567280
H	2.958423	11.100178	29.447122
C	3.864950	12.345504	27.936068
H	3.422701	13.268203	28.318312
C	4.696319	12.392326	26.816980
H	4.885192	13.355048	26.339214
C	2.790253	9.079713	23.082891
C	2.900736	10.182081	23.747638
C	4.390297	8.106306	22.437835
C	4.471570	6.999502	23.515765
C	6.471348	7.775539	23.483082
C	5.755142	8.631252	22.421840
H	3.538229	6.729474	24.019913
H	7.352664	8.202993	23.970843
O	5.397690	7.605788	24.417647
C	4.998800	4.624170	22.423761
C	6.058503	3.862123	21.908574
C	7.360012	4.360798	21.892247
C	7.650893	5.640365	22.389310
H	3.979474	4.230992	22.431692
H	5.861991	2.860702	21.519265
H	8.166662	3.742590	21.491832

H	8.673756	6.024424	22.373965
C	6.603977	6.380018	22.908110
C	5.297260	5.877166	22.925210
H	3.939405	7.889863	21.468552
H	6.234493	8.824688	21.460064
H	2.200913	10.879887	24.199826
C	1.718400	8.155371	22.701974
C	0.883535	7.647721	23.709165
C	1.478998	7.792801	21.368928
C	-0.165441	6.790641	23.387167
H	1.068991	7.930475	24.747968
C	0.424004	6.942102	21.052604
H	2.103375	8.201612	20.570897
C	-0.396619	6.434156	22.059500
H	-0.805770	6.397752	24.180079
H	0.239082	6.676099	20.009448
H	-1.219213	5.761049	21.808210

TS1^{dia}

95

E: -3957.21271628

Co	4.652149	10.377501	23.470545
P	5.094222	11.906081	21.667374
P	6.109514	11.288221	25.044832
C	6.319745	14.439800	21.294028
H	7.051834	15.168942	21.675083
H	5.365387	14.965550	21.143864
H	6.669109	14.092473	20.310848
C	6.162758	13.296550	22.286613
H	7.149103	12.831762	22.453890
C	5.612696	13.759548	23.645211
H	5.735719	14.853075	23.713728
H	4.523290	13.595060	23.700563
C	6.297141	13.165684	24.878714
H	5.731061	13.526039	25.752663
C	7.738518	13.639426	25.020922
H	8.214813	13.264314	25.937191
H	7.762006	14.739855	25.051081
H	8.371266	13.310486	24.183514
C	3.683584	12.755918	20.878607
C	3.735974	13.202328	19.549506
H	4.615662	12.998792	18.935448
C	2.664354	13.903480	19.003698
H	2.713425	14.243132	17.966689
C	1.534281	14.174677	19.776045
H	0.695809	14.724714	19.342796
C	1.477071	13.739107	21.098004
H	0.595851	13.942220	21.710659
C	2.544139	13.029263	21.644945
H	2.479443	12.682492	22.675709
C	6.002439	11.174431	20.265275

C	7.387428	11.311190	20.104154
H	7.963395	11.966732	20.760004
C	8.052300	10.622012	19.089729
H	9.131180	10.744899	18.971044
C	7.344713	9.789222	18.226489
H	7.867169	9.250523	17.433120
C	5.964151	9.651369	18.374779
H	5.402094	9.005652	17.696513
C	5.298403	10.335655	19.386693
H	4.214582	10.229219	19.487504
C	7.810100	10.624222	24.997257
C	8.428351	10.462470	23.750676
H	7.884042	10.700167	22.834903
C	9.728682	9.973166	23.664643
H	10.197473	9.849425	22.685700
C	10.423473	9.630699	24.824815
H	11.440976	9.239336	24.759031
C	9.812218	9.783591	26.067950
H	10.349486	9.511705	26.979288
C	8.512097	10.279797	26.157436
H	8.039149	10.381819	27.136121
C	5.525476	11.114179	26.762945
C	4.481088	10.225430	27.033810
H	4.060330	9.615537	26.231430
C	3.964457	10.119981	28.323634
H	3.144621	9.425796	28.518730
C	4.489694	10.895764	29.353082
H	4.081472	10.815140	30.363090
C	5.536781	11.782284	29.093592
H	5.952062	12.392377	29.898728
C	6.050312	11.895689	27.805566
H	6.863021	12.601566	27.618215
C	2.825064	9.361696	23.394087
C	2.788449	10.501175	23.986742
C	5.763954	8.924292	22.604360
C	6.391956	7.713752	23.330823
C	4.396624	7.070321	22.974787
C	4.417561	8.506585	22.377451
H	7.478672	7.625174	23.232464
H	3.640257	6.401041	22.547577
O	5.714243	6.647631	22.645680
C	6.329135	7.589355	26.003245
C	5.490763	7.211117	27.061261
C	4.170862	6.827730	26.830872
C	3.646280	6.794005	25.530063
H	7.367232	7.870389	26.186553
H	5.880376	7.208674	28.081545
H	3.543179	6.524636	27.672053
H	2.620621	6.460815	25.354434
C	4.488704	7.139631	24.487891
C	5.810241	7.547642	24.721093

H	6.336334	9.359843	21.785334
H	3.948702	8.671299	21.404007
C	1.904807	11.435967	24.658126
C	0.515422	11.340585	24.461315
C	2.402609	12.458985	25.478722
C	-0.345398	12.246388	25.070622
H	0.119456	10.554833	23.814681
C	1.537989	13.356821	26.096012
H	3.476915	12.533718	25.647135
C	0.162790	13.255522	25.890577
H	-1.422321	12.164155	24.907487
H	1.939967	14.141351	26.740672
H	-0.516148	13.964165	26.370040
H	2.198275	8.508689	23.153128

A-TS-H

98

E: -3787.99681715

Co	9.946561	14.098611	34.024269
P	10.124026	12.336878	32.731628
P	12.037722	14.649718	33.761410
C	11.974467	10.519479	31.549960
H	13.037691	10.240373	31.486600
H	11.569089	10.508451	30.528901
H	11.459509	9.738661	32.127005
C	11.842634	11.881528	32.220118
H	12.281605	11.784006	33.229017
C	12.637387	12.967180	31.494229
H	13.689728	12.637101	31.511385
H	12.368322	12.976880	30.424553
C	12.573428	14.427291	31.986667
H	11.704459	14.892927	31.494874
C	13.811310	15.194571	31.540566
H	13.953542	15.057964	30.456928
H	14.724925	14.835578	32.037683
H	13.721444	16.272139	31.731666
C	8.945565	12.154892	31.365776
C	9.333729	12.102853	30.022899
H	10.389557	12.087068	29.750288
C	8.369859	12.093715	29.015342
H	8.680721	12.051841	27.969089
C	7.015463	12.137206	29.341209
H	6.262547	12.129554	28.549961
C	6.622586	12.188466	30.680001
H	5.561967	12.223392	30.938830
C	7.579831	12.202084	31.690412
H	7.272973	12.267312	32.737776
C	9.740068	10.973082	33.886724
C	8.931877	9.882726	33.549958
H	8.477435	9.818889	32.559178
C	8.705930	8.869698	34.480748

H	8.075074	8.019876	34.210690
C	9.283334	8.935905	35.747655
H	9.102405	8.139395	36.472864
C	10.095363	10.018176	36.086077
H	10.557143	10.073548	37.074433
C	10.321475	11.033108	35.161099
H	10.970613	11.870809	35.434540
C	13.092204	13.654775	34.873632
C	12.692595	13.626699	36.219593
H	11.807441	14.189441	36.534147
C	13.420704	12.892531	37.151178
H	13.105164	12.877934	38.196864
C	14.553197	12.181735	36.751336
H	15.123763	11.604770	37.482416
C	14.961058	12.215949	35.419496
H	15.853445	11.669780	35.105830
C	14.236476	12.951896	34.481977
H	14.575306	12.974593	33.445105
C	12.488670	16.336609	34.296029
C	13.833176	16.723826	34.395101
H	14.630283	16.019317	34.149211
C	14.161723	18.002953	34.832060
H	15.210600	18.299499	34.901481
C	13.154761	18.898760	35.195480
H	13.416002	19.900682	35.543675
C	11.819749	18.505453	35.137424
H	11.030827	19.193106	35.450494
C	11.486265	17.227140	34.693733
H	10.446111	16.901934	34.683647
O	9.445175	15.068269	35.661093
C	8.369519	14.390777	35.679201
O	8.139061	13.663416	34.667067
C	7.449747	14.416054	36.848401
H	7.732816	13.596317	37.527462
H	7.542576	15.363684	37.392353
H	6.416044	14.242903	36.524551
H	9.549453	15.133569	32.937961
N	8.689718	16.127300	31.921517
C	9.227391	17.504070	31.999958
C	8.908187	15.405529	30.662417
C	7.338412	15.894916	32.503095
H	9.265829	17.732345	33.074610
C	8.377529	18.599579	31.351543
C	10.658172	17.577192	31.490151
H	9.969306	15.113012	30.621295
C	8.542841	16.052241	29.332467
H	8.354841	14.466032	30.761873
C	7.126621	16.678580	33.789977
H	7.356997	14.831163	32.786680
C	6.153951	16.066140	31.554113
H	8.296901	18.487279	30.263180

H	8.853141	19.572241	31.548088
H	7.363582	18.647081	31.766315
H	11.293966	16.825018	31.968256
H	10.726533	17.458064	30.399672
H	11.083112	18.558296	31.743508
H	9.191963	16.899453	29.075095
H	7.502571	16.397520	29.292456
H	8.668871	15.294805	28.543791
H	6.914090	17.742066	33.610798
H	7.992672	16.610461	34.460199
H	6.258182	16.261142	34.319292
H	6.054761	17.087006	31.160744
H	6.199952	15.367996	30.707351
H	5.231462	15.834986	32.107727

A-H
70
E: -3417.10961723

Co	5.588813	11.772904	24.392651
P	5.509069	12.997496	22.591679
P	7.743277	11.602976	24.338427
C	6.869183	13.562106	20.165484
H	7.768926	13.354874	19.565853
H	6.721376	14.650812	20.175248
H	6.011433	13.109206	19.649231
C	7.045768	12.982504	21.564917
H	7.159088	11.888585	21.450586
C	8.314436	13.495637	22.251152
H	9.160309	13.126973	21.647378
H	8.367548	14.593003	22.164934
C	8.571313	13.146294	23.727037
H	8.038461	13.890183	24.347192
C	10.054972	13.233106	24.068552
H	10.450482	14.208315	23.745216
H	10.642647	12.452454	23.562393
H	10.231084	13.139090	25.148336
C	4.893531	14.668932	22.903045
C	5.580576	15.824422	22.513235
H	6.518416	15.757182	21.961698
C	5.065732	17.080702	22.828884
H	5.604798	17.978965	22.520465
C	3.867011	17.189214	23.530320
H	3.464033	18.175195	23.772194
C	3.183845	16.039161	23.928562
H	2.247919	16.123002	24.485160
C	3.694494	14.780870	23.626699
H	3.170332	13.880290	23.958329
C	4.312394	12.136215	21.532491
C	3.235216	12.779621	20.916715
H	3.081589	13.851680	21.057898
C	2.357588	12.046323	20.120061

H	1.517861	12.550113	19.636442
C	2.548644	10.677270	19.938972
H	1.856255	10.108117	19.314780
C	3.621668	10.032286	20.554836
H	3.772067	8.959647	20.415169
C	4.501516	10.757499	21.352338
H	5.344051	10.242618	21.828844
C	8.153862	10.157778	23.319432
C	7.410038	8.995282	23.586345
H	6.684182	8.988023	24.406670
C	7.609861	7.848304	22.822973
H	7.033478	6.945931	23.038321
C	8.547419	7.851705	21.789580
H	8.703211	6.951759	21.190585
C	9.291846	9.000552	21.525315
H	10.033150	9.000114	20.723275
C	9.099160	10.152120	22.286814
H	9.695041	11.040618	22.073886
C	8.458389	11.284089	25.972164
C	9.533568	10.399333	26.123346
H	9.922591	9.852694	25.261076
C	10.105198	10.212786	27.379264
H	10.942198	9.521023	27.496009
C	9.609520	10.905191	28.483521
H	10.059320	10.755373	29.467569
C	8.534358	11.781170	28.335920
H	8.138277	12.314036	29.202971
C	7.953349	11.967907	27.084944
H	7.095343	12.635345	26.976691
O	5.154642	10.601498	25.899886
C	3.925530	10.886210	25.732217
O	3.657559	11.676192	24.774743
C	2.868965	10.349573	26.628137
H	1.911652	10.286274	26.096737
H	3.167572	9.374106	27.030994
H	2.748651	11.045857	27.473226
H	5.889053	12.969983	25.082525

[3]B-TS-H

112

E: -4096.28544617

Co	4.328250	-8.314092	14.363340
P	3.951094	-9.761819	16.237548
P	6.493910	-9.158647	13.855190
C	5.868051	-10.289787	18.372059
H	6.941644	-10.270456	18.617181
H	5.491737	-11.289006	18.640647
H	5.378819	-9.538237	19.002804
C	5.692065	-9.988848	16.890647
H	6.128226	-8.988223	16.716904
C	6.468287	-11.015502	16.057809

H	7.494177	-11.025482	16.461403
H	6.068629	-12.020568	16.275266
C	6.544707	-10.895024	14.527451
H	5.593322	-11.275568	14.112826
C	7.666652	-11.763656	13.969798
H	7.569981	-12.786777	14.365576
H	8.661913	-11.389304	14.253217
H	7.628719	-11.822774	12.874054
C	3.352883	-11.426097	15.781388
C	3.326380	-12.495735	16.687581
H	3.656259	-12.357358	17.718740
C	2.873131	-13.748041	16.282800
H	2.856759	-14.575243	16.995952
C	2.437524	-13.945540	14.971862
H	2.080633	-14.929052	14.657908
C	2.454918	-12.886875	14.066893
H	2.110638	-13.035756	13.040992
C	2.910209	-11.630986	14.467263
H	2.920561	-10.802826	13.751924
C	2.828885	-9.179322	17.554154
C	2.731101	-9.766660	18.824242
H	3.356370	-10.614874	19.099375
C	1.825517	-9.273604	19.759084
H	1.763386	-9.736092	20.746759
C	0.990749	-8.204249	19.432551
H	0.275555	-7.825753	20.166676
C	1.059070	-7.638044	18.161990
H	0.391597	-6.817350	17.888785
C	1.974831	-8.117120	17.225646
H	2.014425	-7.676330	16.226501
C	7.926250	-8.198890	14.439944
C	8.054689	-6.894691	13.936699
H	7.324026	-6.516681	13.216990
C	9.107584	-6.082065	14.343877
H	9.201442	-5.071901	13.939030
C	10.040446	-6.555389	15.269353
H	10.865570	-5.916355	15.591424
C	9.918794	-7.847666	15.774273
H	10.649853	-8.227525	16.491501
C	8.869153	-8.668600	15.359674
H	8.795859	-9.678676	15.760767
C	6.824595	-9.368918	12.074622
C	8.135938	-9.365350	11.577507
H	8.978080	-9.183292	12.249184
C	8.368101	-9.593094	10.224383
H	9.391384	-9.589076	9.842163
C	7.297858	-9.827382	9.360236
H	7.483148	-10.005766	8.298496
C	5.993684	-9.824685	9.850664
H	5.153490	-9.995325	9.173787
C	5.749884	-9.591339	11.203652

H	4.722445	-9.554932	11.574199
O	3.192448	-8.692947	12.787558
C	2.128304	-8.053867	13.124599
O	2.108758	-7.415213	14.187646
C	0.936878	-8.140193	12.220031
H	0.206943	-7.359909	12.464000
H	1.247046	-8.071798	11.168914
H	0.462836	-9.124316	12.359881
C	4.951134	-5.891415	15.951045
C	4.702979	-6.394125	14.771900
H	4.412349	-5.832999	13.873565
C	5.477099	-6.320404	17.247587
C	4.651930	-6.394546	18.383356
C	6.853466	-6.563198	17.401033
C	5.184510	-6.725839	19.626251
H	3.581394	-6.213598	18.286920
C	7.381265	-6.890083	18.647075
H	7.508264	-6.486328	16.533244
C	6.550777	-6.972007	19.765066
H	4.522157	-6.794424	20.492233
H	8.453472	-7.077290	18.742608
H	6.967248	-7.224971	20.742674
H	4.641138	-4.654607	16.004438
N	4.430218	-3.246446	16.182367
C	3.359383	-3.141958	17.213762
C	5.737031	-2.867186	16.754258
C	4.216994	-2.748188	14.798889
H	3.873494	-3.497334	18.121167
C	2.179538	-4.086889	16.999859
C	2.883088	-1.720661	17.491162
H	5.719076	-1.807982	17.065153
C	6.933407	-3.129692	15.860166
H	5.846974	-3.460401	17.674877
C	2.916043	-3.224065	14.168732
H	5.031438	-3.228805	14.231398
C	4.383044	-1.239466	14.630599
H	1.424006	-3.679517	16.317251
H	1.686216	-4.247713	17.969731
H	2.493693	-5.066625	16.616122
H	3.713716	-1.019882	17.655319
H	2.255794	-1.334569	16.675191
H	2.267327	-1.721207	18.402517
H	6.978559	-2.457980	14.991331
H	6.949246	-4.169010	15.500441
H	7.849628	-2.968307	16.445847
H	2.044536	-2.682789	14.561675
H	2.745351	-4.301013	14.299333
H	2.960678	-3.018869	13.089312
H	3.547273	-0.678281	15.068079
H	5.316053	-0.861195	15.070327
H	4.410538	-1.004243	13.556544

B-H
84
E: -3725.42951206

Co	5.629192	11.585557	24.376000
P	5.547325	12.712304	22.540616
P	7.823863	11.342572	24.390558
C	7.073095	13.543960	20.343639
H	8.040499	13.493972	19.820682
H	6.828941	14.605669	20.496071
H	6.309985	13.113705	19.682251
C	7.177648	12.815218	21.681005
H	7.448145	11.758398	21.509236
C	8.271561	13.471846	22.533770
H	9.204373	13.368689	21.955136
H	8.079532	14.557095	22.567786
C	8.537839	12.997914	23.975242
H	7.925518	13.615439	24.656912
C	9.997091	13.180088	24.377668
H	10.307196	14.218166	24.181546
H	10.668748	12.517784	23.811720
H	10.148619	12.980177	25.447049
C	5.006467	14.443916	22.692892
C	4.187999	15.012252	21.705186
H	3.802769	14.398009	20.889330
C	3.863936	16.365615	21.759630
H	3.223696	16.797655	20.987481
C	4.354450	17.164157	22.791526
H	4.096348	18.224755	22.832161
C	5.174893	16.606758	23.771357
H	5.561690	17.228259	24.581978
C	5.503062	15.253782	23.725038
H	6.118305	14.822702	24.515248
C	4.377814	11.893979	21.421238
C	3.008972	11.984981	21.707779
H	2.661712	12.614059	22.529171
C	2.095175	11.235155	20.974657
H	1.030231	11.306609	21.205552
C	2.536002	10.384830	19.961068
H	1.816386	9.790451	19.394063
C	3.896869	10.287270	19.677737
H	4.246744	9.618528	18.888621
C	4.817926	11.035992	20.405211
H	5.881071	10.926634	20.182972
C	8.578150	10.036414	23.376731
C	8.211535	8.708401	23.651060
H	7.505705	8.489808	24.456294
C	8.736370	7.664636	22.896270
H	8.439757	6.637278	23.118364
C	9.631184	7.931675	21.858771
H	10.041910	7.111486	21.265792

C	10.004804	9.245098	21.584614
H	10.713012	9.458295	20.781066
C	9.483236	10.294841	22.340852
H	9.797997	11.314647	22.118042
C	8.411489	10.998419	26.081744
C	9.502336	10.146767	26.308097
H	9.990806	9.640211	25.473839
C	9.971699	9.946704	27.603416
H	10.822227	9.282634	27.772219
C	9.361708	10.591577	28.678744
H	9.731788	10.429966	29.693671
C	8.280257	11.442352	28.456678
H	7.800583	11.949749	29.296537
C	7.801460	11.649128	27.164898
H	6.955489	12.319192	26.998999
O	5.408557	13.045715	25.764633
C	4.153598	12.925759	25.626354
O	3.754725	12.060032	24.788573
C	3.198184	13.795644	26.361638
H	2.220946	13.307634	26.456886
H	3.602712	14.068649	27.343955
H	3.071023	14.720668	25.775741
C	5.381324	10.024485	23.413938
C	4.339852	9.264945	23.808336
H	6.066418	9.660793	22.641405
C	4.055255	7.895450	23.370939
C	4.841673	7.221143	22.419282
C	2.947030	7.225049	23.912803
C	4.524995	5.927101	22.026021
H	5.709280	7.717546	21.979222
C	2.630702	5.927903	23.519738
H	2.326475	7.736564	24.653430
C	3.418862	5.273667	22.574763
H	5.144243	5.418977	21.282847
H	1.763677	5.424386	23.953262
H	3.173289	4.255643	22.264278
H	3.618849	9.684663	24.52102

16. References

[1] Takizawa, K.; Sekino, T.; Sato, S.; Yoshino, T.; Kojima, M.; Matsunaga, S. Cobalt-Catalyzed Allylic Alkylation Enabled by Organophotoredox Catalysis. *Angew. Chem. Int. Ed.* **2019**, *58*, 9199–9203.

[2] Roy, S.; Das, S. K.; Chattopadhyay, B. Cobalt(II)-based Metalloradical Activation of 2-(Diazomethyl)pyridines for Radical Transannulation and Cyclopropanation. *Angew. Chem. Int. Ed.* **2018**, *57*, 2238-2243.

[3] Murugesan, V.; Syam, A.; Anantharaj, G. V.; Rasappan, R. Alkenylation of Unactivated Alkanes: Synthesis of Z-Alkenes via Dual Co-TBADT Catalysis. *Chem. Commun.* **2024**, *60*, 14049-14052.

[4] Jordan, R. S.; Li, Y. L.; Lin, C. -W.; McCurdy, R. D.; Brosmer, J. L.; Marsh, K. L.; Khan, S. I.; Houk, K. N.; Kaner, R. B.; Rubin, Y. Synthesis of N = 8 Armchair Graphene Nanoribbons from Four Distinct Polydiacetylenes. *J. Am. Chem. Soc.* **2017**, *139*, 15878-15890.

[5] Dutta, U.; Lupton, D. W.; Maiti, D. Aryl Nitriles from Alkynes Using tert-Butyl Nitrite: Metal-Free Approach to C≡C Bond Cleavage. *Org. Lett.* **2016**, *18*, 860-863.

[6] Kosuge, S.; Araki, Y.; Tsuge, K.; Sugimoto, K.; Matsuya, Y. One-Pot Synthesis of Pentasubstituted Pyridines following the Gold(I)-Catalyzed Aza–Enyne Metathesis/6π-Electrocyclization–Aromatization Sequence. *J. Org. Chem.* **2023**, *88*, 6973-6986.

[7] Bergstrom, B. D.; Toth-Williams, G.; Lo, A.; Toman, J. W.; Fettinger, J. C.; Shaw, J. T. One-Pot Assembly and Synthetic Applications of Geminal Acyl/Alkoxy Tetrasubstituted Allenes. *J. Org. Chem.* **2022**, *87*, 12175-12181.

[8] Kawanishi, S.; Oki, S.; Kundu, D.; Akai, S. Lipase/Oxovanadium Co-Catalyzed Dynamic Kinetic Resolution of Propargyl Alcohols: Competition between Racemization and Rearrangement. *Org. Lett.* **2019**, *21*, 2978–2982.

[9] An, T.; Ryu, H.; Choi, T. -L. Living Alternating Ring-Opening Metathesis Copolymerization of 2,3-Dihydrofuran to Provide Completely Degradable Polymers. *Angew. Chem. Int. Ed.* **2023**, *62*, e202309632.

[10] M. J. Frisch, G. W. Trucks, H. B. Schlegel, G. E. Scuseria, M. A. Robb, J. R. Cheeseman, G. Scalmani, V. Barone, G. A. Petersson, H. Nakatsuji, X. Li, M. Caricato, A. V. Marenich, J. Bloino, B. G. Janesko, R. Gomperts, B. Mennucci, H. P. Hratchian, J. V. Ortiz, A. F. Izmaylov, J. L. Sonnenberg, D. Williams-Young, F. Ding, F. Lipparini, F. Egidi, J. Goings, B. Peng, A. Petrone, T. Henderson, D. Ranasinghe, V. G. Zakrzewski, J. Gao, N. Rega, G. Zheng, W. Liang, M. Hada, M. Ehara, K. Toyota, R. Fukuda, J. Hasegawa, M. Ishida, T. Nakajima, Y. Honda, O. Kitao, H. Nakai, T. Vreven, K. Throssell, J. A. Montgomery, Jr., J. E. Peralta, F. Ogliaro, M. J. Bearpark, J. J. Heyd, E. N. Brothers, K. N. Kudin, V. N. Staroverov, T. A. Keith, R. Kobayashi, J. Normand, K. Raghavachari, A. P. Rendell, J. C. Burant, S. S. Iyengar, J. Tomasi, M. Cossi, J. M. Millam, M. Klene, C. Adamo, R. Cammi, J. W. Ochterski, R. L. Martin, K. Morokuma, O. Farkas, J. B. Foresman, D. J. Fox, *Gaussian 16*, *Revision C.02*, Gaussian, Inc., Wallingford CT, **2019**.

[11] C. Adamo, V. Barone, *J. Chem. Phys*. **1999**, *110*, 6158.

[12] a) S. Grimme, J. Antony, S. Ehrlich, H. Krieg, *J. Chem. Phys*. **2010**, *132*, 154104; b) S. Grimme, S. Ehrlich and L. Goerigk, *J. Comp. Chem*. **2011**, *32*, 1456.

[13] a) F. Weigend, R. Ahlrichs, *Phys. Chem. Chem. Phys*. **2005**, *7*, 3297; b) F. Weigend, *Phys. Chem. Chem. Phys*. **2006**, *8*, 1057.

[14] T. A. Halgren, W. N. Lipscomb, *Chem. Phys. Lett*. **1977**, *49*, 225.

[15] a) H. P. Hratchian, H. B. Schlegel, *J. Chem. Phys*. **2004**, *120*, 9918; b) K. Fukui, *Acc. Chem. Res*. **1981**, *14*, 363.

[16] Y. Zhao, D. G. Truhlar, *Theor. Chem. Acc*. **2008**, *120*, 215.

[17] A. V. Marenich, C. J. Cramer, D. G. Truhlar, *J. Phys. Chem. B* **2009**, *113*, 6378.

[18] a) C. P. Kelly, C. J. Cramer, D. G. Truhlar, *J. Chem. Theory Comput*. **2005**, *1*, 1133; b) C. P. Kelly, C. J. Cramer, D. G. Truhlar, *J. Phys. Chem. B* **2006**, *110*, 16066.

[19] a) L. Falivene, R. Credendino, A. Poater, A. Petta, L. Serra, R. Oliva, V. Scarano, L. Cavallo, *Organometallics* **2016**, *35*, 2286; b) L. Falivene, Z. Cao, A. Petta, L. Serra, A. Poater, R. Oliva, V. Scarano, L. Cavallo, *Nat. Chem*. **2019**, *11*, 872; c) B. Maity, C. Zhu, M. Rueping, L. Cavallo, *ACS Catal.* **2021**, *11*, 13973.

[20] a) F. M. Bickelhaupt, K. N. Houk, Angew. Chem. Int. Ed. 2017, 56, 10070; b) I. Fernández, F. M. Bickelhaupt, *Chem. Soc. Rev*. **2014**, *43*, 4953.

[21] a) R. A. Marcus, *J. chem. Phys*. **1956**, *24*, 966; b) N. S. Hush, *Trans. Faraday Soc*. **1961**, *57*, 557.

[22] S. Pradhan, D. Satav, S. Dutta, B. Maity, L. Cavallo, B. Sundararaju, *Nat. Commun*., **2024**, *15*, 1042.

****

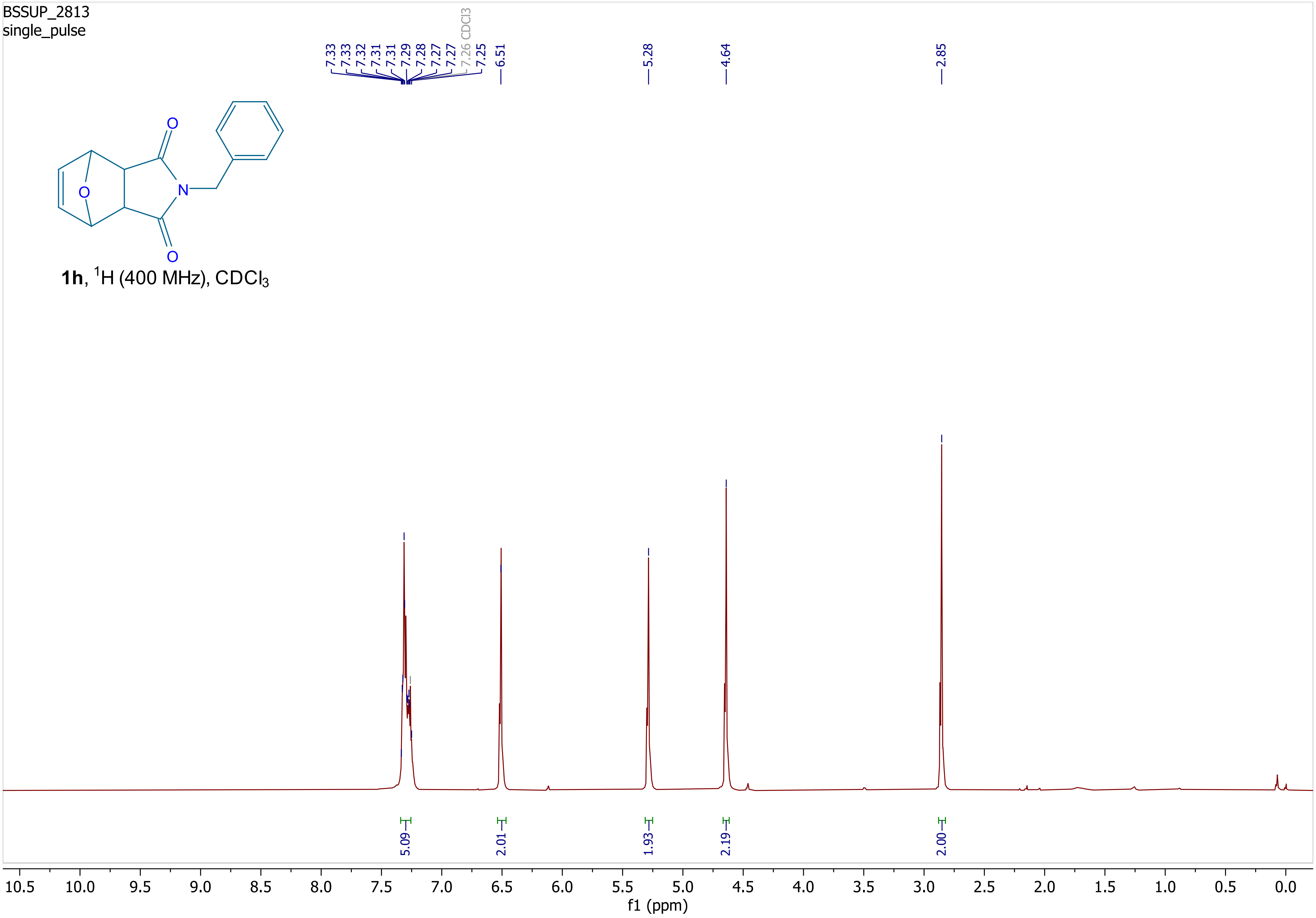
BSSUP_2813
single_pulse
7.33
7.33
7.32
7.31
7.31
7.29
7.28
7.27
7.27
7.26 CDCl3
7.25
6.51
5.28
4.64
2.85
O
O
N
O
1h, ^{1}H (400 MHz), $CDCl_3$
5.09
2.01
1.93
2.19
2.00
10.5
10.0
9.5
9.0
8.5
8.0
7.5
7.0
6.5
6.0
5.5
5.0
4.5
4.0
3.5
3.0
2.5
2.0
1.5
1.0
0.5
0.0
f1 (ppm)

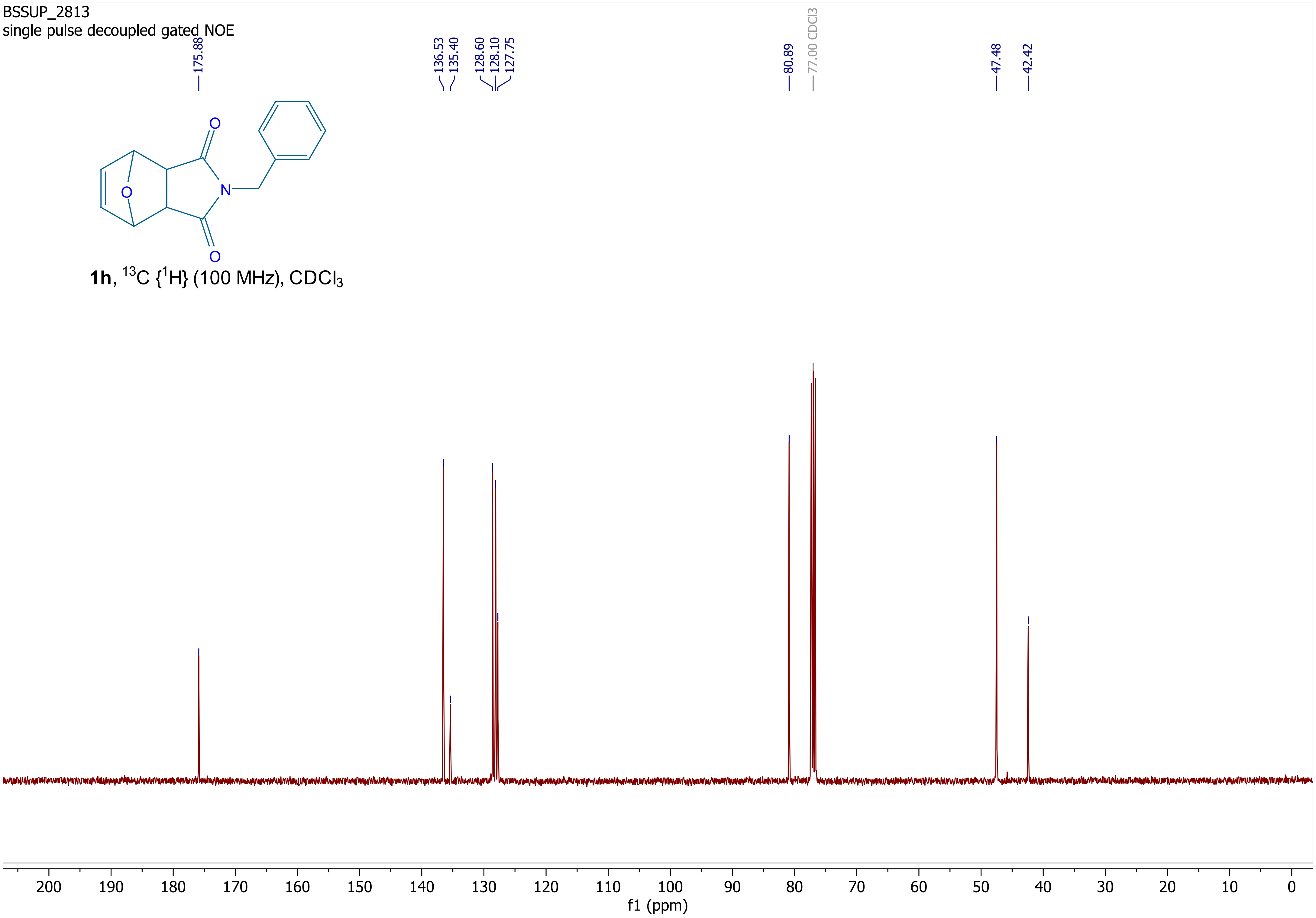

BSSUP_2813
single pulse decoupled gated NOE
175.88
136.53
135.40
128.60
128.10
127.75
80.89
77.00 CDCl3
47.48
42.42
O
O
O
N
1h, $^{13}C\ \{^{1}H\}$ (100 MHz), $CDCl_3$
200 190 180 170 160 150 140 130 120 110 100 90 80 70 60 50 40 30 20 10 0
f1 (ppm)

S#365320

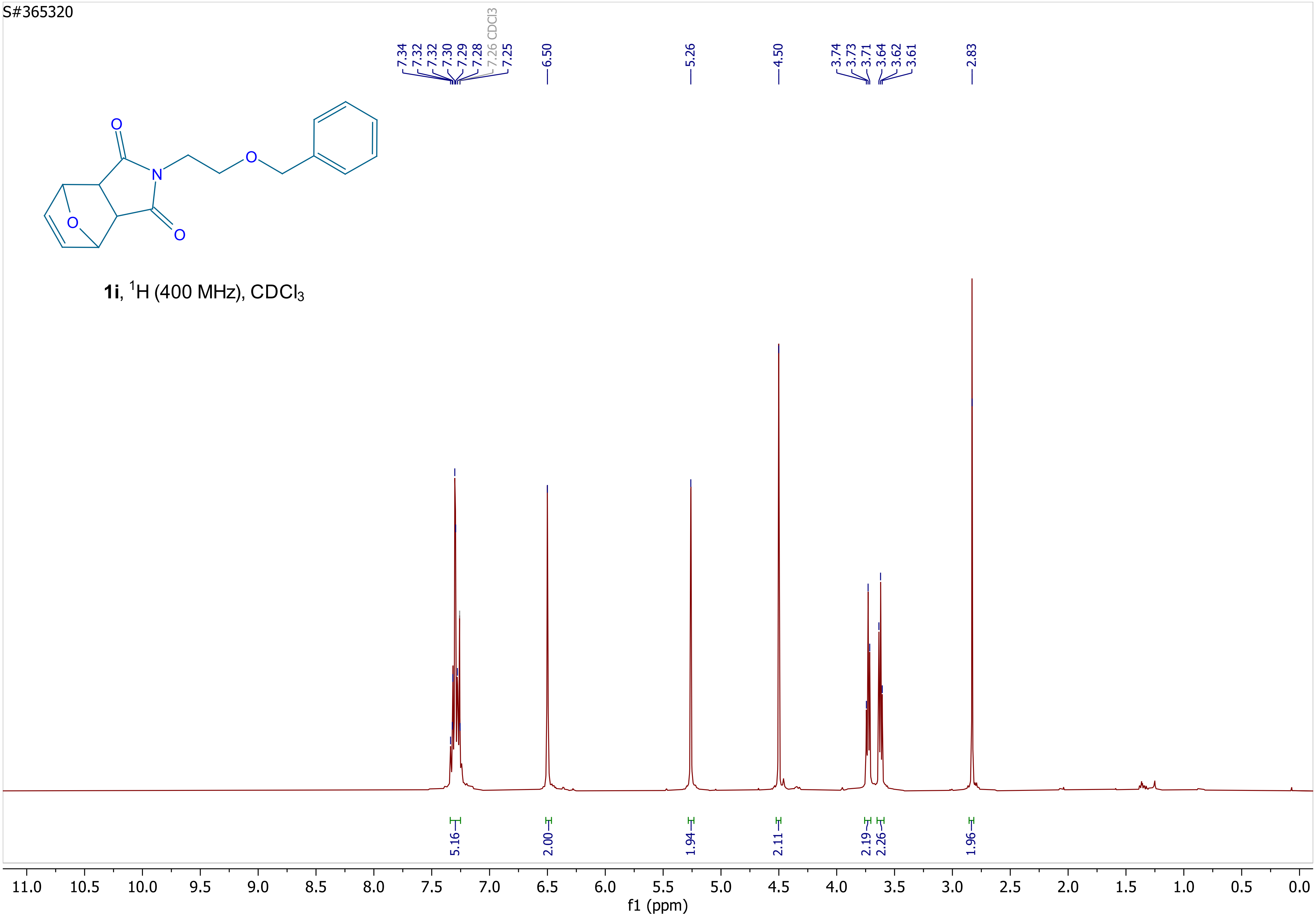

1i, ^{1}H (400 MHz), $CDCl_3$

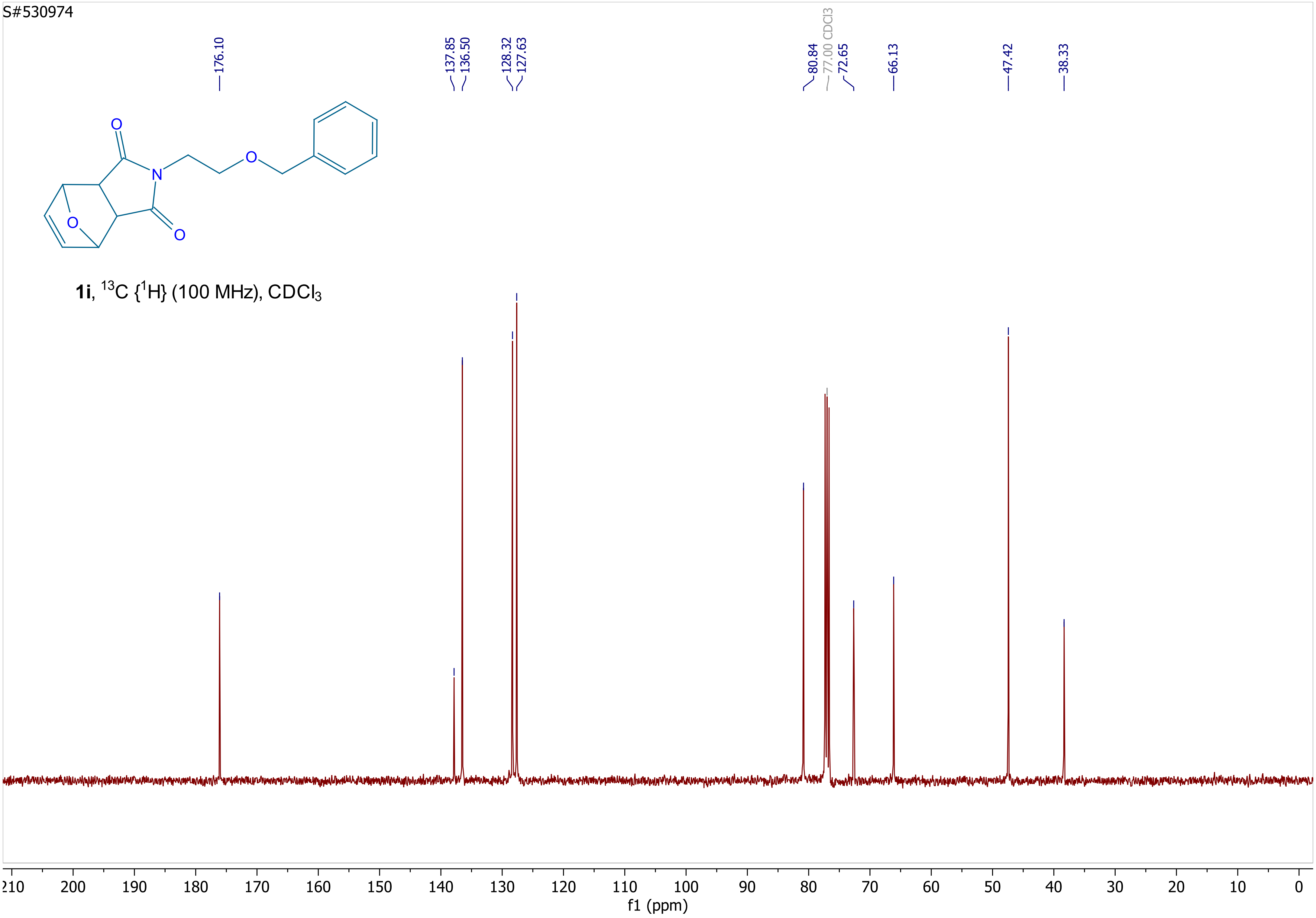
S#530974
176.10
137.85
136.50
128.32
127.63
80.84
77.00 CDCl3
72.65
66.13
47.42
38.33
O
N
O
O
O
1i, 13C {1H} (100 MHz), CDCl3
210 200 190 180 170 160 150 140 130 120 110 100 90 80 70 60 50 40 30 20 10 0
f1 (ppm)

BSSUP1808B

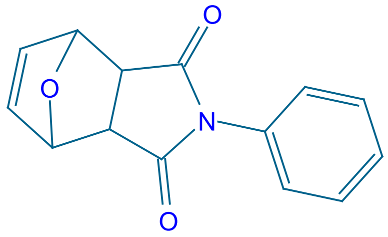

1j, ^{1}H (400 MHz), $CDCl_3$

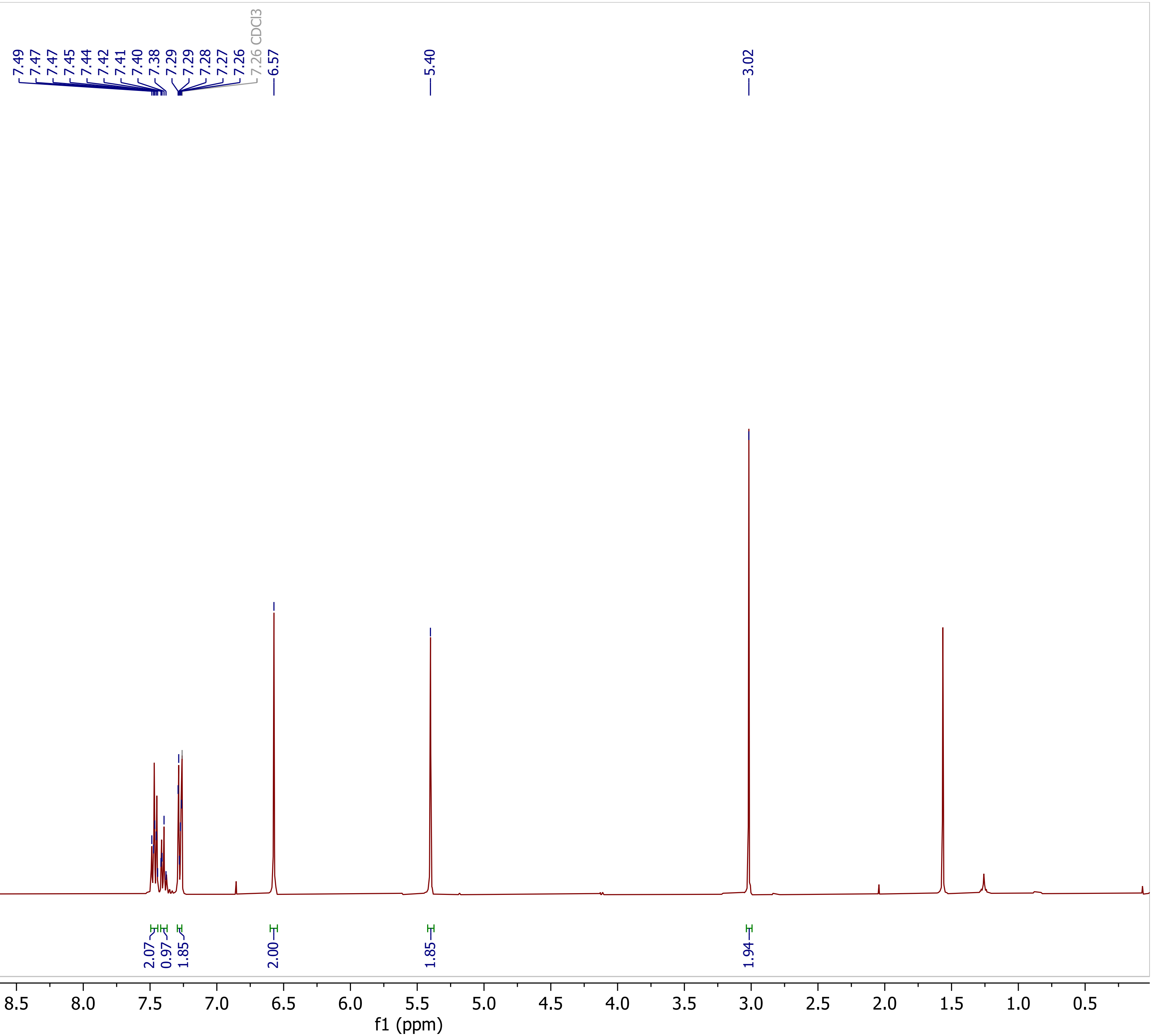

BS/BSARS_PH

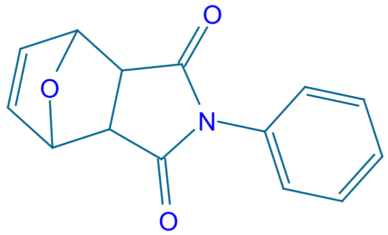

1j, $^{13}C\{^{1}H\}$ (100 MHz), $CDCl_3$

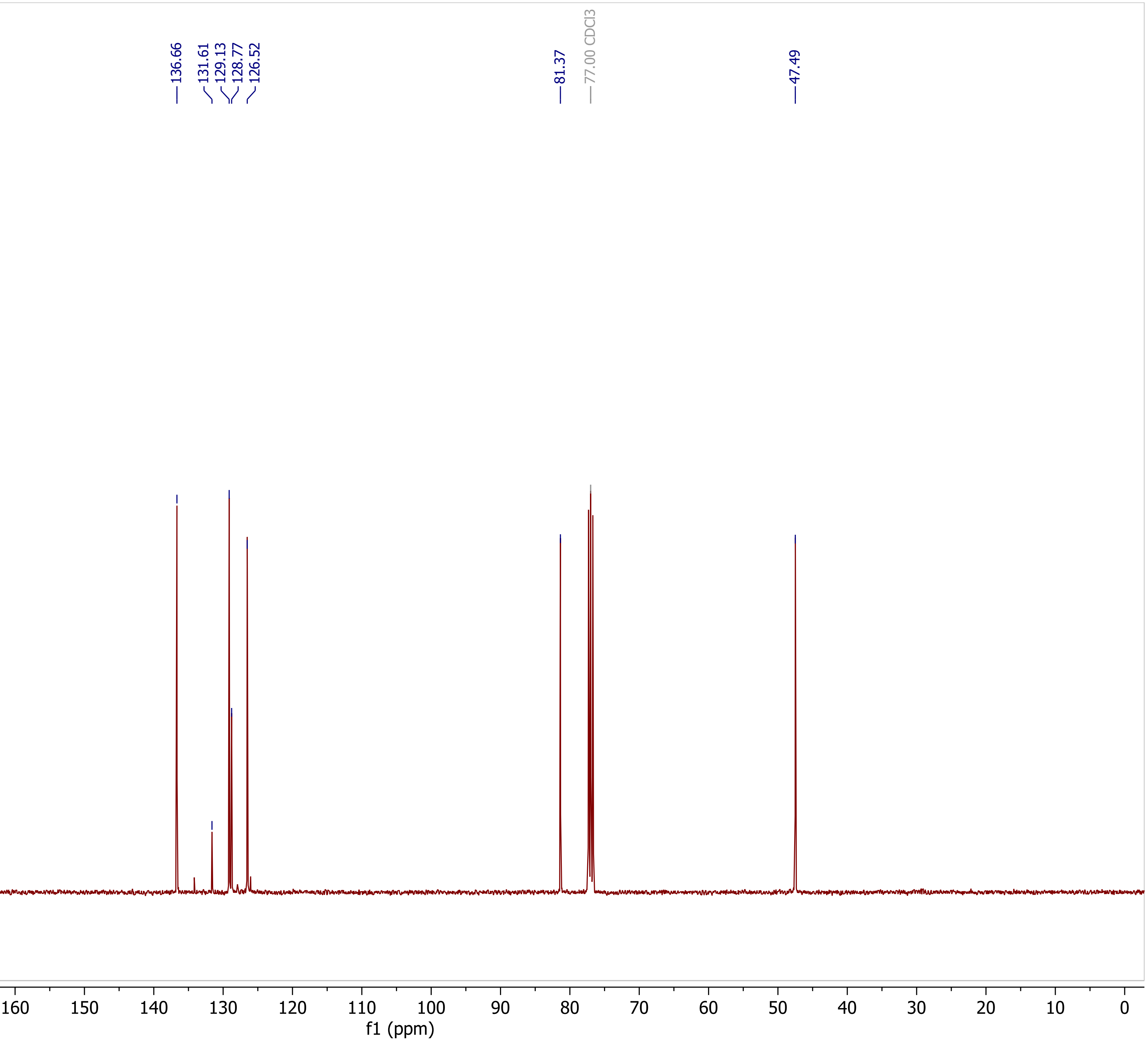

S#361181

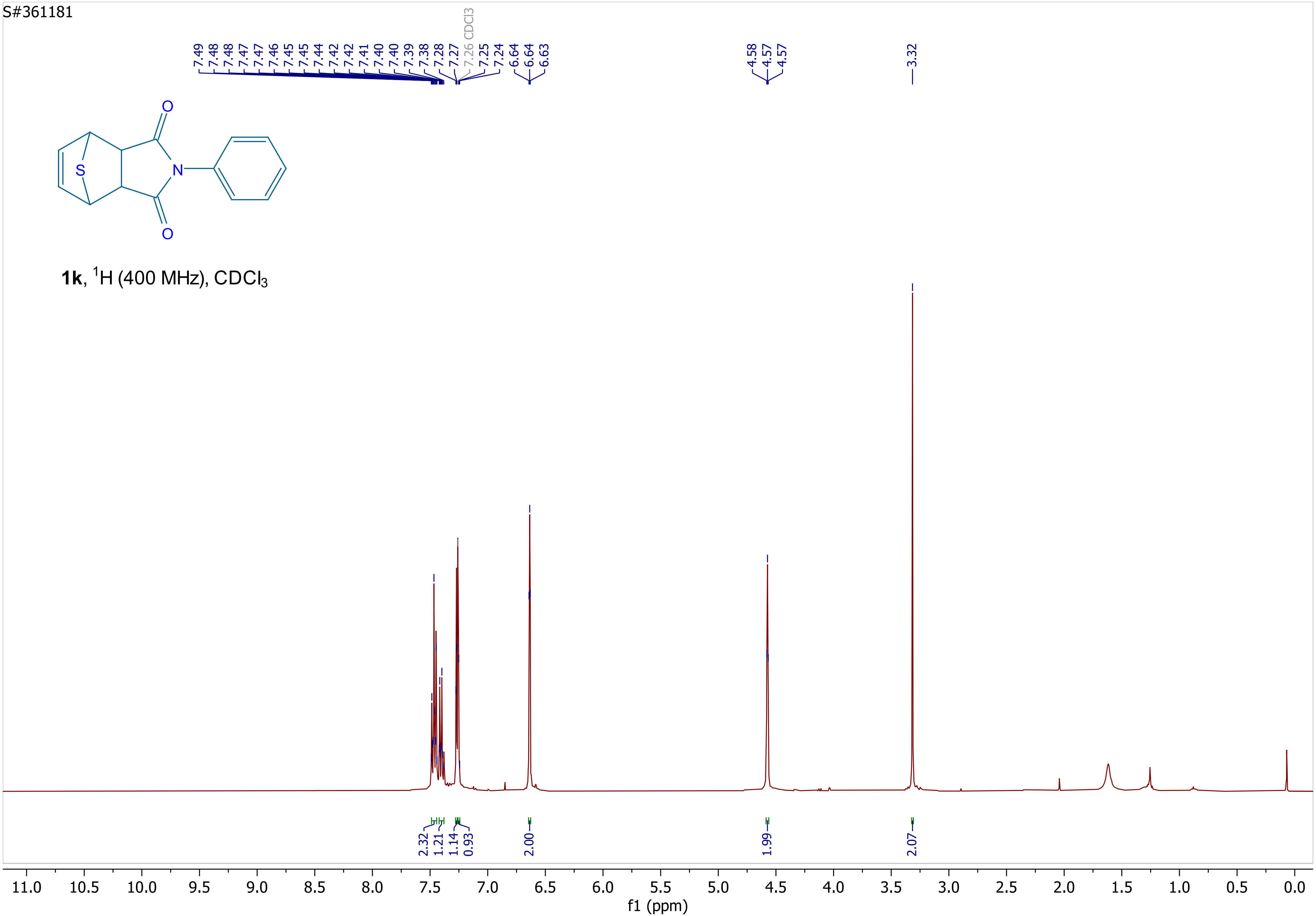

1k, ^{1}H (400 MHz), CDCl$_3$
7.49
7.48
7.48
7.47
7.47
7.46
7.45
7.45
7.44
7.42
7.42
7.41
7.40
7.40
7.39
7.38
7.28
7.27
7.26 CDCl3
7.25
7.24
6.64
6.64
6.63
4.58
4.57
4.57
3.32
2.32
1.21
1.14
0.93
2.00
1.99
2.07
11.0 10.5 10.0 9.5 9.0 8.5 8.0 7.5 7.0 6.5 6.0 5.5 5.0 4.5 4.0 3.5 3.0 2.5 2.0 1.5 1.0 0.5 0.0
f1 (ppm)

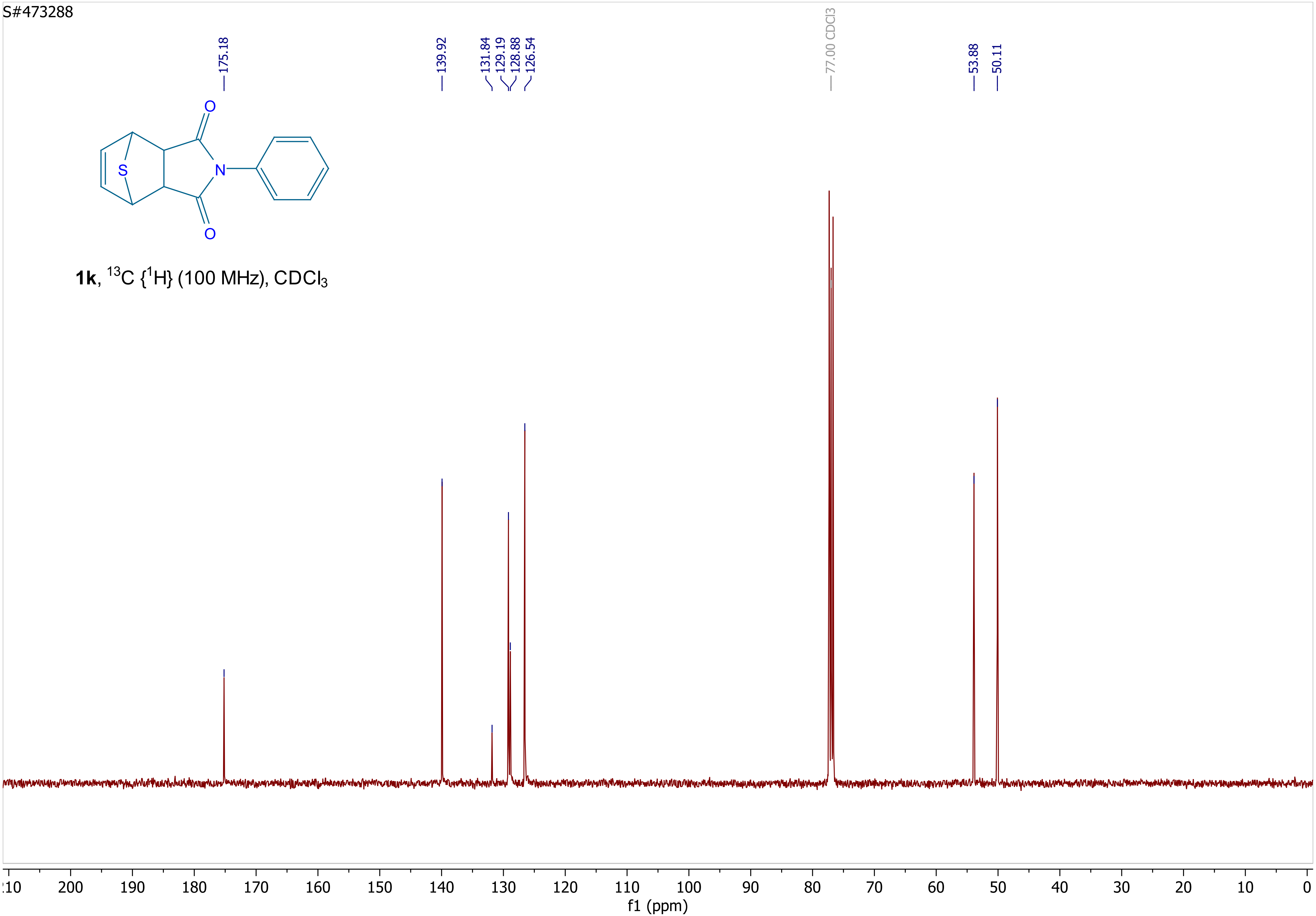
S#473288
175.18
139.92
131.84
129.19
128.88
126.54
77.00 CDCl3
53.88
50.11
O
S
N
O
1k, $^{13}C\ \{^1H\}$ (100 MHz), $CDCl_3$
210
200
190
180
170
160
150
140
130
120
110
100
90
80
70
60
50
40
30
20
10
0
f1 (ppm)

S#352669

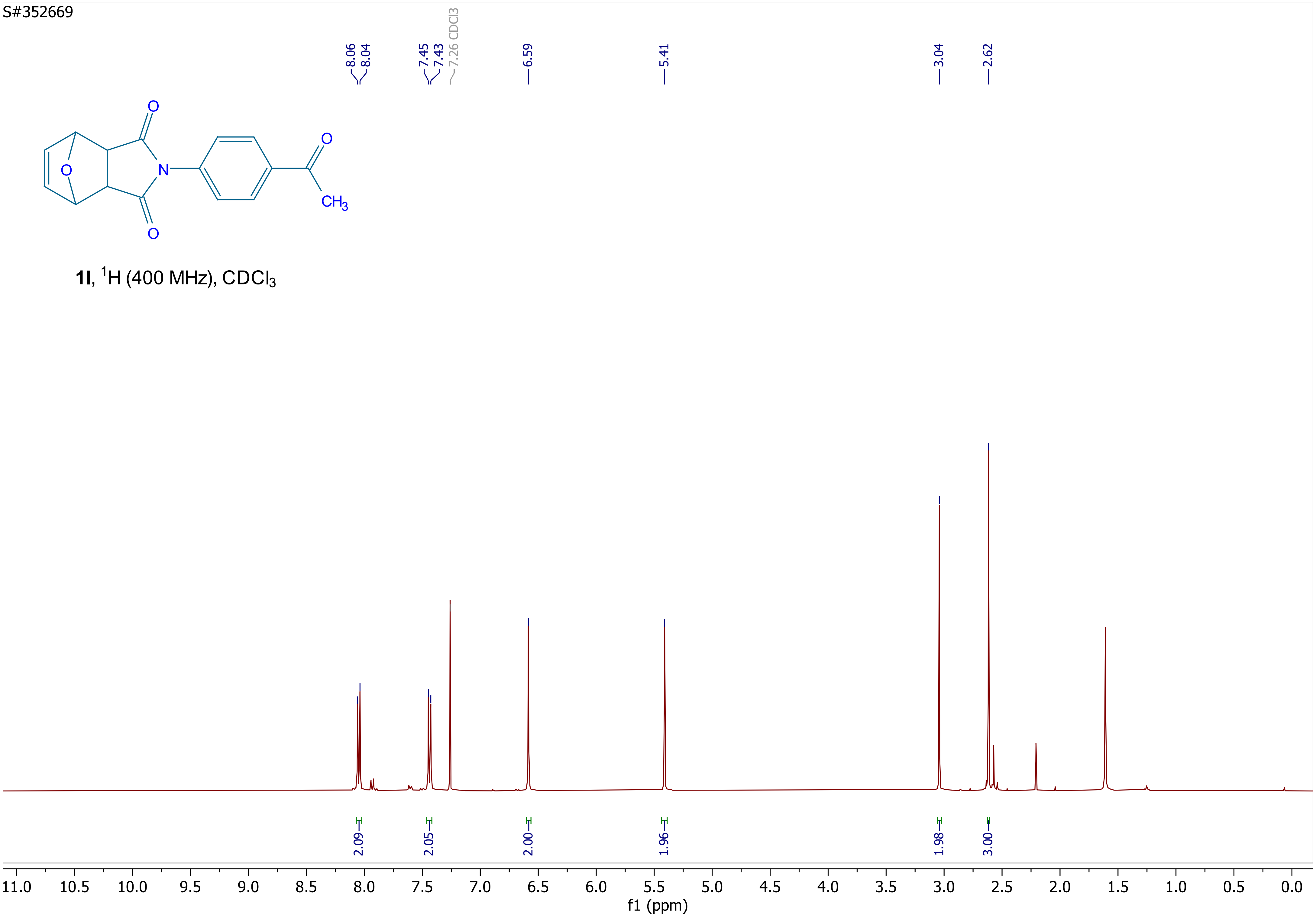

1l, ^{1}H (400 MHz), $CDCl_3$

S#502148

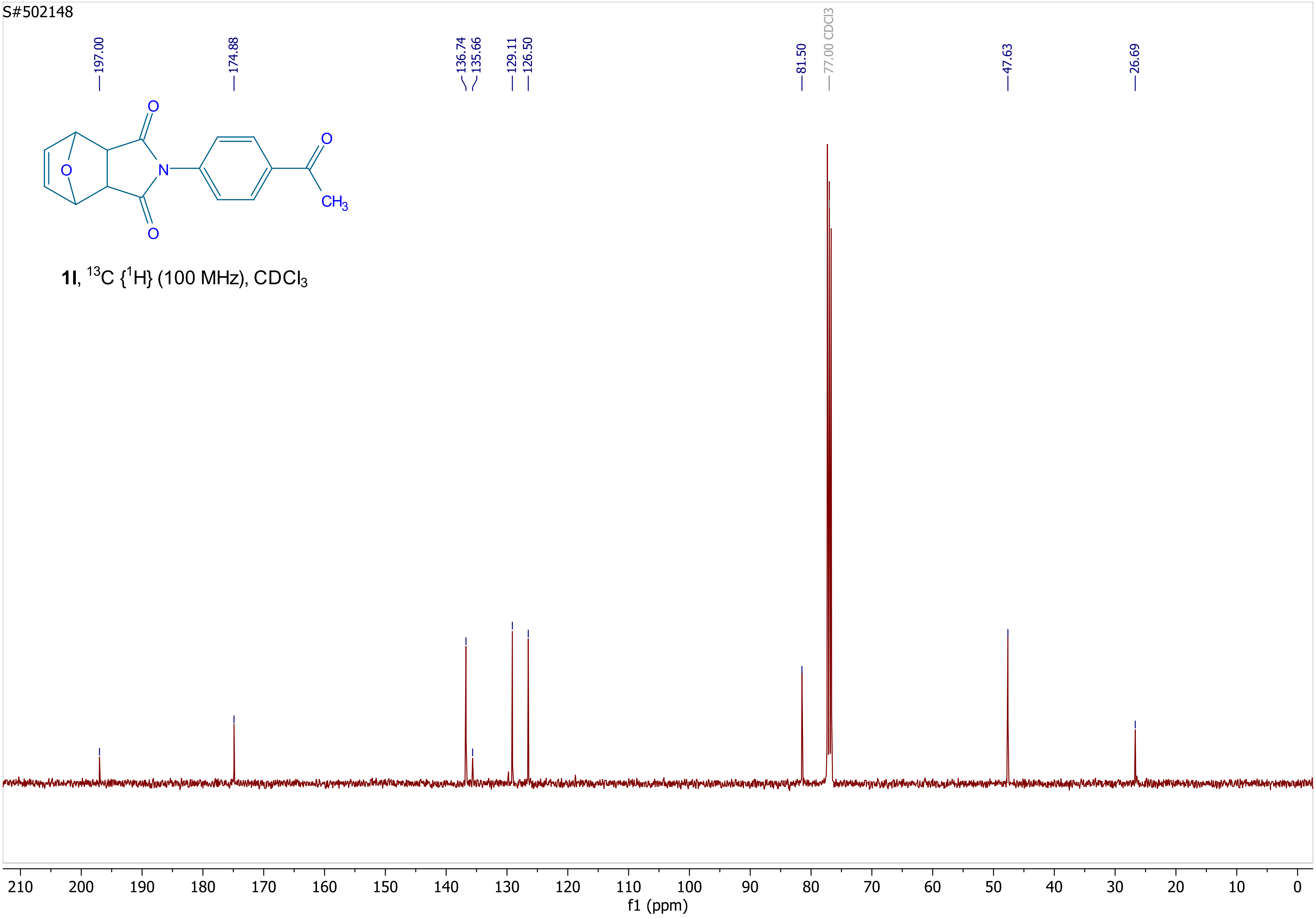

1l, $^{13}C\{^1H\}$ (100 MHz), $CDCl_3$

S#356925

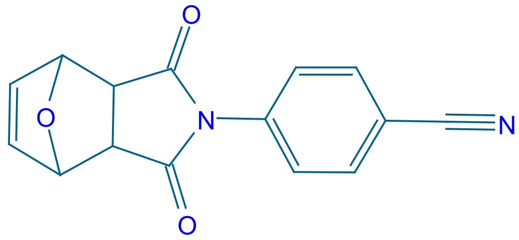

1m, ^{1}H (400 MHz), $CDCl_3$

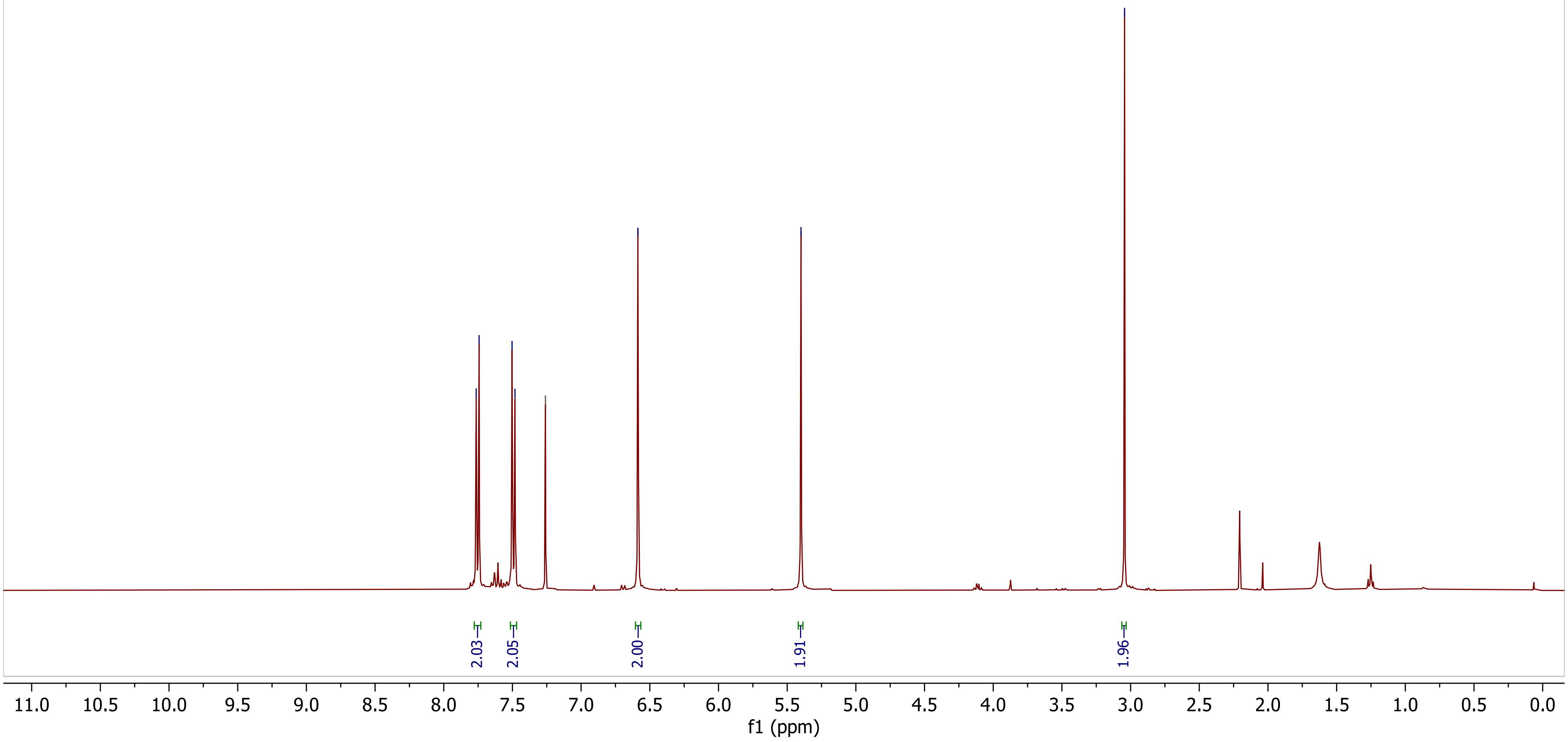

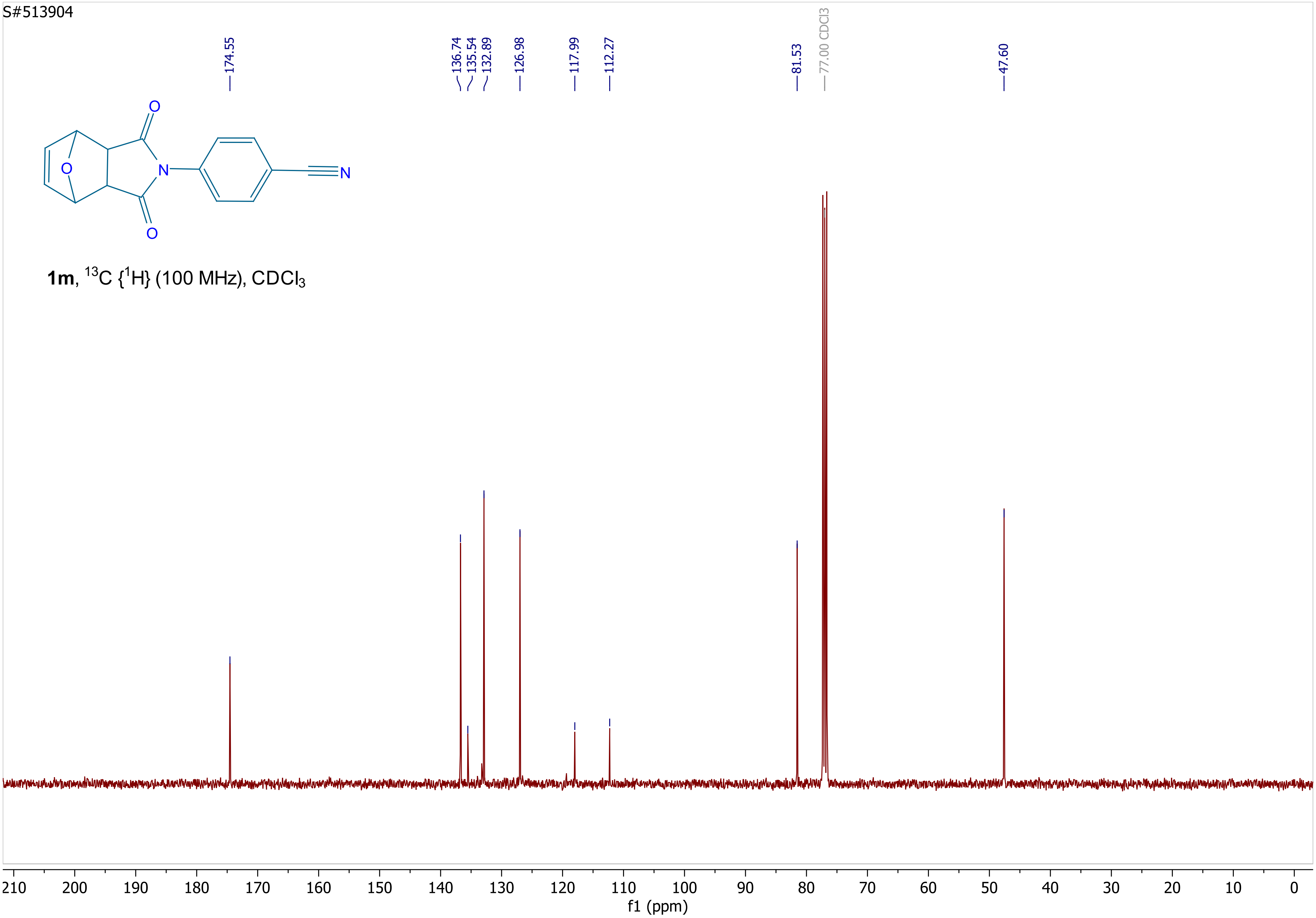
S#513904
174.55
136.74
135.54
132.89
126.98
117.99
112.27
81.53
77.00 CDCl3
47.60
O
O
O
N
N
1m, $^{13}C\ \{^{1}H\}$ (100 MHz), $CDCl_3$
210 200 190 180 170 160 150 140 130 120 110 100 90 80 70 60 50 40 30 20 10 0
f1 (ppm)

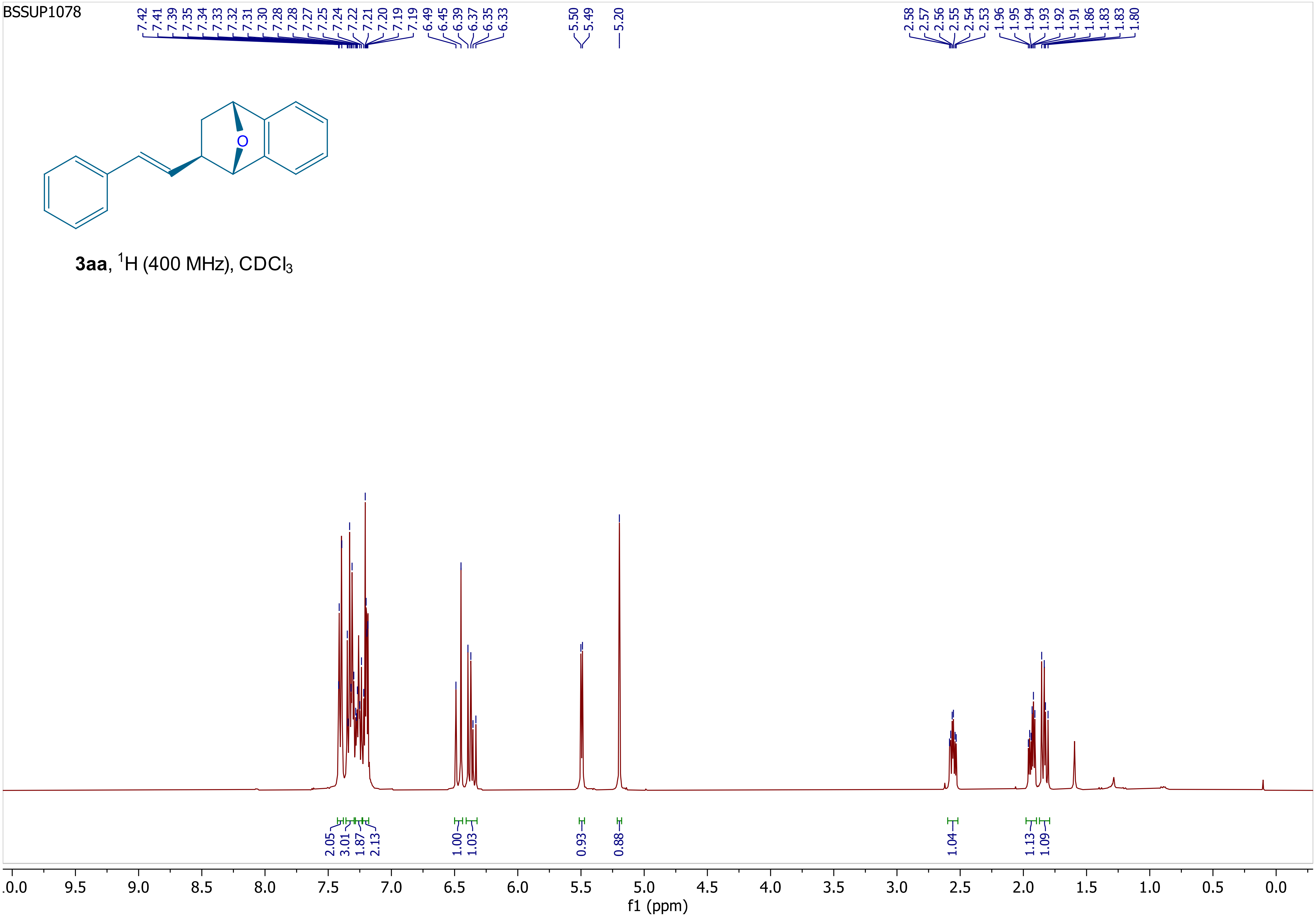
BSSUP1078
7.42 7.41 7.39 7.35 7.34 7.33 7.32 7.31 7.30 7.28 7.28 7.27 7.25 7.24 7.22 7.21 7.20 7.19 7.19 6.49 6.45 6.39 6.37 6.35 6.33
5.50 5.49
5.20
2.58 2.57 2.56 2.55 2.54 2.53 1.96 1.95 1.94 1.93 1.92 1.91 1.86 1.83 1.83 1.80
O
3aa, ^{1}H (400 MHz), $CDCl_3$
2.05 3.01 1.87 2.13
1.00 1.03
0.93
0.88
1.04
1.13 1.09
10.0 9.5 9.0 8.5 8.0 7.5 7.0 6.5 6.0 5.5 5.0 4.5 4.0 3.5 3.0 2.5 2.0 1.5 1.0 0.5 0.0
f1 (ppm)

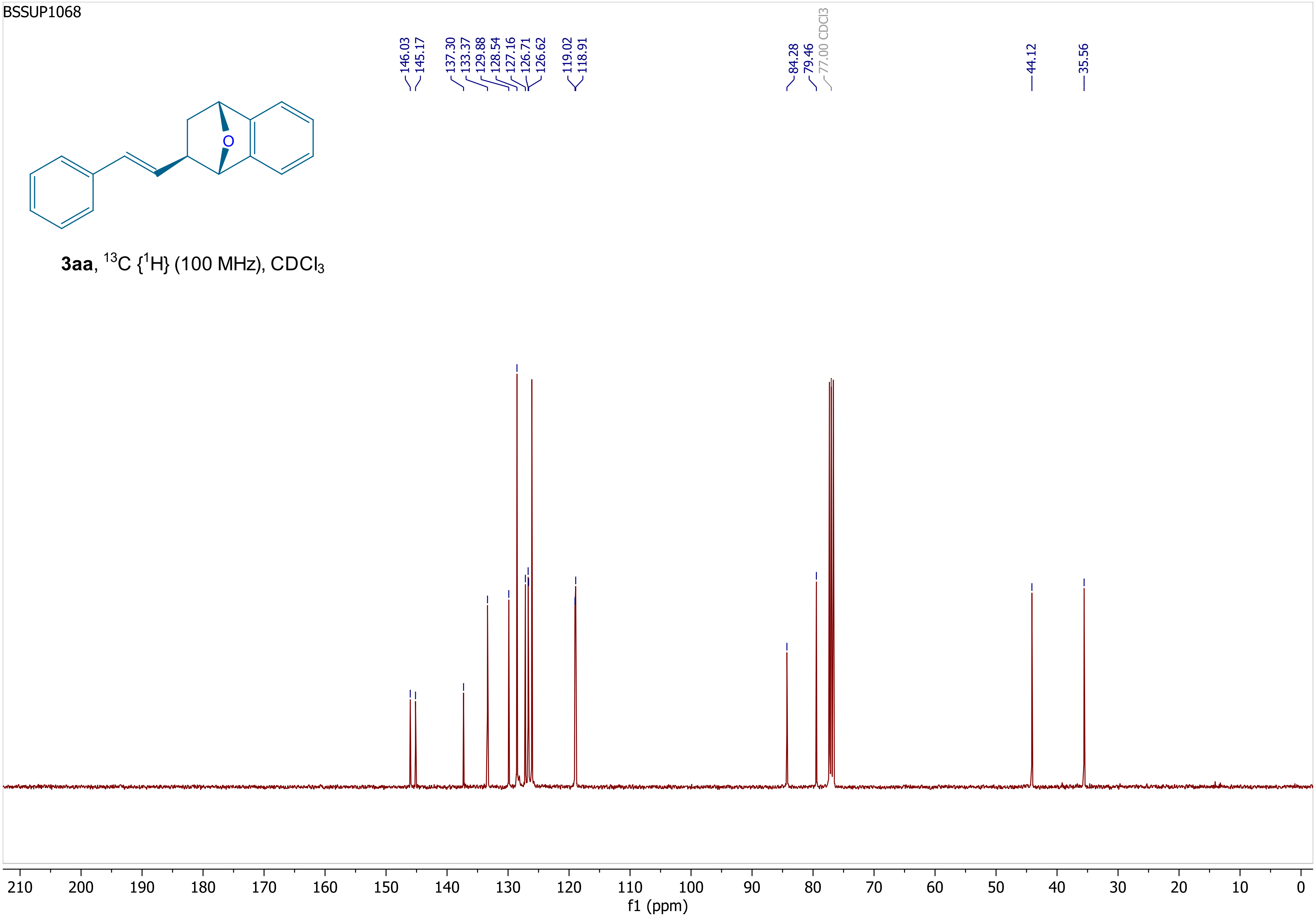

BSSUP1068
146.03
145.17
137.30
133.37
129.88
128.54
127.16
126.71
126.62
119.02
118.91
84.28
79.46
77.00 CDCl3
44.12
35.56
O
3aa, $^{13}C\ \{^1H\}$ (100 MHz), $CDCl_3$
210 200 190 180 170 160 150 140 130 120 110 100 90 80 70 60 50 40 30 20 10 0
f1 (ppm)

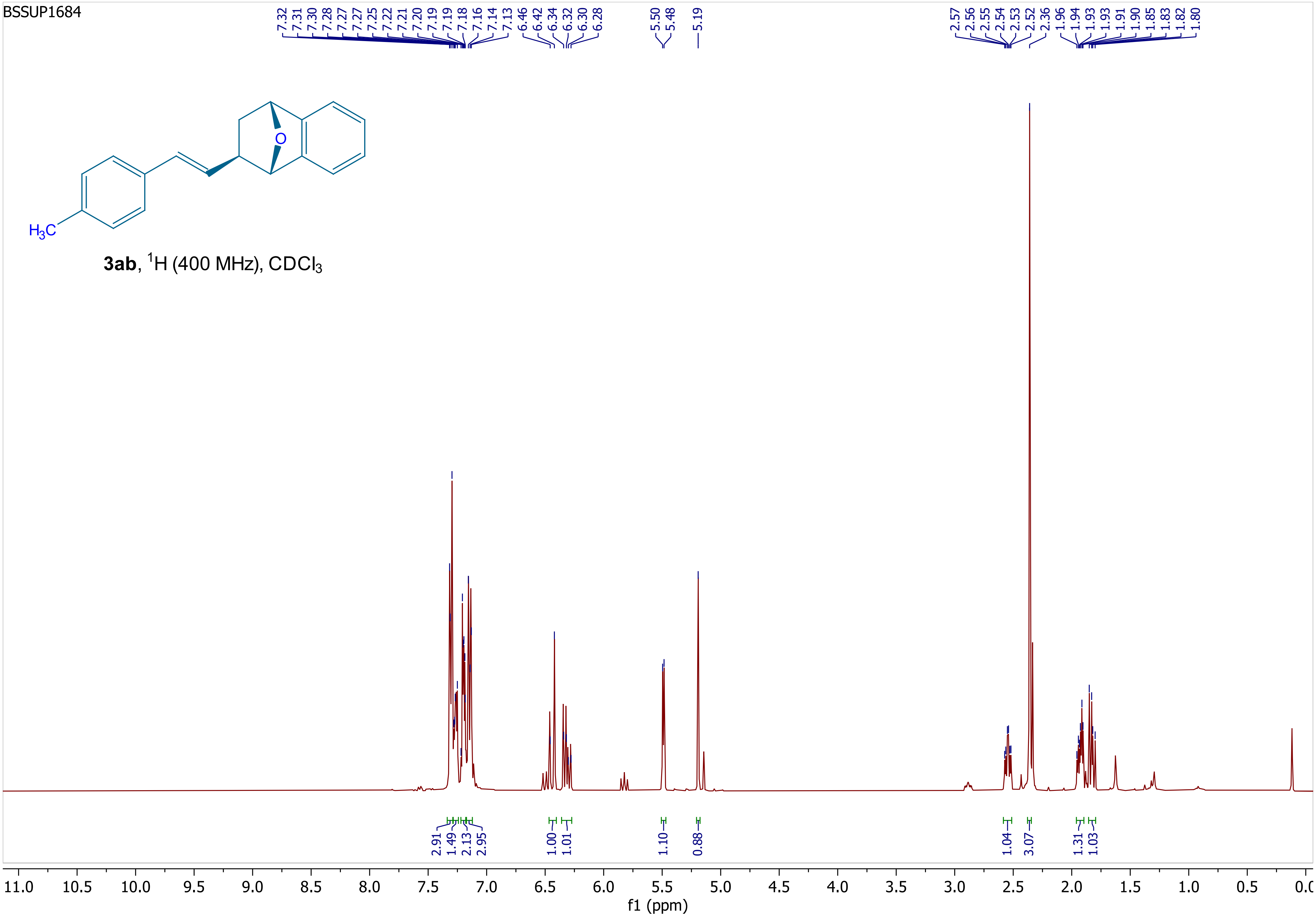
BSSUP1684
H3C
O
3ab, 1H (400 MHz), CDCl3
7.32 7.31 7.30 7.28 7.27 7.27 7.25 7.22 7.21 7.20 7.19 7.19 7.18 7.16 7.14 7.13 6.46 6.42 6.34 6.32 6.30 6.28
5.50 5.48 5.19
2.57 2.56 2.55 2.54 2.53 2.52 2.36 1.96 1.94 1.93 1.93 1.91 1.90 1.85 1.83 1.82 1.80
2.91 1.49 2.13 2.95 1.00 1.01 1.10 0.88 1.04 3.07 1.31 1.03
11.0 10.5 10.0 9.5 9.0 8.5 8.0 7.5 7.0 6.5 6.0 5.5 5.0 4.5 4.0 3.5 3.0 2.5 2.0 1.5 1.0 0.5 0.0
f1 (ppm)

S#200441

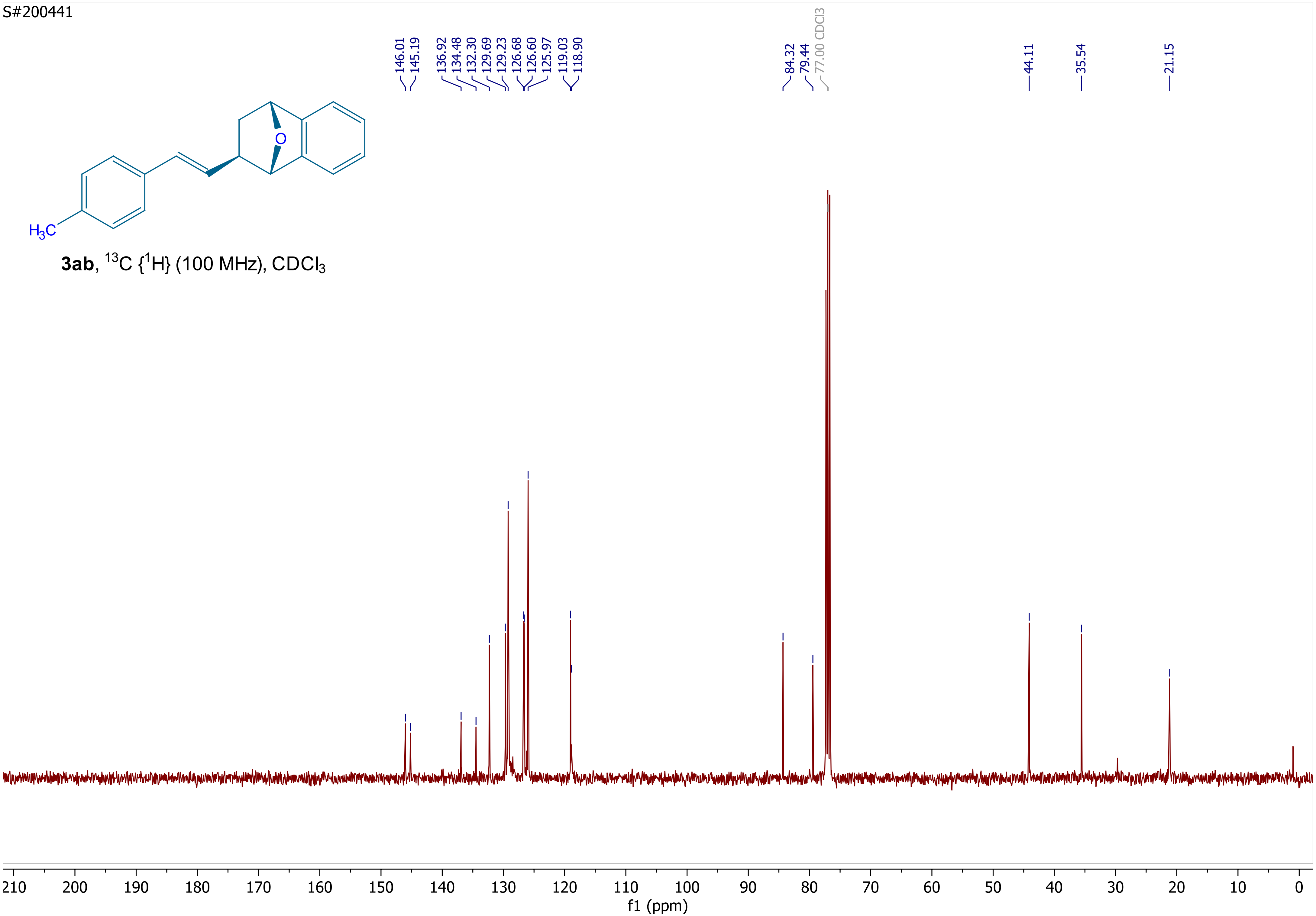
146.01
145.19
136.92
134.48
132.30
129.69
129.23
126.68
126.60
125.97
119.03
118.90
84.32
79.44
77.00 CDCl3
44.11
35.54
21.15
O
H3C
3ab, 13C {1H} (100 MHz), CDCl3
210 200 190 180 170 160 150 140 130 120 110 100 90 80 70 60 50 40 30 20 10 0
f1 (ppm)

S#640351

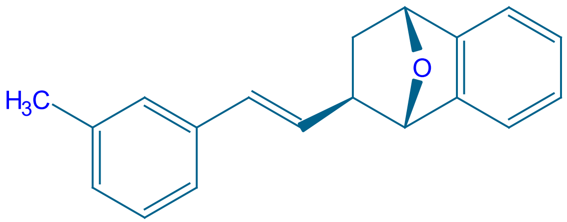

3ac, ^{1}H (400 MHz), $CDCl_3$

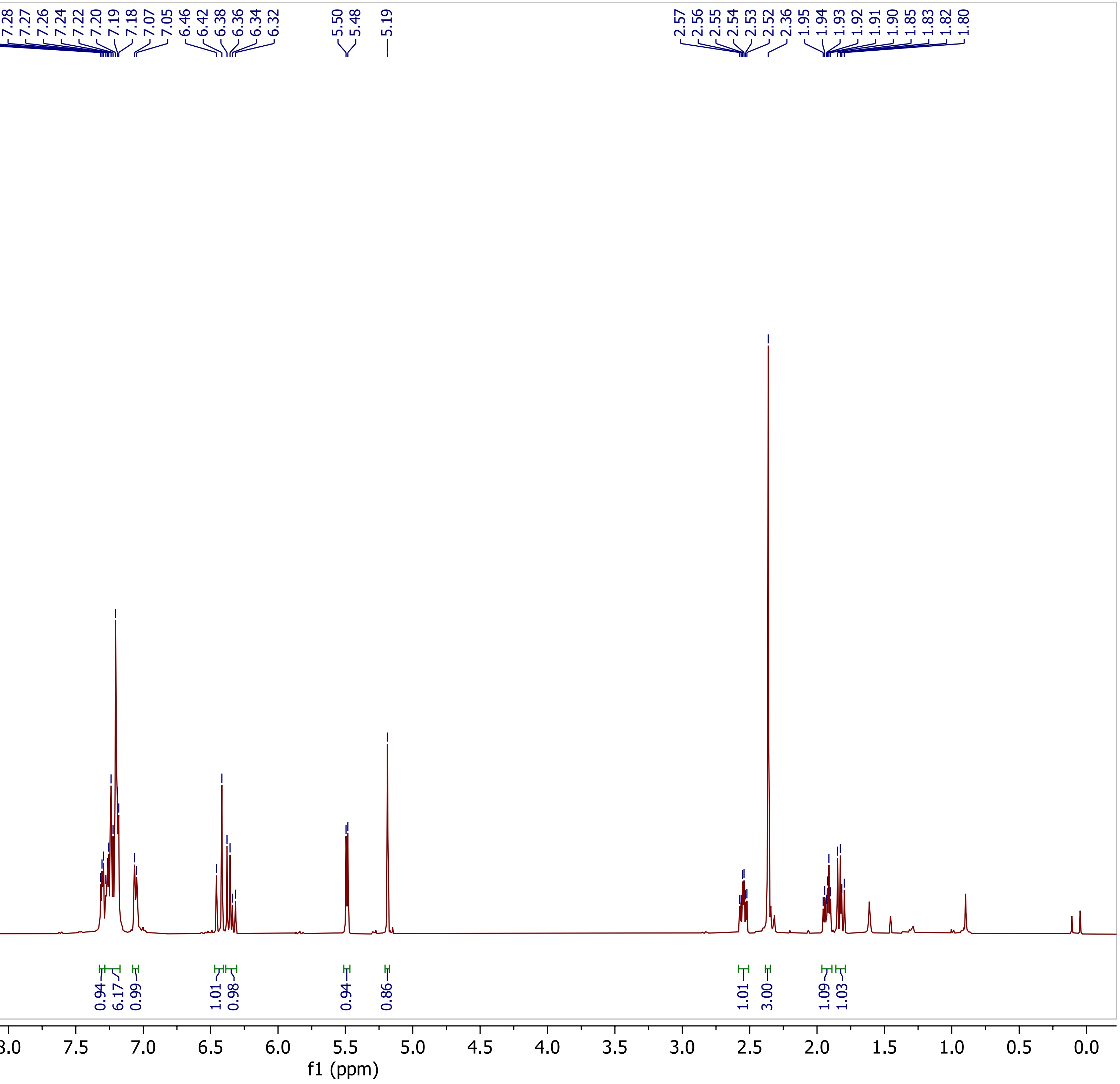

BSSUP1698

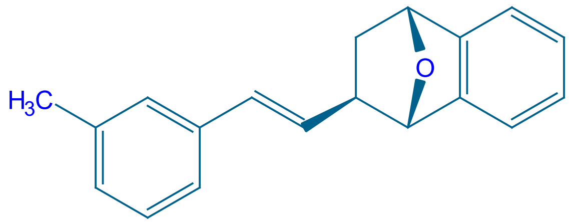

3ac, $^{13}C\ \{^1H\}$ (100 MHz), $CDCl_3$

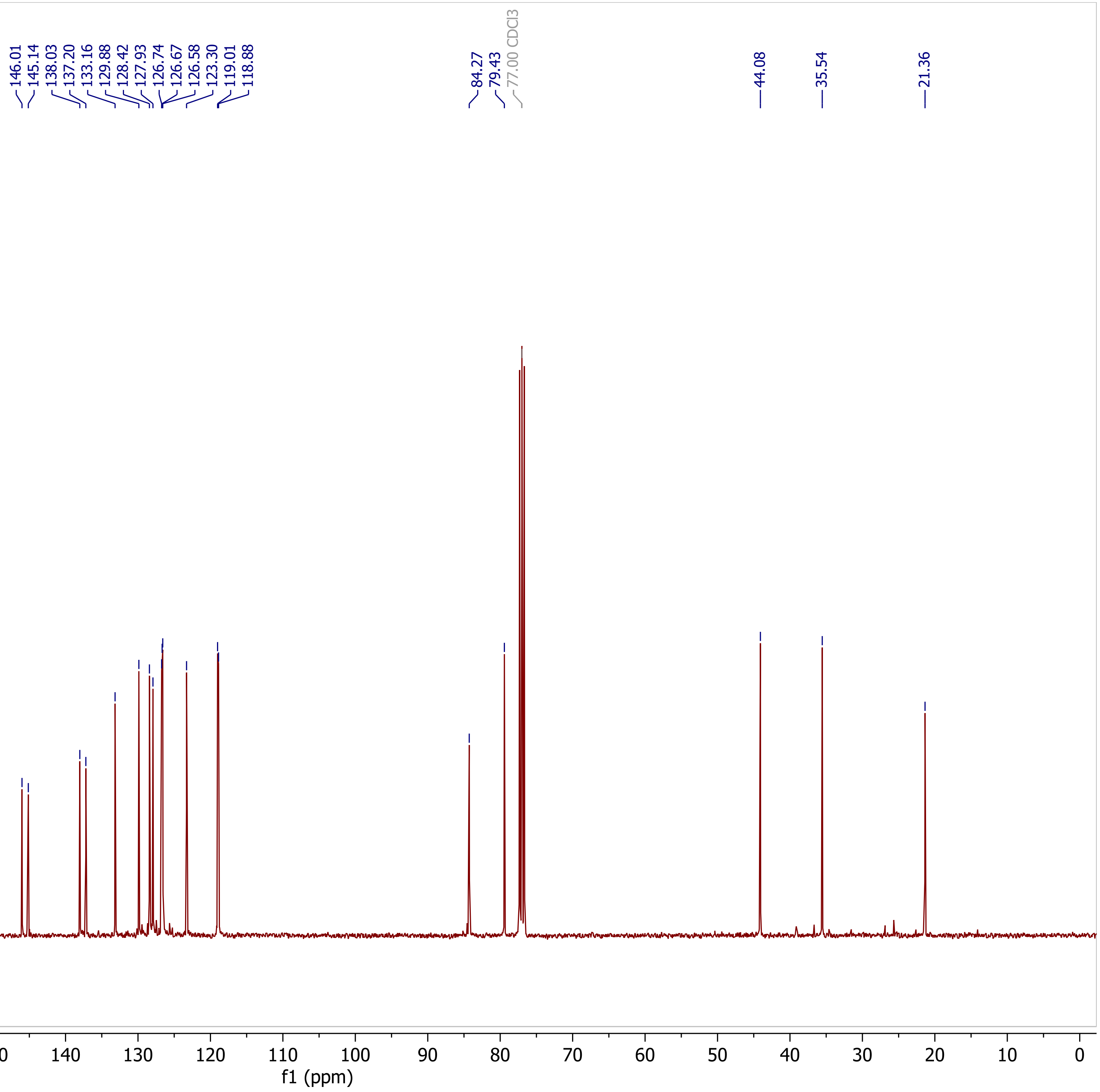

BSSUP1704

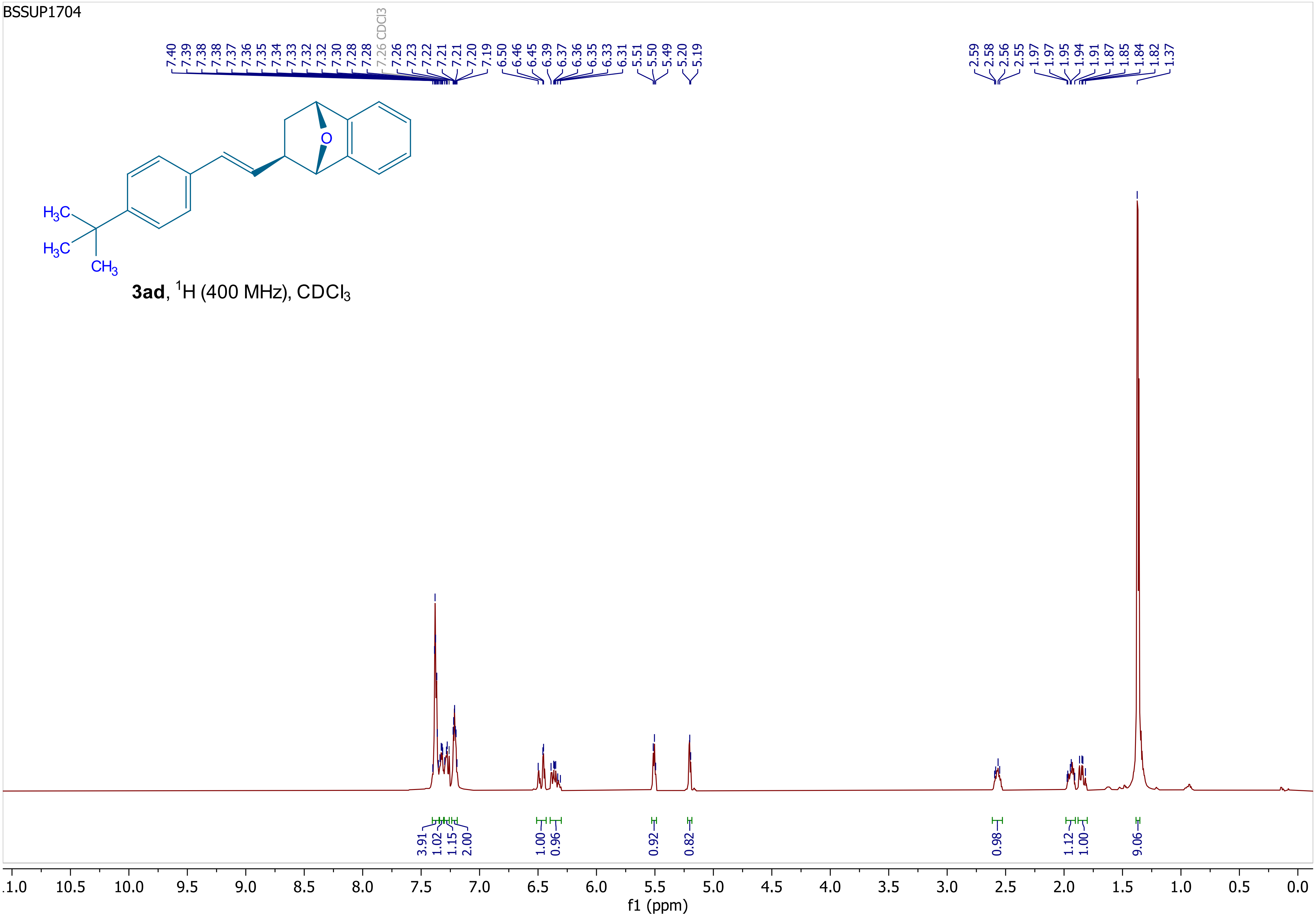
3ad, ¹H (400 MHz), CDCl3
7.40 7.39 7.38 7.38 7.37 7.36 7.35 7.34 7.33 7.32 7.32 7.30 7.28 7.28 7.26 CDCl3 7.26 7.23 7.22 7.21 7.21 7.20 7.19 6.50 6.46 6.45 6.39 6.37 6.36 6.35 6.33 6.31 5.51 5.50 5.49 5.20 5.19
2.59 2.58 2.56 2.55 1.97 1.97 1.95 1.94 1.91 1.87 1.85 1.84 1.82 1.37
3.91 1.02 1.15 2.00 1.00 0.96 0.92 0.82 0.98 1.12 1.00 9.06
f1 (ppm)

BSSUP1704

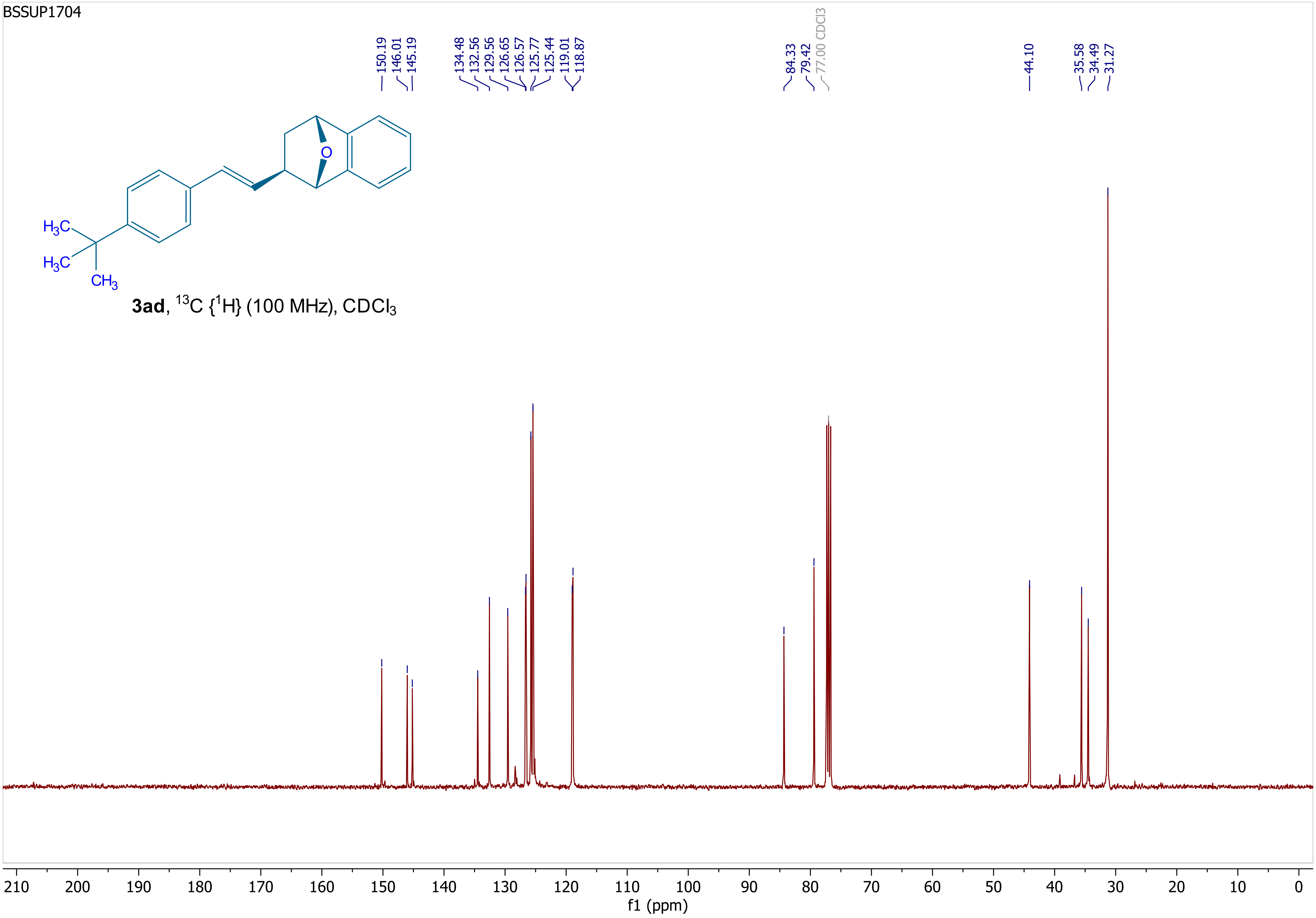

3ad, $^{13}C\ \{^{1}H\}$ (100 MHz), $CDCl_3$

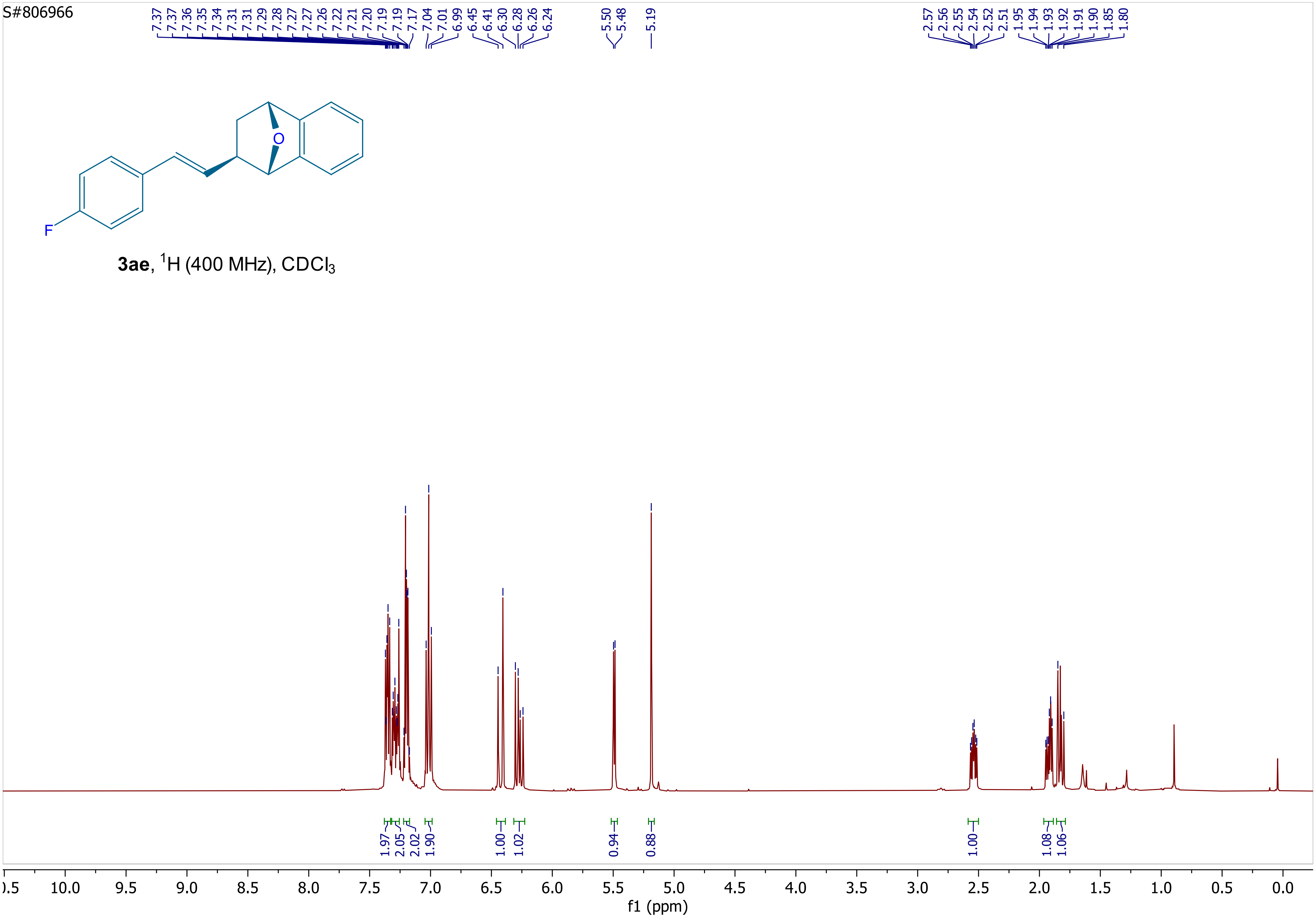
S#806966
3ae, ^{1}H (400 MHz), CDCl$_3$
f1 (ppm)

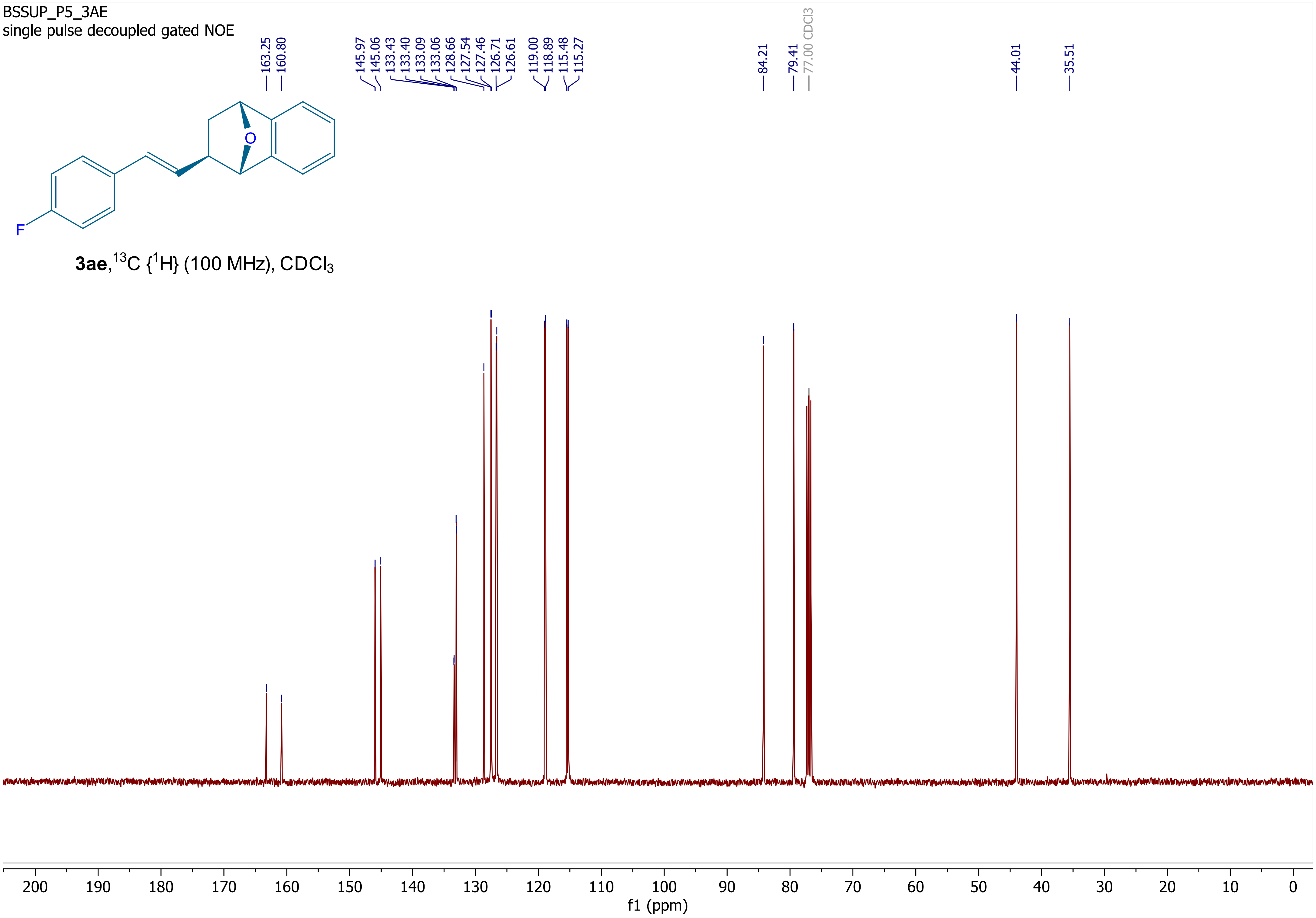
BSSUP_P5_3AE
single pulse decoupled gated NOE
163.25
160.80
145.97
145.06
133.43
133.40
133.09
133.06
128.66
127.54
127.46
126.71
126.61
119.00
118.89
115.48
115.27
84.21
79.41
77.00 CDCl3
44.01
35.51
O
F
3ae, 13C {1H} (100 MHz), CDCl3
200
190
180
170
160
150
140
130
120
110
100
90
80
70
60
50
40
30
20
10
0
f1 (ppm)

BSSUP-1699
19F

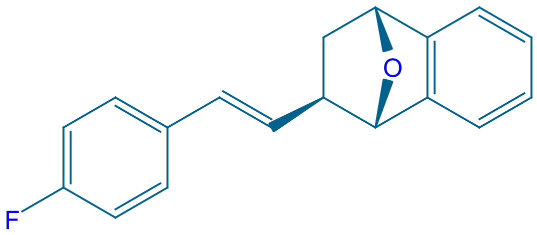

3ae, $^{19}F\ \{^1H\}$ (373 MHz), $CDCl_3$

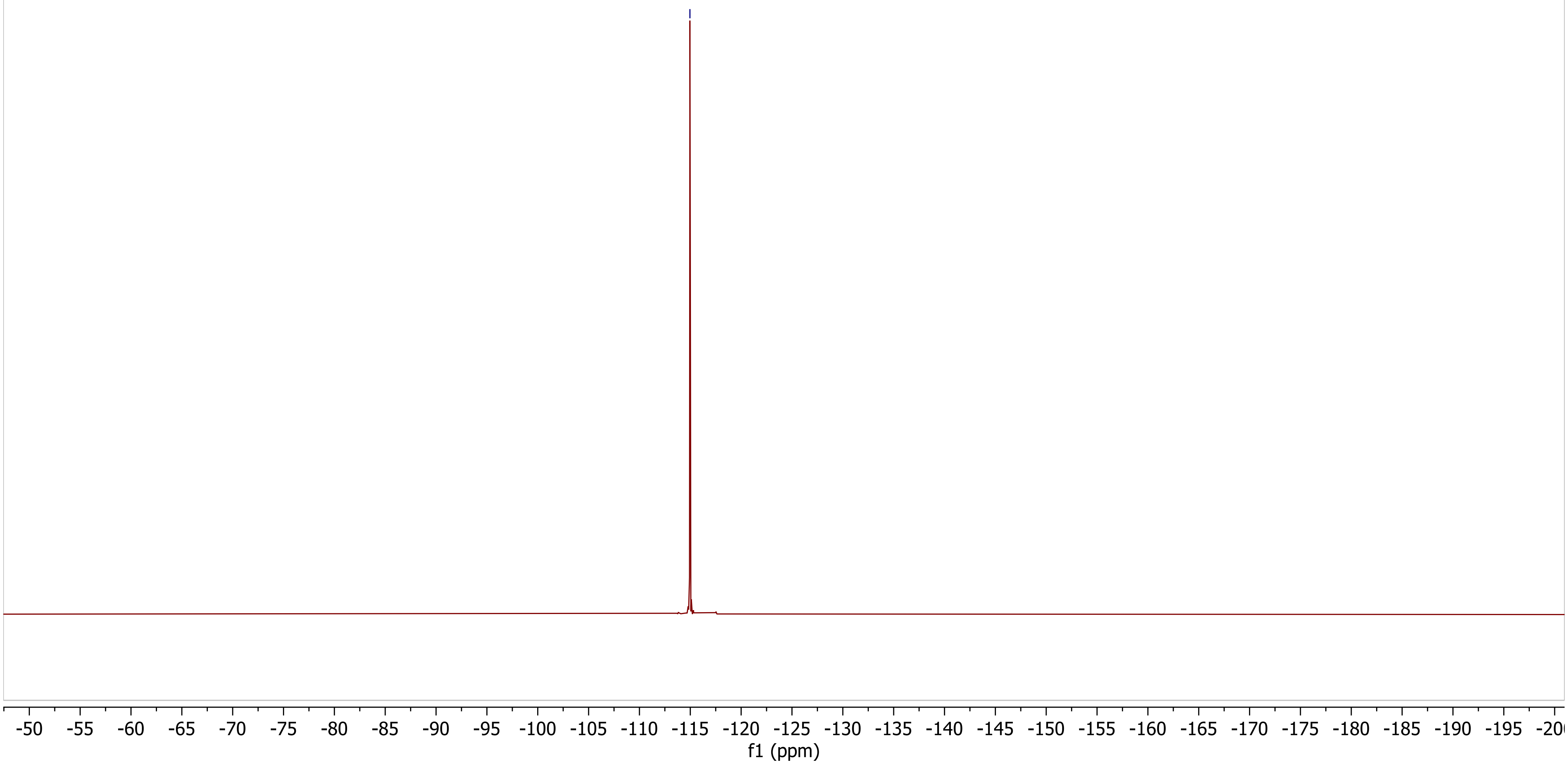

S#579479

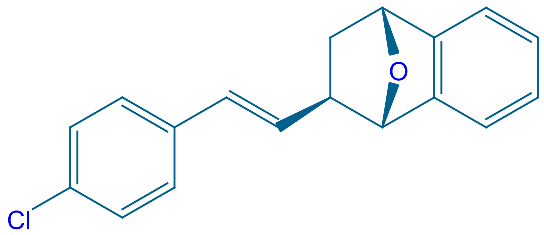

3af, ^{1}H (400 MHz), $CDCl_3$

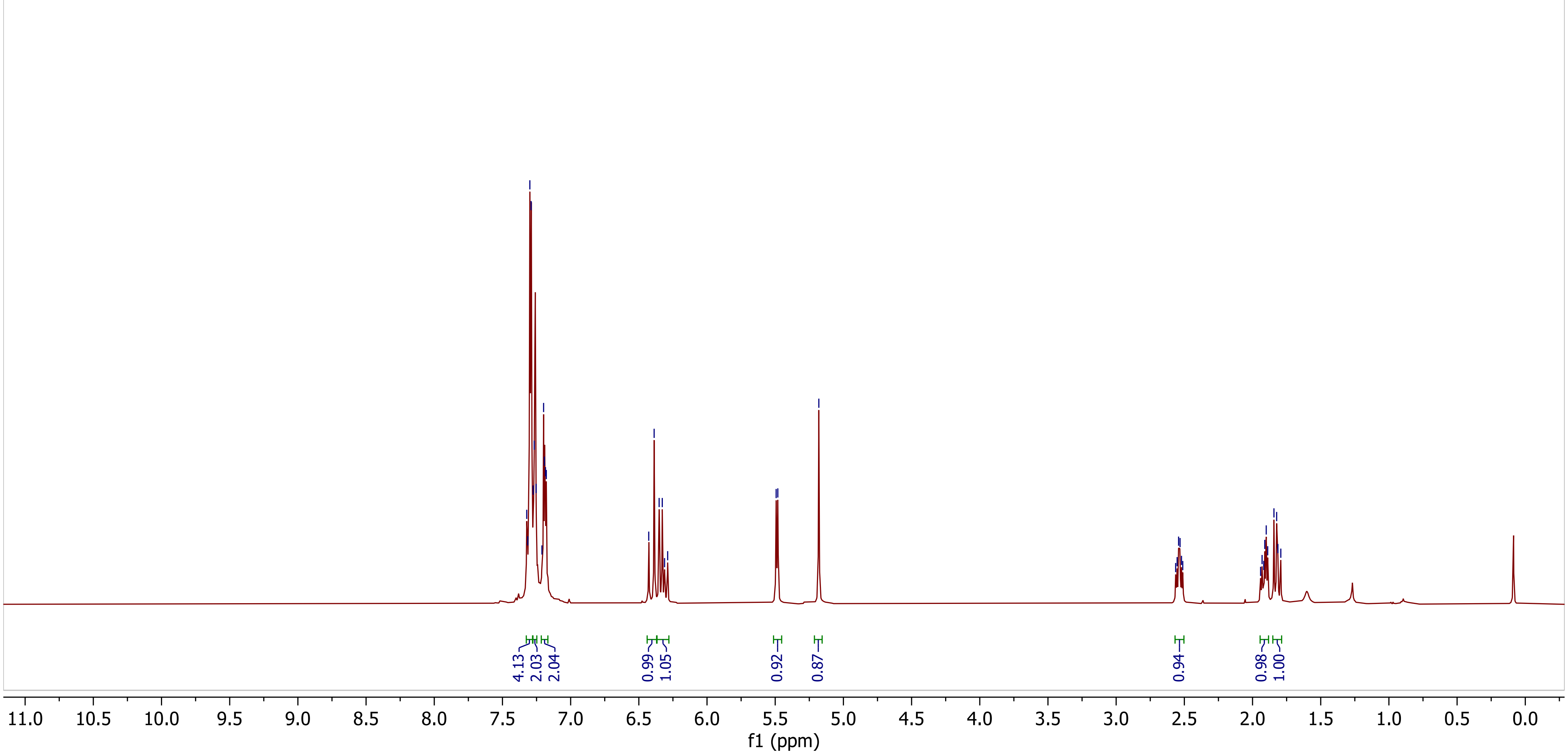

S#450824

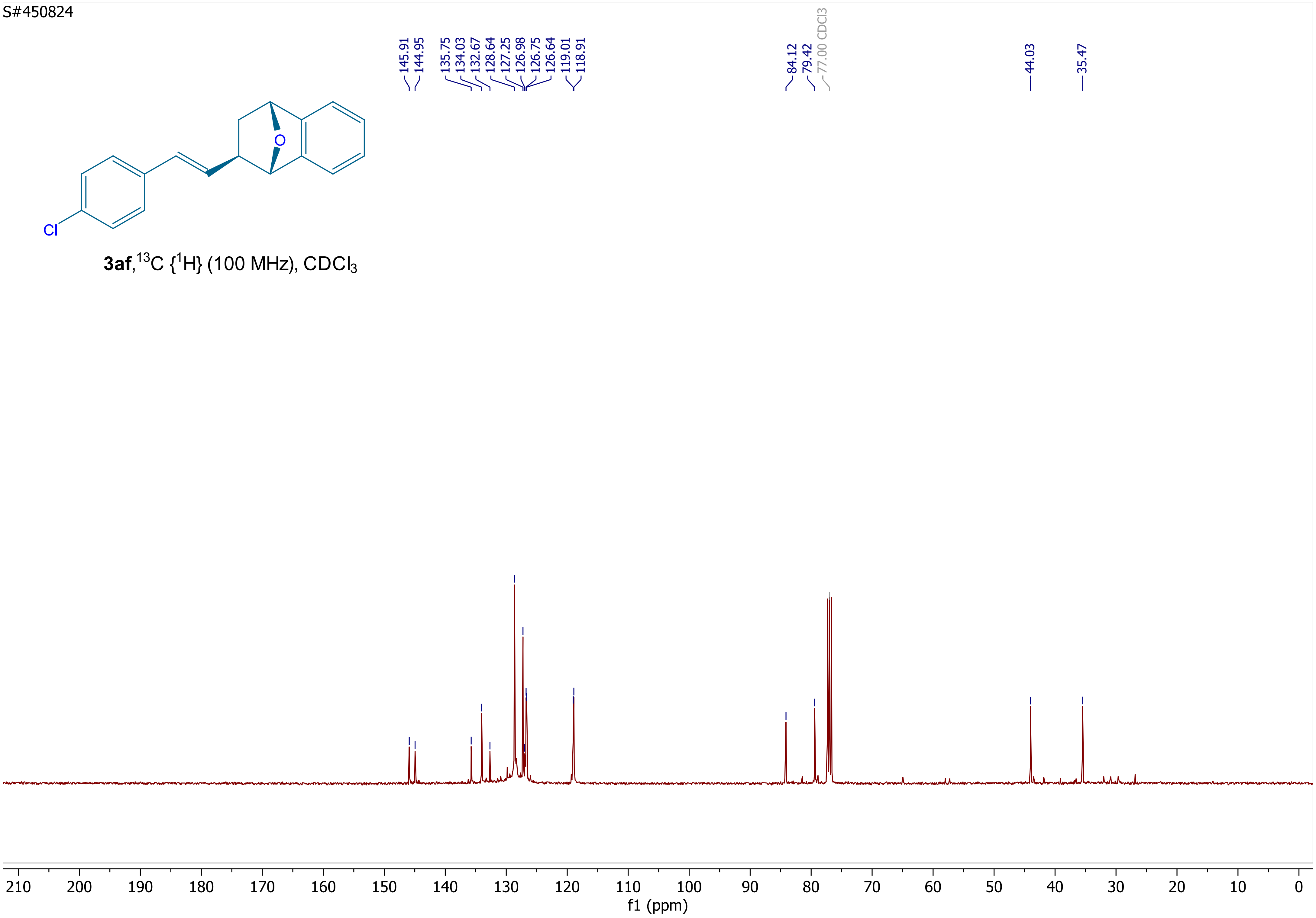

3af, $^{13}C\{^{1}H\}$ (100 MHz), $CDCl_3$

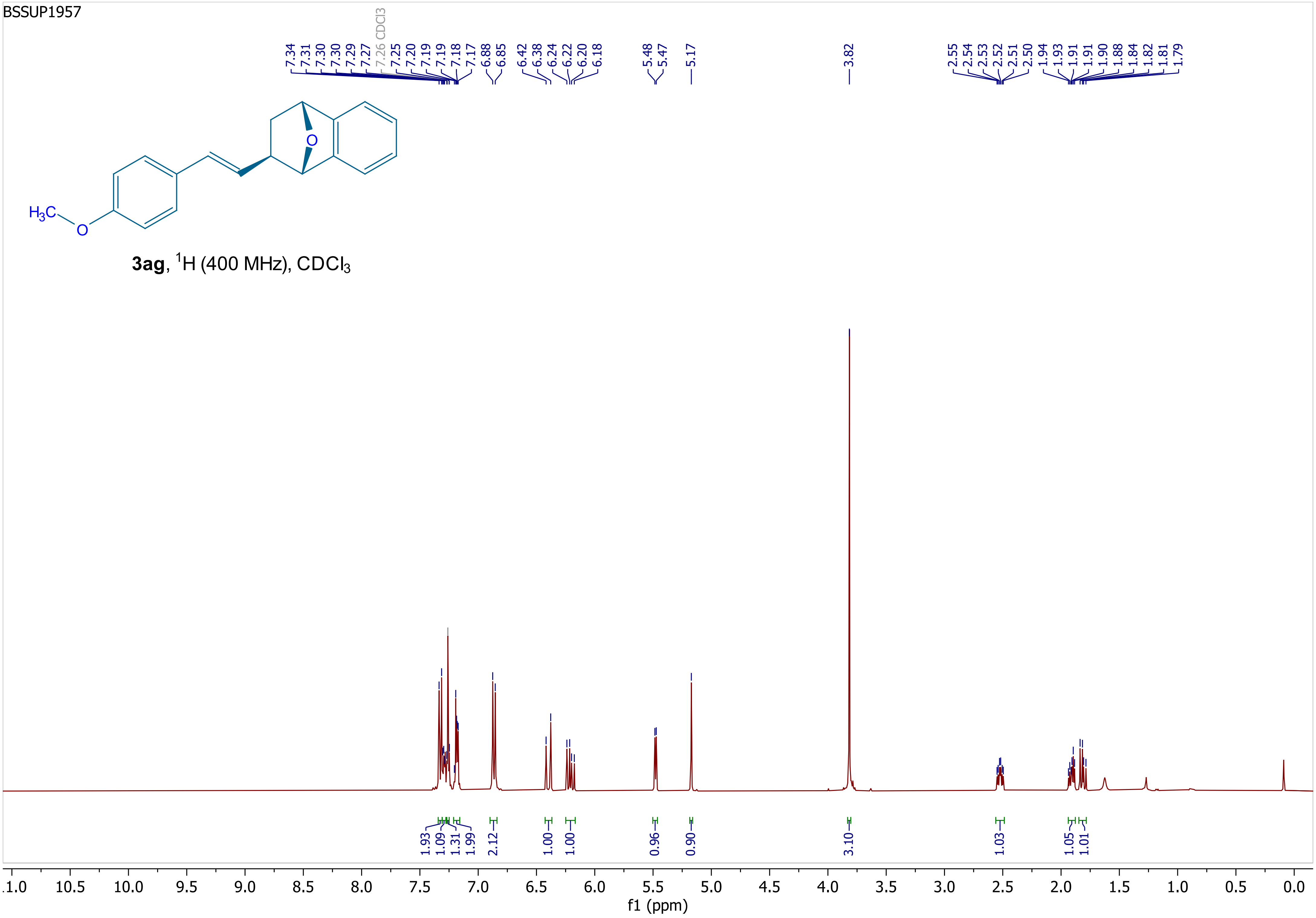

BSSUP1957
3ag, 1H (400 MHz), CDCl3
H3C
O
7.34 7.31 7.30 7.30 7.29 7.27 7.26 CDCl3 7.25 7.20 7.19 7.19 7.18 7.17 6.88 6.85 6.42 6.38 6.24 6.22 6.20 6.18 5.48 5.47 5.17 3.82 2.55 2.54 2.53 2.52 2.51 2.50 1.94 1.93 1.91 1.91 1.90 1.88 1.84 1.82 1.81 1.79
1.93 1.09 1.31 1.99 2.12 1.00 1.00 0.96 0.90 3.10 1.03 1.05 1.01
f1 (ppm)

S#814725

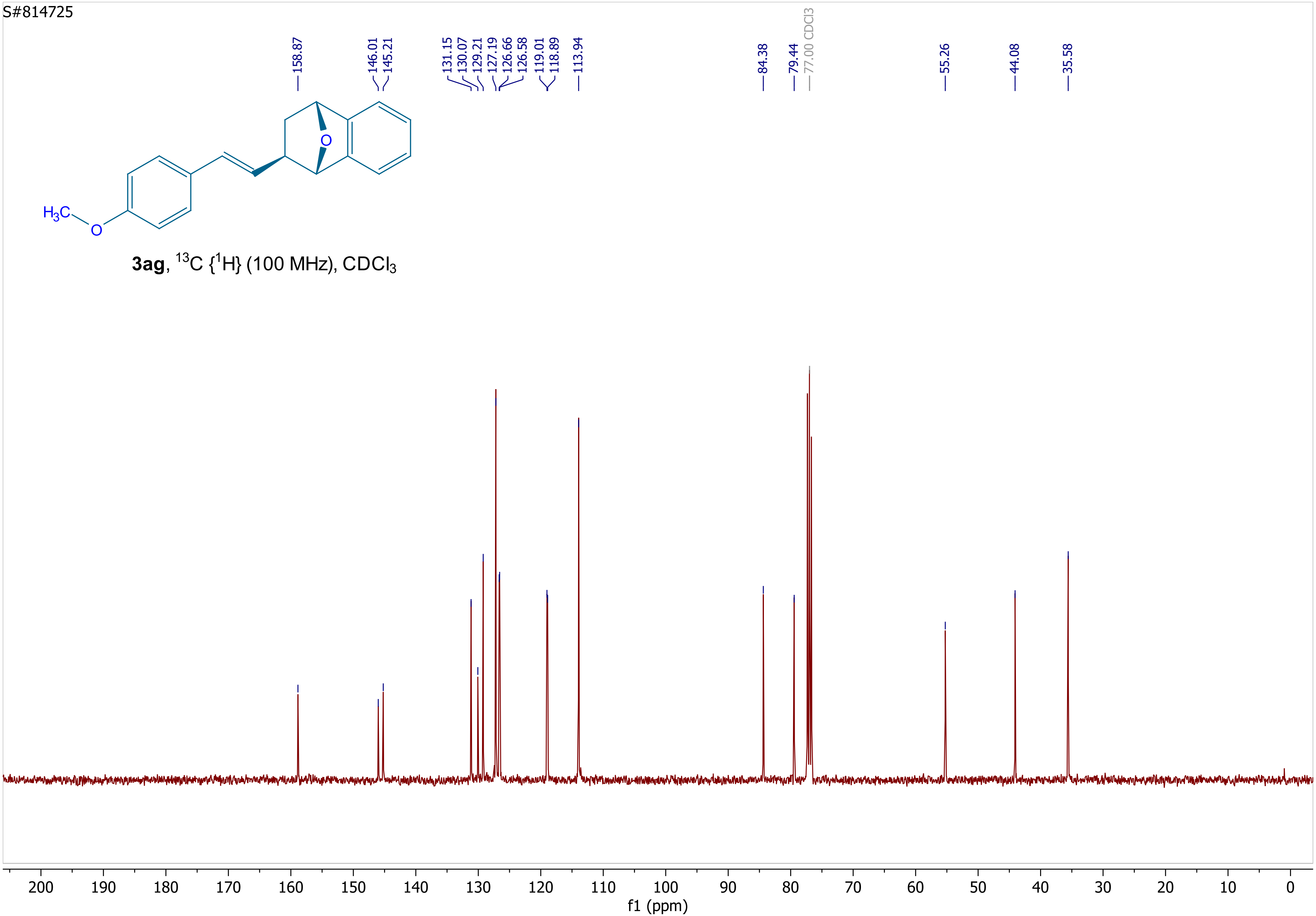

3ag, $^{13}C\ \{^{1}H\}$ (100 MHz), $CDCl_3$

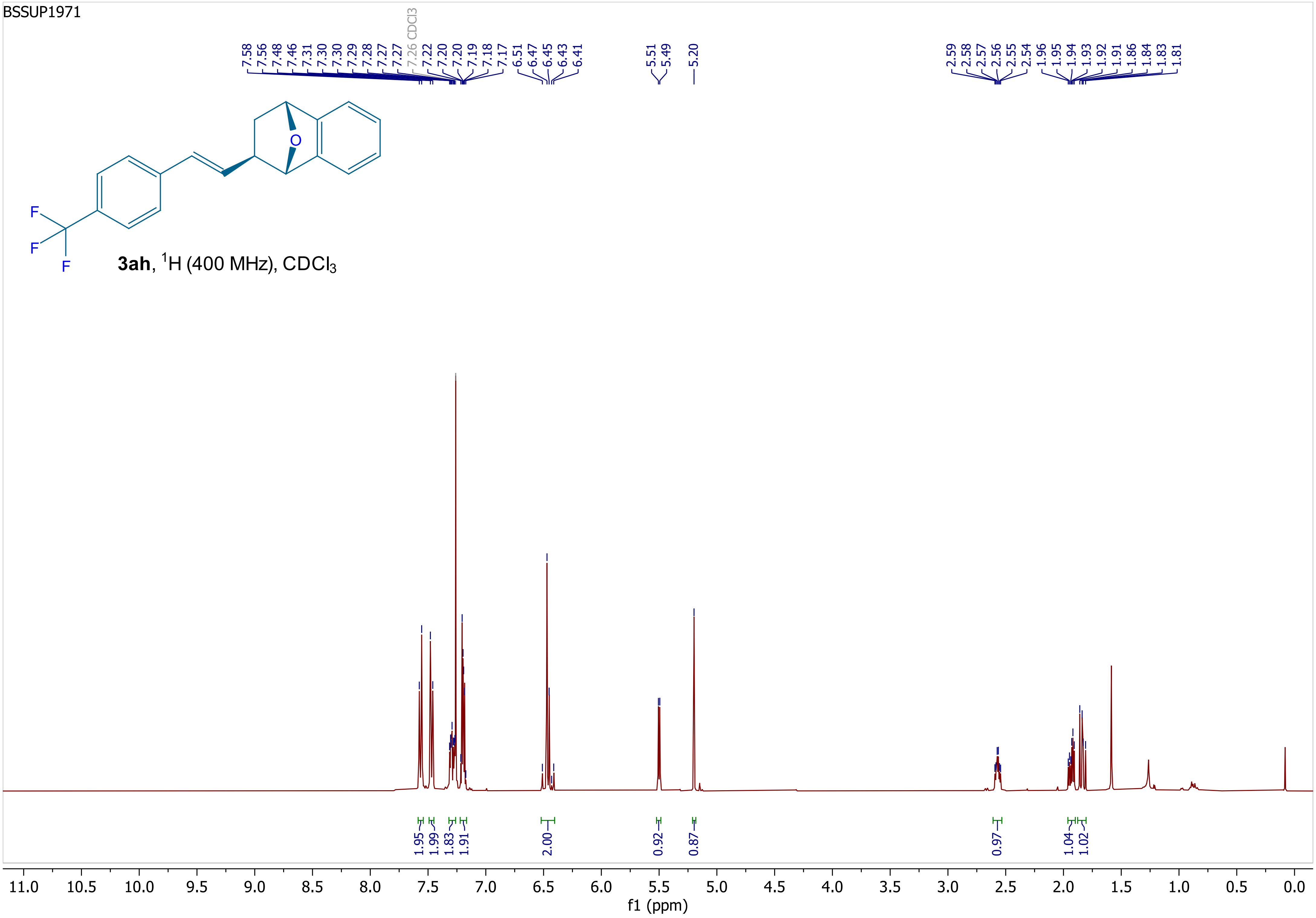
BSSUP1971
7.58
7.56
7.48
7.46
7.31
7.30
7.30
7.29
7.28
7.27
7.27
7.26 CDCl3
7.22
7.20
7.20
7.19
7.18
7.17
6.51
6.47
6.45
6.43
6.41
5.51
5.49
5.20
2.59
2.58
2.57
2.56
2.55
2.54
1.96
1.95
1.94
1.93
1.92
1.91
1.86
1.84
1.83
1.81
O
F
F
F
3ah, ^{1}H (400 MHz), $CDCl_3$
1.95
1.99
1.83
1.91
2.00
0.92
0.87
0.97
1.04
1.02
11.0
10.5
10.0
9.5
9.0
8.5
8.0
7.5
7.0
6.5
6.0
5.5
5.0
4.5
4.0
3.5
3.0
2.5
2.0
1.5
1.0
0.5
0.0
f1 (ppm)

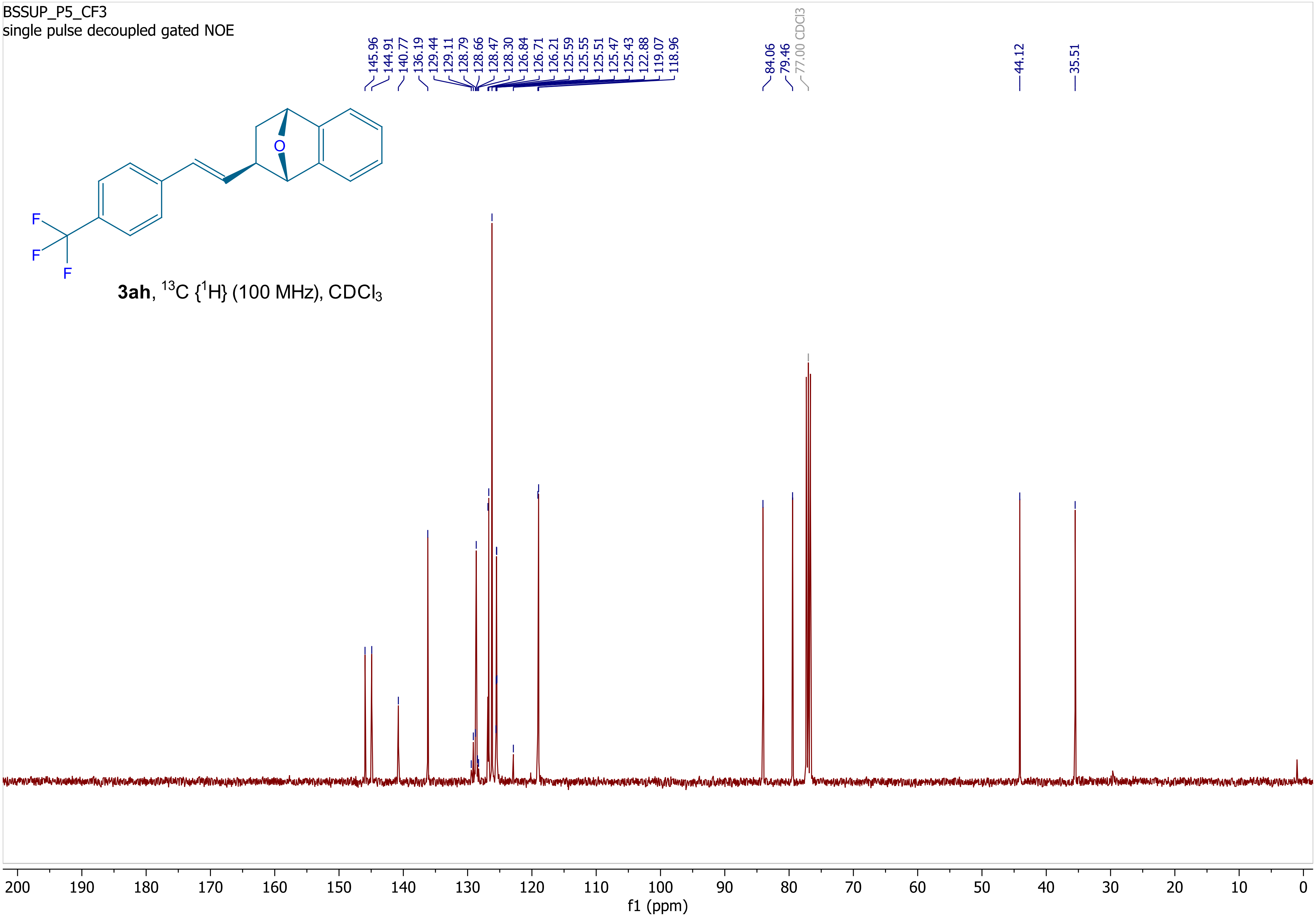
BSSUP_P5_CF3
single pulse decoupled gated NOE
145.96
144.91
140.77
136.19
129.44
129.11
128.79
128.66
128.47
128.30
126.84
126.71
126.21
125.59
125.55
125.51
125.47
125.43
122.88
119.07
118.96
84.06
79.46
77.00 CDCl3
44.12
35.51
F
F
F
O
3ah, ^{13}C {^{1}H} (100 MHz), CDCl$_3$
f1 (ppm)

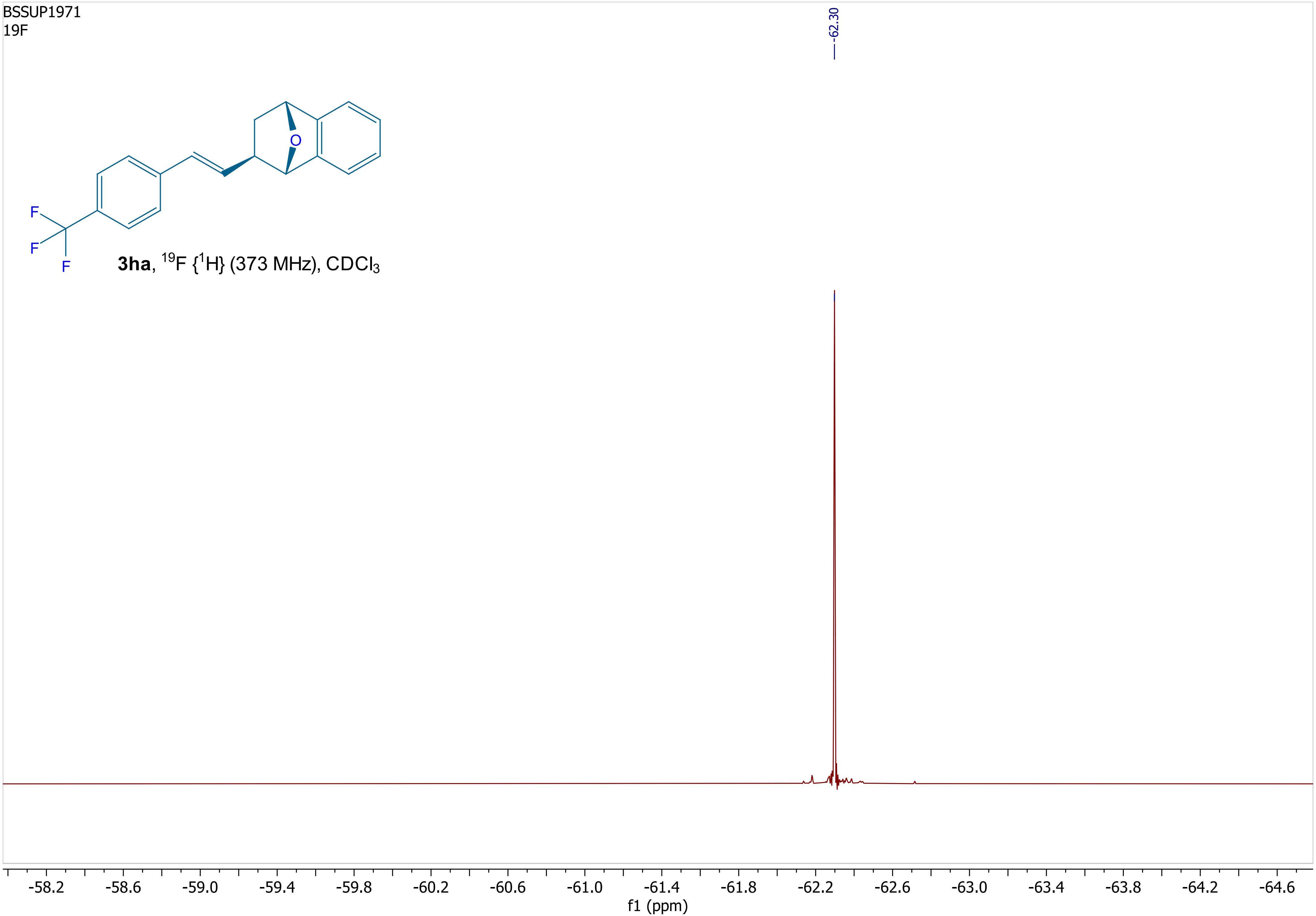

BSSUP1971
19F
-62.30
F
F
F
O
3ha, ^{19}F {^{1}H} (373 MHz), $CDCl_3$
-58.2 -58.6 -59.0 -59.4 -59.8 -60.2 -60.6 -61.0 -61.4 -61.8 -62.2 -62.6 -63.0 -63.4 -63.8 -64.2 -64.6
f1 (ppm)

S#680961

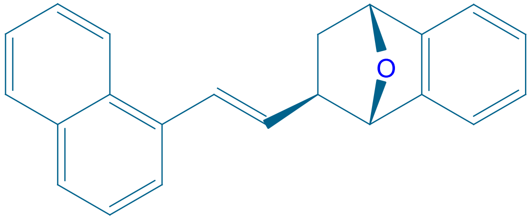

3ai, ^{1}H (400 MHz), $CDCl_3$

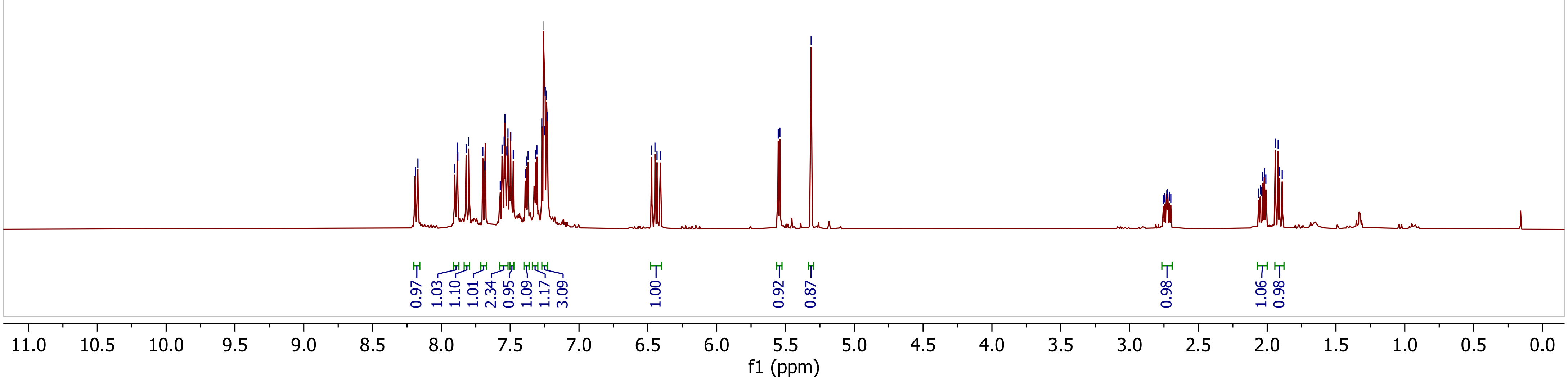

S#68026

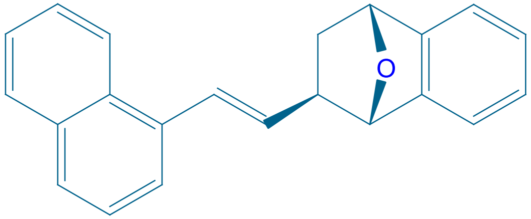

3ai, $^{13}C\ \{^{1}H\}$ (100 MHz), $CDCl_3$

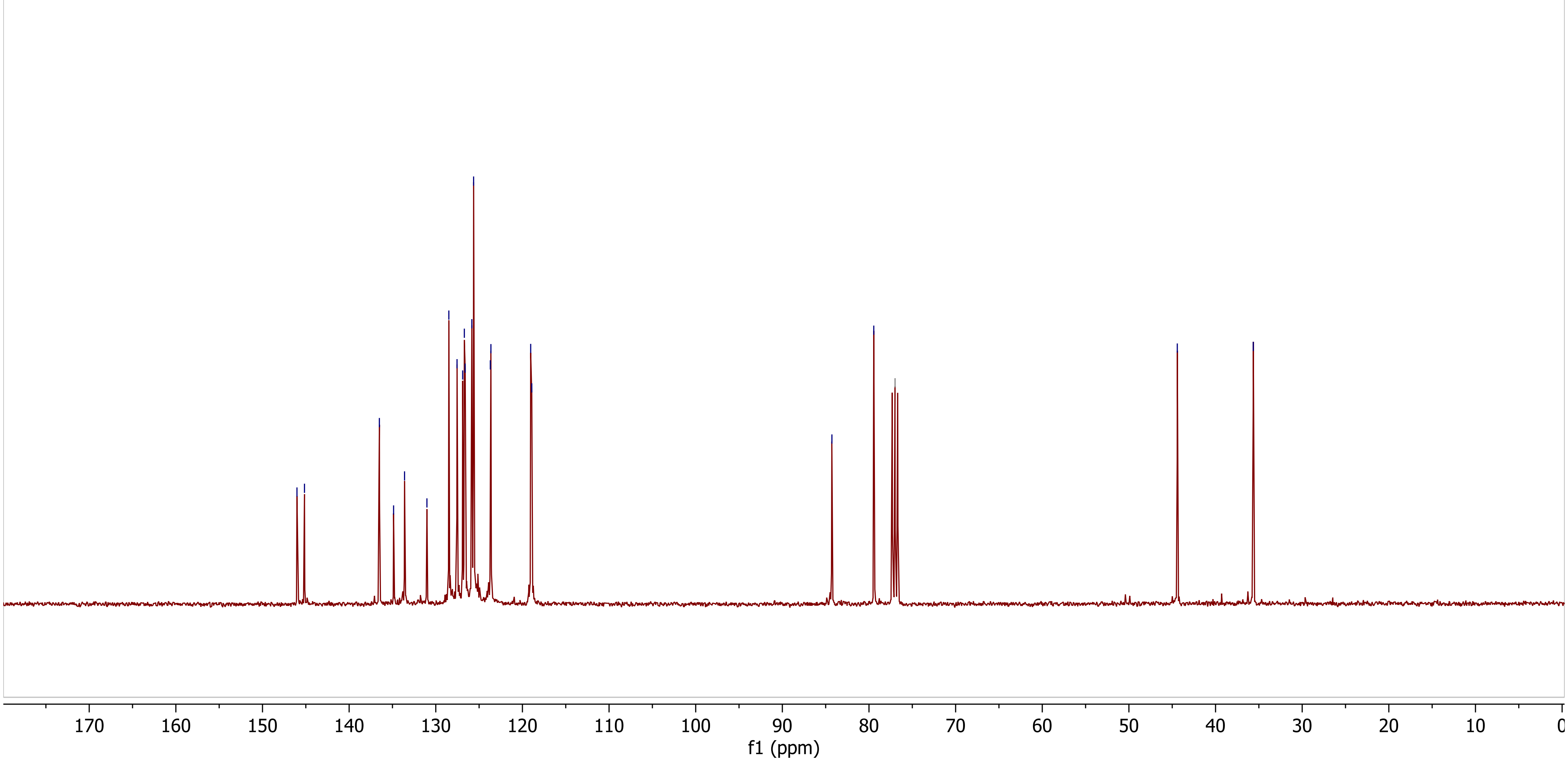

BSSUP_2466B
single_pulse

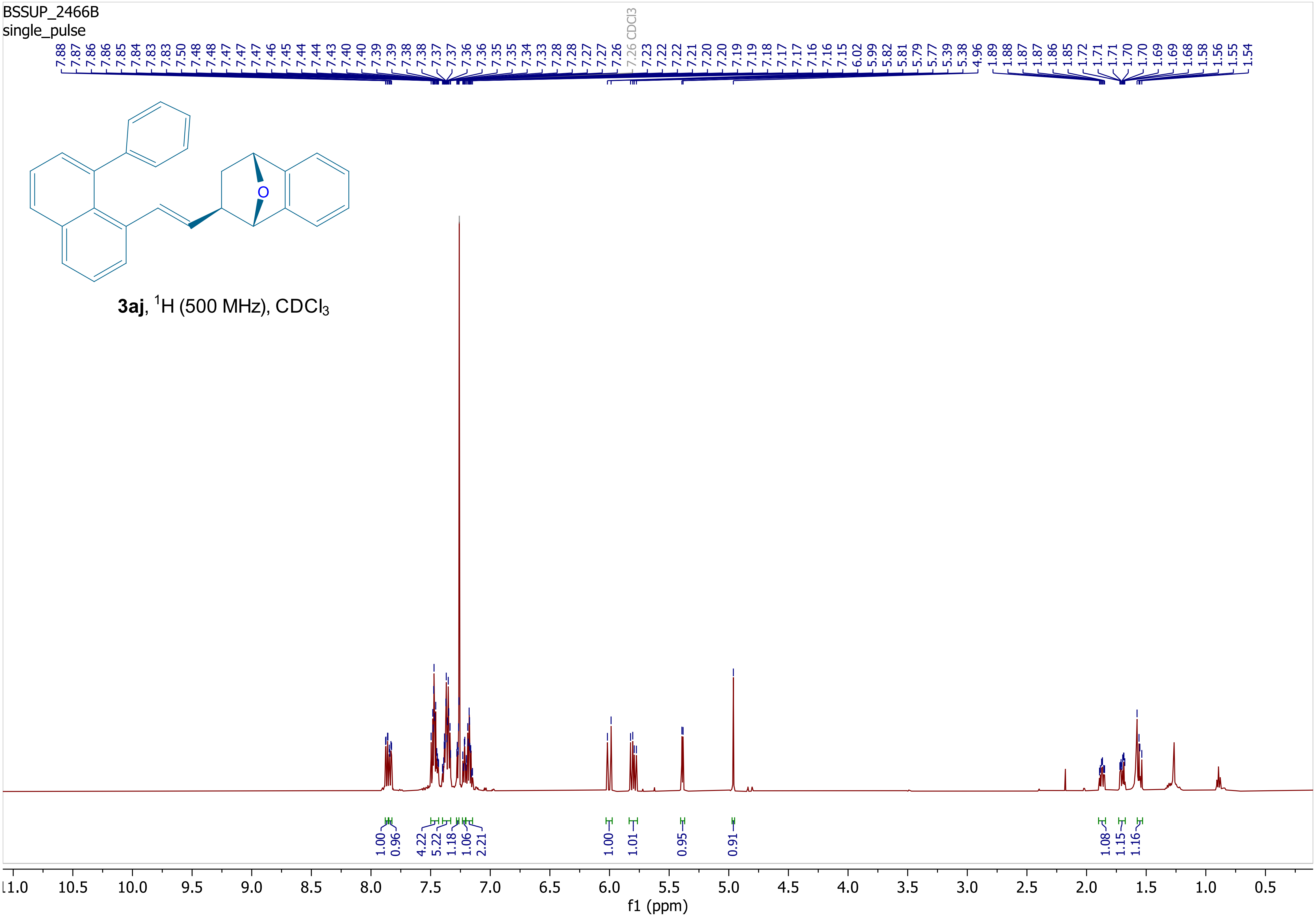

3aj, ^{1}H (500 MHz), $CDCl_3$

BSSUP_2466B
single pulse decoupled gated NOE

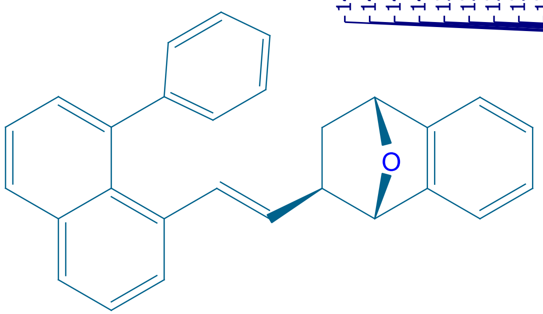

3aj, $^{13}C\{^{1}H\}$ (100 MHz), $CDCl_3$

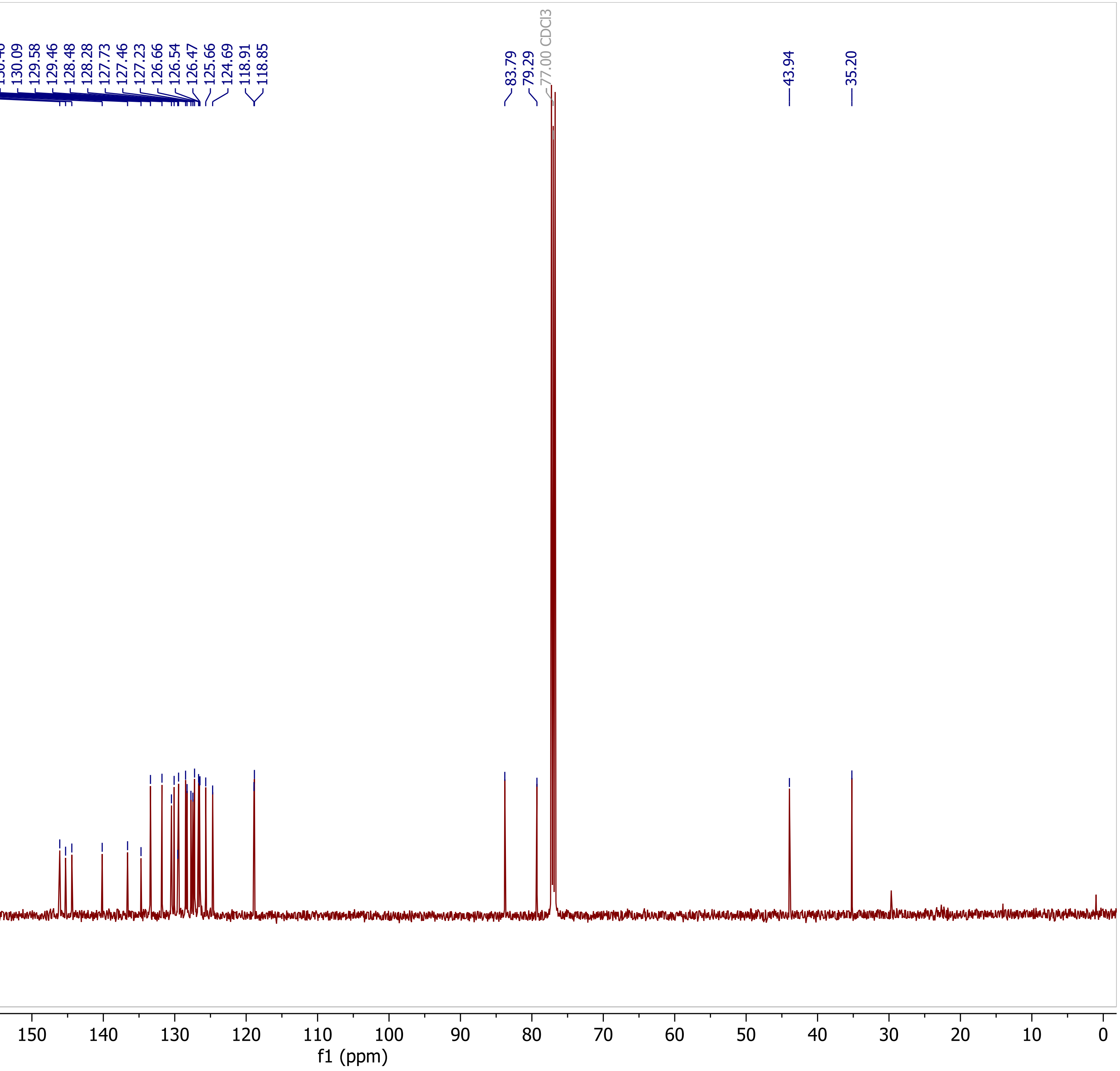

S#458812

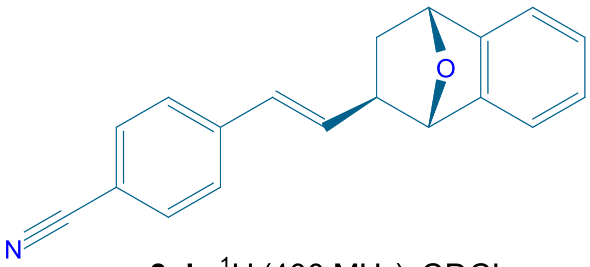

3ak, ^{1}H (400 MHz), $CDCl_3$

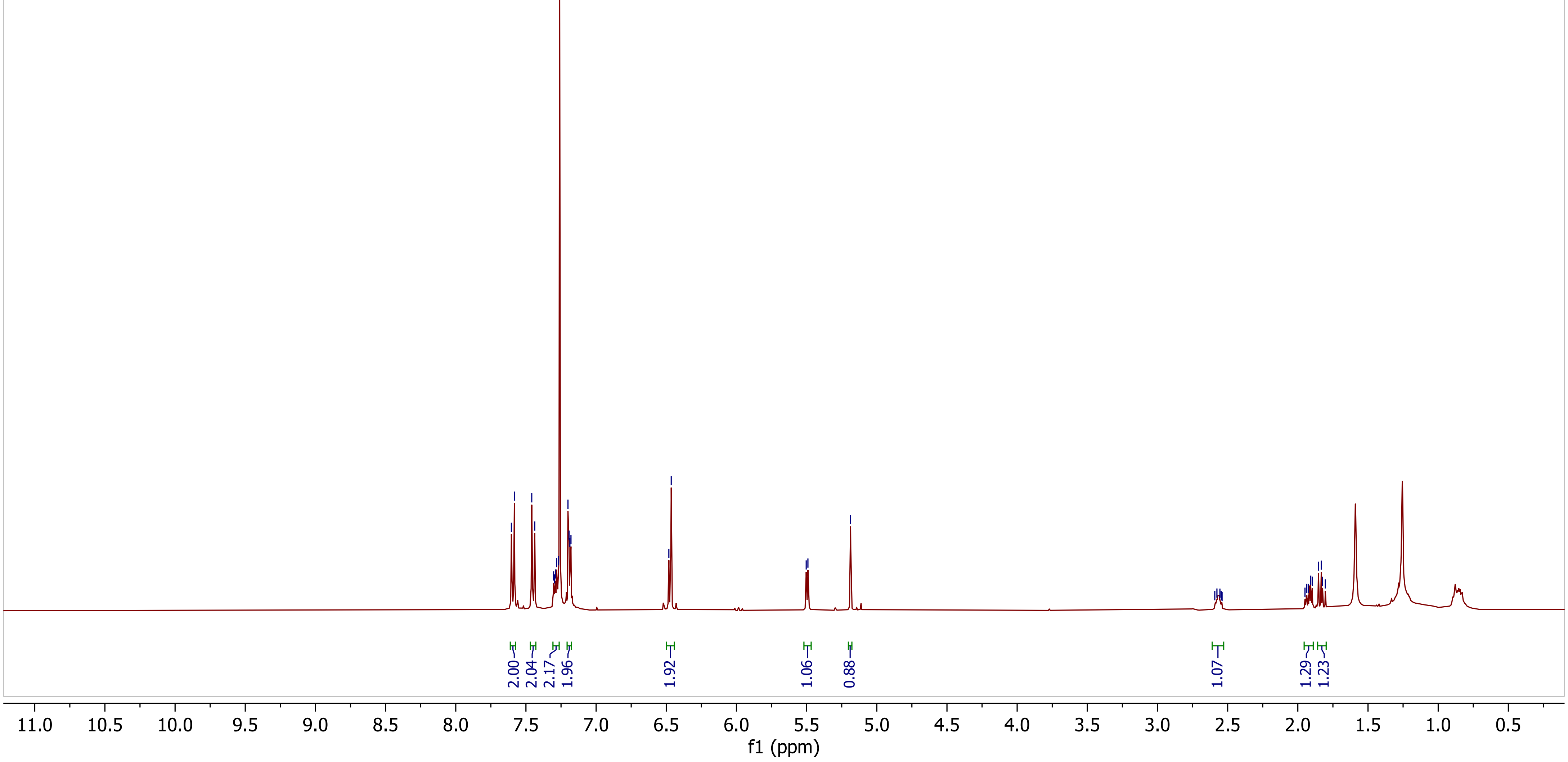

S#608948

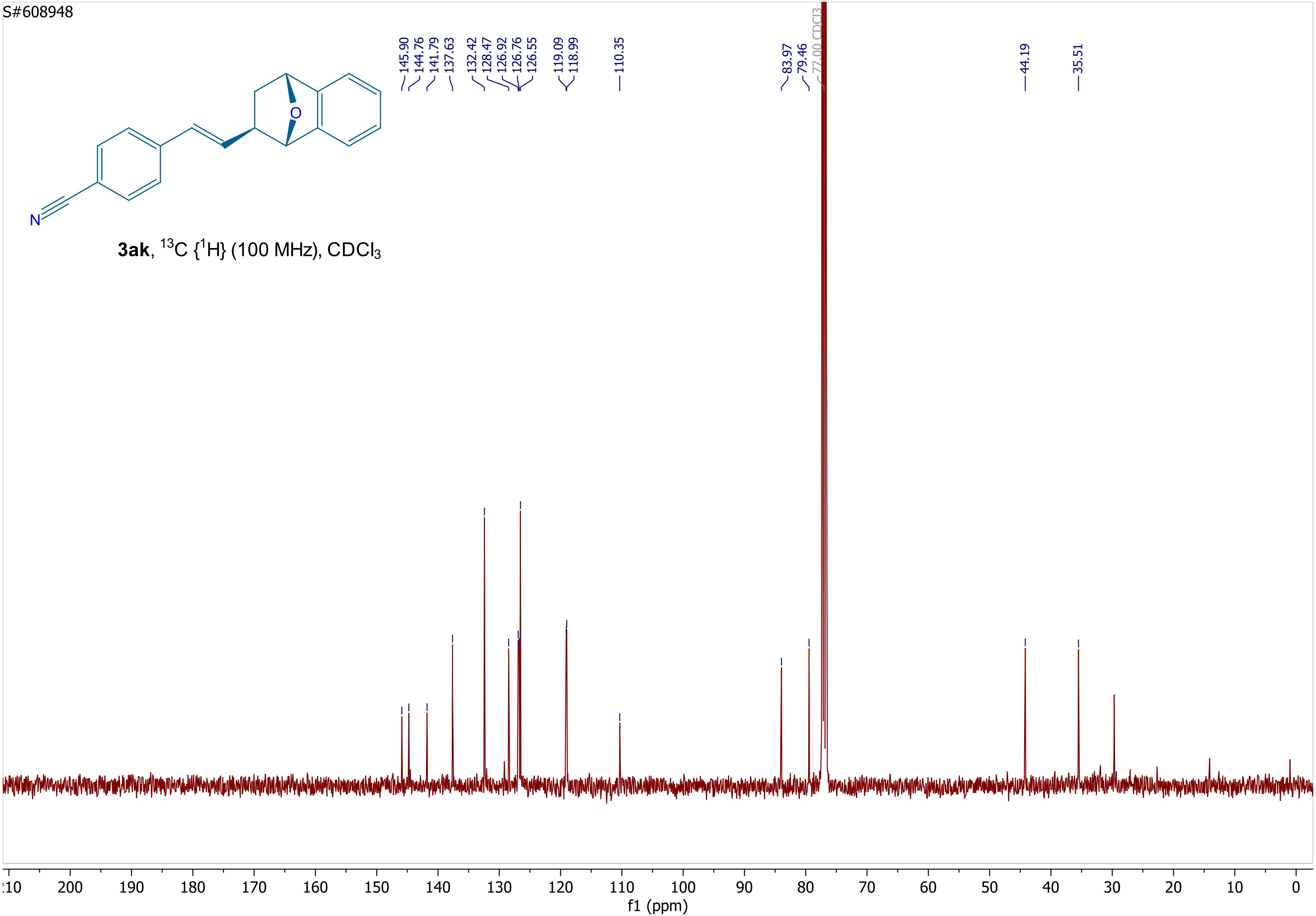

3ak, ^{13}C {^{1}H} (100 MHz), $CDCl_3$

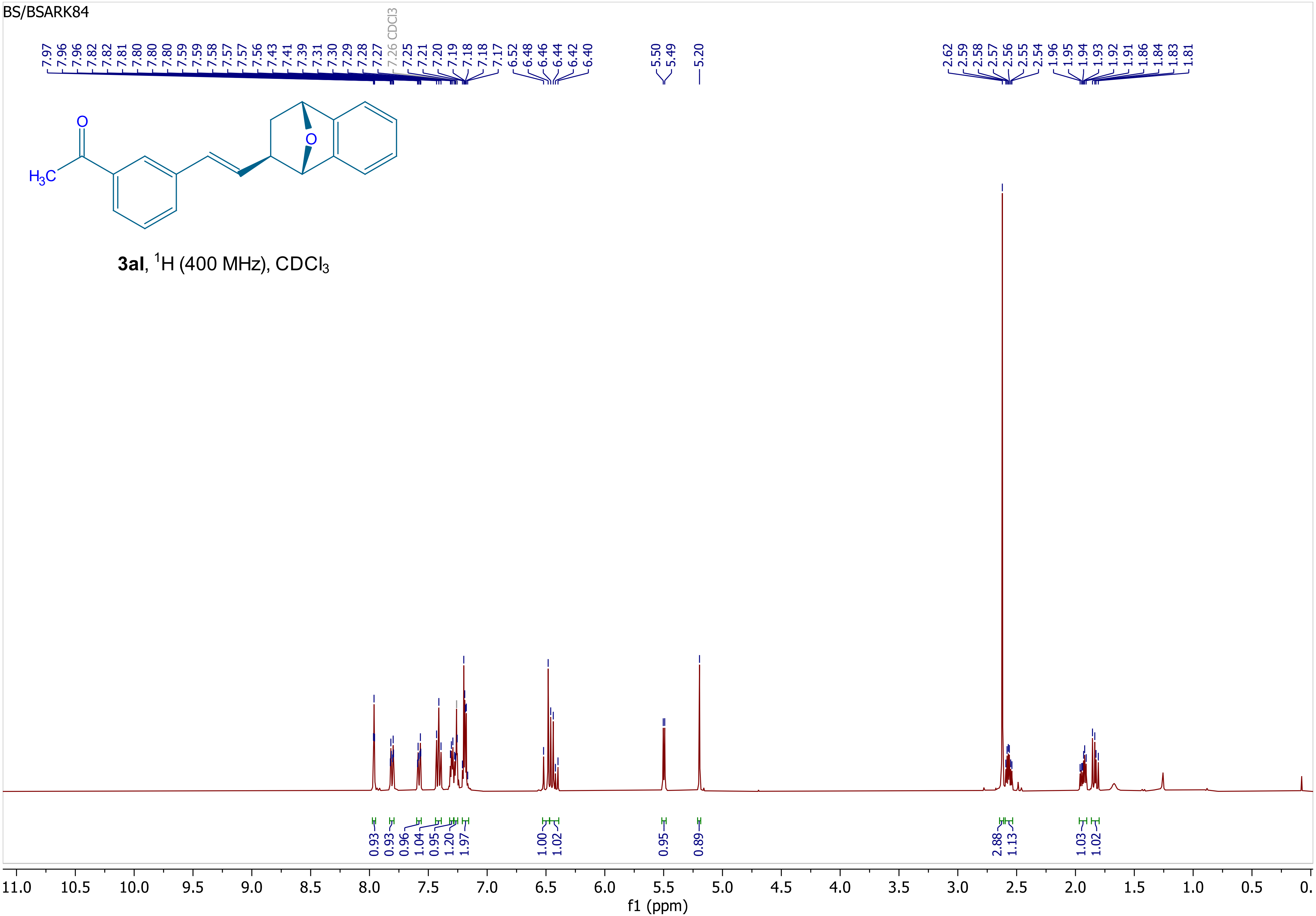

3al, ^{1}H (400 MHz), $CDCl_3$

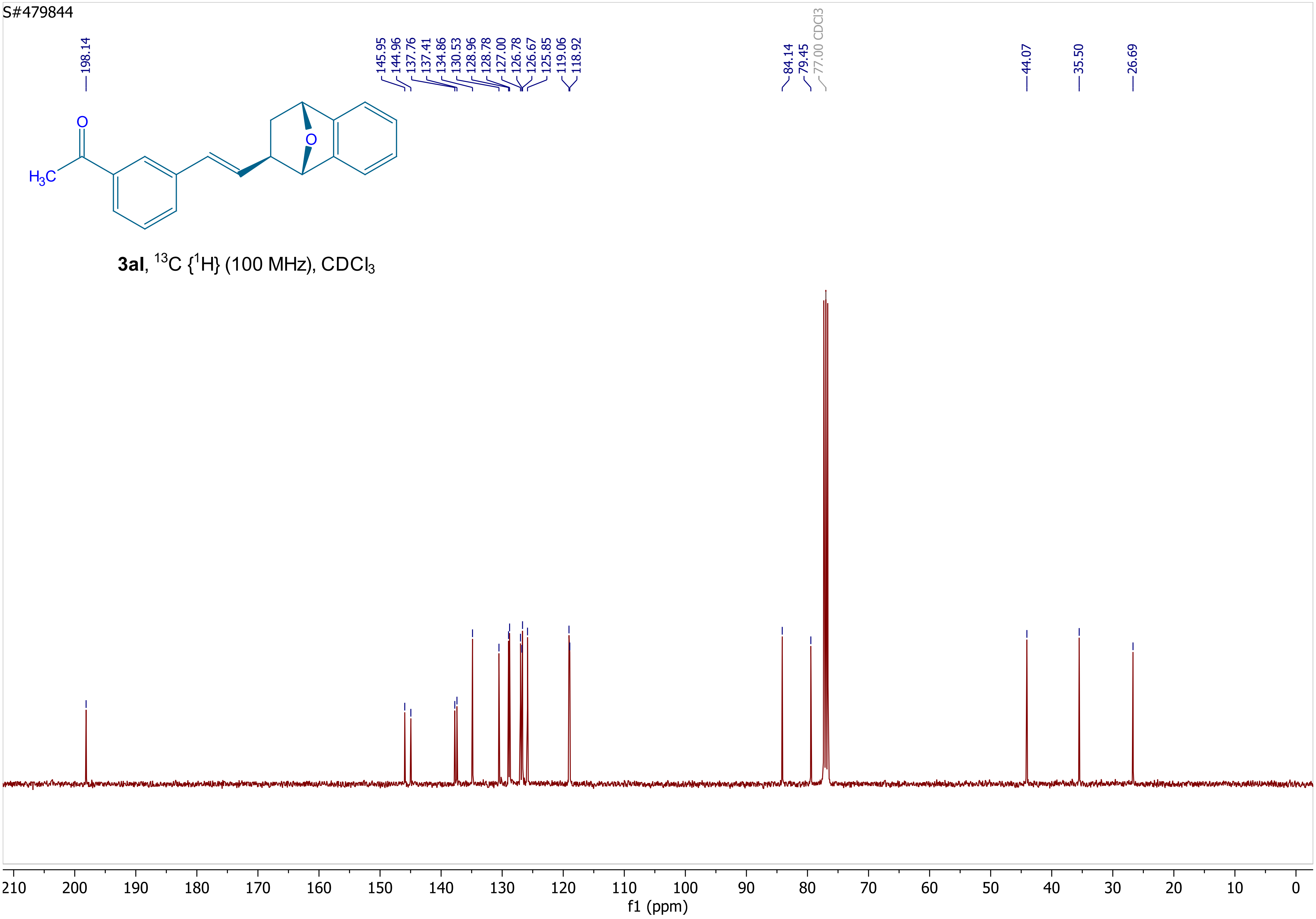

3al, $^{13}C\ \{^{1}H\}$ (100 MHz), $CDCl_3$

S#473224

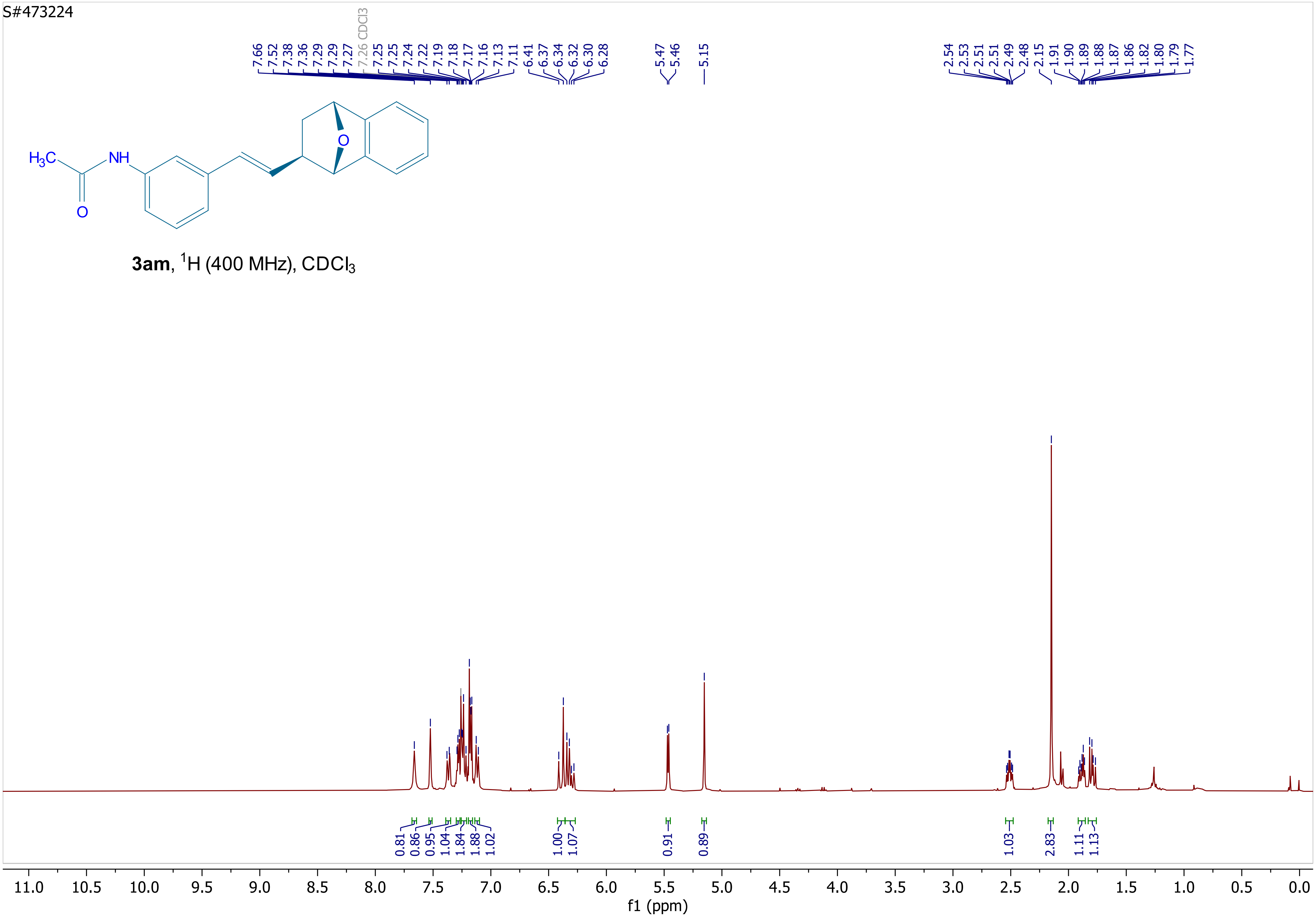

3am, ^{1}H (400 MHz), $CDCl_3$

S#749188

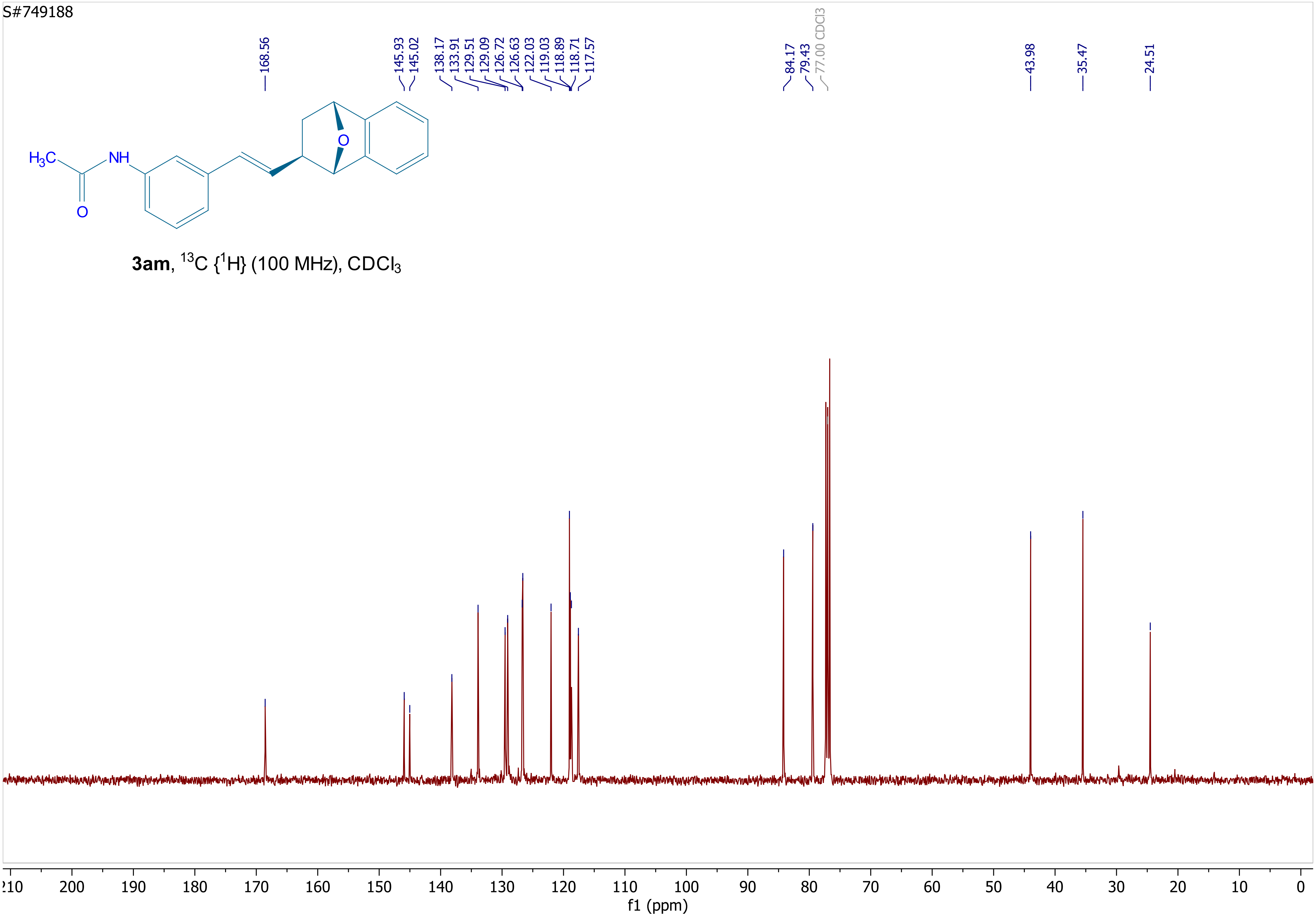

3am, $^{13}C\{^1H\}$ (100 MHz), $CDCl_3$

S#476080

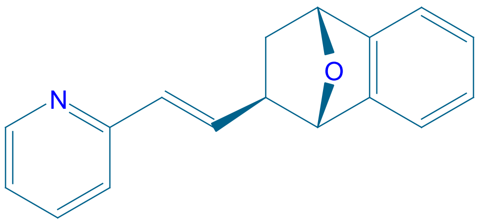

3an, ^{1}H (400 MHz), $CDCl_3$

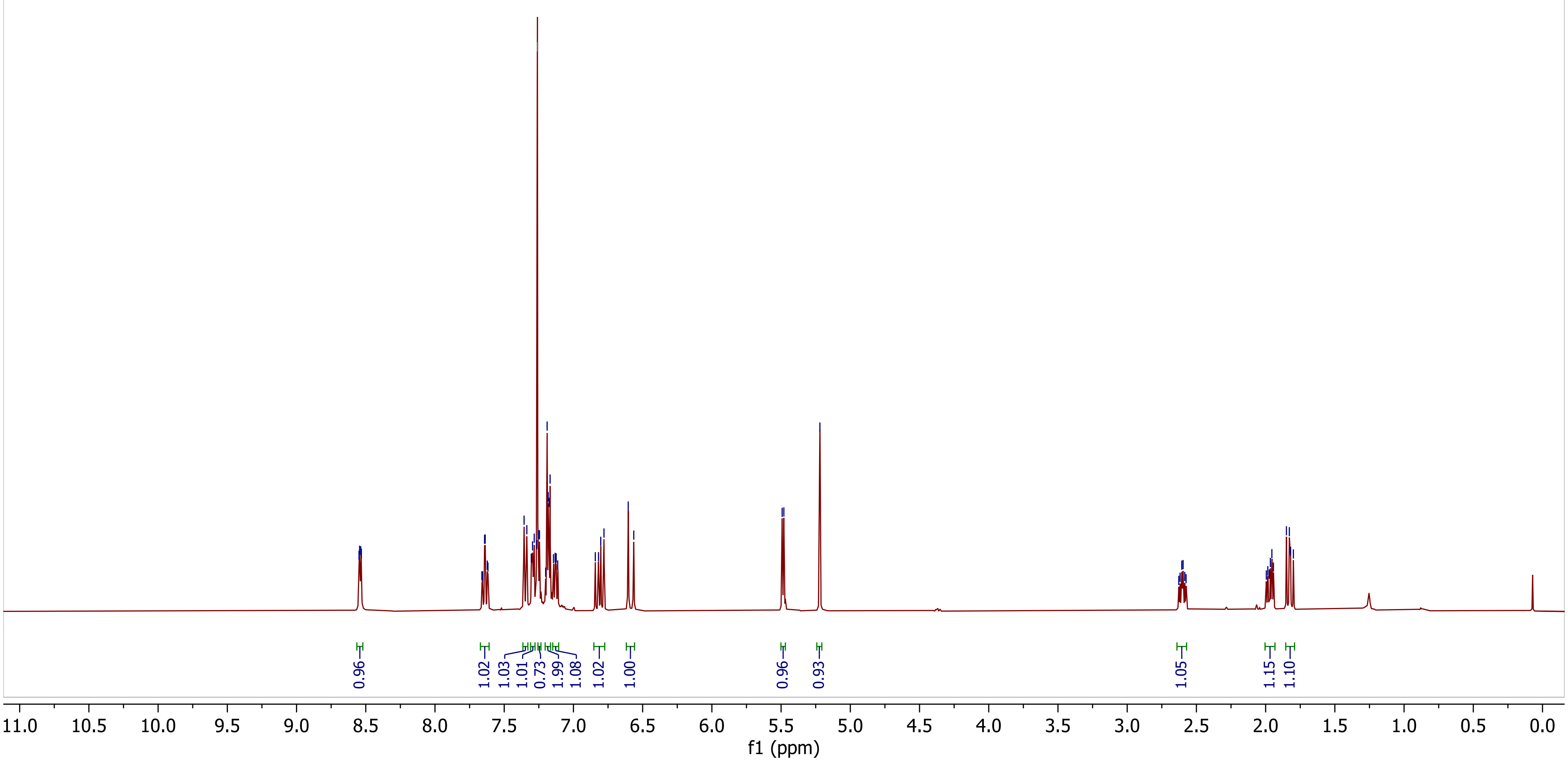

BS/BSSUP1968

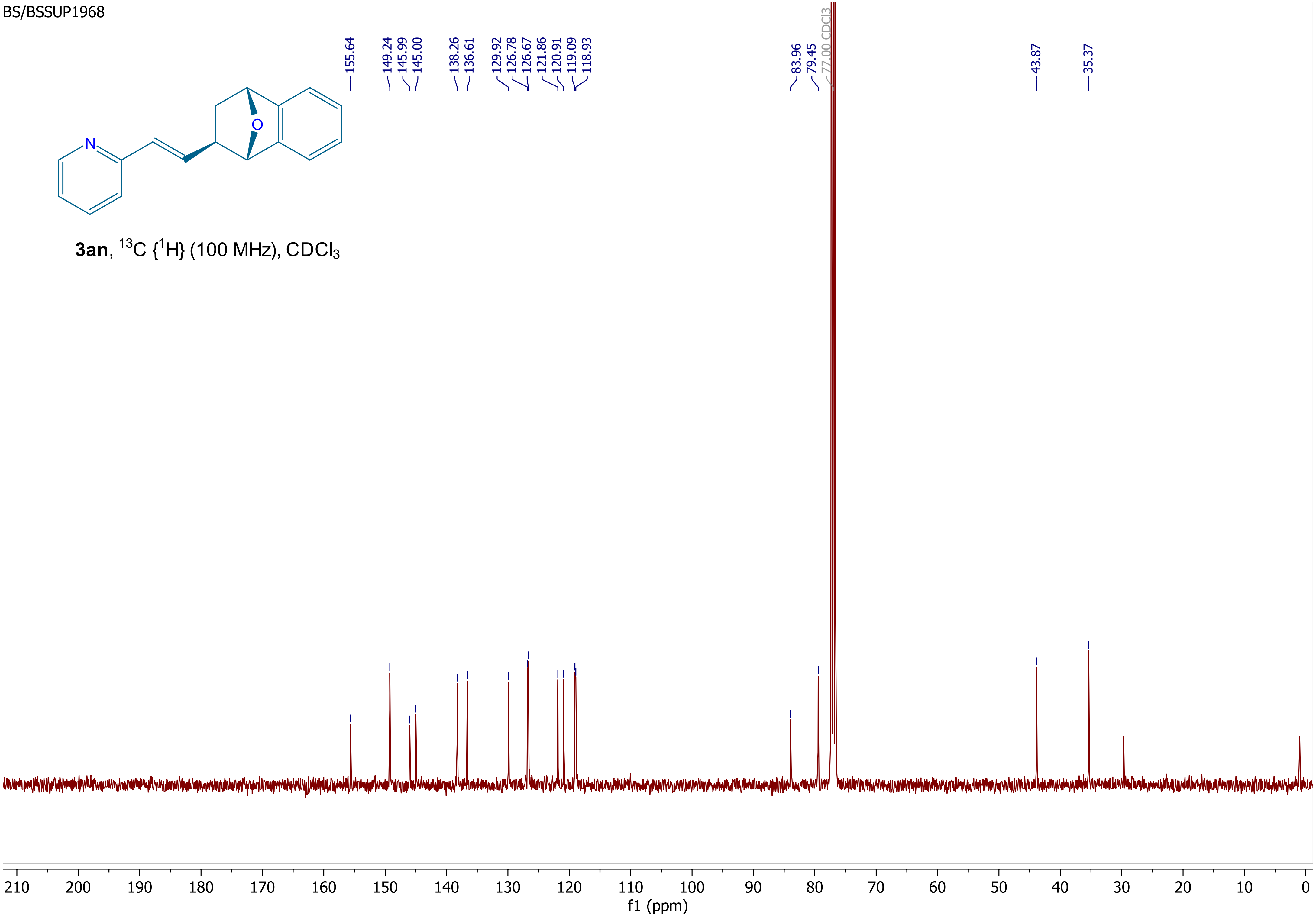

3an, $^{13}C\{^{1}H\}$ (100 MHz), $CDCl_3$

BS/BSARK99

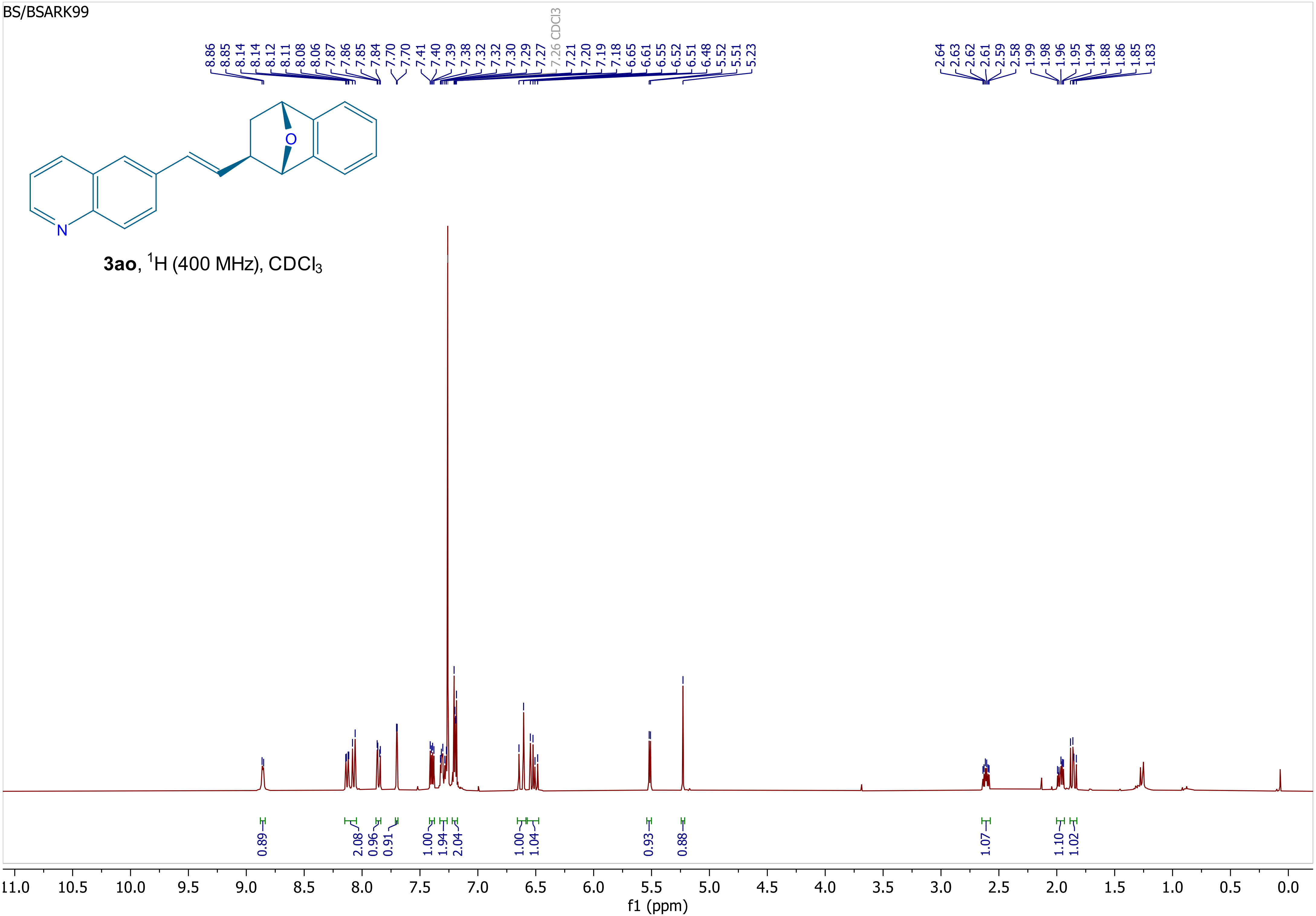

3ao, ^{1}H (400 MHz), CDCl$_3$

BS/BSARK99

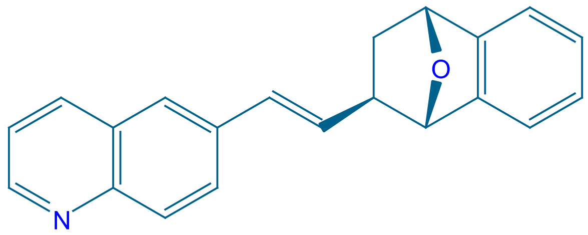

3ao, $^{13}C\{^1H\}$ (100 MHz), $CDCl_3$

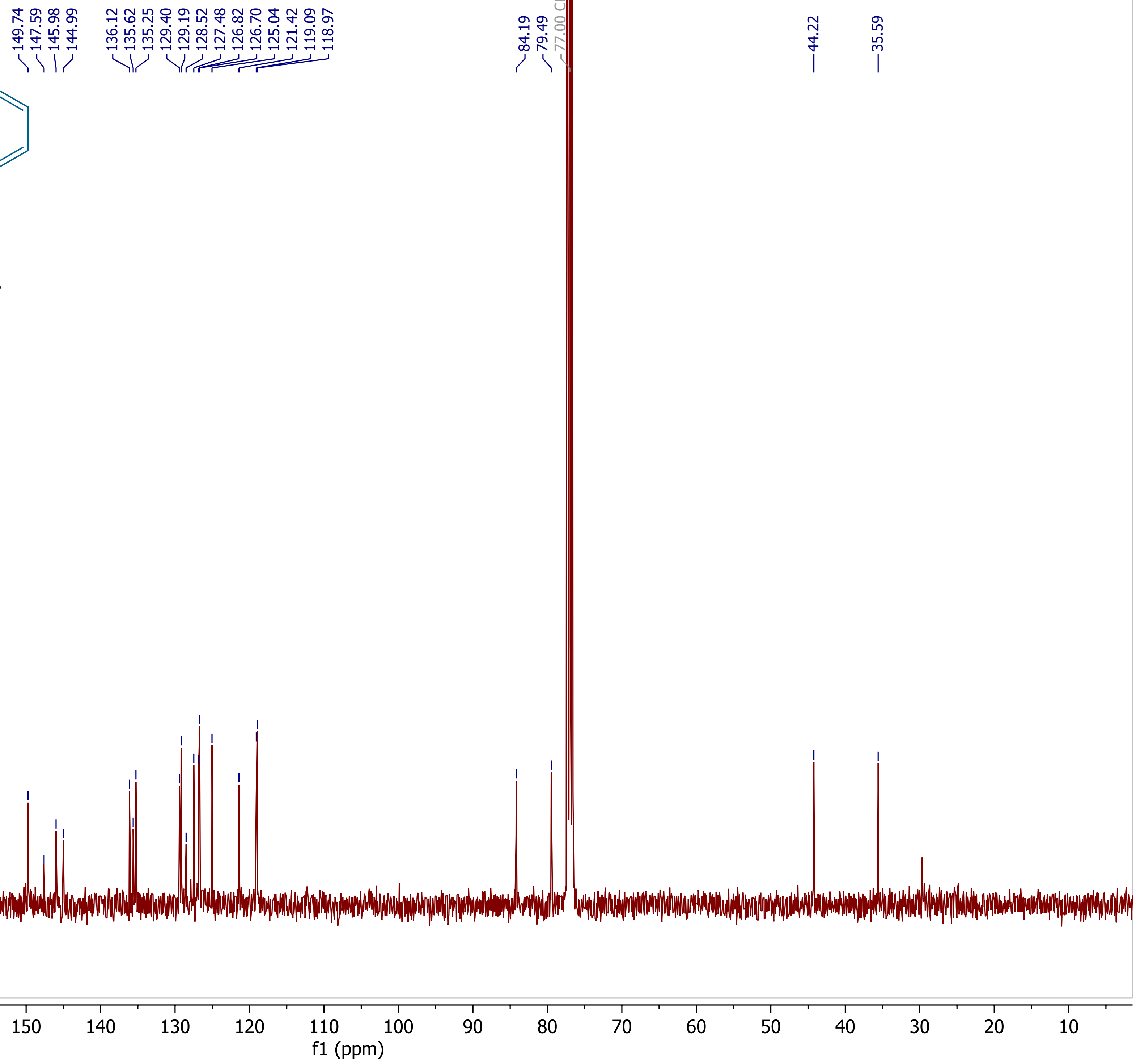

S#817840

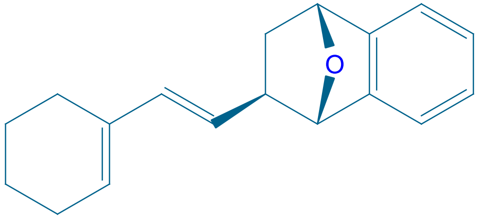

3ap, ^{1}H (400 MHz), $CDCl_3$

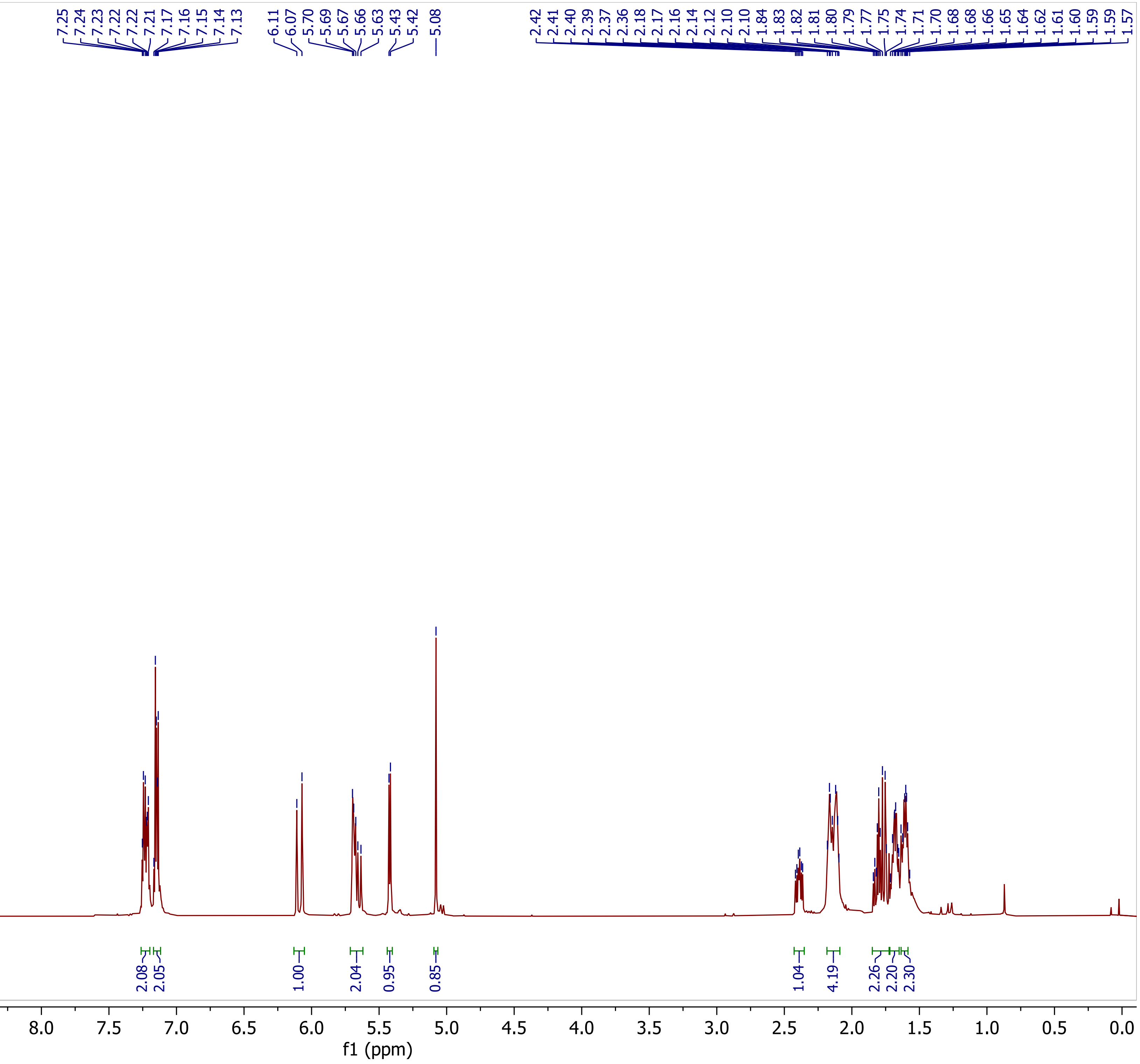

S#850966

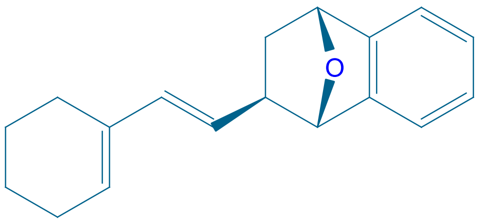

3ap, $^{13}C\ \{^{1}H\}$ (100 MHz), $CDCl_3$

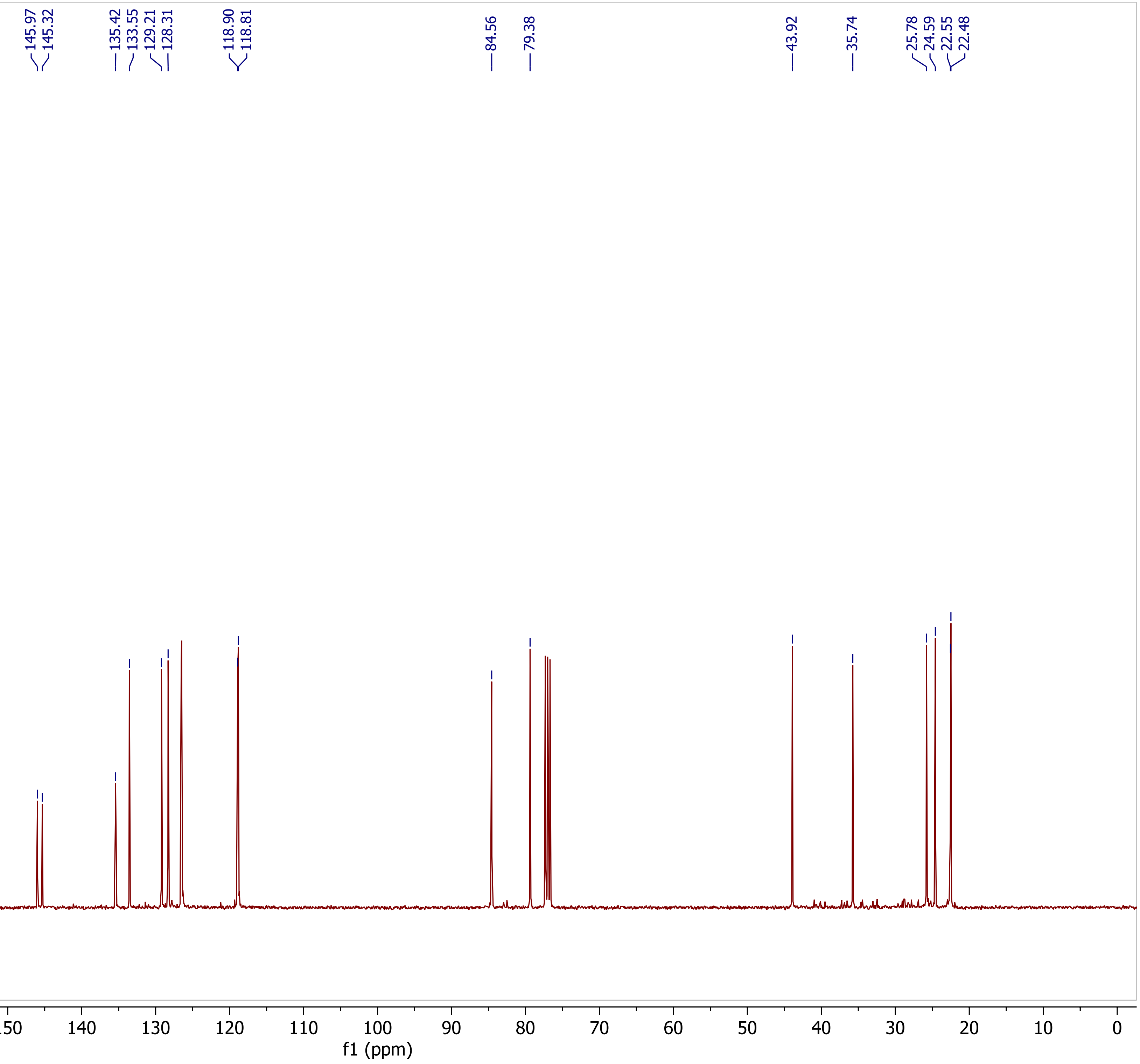

S#734674

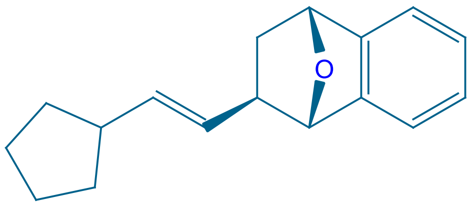

3aq, ^{1}H (400 MHz), $CDCl_3$

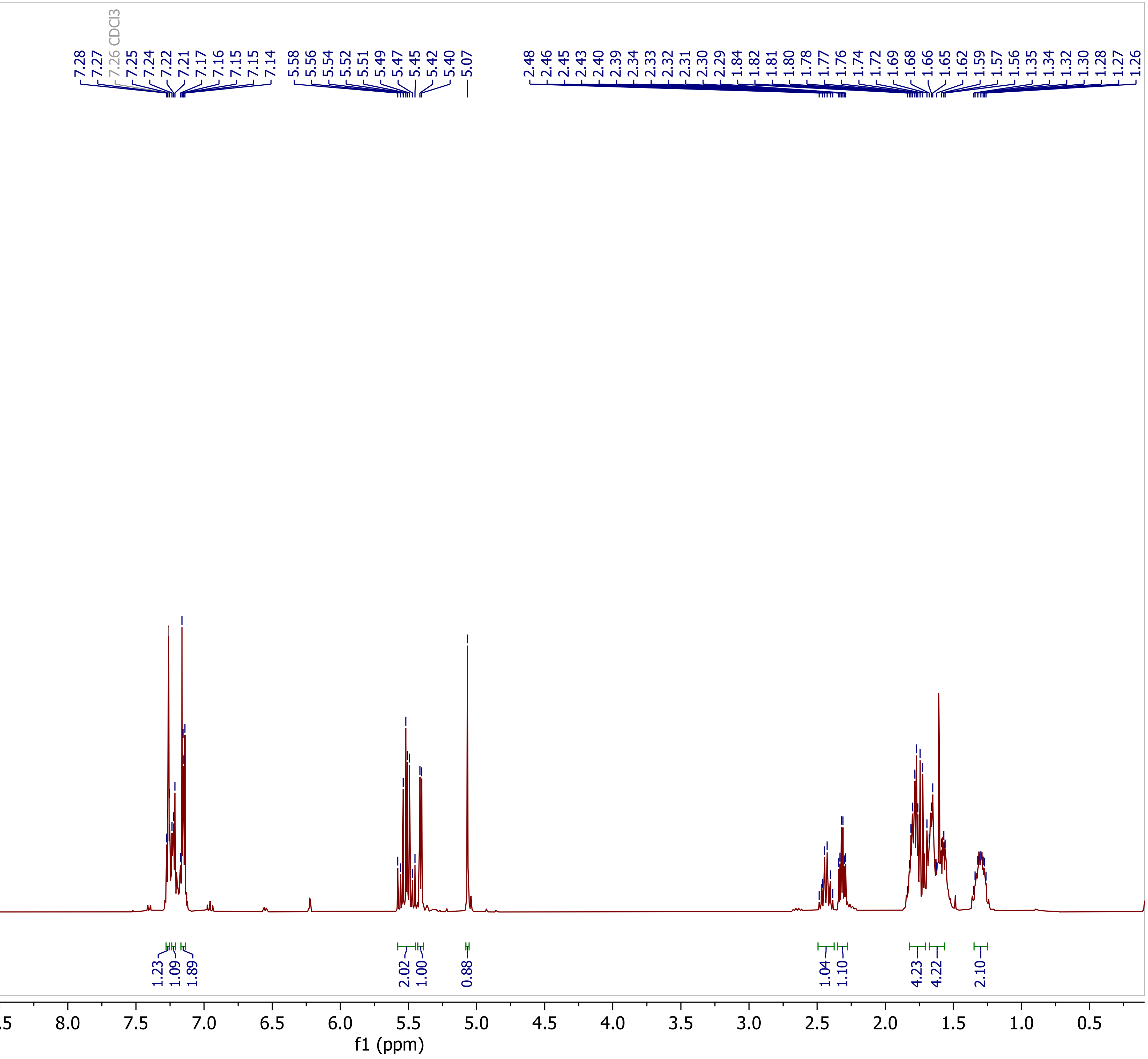

S#592604

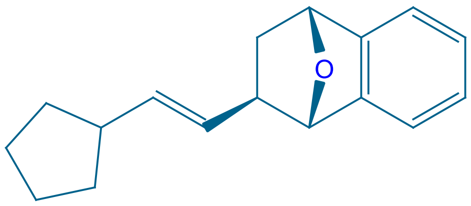

3aq, $^{13}C\ \{^1H\}$ (100 MHz), $CDCl_3$

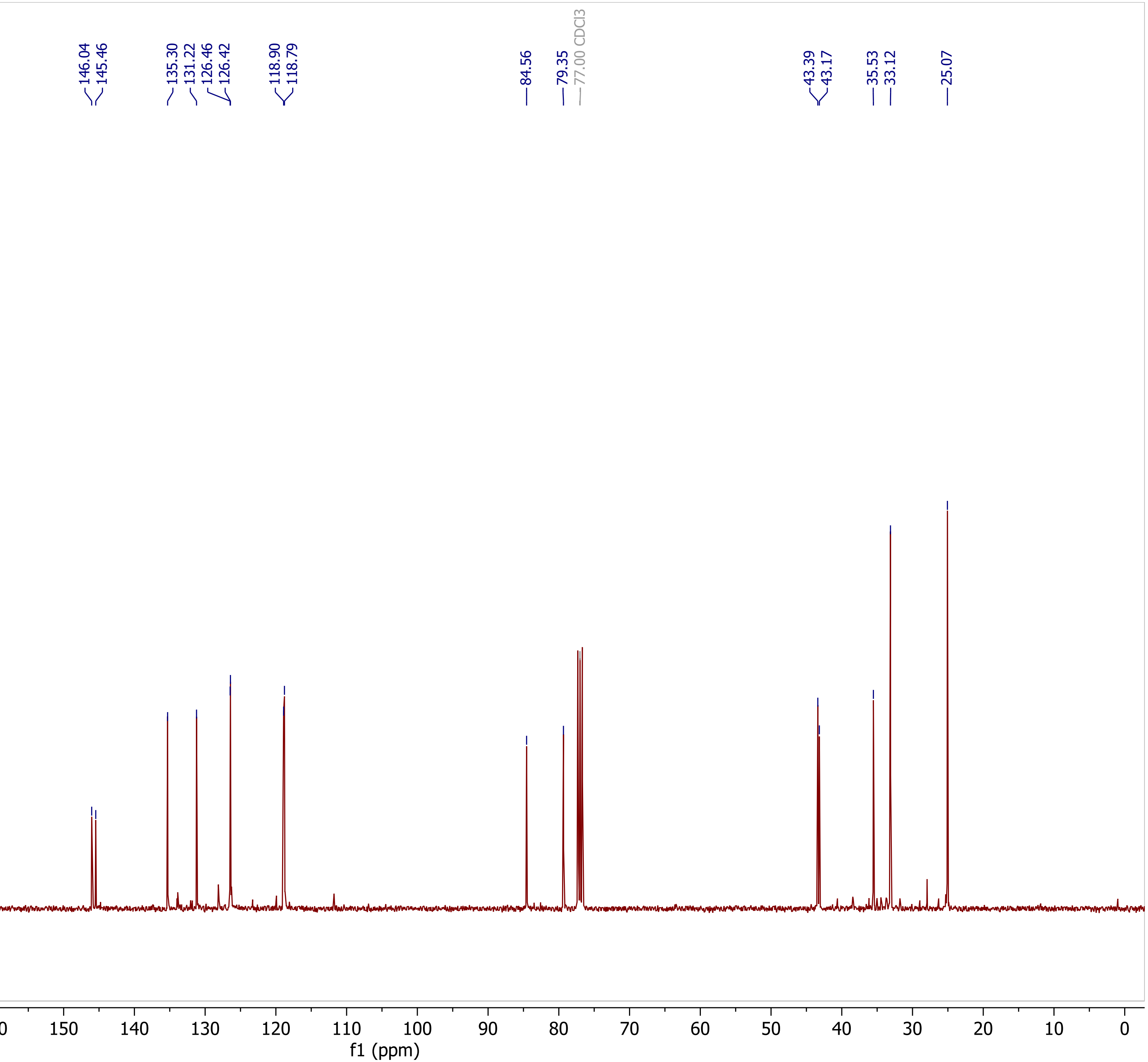

S#730702

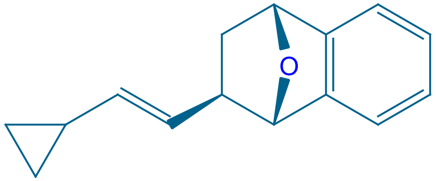

3ar, ^{1}H (400 MHz), $CDCl_3$

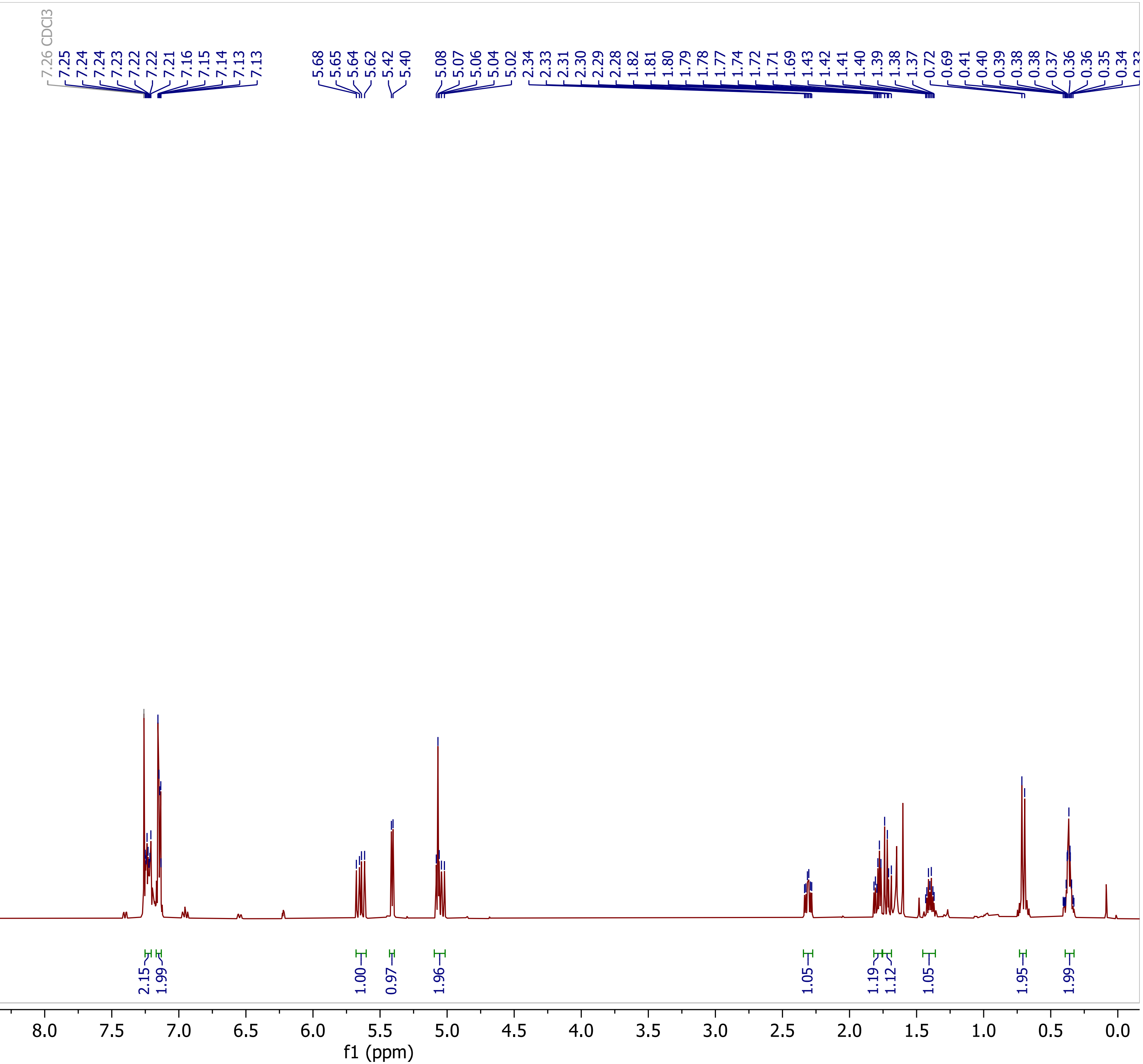

S#498777

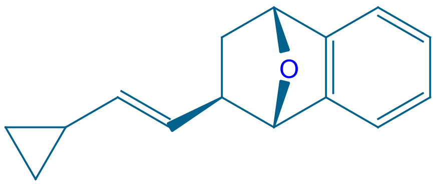

3ar, $^{13}C\ \{^{1}H\}$ (100 MHz), $CDCl_3$

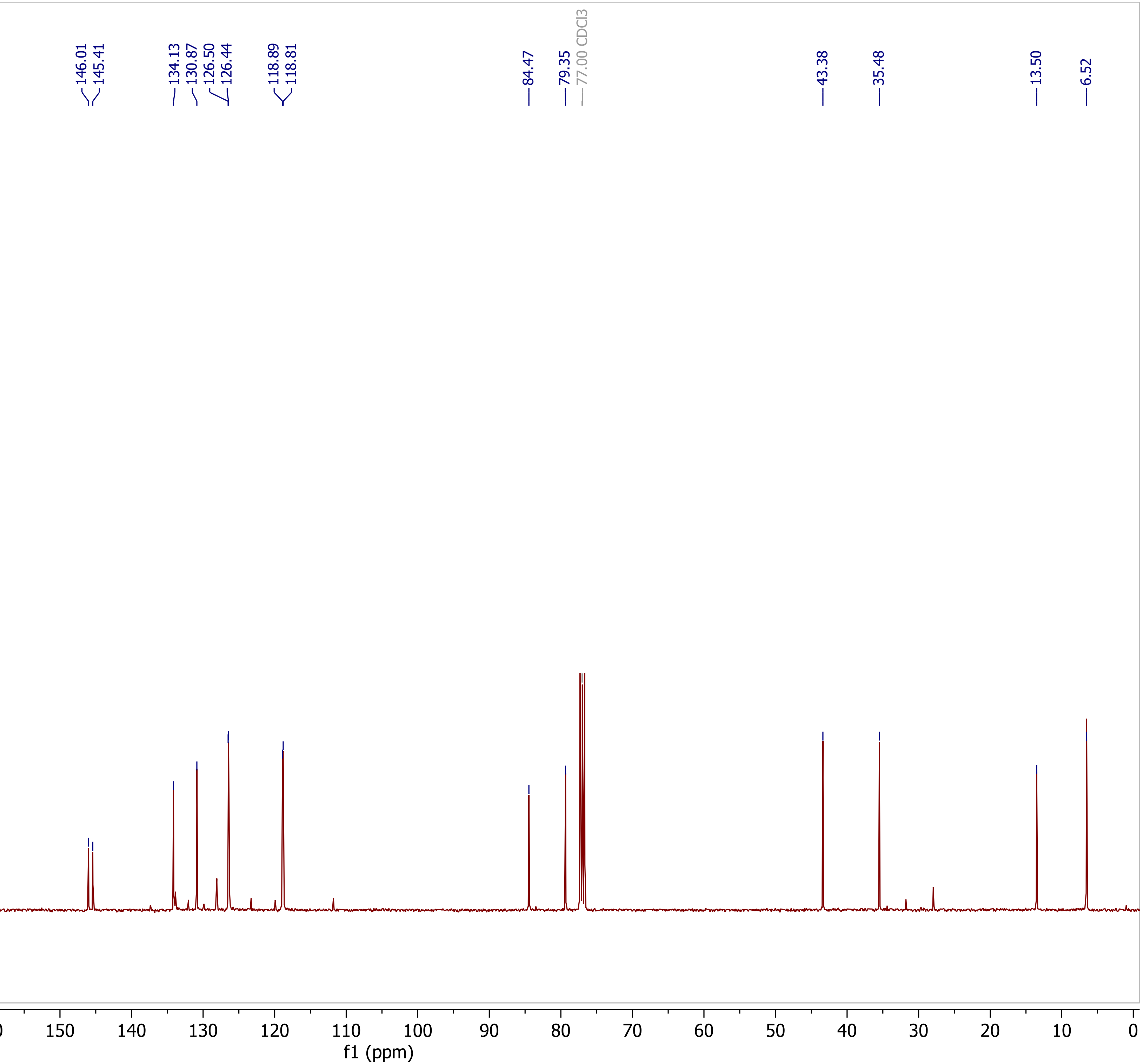

S#354069

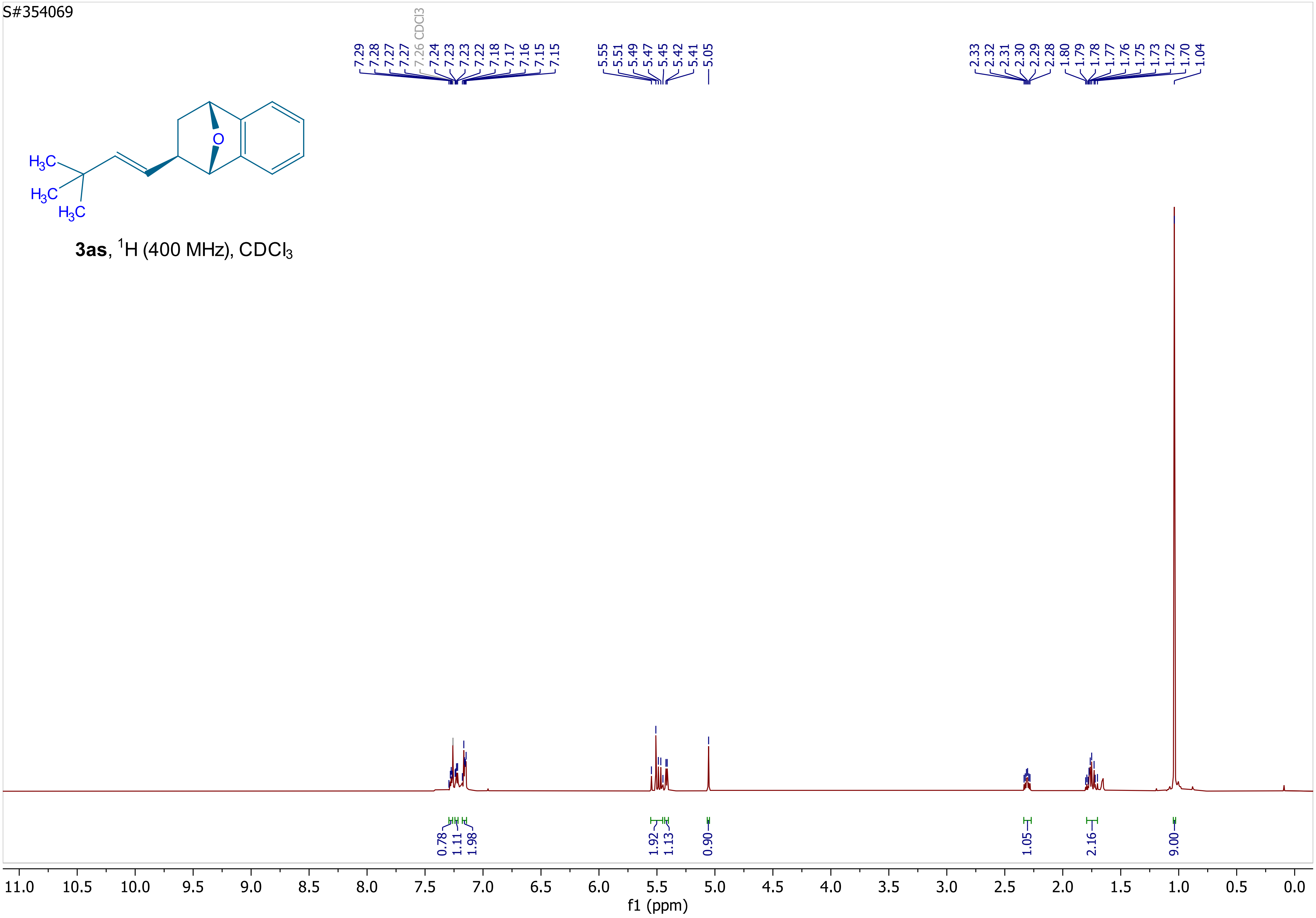

3as, ^{1}H (400 MHz), $CDCl_3$

S#444406

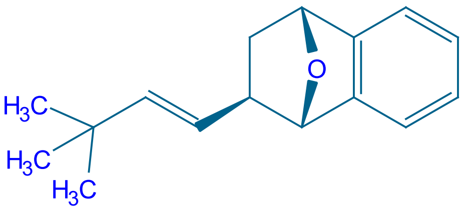

3as, ^{13}C {^{1}H} (100 MHz), $CDCl_3$

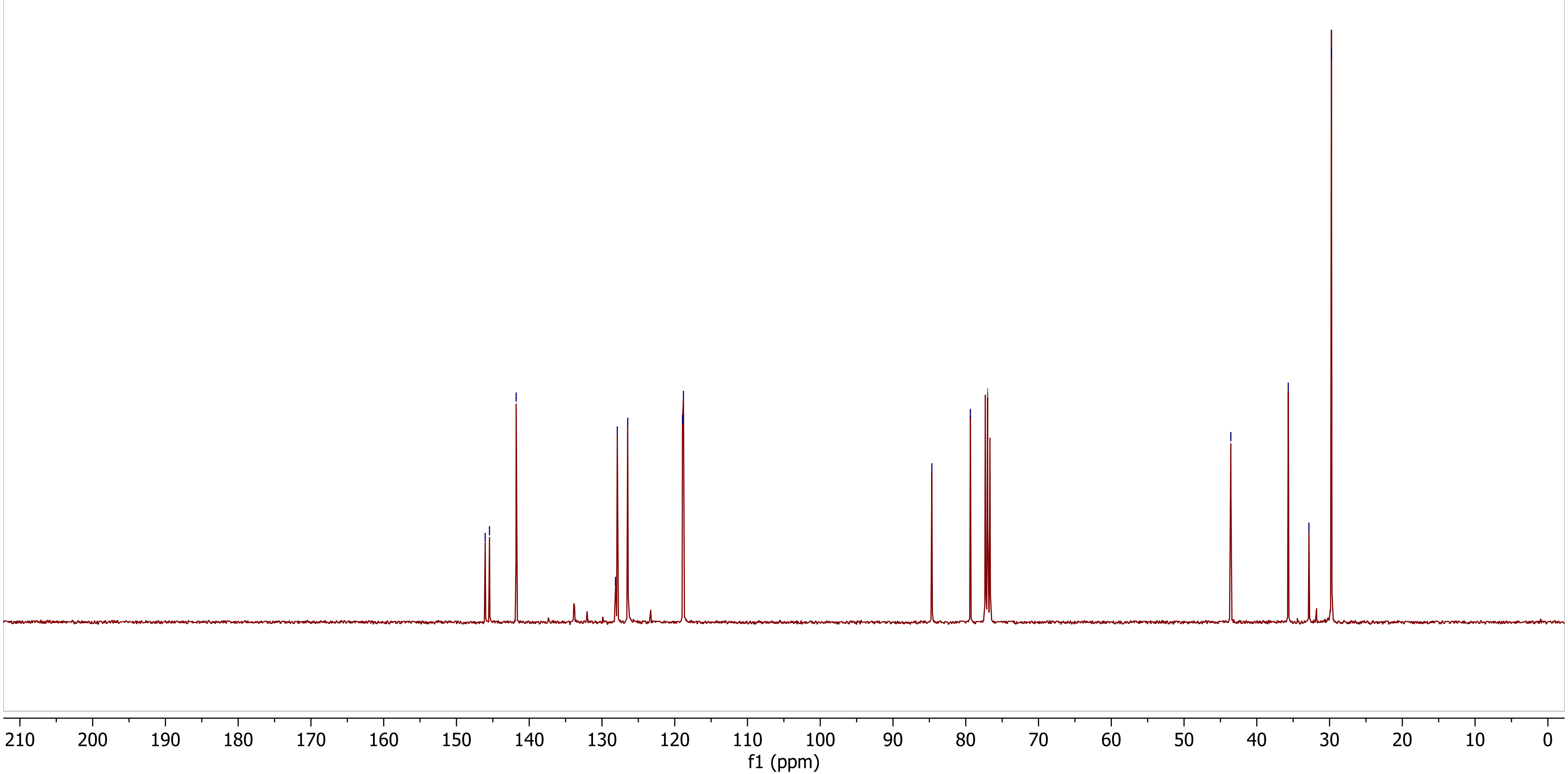

BSSUP1804

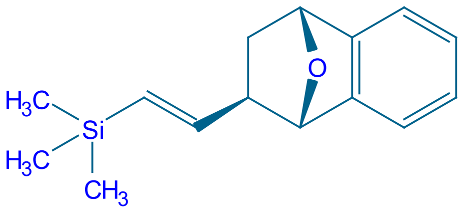

3at, ^{1}H (400 MHz), $CDCl_3$

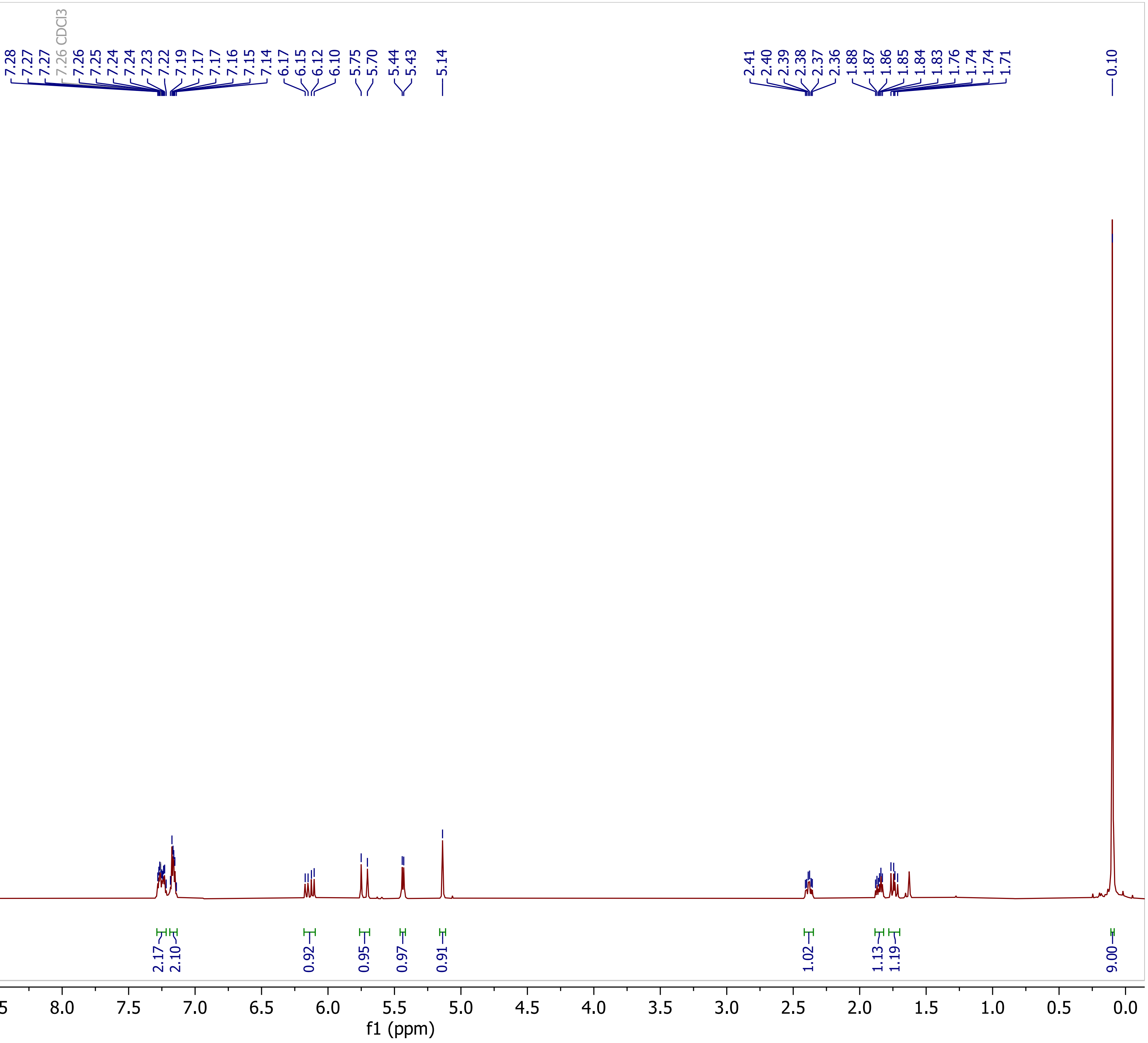

S#109079

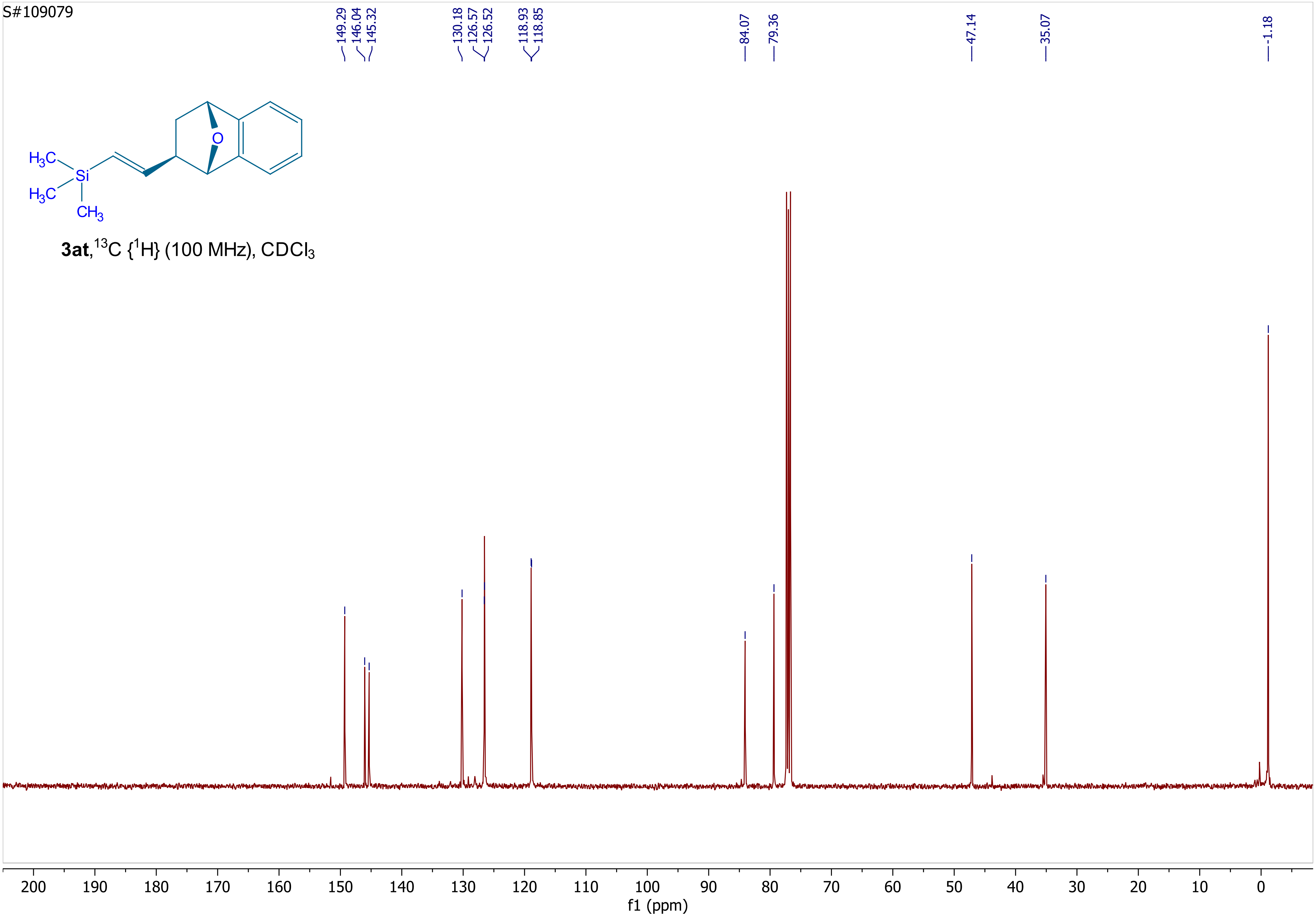

149.29
146.04
145.32
130.18
126.57
126.52
118.93
118.85
84.07
79.36
47.14
35.07
-1.18
H3C
H3C
Si
CH3
O
3at, 13C {1H} (100 MHz), CDCl3
200
190
180
170
160
150
140
130
120
110
100
90
80
70
60
50
40
30
20
10
0
f1 (ppm)

S#692101

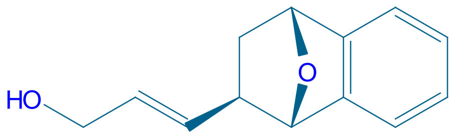

4aa, ^{1}H (400 MHz), $CDCl_3$

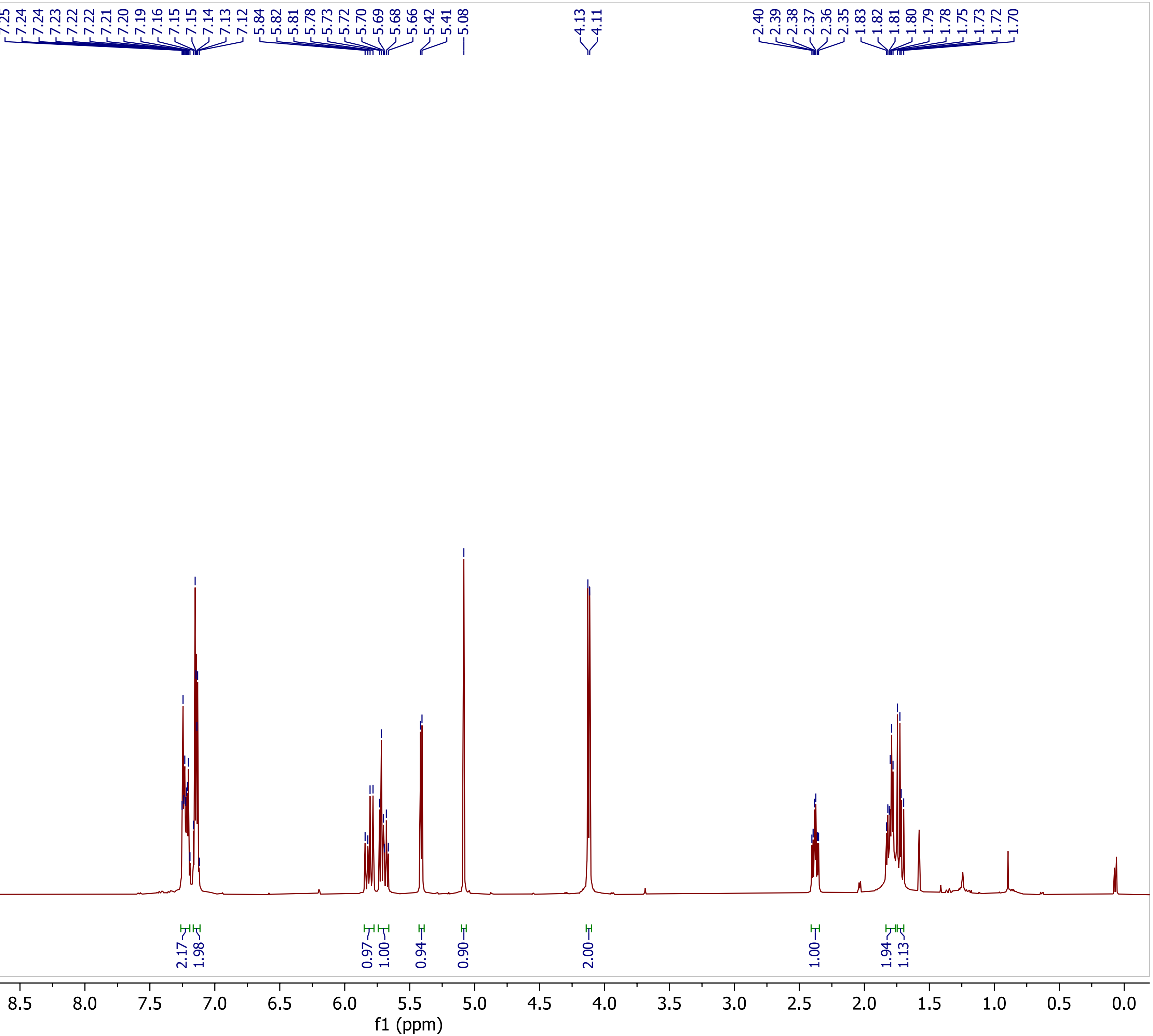

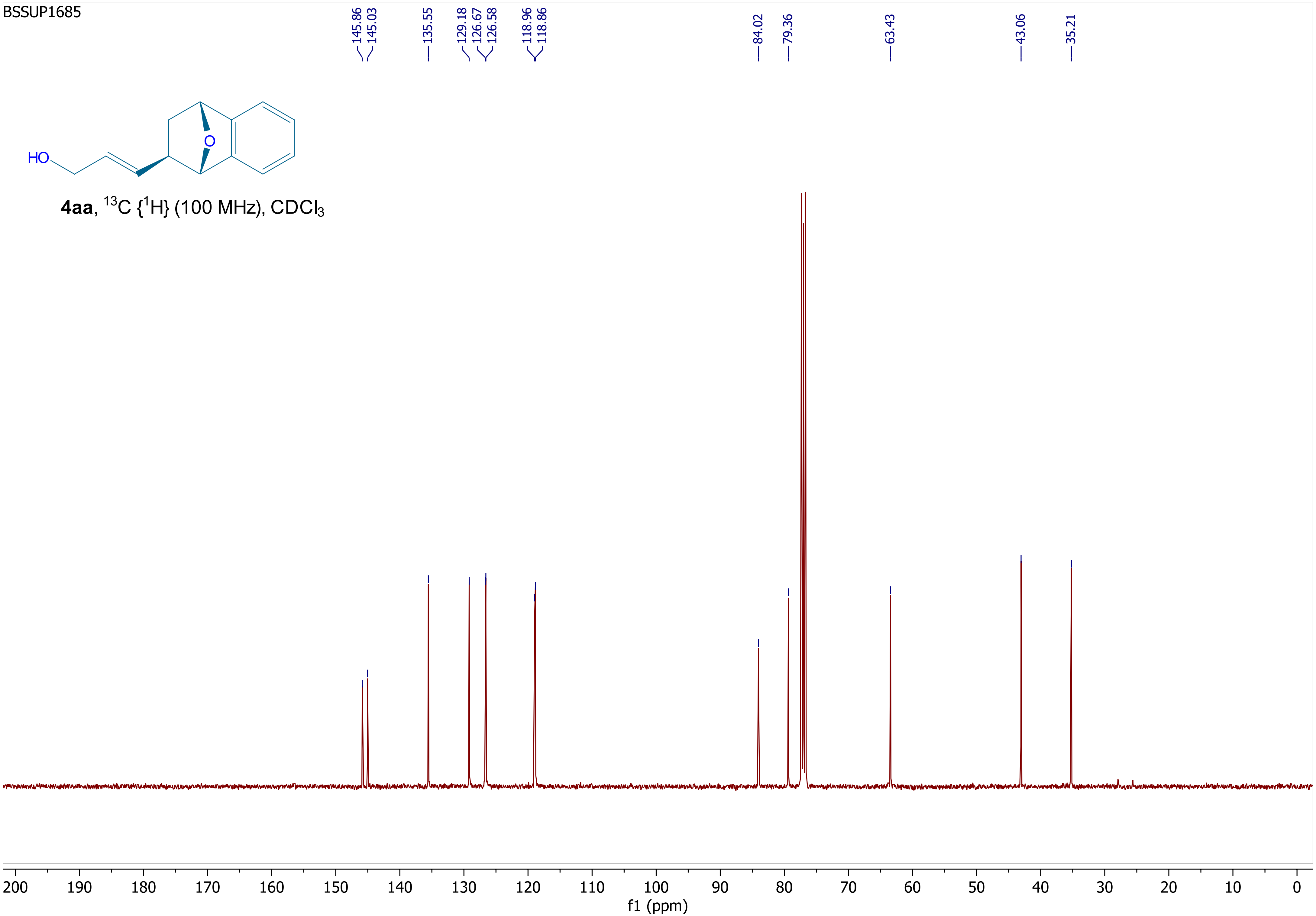
BSSUP1685
145.86
145.03
135.55
129.18
126.67
126.58
118.96
118.86
84.02
79.36
63.43
43.06
35.21
HO
O
4aa, 13C {1H} (100 MHz), CDCl3
200 190 180 170 160 150 140 130 120 110 100 90 80 70 60 50 40 30 20 10 0
f1 (ppm)

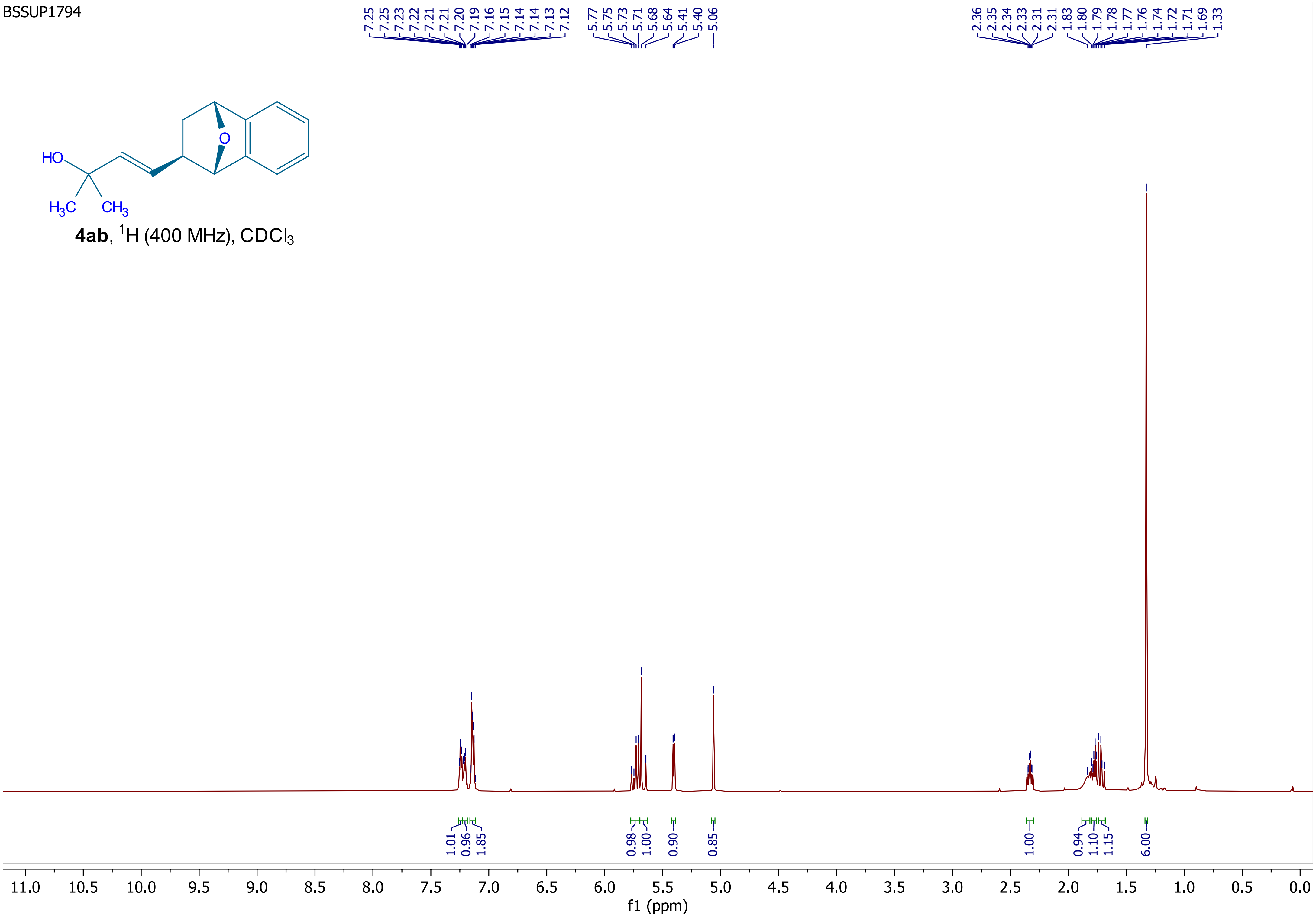
BSSUP1794
7.25 7.25 7.23 7.22 7.21 7.21 7.20 7.19 7.16 7.15 7.14 7.14 7.13 7.12
5.77 5.75 5.73 5.71 5.68 5.64 5.41 5.40 5.06
2.36 2.35 2.34 2.33 2.31 2.31 1.83 1.80 1.79 1.78 1.77 1.76 1.74 1.72 1.71 1.69 1.33
HO
O
H3C
CH3
4ab, 1H (400 MHz), CDCl3
1.01 0.96 1.85
0.98 1.00
0.90
0.85
1.00
0.94 1.10 1.15
6.00
11.0 10.5 10.0 9.5 9.0 8.5 8.0 7.5 7.0 6.5 6.0 5.5 5.0 4.5 4.0 3.5 3.0 2.5 2.0 1.5 1.0 0.5 0.0
f1 (ppm)

BSSUP1795

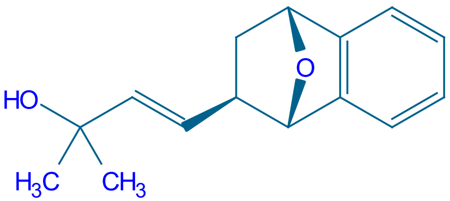

4ab, ^{13}C {^{1}H} (100 MHz), $CDCl_3$

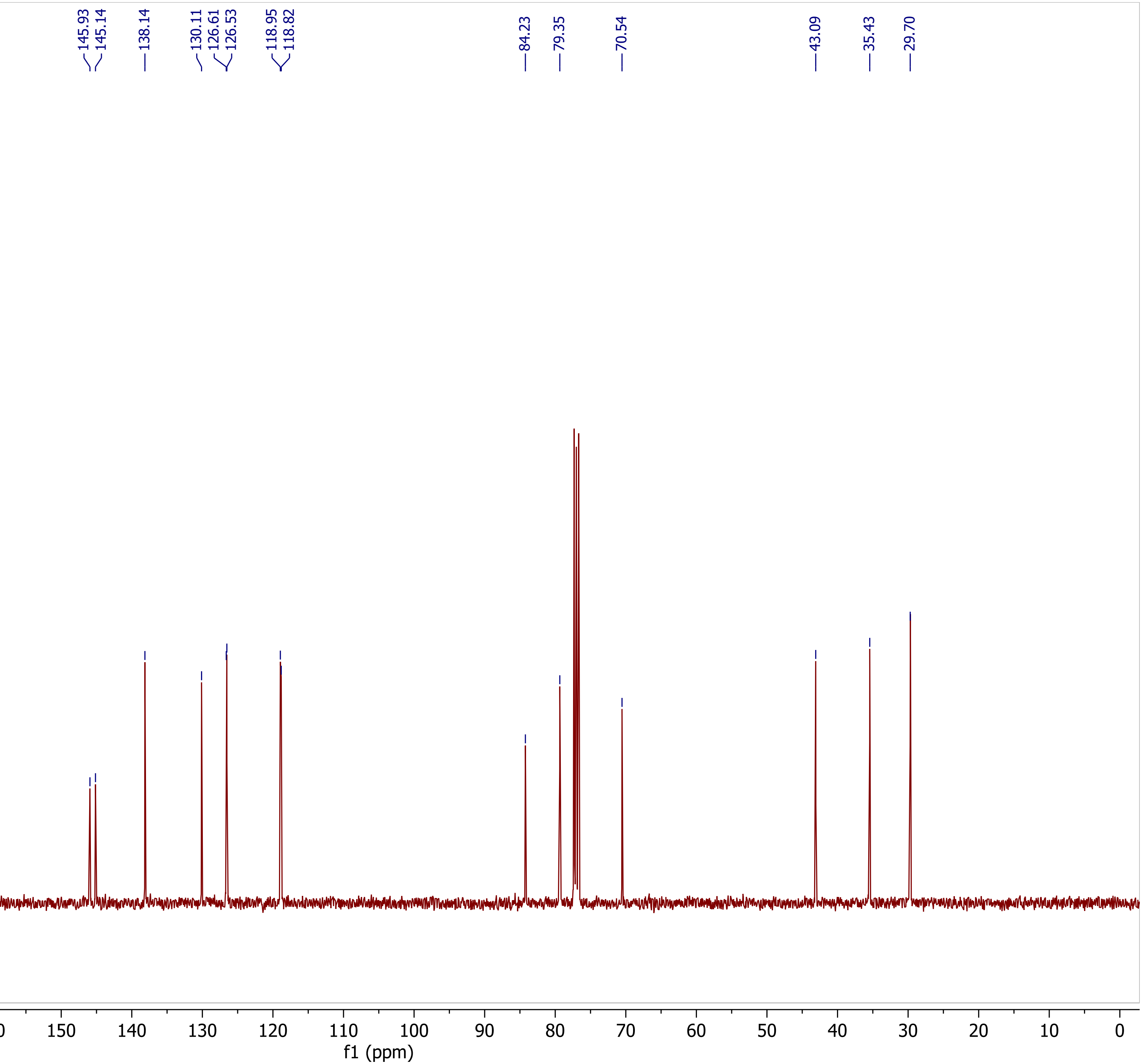

S#378419

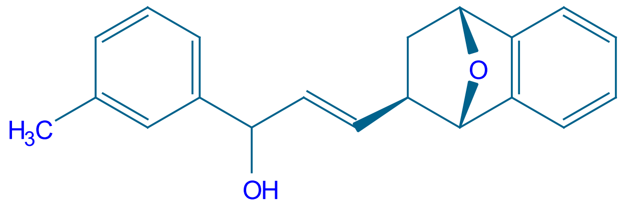

4ac, ¹H (400 MHz), $CDCl_3$

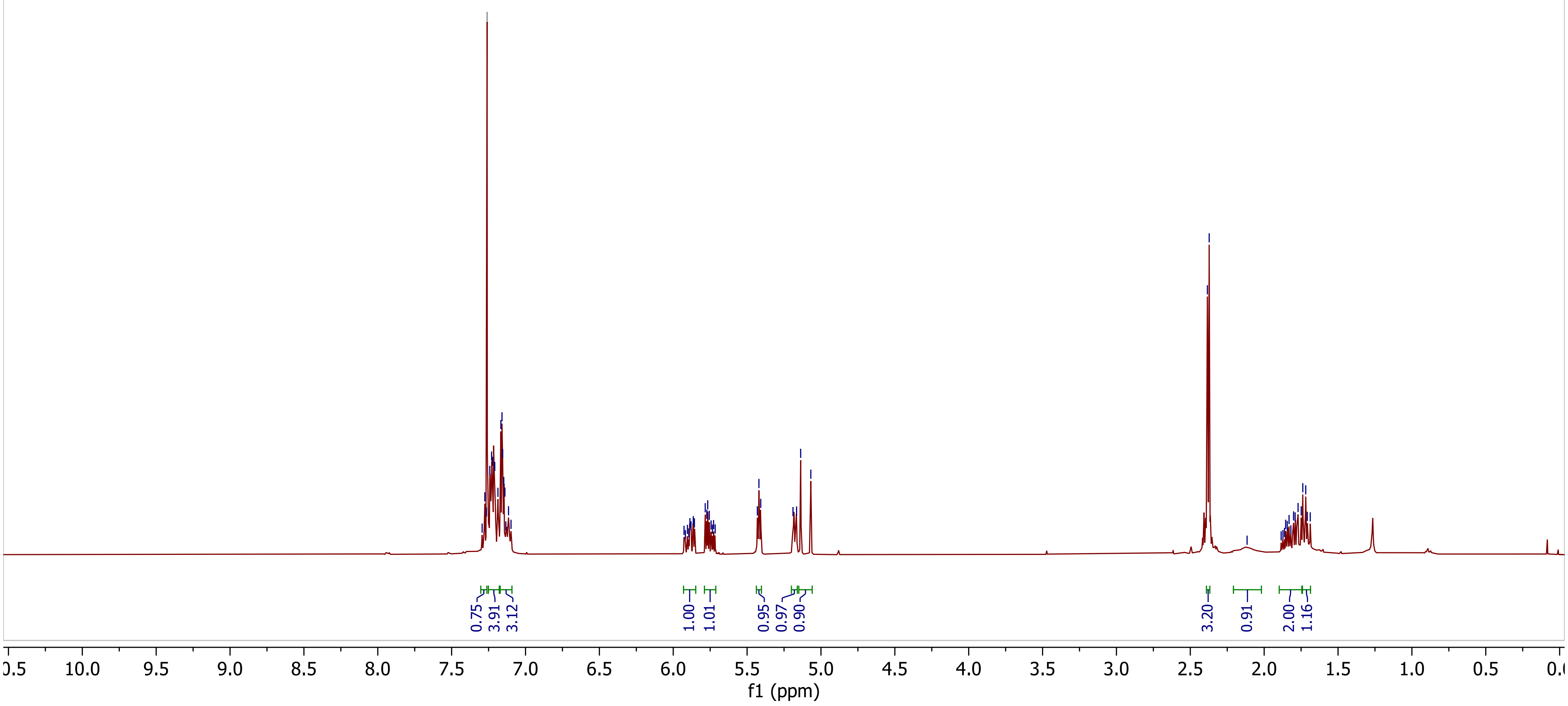

S#162297

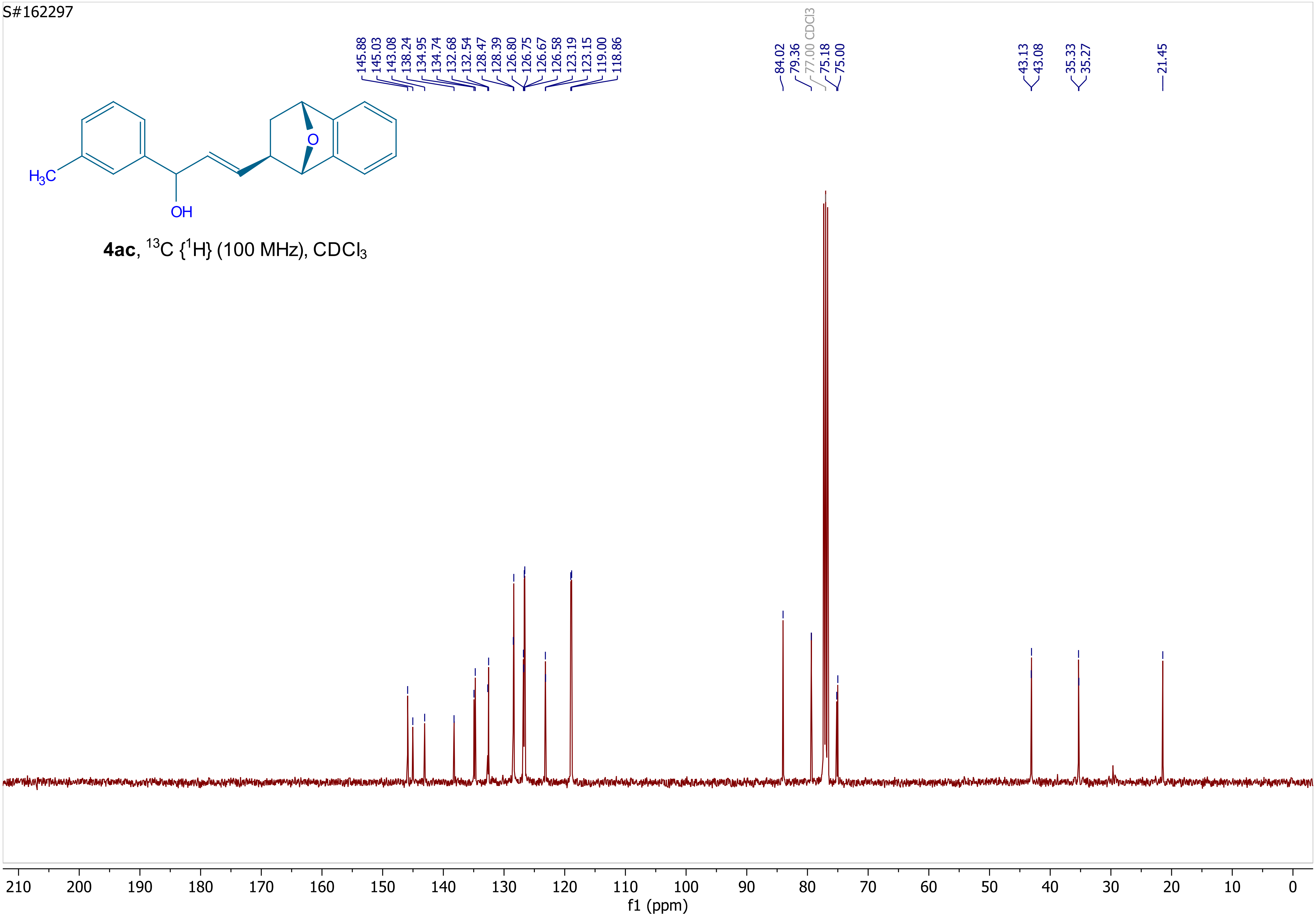

4ac, ^{13}C {^{1}H} (100 MHz), $CDCl_3$

S#443902

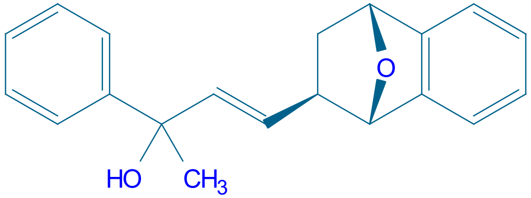

4ad, ^{1}H (400 MHz), $CDCl_3$

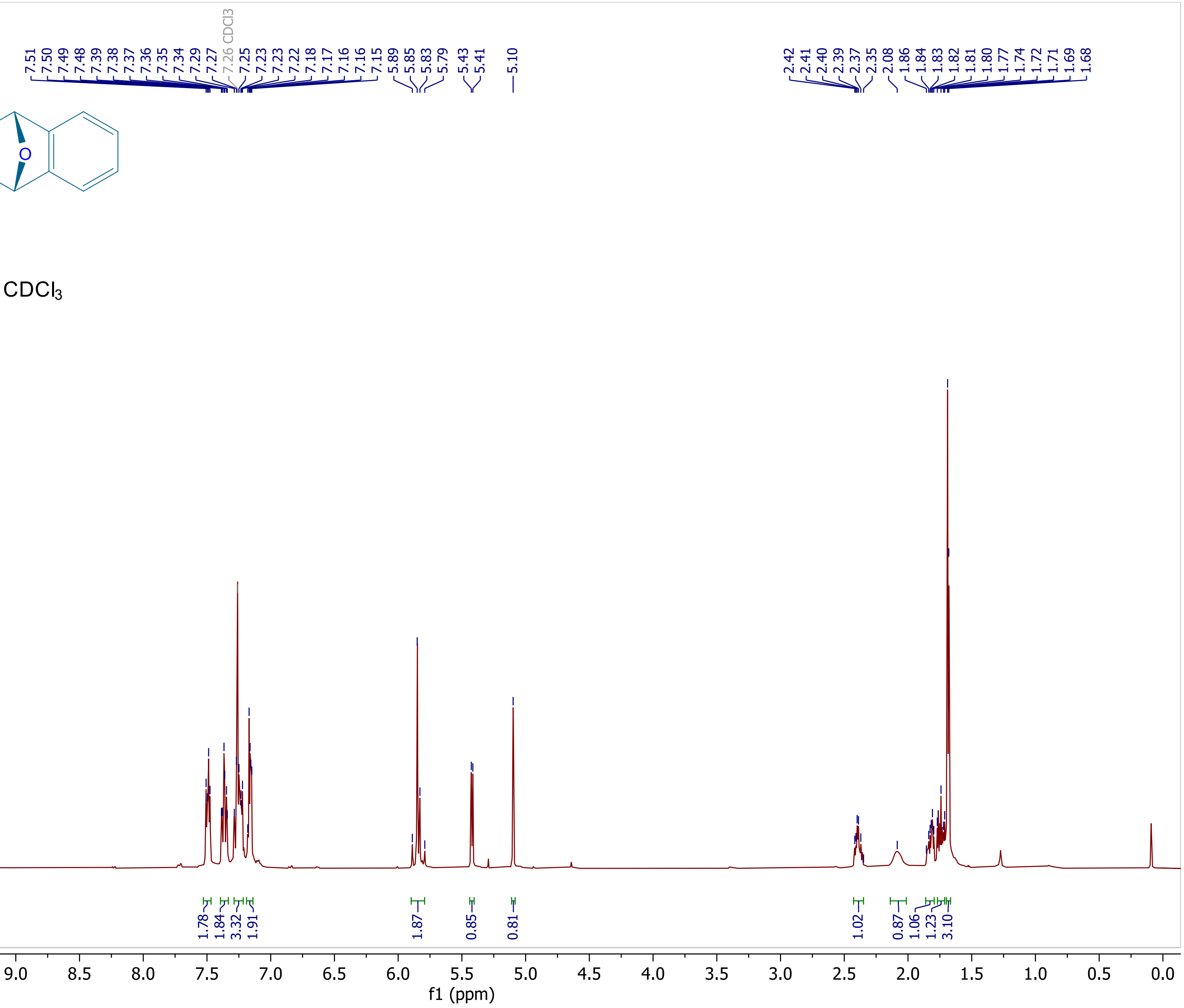

S#620325

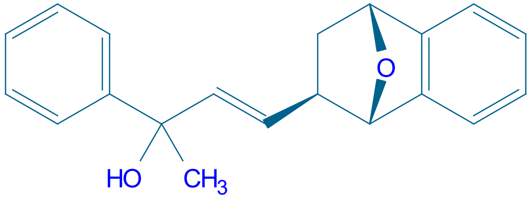

4ad, $^{13}C\{^{1}H\}$ (100 MHz), $CDCl_3$

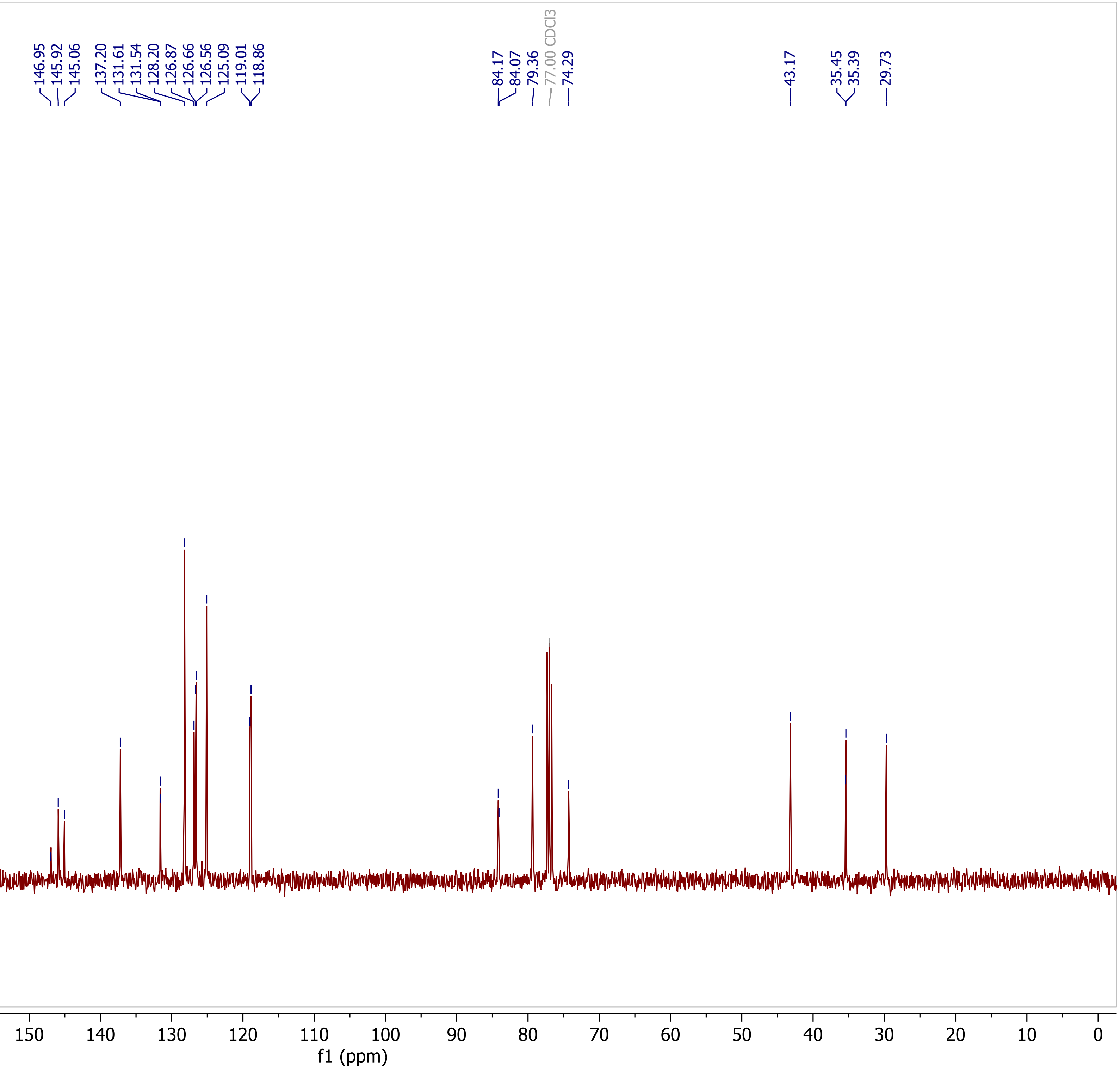

S#471149

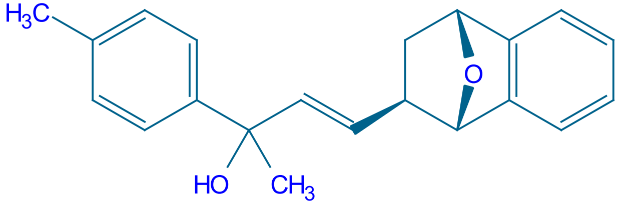

4ae, ^{1}H (400 MHz), $CDCl_3$

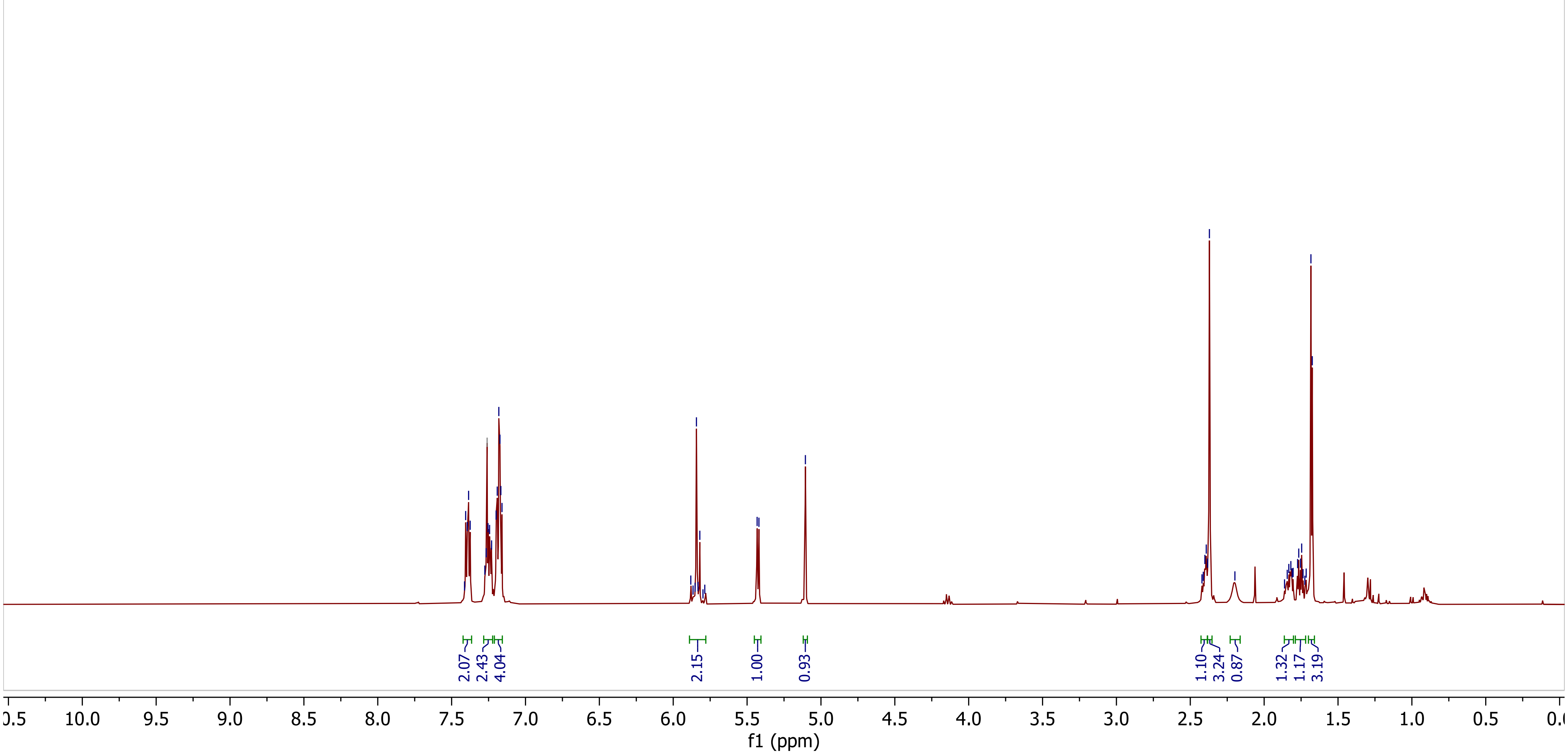

S#472835

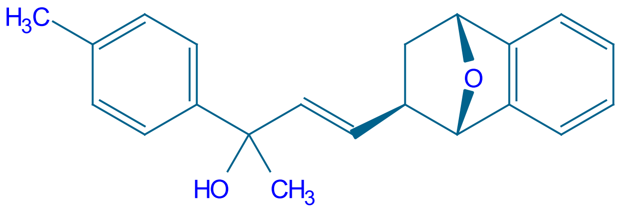

4ad, ^{13}C {^{1}H} (100 MHz), $CDCl_3$

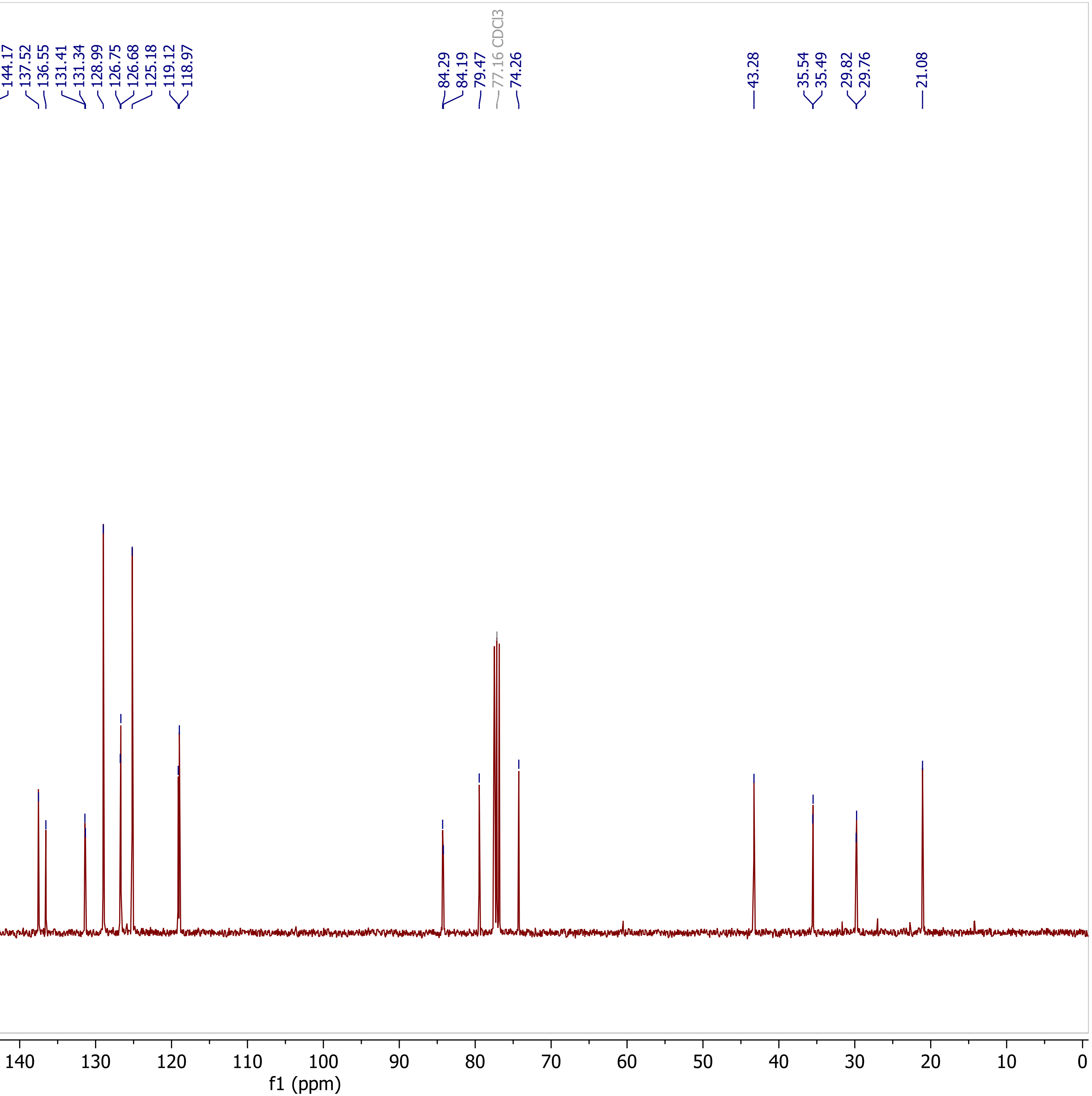

S#435361

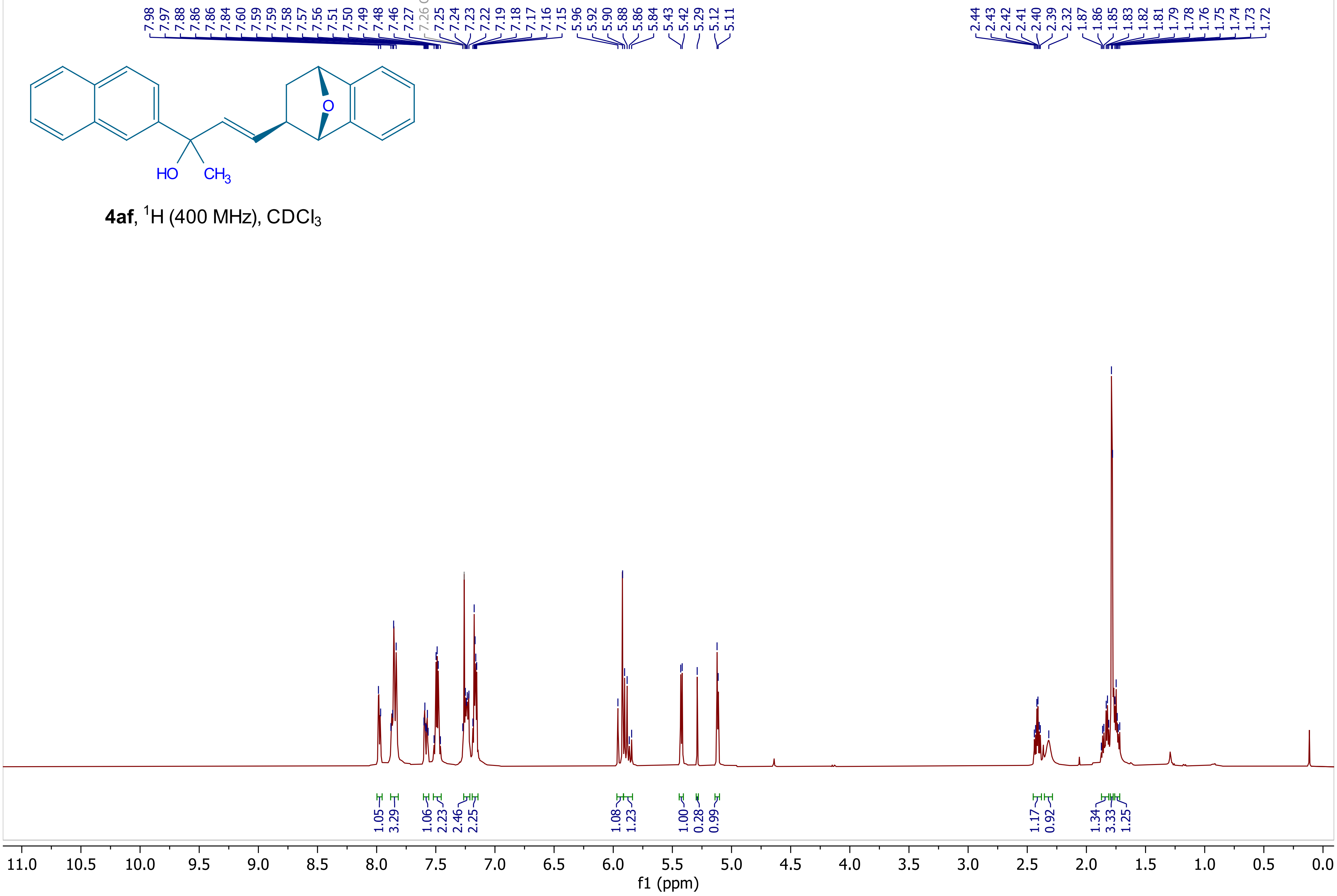

4af, ^{1}H (400 MHz), $CDCl_3$

S#417097

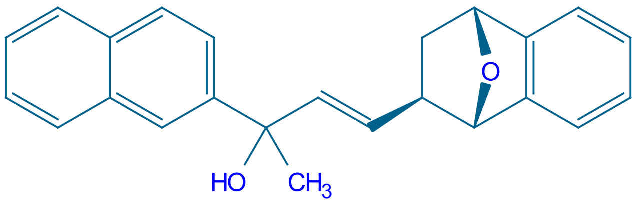

4af, $^{13}C\{^1H\}$ (100 MHz), $CDCl_3$

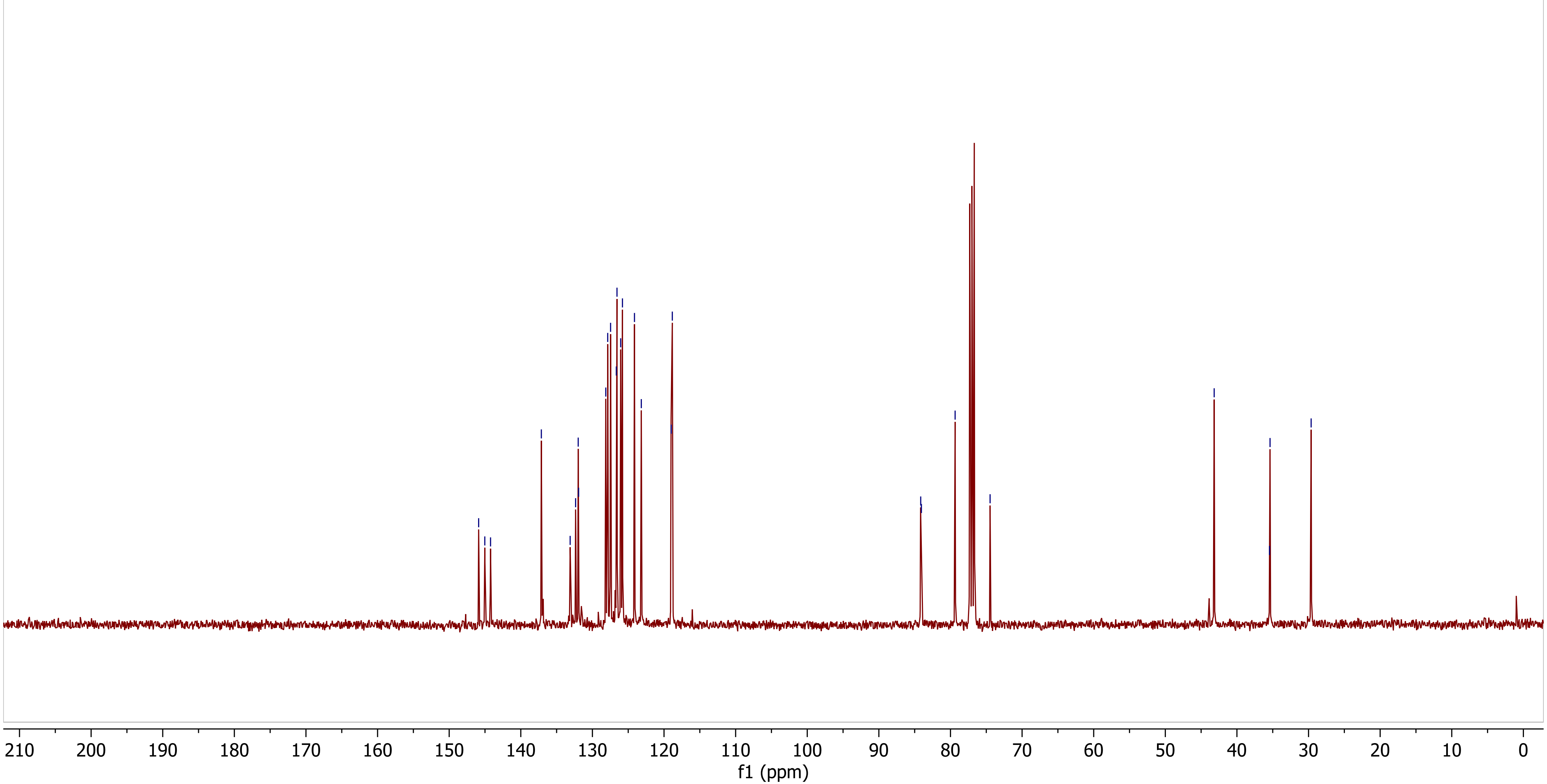

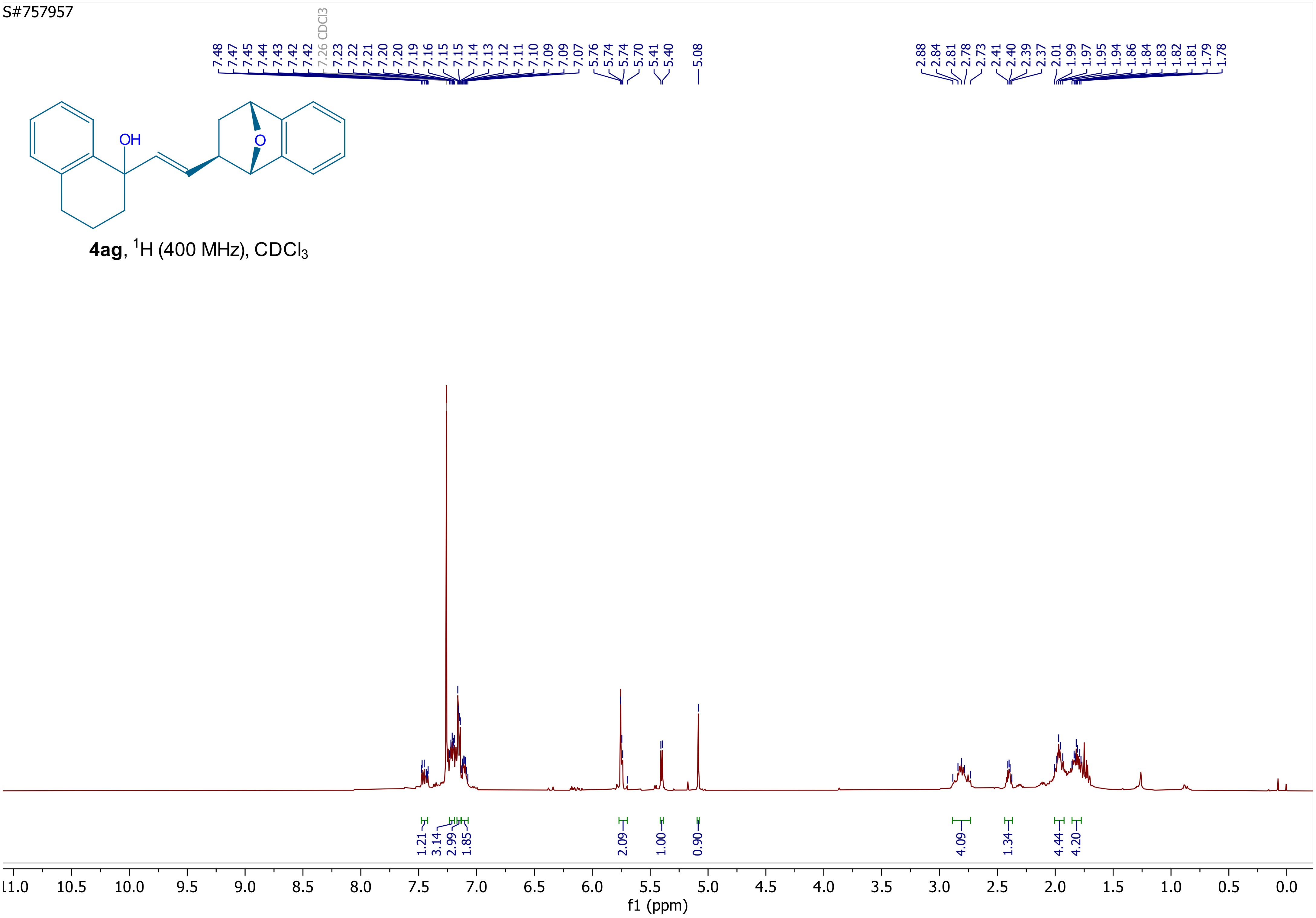

4ag, ^{1}H (400 MHz), $CDCl_3$

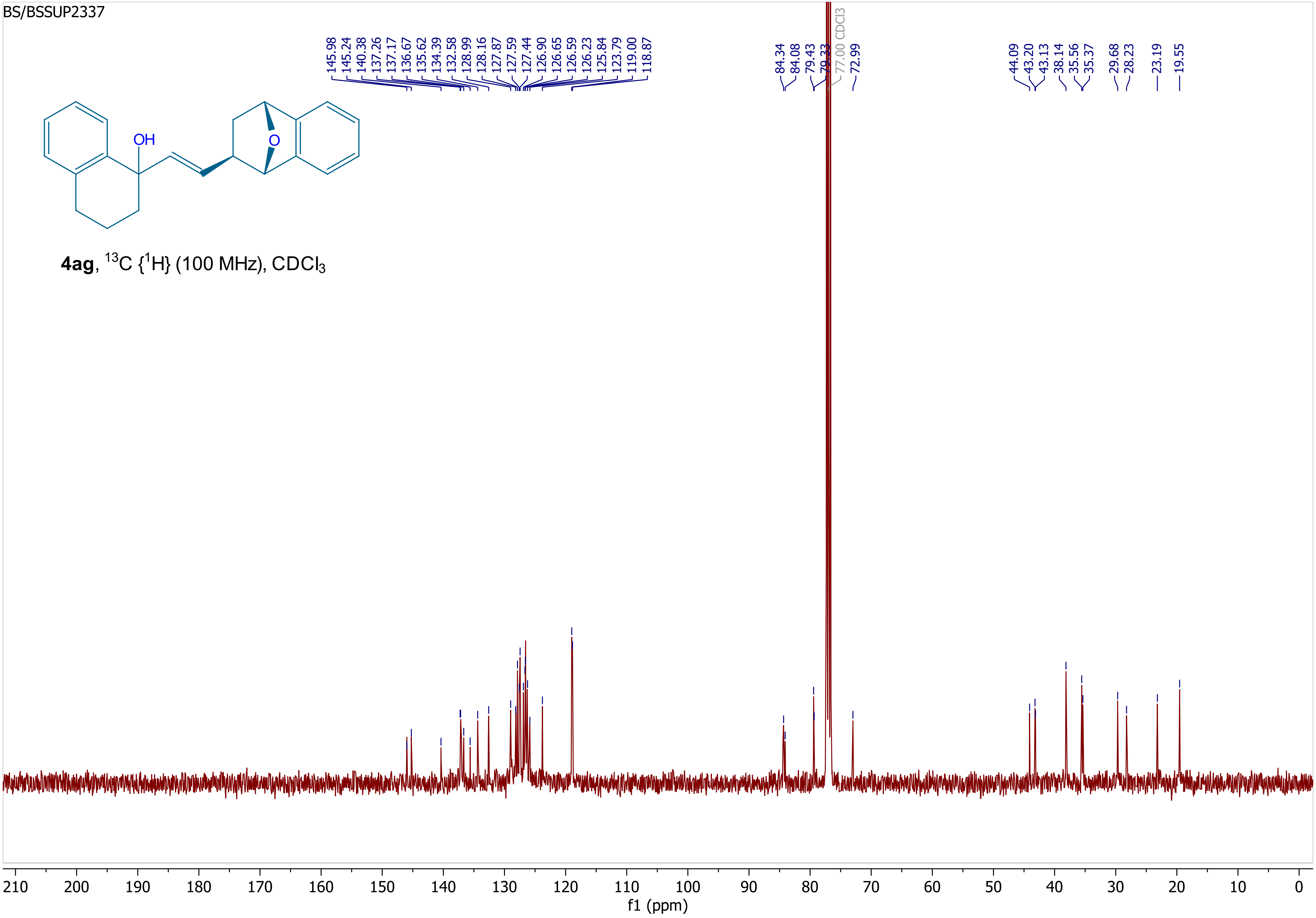
BS/BSSUP2337
145.98
145.24
140.38
137.26
137.17
136.67
135.62
134.39
132.58
128.99
128.16
127.87
127.59
127.44
126.90
126.65
126.59
126.23
125.84
123.79
119.00
118.87
84.34
84.08
79.43
79.33
77.00 CDCl3
72.99
44.09
43.20
43.13
38.14
35.56
35.37
29.68
28.23
23.19
19.55
OH
O
4ag, 13C {1H} (100 MHz), CDCl3
210 200 190 180 170 160 150 140 130 120 110 100 90 80 70 60 50 40 30 20 10 0
f1 (ppm)

S#482632

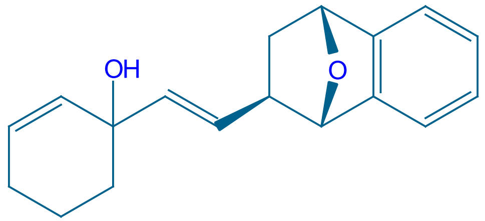

4ah, ^{1}H (400 MHz), $CDCl_3$

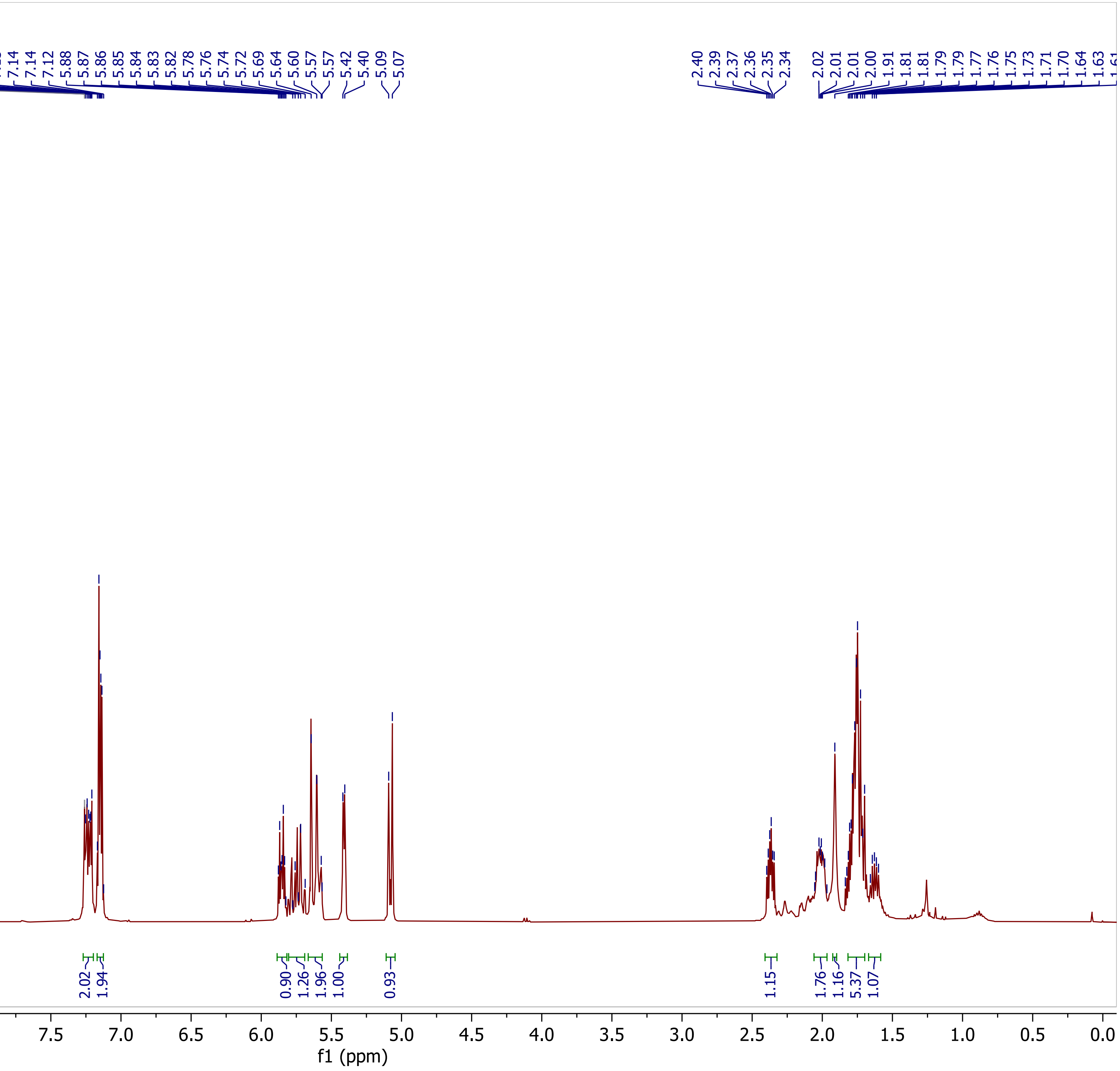

S#484398

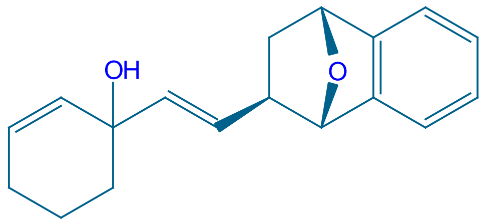

4ah, $^{13}C\ \{^{1}H\}$ (100 MHz), $CDCl_3$

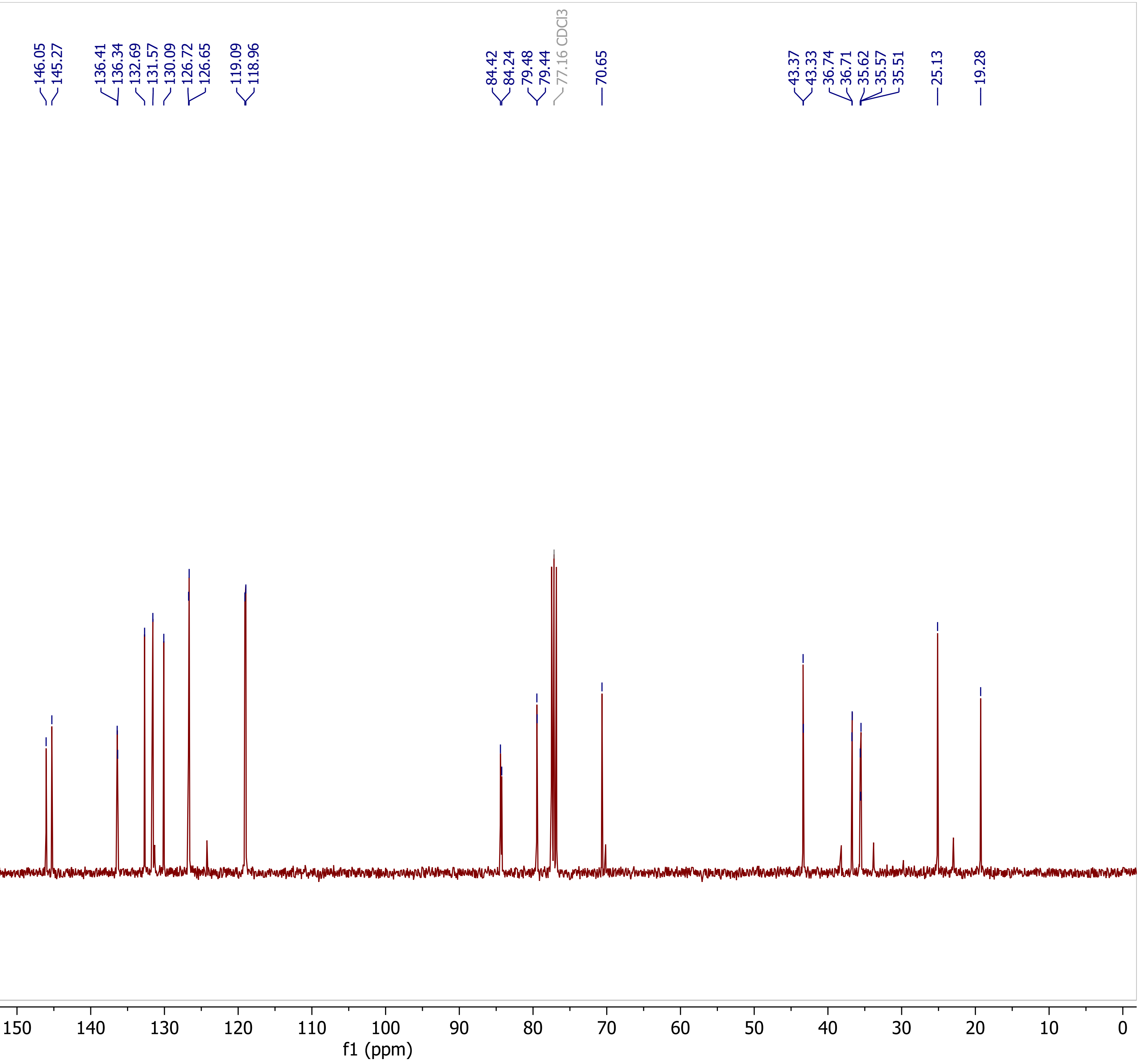

S#376307

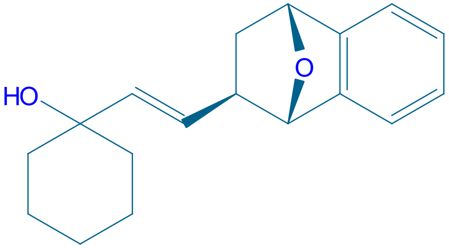

4ai, ^{1}H (400 MHz), $CDCl_3$

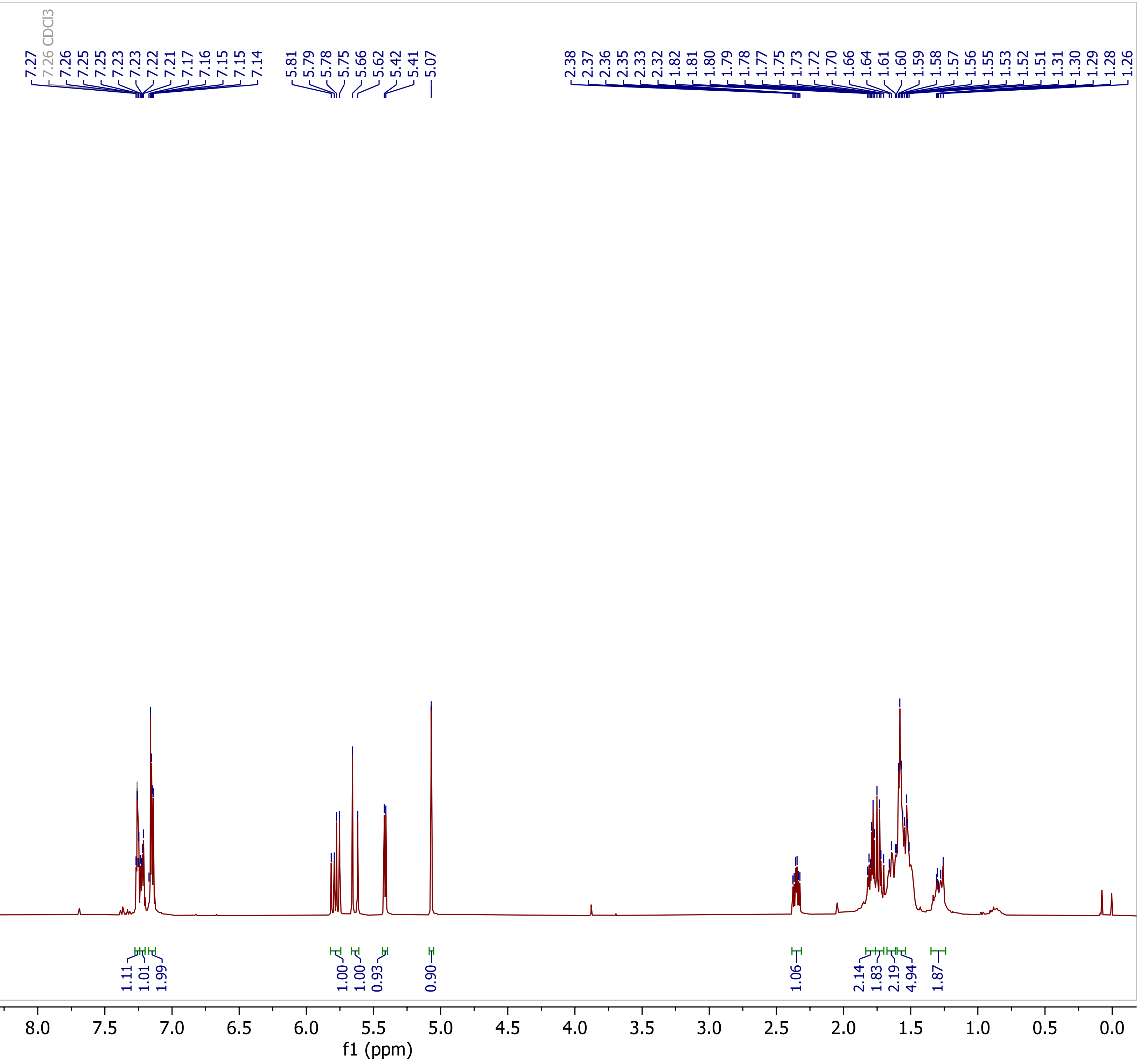

S#505295

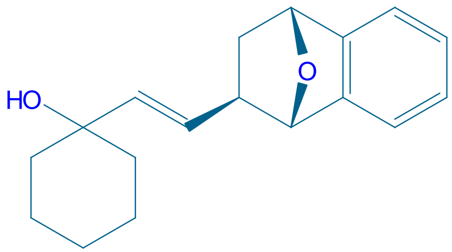

4ai, ^{13}C {^{1}H} (100 MHz), $CDCl_3$

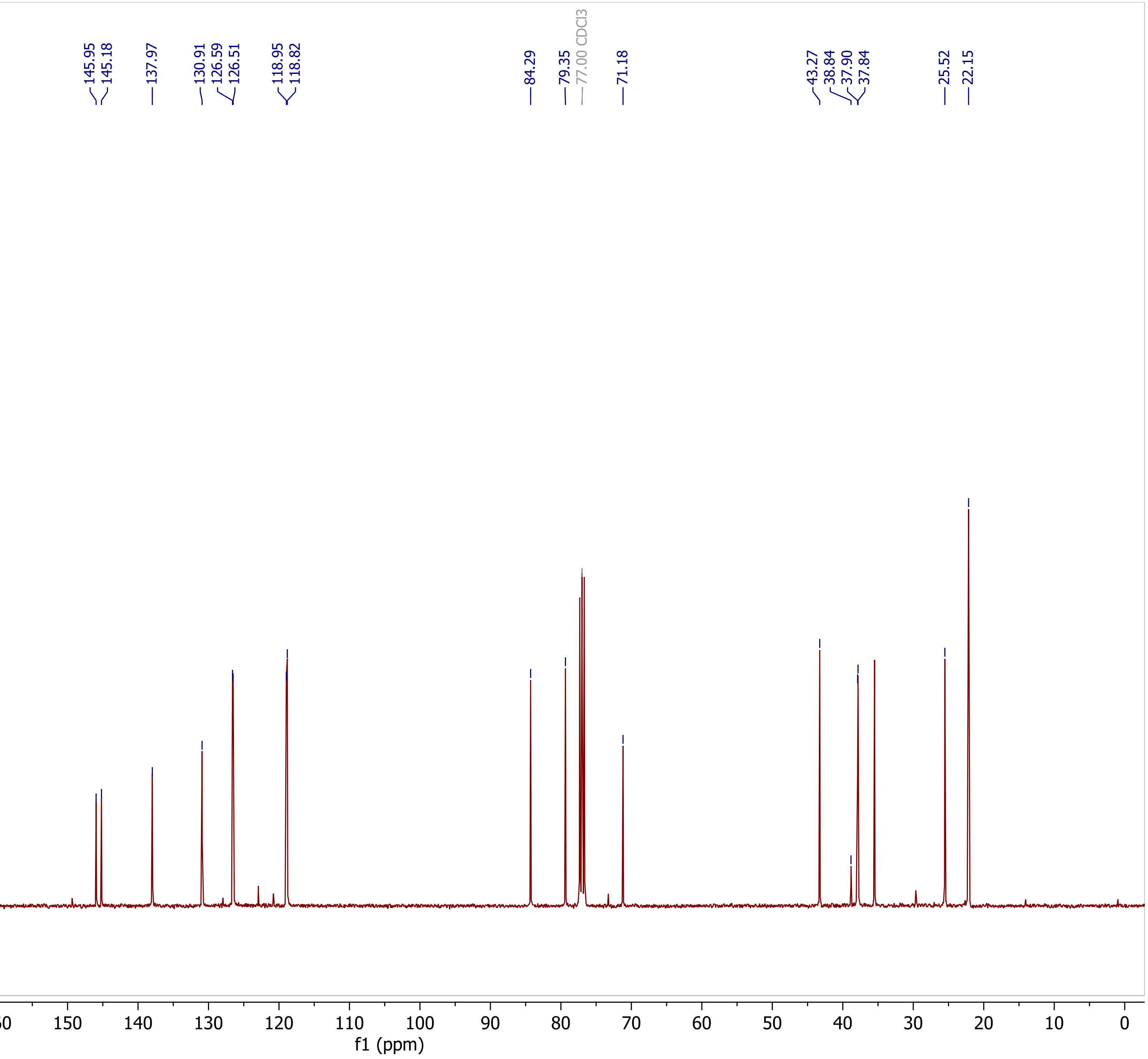

BSSUP1860

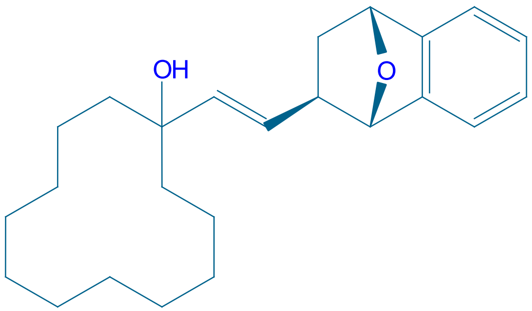

4aj, ^{1}H (400 MHz), $CDCl_3$

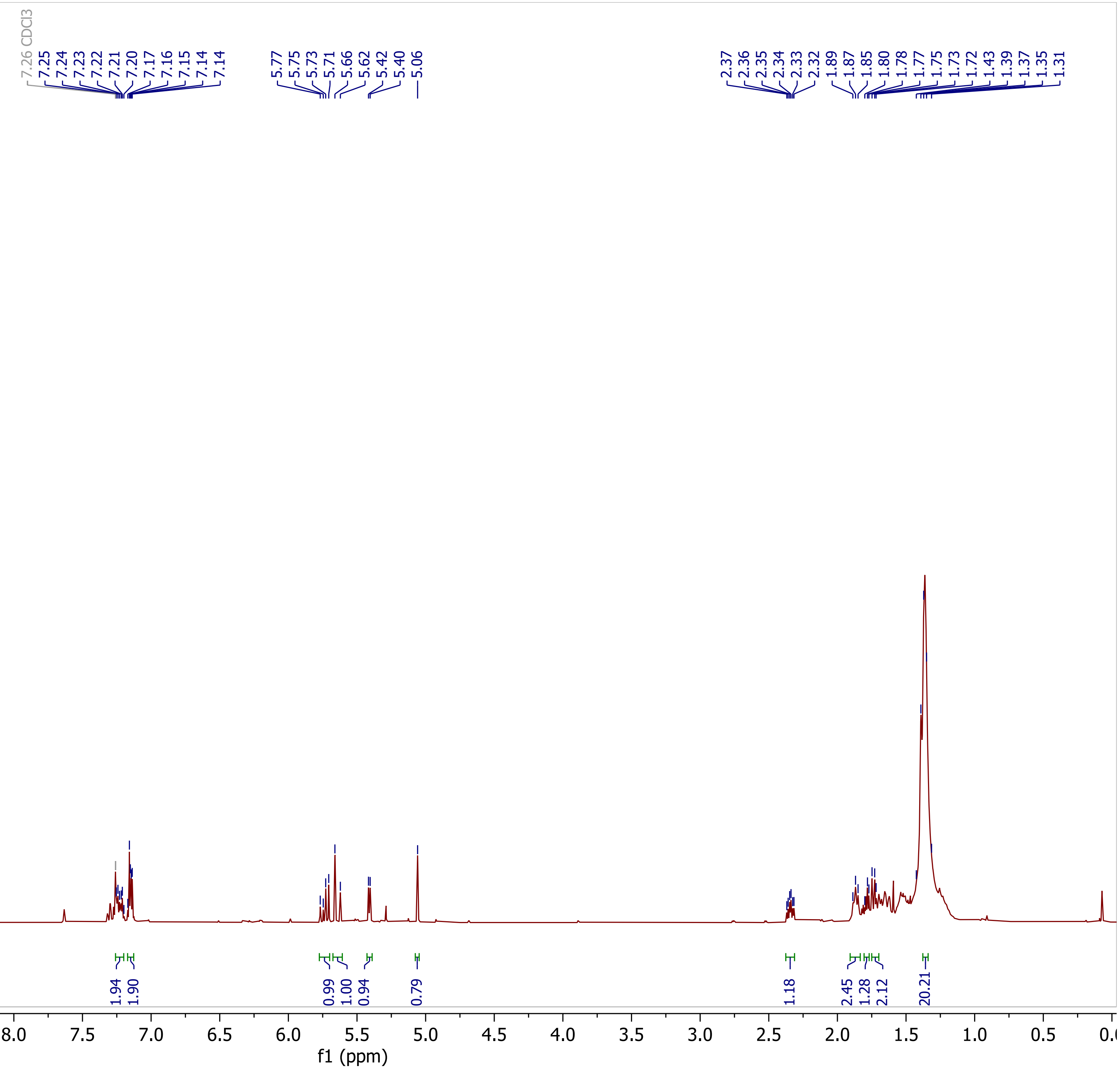

BSSUP1860

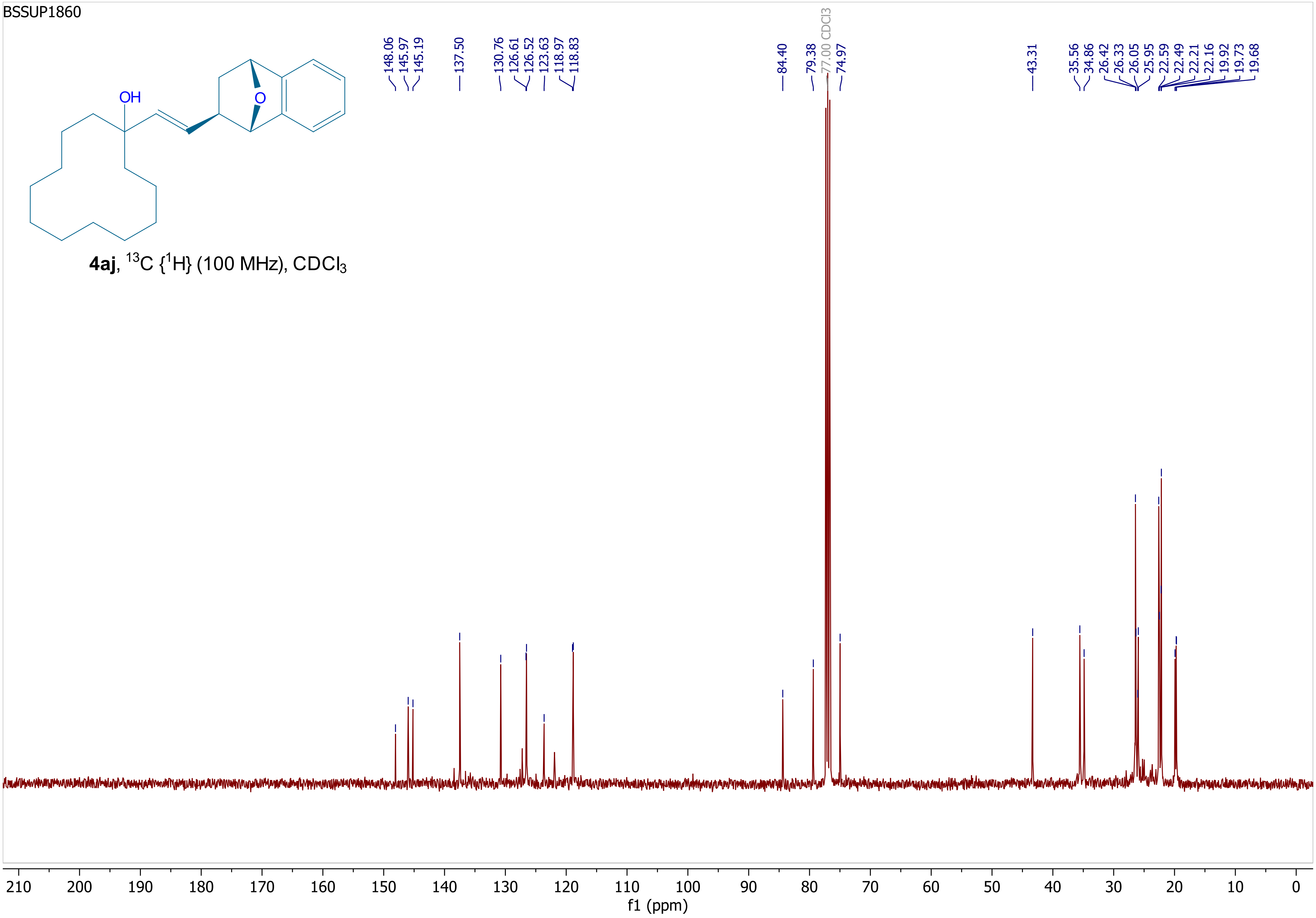

4aj, ^{13}C {^{1}H} (100 MHz), $CDCl_3$

S#379008

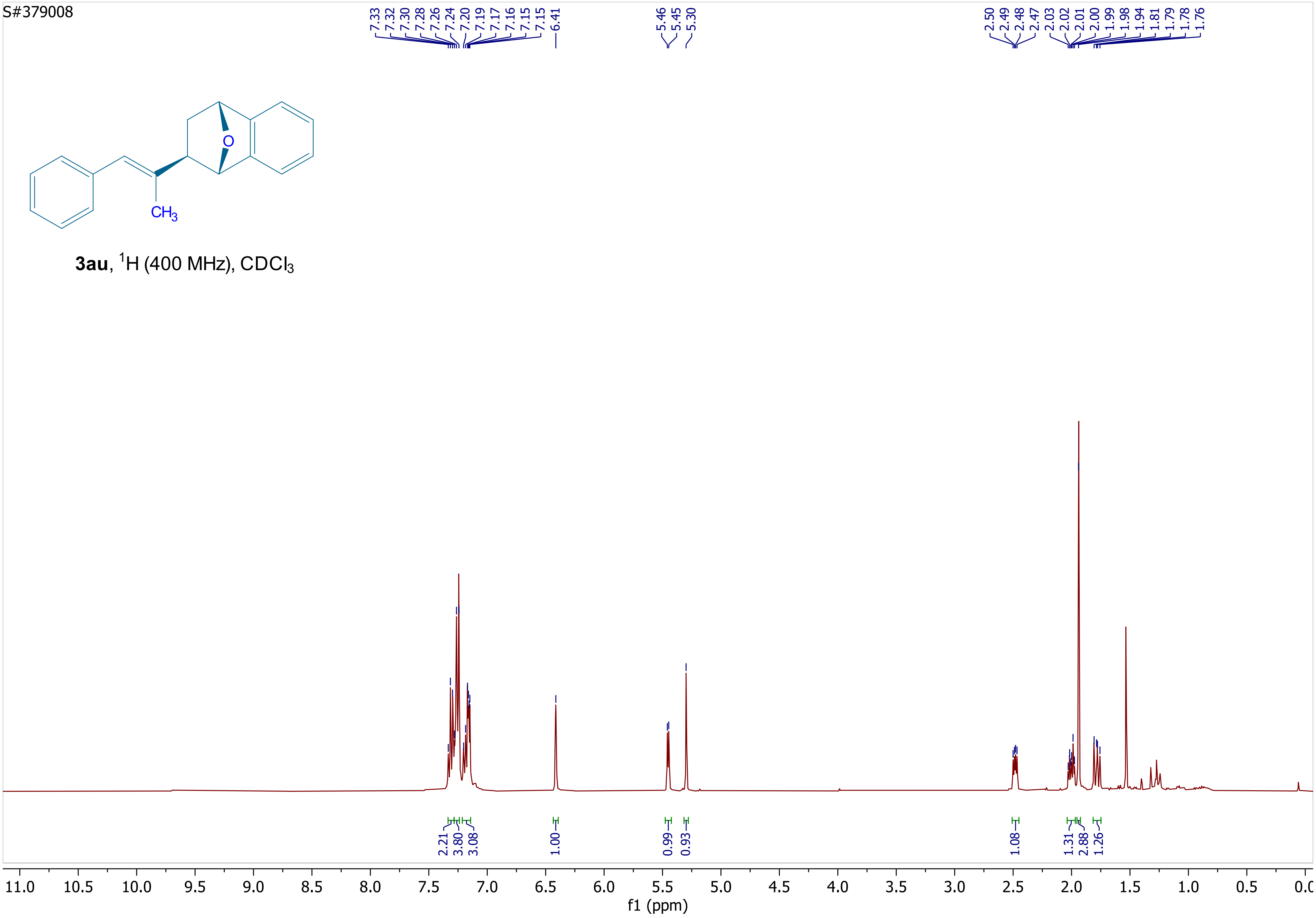

3au, ^{1}H (400 MHz), $CDCl_3$

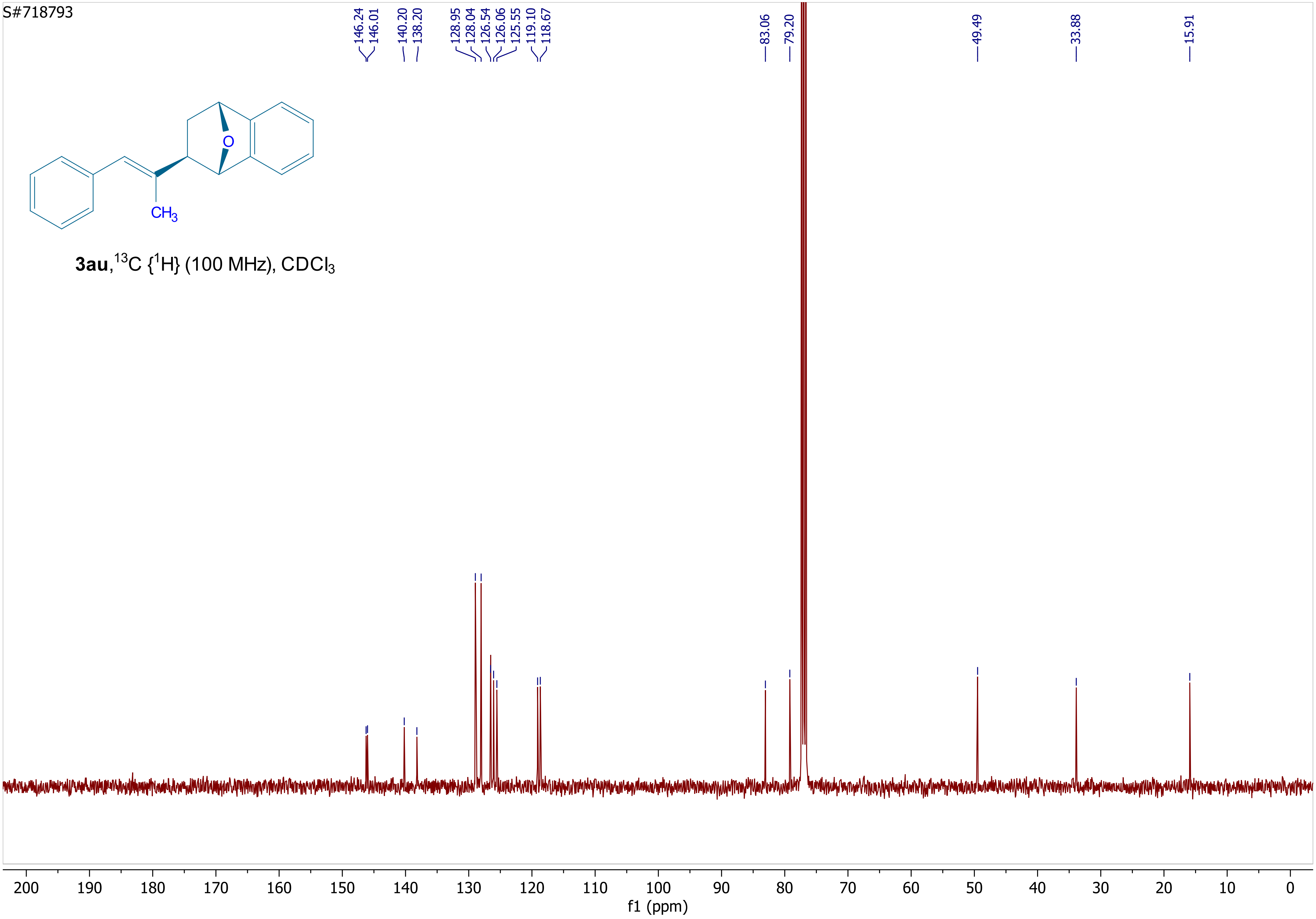
S#718793
146.24
146.01
140.20
138.20
128.95
128.04
126.54
126.06
125.55
119.10
118.67
83.06
79.20
49.49
33.88
15.91
O
CH3
3au, 13C {1H} (100 MHz), CDCl3
200 190 180 170 160 150 140 130 120 110 100 90 80 70 60 50 40 30 20 10 0
f1 (ppm)

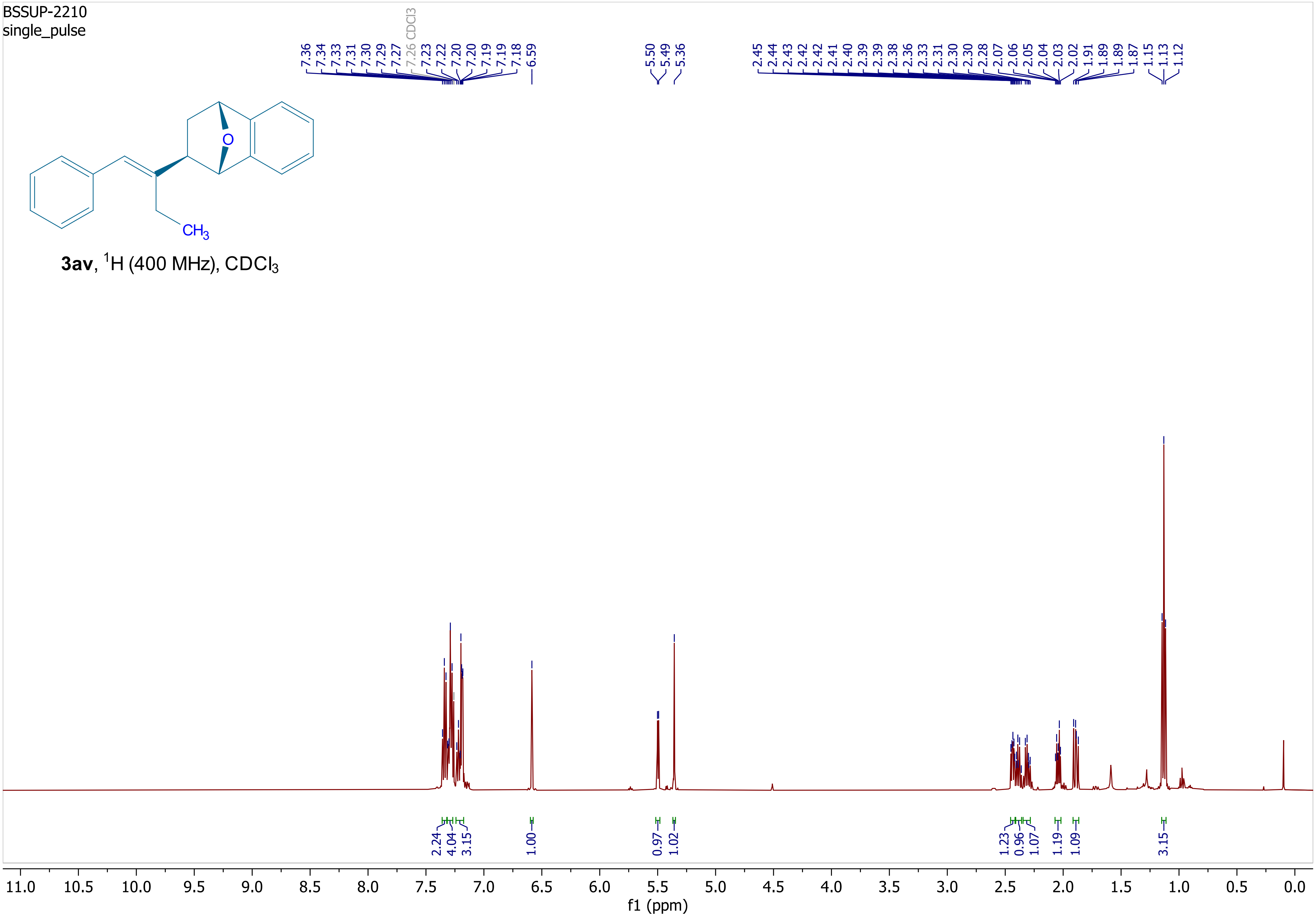

3av, ^{1}H (400 MHz), CDCl$_3$

S#544471

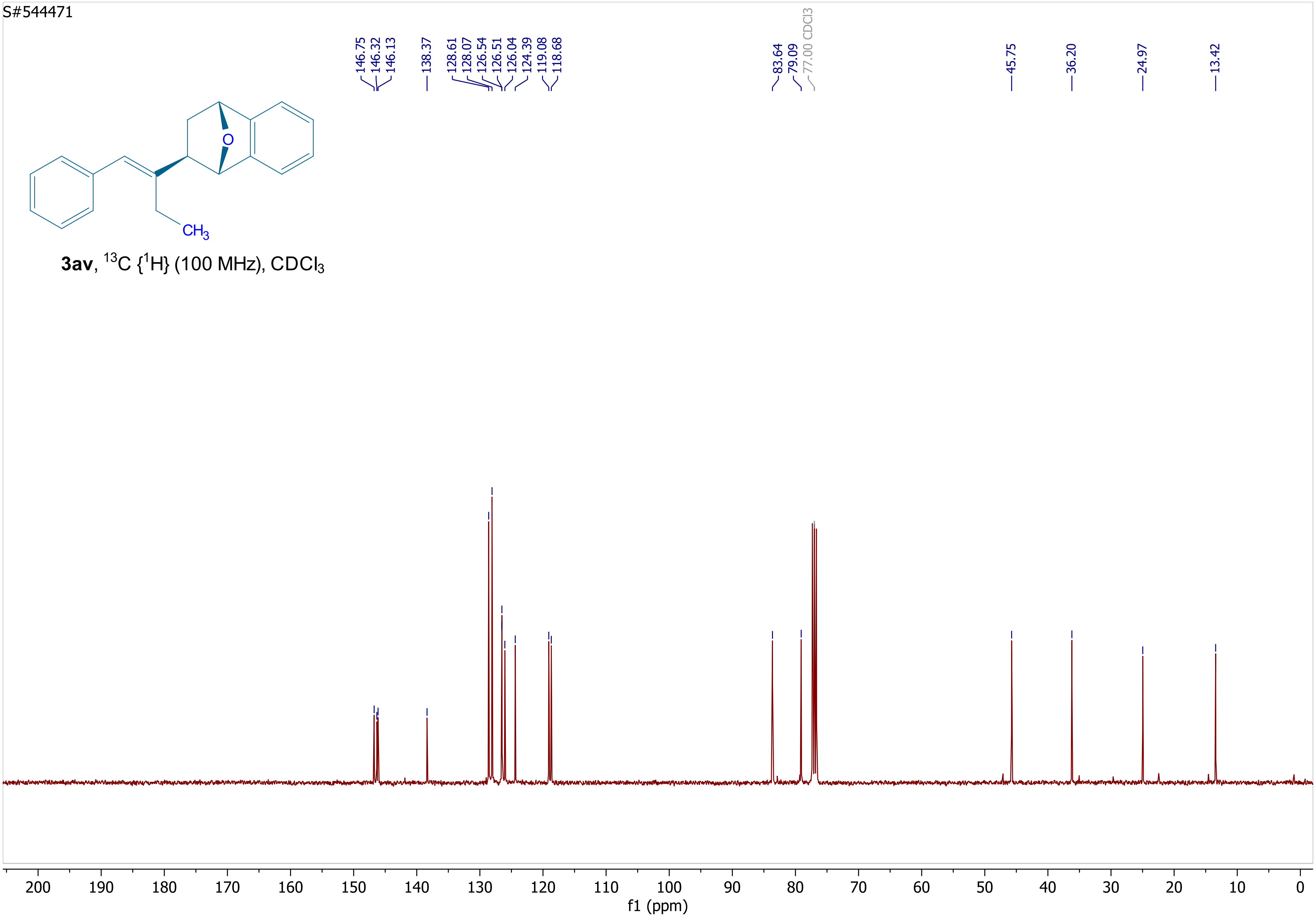

3av, ^{13}C {^{1}H} (100 MHz), $CDCl_3$

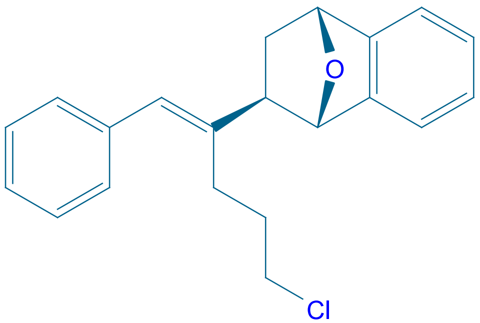

3aw, ^{1}H (400 MHz), $CDCl_3$

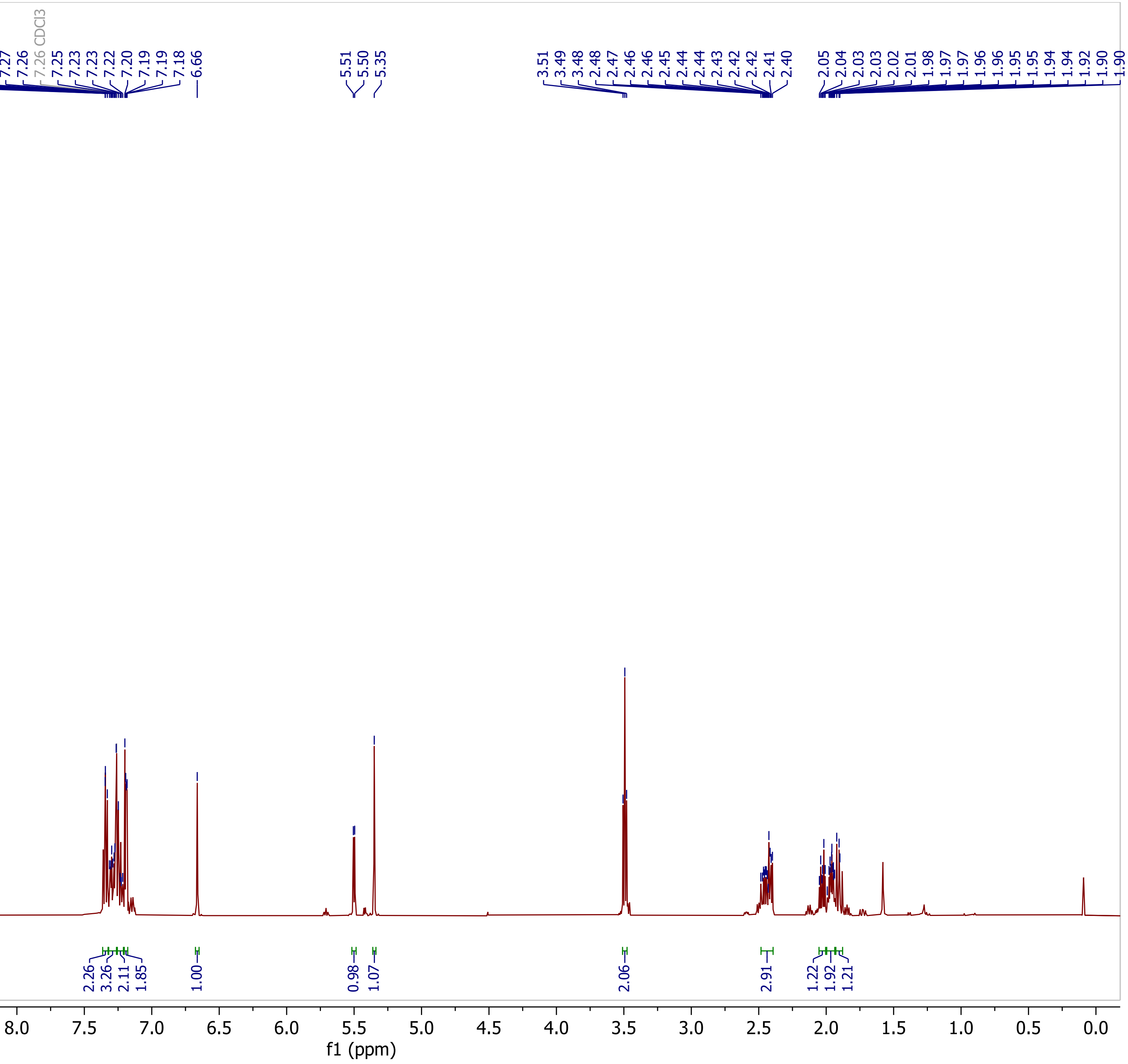

BSSUP2212

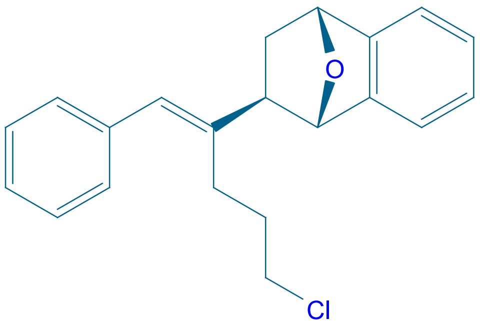

3aw, $^{13}C\{^{1}H\}$ (100 MHz), $CDCl_3$

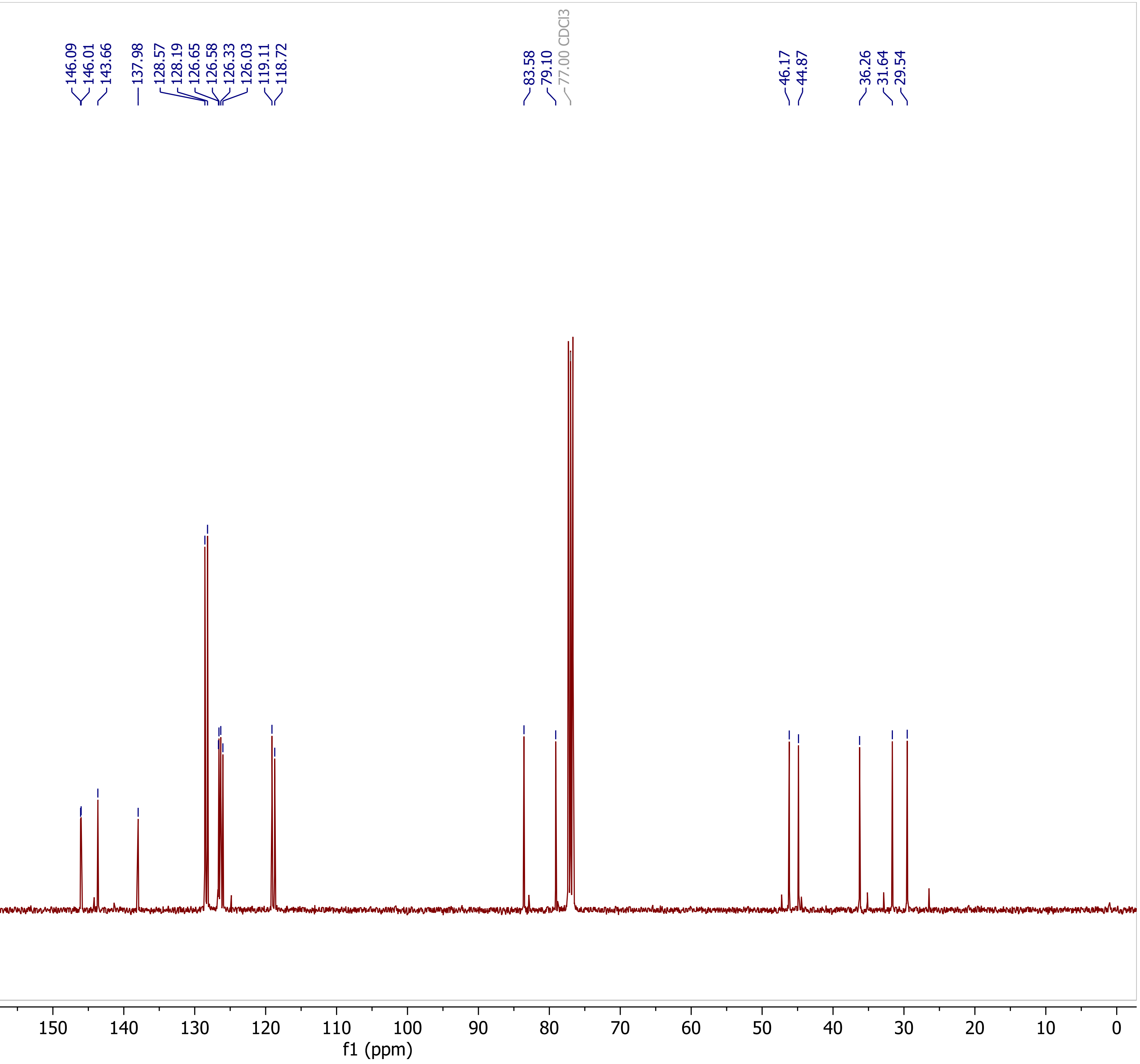

S#410557

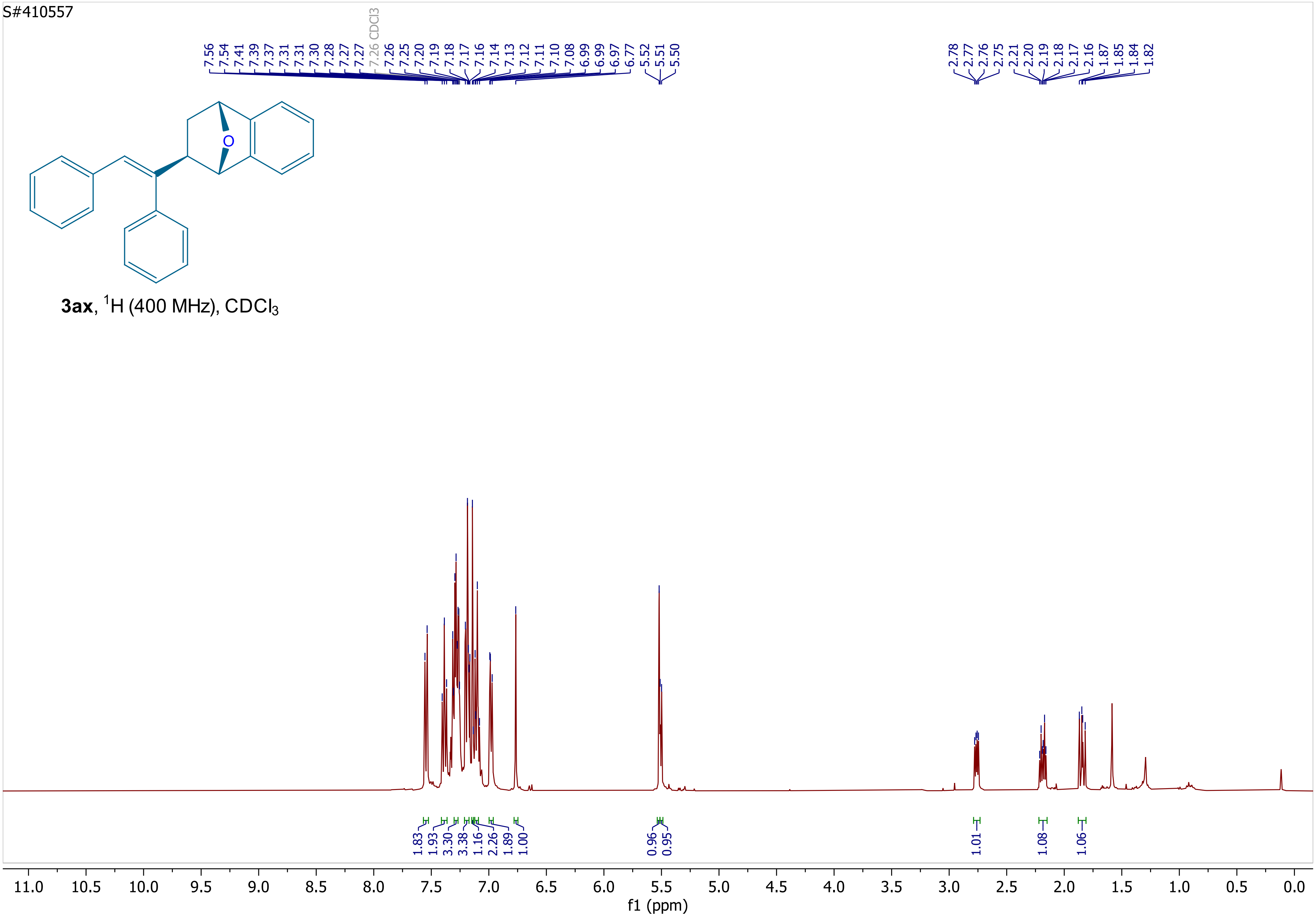

3ax, ^{1}H (400 MHz), $CDCl_3$

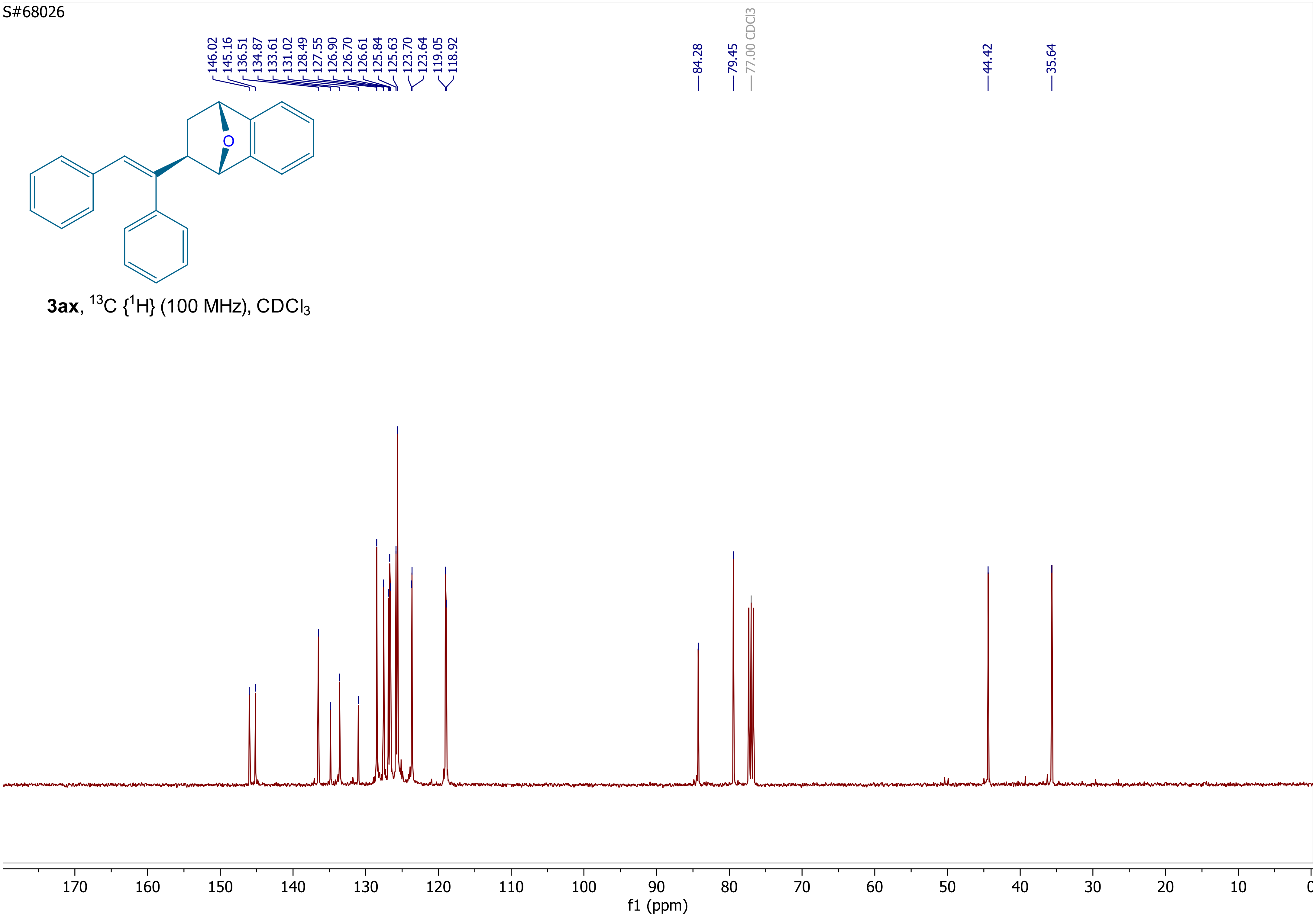

S#68026
146.02
145.16
136.51
134.87
133.61
131.02
128.49
127.55
126.90
126.70
126.61
125.84
125.63
123.70
123.64
119.05
118.92
84.28
79.45
77.00 CDCl3
44.42
35.64
O
3ax, $^{13}C\ \{^1H\}$ (100 MHz), $CDCl_3$
170
160
150
140
130
120
110
100
90
80
70
60
50
40
30
20
10
0
f1 (ppm)

S#372500

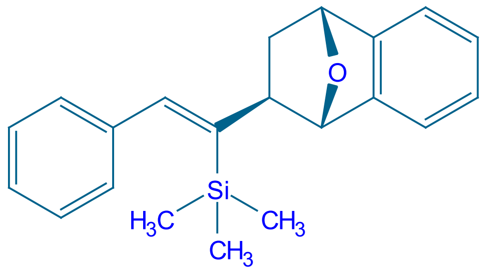

3ay, ^1H (400 MHz), $CDCl_3$

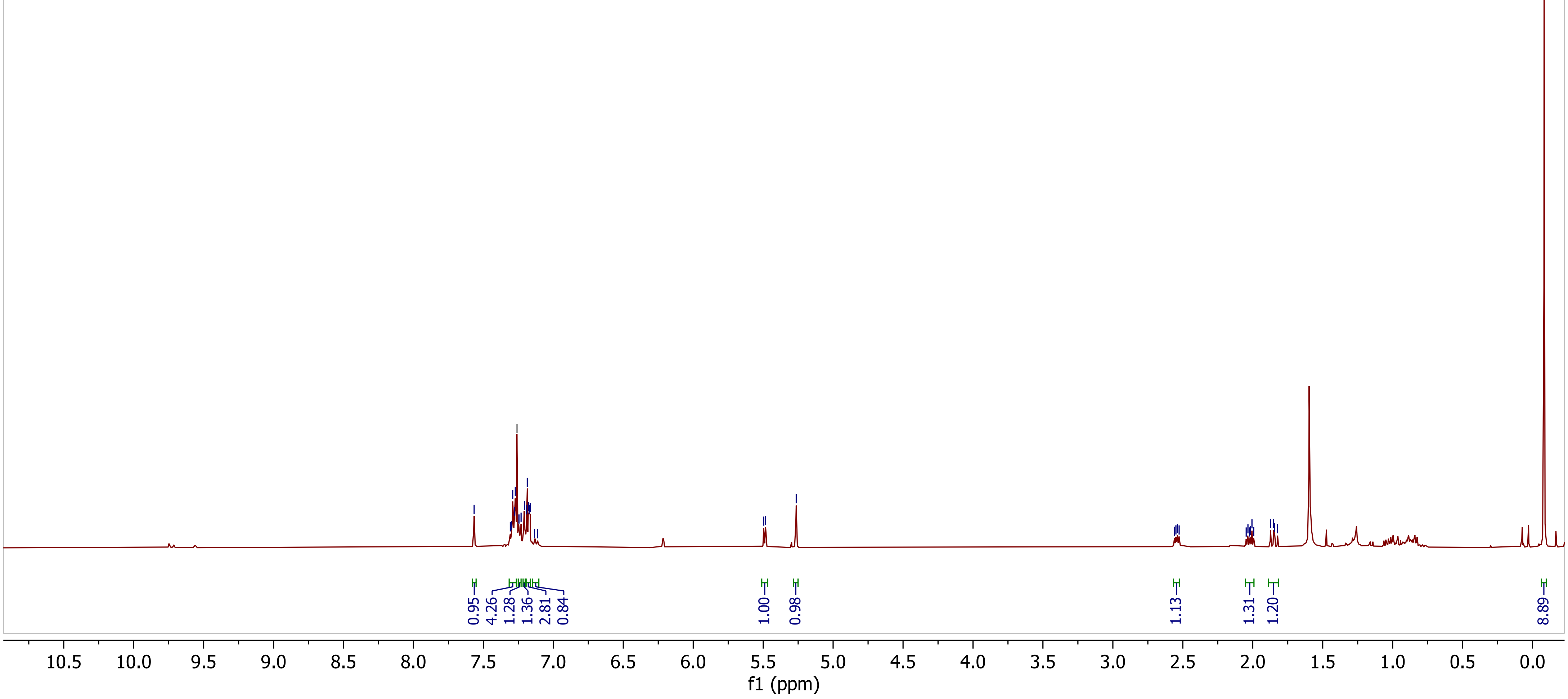

S#522393

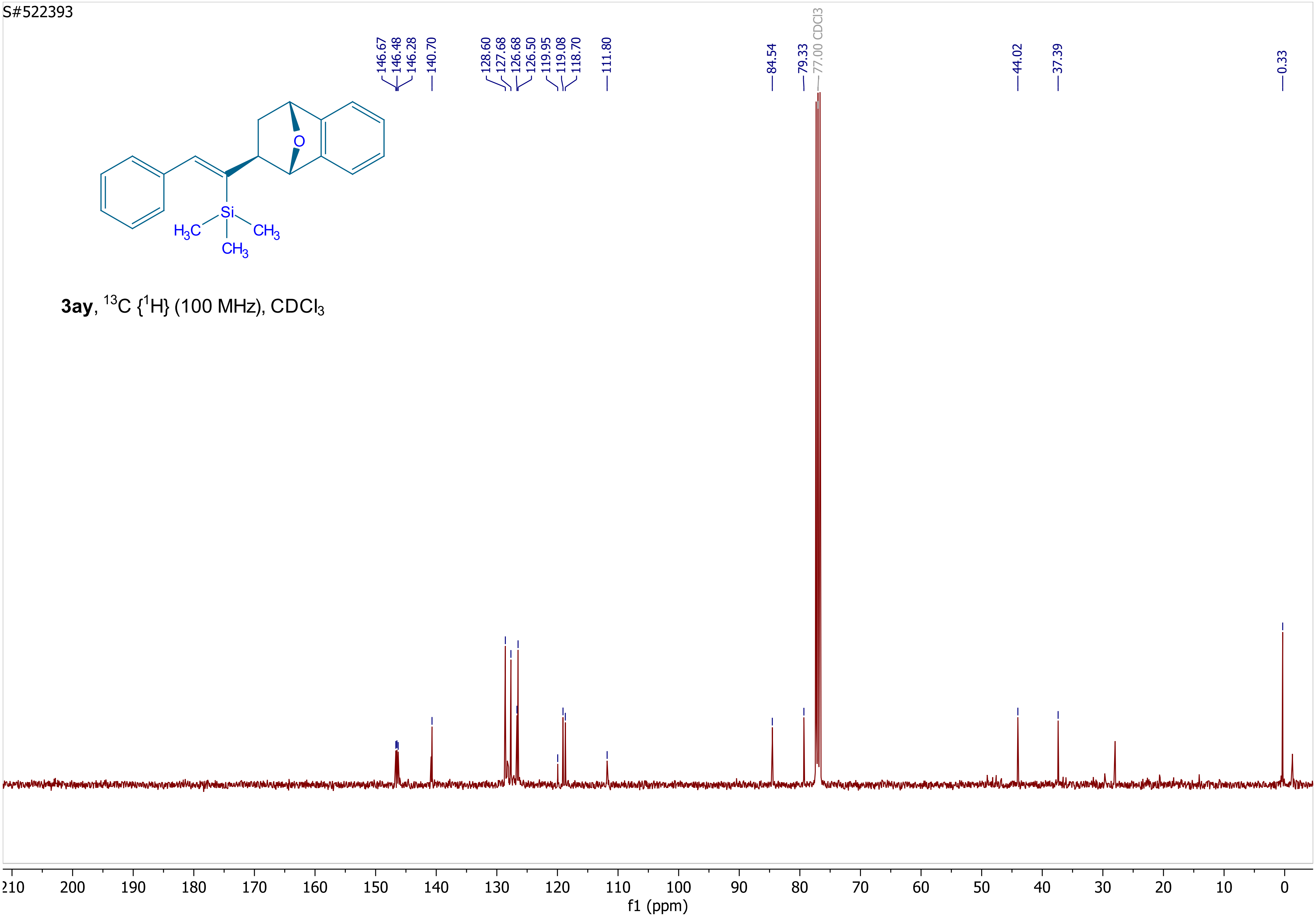

146.67
146.48
146.28
140.70
128.60
127.68
126.68
126.50
119.95
119.08
118.70
111.80
84.54
79.33
77.00 CDCl3
44.02
37.39
0.33
O
Si
H_3C
CH_3
CH_3
3ay, ^{13}C {^{1}H} (100 MHz), $CDCl_3$
210 200 190 180 170 160 150 140 130 120 110 100 90 80 70 60 50 40 30 20 10 0
f1 (ppm)

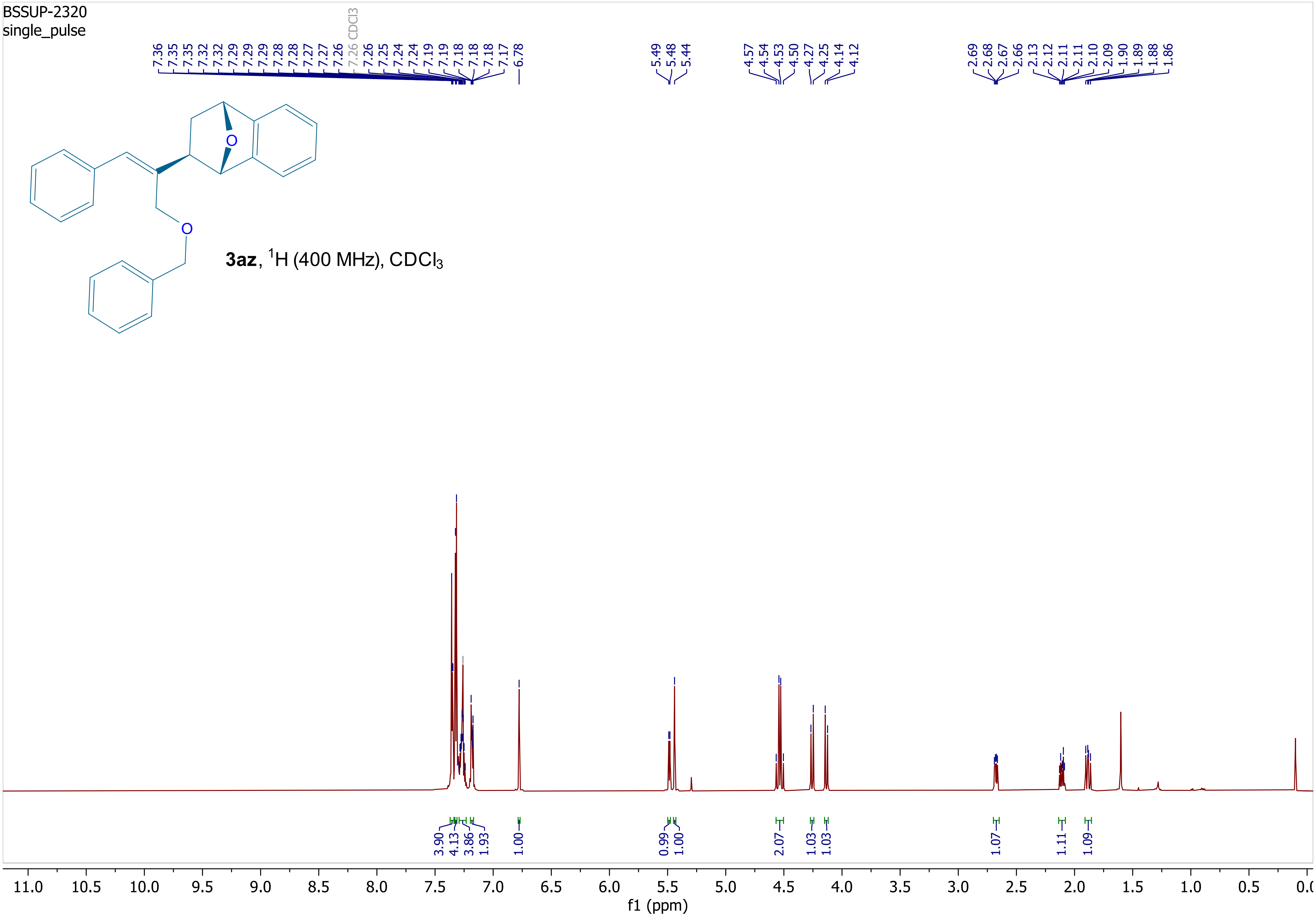

BSSUP-2320
single_pulse
7.36 7.35 7.35 7.32 7.32 7.29 7.29 7.29 7.28 7.28 7.27 7.27 7.26 7.26 CDCl3 7.26 7.25 7.24 7.24 7.19 7.19 7.18 7.18 7.18 7.17 6.78
5.49 5.48 5.44
4.57 4.54 4.53 4.50 4.27 4.25 4.14 4.12
2.69 2.68 2.67 2.66 2.13 2.12 2.11 2.11 2.10 2.09 1.90 1.89 1.88 1.86
O
O
3az, 1H (400 MHz), CDCl3
3.90 4.13 3.86 1.93 1.00 0.99 1.00 2.07 1.03 1.03 1.07 1.11 1.09
11.0 10.5 10.0 9.5 9.0 8.5 8.0 7.5 7.0 6.5 6.0 5.5 5.0 4.5 4.0 3.5 3.0 2.5 2.0 1.5 1.0 0.5
f1 (ppm)

S#860937

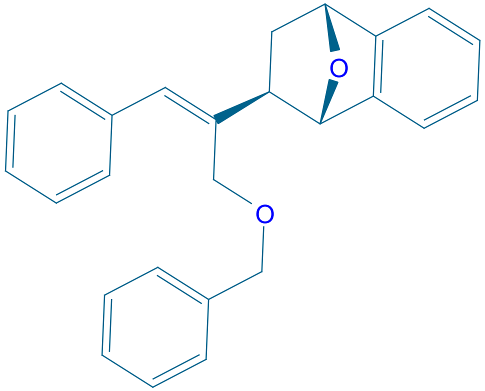

3az, ^{13}C {^{1}H} (100 MHz), $CDCl_3$

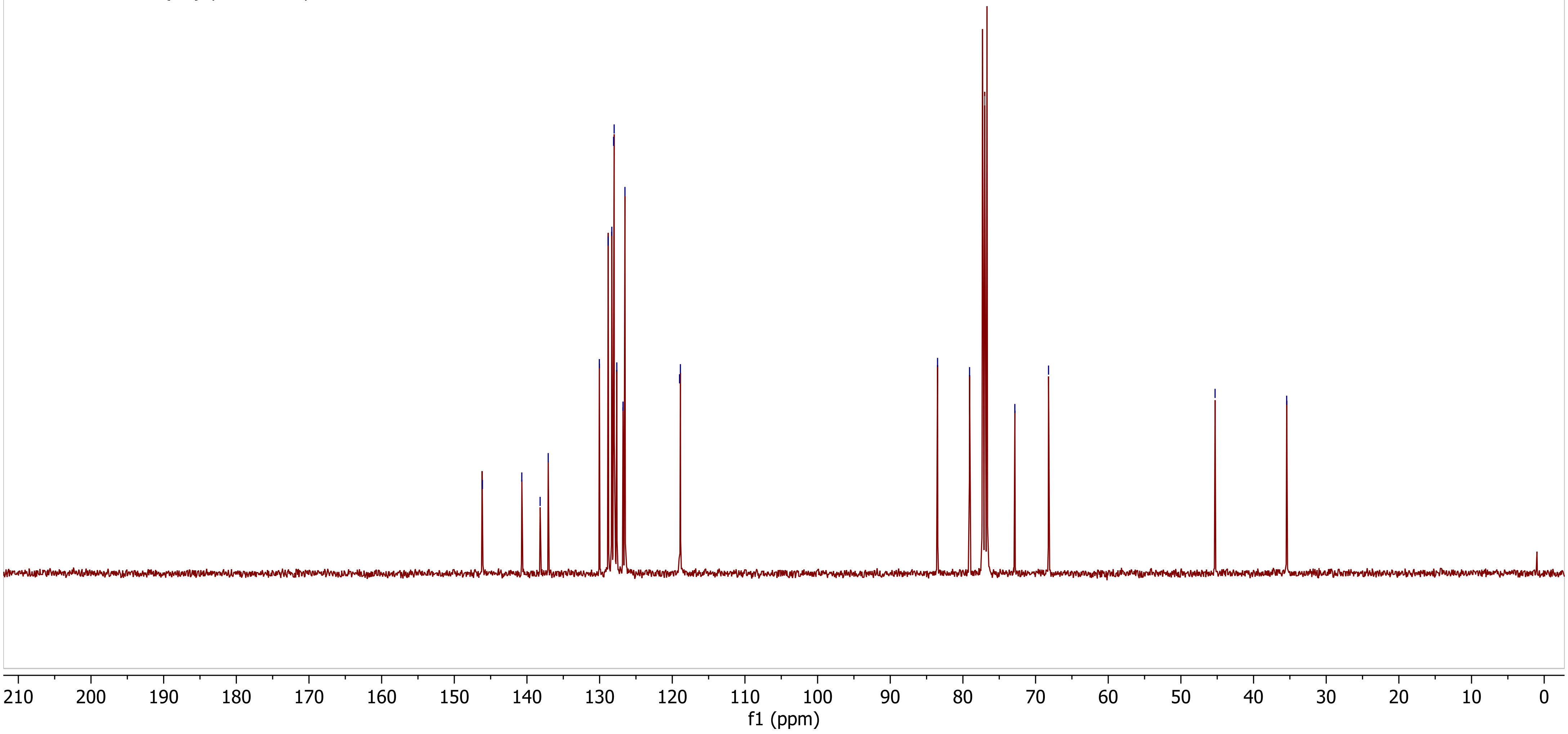

S#376857

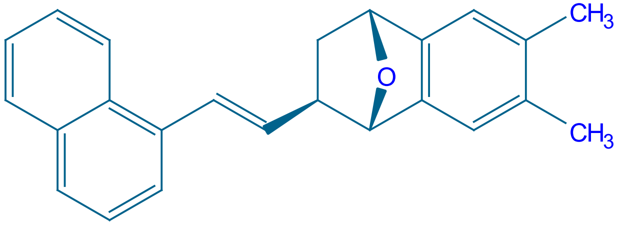

3bi, ^{1}H (400 MHz), $CDCl_3$

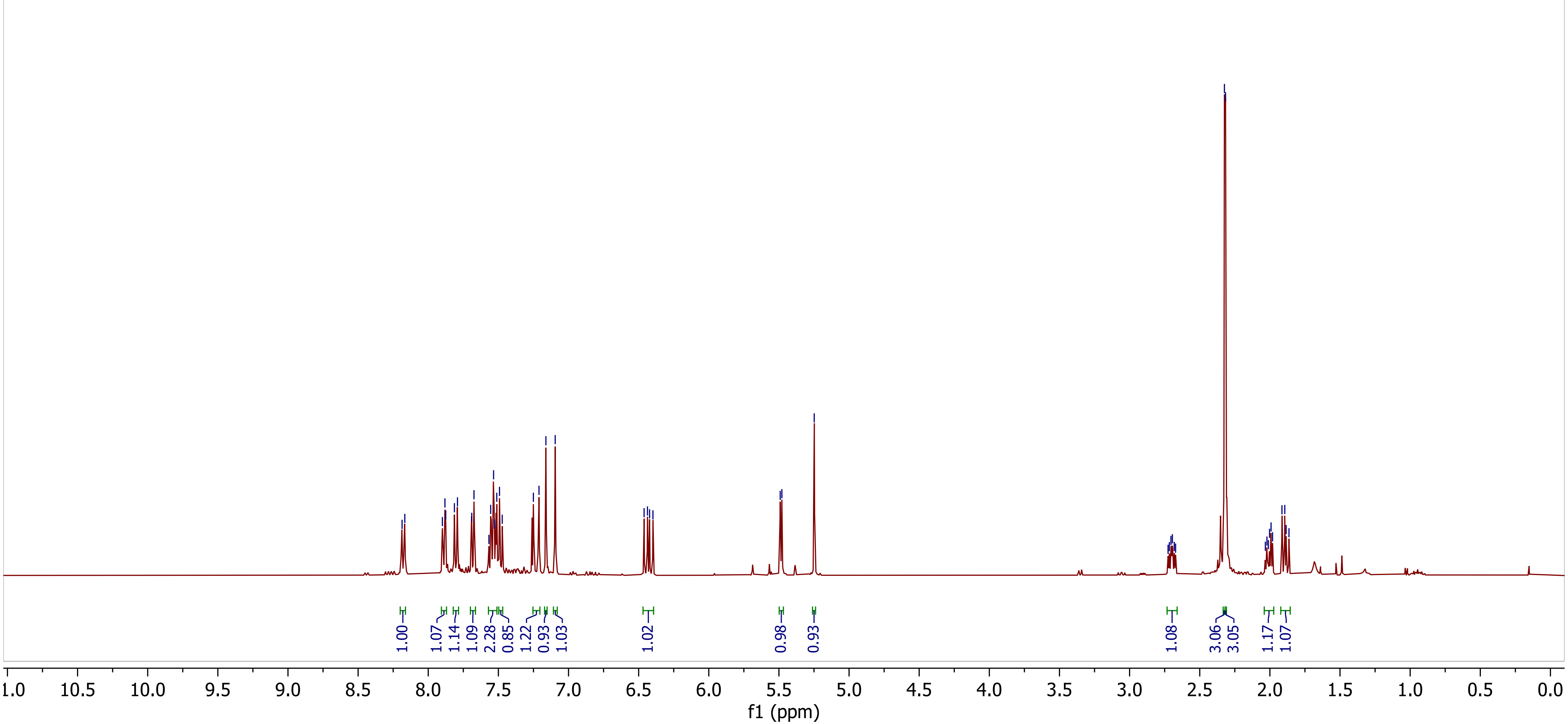

S#749645

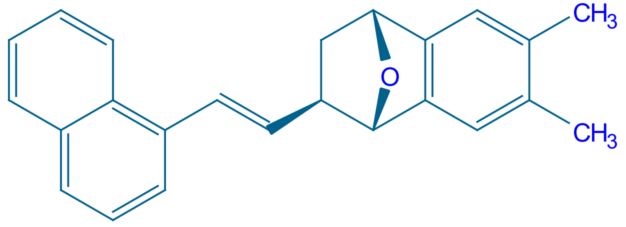

3bi, $^{13}C\{^1H\}$ (100 MHz), $CDCl_3$

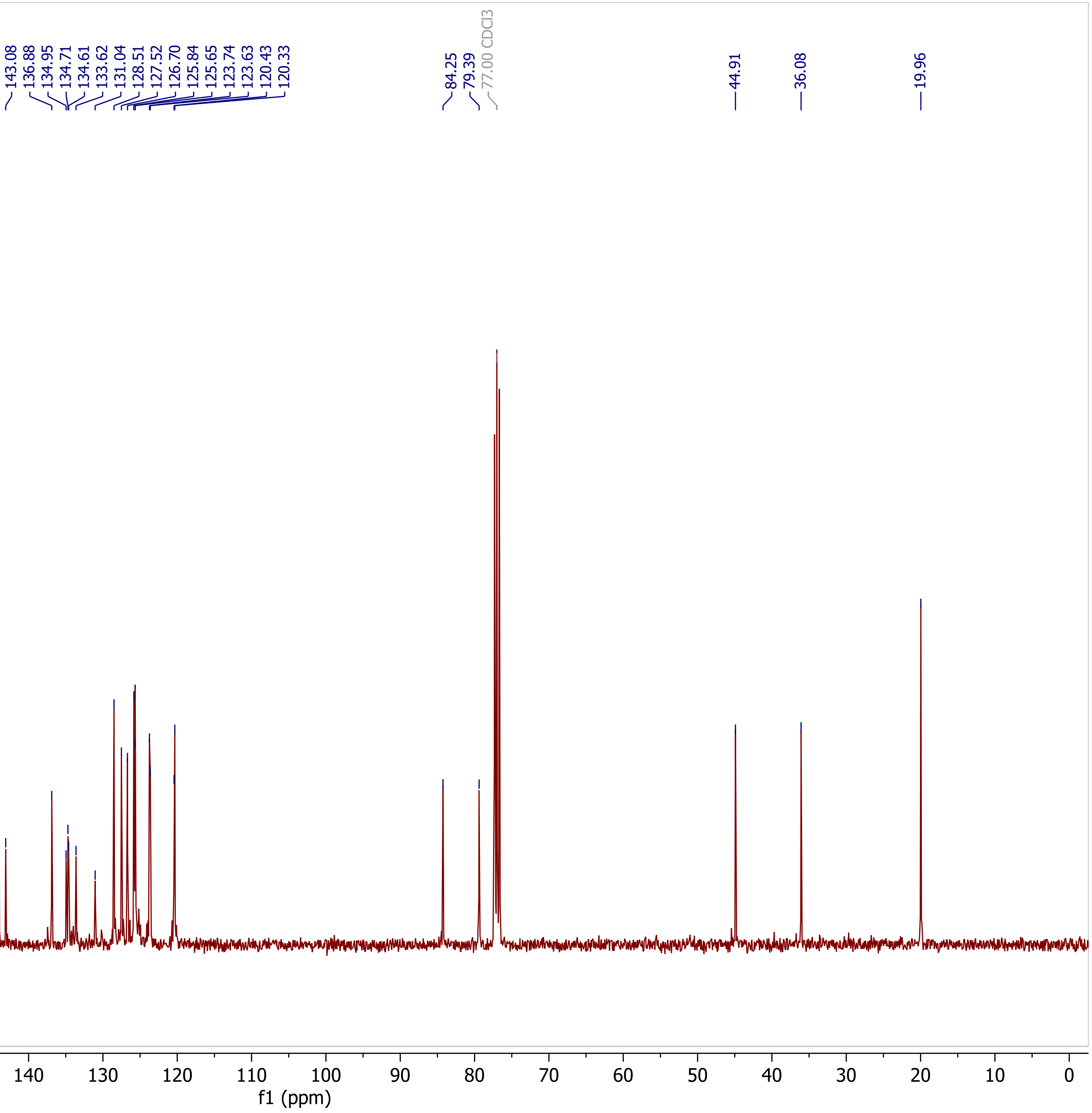

S#413224

3ci,¹H (400 MHz), $CDCl_3$

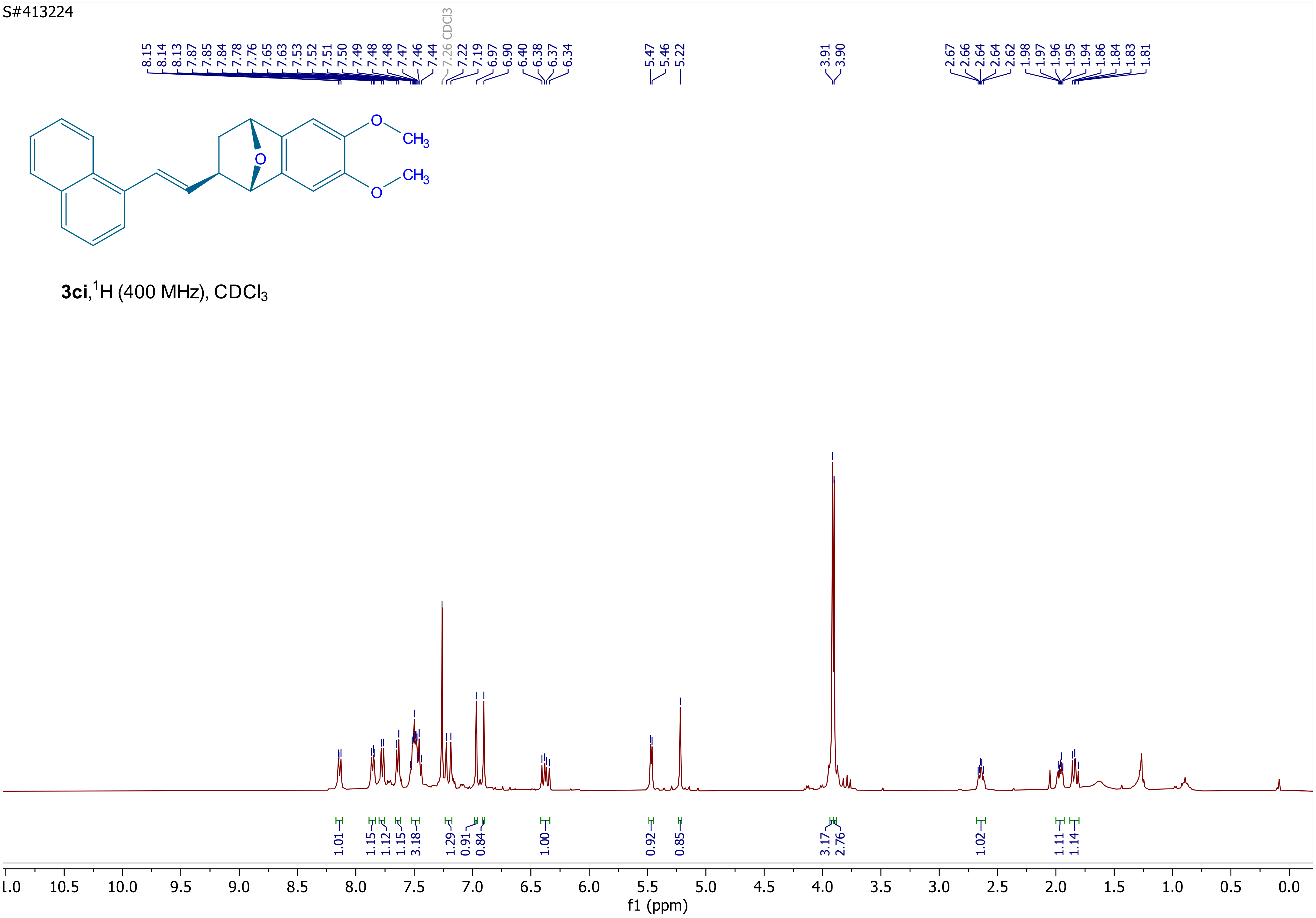

S#747886

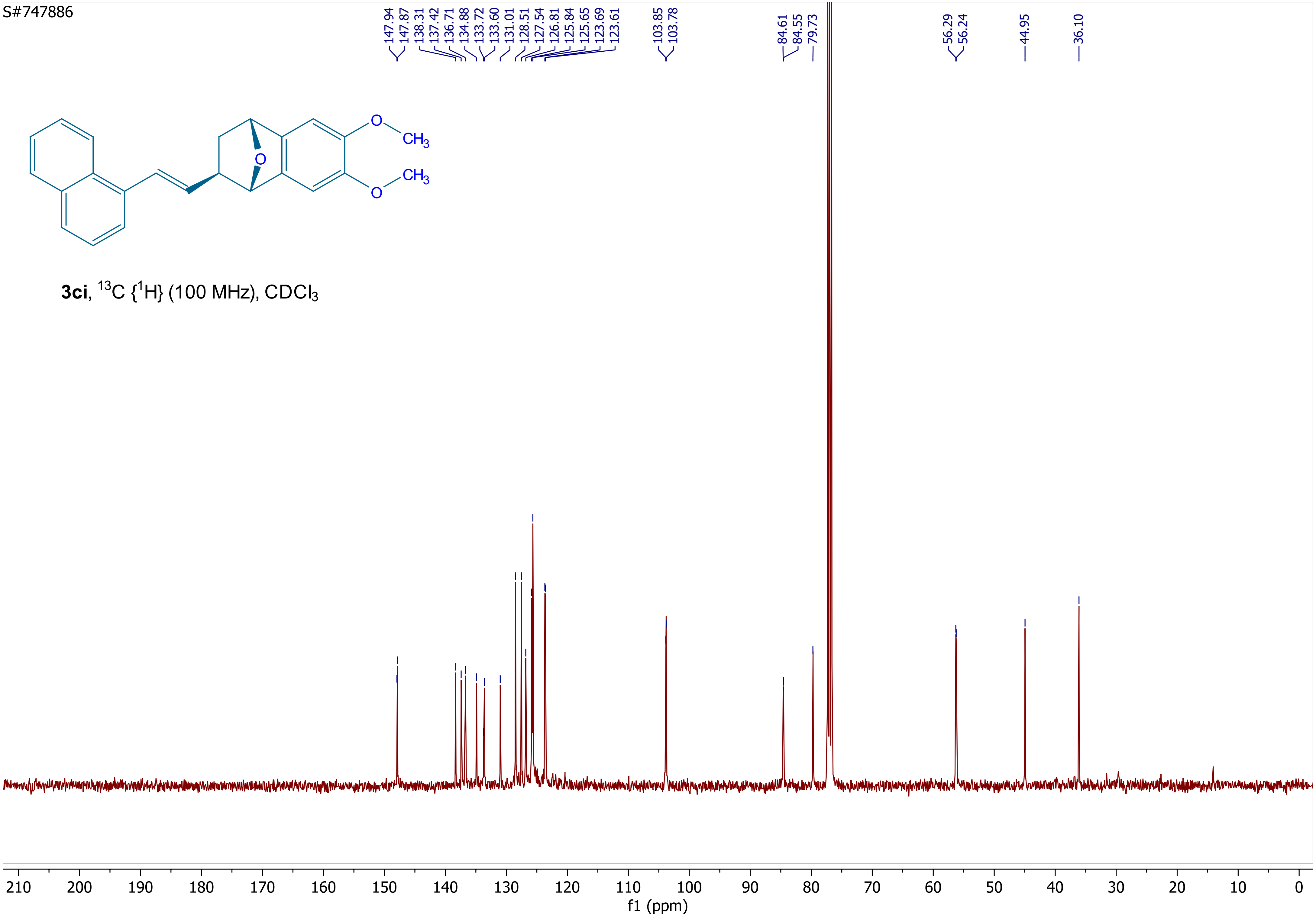

3ci, $^{13}C\ \{^1H\}$ (100 MHz), $CDCl_3$

BSSUP2468
single_pulse

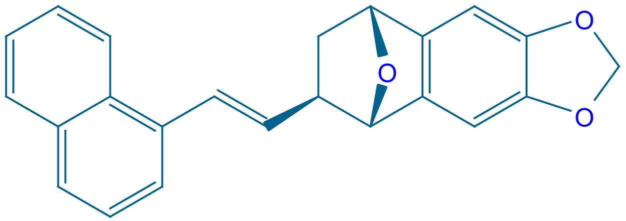

3di, ^{1}H (400 MHz), $CDCl_3$

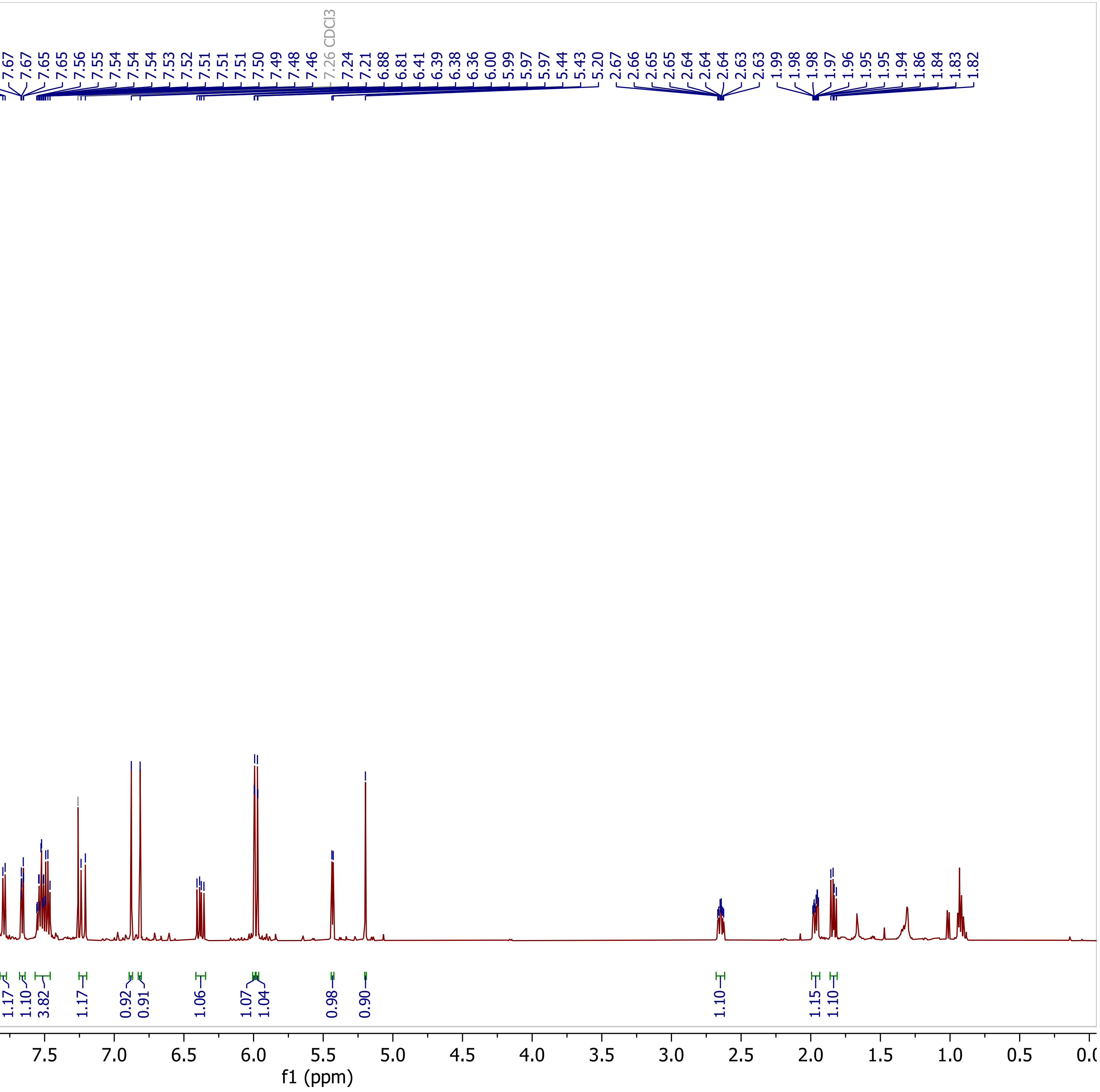

BSSUP1997

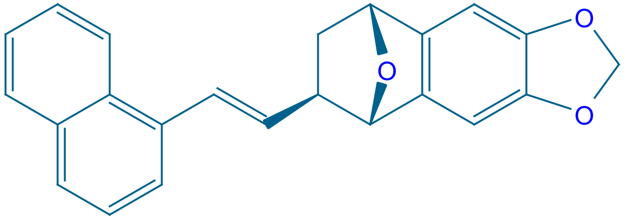

3di, $^{13}C\{^1H\}$ (100 MHz), $CDCl_3$

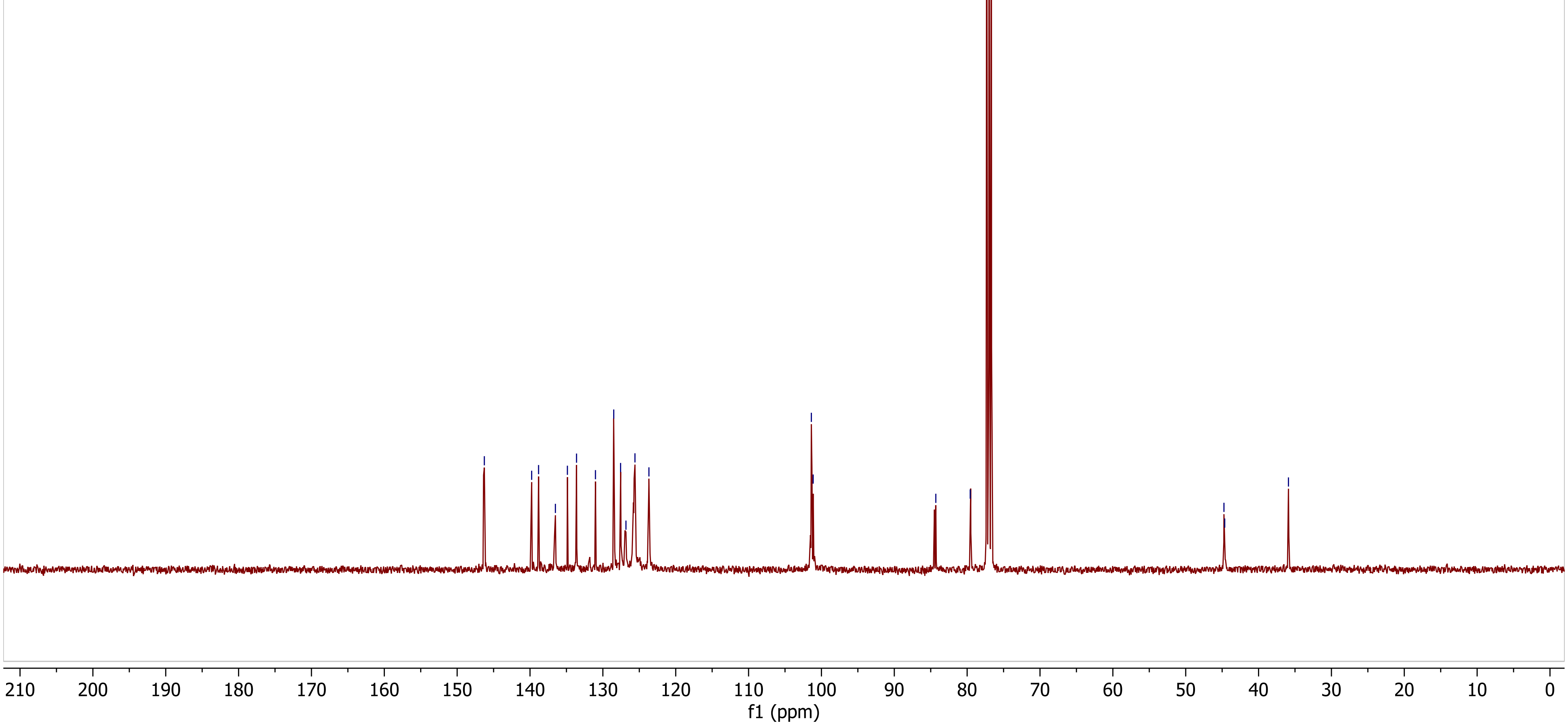

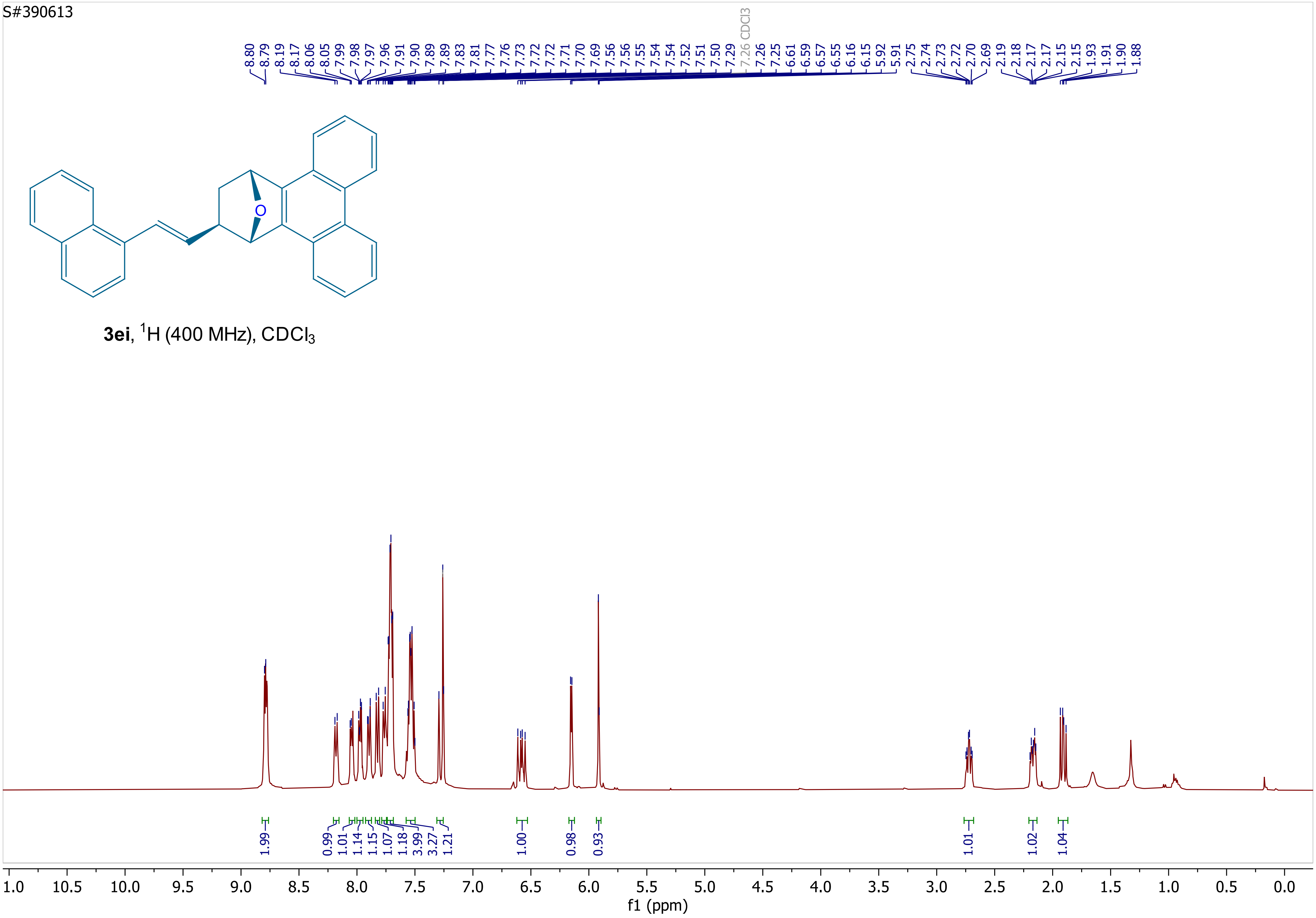
S#390613
8.80 8.79 8.19 8.17 8.06 8.05 7.99 7.98 7.97 7.96 7.91 7.90 7.89 7.89 7.83 7.81 7.77 7.76 7.73 7.72 7.72 7.71 7.70 7.69 7.56 7.56 7.55 7.54 7.54 7.52 7.51 7.50 7.29 7.26 CDCl3 7.26 7.25 6.61 6.59 6.57 6.55 6.16 6.15 5.92 5.91 2.75 2.74 2.73 2.72 2.70 2.69 2.19 2.18 2.17 2.17 2.15 2.15 1.93 1.91 1.90 1.88
O
3ei, ¹H (400 MHz), CDCl3
1.99 0.99 1.01 1.14 1.15 1.07 1.18 3.99 3.27 1.21 1.00 0.98 0.93 1.01 1.02 1.04
1.0 10.5 10.0 9.5 9.0 8.5 8.0 7.5 7.0 6.5 6.0 5.5 5.0 4.5 4.0 3.5 3.0 2.5 2.0 1.5 1.0 0.5 0.0
f1 (ppm)

S#519045

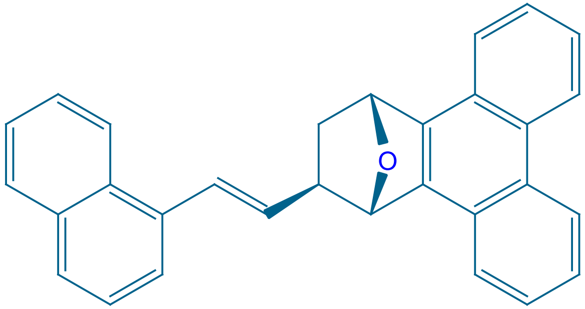

3ei, $^{13}C\{^1H\}$ (100 MHz), $CDCl_3$

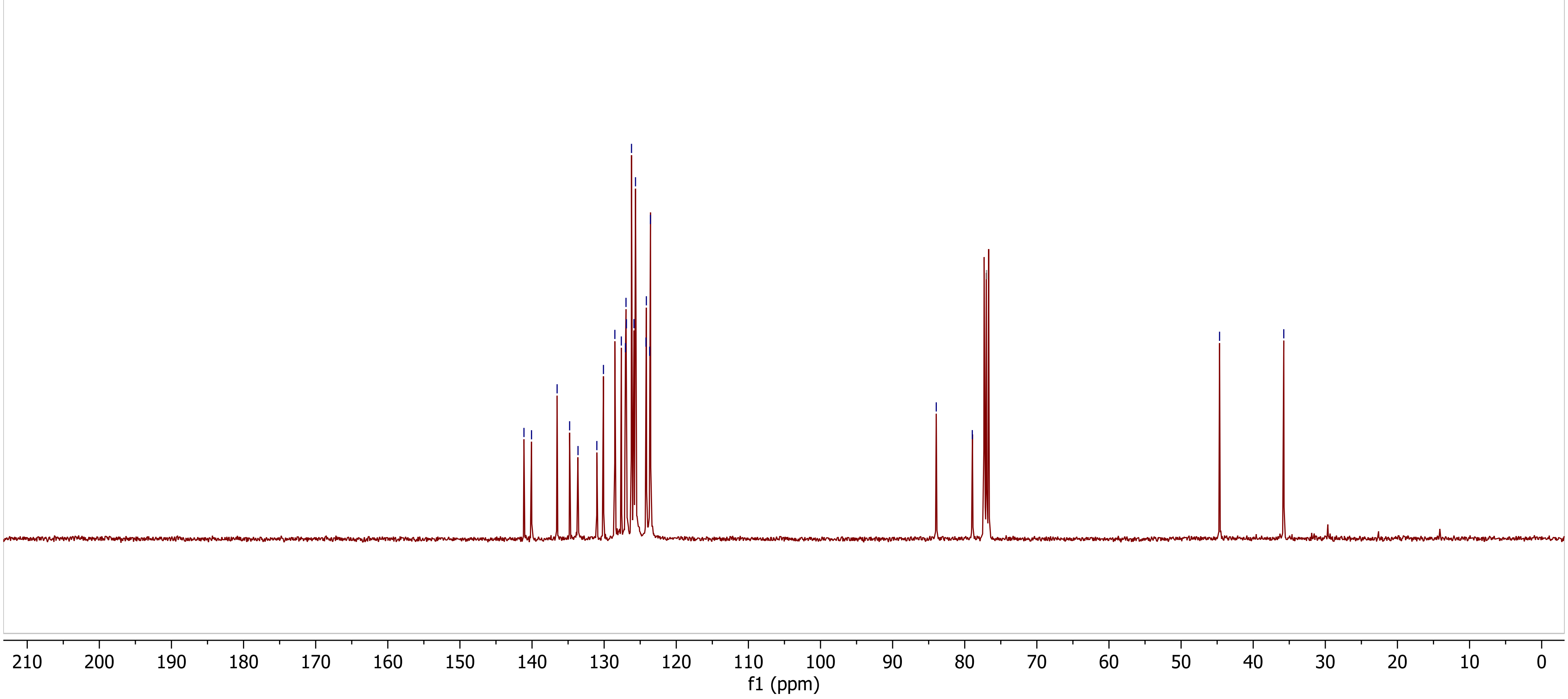

SD/BSSUP2450A

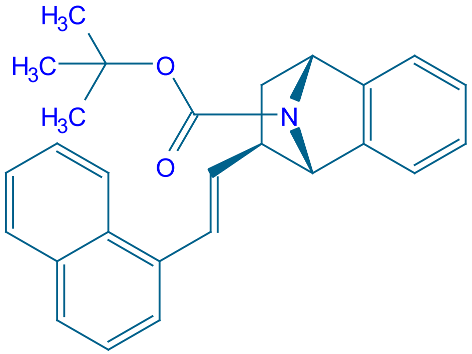

3fi, ^{1}H (400 MHz), CDCl$_3$

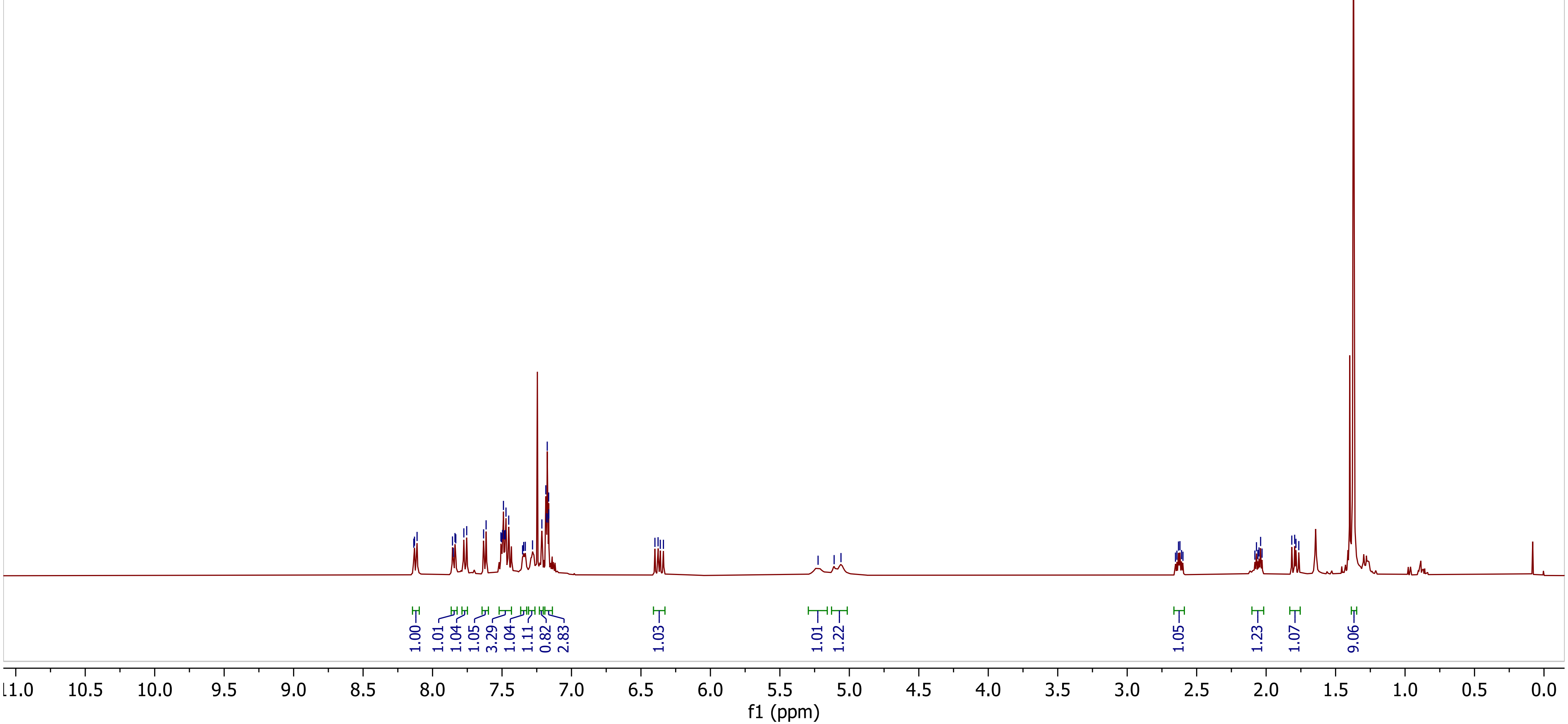

BS/BSSUP2450

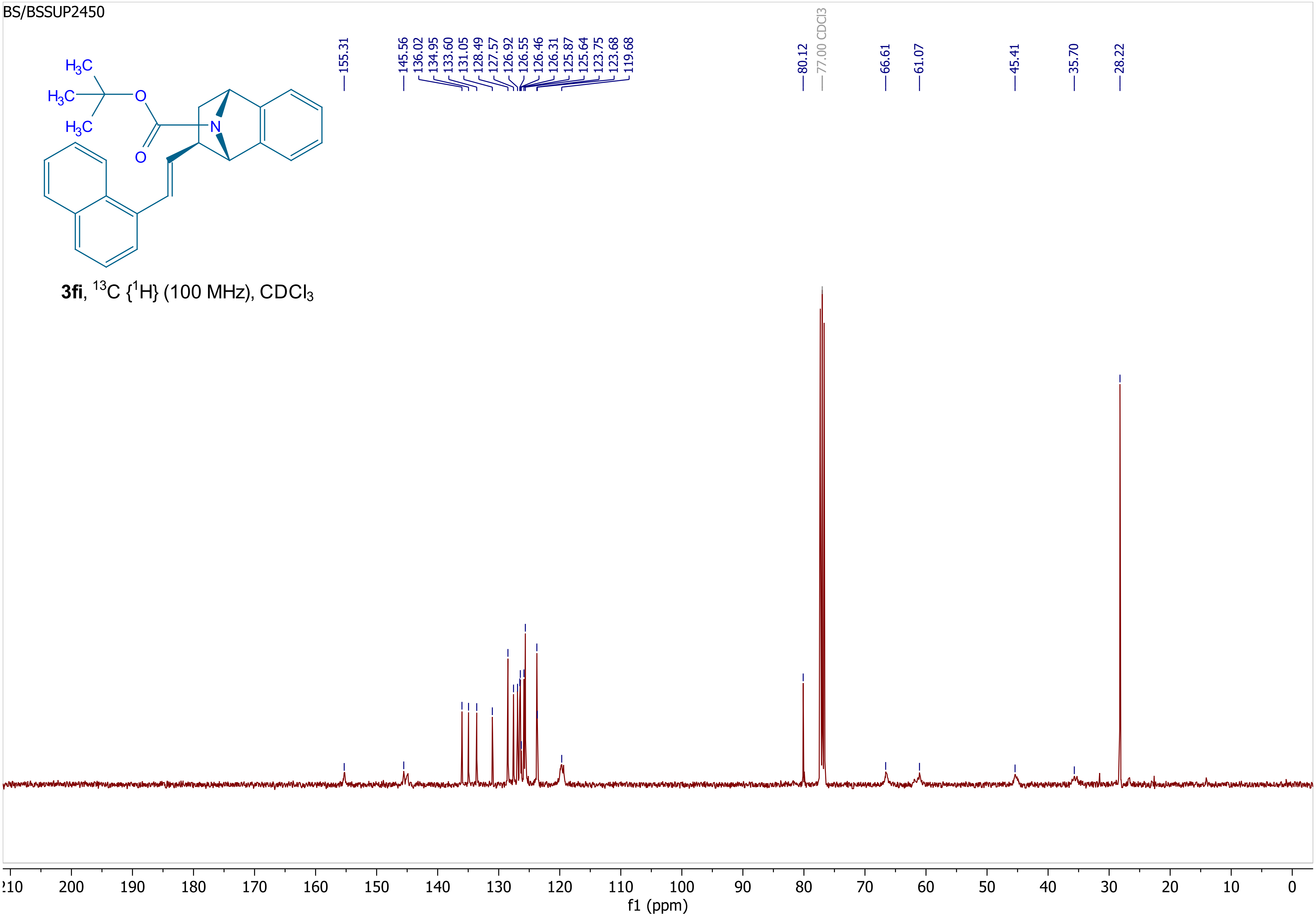

3fi, $^{13}C\ \{^1H\}$ (100 MHz), $CDCl_3$

BSSUP1817

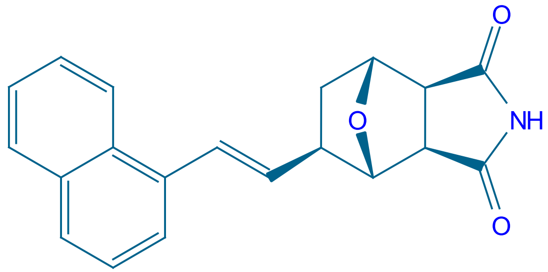

3gi, ^{1}H (400 MHz), $CDCl_3$

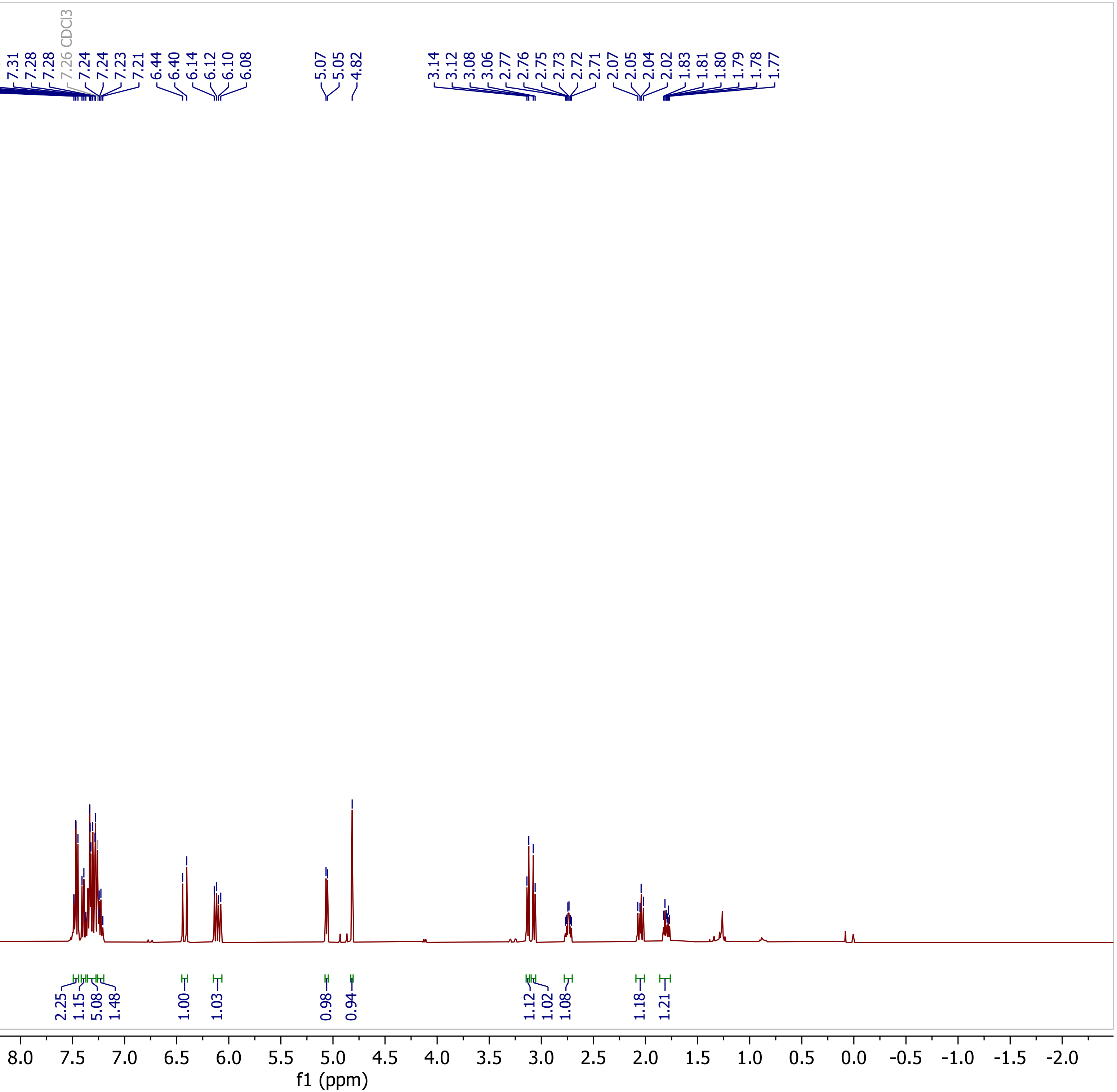

S#396265

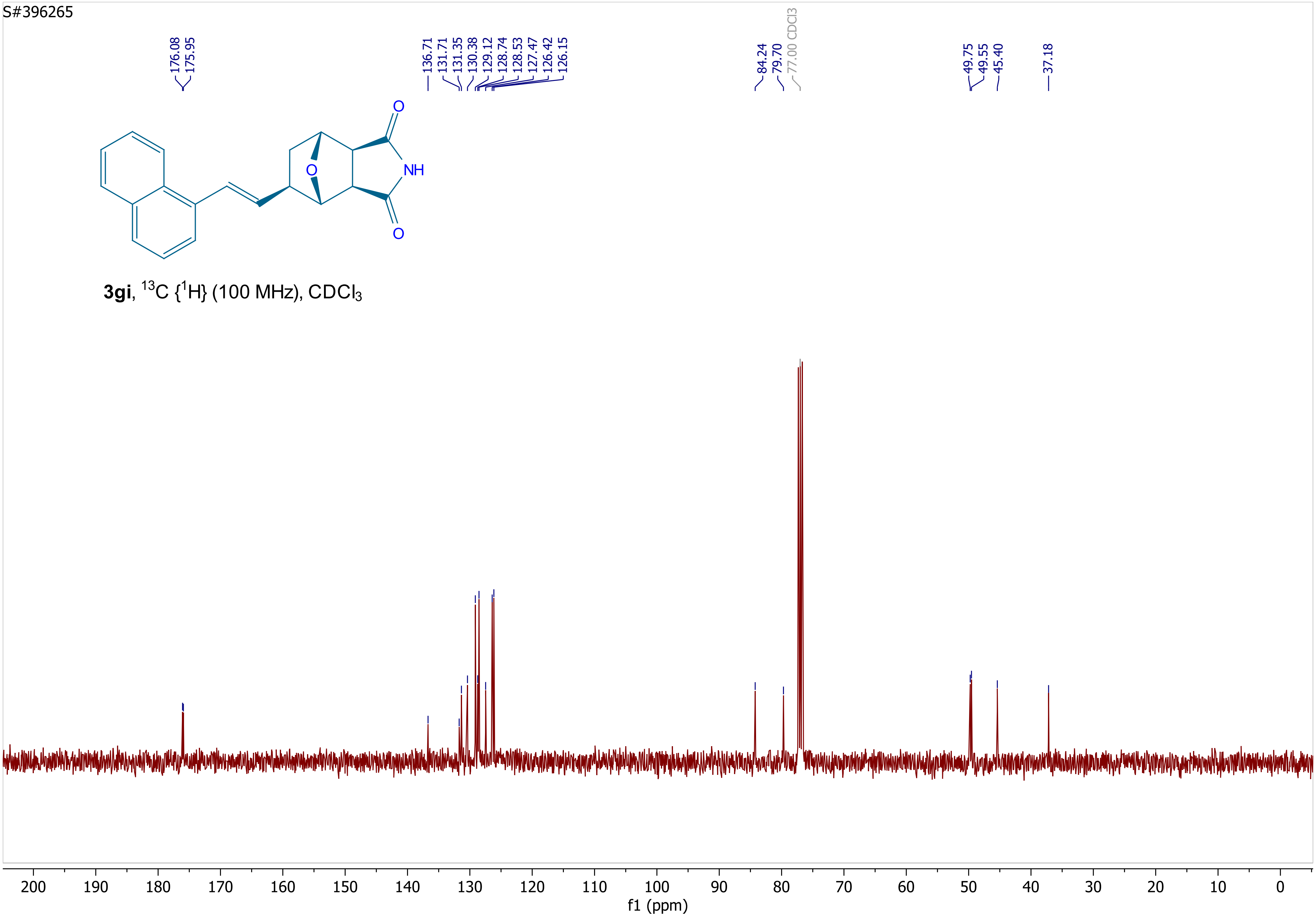

3gi, $^{13}C\ \{^{1}H\}$ (100 MHz), $CDCl_3$

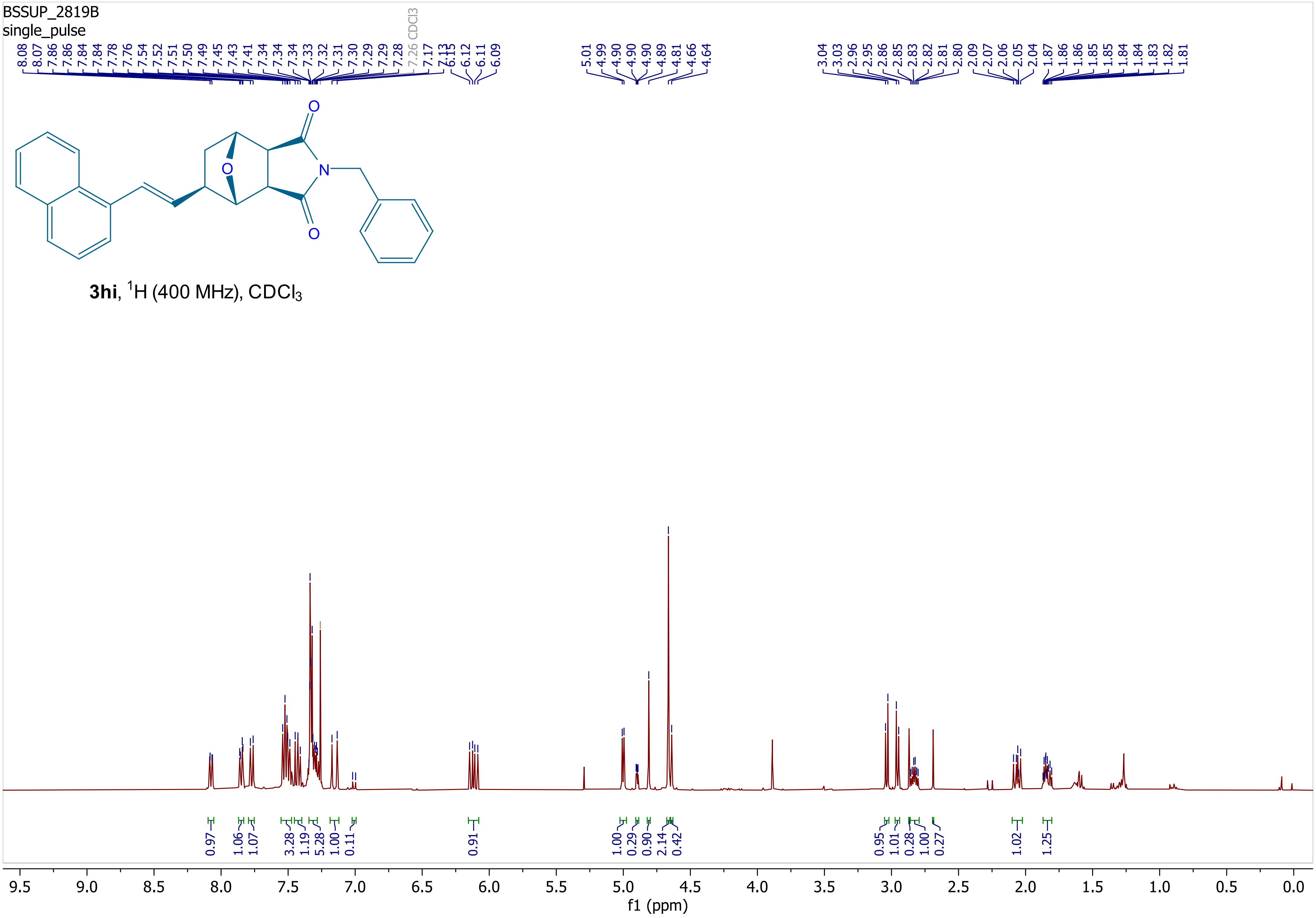

3hi, ^{1}H (400 MHz), $CDCl_3$

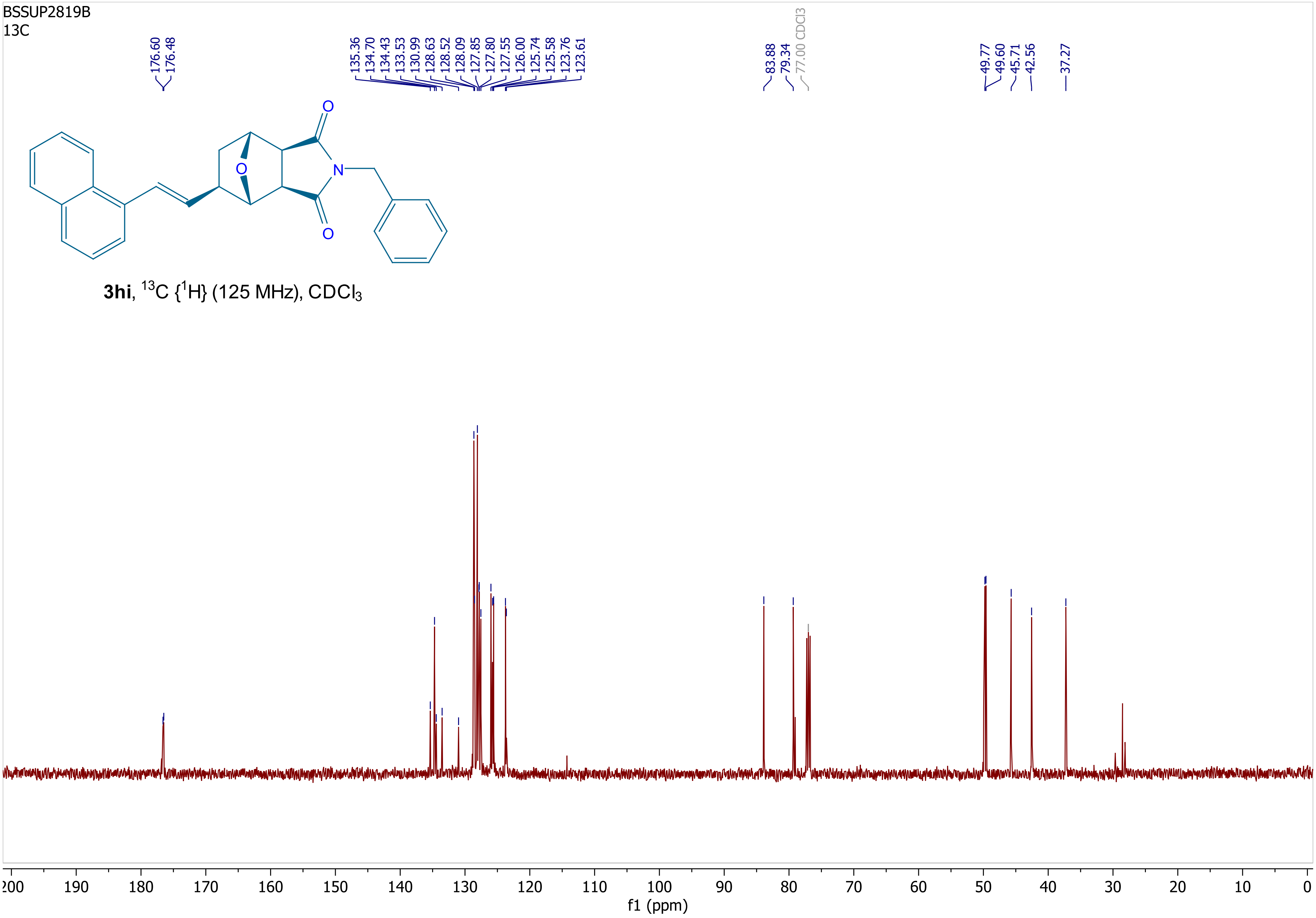

3hi, ^{13}C {^{1}H} (125 MHz), $CDCl_3$

S#603989

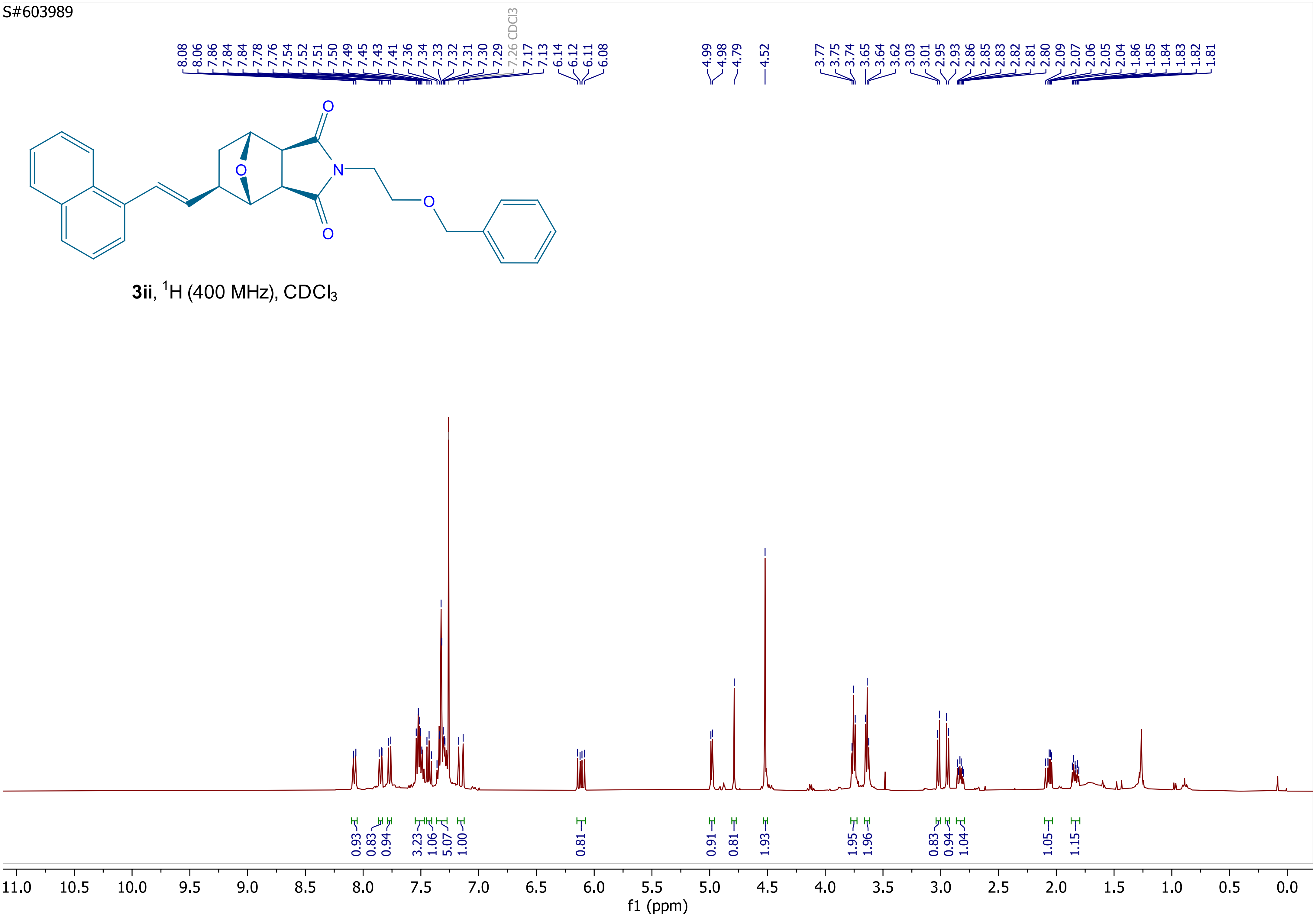

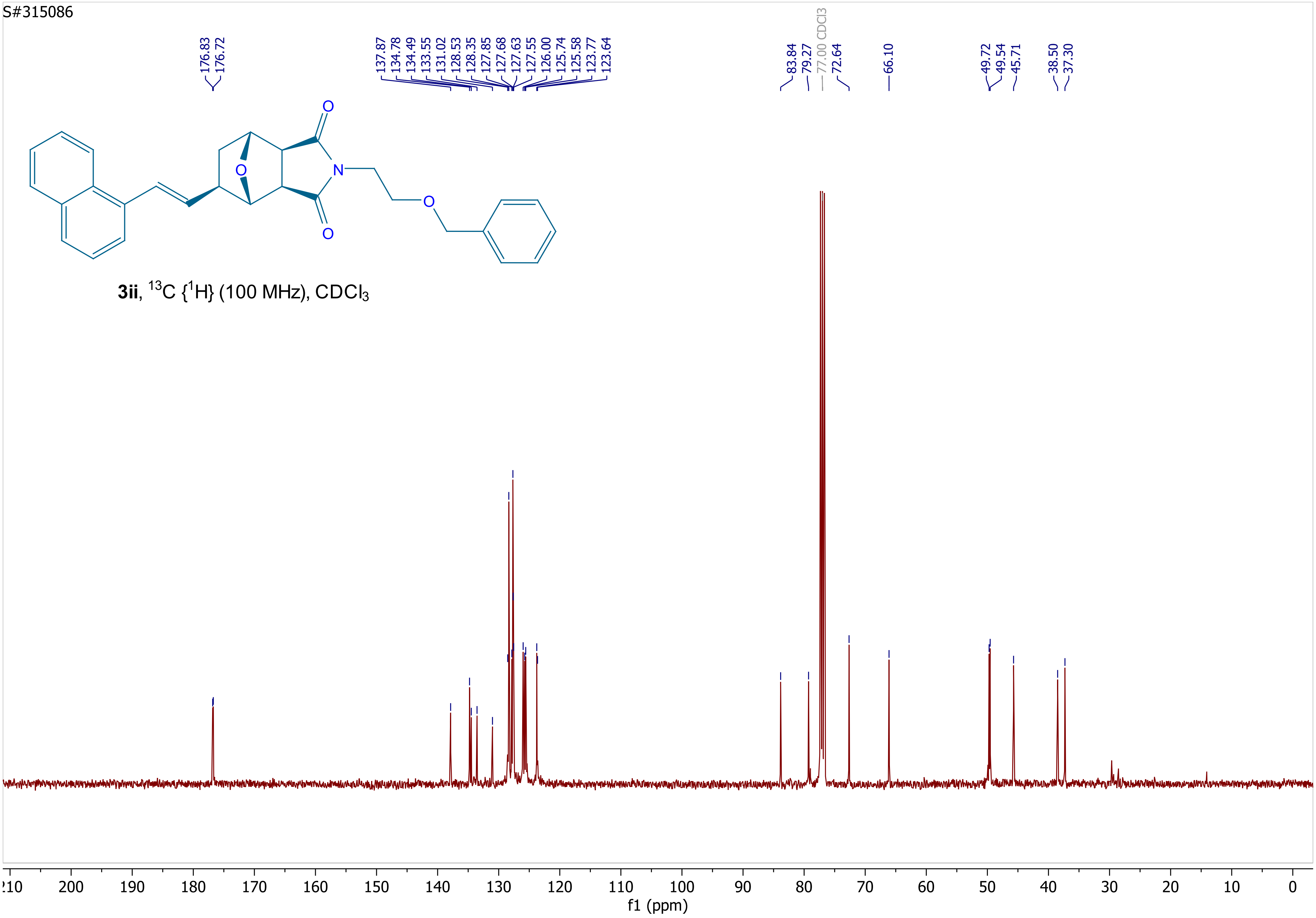
S#315086
176.83
176.72
137.87
134.78
134.49
133.55
131.02
128.53
128.35
127.85
127.68
127.63
127.55
126.00
125.74
125.58
123.77
123.64
83.84
79.27
77.00 CDCl3
72.64
66.10
49.72
49.54
45.71
38.50
37.30
O
O
N
O
O
3ii, 13C {1H} (100 MHz), CDCl3
210
200
190
180
170
160
150
140
130
120
110
100
90
80
70
60
50
40
30
20
10
0
f1 (ppm)

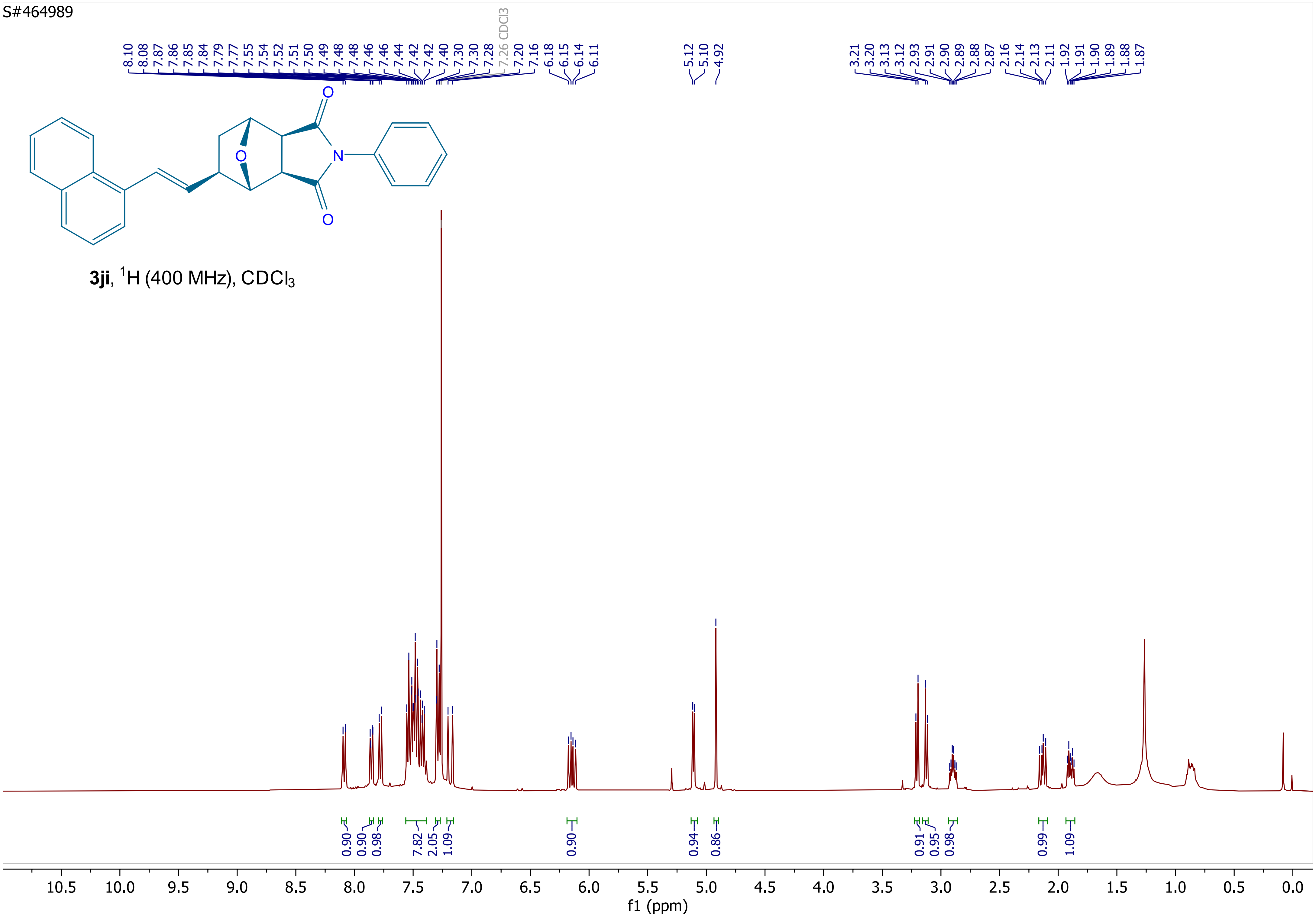

3ji, ^{1}H (400 MHz), $CDCl_3$

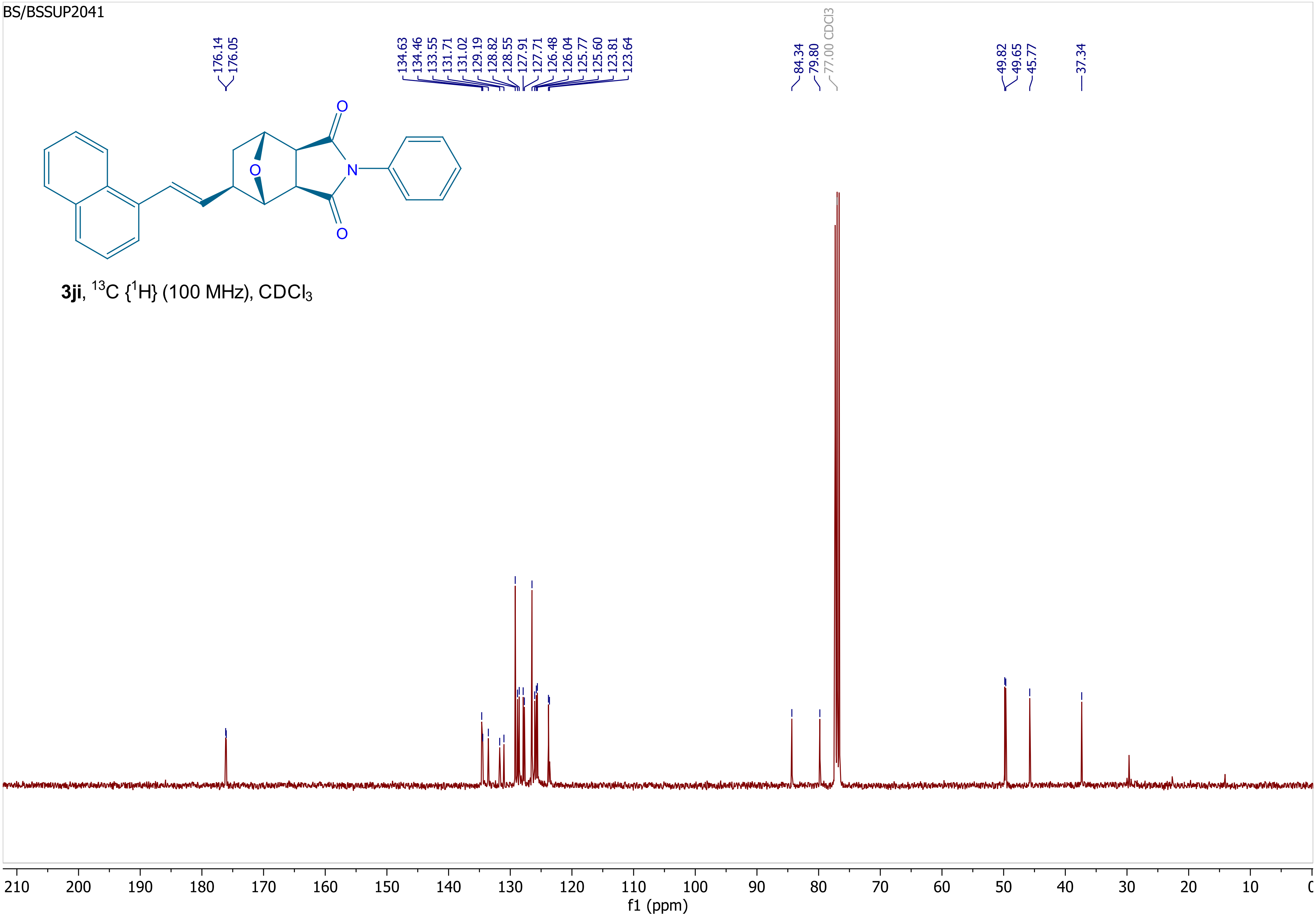

3ji, $^{13}C\{^{1}H\}$ (100 MHz), $CDCl_3$

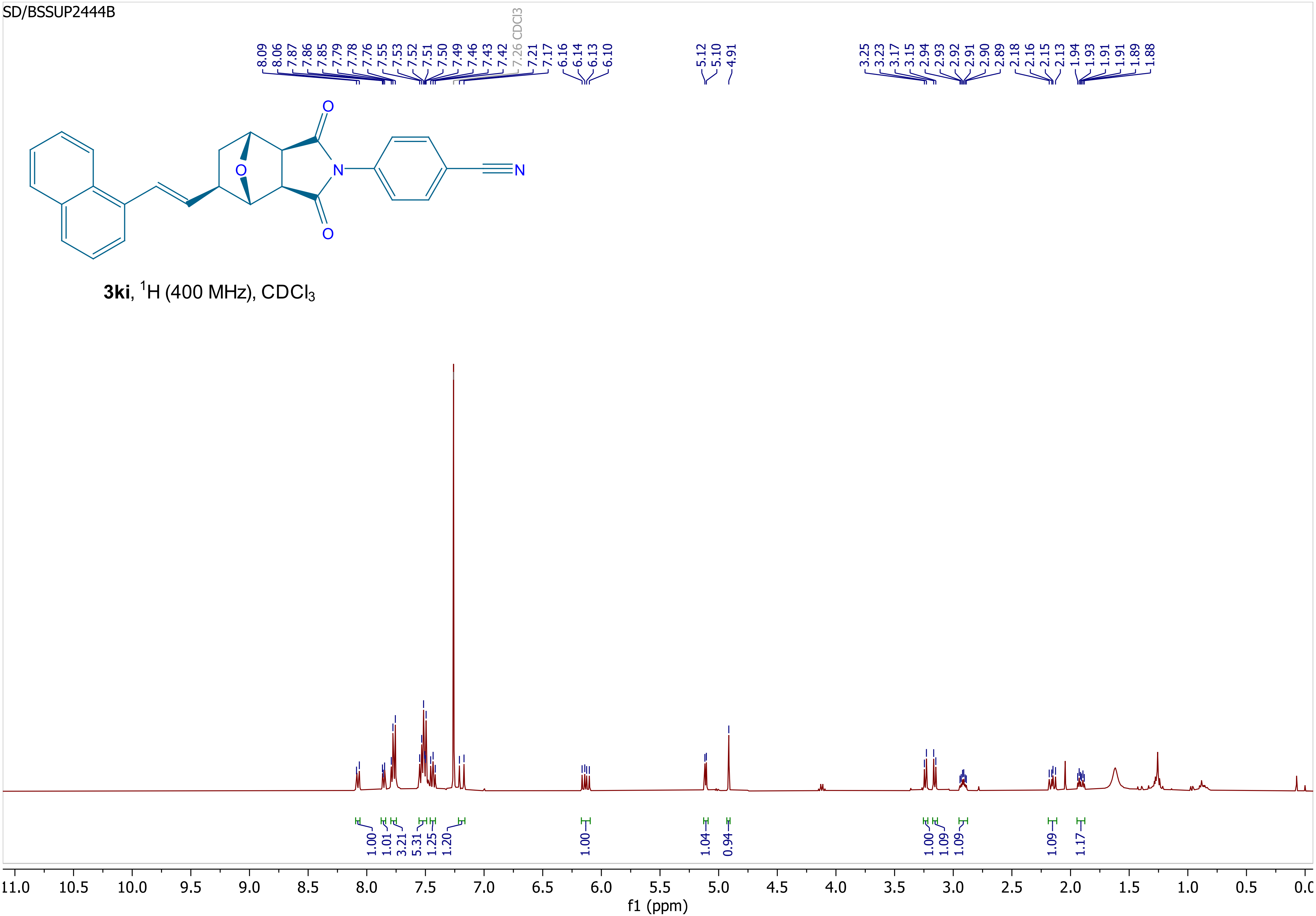
SD/BSSUP2444B
8.09 8.06 7.87 7.86 7.85 7.79 7.78 7.76 7.55 7.53 7.52 7.51 7.50 7.49 7.46 7.43 7.42 7.26 CDCl3 7.21 7.17 6.16 6.14 6.13 6.10
5.12 5.10 4.91
3.25 3.23 3.17 3.15 2.94 2.93 2.92 2.91 2.90 2.89 2.18 2.16 2.15 2.13 1.94 1.93 1.91 1.89 1.88
O
O
N
O
N
3ki, 1H (400 MHz), CDCl3
1.00 1.01 3.21 5.31 1.25 1.20 1.00 1.04 0.94 1.00 1.09 1.09 1.09 1.17
11.0 10.5 10.0 9.5 9.0 8.5 8.0 7.5 7.0 6.5 6.0 5.5 5.0 4.5 4.0 3.5 3.0 2.5 2.0 1.5 1.0 0.5 0.0
f1 (ppm)

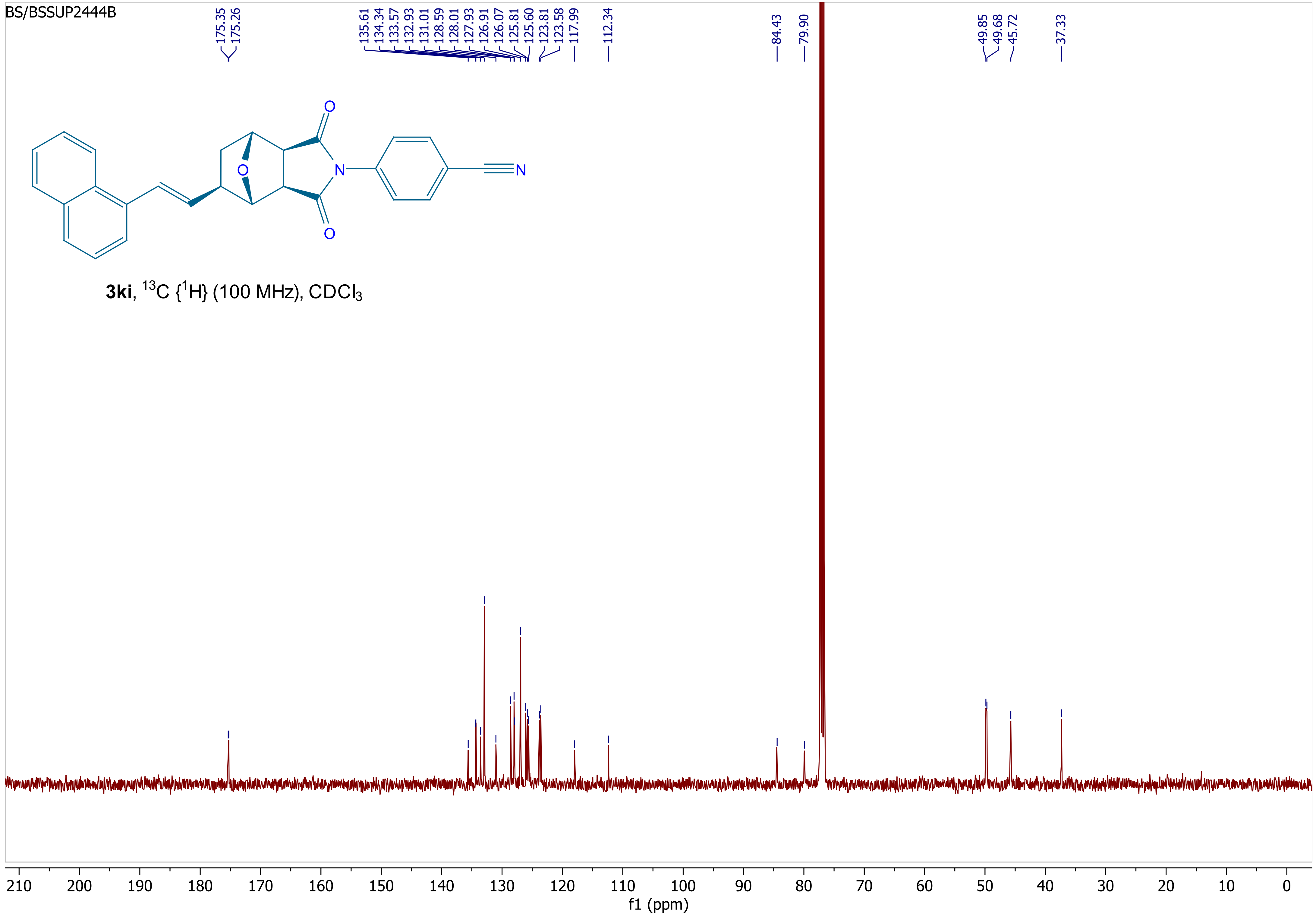
BS/BSSUP2444B
175.35
175.26
135.61
134.34
133.57
132.93
131.01
128.59
128.01
127.93
126.91
126.07
125.81
125.60
123.81
123.58
117.99
112.34
84.43
79.90
49.85
49.68
45.72
37.33
O
O
O
N
N
3ki, 13C {1H} (100 MHz), CDCl3
210
200
190
180
170
160
150
140
130
120
110
100
90
80
70
60
50
40
30
20
10
0
f1 (ppm)

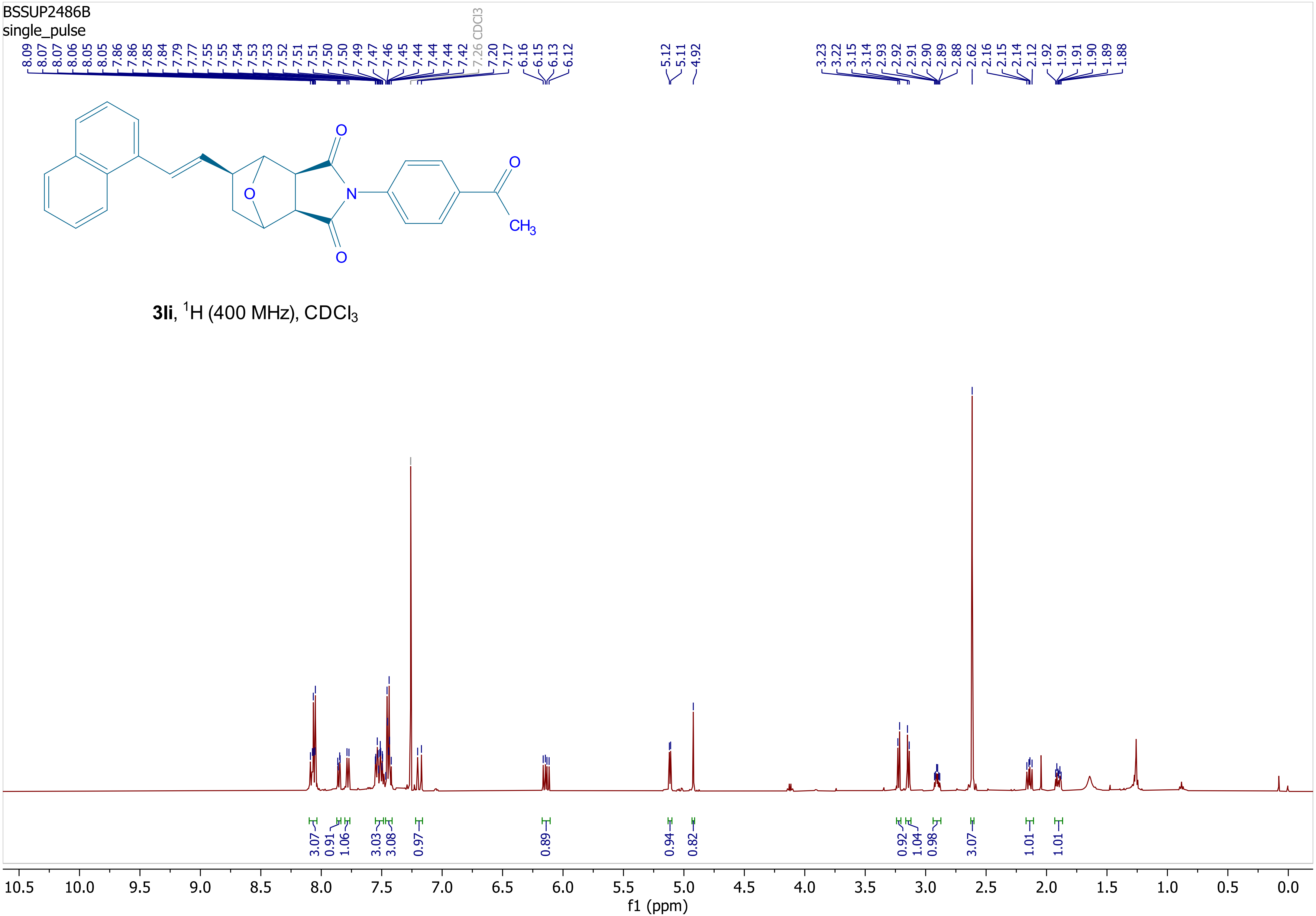

3li, ^{1}H (400 MHz), $CDCl_3$

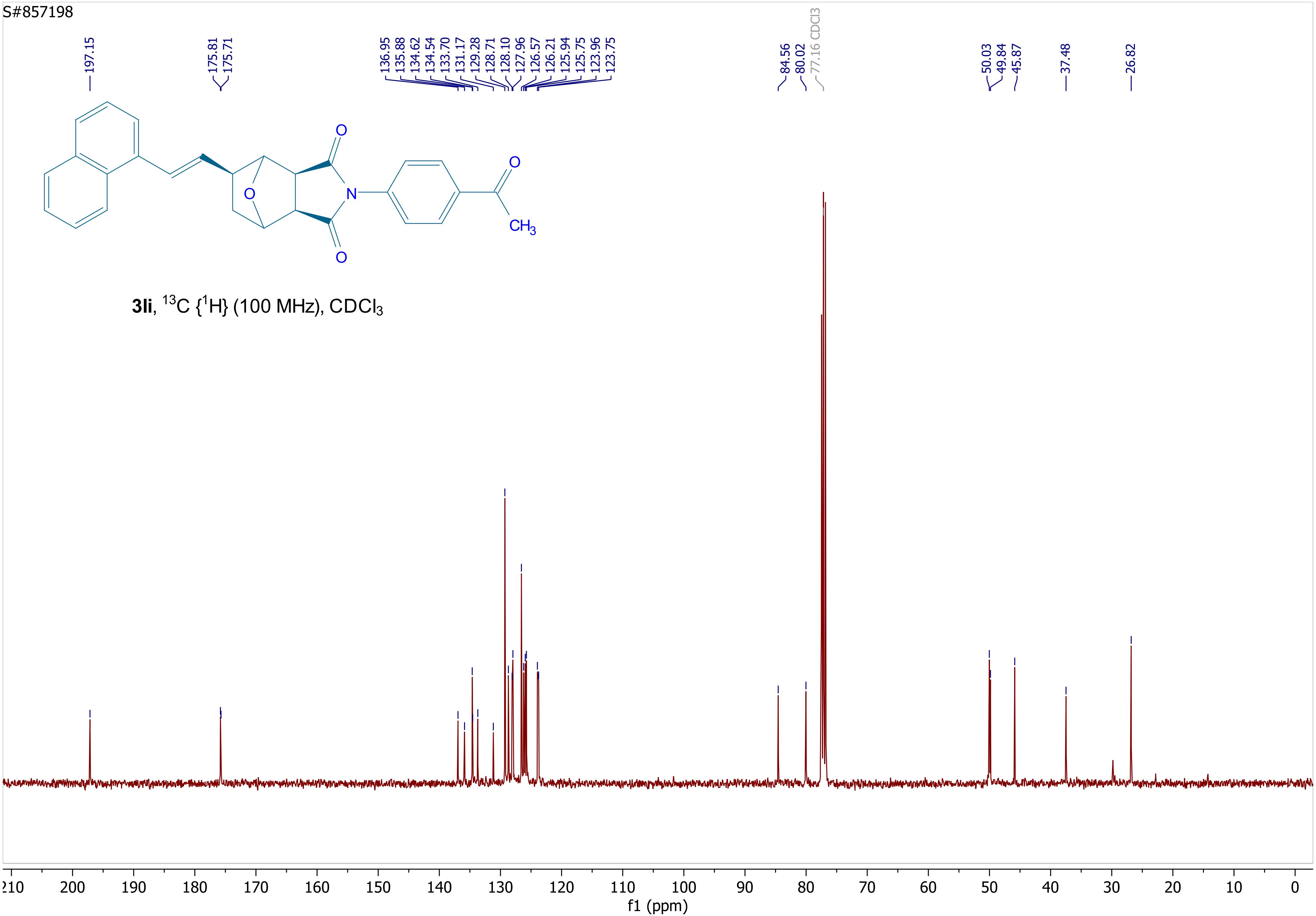
S#857198
197.15
175.81
175.71
136.95
135.88
134.62
134.54
133.70
131.17
129.28
128.71
128.10
127.96
126.57
126.21
125.94
125.75
123.96
123.75
84.56
80.02
77.16 CDCl3
50.03
49.84
45.87
37.48
26.82
O
O
O
N
O
CH3
3li, 13C {1H} (100 MHz), CDCl3
210 200 190 180 170 160 150 140 130 120 110 100 90 80 70 60 50 40 30 20 10 0
f1 (ppm)

BSSUP_2462
single_pulse

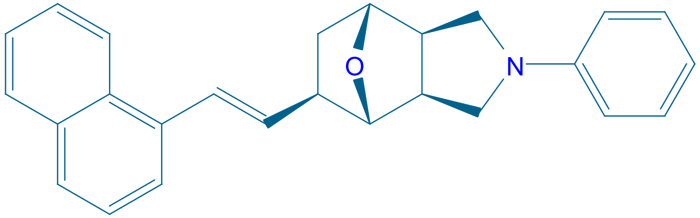

5ji, ^{1}H (500 MHz), $CDCl_3$

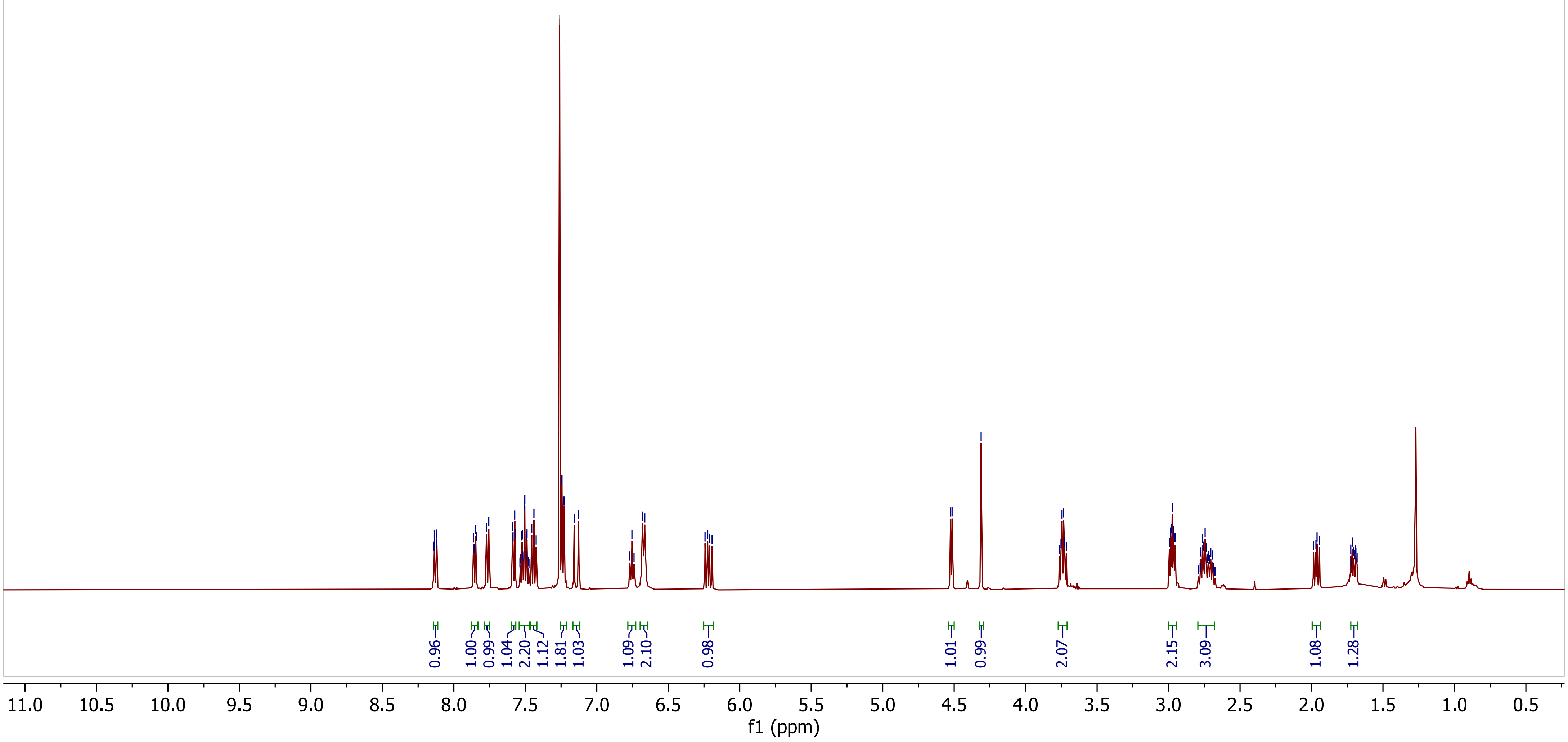

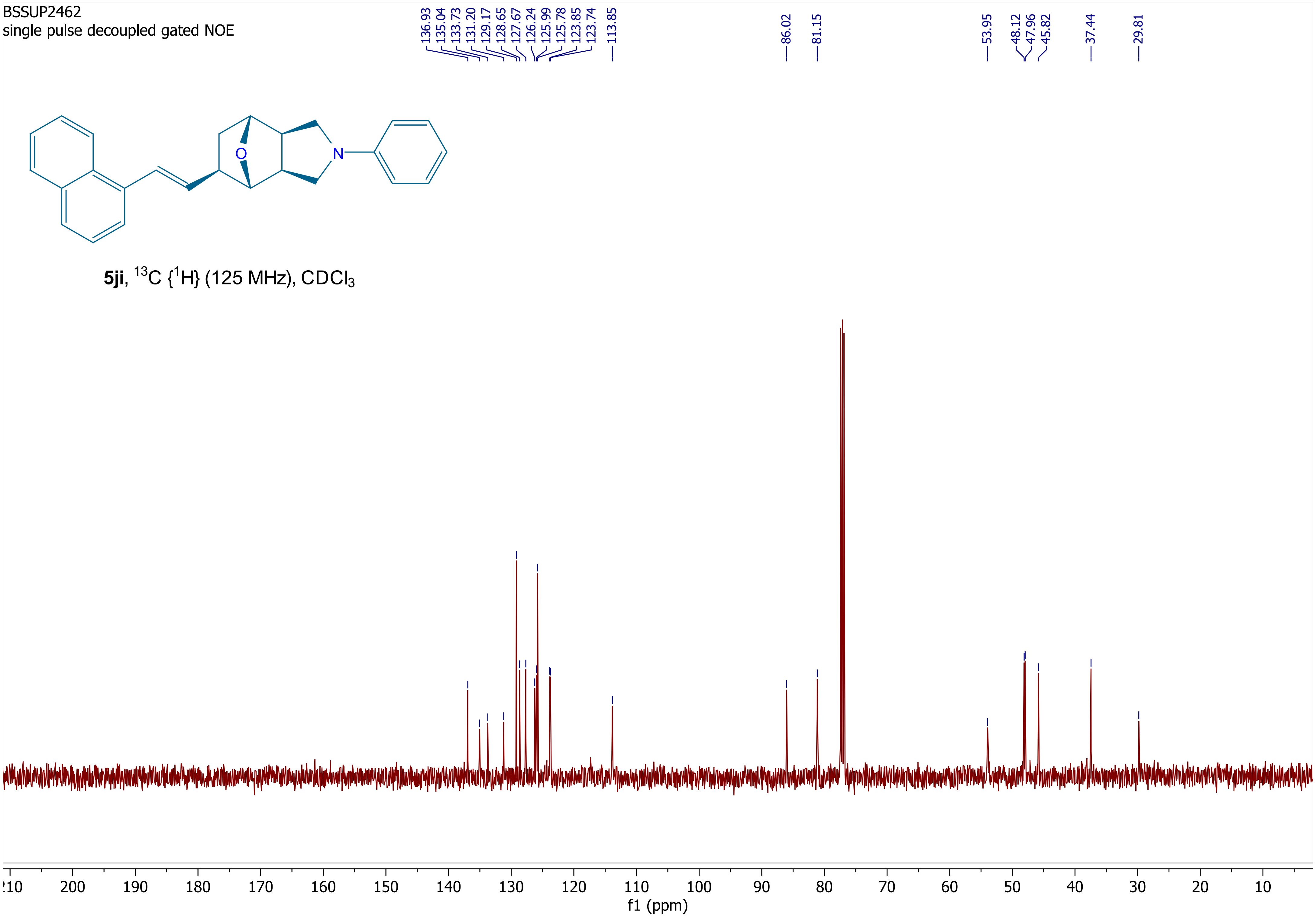

5ji, $^{13}C\ \{^{1}H\}$ (125 MHz), $CDCl_3$

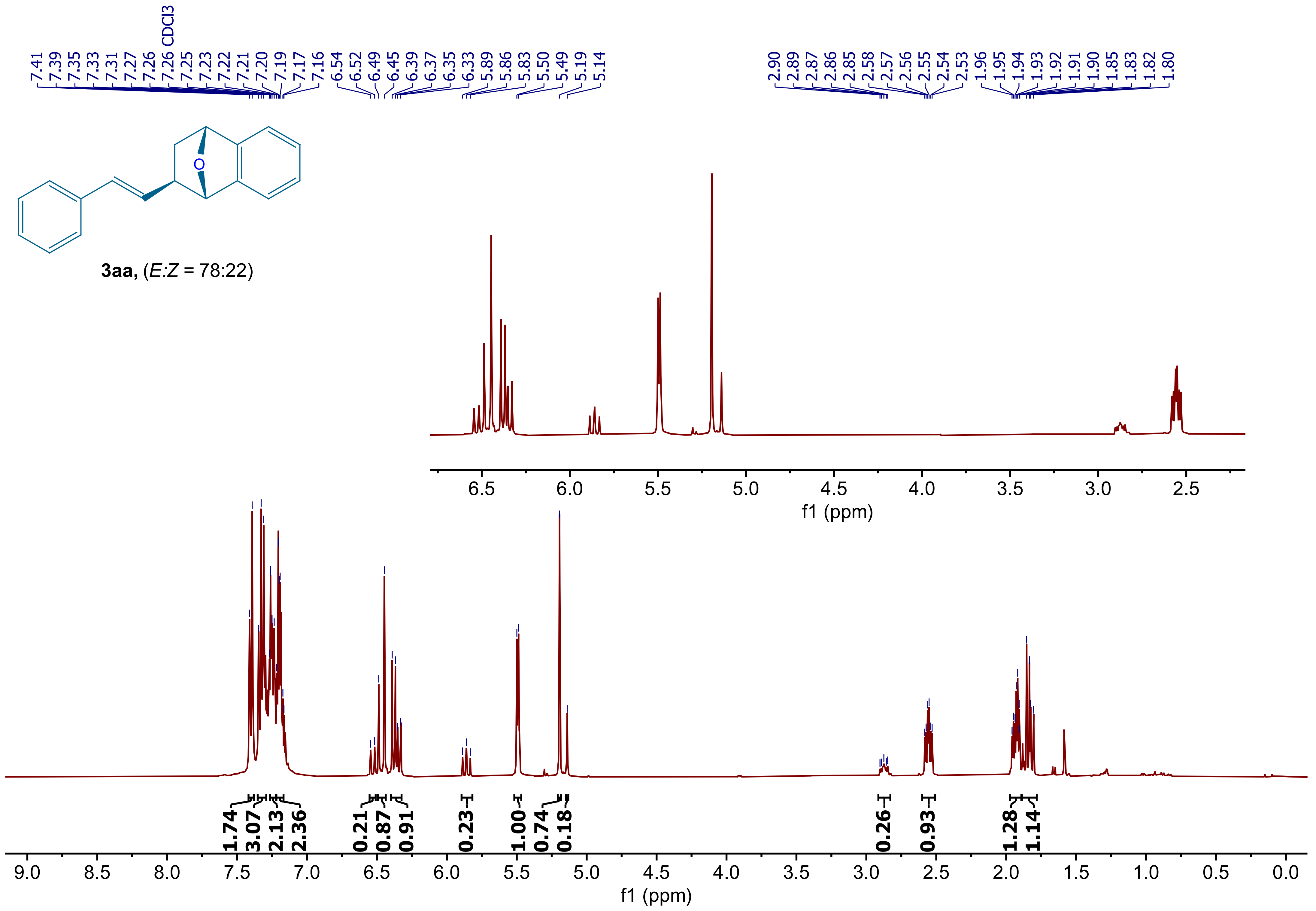

3aa, (E:Z = 78:22)
f1 (ppm)

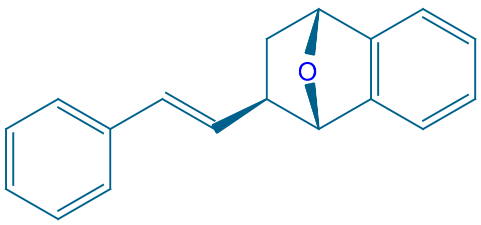

3aa, (*E*:*Z* = 78:22)

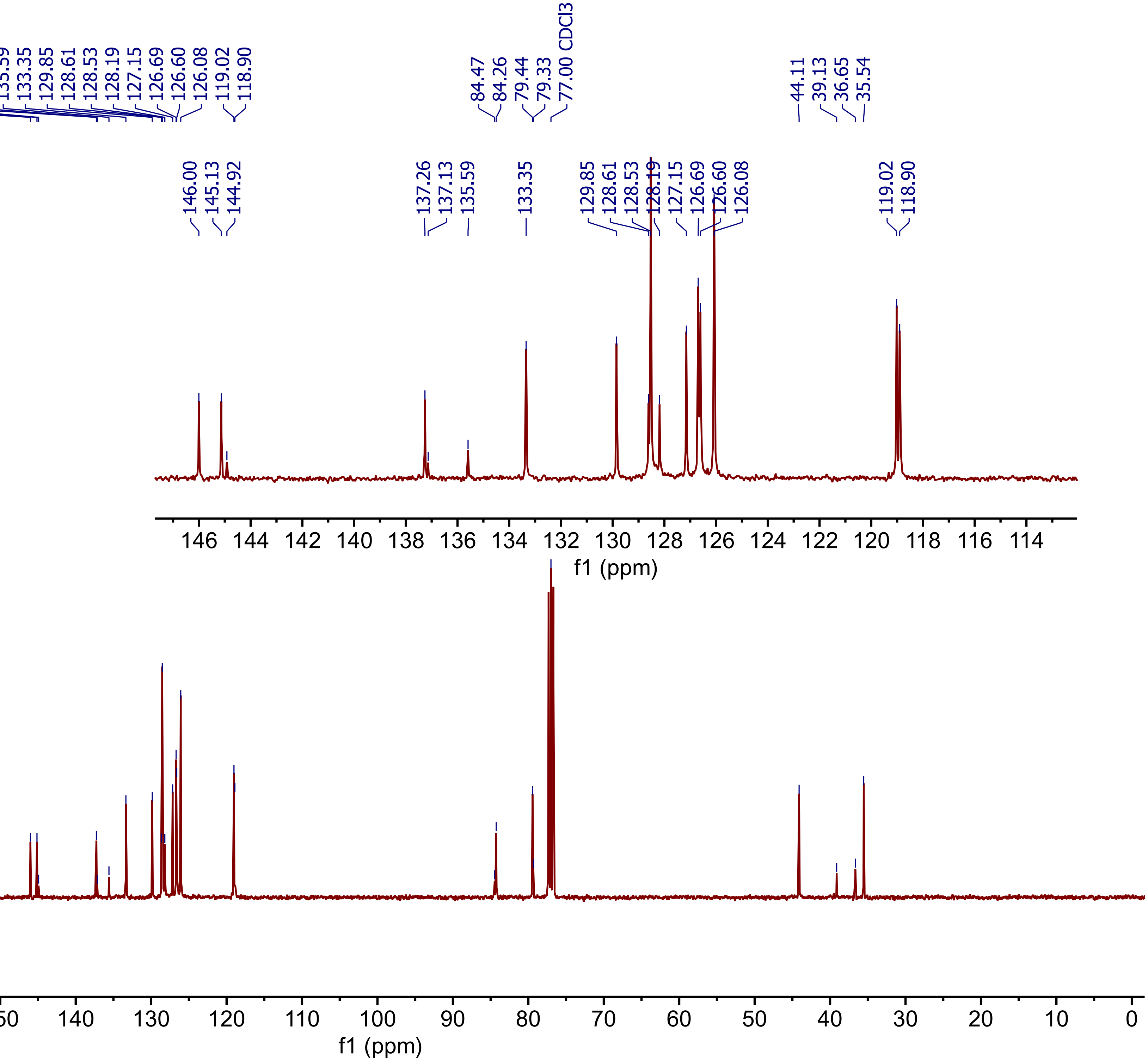

146.00
145.13
144.92
137.26
137.13
135.59
133.35
129.85
128.61
128.53
128.19
127.15
126.69
126.60
126.08
119.02
118.90
84.47
84.26
79.44
79.33
77.00 CDCl3
44.11
39.13
36.65
35.54
146 144 142 140 138 136 134 132 130 128 126 124 122 120 118 116 114
f1 (ppm)
00 190 180 170 160 150 140 130 120 110 100 90 80 70 60 50 40 30 20 10 0
f1 (ppm)

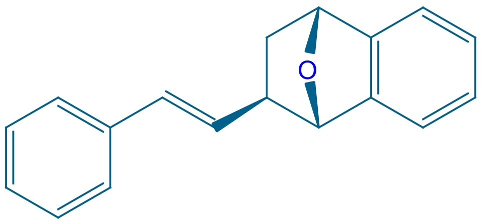

3aa, (*Z*:*E* = 80:20)

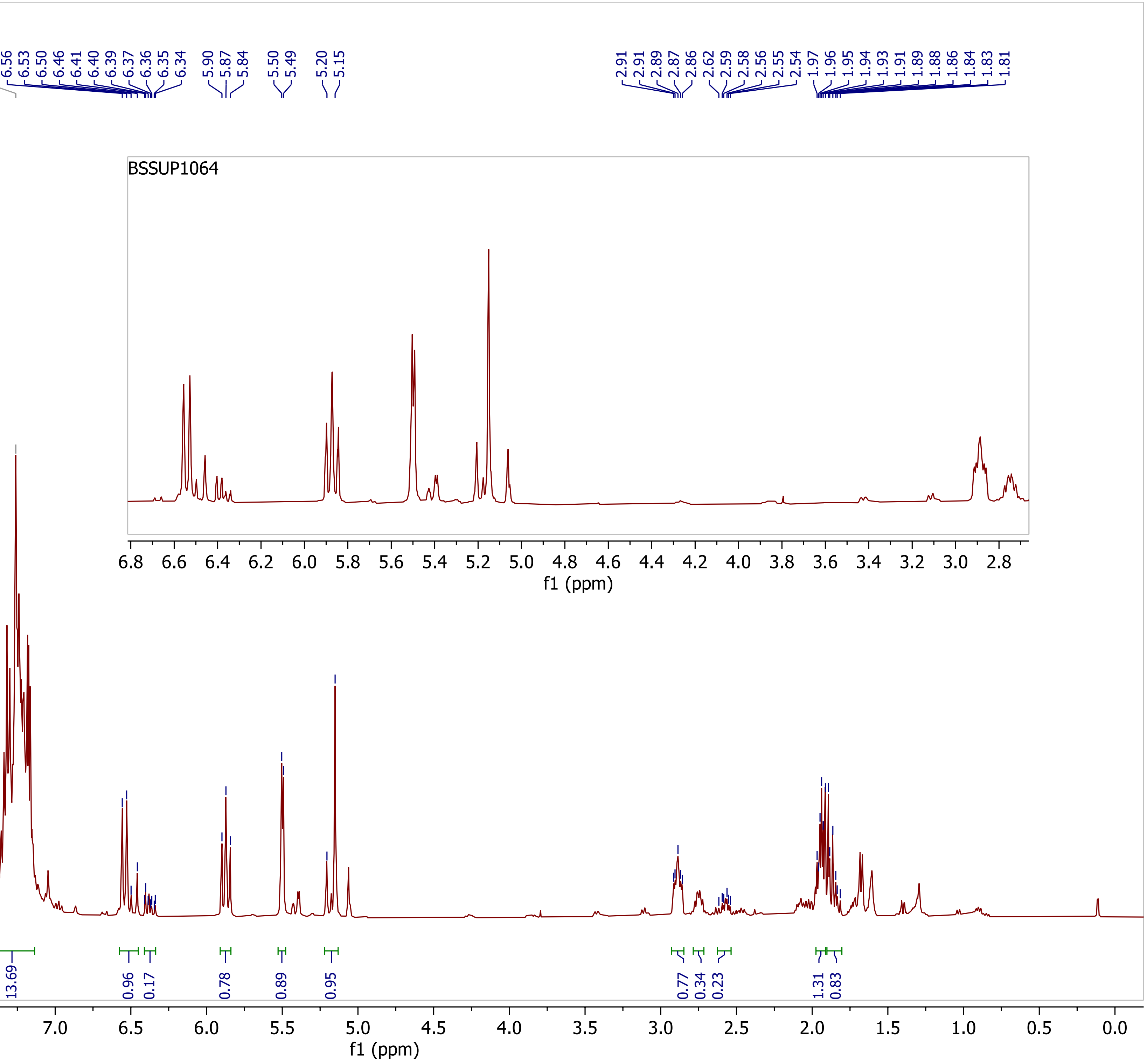
BSSUP1064
7.26 CDCl3
6.56 6.53 6.50 6.46 6.41 6.40 6.39 6.37 6.36 6.35 6.34
5.90 5.87 5.84
5.50 5.49
5.20 5.15
2.91 2.91 2.89 2.87 2.86 2.62 2.59 2.58 2.56 2.55 2.54 1.97 1.96 1.95 1.94 1.93 1.91 1.89 1.88 1.86 1.84 1.83 1.81
13.69 0.96 0.17 0.78 0.89 0.95 0.77 0.34 0.23 1.31 0.83
BSSUP1064
6.8 6.6 6.4 6.2 6.0 5.8 5.6 5.4 5.2 5.0 4.8 4.6 4.4 4.2 4.0 3.8 3.6 3.4 3.2 3.0 2.8
f1 (ppm)
9.5 9.0 8.5 8.0 7.5 7.0 6.5 6.0 5.5 5.0 4.5 4.0 3.5 3.0 2.5 2.0 1.5 1.0 0.5 0.0
f1 (ppm)

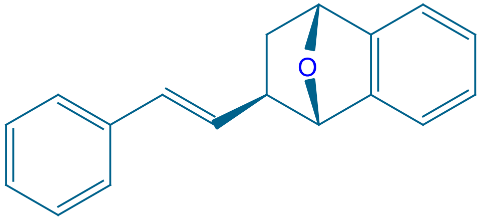

3aa, (*Z*:*E* = 80:20)

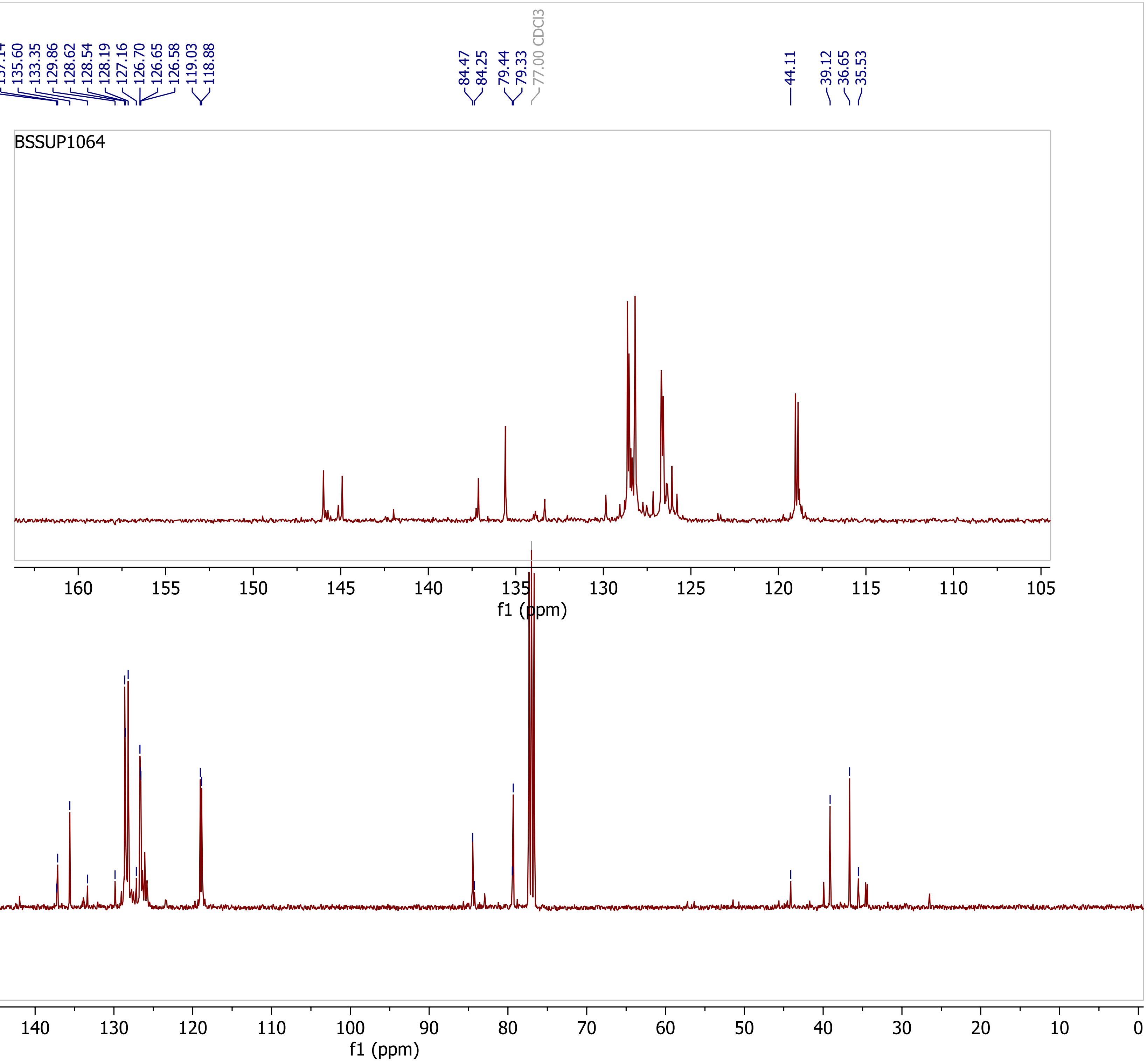
BSSUP1064
145.99
145.14
144.92
137.26
137.14
135.60
133.35
129.86
128.62
128.54
128.19
127.16
126.70
126.65
126.58
119.03
118.88
84.47
84.25
79.44
79.33
77.00 CDCl3
44.11
39.12
36.65
35.53
BSSUP1064
160 155 150 145 140 135 130 125 120 115 110 105
f1 (ppm)
190 180 170 160 150 140 130 120 110 100 90 80 70 60 50 40 30 20 10 0
f1 (ppm)
